# Prediction analysis of carbon emission in China’s electricity industry based on the dual carbon background

**DOI:** 10.1371/journal.pone.0302068

**Published:** 2024-05-17

**Authors:** Ze-qun Ding, Hong-qing Zhu, Wei-ye Zhou, Zhi-gang Bai

**Affiliations:** 1 School of Environmental Science and Engineering, Tianjin University, Tianjin, P.R. China; 2 State Nuclear Electric Power Planning Design & Research Institute, Beijing, P.R. China; 3 Urban Gas & Heat Research Institute, North China Municipal Engineering Design & Research Institute Co., Ltd, Tianjin, P.R. China; Czestochowa University of Technology: Politechnika Czestochowska, POLAND

## Abstract

The electric power sector is the primary contributor to carbon emissions in China. Considering the context of dual carbon goals, this paper examines carbon emissions within China’s electricity sector. The research utilizes the LMDI approach for methodological rigor. The results show that the cumulative contribution of economies scale, power consumption factors and energy structure are 114.91%, 85.17% and 0.94%, which contribute to the increase of carbon emissions, the cumulative contribution of power generation efficiency and ratio of power dissipation to generation factor are -19.15% and -0.01%, which promotes the carbon reduction. The decomposition analysis highlights the significant influence of economic scale on carbon emissions in the electricity industry, among the seven factors investigated. Meanwhile, STIRPAT model, Logistic model and GM(1,1) model are used to predict carbon emissions, the average relative error between actual carbon emissions and the predicted values are 0.23%, 8.72% and 7.05%, which indicates that STIRPAT model is more suitable for medium- to long-term predictions. Based on these findings, the paper proposes practical suggestions to reduce carbon emissions and achieve the dual carbon goals of the power industry.

## Introduction

### Background

The increasing carbon emissions have raised several questions [1]. The beginning of China’s reform and open in 1978 has led to the second-largest economy global with sustained economic growth [2]. However, this has also made China one of the biggest energy consumers in the world, surpassing the United States [3]. At the same time, it also became the world’s biggest carbon dioxide emitter [4, 5]. Given the need to decrease greenhouse gases emissions as well as address global energy crisis, China urgently needs to reduce emissions and save energy.

As a responsible developing country, China attaches great importance to reducing carbon emissions and pays great attention to developing a low-carbon economy with low emissions, low energy consumption, and low pollution. China has given many commitments to reduce emissions at world conferences and has incorporated the goal of climate change action into its national socio-economic development plan by formulating a low-carbon development route and strategy in long-term. China is actively implementing " National Climate Change Adaptation Strategy " [6],"the 13th Five-Year Plan for comprehensive work on energy conservation and emission Reduction " [7], and various provincial special plans. These plans can adjust and improve the working pattern of climate change in real-time and guide the gradual decomposition of the target task of climate change based on the carbon emission index. "China’s own national initiative to address climate change action " [8], published in 2015, identifies the autonomous target of action for 2030: the carbon emissions can reach their peak in 2030, as soon as possible afterwards; compared to 2005 carbon intensity will decrease by 60%-65%, and non-fossil energy could occupy about 20 percent of the basic energy consumption.

Among all industries, the power industry is the largest contributor to fossil fuel consumption and carbon emissions. Therefore, achieving China’s dual carbon goals requires the power industry to play a core role in carbon reduction.

## Literature review

By reviewing the existing literature, it is evident that in recent years, many scholars in different countries have used factor decomposition method (DA) to investigate driving factors that affecting carbon emissions in various regions, including transportation, construction, and chemical industries [9–11]. Wang et al. [12] use the temporal LMDI model to evaluate the driving factors of carbon emissions and the spatial LMDI model to explore regional differences. Wu et al. [13] employed LMDI model to analyze the driving factors behind China’s power industrial carbon emission changes from 2000 to 2018. Additionally, they simulated the various scenarios evolution trend of carbon emissions by Monte Carlo algorithm. Similarly, Liu et al. [14] built a carbon emissions decomposition analysis by using the LMDI method for Beijing, Tianjin, Shanghai, and Chongqing in China.

Ehrlich and Holden were the first to propose "IPAT" equation, which reflects that effect on the environment from the population. The equation consists of environmental impact (I), population (P), wealth per capita (A), as well as the environmental destruction technical level (T), represented as "I = PAT." Building upon IPAT, York developed the STIRPAT model, widely used for carbon emission forecasting. Liu et al. [15] conducted a comparative analysis of carbon emissions influencing factors and predicted carbon peak and carbon neutrality scenarios in Tianjin under baseline, low-carbon, and ultra-low-carbon scenarios. Jia and Li [16] also employed the STIRPAT model to forecast carbon emissions in Jiangsu province.

The Logistic Model has been extensively utilized by scholars to predict carbon emissions. Ge et al. [17] applied Logistic predict model to analyze and forecast industrial carbon emissions in Tianjin. Zheng et al. [18] forecasted the growth of power system carbon emissions in Fujian province, China. The grey forecast theory also has been employed widely for the analysis, modeling, and prediction of grey systems. Numerous scholars have successfully utilized the grey forecast model (GM) model to predicted carbon emissions. Zhu et al. [19] employed GM model to predict industrial carbon emissions in Tianjin. He et al. [20] applied GM model to predict carbon emissions in Hebei province. Ren and Gu [21] predicted energy consumption and analyzed energy structure using the GM model for China. Jia et al. [22]. analyzed the major drivers of the ecological footprint using the STIRPAT model and the PLS method—A case study in Henan Province, China.

However, in recent years, there have been few reports on predicting carbon dioxide emissions in the entire Chinese power industry. Some of the only predictions have only been made using 1–2 methods and have not been combined with the current dual carbon background, so the predicted results and suggestions may also have certain shortcomings. In this research, the decomposition analysis and a forecast method are used to study carbon emissions in China’s electricity industry based on the dual carbon background. Furthermore, this paper also provides insights into the current situation.

## Methodology

### Improved LMDI decomposition model

The LMDI model is presented in this paper with improvements. The gross effect can be decomposed into seven parts: ΔCes, representing the effect of energy structural adjustment means; ΔCcr, representing the effect of changes in power generation efficiency; ΔCs, representing the effect of structural adjustment of the electricity industry; ΔCr, representing the effect of the power consumption-to-generation ratio; ΔCec, representing the effect of the scale of electric power consumption; ΔCy, representing the effect of economic scale; and ΔCemf, representing the effect of carbon emission factors. Therefore, the model is formulated as follows:

$$\Delta C_{\text{tot}}=\Delta C^{T}-\Delta C^{0}$$ (1)

$${\Delta \text{C}}_{\text{tot}}=\Delta C_{es}+\Delta C_{cr}+\Delta C_{s}+\Delta C_{r}+\Delta C_{ec}+\Delta C_{y}+\Delta C_{emf}$$ (2)

$${\Delta \text{C}}_{\text{es}}=\sum_{k}\text{L}\left(C_{k}^{T},C_{k}^{0}\right)\;\text{ln}\left(ES_{k}^{T}/ES_{k}^{0}\right)$$ (3)

$${\Delta \text{C}}_{\text{cr}}=\sum_{k}\text{L}\left(C_{k}^{T},C_{k}^{0}\right)\;\text{ln}\left(CR^{T}/CR^{0}\right)$$ (4)

$${\Delta \text{C}}_{\text{s}}=\sum_{k}\text{L}\left(C_{k}^{T},C_{k}^{0}\right)\;\text{ln}\left(S^{T}/S^{0}\right)$$ (5)

$${\Delta \text{C}}_{\text{r}}=\sum_{k}\text{L}\left(C_{k}^{T},C_{k}^{0}\right)\;\text{ln}\left(R^{T}/R^{0}\right)$$ (6)

$${\Delta \text{C}}_{\text{ec}}=\sum_{k}\text{L}\left(C_{k}^{T},C_{k}^{0}\right)\;\text{ln}\left(EC^{T}/EC^{0}\right)$$ (7)

$${\Delta \text{C}}_{\text{y}}=\sum_{k}\text{L}\left(C_{k}^{T},C_{k}^{0}\right)\;\text{ln}\left(Y^{T}/Y^{0}\right)$$ (8)

$${\Delta \text{C}}_{\text{emf}}=\sum_{k}\text{L}\left(C_{k}^{T},C_{k}^{0}\right)\;\text{ln}\left(EMF_{k}^{T}/EMF_{k}^{0}\right)$$ (9)

Where,

$$\sum_{k}\text{L}\left(C_{k}^{T},C_{k}^{0}\right)=\left(C_{k}^{T}-C_{k}^{0}\right)/\text{ln}\left(C_{k}^{T}/C_{k}^{0}\right)$$ 10

*E*^*T*^ represents *t* year’s energy consumption, $E_{k}^{T}$means total consumption of fossil fuel type *k* in year *t*, *TP*^*T*^ means *t* year’s thermal power generation, *G*^*T*^ means *t* year’s total power generation, *EC*^*T*^ means *t* year’s total electric power consumption, *Y*^*T*^ means *t* year’s GDP, $C_{k}^{T}$ means carbon emissions of fuel *k* in year *t*, $ES_{k}^{T}$ means energy consumption proportion of the total consumption for fuel *k* in year *t* $\left(E_{k}^{T}/E^{T}\right)$, *CR*^*T*^ means *t* year’s energy consumption per unit thermal power generating production(*E*^*T*^/*TP*^*T*^), $S_{k}^{T}$ means *t* year’s proportion of thermal power production to the total power production (*TP*^*T*^/*G*^*T*^), *R*^*T*^ means the ratio of *t* year’s power production and consumption (*G*^*T*^/*EC*^*T*^), $EMF_{k}^{T}$ means the ratio of carbon emissions to energy consumption in *t* year for fuel *k*.

### STIRPAT model

STIRPAT model known as the random regression model that proposed by Dietz, is used in the paper to analyze the influence of population, affluence, and technology on environmental stress. That can be represented as below:

$$\text{A}={\text{aP}}^{{\text{c}}_{1}}{\text{A}}^{{\text{c}}_{2}}{\text{T}}^{{\text{c}}_{3}}\text{e}$$ (11)

In this equation, ’a’ means a constant, while c_1_, c_2_, and c_3_ are fitting coefficients. ’I’ means environmental stress, ’A’ represents wealth, ’P’ represents the population, ’e’ means the error of the model, and ’T’ represents technology.

The STIRPAT model allows for parameter estimation of the coefficients to assess the effect of each factor and enables proper decomposition. Previous studies have made improvements to the relevant variables based on their research purposes and needs.

In this paper, we made adjustments to the variables based on the LMDI model presented in previous chapters. The main five factors affect carbon emissions were identified as economy scale, electricity consumption scale, generation efficiency, electric structure, and energy structure. In this study, the economic scale factor is represented by GDP, electricity consumption is represented by total electricity consumption, generation efficiency is represented by energy consumption per unit of thermal power production per year, electric structure is represented by the proportion of thermal power generation to the total, and energy structure is measured by the proportion of coal and coke consumption to the total consumption. It is important to note that coal has a higher carbon intensity compared to petroleum and natural gas, accounting for 36% and 61% respectively in terms of heat generation. Therefore, the proportion of coal and coke consumption are used as an index for measure the energy structure.

The formula for completing the improvement represented as below:

$$\text{C}=\text{a}\beta_{1}^{c_{1}}\beta_{2}^{c_{2}}\beta_{3}^{c_{3}}\beta_{4}^{c_{4}}\beta_{5}^{c_{5}}e$$ (12)

That a expressed a constant, *c*_1_, *c*_2_, *c*_3_,*c*_4_,*c*_5_ are fitting coefficient, *β*_1_ represent scale of economy, *β*_2_ represent the electricity consumption, *β*_3_ represent generating efficiency, *β*_4_ represent energy structure, *β*_5_ represent electric industry structure, *e* represent the model error. Eq (13) can be obtained as below by transforming the formula (12):

$$\text{ln}C=\text{lna}+c_{1}\text{ln}\beta_{1}+c_{2}\text{ln}\beta_{2}+c_{3}\text{ln}\beta_{3}+c_{4}\text{ln}\beta_{4}+c_{5}\text{ln}\beta_{5}+1$$ (13)

### Logistic model

The logistic model, as well as known as the block growth model, were utilized in various applications such as forecasting microbial growth and population projections, forecast energy consumption and carbon emissions, and exploring disease risk factors for disease control. The logistic model is advantageous due to its simplicity, mathematical feasibility, and lack of stringent assumptions. Therefore, it is well-suited for depicting feedback mechanisms among energy consumption, environmental impacts, and economic growth. The logistic model employed for predicting carbon emissions can be represented as below:

$$\frac{\text{dC}}{\text{dt}}=\gamma \text{C}\left(1-\frac{C}{K}\right),C_{0}={C|}_{t=0}$$ (14)

Eq (14) can be obtained as below by transforming the formula (13):

$$N=\frac{K}{1+\left(\frac{K}{C_{0}}-1\right)e^{-\gamma \text{t}}}$$ (15)

In the given context, the variables are defined as follows: *C* means carbon emissions, *C*_0_ means carbon emissions in the base year, *r* represents growth factor, and *K* represents the carbon emissions ultimate amount.

Set

$$Y=\text{ln}\left(\frac{K-N}{C}\right)$$ (16)

$$\frac{K}{c_{0}}-1=e^{\alpha }$$ (17)

Eq (18) can be obtained as below by transforming the formula (15):

Y=α−γ (18)

To obtain the optimal values of α and γ, an alternating iterative method can be applied, as described in Eq (18) [23]. In this study, we aim to predict carbon emissions.

### GM (1, 1) model

The grey forecast theory has widely been utilized for the analysis, modeling, and prediction of grey systems. Grey prediction is employed when there is a combination of known and uncertain information within a system. When examining the growth trend of power industry carbon emissions, some information is known while other information remains unknown, with uncertain relationships among various factors in the system. Therefore, the grey forecasting model can be used to predict carbon dioxide emissions.

The original sequence data should undergo processing to generate a cumulative time series in order to reduce the randomness of the original time series.

The original time series can be represented as below:

$$C^{\left(0\right)}=\left(C^{\left(0\right)}(1),C^{\left(0\right)}(2),\ldots ,C^{\left(0\right)}(n)\right)$$ (19)

One-accumulate of the time-series is represented as below:

$$C^{\left(1\right)}=\left(c^{\left(1\right)}(1),c^{\left(1\right)}(2),\ldots ,c^{\left(1\right)}(n)\right)$$ (20)

where

$$c^{\left(1\right)}(k)=\sum_{i=1}^{k}c^{\left(0\right)}(i),\text{k}=1,2\ldots ,\text{n}.$$ (21)

Neighbor sequences of the time-series can be represented as below:

$$Y^{\left(1\right)}=\left(y^{\left(0\right)}(1),y^{\left(1\right)}(2),\ldots ,y^{\left(1\right)}(n)\right)$$ (22)

Where

$$y^{\left(1\right)}(k)=0.5c^{\left(1\right)}(k)+0.5c^{\left(1\right)}(k-1)$$ (23)

For the original time series, corresponding to GM(1,1), the differential equation of GM is:

$$\frac{\text{d}x^{\left(1\right)}}{\text{dt}}+\text{a}c^{\left(1\right)}=\text{b}$$ (24)

The forecast model can be obtained to solve the differential equations:

$${\hat{C}}^{\left(1\right)}(k-1)=\left[c^{\left(0\right)}(1)-\frac{b}{a}\right]e^{-ak}+\frac{b}{a}\cdot \text{k}=1,2,\ldots ,\text{n}$$ (25)

By using the least square method, the parameters of a and b can be estimated as follow.


$$\hat{a}={\left(a,b\right)}^{T}$$ (26)

$$\hat{a}={\left(B^{T}B\right)}^{-1}B^{T}Z_{n}$$ (27)

Where

$$\text{B}=\left[\begin{array}{cc} -y^{\left(1\right)}(2) & 1\\ -y^{\left(1\right)}(3) & 1\\ \ldots & \ldots \\ -y^{\left(1\right)}(n) & 1 \end{array}\right],\;z_{n}=\left[\begin{array}{c} c^{\left(0\right)}(2)\\ c^{\left(0\right)}(3)\\ \ldots \\ c^{\left(0\right)}(n) \end{array}\right]$$ (28)

### Data source

The primary data sources for this article include the China Statistical Yearbook [2], China Electric Power Yearbook [24] (S1 File), and China Energy Statistical Yearbook [25] (S2 File). Conversion coefficients were calculated using industrial organization data. Energy consumption is measured in tonnes of standard coal equivalent (tce), unless otherwise specified.

To ensure consistency, the industrial output for the year 2000 remains constant in the LMDI model employed in this study. Due to limited data availability, carbon emissions are calculated only up until the year 2020.

## Results and discussion

### Electricity industry carbon emissions in China power industry

**Fig 1:**
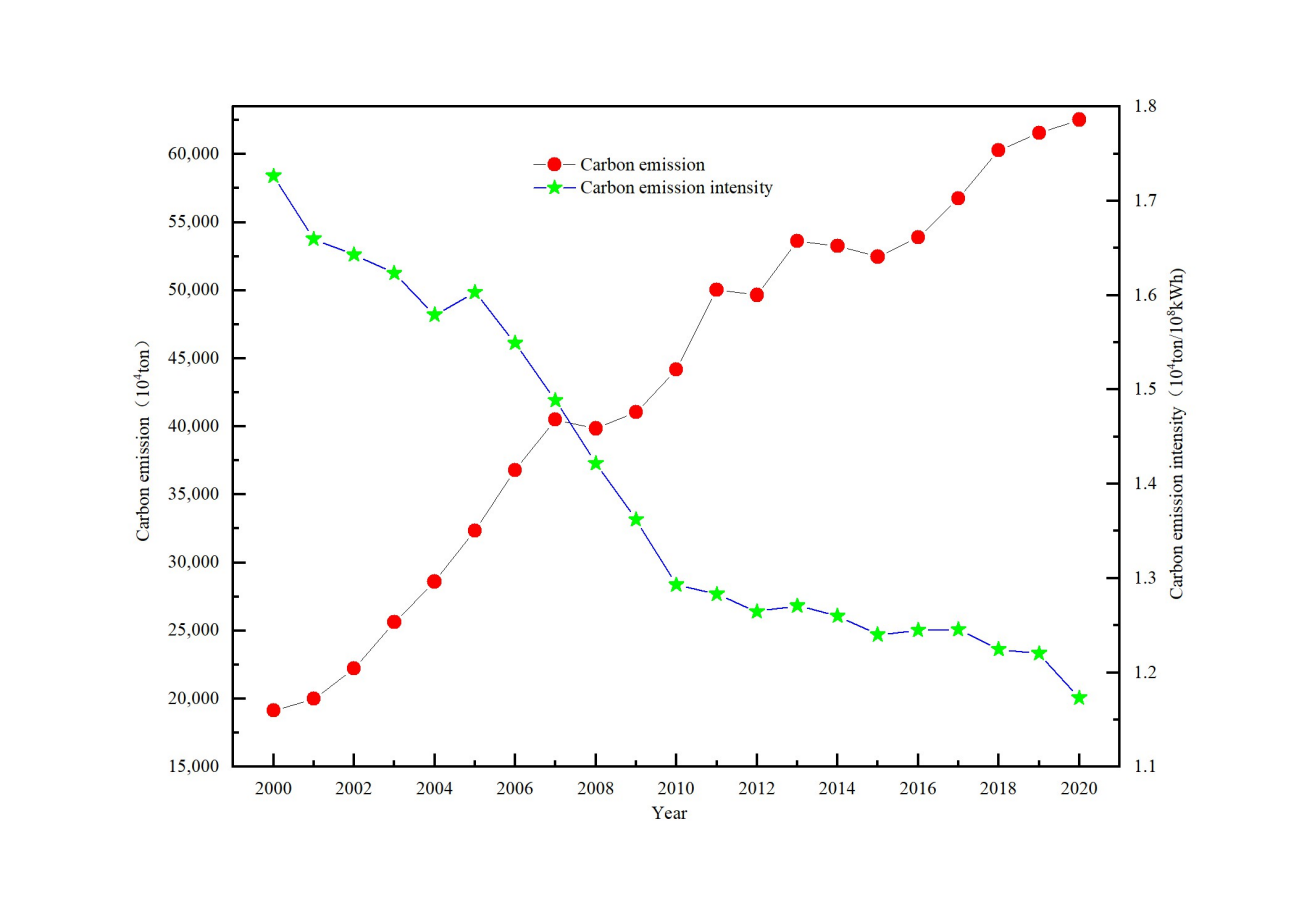
Variation of carbon emission and intensity from 2000 to 2020.

The intensity of carbon emissions refers means amount of carbon emitted per unit of power generation. Fig 1 depicts carbon emissions as well as emission intensity of the electricity industry over the past twenty years.

As shown in Fig 1, carbon emissions have grown from 19126.82×10^4^ tonnes in 2000 to 62528.64×10^4^ tonnes in 2020, with a 226.91% increase in 2020 compared to the 2000 level. The growth rate per year is 10.81%. This growth is mainly due to China’s high-speed economic development over the past two decades, which has led to carbon emissions increased rapidly.

However, Fig 1 also depicts the emission intensity decreased from 1.73 tonnes/10^8^ kWh to 1.17 tonnes/10^8^ kWh over the same period, indicating a decrease of 32.4%. This is mainly due to improvements in coal quality and reductions in energy consumption in thermal power plants. Power generating enterprises should strictly implement national energy efficiency and emission reduction policies, including targets such as generating unit energy consumption, water consumption, and emissions. The assessment of these factors should also be strengthened. Power generating units with high energy consumption and pollution should be restricted from investing in small fossil-fuel generators and encouraged to invest in efficient cleaning units and renewable energy units. Additionally, the implementation of energy conservation policies and technologies has also make a critical part in promoting the descend of carbon emission intensity.

### Decomposition of electric power industry carbon emission in China power industry

**Fig 2:**
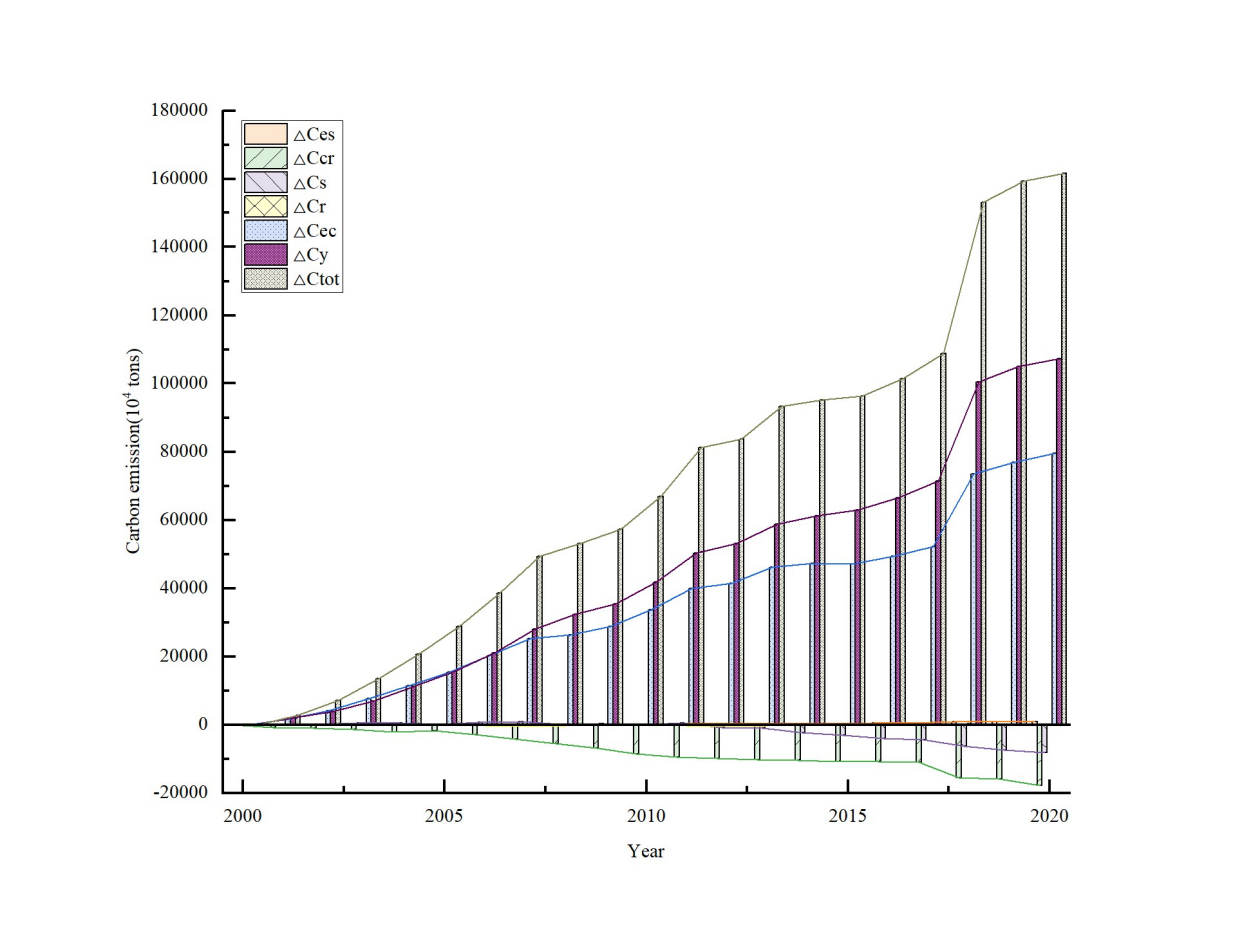
Carbon emission decomposition from 2000 to 2020, China.

To better understand the transformation of carbon emissions in the electricity industry, we utilized the LMDI model to analyze the carbon emissions. The data were input into formulas (1) to (9), and the findings are presented in Fig 2. As the carbon emission factor remains constant in practical application, it is assumed to be equal to zero.

According to the analysis presented in Fig 2, several factors have been found to impact carbon emissions in the period from 2000 to 2020. Economic scale, electricity consumption, energy structure all have a positive effect on emissions, while the effect between power generation efficiency and power consumption is negative. The electricity industry structure factor become negative from positive. As the magnitude of effect increases, the overall effect on carbon emissions increases in the same time.

That also indicate the energy structure, electric power consumption scale, and economic scale contribute to the raise of carbon emissions. While, power generation efficiency, the ratio of consumption to generation, electricity industry structure promotes the carbon emissions reduction.

The continuous increase in economic size is one of the main drivers of carbon emissions growth in China’s electricity industry. From 2001 to 2020, the contribution of economies of scale to carbon emissions showed an upward trend, with a cumulative contribution of 114.91%. The impact of power consumption factors on carbon emissions is another significant driver, although its influence is less pronounced compared to economies of scale. From 2000 to 2020, the cumulative contribution of power consumption factors to carbon emissions was 85.17%.

Although energy structure has a positive impact on carbon emissions changes, its influence is much smaller compared to economies of scale and power consumption. From 2000 to 2020, the cumulative contribution of energy structure factors to carbon emissions changes was only 0.94%, showing a downward trend. This suggests that the impact of energy structure changes on carbon emissions in China’s electricity industry is weakening.

The electricity industry structure effect shifted from positive to negative from 2000 to 2020, indicating that with the adjustments made in the electricity industry, this factor has started to reduce carbon emissions.

Improving power generation efficiency is a crucial factor in reducing the growth of carbon emissions in the electricity industry and plays a decisive role in energy-saving and emission-reduction efforts in China. During the period from 2000 to 2020, changes in power generation efficiency resulted in a cumulative contribution of -19.15% to carbon emissions, showing an overall upward trend. This emphasizes that improving power generation efficiency is a decisive factor in reducing carbon emissions in China’s electricity industry.

The ratio of power dissipation to generation factor has the smallest impact on carbon emissions variation, with a cumulative contribution of only -0.01%. However, it remains a decisive factor in reducing carbon emissions in the electricity industry.

### Prediction model

#### The prediction results of STIRPAT model

Based on the history data and the STIRPAT predict model, the stepwise regression method of SPSS was employed to remove the generation scale factor and economic scale factor due to collinearity issues. The resulting model is presented below:

$$\text{lnC}=0.986\text{ln}\beta_{2}+0.954\text{ln}\beta_{3}+0.986\text{ln}\beta_{4}-4.754$$ (29)

And the comparison with the actual value for the same period is shown in Table 1.

**Table 1:** The predicted carbon emissions and the data error table (STIRPAT model).

Year	Predicted data(10^4^ tonnes)	Actual data(10^4^ tonnes)	Relative errors
**2001**	19988.62	20054.12	0.33%
**2002**	22216.20	22331.42	0.52%
**2003**	25631.04	25626.95	0.02%
**2004**	28591.48	28550.22	0.14%
**2005**	32350.35	32236.67	0.35%
**2006**	36782.36	36752.81	0.08%
**2007**	40499.31	40589.72	0.22%
**2008**	39848.95	39909.51	0.15%
**2009**	41027.76	41041.13	0.03%
**2010**	44171.03	44178.79	0.02%
**2011**	50044.48	50005.59	0.08%
**2012**	49644.98	49722.86	0.16%
**2013**	53640.75	53581.55	0.11%
**2014**	53259.98	53129.10	0.25%
**2015**	52461.88	52324.50	0.26%
**2016**	53874.52	53713.52	0.30%
**2017**	56743.98	56979.11	0.41%
**2018**	60290.79	60518.84	0.38%
**2019**	61558.01	61778.46	0.36%
**2020**	62528.64	62232.34	0.47%

Table 1 illustrates the comparison between actual carbon emissions and the predicted values. The maximum error observed is 0.52%, the minimum error is 0.02%, and the average relative error is 0.23%.

Regarding the scenario assumptions derived from the improved STIRPAT model:

1. Electricity consumption factor: China’s electricity consumption has exhibited slow growth over the years. Based on the average growth rate of the past 10 years (6.39%), this value is considered as the benchmark data.
2. Generating efficiency factor: The improvement rate in China’s power generation efficiency has decelerated. Using the average growth rate of the past 20 years (-1.9%) as the benchmark data, the target value under efficient emission reduction conditions is set at the maximum rate observed (-3.87%). Conversely, the lowest growth rate in nearly 10 years (-0.37%) represents an inefficient emission reduction state.
3. Energy intensity: Recent years have witnessed an accelerated adjustment in China’s power structure. Based on the average growth rate of the past 10 years (-1.71%), this value is considered as the benchmark data. The target value for efficient emission reduction conditions is set at the maximum rate observed (-4.52%), while the lowest growth rate in nearly 10 years (-0.18%) represents an inefficient emission reduction state.

The results of these analyses are presented in Table 4.

#### The prediction results of logistic model

Based on the history data and the logistic predict model, we can derive the forecast formula for carbon emissions below:

$$C=\frac{111350}{1+4.57e^{-0.1061t}}$$ (30)

**Fig 3:**
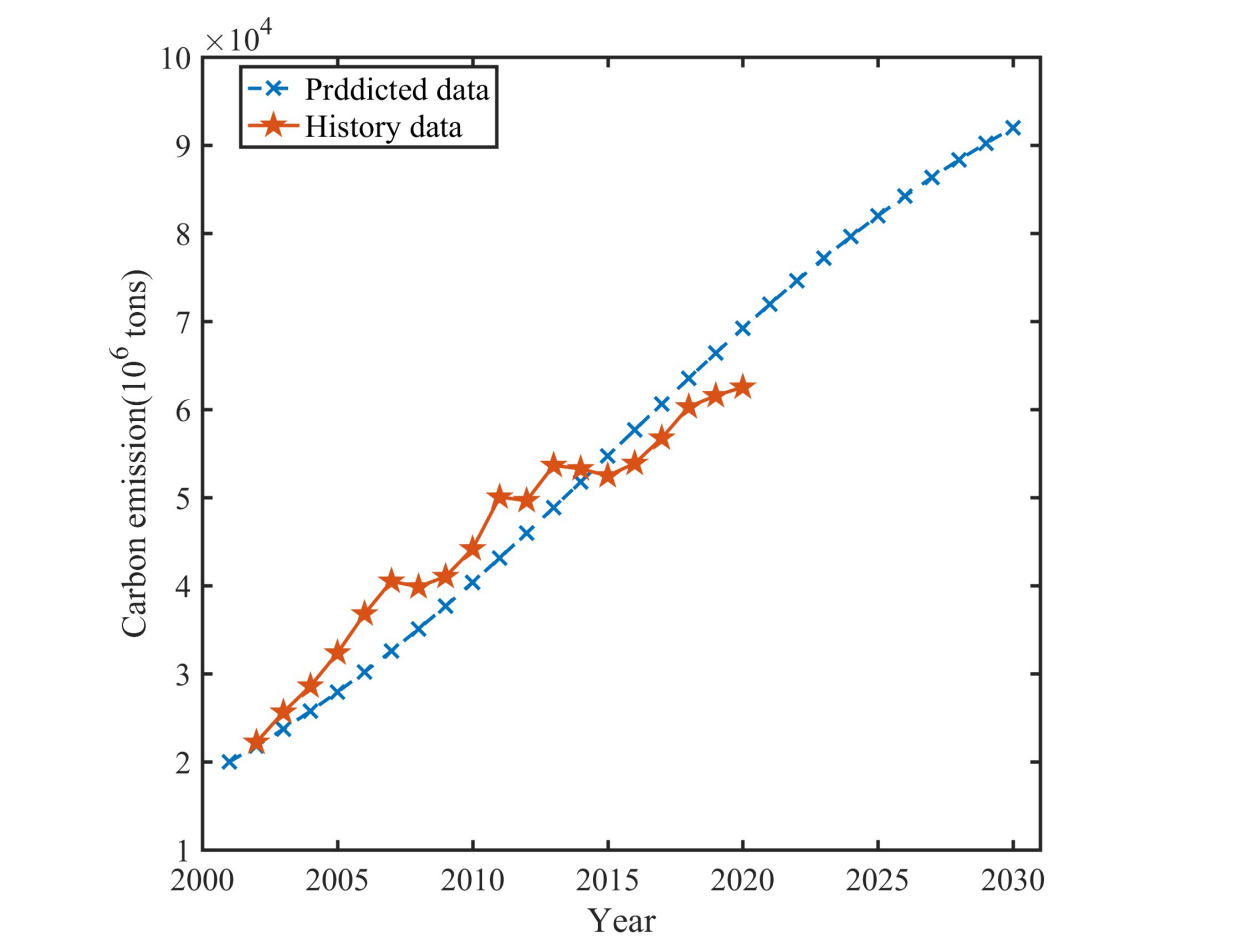
The predicted carbon emissions and the actual from 2000 to 2020, China.

Formula (30) allows us to predict and calculate the total amount of carbon emissions from 2000 to 2020. A comparison with the actual values is presented in Table 2 and Fig 3.

**Table 2:** The predicted carbon emissions and the data error table (Logistic model).

Year	Predicted data(10^4^ tonnes)	Actual data(10^4^ tonnes)	Relative errors
**2001**	19988.62	19988.62	0.00%
**2002**	22216.20	21788.10	1.93%
**2003**	25631.04	23707.54	7.50%
**2004**	28591.48	25747.47	9.95%
**2005**	32350.35	27907.03	13.73%
**2006**	36782.36	30183.85	17.94%
**2007**	40499.31	32573.91	19.57%
**2008**	39848.95	35071.44	11.99%
**2009**	41027.76	37668.90	8.19%
**2010**	44171.03	40356.94	8.63%
**2011**	50044.48	43124.53	13.83%
**2012**	49644.98	45959.05	7.42%
**2013**	53640.75	48846.47	8.94%
**2014**	53259.98	51771.67	2.79%
**2015**	52461.88	54718.69	4.30%
**2016**	53874.52	57671.06	7.05%
**2017**	56743.98	60612.23	6.82%
**2018**	60290.79	63525.88	5.37%
**2019**	61558.01	66396.28	7.86%
**2020**	62528.64	69208.69	10.68%

Table 2 reveals several key findings. The maximum error observed between the actual values and the estimated values is -19.57% in 2007. On the other hand, the minimum error is 0% in 2001. The average relative error is 8.72% from 2001 to 2020. These results indicate that logistic predict model demonstrates a high level of accuracy.

#### The prediction results of GM(1,1) model

Based on the history data and the GM predict model, we can derive the model formula can be representing as follows:

$${\hat{C}}^{\left(1\right)}(k-1)=\left[c^{\left(0\right)}(1)+623620\right]e^{-0.0450k}-623620,\text{k}=1,2,\ldots ,\text{n}$$ (31)

**Fig 4:**
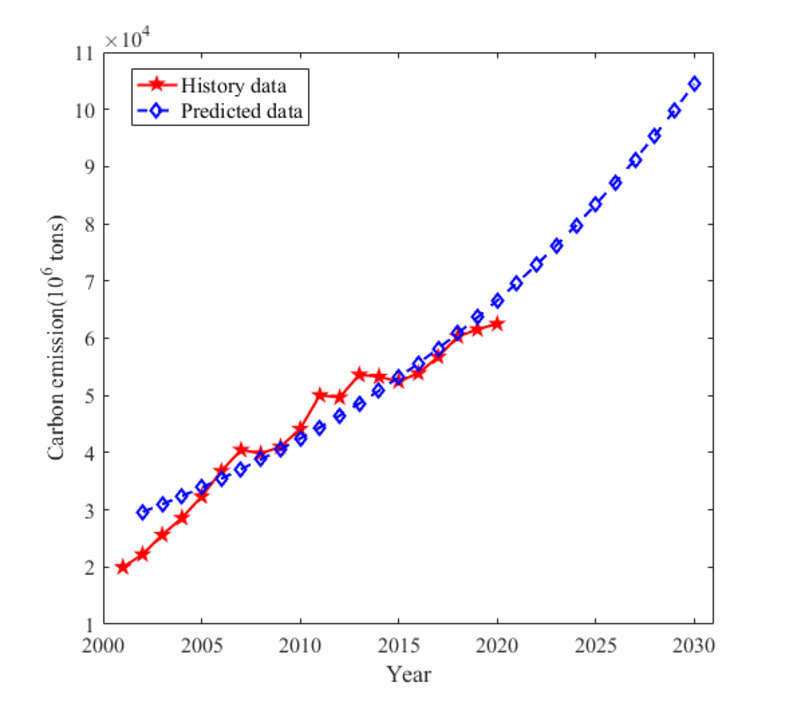
The predicted carbon emissions and the actual from 2000 to 2020, China.

Using formula (31), we can predict and calculate carbon emissions from 2000 to 2020. A comparison with the actual values is presented in Table 3 and Fig 4.

**Table 3:** The predicted carbon emissions and the data error table (GM model).

Year	Predicted data(10^4^ tonnes)	Actual data(10^4^ tonnes)	Relative errors
**2001**	19988.62	19988.62	0.00%
**2002**	22216.20	29625.46	33.35%
**2003**	25631.04	30989.13	20.90%
**2004**	28591.48	32415.58	13.37%
**2005**	32350.35	33907.68	4.81%
**2006**	36782.36	35468.47	3.57%
**2007**	40499.31	37101.10	8.39%
**2008**	39848.95	38808.88	2.61%
**2009**	41027.76	40595.27	1.05%
**2010**	44171.03	42463.89	3.86%
**2011**	50044.48	44418.52	11.24%
**2012**	49644.98	46463.13	6.41%
**2013**	53640.75	48601.85	9.39%
**2014**	53259.98	50839.02	4.55%
**2015**	52461.88	53179.16	1.37%
**2016**	53874.52	55627.02	3.25%
**2017**	56743.98	58187.56	2.54%
**2018**	60290.79	60865.96	0.95%
**2019**	61558.01	63667.65	3.43%
**2020**	62528.64	66598.30	6.51%

Table 3 reveals several key findings. The maximum error observed between the actual values and the predicted values is -33.35% in 2002. Conversely, the minimum error is 0% in 2001, and the average relative error from 2001 to 2020 is 7.05%. These results indicate that the GM (1, 1) model demonstrates higher precision compared to the Logistic model.

#### Comparison of prediction models

**Fig 5:**
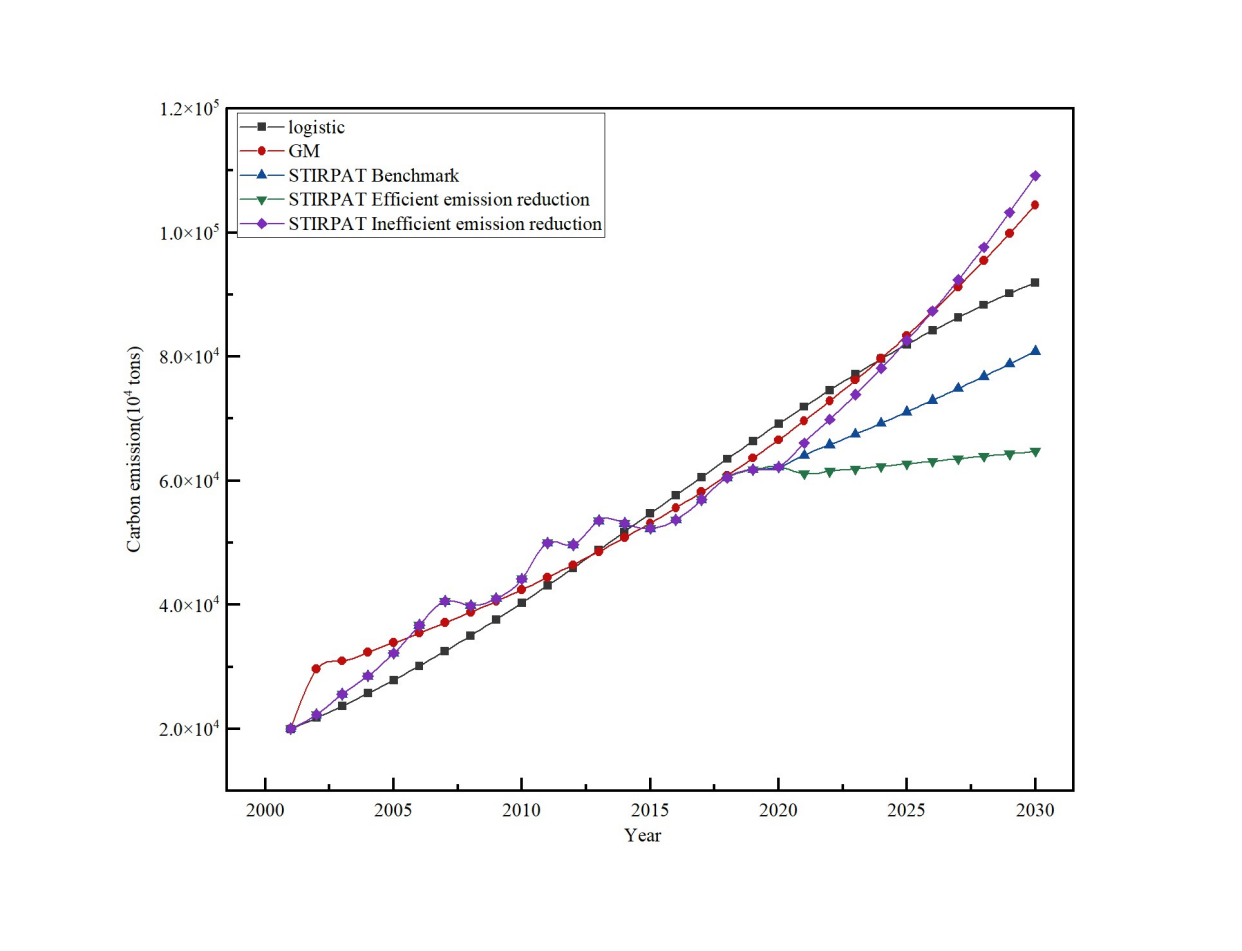
The predicted carbon emissions with different model.

Based on the predictions, it is evident that the results obtained from the three methods are inconsistent. Moreover, as time progresses, the differences between the predictions become more pronounced (Fig 5 and Table 4). Fig 5 reveals that the STIRPAT model with efficient emission reduction status predicts the slowest growth in carbon emissions, while the STIRPAT model in inefficient emission reduction status predicts the steepest growth. The predictions from the logistic method and the STIRPAT model in benchmark status fall in between. Notably, when the STIRPAT model is in inefficient emission reduction status, its prediction closely aligns with that of the GM model.

**Table 4:** The predicted carbon emissions with different model(10^4^ tonnes).

	Logistic	GM	STIRPAT		
			Benchmark	Efficient reduction	Inefficient reduction
2021	71949.54	69663.86	62511.47	62511.47	62511.47
2022	74606.75	72870.52	64143.16	61139.94	66096.22
2023	77169.88	76224.78	65817.44	61533.39	69886.54
2024	79630.21	79733.45	67535.43	61929.37	73894.22
2025	81980.82	83403.62	69298.25	62327.90	78131.71
2026	84216.59	87242.72	71107.09	62729.00	82612.21
2027	86334.06	91258.55	72963.15	63132.67	87349.65
2028	88331.44	95459.22	74867.65	63538.95	92358.75
2029	90208.38	99853.26	76821.86	63947.83	97655.11
2030	91965.83	104449.55	78827.08	64359.35	103255.20

The reason behind these discrepancies is that the STIRPAT model, in its inefficient emission reduction status, solely relies on historical data to predict future emissions, assuming all factors influencing carbon emissions remain constant. This approach fails to consider the quantitative measures implemented by the country and government to reduce carbon emissions. Consequently, predictions based solely on historical data may result in overestimations of future carbon emissions.

Similarly, the GM model also relies solely on historical data to predict future emissions, assuming constant factors. While this assumption holds some validity in the short to medium term, significant changes in factors can occur in the long term. Therefore, GM model could be more suitable in short- to medium-term predictions, especially when the future is uncertain. The GM model’s prediction appears as the second steepest due to the absence of considerations for the country’s energy policy and quantitative measures for carbon emission reduction.

Conversely, the prediction curve of the STIRPAT model with efficient emission reduction status lies at the bottom. This is primarily because the model incorporates a broader range of influential factors, providing a better representation of the country’s policy as well as the environmental effect feedback mechanism. Consequently, the prediction curve appears flatter, making this approach more suitable for medium- to long-term predictions.

Fig 5 demonstrates that using the logistic model, carbon emissions in China’s electric industry are projected to flatten out and gradually peak around 2030. Meanwhile, employing the STIRPAT model in benchmark status, carbon emissions in China’s electric industry are expected to plateau by 2030, following a peak during the 2021–2030 period. These results provide valuable insights for guiding the path of China’s electric industry in achieving the dual carbon goal.

## Results discussion

From 2000 to 2020, carbon emissions experienced a significant increase, rising from 19,126.82×10^4^ tonnes in 2000 to 62,528.64×10^4^ tonnes in 2020. This represents a growth of 226.91% compared to the 2000 level, with an average annual growth rate of 10.81%. However, carbon emission intensity decreased at the same period, declining from 1.73 tonnes/10^8^ kWh to 1.17 tonnes/10^8^ kWh, a reduction of 32.4%. This reduction can be attributed to the implementation of energy-saving measures, emission reduction policies, and technologies.

The LMDI analysis reveals that among all the factors analyzed in this research, the economy scale has the most notable influence on carbon emissions in the electricity industry. Both the scale of electric power consumption as well as energy structure have notable effects on carbon emissions. Conversely, power generation efficiency as well as the ratio of consumption to generation have a negative impact, while the electricity sector structure changes from positive to negative. Chen et al. [26] indicated that the economic activity effect was the most significant driver on CO_2_ emissions in all provinces in China, which is consistent with the research in this article.

The STIRPAT model predicts the slowest carbon emission growth in the efficient emission reduction scenario, the steepest growth in the inefficient emission reduction scenario, and an intermediate result when using the logistic method and the STIRPAT model in the benchmark status. Notably, the prediction from the STIRPAT model in the inefficient emission reduction scenario closely aligns with the prediction of the GM model.

## Conclusions and policy implications

### Conclusions

The paper studied the electricity industry carbon emissions and in China power industry and emission decomposition of electric power industry carbon emission, and draws the following conclusions:

1. The factors that affect carbon emissions between 2000 and 2020 are economic scale, electricity consumption, energy structure, power generation efficiency and ratio of power dissipation to generation.
2. The cumulative contribution of economies scale, power consumption factors and energy structure are 114.91%, 85.17% and 0.94%, which contribute to the increase of carbon emissions, the cumulative contribution of power generation efficiency and ratio of power dissipation to generation factor are -19.15% and -0.01%, which promotes the carbon reduction.
3. STIRPAT model, Logistic model and GM(1,1) model are used to predict carbon emissions, the average relative error between actual carbon emissions and the predicted values are 0.23%, 8.72% and 7.05%, which indicates that STIRPAT model is more suitable for medium- to long-term predictions.

### Policy implications

Without changes in the developmental pattern of the electricity industry in China, a continuous increase carbon emission can be expected. It is crucial to transition from a high-consumption and high-investment raise mode to a focus on low-carbon and environmental protection.

Government departments at all levels should collaborate, take responsibility, and strengthen supervision to effectively implement efficiency policies. Leveraging the carbon emissions exchange mechanism can help achieve green, low-carbon development at a low cost. Additionally, the government should enhance energy-saving knowledge and promote the concept of low-carbon power through educational institutions, communities, and industry associations. Raising awareness of energy saving and environmental protection within society and continually reducing electric consumption intensity in the industry are crucial.

Efforts should be directed towards the development of large-scale new energy power sector, such as wind and solar power, as well as clean energy sources like nuclear power. It is essential to build a new power system centered around new energy. This includes planning and constructing a new energy supply and consumption system. The concentration and distribution of new energy, both onshore and offshore, should be promoted, along with on-site utilization and long-distance transmission, to establish a new pattern of diversified development and utilization of new energy.

The development of transformative power generation technologies should be prioritized. The application of key technologies and major equipment should be vigorously promoted to enhance the efficiency and quality of new energy power generation. Simultaneously, efforts should focus on improving the efficiency of coal-fired power generation and reducing unit carbon emissions through comprehensive upgrading and transformation technology. This includes the promotion of key technologies for flexible transformation of coal-fired power units that adapt to low load and frequent variable load operation.

Supporting policies and institutional mechanisms are crucial for building a new type of power system. Establishing an electricity market system that aligns with the new power system and fostering a unified, open, competitive, safe, efficient, and well-governed national electricity market system is essential. It is also necessary to create an independent and innovative technology research and development system that effectively supports the research and application of key technologies required for the construction of the new power system. Strengthening multidirectional integration of scientific and technological research and development, promoting cross-domain and cross-industry collaborative innovation, and fostering "cross-border integration" between the new power system and other fields are important. Building a green, low-carbon, and competitive power industry system, constructing new power demonstration zones in areas that embody the characteristics of the new power system and fast energy clean transformation, and focusing on breakthroughs and comprehensive promotion are key steps in promoting the comprehensive construction of the new power system. Furthermore, improving the advanced and efficient governance system of the power industry, enhancing the guiding role of power planning, leveraging market mechanisms, strengthening industry supervision, clarifying responsibilities of all parties, and exploring the establishment of a credit-based regulatory mechanism are vital.

## Supporting information

S1 File (PDF)

S2 File (PDF)

S3 File (ZIP)

S4 File (ZIP)

## References

[1] Xiao-ling Ge, Yang Wang, Hong-qing Zhu, Zequn Ding. Analysis and forecast of the Tianjin industrial carbon dioxide emissions resulted from energy consumption. International Journal of Sustainable Energy 36 (7), 637–653.

[2] National Bureau of Statistics (NBS). International Statistical Yearbook 2013.China Statistics: Press 2013; Beijing.

[3] Sheng Zhou, Qing Tong, Sha Yu, Yu Wang, Qi-min Chai, Xi-liang Zhang. Role of non-fossil energy in meeting China’s energy and climate target for 2020. Energy Policy 51, 14–17.

[4] Lin Zeng, Ming Xu, Sai Liang, Si-yu Zeng, Tian-zhu Zhang. Revisiting drivers of energy intensity in China during 1997–2007: A structural decomposition analysis. Energy Policy 67, 640–647.

[5] Hongqing Zhu, Xiaoling Ge, Yang Wang, Zequn Ding. Analysis and forecast of Tianjin’s industrial energy consumption. International Journal of Energy Sector Management,11(1),46–64.

[6] National Development and Reform Commission (NDRC). China’s National Climate Change Programme. Available at: (http://www.gov.cn/zwgk/2007-06/08/content_641704.htm)

[7] National Development and Reform Commission (NDRC). The comprehensive work plan for energy saving and emission reduction in 13th Five-Year. Available from: (http://www.gov.cn/zhengce/content/2017-01/05/content_5156789.htm)

[8] National Development and Reform Commission (NDRC). China’s own national initiative to strengthen climate change action—China’s own national contribution. Available from: (http://www.scio.gov.cn/xwfbh/xwbfbh/wqfbh/2015/20151119/xgbd33811/Document/1455864/1455864.htm)

[9] Sun H., Chen T., Wang C. N.. Spatial impact of digital finance on carbon productivity, Geoscience Frontiers, 2023. doi: 10.1016/j.gsf.2023.101674

[10] Liu Y., Sun H., Meng B., Jin S., & Chen B. How to purchase carbon emission right optimally for energy-consuming enterprises? Analysis based on optimal stopping model, Energy Economics, 2023, 124. doi: 10.1016/j.eneco.2023.106758

[11] Junsong Jia, Lele Xin, Chengfang Lu, Bo Wu, Yexi Zhong. China’s CO2 emissions: A systematical decomposition concurrently from multi-sectors and multi-stages since 1980 by an extended logarithmic mean divisia index. Energy Strategy Reviews, 2023, (49):101141. doi: 10.1016/j.esr.2023.101141

[12] Xue Wang, Lu Li, Fusen Zhao. Decomposition Analysis of CO2 Emissions in Northeast China: Insights From Investment Factors. FRONTIERS IN ENERGY RESEARCH. 2021(9), 1–13.

[13] Wu X, Xu C, Ma T, et al. Carbon emission of China’s power industry: driving factors and emission reduction path. Environmental Science and Pollution Research, 2022, 29(52):78345–78360. doi: 10.1007/s11356-022-21297-5 35690704 PMC9188421

[14] Liu Y, Jiang Y, Liu H, et al. Driving factors of carbon emissions in China’s municipalities: a LMDI approach. Environmental Science and Pollution Research, 2022, 29(15):21789–21802 doi: 10.1007/s11356-021-17277-w 34767167 PMC8586619

[15] Maohui Liu, Xiaowen Deng, Shengnan Liu. Carbon emission analysis of Tianjin City based on LMDI method and Tapio decoupling model. Environmental Pollution & Control 2022, 44(10):1397–1401.

[16] Jia Dong, Cunbin Li. Scenario prediction and decoupling analysis of carbon emission in Jiangsu Province, China. Technological Forecasting and Social Change 2022; 185:1–11.

[17] Ge X L, Wang Y, Zhu H Q, Z Ding. Analysis and forecast of the Tianjin industrial carbon dioxide emissions resulted from energy consumption[J].International Journal of Sustainable Energy,2017,36,637–653.

[18] Nan Zheng, Wanqing Chen, Simin Chen, Jinchun Chen, Han Chen, Xiaofan Lin. Research on Carbon Emission Prediction Method of Power Systems Considering Unit Coal Consumption. 6th IEEE Conference on Energy Internet and Energy System Integration; 2022,2666–2670.

[19] Zhu H, Ge X, Wang Y, Z Ding. Analysis and forecast of Tianjin’s industrial energy consumption. International Journal of Energy Sector Management, 2017, 11(1):46–64.

[20] Yonggui He, Jianghao Yu. Study on the Change Trend of Carbon Emissions and Its Influencing Factors in Hebei Province. Environmental Science and Technology 2018; 41(1):184–191

[21] Feng R, Lihong G. Study on Transition of Primary Energy Structure and Carbon Emission Reduction Targets in China Based on Markov Chain Model and GM (1, 1).Mathematical Problems in Engineering,2016,(2016-12-7), 2016, 2016:1–8

[22] Junsong Jia, Hongbing Deng, Jing Duan, Jingzhu Zhao. Analysis of the major drivers of the ecological footprint using the STIRPAT model and the PLS method—A case study in Henan Province, China. Ecological Economics, 2009, 68(11):2818–2824 doi: 10.1016/j.ecolecon.2009.05.012

[23] Hua Chen, Shao-gui Deng, Yi-ren Fan. Application of homotopy alternative iteration method in double exponential fitting. Computer Engineering and Applications 2007; 43(25):204–205.

[24] National Bureau of Statistics (NBS). China Electric Power Yearbook 2001-2021.China Statistics Press, Beijing.

[25] National Bureau of Statistics (NBS). China Energy Statistic Yearbook 2001-2021.China Statistics Press, Beijing.

[26] Jianfeng Chen, Junsong Jia, Lin Wang, Chenglin Zhong, Bo Wu. Carbon Reduction Countermeasure from a System Perspective for the Electricity Sector of Yangtze River Delta (China) by an Extended Logarithmic Mean Divisia Index (LMDI). Systems, 2023, 11(3):117. doi: 10.3390/systems11030117

