# Supplementary material for: Prediction analysis of carbon emission in China’s electricity industry based on the dual carbon background
Source: PLoS One. 2024 May 17;19(5):e0302068. doi: 10.1371/journal.pone.0302068 (PMC11101092; doi:10.1371/journal.pone.0302068)
Supplement: S1 File — (PDF) [file pone.0302068.s001.pdf]

全 国 部 分

国民经济主要指标

	单　位	2000 年	1999 年	比 1999 年增长 (%)
全国总人口	万人	126583	125909	0.54
城镇人口	万人	45844	38892	17.88
乡村人口	万人	80739	87017	-7.21
年末从业人员	万人	71150	70586	0.80
国内生产总值	亿元	89404	82067	8.00
第一产业	亿元	14212	14472	2.40
第二产业	亿元	45488	40558	9.60
第三产业	亿元	29704	27038	7.80
工业增加值	亿元	39570	35087	9.90
全社会固定资产投资总额	亿元	32619	29855	9.30
国有经济投资总额	亿元	23284	21320	9.20
基本建设投资	亿元	13215	12455	6.10
更新改造投资	亿元	5077	4485	13.20
房地产	亿元	4902	4103	19.50
一次能源生产总量(标准煤)	万 t	109000	109126	-0.12
原　煤	万 t	99800	104500	-4.50
原　油	万 t	16300	16000	1.88
天然气	亿 m^3	277	252	9.92

注　国内生产总值按现价计算，增长率按可比价计算；工业总产值按当年价格计算。

2000 年电力生产基本情况

	单　位	2000 年	1999 年	比 1999 年增长 (%)
**一、发电装机容量**	万 kW	**31932.09**	**29876.79**	**6.88**
水　电	万 kW	7935.22	7297.08	8.75
火　电	万 kW	23754.02	22343.40	6.31
核　电	万 kW	210.00	210.00	—

?1994-2018 China Academic Journal Electronic Publishing House. All rights reserved.　http://www.cnki.net

续表

	单　位	2000 年	1999 年	比 1999 年增长 (%)
**二、单机 6000kW 及以上机组**	**台**	**5235**	**5107**	**128**
	万 kW	28288.29	26585.13	6.41
水　电	台	1062	984	78
	万 kW	5747.13	5202.35	10.47
火　电	台	4170	4120	50
	万 kW	22331.15	21172.77	5.47
其中：供热	台	1493	1313	180
	万 kW	2986.41	2815.91	6.05
其中：国外机组	台	1051	1061	-10
	万 kW	7773.75	7064.81	10.03
水　电	台	125	115	10
	万 kW	1444.28	1274.78	13.30
火　电	台	924	944	-20
	万 kW	6149.47	5610.04	9.62
平均单机容量	万 kW/台	5.4	5.21	0.19
**三、35kV 及以上输电线路长度**	**km**	**726167**	**686084**	**5.84**
其中：500kV	km	26837	22927	17.05
330kV	km	8669	7949	9.06
220kV	km	128114	121790	5.19
110kV	km	201230	190961	5.38
**四、35kV 及以上变电设备容量**	**万 kV·A**	**99612**	**91775**	**8.54**
其中：500kV	万 kV·A	9447	8012	17.91
330kV	万 kV·A	1410	1248	12.98
220kV	万 kV·A	30632	28027	9.29
110kV	万 kV·A	35384	32406	9.19
**五、发电量**	**亿 kW·h**	**13684.82**	**12331.41**	**10.98**
水　电	亿 kW·h	2431.34	2129.27	14.19
火　电	亿 kW·h	11079.36	10047.37	10.27
核　电	亿 kW·h	167.37	148.33	12.84
**六、6000kW 及以上电厂供热量**	**万百万 kJ**	**120434.27**	**108907**	**10.58**
**七、6000kW 及以上电厂供电煤耗**	**g/（kW·h）**	**392**	**399**	**-7**
**八、6000kW 及以上电厂发电煤耗**	**g/（kW·h）**	**363**	**369**	**-6**
**九、6000kW 及以上电厂厂用电率**	**%**	**6.28**	**6.5**	**-0.22**
水　电	%	0.49	0.55	-0.06
火　电	%	7.31	7.51	-0.2
**十、6000kW 及以上电厂利用小时**	**h**	**4517**	**4393**	**124**
水　电	h	3258	3198	60
火　电	h	4848	4719	129
**十一、供电量**	**亿 kW·h**	**11365.77**	**10336.01**	**9.96**
售电量	亿 kW·h	10490.4	9498.47	10.44
线损电量	亿 kW·h	875.38	837.54	4.52
线路损失率	%	7.7	8.10	-0.4

?1994-2018 China Academic Journal Electronic Publishing House. All rights reserved. http://www.cnki.net

续表

	单 位	2000 年	1999 年	比 1999 年增长（%）
**十二、6000kW 及以上电厂燃料消耗**				
发电消耗标准煤量	万 t	39789.37	36539	8.89
发电消耗原煤量	万 t	52810.45	48187	9.60
发电消耗燃油量	万 t	1041.56	1159	-10.14
发电消耗燃气量	万 m^3	1538163	1290668	19.18
供热消耗标准煤量	万 t	4780.64	4489	6.50
供热消耗原煤量	万 t	6382.3	5689	12.18
供热消耗燃油量	万 t	175.6	201	-12.77
供热消耗燃气量	万 m^3	873543	965192	-9.50
**十三、6000kW 及以上电厂热效率**				
电厂发电热效率	%	33.84	33.46	0.38
电厂供热效率	%	85.96	82.78	3.18
能源转换总效率	%	39.43	38.85	0.58
**十四、发用电设备比**				
发电设备容量：用电设备容量		1:2.28	1:2.16	
**十五、电力弹性系数**				
电力生产弹性系数		1.37	0.92	0.45
电力消费弹性系数		1.42	0.92	0.5
**十六、电力消费能源占一次能源的比重**	**%**	**41.72**	**40.07**	**1.65**

2000 年电力工业建设项目投资完成情况

	计算单位	2000 年	1999 年	比 1999 年增长（%）
**一、固定资产投资完成额**	**亿元**	**2125.53**	**1837.06**	**15.70**
基本建设投资	亿元	953.66	1153.70	-17.34
“大代小”投资	亿元	43.39	33.77	28.49
城乡电网投资	亿元	1128.48	649.59	73.72
**（一）基本建设投资完成额**	**亿元**	**953.67**	**1153.70**	**-17.34**
**1. 按资金来源分**				
非经营基金	亿元	1.06	1.45	-26.90
开行软贷	亿元		12.85	
开行贷款	亿元	216.36	300.93	-28.10
商行贷款	亿元	170.81	198.36	-13.89
利用外资	亿元	210.81	220.55	-4.42
中央专项	亿元	25.66	25.55	0.43
三峡基金	亿元	14.77	9.20	60.54
煤代油	亿元		0.60	

?1994-2018 China Academic Journal Electronic Publishing House. All rights reserved. http://www.cnki.net

续表

	计算单位	2000年	1999年	比1999年增长(%)
企业自有	亿元	138.34	154.64	-10.54
中央债券	亿元	0.19		
地方债券	亿元		4.30	
地方专项	亿元	62.11	67.96	-8.61
地方其他	亿元	33.01	54.73	-39.69
其　他	亿元	80.55	102.58	-21.48
2.按类型分				
电　源	亿元	642.38	787.47	-18.42
电　网	亿元	260.08	300.98	-13.59
其　他	亿元	51.21	65.25	-21.52
3.按隶属关系分				
国家电力公司全资	亿元	327.76	375.85	-12.79
国家电力公司控股	亿元	308.50	378.69	-18.53
国家电力公司参股	亿元	158.69	222.79	-28.77
地方电力企业	亿元	112.33	97.71	14.96
其他电力企业	亿元	46.38	78.66	-41.04
4.按构成分				
建筑工程	亿元	246.75	314.00	-21.42
安装工程	亿元	134.67	148.22	-9.14
设备工器具购置	亿元	316.10	393.81	-19.73
其他工程	亿元	256.14	297.66	-13.95
**(二)"大代小"投资完成额**	**亿元**	**43.40**	**33.77**	**28.52**
1.按资金来源分				
开行贷款	亿元		2.29	
商行贷款	亿元	19.43	9.37	107.36
利用外资	亿元	4.19	5.75	-27.13
企业自有	亿元	7.27	11.22	-35.20
中央专项	亿元	0.20		
地方专项	亿元	0.81	0.45	80.00
地方其他	亿元	2.27	1.06	114.15
其　他	亿元	9.23	3.63	154.27
2.按构成分				
建筑工程	亿元	12.35	8.00	54.38
安装工程	亿元	5.65	6.69	-15.55
设备工器具购置	亿元	15.62	10.66	46.53
其他工程	亿元	8.78	8.42	4.28
**(三)城乡电网建设与改造投资完成额**	**亿元**	**1128.48**	**649.59**	**73.72**
1.按电网构成分				
城网部分	亿元	354.21	254.04	39.43
农网部分	亿元	774.27	395.55	95.75

?1994-2018 China Academic Journal Electronic Publishing House. All rights reserved. http://www.cnki.net

续表

	计算单位	2000年	1999年	比1999年增长（%）
其中：农网直供部分	亿元	376.57	226.93	65.94
农网趸售部分	亿元	397.60	168.62	135.86
2.按电压等级分				
220kV	亿元	21.76	20.11	8.20
110kV	亿元	174.72	125.42	39.31
35kV	亿元	113.00	51.37	119.97
10kV及以下	亿元	749.45	412.19	81.82
其　他	亿元	69.56	40.50	71.75
(1) 城网部分				
220kV	亿元	20.34	18.55	9.65
110kV	亿元	91.07	74.43	22.36
35kV	亿元	23.87	15.40	55.00
10kV及以下	亿元	167.07	110.56	51.11
其　他	亿元	51.86	35.10	47.75
(2) 农网部分				
220kV	亿元	1.42	1.56	-8.97
110kV	亿元	83.64	50.99	64.03
35kV	亿元	89.12	35.98	147.69
10kV及以下	亿元	582.38	301.63	93.08
其　他	亿元	17.70	5.40	227.78
**二、年新增固定资产**	**亿元**	**865.42**	**1034.98**	**-16.38**
基本建设新增	亿元	838.22	969.10	-13.51
“大代小”新增	亿元	27.20	65.88	-58.71
**三、基建新增生产能力**				
**1.新增单机500kW及以上机组**	**万kW**	**2012.04**	**2052.30**	**-1.96**
水　电	万kW	452.41	628.66	-28.04
火　电	万kW	1559.63	1423.64	9.55
其中：新增大中型机组	万kW	1934.03	1890.88	2.28
水　电	万kW	430.30	587.65	-26.78
火　电	万kW	1503.73	1303.23	15.38
**2.新增110kV及以上线路**	**km**	**15778.27**	**13470.74**	**17.13**
500kV	km	6205.21	3057.62	102.94
330kV	km	706.00	757.35	-6.78
220kV	km	6585.26	6912.05	-4.73
110kV	km	2281.80	2743.72	-16.84
**3.新增110kV及以上变电设备**	**万kV·A**	**4284.55**	**3935.40**	**8.87**
500kV	万kV·A	1974.40	1135.20	73.93
330kV	万kV·A	147.00	207.00	-28.99
220kV	万kV·A	2037.00	2278.20	-10.59
110kV	万kV·A	126.15	315.00	-59.95

?1994-2018 China Academic Journal Electronic Publishing House. All rights reserved. http://www.cnki.net

续表

	计算单位	2000 年	1999 年	比 1999 年增长 (%)
**四、"大代小"投产能力**				
**1. 新增单机 500kW 及以上机组**	**万 kW**	**40.90**	**132.20**	**-69.06**
火　电	万 kW	40.90	132.20	-69.06
**2. 新增 110kV 及以上线路**	**km**	**0.80**		
220kV	km	0.80		
**3. 新增 110kV 及以上变电设备**	**万 kV·A**	**18.00**		
220kV	万 kV·A	18.00		
**五、城乡电网建设与改造投产能力**				
**1. 投产线路**	**km**	**1923711.57**	**758581.81**	**153.59**
220kV	km	1193.86	1028.34	16.10
110kV	km	11169.00	7580.65	47.34
35kV	km	24977.07	15464.76	61.51
1～10kV	km	458643.41	287569.90	59.49
低压线路	km	1427728.23	446938.16	219.45
**2. 投产变电设备**	**万 kV·A**	**8791.34**	**6149.73**	**42.95**
220kV	万 kV·A	336.00	551.30	-39.05
110kV	万 kV·A	3420.71	2257.77	51.51
35kV	万 kV·A	1852.43	877.15	111.19
1～10kV	万 kV·A	3182.20	2463.51	29.17
**六、施工、竣工房屋建筑面积**				
**1. 施工房屋面积**	**万 m^2**	**902.64**	**997.80**	**-9.54**
其中：住宅	万 m^2	393.94	591.06	-33.35
**2. 竣工面积**	**万 m^2**	**373.31**	**595.68**	**-37.33**
其中：住宅	万 m^2	208.92	393.79	-46.95
**七、基本建设规模**				
**1. 上年结转规模**	**万 kW**	**6273.04**	**7105.20**	**-11.71**
水　电	万 kW	2600.19	3155.84	-17.61
火　电	万 kW	3672.85	3949.36	-7.00
**2. 当年新开工规模**	**万 kW**	**593.68**	**582.65**	**1.89**
水　电	万 kW	95.70	46.95	103.83
火　电	万 kW	497.98	535.70	-7.04
**3. 当年在建规模**	**万 kW**	**7462.65**	**8163.92**	**-8.59**
水　电	万 kW	3167.92	3187.84	-0.62
火　电	万 kW	4294.73	4976.08	-13.69
**4. 当年投产规模**	**万 kW**	**1934.03**	**1890.88**	**2.28**
水　电	万 kW	430.30	587.65	-26.78
火　电	万 kW	1503.73	1303.23	15.38
**5. 年末建设规模**	**万 kW**	**5528.62**	**6273.04**	**-11.87**
水　电	万 kW	2737.62	2600.19	5.29
火　电	万 kW	2791.00	3672.85	-24.01
**6. 当年投产容量与建设规模比**		**1:3.9**	**1:4.3**	
水　电		1:7.4	1:5.4	
火　电		1:2.9	1:3.8	

注　在建规模中含三峡、万家寨、小浪底的规模。

?1994-2018 China Academic Journal Electronic Publishing House. All rights reserved.　http://www.cnki.net

国民经济主要指标

	单 位	2002年	比2001年增长(%)
全国总人口	万人	128453	0.65
城镇人口	万人	50212	4.47
乡村人口	万人	78241	-1.66
年末从业人员	万人	73740	0.98
国内生产总值	亿元	102398	8.00
第一产业	亿元	14883	2.90
第二产业	亿元	52982	9.90
第三产业	亿元	34533	7.30
工业增加值	亿元	45935	10.20
全社会固定资产投资总额	亿元	43202	16.10
国有经济投资总额	亿元	31020	17.00
基本建设投资	亿元	17251	16.40
更新改造投资	亿元	6584	11.10
房地产	亿元	7736	21.90
一次能源生产总量（标准煤）	亿t	13.87	18.50
原 煤	万t	13.80	18.90
原 油	万t	1.67	1.80

注 国内生产总值、工业增加值绝对数按现价计算，增长速度按可比价格计算。

2002年电力生产基本情况

	单 位	2002年	2001年	比2001年增长(%)
**一、发电装机容量**	**万kW**	**35657.09**	**33848.69**	**5.34**
水 电	万kW	8607.46	8300.64	3.70
火 电	万kW	26554.67	25301.2	4.95
核 电	万kW	446.80	210.00	112.76
**二、单机6000kW及以上机组**	**台**	**6100**	**5860**	**240**
	万kW	32570.57	30924.65	5.32
水 电	台	1424	1371	53
	万kW	6458.22	6307.14	2.40

?1994-2018 China Academic Journal Electronic Publishing House. All rights reserved. http://www.cnki.net

续表

	单 位	2002 年	2001 年	比 2001 年增长（%）
火 电	台	4670	4486	184
	万 kW	25665.55	24407.51	5.15
其中：供 热	台	1937	1827	110
	万 kW	3743.67	3478.07	7.64
其中：国外机组	台	1125	1094	31
	万 kW	9318.07	8569.66	8.73
水 电	台	151	144	7
	万 kW	1624.92	1594.38	1.92
火 电	台	974	950	24
	万 kW	7693.15	6975.28	10.29
平均单机容量	万 kW/台	5.34	5.28	0.06
**三、35kV 及以上输电线路长度**	**km**	**803505**	**781854**	**2.77**
其中：500kV	km	36745	31486	16.70
330kV	km	9612	9177	4.74
220kV	km	142362	135935	4.73
110kV	km	226567	220051	2.96
**四、35kV 及以上变电设备容量**	**万 kVA**	**124481**	**111771**	**11.37**
其中：500kV	万 kVA	13750	11731	17.21
330kV	万 kVA	1755	1527	14.93
220kV	万 kVA	37209	34026	9.35
110kV	万 kVA	44762	40238	11.24
**五、发电量**	**亿 kWh**	**16541.64**	**14838.56**	**11.48**
水 电	亿 kWh	2745.65	2611.08	5.15
火 电	亿 kWh	13522.04	12044.78	12.26
核 电	亿 kWh	264.89	174.72	51.61
**六、6000kW 及以上电厂供热量**	**万百万 kJ**	**139150.48**	**128743.69**	**8.08**
**七、6000kW 及以上电厂供电煤耗**	**g/(kWh)**	**383**	**385**	**-2**
**八、6000kW 及以上电厂发电煤耗**	**g/(kWh)**	**356**	**357**	**-1**
**九、6000kW 及以上电厂厂用电率**	**%**	**6.15**	**6.24**	**-0.09**
水 电	%	0.49	0.46	0.03
火 电	%	7.10	7.25	-0.15
**十、6000kW 及以上电厂利用小时**	**h**	**4860**	**4588**	**272**
水 电	h	3289	3129	160
火 电	h	5272	4900	372
**十一、供电量**	**亿 kWh**	**14032.27**	**12556.45**	**11.75**
售电量	亿 kWh	12976.89	11608.11	11.79
线损电量	亿 kWh	1055.38	948.34	11.29
线路损失量	%	7.52	7.55	-0.03
**十二、6000kW 及以上电厂燃料消耗**				
发电消耗标准煤量	万 t	47290.08	42161.79	12.16
发电消耗燃煤量	万 t	65594.55	57637.06	13.81

?1994-2018 China Academic Journal Electronic Publishing House. All rights reserved. http://www.cnki.net

续表

	单 位	2002 年	2001 年	比 2001 年增长（%）
发电消耗燃油量	万 t	1089.12	1022.81	6.48
发电消耗燃气量	万 m^3	2114728	1811165	16.76
供热消耗标准煤量	万 t	5633.19	5160.31	9.16
供热消耗原煤量	万 t	7689.50	6924.35	11.05
供热消耗燃油量	万 t	153.75	148.27	3.70
供热消耗燃气量	万 m^3	1042125	915449	13.84
**十三、6000kW 及以上电厂热效率**				
电厂热效率	%	35.12	34.81	0.31
电厂供热效率	%	84.28	85.13	-0.85
能源转换总效率	%	40.36	40.55	-0.19
**十四、发用电设备比**				
发电设备容量：用电设备容量		1:2.68	1:2.46	
**十五、电力弹性系数**				
电力生产弹性系数		1.43	1.15	0.28
电力消费弹性系数		1.45	1.19	0.26
**十六、电力消费能源占一次能源的比重**	**%**	**43.56**	**42.90**	**0.66**

2002 年电力工业建设项目投资完成情况

	计算单位	2002 年	2001 年	比 2001 年增长（%）
**一、固定资产投资完成额**	**亿元**	**2296.92**	**1944.55**	**18.12**
基本建设投资	亿元	1238.81	1010.71	22.57
“大代小”投资	亿元	70.31	58.85	19.47
城乡电网投资	亿元	987.80	874.99	12.89
**（一）基本建设投资完成额**	**亿元**	**1238.81**	**1010.70**	**22.57**
**1. 按资金来源分**				
非经营基金	亿元	0.95	0.52	82.69
开行软贷	亿元	2.09	0.58	260.34

?1994-2018 China Academic Journal Electronic Publishing House. All rights reserved. http://www.cnki.net

续表

	计算单位	2002年	2001年	比2001年增长（%）
开行贷款	亿元	238.34	207.05	15.11
商行贷款	亿元	435.19	270.03	61.16
利用外资	亿元	172.46	172.43	0.02
中央专项	亿元	2.43	19.10	-87.28
三峡基金	亿元	16.81	36.94	-54.49
煤 代 油	亿元			
企业自有	亿元	179.53	161.13	11.42
中央债券	亿元	19.25	0.19	10031.58
地方债券	亿元			
地方专项	亿元	24.15	24.32	-0.70
地方其他	亿元	35.63	42.00	-15.17
其　　他	亿元	111.67	76.60	45.78
**2.按类型分**				
电　　源	亿元	677.12	592.69	14.25
电　　网	亿元	519.68	361.20	43.88
其　　他	亿元	42.01	56.82	-26.06
**3.按隶属关系分**				
国家电力公司全资	亿元	504.25	394.51	27.82
国家电力公司控股	亿元	261.36	326.65	-19.99
国家电力公司参股	亿元	232.64	193.14	20.45
地方电力企业	亿元	32.22	67.29	-52.12
其他电力企业	亿元	208.34	29.12	615.45
**4.按构成分**				
建筑工程	亿元	398.72	289.94	37.52
安装工程	亿元	161.92	146.03	10.88
设备工器具购置	亿元	381.50	324.72	17.49
其他工程	亿元	296.67	250.02	18.66
**（二）“大代小”投资完成额**	**亿元**	**70.31**	**58.85**	**19.47**
**1.按资金来源分**				
开行贷款	亿元	13.24	3.36	294.05

?1994-2018 China Academic Journal Electronic Publishing House. All rights reserved. http://www.cnki.net

续表

	计算单位	2002年	2001年	比2001年增长（%）
商行贷款	亿元	23.19	38.98	-40.51
利用外资	亿元	1.08	2.52	-57.14
企业自有	亿元	13.65	7.35	85.71
中央专项	亿元			
地方专项	亿元	3.88		
地方其他	亿元	1.42	4.85	-70.72
其　他	亿元	13.87	1.79	674.86
**2. 按构成分**				
建筑工程	亿元	13.91	11.91	16.79
安装工程	亿元	13.77	11.60	18.71
设备工器具购置	亿元	29.43	24.98	17.81
其他工程	亿元	13.19	10.36	27.32
**(三) 城乡电网建设与改造投资完成额**	**亿元**	**987.80**	**874.99**	**12.89**
**按电网构成分**				
城网部分	亿元	173.86	372.52	-53.33
农网部分	亿元	813.94	502.47	61.99
其中：农网直供部分	亿元	454.99	254.99	78.43
农网趸售部分	亿元	358.95	247.48	45.04
**二、年新增固定资产**	**亿元**	**911.92**	**898.10**	**1.54**
基本建设新增	亿元	833.05	841.24	-0.97
"大代小"新增	亿元	78.87	56.86	38.71
**三、基建新增生产能力**				
**1. 新增单机500kW及以上机组**	**万kW**	**1193.01**	**1586.87**	**-24.82**
水　电	万kW	148.22	289.55	-48.81
火　电	万kW	1044.79	1297.32	-19.47
其中：新增大中型机组	万kW	1130.36	1516.36	-25.45
水　电	万kW	123.62	266.51	-53.62
火　电	万kW	1006.74	1249.85	-19.45
**2. 新增110kV及以上线路**	**km**	**17797.95**	**14302.46**	**24.44**
500kV	km	5728.14	4335.01	32.14

?1994-2018 China Academic Journal Electronic Publishing House. All rights reserved.　http://www.cnki.net

续表

	计算单位	2002年	2001年	比2001年增长（%）
330kV	km	537.12	418.00	28.50
220kV	km	7385.10	7178.08	2.88
110kV	km	4147.59	2371.37	74.90
**3. 新增110kV及以上变电设备**	**万kVA**	**5872.00**	**5319.30**	**10.39**
500kV	万kVA	2233.00	2133.00	4.69
330kV	万kVA	129.00	153.00	-15.69
220kV	万kVA	2596.00	2621.00	-0.95
110kV	万kVA	914.00	412.30	121.68
**四、"大代小"投产能力**				
**新增单机500kW及以上机组**	**万kW**	**240.25**	**132.20**	**81.73**
火　电	万kW	240.25	131.50	82.70
**五、城乡电网建设与改造投产能力**				
**1. 投产线路**	**km**	**2069595.59**	**1452278.93**	**42.51**
220kV	km	862.01	1294.76	-33.42
110kV	km	10998.62	16093.12	-31.66
35kV	km	26536.79	34203.81	-22.42
1~10kV	km	608380.16	331669.73	83.43
低压线路	km	1422818.01	1069017.51	33.10
**2. 投产变电设备**	**万kVA**	**22660.65**	**12643.06**	**79.23**
220kV	万kVA	824.10	1197.71	-31.19
110kV	万kVA	3338.40	4627.26	-27.85
35kV	万kVA	1734.87	2454.81	-29.33
1~10kV	万kVA	16763.28	4363.28	284.19
**六、基本建设规模**				
**1. 上年结转规模**	**万kW**	**6354.06**	**5528.62**	**14.93**
水　电	万kW	3585.01	2737.62	30.95
火　电	万kW	2769.05	2791.00	-0.79
**2. 当年新开工规模**	**万kW**	**2077.50**	**2058.94**	**0.90**
水　电	万kW	963.50	963.40	0.01
火　电	万kW	1114.00	1095.54	1.69
**3. 当年在建规模**	**万kW**	**8431.56**	**7870.42**	**7.13**

?1994-2018 China Academic Journal Electronic Publishing House. All rights reserved. http://www.cnki.net

电力行业统计资料

2004 年发电技术经济指标

地 区	发电设备平均利用小时（h）			发电厂用电率（%）			标准煤耗（g/kWh）	
	合计	水电	火电	合计	水电	火电	发电	供电
全国总计	5455	3462	5991	5.95	0.47	6.85	349	376
北京市	5379	330	7322	7.84	1.93	7.94	317	348
天津市	5656		5656	6.35		6.35	323	344
河北省	6086	444	6350	6.49	2.70	6.50	350	375
山西省	6450	2667	6622	7.57	0.42	7.70	361	391
内蒙古自治区	6436	3939	6712	7.08	0.91	7.17	336	369
辽宁省	5460	2931	5715	6.94	1.33	7.21	349	376
吉林省	4147	1655	5633	6.64	0.75	7.68	362	392
黑龙江省	4612	1528	4837	7.69	1.27	7.84	376	408
上海市	6243		6243	5.22		5.22	329	347
江苏省	6385	2242	6402	5.92	1.03	5.93	346	368
浙江省	5701	1284	6923	5.69	0.62	5.68	340	361
安徽省	6155	1684	6450	5.94	0.23	6.03	341	369
福建省	4768	2093	6469	5.09	0.30	6.07	334	359
江西省	4637	1506	5634	6.58	1.20	7.04	353	380
山东省	5145		5148	7.32		7.32	337	364
河南省	5487	2862	5819	7.72	0.43	8.19	411	377
湖北省	5228	5407	4968	2.51	0.12	6.58	345	376
湖南省	4761	3620	5778	4.98	0.51	7.47	362	391
广东省	5503	2026	5908	5.00	0.60	5.42	336	358
广西自治区	4894	3914	6104	4.53	0.43	8.33	361	394
海南省	3623	1887	4155	5.62	1.06	6.24	334	358
重庆市	5065	4909	5123	8.70	2.09	11.06	386	434
四川省	4946	4671	5412	4.05	0.39	9.41	407	455
贵州省	5403	3419	6970	5.17	0.30	7.06	339	370
云南省	5153	4428	6238	3.82	0.30	7.56	368	398
西藏自治区	2908	3034		4.91	2.52	22.57		
陕西省	5515	2607	6087	7.01	0.60	7.50	358	387
甘肃省	5520	3449	7126	4.73	0.70	6.21	348	368
青海省	3804	3109	6124	3.61	1.05	7.96	396	430
宁夏自治区	7276	3211	7732	5.25	0.41	5.45	336	358
新疆自治区	4420	3772	4588	8.03	1.50	9.07	416	460

?1994-2018 China Academic Journal Electronic Publishing House. All rights reserved. http://www.cnki.net

2004 年全国分地区发电设备容量

地区	装机容量（万 kW）					比 2003 年增减（%）				
	合计	水电	火电	核电	其他	合计	水电	火电	核电	其他
全国总计	44238.73	10524.16	32948.30	683.60	81.97	13.02	10.90	13.70	10.51	47.83
北京市	451.44	105.59	345.85			2.47	—0.21	3.32		
天津市	601.35	0.50	600.85			0.00	0.00	0.00		
河北省	2073.00	78.38	1993.27		1.35	12.20	2.55	12.62		
山西省	1848.05	78.73	1769.33			16.73	—1.06	17.67		
内蒙古自治区	1432.12	56.79	1364.15		11.17	18.45	—4.08	19.43		45.87
辽宁省	1650.64	140.41	1496.03		14.20	0.96	0.57	0.97		3.26
吉林省	959.59	360.12	595.87		3.61	1.99	0.43	2.87		19.96
黑龙江省	1214.30	84.46	1125.91		3.93	2.14	1.20	1.85		
上海市	1201.83	0.00	1201.49		0.34	8.34		8.31		
江苏省	2843.36	12.65	2828.95		1.75	27.03	—8.17	27.17		
浙江省	3095.39	641.84	2143.98	305.60	3.97	29.94	6.01	39.94	27.02	—0.13
安徽省	1005.73	69.28	936.45			1.24	6.73	0.86		
福建省	1550.75	718.01	831.54		1.20	11.84	6.20	17.24		
江西省	804.59	254.99	549.60			4.29	10.51	1.63		
山东省	3292.36	5.08	3286.04		1.23	7.79	0.09	7.76		
河南省	2422.65	243.80	2178.85			20.69	0.00	23.55		
湖北省	2462.44	1511.51	950.93			24.93	31.01	16.35		
湖南省	1422.78	744.82	677.95			9.03	12.80	5.16		
广东省	4262.10	858.46	3017.29	378.00	8.34	8.72	5.89	10.80		0.05
广西自治区	941.85	504.04	437.81			22.08	11.39	37.24		
海南省	217.04	56.22	159.95		0.87	23.35	1.97	33.35		
重庆市	467.90	140.79	327.11			5.00	5.87	4.64		
四川省	2028.32	1338.29	690.03			9.96	8.44	13.05		
贵州省	1469.83	689.65	780.18			15.74	10.63	20.66		
云南省	1136.55	705.86	430.69			12.53	7.88	21.09		
西藏自治区	46.92	40.35	3.46		2.42	24.72	29.58	0.00		
陕西省	951.69	187.65	764.04			8.29	28.32	4.29		
甘肃省	867.99	356.61	497.56		13.82	7.86	8.70	4.86		
青海省	494.32	405.34	88.98			16.39	21.32	—1.77		
宁夏自治区	419.07	36.62	378.20		4.25	22.53	18.82	21.92		
新疆自治区	602.80	97.30	495.97		9.53	9.70	—1.70	12.38		4.38

?1994-2018 China Academic Journal Electronic Publishing House. All rights reserved. http://www.cnki.net

2004年全国分地区发电量

地 区	发电量（亿 kWh）					比 2003 年增减（%）				
	合计	水电	火电	核电	其他	合计	水电	火电	核电	其他
全国总计	21943.52	3309.90	18103.80	504.69	25.13	15.18	17.65	14.66	15.08	137.84
北京市	189.26	3.47	185.79			−1.87	−48.84	−0.16		
天津市	339.52	0.00	339.52			5.44	−100.00	5.47		
河北省	1255.35	5.25	1249.70		0.40	15.38	4.08	15.43		8.43
山西省	1069.58	20.32	1049.26			11.59	7.50	11.67		
内蒙古自治区	814.58	8.13	804.27		2.18	23.52	16.63	23.53		51.66
辽宁省	887.54	39.47	845.43		2.64	7.79	65.65	6.01		30.48
吉林省	394.70	61.47	332.42		0.81	16.49	50.67	11.78		
黑龙江省	548.66	13.38	534.82		0.46	10.62	21.13	10.29		
上海市	711.34	0.00	711.27		0.07	2.43		2.42		
江苏省	1639.01	3.27	1635.45		0.29	22.61	−18.24	22.71		
浙江省	1258.83	85.45	952.55	219.88	0.95	15.26	−23.18	14.64	47.34	11.29
安徽省	611.02	12.27	598.75			9.67	−21.37	10.56		
福建省	659.66	154.57	504.90		0.19	8.01	−18.21	19.80		−20.21
江西省	340.17	38.90	301.27			9.63	0.67	10.90		
山东省	1639.75	0.41	1639.18		0.16	17.49	114.95	17.46		
河南省	1162.36	68.84	1093.52			15.11	26.14	14.48		
湖北省	1125.46	695.12	430.34			43.72	79.27	8.86		
湖南省	614.23	242.36	371.86			13.95	−0.67	26.05		
广东省	2121.33	141.14	1693.89	284.81	1.49	11.90	−17.64	18.16	−1.55	
广西自治区	373.72	172.29	201.43			2.76	−10.68	17.94		
海南省	68.74	11.77	56.87		0.10	15.69	−18.88	26.97		−18.71
重庆市	229.14	56.70	165.20		7.25	12.92	43.50	1.09		
四川省	935.29	589.02	346.27			12.98	17.80	5.63		
贵州省	731.00	233.79	497.20			15.47	16.84	14.84		
云南省	536.72	293.50	243.22			16.96	9.37	27.64		
西藏自治区	12.06	10.88	0.06		1.12	19.99	19.21	11.02		
陕西省	514.81	70.43	444.39			20.55	54.44	16.50		
甘肃省	475.26	120.47	332.42		4.38	16.23	22.77	12.71		
青海省	172.78	110.71	62.08			27.22	55.14	−3.70		
宁夏自治区	263.27	9.84	252.98		0.46	31.64	19.68	31.93		
新疆自治区	266.41	36.68	227.52		2.21	12.84	2.77	14.71		6.20

?1994-2018 China Academic Journal Electronic Publishing House. All rights reserved. http://www.cnki.net

电力行业统计资料

2005年全国分地区发电设备容量

单位：万 kW

地区	容量	同比增长
全国总计	51718.48	16.91%
北京市	491.09	8.78%
天津市	617.89	2.75%
河北省	2316.57	11.75%
山西省	2307.48	24.86%
内蒙古自治区	1995.02	39.31%
辽宁省	1753.84	6.25%
吉林省	1016.55	5.94%
黑龙江省	1247.48	2.73%
上海市	1336.68	11.22%
江苏省	4270.79	50.20%
浙江省	3774.34	21.93%
安徽省	1224.78	21.78%
福建省	1762.23	13.64%
江西省	892.50	10.93%
山东省	3742.65	13.68%
河南省	2880.77	18.91%
湖北省	2741.52	11.33%
湖南省	1506.03	5.85%
广东省	4808.18	12.81%
广西自治区	1101.66	16.97%
海南省	211.48	−2.57%
重庆市	567.62	21.31%
四川省	2245.56	10.71%
贵州省	1686.78	14.76%
云南省	1275.15	12.19%
西藏自治区	48.18	2.69%
陕西省	1166.44	22.57%
甘肃省	985.73	13.56%
青海省	571.18	15.55%
宁夏自治区	518.35	23.69%
新疆自治区	654.00	8.49%

2005年全国分地区发电量

单位：亿 kWh

地区	发电量	增长率
全国总计	24975.26	13.82%
北京市	213.90	13.02%
天津市	372.57	9.73%
河北省	1338.57	6.63%
山西省	1311.97	22.66%
内蒙古自治区	1056.59	29.71%
辽宁省	904.21	1.88%
吉林省	433.43	9.81%
黑龙江省	606.67	10.57%
上海市	741.62	4.26%
江苏省	2120.00	29.35%
浙江省	1456.42	15.70%
安徽省	648.89	6.20%
福建省	778.14	17.96%
江西省	373.49	9.80%
山东省	1911.39	16.57%
河南省	1381.81	18.88%
湖北省	1289.80	14.60%
湖南省	644.41	4.91%
广东省	2278.59	7.41%
广西自治区	446.04	19.35%
海南省	82.19	19.56%
重庆市	253.70	10.72%
四川省	1018.77	8.93%
贵州省	797.65	9.12%
云南省	605.09	12.74%
西藏自治区	13.88	15.08%
陕西省	548.80	6.60%
甘肃省	505.44	10.54%
青海省	222.35	28.69%
宁夏自治区	308.73	17.27%
新疆自治区	310.14	16.41%

(C)1994-2024 China Academic Journal Electronic Publishing House. All rights reserved. http://www.cnki.net

2005年全国主要电网生产情况

电网	最高发电负荷		最高用电负荷		发电量	
	(万 kW)	增长率(%)	(万 kW)	增长率(%)	(万 kWh)	增长率(%)
国调直调	964	15.17			605.45	17.32
华北	7997	21.41	8035	21.95	4981.80	16.87
东北	3211	7.14	3141	4.87	2053.27	6.16
华东	8223	26.04	8581	23.95	5049.37	16.61
华中	5885	11.40	5959	15.17	3384.82	12.43
西北	2033	9.83	1993	7.67	1373.01	10.57
南方	5055	13.39	5220	13.31	3040.59	10.23
新疆	275	13.17	270	11.57	160.70	16.04
西藏	21	5.00	21	5.00	10.39	15.62
合计	33664	16.89	33220	16.97	20659.40	13.51

注 本表摘自国家电网公司《电力生产旬报(2005年12月下旬)》。

2005年全国分地区电厂供热生产情况

地区	供热设备容量(万 kW)	供热量(10^{10}kJ)	供热厂用电率(kWh/10^6kJ)	供热煤耗率(kg/10^6kJ)
全国总计	6981.00	192549.84	6.48	40.24
北京市	328.70	6085.59	8.35	39.24
天津市	179.15	5715.80	7.38	39.62
河北省	500.13	13810.39	7.46	40.14
山西省	323.80	5311.28	8.18	41.03
内蒙古自治区	352.30	5291.00	10.20	41.08
辽宁省	517.53	20919.58	9.02	41.95
吉林省	411.05	10130.79	8.94	40.88
黑龙江省	445.43	9984.25	8.66	42.47
上海市	366.39	5208.67	6.55	38.86
江苏省	1035.26	29759.16	5.72	37.91
浙江省	454.32	23323.31	4.95	39.00
安徽省	66.60	2079.59	6.33	40.00
福建省	31.80	2121.17	6.63	44.23
江西省				
山东省	1098.73	26680.69	6.94	41.59
河南省	241.10	4042.79	7.84	46.19
湖北省	21.40	434.45	8.56	49.89

(C)1994-2024 China Academic Journal Electronic Publishing House. All rights reserved. http://www.cnki.net

续表

地区	供热设备容量（万 kW）	供热量（10^{10}kJ）	供热厂用电率（kWh/10^6kJ）	供热煤耗率（kg/10^6kJ）
湖南省	43.70	4793.68	4.76	34.81
广东省	170.15	4518.63	5.86	37.57
广西自治区				
海南省				
重庆市	9.40	1044.82	8.99	60.71
四川省	60.79	2717.00	5.58	40.71
贵州省				
云南省				
西藏自治区				
陕西省	72.20	1383.25	7.93	43.20
甘肃省	122.30	3698.61		
青海省				
宁夏自治区	9.00	202.85	13.31	37.00
新疆自治区	119.45	3292.50	7.80	39.20

2005年发电生产主要技术经济指标

地区	发电设备平均利用小时（h）			发电厂用电率（%）	标准煤耗（g/kWh）	
	合计	水电	火电		发电	供电
全国总计	5425	3664	5865	5.87	343	370
北京市	4460	391	5698	7.73	315	348
天津市	6040		6040	6.63	321	343
河北省	6244	480	6430	6.57	345	370
山西省	6292	2729	6420	7.42	357	386
内蒙古自治区	5843	1979	6022	7.01	319	366
辽宁省	5319	4174	5445	7.03	352	380
吉林省	4543	2194	5933	6.59	358	388
黑龙江省	5011	2056	5231	7.96	369	402
上海市	5978		5974	5.05	325	343
江苏省	6109	2144	6118	5.96	330	350
浙江省	5541	1825	6369	5.59	337	358
安徽省	5916	1565	6202	5.90	337	361
福建省	4932	3695	5724	4.57	327	351
江西省	4644	2279	5376	6.48	352	381
山东省	5475	2327	5474	7.14	338	364

(C)1994-2024 China Academic Journal Electronic Publishing House. All rights reserved. http://www.cnki.net

续表

地　区	发电设备平均利用小时（h）			发电厂用电率（%）	标准煤耗（g/kWh）	
	合　计	水　电	火　电		发　电	供　电
河 南 省	5394	2810	5665	7.32	360	390
湖 北 省	5038	5060	5003	2.51	342	367
湖 南 省	4620	3190	5928	5.00	353	381
广 东 省	5421	2321	5620	5.04	333	353
广西自治区	4604	3835	5396	4.61	348	378
海 南 省	4079	1894	4670	7.66	313	377
重 庆 市	5027	3972	5442	8.05	376	418
四 川 省	4914	4727	5229	4.27	400	441
贵 州 省	5251	3111	6759	5.31	345	371
云 南 省	5239	4522	6339	3.71	363	392
西藏自治区	3926	3847				
陕 西 省	5222	3366	5571	7.16	349	378
甘 肃 省	5280	4079	6232	4.23	339	357
青 海 省	4198	3760	6219	2.69	388	420
宁夏自治区	7090	3924	7559	5.73	337	364
新疆自治区	4742	3112	5261	8.80	429	472

2005年全国装机100万kW及以上水电厂统计表

序号	单 位 名 称	机组构成	期末发电设备容量（kW）	发电量（亿kWh）	平均利用小时（h）	发电厂用电率
	百万电厂合计		19293	10491		
	其中：水电小计		4015	1557		
1	湖北三峡水电厂	14×70	980	491	5643	0.08
2	四川二滩水电厂	6×55	330	157	4768	0.16
3	湖北葛洲坝水电厂	2×17<br>19×12.5	272	162	5985	0.20
4	广东广州抽水蓄能电站	8×30	240	33	1372	0.25
5	浙江天荒坪抽水蓄能电站	6×30	180	26	1466	0.26
6	河南小浪底水电厂	6×30	180	50	2804	0.42
7	吉林白山水电站总厂	5×30<br>4×5	170	26	1512	0.52
8	青海李家峡水电厂	4×40	160	47	2906	0.42
9	福建水口水力发电厂	7×20	140	57	4049	0.14
10	云南国投大朝山水电有限公司	6×22.5	135	65	4794	0.18

(C)1994-2024 China Academic Journal Electronic Publishing House. All rights reserved. http://www.cnki.net

电力行业统计资料

2006年全国各省（市、自治区）发电设备容量

单位：万 kW

地区	全部		水电		火电		核电		风电		其他	
	2006	同比(%)	2006	同比(%)	2006	同比(%)	2006	同比(%)	2006	同比(%)	2006	同比(%)
全国总计	62369.8	20.6	13029.2	11.0	48382.2	23.6	684.6		207.2	96.3	66.5	28.1
北京市	506.1	3.1	105.3		398.4	3.9					2.4	
天津市	654.1	5.9	0.5		651.2	5.5					2.4	
河北省	2709.0	16.9	78.5	0.1	2608.7	16.8			21.8	354.1		
山西省	2745.1	19.0	79.0	0.9	2666.1	19.6						
内蒙古自治区	3028.1	51.8	81.8	44.0	2889.9	50.7			54.8	162.2	1.7	
辽宁省	1833.8	4.6	140.1	−0.2	1672.1	4.5			20.7	63.4	0.9	
吉林省	1113.1	9.5	387.2	4.1	703.9	10.7			20.8	162.4	1.3	108.3
黑龙江省	1342.5	7.6	85.3	0.8	1245.6	7.6			11.5	119.7		
上海市	1478.0	10.6	0.0		1452.6	10.8			2.4		22.9	
江苏省	5207.3	31.9	13.6	−4.9	5177.6	21.8			1.5		14.7	149.4
浙江省	4687.0	34.2	836.9	20.4	3539.1	27.9	306.6		3.7		0.6	
安徽省	1513.5	23.6	100.1	35.4	1413.4	22.8						
福建省	2204.7	25.1	895.7	8.9	1300.1	39.1			8.9	70.8		
江西省	985.6	10.4	328.8	8.9	656.8	11.2						
山东省	5005.5	33.7	55.3	973.4	4939.5	32.3			10.6	191.2		
河南省	3515.6	22.0	255.3	0.5	3260.3	24.1						
湖北省	2994.4	9.2	1832.1	2.4	1162.3	22.0						
湖南省	1938.0	28.7	864.8	10.2	1071.5	48.6					1.7	
广东省	5389.9	12.1	932.0	3.1	4061.5	15.4	378		18.3	119.9		
广西自治区	1305.8	18.5	762.4	25.3	543.4	10.2						
海南省	258.0	22.0	58.9	2.6	198.2	29.4			0.9			
重庆市	759.7	33.8	197.9	3.5	559.4	49.6					2.4	
四川省	2728.5	31.0	1773.0	18.0	955.5	27.1						
贵州省	2188.3	29.7	753.4	4.2	1435.0	48.9						
云南省	1826.1	43.2	969.8	21.2	856.4	80.2						
西藏自治区	37.9	−21.4	34.3	−18.2	1.2	−63.3					2.4	−20.3
陕西省	1193.4	8.3	216.5	9.5	972.3	8.1					4.6	
甘肃省	1093.9	11.0	429.1	7.1	644.8	12.9			11.3	117.2	8.6	
青海省	694.0	21.5	542.3	12.4	151.7	71.0						
宁夏自治区	644.1	24.3	42.9	−0.2	600.2	29.3			1.1	−90.6		
新疆自治区	789.2	20.7	176.6	29.9	593.7	17.6			18.9	41.5		

?1994-2018 China Academic Journal Electronic Publishing House. All rights reserved. http://www.cnki.net

2006年全国各省（市、自治区）发电量

单位：亿kWh

地 区	发电量	增长率（%）
全国总计	28598	14.1
北京市	211	−1.2
天津市	363	−2.6
河北省	1461	9.1
山西省	1526	16.3
内蒙古自治区	1416	34.0
辽宁省	1011	11.8
吉林省	456	5.1
黑龙江省	646	6.5
上海市	727	−2.0
江苏省	2536	19.6
浙江省	1766	21.3
安徽省	734	13.1
福建省	904	16.2
江西省	436	16.7
山东省	2273	18.9
河南省	1583	14.5
湖北省	1308	1.4
湖南省	748	16.1
广东省	2472	8.5
广西自治区	523	17.3
海南省	95	15.3
重庆市	289	13.8
四川省	1227	20.4
贵州省	980	22.9
云南省	757	25.2
西藏自治区	13	−5.7
陕西省	582	6.0
甘肃省	529	4.6
青海省	280	25.9
宁夏自治区	391	26.6
新疆自治区	356	14.8

2006年全国各省（市、自治区）发电设备利用小时

地 区	合计（h）	水电（h）	火电（h）
全国总计	5198	3393	5612
北京市	4039	404	4924
天津市	5843		5843
河北省	5939	666	6099
山西省	6275	3186	6369
内蒙古自治区	5785	2261	5916
辽宁省	5746	3362	5978
吉林省	4360	1321	6151
黑龙江省	5147	1648	5404
上海市	5102		5079
江苏省	5398	1926	5373
浙江省	5339	1765	5961
安徽省	5279	1240	5483
福建省	4669	4015	5042
江西省	4797	2730	5415
山东省	5324	94	5364
河南省	5152	3129	5331
湖北省	4774	4245	5712
湖南省	4367	3313	5117
广东省	5121	2727	5161
广西自治区	4577	3691	5643
海南省	3936	2192	4303
重庆市	4729	2799	5341
四川省	4409	4105	4901
贵州省	5342	1669	6660
云南省	4942	3871	6275
西藏自治区	3547	3531	
陕西省	4924	2221	5356
甘肃省	5202	4089	6021
青海省	4198	3760	6219
宁夏自治区	7099	3892	7499
新疆自治区	4939	3120	5578

?1994-2018 China Academic Journal Electronic Publishing House. All rights reserved. http://www.cnki.net

2006 年全国各省（市、自治区）厂用电率情况

地　区	厂用电率（%）
全国总计	5.93
北京市	7.51
天津市	6.86
河北省	6.63
山西省	7.45
内蒙古自治区	7.58
辽宁省	6.62
吉林省	6.78
黑龙江省	7.85
上海市	5.06
江苏省	5.69
浙江省	5.62
安徽省	6.05
福建省	4.51
江西省	6.17
山东省	7.12
河南省	7.06
湖北省	2.75
湖南省	4.95
广东省	5.27
广西自治区	4.45
海南省	7.56
重庆市	8.45
四川省	4.51
贵州省	6.06
云南省	4.12
西藏自治区	
陕西省	6.97
甘肃省	4.29
青海省	2.57
宁夏自治区	
新疆自治区	8.02

2006 年全国各省（市、自治区）煤耗情况

地　区	发电（g/kWh）	供电（g/kWh）
全国总计	342	367
北京市	303	333
天津市	319	342
河北省	345	370
山西省	344	371
内蒙古自治区	338	369
辽宁省	346	371
吉林省	353	382
黑龙江省	362	393
上海市	321	338
江苏省	334	354
浙江省	327	347
安徽省	335	357
福建省	328	352
江西省	350	380
山东省	355	379
河南省	348	377
湖北省	339	363
湖南省	349	375
广东省	332	352
广西自治区	345	374
海南省	296	324
重庆市	364	403
四川省	387	426
贵州省	343	368
云南省	348	373
西藏自治区		
陕西省	342	368
甘肃省	340	361
青海省	387	418
宁夏自治区	337	365
新疆自治区	425	469

?1994-2018 China Academic Journal Electronic Publishing House. All rights reserved. http://www.cnki.net

2006年全国各省(市、自治区)供热情况

地 区	供热容量(万kW)	供热量(GJ)
全国总计	8311	2275657476
北京市	304	60700224
天津市	182	58384021
河北省	616	136001157
山西省	354	55120078
内蒙古自治区	405	57091587
辽宁省	563	209382110
吉林省	404	114942841
黑龙江省	536	110995803
上海市	366	56156945
江苏省	1270	381183009
浙江省	458	313650852
安徽省	132	41598982
福建省	59	24312497
江西省		
山东省	1467	356367894
河南省	401	50894701
湖北省	113	3487398
湖南省	45	42706488
广东省	175	47862012
广西自治区		
海南省		
重庆市	16	23271522
四川省	31	36463934
贵州省		
云南省		
西藏自治区		
陕西省	72	15427858
甘肃省	122	38192041
青海省		
宁夏自治区	47	3518968
新疆自治区	173	37944554

2006年全国各省(市、自治区)全社会用电量情况

地 区	全年累计(万kWh)	同比(%)
全国合计	283678849	14.16
北京市	6115719	7.19
天津市	4336501	12.68
河北省	17348320	15.51
山西省	10976771	15.99
内蒙古自治区	8849083	32.53
辽宁省	12282742	10.60
吉林省	4124577	9.05
黑龙江	5970466	7.41
上海市	9901450	7.31
江苏省	25697523	17.16
浙江省	19092315	16.25
安徽省	6621832	13.75
福建省	8668444	14.57
江西省	4461968	13.83
山东省	22720720	15.24
河南省	15235026	12.63
湖北省	8767570	11.14
湖南省	7687729	13.99
广东省	30040334	12.36
广西区	5794618	13.59
海南省	976759	20.06
重庆市	4051961	15.25
四川省	10594386	12.40
贵州省	5819780	16.09
云南省	6456136	15.87
西藏自治区	130845	11.69
陕西省	5807287	12.36
甘肃省	5363349	9.57
青海省	2444149	18.33
宁夏自治区	3778500	24.75
新疆自治区	3561989	14.85

?1994-2018 China Academic Journal Electronic Publishing House. All rights reserved. http://www.cnki.net

电力行业统计资料

2008年全国分地区发电设备容量

装机容量（万 kW）

地区	全部			水电			火电			核电			风电			其他
	2008年	2007年	同比(%)	2008年	2007年	同比(%)	2008年	2007年	同比(%)	2008年	2007年	同比(%)	2008年	2007年	同比(%)	2008年
**全国**	**79 273**	**71 822**	**10.4**	**17 260**	**14 823**	**16.4**	**60 286**	**55 607**	**8.4**	**885**	**885**		**839**	**420**	**99.8**	**3.53**
北京市	581	495	17.4	105	105		476	390	22.1							
天津市	749	693	8.0		1		749	692	8.1							
河北省	3211	3021	6.3	154	78	96.7	2987	2902	2.9				70	41	73.1	
山西省	3604	3174	13.5	79	79	−0.1	3525	3095	13.9							
内蒙古	4886	4179	16.9	83	83		4574	3987	14.7				230	108	113.1	
辽宁省	2219	2150	3.2	143	141	1.1	1990	1972	0.9				85	35	143.6	0.90
吉林省	1300	1198	8.5	389	389	0.2	835	758	10.1				76	47	62.1	
黑龙江省	1813	1518	19.4	94	87	8.3	1657	1408	17.7				62	23	168.2	
上海市	1682	1442	16.6				1678	1415	18.6				4	2	61.5	0.22
江苏省	5442	5599	−2.8	114	14		5068	5334	−5.0	200	200		61	25	145.9	
浙江省	5317	5112	4.0	896	852	5.2	4099	3949	3.8	307	307		15	4	286.5	
安徽省	2638	1927	36.9	156	151	3.4	2482	1776	39.7							
福建省	2627	2398	9.5	1058	980	7.9	1543	1391	10.9				26	23	12.5	
江西省	1308	1284	1.8	371	357	4.0	934	927	0.7				3			
山东省	5736	5540	3.5	105	105		5593	5414	3.3				37	21	71.7	
河南省	4572	4129	10.7	302	274	10.1	4268	3854	10.7				3			
湖北省	4328	3708	16.7	2905	2402	20.9	1421	1304	8.9				1	1		
湖南省	2508	2260	10.9	1065	922	15.4	1443	1336	8.0							
广东省	6008	5886	2.1	1028	1011	1.7	4573	4471	2.3	378	378		29	25	16.2	
广西区	2424	1975	22.7	1397	1044	33.9	1027	931	10.3							
海南省	279	302	−7.5	41	59	−30.3	237	240	−1.4				1	1		
重庆市	1073	863	24.3	406	224	81.6	666	637	4.6							
四川省	3501	3186	9.9	2224	1986	12.0	1277	1200	6.4							
贵州省	2664	2417	10.2	947	821	15.3	1717	1596	7.6							
云南省	2585	2221	16.4	1574	1158	35.8	1003	1063	−5.6				8			
西藏区	54	42	27.5	43	39	12.6	8	1								2.41
陕西省	1966	1416	38.9	181	179	1.1	1785	1229	45.2							
甘肃省	1502	1259	19.3	544	440	23.6	898	784	14.5				60	26	129.8	
青海省	791	774	2.2	591	583	1.3	200	190	5.2							
宁夏区	814	751	8.5	43	43		754	703	7.3				17	5	248.5	
新疆区	1090	903	20.7	219	214	2.1	820	656	25.0				51	33	57.1	

注 表中其他部分的统计口径与2007年不同。

?1994-2018 China Academic Journal Electronic Publishing House. All rights reserved.   http://www.cnki.net

2008 年全国分地区发电量

地区	全部			水电			火电			核电			风电			其他
	2008年	2007年	同比(%)	2008年	2007年	同比(%)	2008年	2007年	同比(%)	2008年	2007年	同比(%)	2008年	2007年	同比(%)	2008年
**全国**	**34 510**	**32 644**	**5.7**	**5655**	**4714**	**20.0**	**28 030**	**27 207**	**3.0**	**692**	**629**	**10.1**	**130.8**	**57.1**	**129.0**	**1.7**
北京市	247	227	9.0	5	4	5.5	243	223	9.1							
天津市	397	399	−0.4		0.1		397	399	−0.4							
河北省	1601	1646	−2.7	7	6	19.6	1580	1633	−3.2				13.6	7.1	90.4	
山西省	1786	1759	1.5	23	26	−10.0	1762	1734	1.7							
内蒙区	2057	1830	12.4	11	14	−21.9	2008	1801	11.5				37.4	14.4	160.5	
辽宁省	1139	1113	2.4	42	44	−3.2	1085	1065	1.9				10.8	3.3	231.5	0.2
吉林省	526	501	4.9	48	56	−14.5	464	437	6.2				13.7	6.4	113.1	
黑龙江省	739	699	5.8	14	12	16.2	715	684	4.6				10.9	3.6	203.4	
上海市	795	742	7.2				794	726	9.4				0.6	0.4	39.8	0.01
江苏省	2887	2825	2.2	3.3	3.2	3.4	2735	2709	1.0	141	100	40.5	7.8	2.1	266.0	
浙江省	2134	2080	2.6	147	130	12.7	1748	1723	1.4	238	227	5.1	1.3	0.5	162.2	
安徽省	1103	868	27.0	28	20	41.0	1074	848	26.6							
福建省	1085	1039	4.4	332	312	6.4	748	723	3.4				5.9	4.0	45.2	
江西省	494	494	−0.1	89	73	21.5	405	421	−3.9				0.01			
山东省	2697	2596	3.9	2.0	2.0	13.7	2689	2591	3.8				5.4	2.7	104.2	
河南省	1972	1864	5.8	81	91	−10.4	1890	1773	6.6				0.2			
湖北省	1752	1541	13.7	1199	933	28.6	553	609	−9.2				0.2	0.04	425.8	
湖南省	850	837	1.5	312	294	6.1	537	542	−0.8				0.01			
广东省	2682	2695	−0.5	256	232	10.0	2107	2157	−2.3	313	302	3.8	6.0	3.9	55.9	
广西区	855	685	24.9	513	324	58.4	342	361	−5.2							
海南省	118	114	3.5	11	12	−11.2	107	101	6.1				0.14	0.13	14.6	
重庆市	403	366	10.0	116	77	52.0	286	288	−0.7							
四川省	1236	1226	0.8	836	775	7.8	401	451	−11.1							
贵州省	1179	1138	3.7	366	295	24.3	813	843	−3.5							
云南省	1040	905	14.9	622	431	44.2	418	474	−11.8				0.3			
西藏区	16	15	6.8	14.2	13.6	4.5	0.13	0.01								1.4
陕西省	769	645	19.3	54	52	5.4	715	591	21.0							
甘肃省	691	620	11.5	217	189	14.7	468	424	10.3				6.3	3.1	103.8	
青海省	322	302	6.6	216	205	5.1	107	97	9.8							
宁夏区	459	453	1.4	16	17	−7.2	440	435	1.3				2.4	0.5	370.7	
新疆区	479	420	14.0	74	70	6.6	397	346	14.9				7.8	5.0	56.5	

注　表中其他部分的统计口径与 2007 年不同。

?1994-2018 China Academic Journal Electronic Publishing House. All rights reserved.   http://www.cnki.net

2008年全国新增发电机组设备能力

万kW

地区	合计	其中									
		水电		火电					核电	风电	其他
			其中：抽水蓄能		燃煤	燃气	燃油	其他			
**全国**	**9202**	**2148**	**175**	**6555**	**6040**	**321**	**8**	**185**		**499**	
北京市	145			145	16	129					
天津市	60			60	60						
河北省	365	75	75	261	257	2		2		30	
山西省	459			450	418			32		10	
内蒙古	773			611	515			96		162	
辽宁省	290			240	240					50	
吉林省	112			70	69			1		42	
黑龙江	219	1		189	184			6		29	
上海市	267			266	266					1	
江苏省	343	100	100	192	179			13		50	
浙江省	417	55		347	269	78				14	
安徽省	750			750	744			6			
福建省	372	41		316	246	70				15	
江西省	73			70	70					3	
山东省	326			309	307			2		17	
河南省	460	4		457	418	39					
湖北省	608	488		120	120						
湖南省	241	115		126	126						
广东省	300			300	300						
广西区	403	342		62	60			2			
海南省	17	0		12			2	10		5	
重庆市	218	185		33	33						
四川省	292	200		92	92						
贵州省	306	126		180	180						
云南省	316	306		3				3		8	
西藏区	11	3		8			7	2			
陕西省	535			535	522	3		10			
甘肃省	231	77		120	120					34	
青海省	22	9		14	14						
宁夏区	57			43	43	0				13	
新疆区	214	23		175	174			1		17	

?1994-2018 China Academic Journal Electronic Publishing House. All rights reserved.   http://www.cnki.net

2008 年全社会用电量情况

亿 kWh

地　区	2008 年	同比（%）
**全国合计**	**34 379.69**	**5.49**
北京市	689.72	3.40
天津市	515.88	4.24
河北省	2095.02	4.04
山西省	1314.33	−2.56
内蒙古	1220.57	5.20
辽宁省	1412.00	3.86
吉林省	496.49	7.32
黑龙江	669.90	6.51
上海市	1138.22	6.14
江苏省	3118.32	5.63
浙江省	2322.87	6.10
安徽省	858.88	11.67
福建省	1073.55	7.32
江西省	546.77	6.98
山东省	2726.97	5.04
河南省	1970.77	9.00
湖北省	1058.53	7.01
湖南省	916.79	2.94
广东省	3506.78	3.32
广西区	760.79	11.69
海南省	122.97	8.58
重庆市	487.69	8.56
四川省	1213.39	3.05
贵州省	679.18	1.51
云南省	829.44	7.76
西藏区	15.87	6.85
陕西省	708.03	8.25
甘肃省	677.76	10.25
青海省	313.23	9.74
宁夏区	439.62	−0.04
新疆区	479.37	15.98

?1994-2018 China Academic Journal Electronic Publishing House. All rights reserved.　http://www.cnki.net

2011
中国电力年鉴

电力行业统计资料

2010年电力统计基本数据一览表

统计口径	单位	2010年	2009年	比2009年增长(%)
**一、发电装机容量**	**万kW**	**96 641.30**	**87 409.72**	**10.56**
水电	万kW	21 605.72	19 629.02	10.07
火电	万kW	70 967.21	65 107.63	9.00
核电	万kW	1082.40	907.82	19.23
风电	万kW	2957.55	1759.94	68.05
地热、潮汐	万kW	2.81	2.81	
太阳能	万kW	25.62	2.50	925.14
**6000千瓦及以上火电厂设备容量**	**万kW**	**70 391.13**	**64 522.91**	**9.09**
其中：燃煤	万kW	64 660.64	59 215.17	9.20
燃油	万kW	877.99	823.00	6.68
燃气	万kW	2642.40	2402.96	9.96
煤矸石	万kW	836.80	674.00	24.15
生物质	万kW	170.45	108.80	56.66
垃圾	万kW	170.94	130.29	31.20
余温、余压、余气等	万kW	1007.13	1168.70	−13.82
**二、关停小火电机组容量**	**万kW**	**1305.24**	**1812.79**	**−28.00**
**三、新增发电设备能力**	**万kW**	**9124.00**	**9667.35**	**−5.62**
水电	万kW	1642.85	2105.70	−21.98
火电	万kW	5830.56	6585.76	−11.47
其中：燃煤	万kW	5425.84	6186.71	−12.30
燃气	万kW	123.50	127.34	−3.02
煤矸石	万kW	130.14	155.00	−16.04
生物质	万kW	43.11	23.20	85.82
垃圾	万kW	14.03	11.34	23.72
余温、余压、余气等	万kW	81.77	68.25	19.81
核电	万kW	173.69		
风电	万kW	1457.31	973.00	49.77
太阳能及地热	万kW	19.59	2.79	602.15
**四、年底电源在建规模**	**万kW**	**18 338.94**	**18 355.56**	**−0.09**
水电	万kW	6551.05	6724.80	−2.58

?1994-2018 China Academic Journal Electronic Publishing House. All rights reserved. http://www.cnki.net

续表

统计口径	单 位	2010年	2009年	比2009年增长(%)
火 电	万kW	7400.13	7749.10	-4.50
核 电	万kW	3395.42	3140.00	8.13
风 电	万kW	966.54	720.75	34.10
**五、基建新增110kV及以上输电线路长度**	**km**	**76 574**	**69 217**	**10.63**
其中：1000kV	km		640	-100.00
±800kV	km	1907	1375	38.69
750kV	km	4491	2021	122.24
±660kV	km	1335		
500kV	km	10 793	12 959	-16.71
其中：±500kV	km	3189	574	455.61
330kV	km	1699	1766	-3.78
220kV	km	24 499	22 697	7.94
110kV	km	31 850	27 760	14.73
**六、基建新增110kV及以上变电设备容量**	**万kVA**	**35 335**	**36 155**	**-2.27**
其中：1000kV	万kVA		600	-100.00
750kV	万kVA	1920	1080	77.78
500kV	万kVA	9495	11 545	-17.76
330kV	万kVA	960	780	23.08
220kV	万kVA	13 438	12 801	4.98
110kV	万kVA	9522	9349	1.85
**七、单机6000kW及以上机组平均单机容量**				
水电：单机容量	万kW/台	5.61	5.51	0.10
机组台数	台	3097	2860	237
机组容量	万kW	17 386.83	15 768.11	1619
火电：单机容量	万kW/台	10.88	10.31	0.57
机组台数	台	6373	6221	152
机组容量	万kW	69 349.28	64 133.47	5216
**八、35kV及以上输电线路长度**	**km**	**1 336 772**	**1 231 883**	**8.51**
其中：1000kV	km	1006	640	57.25
±800kV	km	3334	1375	142.45
750kV	km	6685	2640	153.22
±660kV	km	1095		
500kV	km	135 180	124 559	8.53
其中：±500kV	km	8081	6901	17.10
330kV	km	20 338	19 156	6.17
220kV	km	277 988	253 573	9.63
110kV	km	458 477	422 863	8.42
35kV	km	432 668	407 077	6.29
**九、35kV及以上变电设备容量(交流)**	**万kVA**	**361 742**	**319 542**	**13.21**
其中：1000kV	万kVA	600	600	
750kV	万kVA	3870	1740	122.41

?1994-2018 China Academic Journal Electronic Publishing House. All rights reserved. http://www.cnki.net

续表

统计口径	单位	2010年	2009年	比2009年增长(%)
500kV	万kVA	69 843	60 114	16.18
330kV	万kVA	6457	5523	16.91
220kV	万kVA	118 247	103 040	14.76
110kV	万kVA	125 224	112 958	10.86
35kV	万kVA	37 501	35 567	5.44
**十、电力投资当年完成**	**亿元**	**7417.47**	**7701.61**	**−3.69**
**1. 电源投资**	**亿元**	**3969.36**	**3803.31**	**4.37**
其中：水　电	亿元	819.18	867.19	−5.54
火　电	亿元	1426.14	1543.56	−7.61
核　电	亿元	647.57	584.01	10.88
风　电	亿元	1037.55	781.78	32.72
**2. 电网投资**	**亿元**	**3448.10**	**3898.30**	**−11.55**
送变电	亿元	3338.45	3776.91	−11.61
其中：直流	亿元	283.54	276.76	2.45
±800kV	亿元	116.05	189.44	−38.74
±660kV	亿元	71.40	9.08	686.41
±500kV	亿元	81.46	78.24	4.11
±400kV	亿元	14.62		
其中：交流	亿元	3054.92	3099.79	−1.45
1000kV	亿元	1.97	4.88	−59.65
750kV	亿元	173.85	125.46	38.57
500kV	亿元	444.13	699.74	−36.53
330kV	亿元	37.25	56.93	−34.57
220kV	亿元	1083.16	891.28	21.53
110kV及以下	亿元	1314.55	1321.50	−0.53
其他(含小型基建)	亿元	109.65	121.39	−9.67
**十一、发电量**	**亿kWh**	**42 277.71**	**36 811.86**	**14.85**
水　电	亿kWh	6867.36	5716.82	20.13
火　电	亿kWh	34 166.28	30 116.87	13.45
核　电	亿kWh	747.42	700.50	6.70
风　电	亿kWh	494.00	276.15	78.89
地热、潮汐、太阳能等	亿kWh	2.65	1.52	73.71
**6000kW及以上火电厂发电量**	**亿kWh**	**34 086.11**	**30 050.07**	**13.43**
其中：燃　煤	亿kWh	32 162.69	28 347.71	13.46
燃　油	亿kWh	161.89	170.76	−5.20
燃　气	亿kWh	776.27	565.59	37.25
煤矸石	亿kWh	361.00	317.76	13.61
生物质	亿kWh	74.25	52.17	42.33
垃　圾	亿kWh	86.84	67.48	28.70
余温、余压、余气等	亿kWh	455.99	528.60	−13.74

?1994-2018 China Academic Journal Electronic Publishing House. All rights reserved. http://www.cnki.net

续表

统计口径	单 位	2010 年	2009 年	比 2009 年增长(%)
**十二、6000kW 及以上电厂供热量**	**万 GJ**	**280 759.99**	**258 198.07**	**8.74**
**十三、6000kW 及以上电厂供电煤耗**	**g/kWh**	**333**	**340**	**−7**
**十四、6000kW 及以上电厂发电煤耗**	**g/kWh**	**312**	**320**	**−8**
**十五、6000kW 及以上电厂厂用电率**	**%**	**5.43**	**5.76**	**−0.32**
水 电	%	0.33	0.40	−0.07
火 电	%	6.33	6.62	−0.29
**十六、6000kW 及以上电厂利用小时数**	**h**	**4650**	**4546**	**104**
水 电	h	3404	3328	76
火 电	h	5031	4865	166
核 电	h	7840	7716	124
风 电	h	2047	2077	−30
**十七、供、售电量及线损**				
供电量	亿 kWh	38 041.72	32 613.74	6.52
售电量	亿 kWh	35 556.32	30 423.09	16.87
线损电量	亿 kWh	2485.39	2190.65	13.45
线路损失率	%	6.53	6.72	−0.19
**十八、全社会用电量**	**亿 kWh**	**41 998.82**	**36 598.42**	**14.76**
**A. 全行业用电合计**	**亿 kWh**	**36 904.87**	**32 023.23**	**15.24**
第一产业	亿 kWh	976.49	939.90	3.89
第二产业	亿 kWh	31 450.01	27 139.60	15.88
其中：工业	亿 kWh	30 966.77	26 757.69	15.73
1. 轻工业	亿 kWh	5336.23	4635.56	15.12
2. 重工业	亿 kWh	25 630.53	22 122.13	15.86
第三产业	亿 kWh	4478.36	3943.73	13.56
**B. 城乡居民生活用电合计**	**亿 kWh**	**5093.96**	**4575.19**	**11.34**
其中：城镇居民	亿 kWh	2959.87	2670.03	10.86
乡村居民	亿 kWh	2134.09	1905.16	12.02
**十九、6000kW 及以上电厂燃料消耗**				
发电消耗标准煤量	万 t	102 006.24	91 478.17	11.51
发电消耗原煤量	万 t	158 970.92	139 669.62	13.82
供热消耗标准煤量	万 t	11 171.65	10 199.06	9.54
供热消耗原煤量	万 t	16 769.41	14 959.97	12.10
**二十、6000kW 及以上电厂热效率**				
电厂热效率	%	39.42	38.44	0.99
电厂供热效率	%	85.75	86.38	−0.63
能源转换总效率	%	44.00	43.25	0.75
**二十一、发用电设备比**				
发电设备容量：用电设备容量		1∶3.18	1∶3.16	
**二十二、电力弹性系数**				
电力生产弹性系数		1.44	0.72	0.72
电力消费弹性系数		1.43	0.70	0.73

?1994-2018 China Academic Journal Electronic Publishing House. All rights reserved. http://www.cnki.net

2013
中国电力年鉴

电力行业统计资料❶

2012年电力统计基本数据一览表

	单 位	2012年	2011年	同比增长(%)
一、发电量	亿kWh	49 865	47 306	5.41
水电	亿kWh	8556	6681	28.06
其中：抽水蓄能	亿kWh	93	109	－14.56
火电	亿kWh	39 255	39 003	0.65
核电	亿kWh	983	872	12.75
风电	亿kWh	1030	741	39.15
太阳能发电	亿kWh	36	6	494.22
其他	亿kWh	5	2	117.20
6000kW及以上火电厂发电量	亿kWh	39 160	38 893	0.69
燃煤	亿kWh	37 104	36 961	0.38
其中：煤矸石发电	亿kWh	746	672	11.02
燃油	亿kWh	54	59	－9.01
燃气	亿kWh	1092	1088	0.39
其中：煤层气发电	亿kWh	15	18	－16.74
其他	亿kWh	911	785	16.09
其中：余温、余气、余压发电	亿kWh	594	552	7.70
垃圾焚烧发电	亿kWh	120	97	23.78
秸秆、蔗渣、林木质发电	亿kWh	196	135	44.78
二、全社会用电量	亿kWh	49 657	47 022	5.60
A. 全行业用电合计	亿kWh	43 429	41 401	4.90
第一产业	亿kWh	1003	1014	1.12
第二产业	亿kWh	36 733	35 282	4.11
其中：工业	亿kWh	36 122	34 710	4.07
1. 轻工业	亿kWh	6114	5825	4.96
2. 重工业	亿kWh	30 008	28 885	3.89
第三产业	亿kWh	5693	5105	11.52
B. 城乡居民生活用电合计	亿kWh	6228	5621	10.79

❶ 本统计资料未包含香港、澳门地区及台湾省资料。

?1994-2018 China Academic Journal Electronic Publishing House. All rights reserved. http://www.cnki.net

续表

	单位	2012年	2011年	同比增长(%)
其中：城镇居民	亿kWh	3562	3202	11.25
乡村居民	亿kWh	2666	2419	10.18
三、发电装机容量	万kW	114 676	106 253	7.93
水电	万kW	24 947	23 298	7.08
其中：抽水蓄能	万kW	2033	1838	10.61
火电	万kW	81 968	76 834	6.68
核电	万kW	1257	1257	
风电	万kW	6142	4623	32.86
太阳能发电	万kW	341	212	60.63
其他	万kW	20.5	19.0	7.84
6000kW及以上火电厂装机容量	万kW	81 426	76 302	6.71
燃煤	万kW	75 382	70 929	6.28
其中：煤矸石发电	万kW	1574	1295	21.54
燃油	万kW	301	328	−8.23
燃气	万kW	3717	3415	8.84
其中：煤层气发电	万kW	29	38	−23.92
其他	万kW	2025	1631	24.19
其中：余温、余气、余发电	万kW	1256	1072	17.23
垃圾焚烧发电	万kW	251	210	19.91
秸秆、蔗渣、林木质发电	万kW	518	349	48.12
四、35kV及以上输电线路回路长度	km	1 479 963	1 409 698	4.98
1000kV	km	639	639	
±800kV	km	5466	3334	63.96
750kV	km	10 088	10 005	0.83
±660kV	km	1400	1400	
500kV	km	146 250	140 263	4.27
其中：±500kV	km	9145	8837	3.49
±400kV	km	1051	1051	
330kV	km	22 701	22 267	1.95
220kV	km	318 217	295 978	7.51
110kV(含66kV)	km	517 983	491 322	5.43
35kV	km	456 168	443 440	2.87
五、35kV及以上变电设备容量	万kVA	445 899	408 398	9.18
1000kV	万kVA	1800	1800	
±800kV	万kVA	4360	2669	63.34
750kV	万kVA	5320	5320	
±660kV	万kVA	946	946	

?1994-2018 China Academic Journal Electronic Publishing House. All rights reserved. http://www.cnki.net

续表

	单 位	2012年	2011年	同比增长(%)
500kV	万kVA	90 625	82 109	10.37
其中：±500kV	万kVA	7230	6011	20.27
±400kV	万kVA	141	71	98.86
330kV	万kVA	7714	7424	3.91
220kV	万kVA	144 228	131 060	10.05
110kV(含66kV)	万kVA	149 231	137 776	8.31
35kV	万kVA	41 534	39 223	5.89
六、新增发电装机容量	万kW	8315	9436	−11.88
水电	万kW	1676	1283	30.63
其中：抽水蓄能	万kW	165	175	−5.71
火电	万kW	5236	6241	−16.10
其中：燃煤	万kW	4788	5837	−17.97
其中：煤矸石发电	万kW	156	183	−14.75
燃油	万kW			
燃气	万kW	247	237	4.38
其中：煤层气发电	万kW	1		
其他	万kW	201	168	19.57
其中：余温、余气、余压	万kW	105	77	35.72
垃圾焚烧发电	万kW	20	26	−22.04
秸秆、蔗渣、林木质发电	万kW	75	65	14.98
核电	万kW		175	
风电	万kW	1296	1528	−15.18
太阳能发电	万kW	107	196	−45.23
其他	万kW		13.1	
七、火电机组退役和关停容量	万kW	616	955	−35.52
八、年底主要发电企业电源在建规模	万kW	16 235	17 084	−4.97
水电	万kW	6648	7121	−6.64
火电	万kW	5166	5558	−7.05
核电	万kW	3383	3347	1.08
风电	万kW	971	1047	−7.23
九、新增110kV及以上输电线路长度	km	66 269	66 903	−0.95
1000kV	km		1[illegible]4	
±800kV	km	2090		
750kV	km	741	2740	−72.96
±660kV	km			
500kV	km	4747	7331	−35.24
其中：±500kV	km			

?1994-2018 China Academic Journal Electronic Publishing House. All rights reserved. http://www.cnki.net

续表

	单 位	2012年	2011年	同比增长(%)
±400kV	km		1038	
330kV	km	219	965	−77.32
220kV	km	26 431	24 129	9.54
110kV(含66kV)	km	32 040	30 698	4.37
十、新增110kV及以上变电设备容量	万kVA	28 835	31 713	−9.07
1000kV	万kVA		1200	
±800kV	万kVA	1440		
750kV	万kVA		1660	
±660kV	万kVA		400	
500kV	万kVA	7650	6465	18.33
其中：±500kV	万kVA	450	600	−25.00
±400kV	万kVA		120	
330kV	万kVA	372	614	−39.41
220kV	万kVA	11 269	12 032	−6.34
110kV(含66kV)	万kVA	9994	9222	8.37
十一、电力投资当年完成	亿元	7393	7614	−2.90
1. 电源投资	亿元	3732	3927	−4.98
水电	亿元	1239	971	27.64
火电	亿元	1002	1133	−11.55
核电	亿元	784	764	2.69
风电	亿元	607	902	−32.70
太阳能发电	亿元	99	155	−36.18
其他	亿元		2.5	
2. 电网投资	亿元	3661	3687	−0.69
送变电	亿元	3458	3498	−1.13
其中：直流	亿元	278	222	25.19
交流	亿元	3180	3275	−2.90
其他	亿元	203	189	7.38
十二、单机6000kW及以上机组平均单机容量				
水电：单机容量	万kW/台	5.77	5.66	0.11
机组台数	台	3530	3328	202
机组容量	万kW	20 377	18 834	1543
火电：单机容量	万kW/台	11.80	11.40	0.40
机组台数	台	6805	6595	210
机组容量	万kW	80 302	75 215	5087
十三、6000kW及以上电厂供热量	万GJ	307 749	297 859	3.32
十四、6000kW及以上电厂发电标准煤耗	g/kWh	305	308	−3

?1994-2018 China Academic Journal Electronic Publishing House. All rights reserved. http://www.cnki.net

续表

	单 位	2012年	2011年	同比增长（%）
十五、6000kW及以上电厂供电标准煤耗	g/kWh	325	329	−4
十六、6000kW及以上电厂厂用电率	%	5.10	5.39	−0.29
水电	%	0.33	0.36	−0.03
火电	%	6.08	6.23	−0.15
十七、6000kW及以上电厂发电设备利用小时	h	4579	4730	−151
水电	h	3591	3019	572
其中：抽水蓄能	h	592	619	−27
火电	h	4982	5305	−323
核电	h	7855	7759	96
风电	h	1929	1875	54
十八、6000kW及以上电厂燃料消耗				
发电消耗标准煤量	万t	114 770	114 400	0.32
发电消耗原煤量	万t	178 968	182 382	−1.87
供热消耗标准煤量	万t	12 247	11 854	3.32
供热消耗原煤量	万t	18 447	18 262	1.01
十九、6000kW及以上火电厂热效率				
电厂热效率	%	41.91	41.76	0.15
电厂供热效率	%	85.74	85.74	
电厂能源转换总效率	%	46.10	44.37	1.73
二十、供、售电量及线损				
供电量	亿kWh	44 798	42 768	4.75
售电量	亿kWh	41 781	39 980	4.51
线损电量	亿kWh	3018	2788	8.23
线路损失率	%	6.74	6.52	0.22
二十一、发用电设备比				
发电装机容量：用电设备容量		1∶3.47	1∶3.31	
二十二、电力弹性系数				
电力生产弹性系数		0.69	1.28	−0.58
电力消费弹性系数		0.72	1.29	−0.57

注 1. 2012年，全国基建新增生物质发电装机容量99万kW，同比增长8.51%；年底6000kW及以上电厂生物质装机容量769万kW，同比增长37.57%；全年6000kW及以上电厂生物质发电量316亿kWh，同比增长35.62%；

2. 本年35kV及以上变电设备容量包含换流站两端变压器容量，2011年同期数据相应调整。

?1994-2018 China Academic Journal Electronic Publishing House. All rights reserved. http://www.cnki.net

电力统计基本数据一览表

基本数据项目	单位	2014 年	2013 年	比 2013 年增长（±、%）
**一、发电量**	**亿 kWh**	**56 045**	**53 721**	**4.33**
水电	亿 kWh	10 601	8921	18.83
其中：抽水蓄能	亿 kWh	132	107	23.51
火电	亿 kWh	42 274	42 216	0.14
其中：燃煤	亿 kWh	39 510	39 805	−0.74
燃气	亿 kWh	1333	1164	14.52
燃油	亿 kWh	44	52	−15.10
核电	亿 kWh	1332	1115	19.48
风电	亿 kWh	1598	1383	15.55
太阳能发电	亿 kWh	235	84	180.78
其他	亿 kWh	5	3	92.22
**6000kW 及以上火电厂发电量**	**亿 kWh**	**42 169**	**42 134**	**0.08**
燃煤	亿 kWh	39 449	39 776	−0.82
其中：煤矸石发电	亿 kWh	1587	1042	52.22
燃气	亿 kWh	1322	1156	14.36
其中：常规燃气	亿 kWh	1288	1129	14.07
煤层气发电	亿 kWh	21	27	−19.86
燃油	亿 kWh	44	52	−15.08
其他	亿 kWh	1354	1120	20.82
其中：余温、余气、余压发电	亿 kWh	892	737	21.02
垃圾焚烧发电	亿 kWh	245	176	38.98
秸秆、蔗渣、林木质发电	亿 kWh	216	207	4.62
**二、全社会用电量**	**亿 kWh**	**55 637**	**53 423**	**4.14**
**1. 全行业用电合计**	**亿 kWh**	**48 701**	**46 630**	**4.44**
第一产业	亿 kWh	1013	1026	−1.22
第二产业	亿 kWh	41 017	39 335	4.28
其中：工业	亿 kWh	40 296	38 660	4.23
其中：轻工业	亿 kWh	6693	6427	4.15
重工业	亿 kWh	33 603	32 233	4.25
第三产业	亿 kWh	6670	6269	6.39
**2. 城乡居民生活用电合计**	**亿 kWh**	**6936**	**6793**	**2.10**
其中：城镇居民	亿 kWh	3933	3860	1.89

?1994-2018 China Academic Journal Electronic Publishing House. All rights reserved. http://www.cnki.net

续表

基本数据项目	单位	2014 年	2013 年	比 2013 年增长（±、%）
乡村居民	亿 kWh	3003	2933	2.39
**三、发电装机容量**	**万 kW**	**137 018**	**125 768**	**8.95**
水电	万 kW	30 486	28 044	8.71
其中：抽水蓄能	万 kW	2211	2153	2.68
火电	万 kW	92 363	87 009	6.15
其中：燃煤	万 kW	83 233	79 578	4.59
燃气	万 kW	5697	4277	33.19
燃油	万 kW	512	590	−13.33
核电	万 kW	2008	1466	36.97
风电	万 kW	9657	7652	26.20
太阳能发电	万 kW	2486	1589	56.50
其他	万 kW	19	8	128.45
**6000kW 及以上火电厂装机容量**	**万 kW**	**91 861**	**86 473**	**6.23**
燃煤	万 kW	83 106	79 450	4.60
其中：煤矸石发电	万 kW	3349	2062	62.42
燃气	万 kW	5666	4252	33.26
其中：常规燃气	万 kW	5526	4157	32.93
煤层气发电	万 kW	91	95	−4.64
燃油	万 kW	270	293	−7.70
其他	万 kW	2819	2478	13.74
其中：余温、余气、余压发电	万 kW	1838	1610	14.15
垃圾焚烧发电	万 kW	469	359	30.58
秸秆、蔗渣、林木质发电	万 kW	512	509	0.55
**四、35kV 及以上输电线路回路长度**	**km**	**1 628 472**	**1 554 236**	**4.78**
**1. 交流**	**km**	**1 603 488**	**1 534 248**	**4.51**
其中：1000kV	km	3111	1936	60.70
750kV	km	13 881	12 666	9.59
500kV	km	152 107	146 166	4.06
330kV	km	25 146	24 065	4.49
220kV	km	358 377	339 075	5.69
110kV	km	566 571	545 815	3.80
35kV	km	484 296	464 525	4.26
**2. 直流**	**km**	**24 984**	**19 988**	**24.99**
其中：±800kV	km	10 132	6904	46.75
±660kV	km	1336	1400	−4.54
±500kV	km	11 875	10 653	11.48
±400kV	km	1640	1031	59.00
**五、35kV 及以上变电设备容量**	**万 kVA**	**526 685**	**483 427**	**8.95**
**1. 交流**	**万 kVA**	**509 134**	**470 047**	**8.32**
其中：1000kV	万 kVA	5700	3900	46.15

?1994-2018 China Academic Journal Electronic Publishing House. All rights reserved. http://www.cnki.net

续表

基本数据项目	单位	2014 年	2013 年	比 2013 年增长（±、%）
750kV	万 kVA	8090	6500	24.47
500kV	万 kVA	100 011	90 112	10.99
330kV	万 kVA	10 493	8575	22.36
220kV	万 kVA	167 342	155 699	7.48
110kV	万 kVA	171 588	161 661	6.14
35kV	万 kVA	45 909	43 600	5.30
**2. 直流**	**万 kVA**	**17 551**	**13 380**	**31.17**
其中：±800kV	万 kVA	3180	4654	−31.67
±660kV	万 kVA		948	−100.00
±500kV	万 kVA	14 230	7637	86.32
±400kV	万 kVA	141	141	0.00
**六、新增发电装机容量**	**万 kW**	**10 443**	**10 222**	**2.16**
水电	万 kW	2180	3096	−29.59
其中：抽水蓄能	万 kW	60	120	−50.00
火电	万 kW	4791	4175	14.74
其中：燃煤	万 kW	3498	3447	1.47
其中：煤矸石发电	万 kW	30	61	−50.50
燃气	万 kW	946	440	115.18
其中：常规燃气	万 kW	938	439	113.91
煤层气发电		8		
燃油	万 kW		15	−100.00
其他	万 kW	346	273	26.83
其中：余温、余气、余压	万 kW	254	182	39.43
垃圾焚烧发电	万 kW	21	34	−39.36
秸秆、蔗渣、林木质发电	万 kW	71	56	26.32
核电	万 kW	547	221	147.84
风电	万 kW	2101	1487	41.25
太阳能发电	万 kW	825	1243	−33.63
其他	万 kW			
**七、火电机组退役和关停容量**	**万 kW**	**909**	**803**	**13.15**
**八、年底主要发电企业电源项目在建规模**	**万 kW**	**14 500**	**15 950**	**−9.10**
水电	万 kW	4328	5129	−15.61
火电	万 kW	5524	5953	−7.21
核电	万 kW	2863	3387	−15.49
风电	万 kW	1676	1457	15.04
**九、新增直流输电线路长度及换流容量**				
**1. 线路长度**	**km**	**2876**	**4846**	**−40.65**
其中：±800kV	km	1653	3623	−54.37
±660kV	km			
±500kV	km	1223	1223	0.00

?1994-2018 China Academic Journal Electronic Publishing House. All rights reserved. http://www.cnki.net

续表

基本数据项目	单位	2014 年	2013 年	比 2013 年增长（±、%）
±400kV	km			
**2. 换流容量**	**万 kW**	**3860**	**1370**	**181.75**
其中：±800kV	万 kW	2900	1050	176.19
±660kV	万 kW			
±500kV	万 kW	960	320	200.00
±400kV	万 kW			
**十、新增交流 110kV 及以上输电线路长度及变电设备容量**				
**1. 线路长度**	**km**	**59 799**	**59 604**	**0.33**
其中：1000kV	km	1206	1298	−7.09
750kV	km	1314	2218	−40.78
500kV	km	7272	7469	−2.64
330kV	km	1202	1598	−24.77
220kV	km	22 098	21 498	2.79
110kV（含 66kV）	km	26 708	25 523	4.64
**2. 变电设备容量**	**万 kVA**	**30 853**	**28 577**	**7.97**
其中：1000kV	万 kVA	1800	2100	−14.29
750kV	万 kVA	660	1180	−44.07
500kV	万 kVA	7555	5580	35.39
330kV	万 kVA	741	573	29.32
220kV	万 kVA	11 602	10 398	11.58
110kV（含 66kV）	万 kVA	8495	8746	−2.87
**十一、本年完成电力投资**	**亿元**	**7805**	**7728**	**1.00**
**1. 电源投资**	**亿元**	**3686**	**3872**	**−4.80**
水电	亿元	943	1223	−22.92
火电	亿元	1145	1016	12.67
核电	亿元	533	660	−19.31
风电	亿元	915	650	40.93
太阳能发电	亿元	150	323	−53.43
其他	亿元			
**2. 电网投资**	**亿元**	**4119**	**3856**	**6.82**
送变电	亿元	3993	3768	5.98
其中：直流	亿元	168	354	−52.68
交流	亿元	3825	3413	12.07
其他	亿元	126	89	42.52
**十二、单机 6000kW 及以上机组平均单机容量**				
水电：单机容量	万 kW/台	6.34	5.91	7.36
机组台数	台	3945	3998	−1.33
机组容量	万 kW	25 020	23 617	5.94
火电：单机容量	万 kW/台	12.53	11.75	6.59
机组台数	台	7162	7223	−0.84

?1994-2018 China Academic Journal Electronic Publishing House. All rights reserved. http://www.cnki.net

续表

基本数据项目	单位	2014年	2013年	比2013年增长（±、%）
机组容量	万kW	89 723	84 891	5.69
**十三、6000kW及以上电厂供热量**	**万GJ**	**318 362**	**324 128**	**−1.78**
**十四、6000kW及以上电厂发电标准煤耗**	**g/kWh**	**300**	**302**	**−2**
**十五、6000kW及以上电厂供电标准煤耗**	**g/kWh**	**319**	**321**	**−2**
**十六、6000kW及以上电厂厂用电率**	**%**	**4.83**	**5.05**	**−0.23**
水电	%	0.50	0.33	0.17
火电	%	5.84	6.01	−0.18
**十七、6000kW及以上电厂发电设备利用小时**	**h**	**4318**	**4521**	**−204**
水电	h	3669	3359	310
其中：抽水蓄能	h	609	513	97
火电	h	4739	5021	−282
核电	h	7787	7874	−87
风电	h	1900	2025	−124
太阳能发电	h	1235	1342	−107
**十八、6000kW及以上电厂燃料消耗**				
发电消耗标煤量	万t	117 737	122 127	−3.59
发电消耗原煤量	万t	175 777	185 782	−5.39
供热消耗标煤量	万t	12 310	12 834	−4.08
供热消耗原煤量	万t	18 631	19 168	−2.80
**十九、6000kW及以上火电厂热效率**				
电厂热效率	%	43.99	42.38	1.62
电厂供热效率	%	88.24	86.17	2.07
电厂能源转换总效率	%	48.18	45.42	2.76
**二十、供、售电量及线损**				
供电量	亿kWh	48 676	47 075	3.40
售电量	亿kWh	45 442	43 768	3.82
线损电量	亿kWh	3234	3306	−2.19
线路损失率	%	6.64	7.02	−0.38
**二十一、发用电设备比**				
发电装机容量∶用电设备容量		1∶3.71	1∶3.51	
**二十二、电力弹性系数**				
电力生产弹性系数		0.58	1.00	−0.42
电力消费弹性系数		0.56	0.98	−0.43

注 1. 风电、太阳能发电装机容量和发电量等指标均为并网口径。
2. 变电设备容量包含换流变容量，2014年国家电网公司调整±800kV和±660kV换流容量至转换对应电压等级。
3. 2014年起国家电网公司供售电统计中剔除了自发自用电量，相应按照新口径调整了2013年供售电量、线损数值。
4. 生物质发电2014年底装机容量1030万kW，2014年发电量474亿kWh。

?1994-2018 China Academic Journal Electronic Publishing House. All rights reserved. http://www.cnki.net

电力统计基本数据一览表

基本数据项目	单位	2015 年	2014 年	同比增长（%）
一、发电量	亿 kWh	57 399	56 801	1.05
水电	亿 kWh	11 127	10 601	4.96
其中：抽水蓄能	亿 kWh	158	132	19.44
火电	亿 kWh	42 307	43 030	-1.68
其中：燃煤	亿 kWh	38 977	40 266	-3.20
燃气	亿 kWh	1669	1333	25.23
燃油	亿 kWh	42	44	-6.10
核电	亿 kWh	1714	1332	28.65
风电	亿 kWh	1856	1598	16.17
太阳能发电	亿 kWh	395	235	67.92
其他	亿 kWh	1	5	-72.40
6000kW 及以上火电厂发电量	亿 kWh	42 181	42 925	-1.73
燃煤	亿 kWh	38 948	40 205	-3.13
其中：煤矸石发电	亿 kWh	2282	1587	43.83
燃气	亿 kWh	1654	1322	25.12
其中：常规燃气	亿 kWh	1586	1288	23.10
煤层气发电	亿 kWh	47	21	118.91
燃油	亿 kWh	38	44	-13.56
其他	亿 kWh	1541	1354	13.83
其中：余温、余气、余压发电	亿 kWh	1002	892	12.26
垃圾焚烧发电	亿 kWh	252	245	2.86
秸秆、蔗渣、林木质发电	亿 kWh	287	216	32.72
二、全社会用电量	亿 kWh	56 933	56 393	0.96
1. 全行业用电合计	亿 kWh	49 648	49 455	0.39
第一产业	亿 kWh	1040	1014	2.55
第二产业	亿 kWh	41 442	41 770	-0.79
其中：工业	亿 kWh	40 743	41 048	-0.74
其中：轻工业	亿 kWh	6772	6677	1.41
重工业	亿 kWh	33 971	34 371	-1.16
第三产业	亿 kWh	7166	6671	7.42
2. 城乡居民生活用电合计	亿 kWh	7285	6938	5.01
其中：城镇居民	亿 kWh	4104	3934	4.31
乡村居民	亿 kWh	3181	3004	5.92
三、发电装机容量	万 kW	152 527	137 887	10.62
水电	万 kW	31 954	30 486	4.82

(C)1994-2024 China Academic Journal Electronic Publishing House. All rights reserved. http://www.cnki.net

续表

基本数据项目	单位	2015 年	2014 年	同比增长（%）
其中：抽水蓄能	万 kW	2303	2211	4.16
火电	万 kW	100 554	93 232	7.85
其中：燃煤	万 kW	90 009	84 102	7.02
燃气	万 kW	6603	5697	15.91
燃油	万 kW	434	512	−15.12
核电	万 kW	2717	2008	35.31
风电	万 kW	13 075	9657	35.40
太阳能发电	万 kW	4218	2486	69.66
其他	万 kW	9	19	−54.69
6000kW 及以上火电厂装机容量	万 kW	100 036	92 731	7.88
燃煤	万 kW	89 913	83 976	7.07
其中：煤矸石发电	万 kW	4473	3349	33.55
燃气	万 kW	6562	5666	15.81
其中：常规燃气	万 kW	6317	5526	14.32
煤层气发电	万 kW	153	91	69.25
燃油	万 kW	219	270	−18.75
其他	万 kW	3341	2819	18.52
其中：余温、余气、余发电	万 kW	2200	1838	19.67
垃圾焚烧发电	万 kW	477	469	1.80
秸秆、蔗渣、林木质发电	万 kW	664	512	29.69
四、35kV 及以上输电线路回路长度	km	1 696 849	1 628 472	4.20
1. 交流	km	1 671 420	1 603 488	4.24
其中：1000kV	km	3114	3111	0.10
750kV	km	15 665	13 881	12.85
500kV	km	157 974	152 107	3.86
330kV	km	26 811	25 146	6.62
220kV	km	380 121	358 377	6.07
110kV	km	591 637	566 571	4.42
35kV	km	496 098	484 296	2.44
2. 直流	km	25 429	24 984	1.78
其中：±800kV	km	10 580	10 132	4.42
±660kV	km	1336	1336	0.00
±500kV	km	11 872	11 875	0.00
±400kV	km	1640	1640	0.00
五、35kV 及以上变电设备容量	万 kVA	569 928	526 685	8.21
1. 交流	万 kVA	551 546	509 134	8.33
其中：1000kV	万 kVA	5700	5700	0.00
750kV	万 kVA	10 850	8090	34.11

(C)1994-2024 China Academic Journal Electronic Publishing House. All rights reserved. http://www.cnki.net

续表

基本数据项目	单位	2015 年	2014 年	同比增长（%）
500kV	万 kVA	107 082	100 011	7.07
330kV	万 kVA	11 679	10 493	11.31
220kV	万 kVA	182 893	167 342	9.29
110kV	万 kVA	185 819	171 588	8.29
35kV	万 kVA	47 521	45 909	3.51
**2. 直流**	**万 kVA**	**18 383**	**17 551**	**4.74**
其中：±800kV	万 kVA	3180	3180	0.00
±660kV	万 kVA			
±500kV	万 kVA	15 203	14 230	6.84
±400kV	万 kVA		141	
**六、新增发电装机容量**	**万 kW**	**13 184**	**10 443**	**26.25**
水电	万 kW	1375	2180	−36.92
其中：抽水蓄能	万 kW	92	60	53.33
火电	万 kW	6678	4791	39.40
其中：燃煤	万 kW	5402	3498	54.42
燃气	万 kW	696	946	−26.49
其中：常规燃气	万 kW	692	938	−26.25
煤层气发电		4	8	−54.49
燃油	万 kW			
其他	万 kW	581	346	67.68
其中：余温、余气、余压	万 kW	529	254	107.96
垃圾焚烧发电	万 kW	4	21	−79.57
秸秆、蔗渣、林木质发电	万 kW	47	71	−33.35
核电	万 kW	612	547	11.88
风电	万 kW	3139	2101	49.42
太阳能发电	万 kW	1380	825	67.30
其他	万 kW			
**七、火电机组退役和关停容量**	**万 kW**	**1091**	**909**	**20.04**
**八、年底主要发电企业电源项目在建规模**	**万 kW**	**18 175**	**14 500**	**25.35**
水电	万 kW	5748	4328	32.82
火电	万 kW	7824	5524	41.64
核电	万 kW	3054	2863	6.68
风电	万 kW	1317	1676	−21.38
**九、新增直流输电线路长度及换流容量**				
**1. 线路长度**	**km**		**2876**	**−100.00**
其中：±800kV	km		1653	−100.00
±660kV	km			
±500kV	km		1223	−100.00

(C)1994-2024 China Academic Journal Electronic Publishing House. All rights reserved. http://www.cnki.net

续表

基本数据项目	单位	2015年	2014年	同比增长（%）
±400kV	km			
**2. 换流容量**	**万kW**	**250**	**3860**	**-93.52**
其中：±800kV	万kW	250	2900	-91.38
±660kV	万kW			
±500kV	万kW		960	-100.00
±400kV	万kW			
**十、新增交流110kV及以上输电线路长度及变电设备容量**				
**1. 线路长度**	**km**	**57 110**	**59 799**	**-4.50**
其中：1000kV	km	5	1206	-99.59
750kV	km	1639	1314	24.78
500kV	km	7389	7272	1.61
330kV	km	2162	1202	79.87
220kV	km	22 054	22 098	-0.20
110kV（含66kV）	km	23 862	26 708	-10.66
**2. 变电设备容量**	**万kVA**	**29 432**	**30 853**	**-4.61**
其中：1000kV	万kVA		1800	-100.00
750kV	万kVA	3570	660	440.91
500kV	万kVA	8880	7555	17.54
330kV	万kVA	642	741	-13.36
220kV	万kVA	8810	11 602	-24.06
110kV（含66kV）	万kVA	7530	8495	-11.36
**十一、本年完成电力投资**	**亿元**	**8576**	**7805**	**9.87**
**1. 电源投资**	**亿元**	**3936**	**3686**	**6.78**
水电	亿元	789	943	-16.28
火电	亿元	1163	1145	1.61
核电	亿元	565	533	6.07
风电	亿元	1200	915	31.10
太阳能发电	亿元	218	150	45.21
其他	亿元			
**2. 电网投资**	**亿元**	**4640**	**4119**	**12.64**
送变电	亿元	4514	3993	13.04
其中：直流	亿元	216	168	28.90
交流	亿元	4298	3825	12.35
其他	亿元	126	126	-0.27
**十二、单机6000kW及以上机组平均单机容量**				
水电：单机容量	万kW/台	6.40	6.34	0.06
机组台数	台	4119	3945	4.41
机组容量	万kW	26 361	25 020	5.36

(C)1994-2024 China Academic Journal Electronic Publishing House. All rights reserved. http://www.cnki.net

续表

基本数据项目	单位	2015年	2014年	同比增长（%）
火电：单机容量	万kW/台	12.89	12.53	0.37
机组台数	台	7526	7162	5.08
机组容量	万kW	97 033	89 723	8.15
十三、6000kW及以上电厂供热量	万GJ	368 012	323 687	13.69
十四、6000kW及以上电厂发电标准煤耗	g/kWh	297	300	-3
十五、6000kW及以上电厂供电标准煤耗	g/kWh	315	319	-4
十六、6000kW及以上电厂厂用电率	%	5.09	4.85	0.24
水电	%	0.32	0.50	-0.19
火电	%	6.04	5.85	0.19
十七、6000kW及以上电厂发电设备利用小时	h	3988	4348	-360
水电	h	3590	3669	-79
其中：抽水蓄能	h	702	609	93
火电	h	4364	4778	-414
核电	h	7403	7787	-384
风电	h	1724	1900	-176
太阳能发电	h	1225	1235	-11
十八、6000kW及以上电厂燃料消耗				
发电消耗标煤量	万t	115 015	120 346	-4.43
发电消耗原煤量	万t	167 310	179 498	-6.79
供热消耗标煤量	万t	14 405	12 509	15.15
供热消耗原煤量	万t	21 104	18 950	11.37
十九、6000kW及以上火电厂热效率				
电厂热效率	%	45.05	43.81	1.24
电厂供热效率	%	87.17	88.29	-1.12
电厂能源转换总效率	%	48.54	46.87	1.66
二十、供、售电量及线损				
供电量	亿kWh	48 572	48 676	-0.21
售电量	亿kWh	45 347	45 442	-0.21
线损电量	亿kWh	3226	3234	-0.26
线路损失率	%	6.64	6.64	0.00
二十一、发用电设备比				
发电装机容量：用电设备容量		1:3.77	1:3.71	
二十二、电力弹性系数				
电力生产弹性系数		0.15	0.58	-0.43
电力消费弹性系数		0.14	0.56	-0.42

注 1. 风电、太阳能发电装机容量和发电量等指标均为并网口径。
2. 变电设备容量包含换流变压器容量，2014年国家电网公司调整直流换流容量至转换对应电压等级。
3. 生物质发电2015年底装机容量1280万kW，2015年发电量578亿kWh。
4. 自2015年起，山东魏桥集团正式纳入电力行业统计口径，对2014年同期数据进行了相应调整。

(C)1994-2024 China Academic Journal Electronic Publishing House. All rights reserved. http://www.cnki.net

2017年全国电力统计基本数据一览表

	单　　位	2017年	2016年	比上年增长（±、%）
**一 、发　电　量**	**亿千瓦时**	**64171**	**60228**	**6.55**
水　　电	亿千瓦时	11931	11748	1.55
其中：抽水蓄能	亿千瓦时	328	308	6.41
火　　电	亿千瓦时	45558	43273	5.28
其中：燃煤	亿千瓦时	41498	39457	5.17
燃气	亿千瓦时	2028	1883	7.69
核　　电	亿千瓦时	2481	2132	16.39
风　　电	亿千瓦时	3034	2409	25.97
太阳能发电	亿千瓦时	1166	665	75.29
**二、全社会用电量**	**亿千瓦时**	**63625**	**59710**	**6.56**
**A、全行业用电合计**	**亿千瓦时**	**54923**	**51633**	**6.37**
第一产业	亿千瓦时	1175	1093	7.49
第二产业	亿千瓦时	44922	42567	5.53
其中:工　业	亿千瓦时	44133	41840	5.48
其中：轻工业	亿千瓦时	7526	7046	6.82
重工业	亿千瓦时	36607	34794	5.21
第三产业	亿千瓦时	8825	7973	10.69
**B、城乡居民生活用电合计**	**亿千瓦时**	**8703**	**8077**	**7.74**
城镇居民	亿千瓦时	4962	4571	8.54
乡村居民	亿千瓦时	3741	3506	6.71
**三、发电装机容量**	**万千瓦**	**177708**	**165051**	**7.67**
水　　电	万千瓦	34359	33207	3.47
其中：抽水蓄能	万千瓦	2869	2669	7.49
火　　电	万千瓦	110495	106094	4.15
其中：燃煤	万千瓦	98130	94624	3.70
燃气	万千瓦	7570	7011	7.98
核　　电	万千瓦	3582	3364	6.47
风　　电	万千瓦	16325	14747	10.70
太阳能发电	万千瓦	12942	7631	69.59
**四、35千伏及以上输电线路回路长度**	**千米**	**1825611**	**1756141**	**3.96**
**1、交流**	**千米**	**1788212**	**1727333**	**3.52**
其中: 1000千伏	千米	10073	7245	39.04
750千伏	千米	18830	17968	4.80
500千伏	千米	173772	165875	4.76
330千伏	千米	30183	28366	6.41
220千伏	千米	415311	397050	4.60
110千伏	千米	631361	611431	3.26
35千伏	千米	508682	499400	1.86
**2、直流**	**千米**	**37399**	**28808**	**29.82**
其中：±800千伏	千米	20874	12295	69.77
±660千伏	千米	1334	1334	
±500千伏	千米	13552	13539	0.09

	单　　位	2017年	2016年	比上年增长（±、%）
±400千伏	千米	1640	1640	
**五、35千伏及以上变电设备容量**	**万千伏安**	**663108**	**629982**	**5.3**
**1、交流**	**万千伏安**	**630065**	**606236**	**3.9**
其中: 1000千伏	万千伏安	13800	9900	39.4
750千伏	万千伏安	14540	13570	7.1
500千伏	万千伏安	125508	117128	7.2
330千伏	万千伏安	12613	11320	11.4
220千伏	万千伏安	203159	193599	4.9
110千伏	万千伏安	209801	207426	1.1
35千伏	万千伏安	50643	53294	-5.0
**2、直流**	**万千伏安**	**33043**	**23746**	**39.2**
其中：±800千伏	万千伏安	10696	4882	119.1
±660千伏	万千伏安	484	484	
±500千伏	万千伏安	18831	17567	7.2
±400千伏	万千伏安			
**六、新增发电装机容量**	**万千瓦**	**13118**	**12143**	**8.03**
水　　电	万千瓦	1287	1179	9.20
其中：抽水蓄能	万千瓦	200	366	-45.36
火　　电	万千瓦	4453	5048	-11.78
其中：燃煤	万千瓦	3504	3866	-9.35
燃气	万千瓦	571	214	166.38
核　　电	万千瓦	218	720	-69.81
风　　电	万千瓦	1819	2024	-10.13
太阳能发电	万千瓦	5341	3171	68.42
**七、火电机组退役和关停容量**	**万千瓦**	**929**	**571**	**62.64**
**八、年底主要发电企业电源项目在建规模**	**万千瓦**	**20804**	**20757**	**0.23**
水　　电	万千瓦	7887	7433	6.11
火　　电	万千瓦	8637	9129	-5.39
核　　电	万千瓦	2289	2447	-6.44
风　　电	万千瓦	1909	1597	19.55
**九、新增直流输电线路长度及换流容量**				
**1、线路长度**	**千米**	**8339**	**3391**	**145.91**
其中: ±800千伏	千米	8339	1720	384.81
±660千伏	千米			
±500千伏	千米	0	1671	-100.00
±400千伏	千米			
**2、换流容量**	**万千瓦**	**7900**	**3240**	**143.83**
其中: ±800千伏	万千瓦	7700	1600	381.25
±660千伏	万千瓦			
±500千伏	万千瓦	200	1640	-87.80
±400千伏	万千瓦			
**十、新增交流110千伏及以上输电线路长度及变电设备容量**				
**1、线路长度**	**千米**	**58084**	**56679**	**2.48**
其中: 1000千伏	千米	2846	4252	-33.07
750千伏	千米	899	1813	-50.42

	单　　位	2017年	2016年	比上年增长（±、%）
500千伏	千米	7999	6931	15.42
330千伏	千米	2521	1525	65.26
220千伏	千米	18810	17088	10.08
110千伏（含66千伏）	千米	25010	25070	-0.24
**2、变电设备容量**	**万千伏安**	**32595**	**34585**	**-5.75**
其中：1000千伏	万千伏安	3000	5100	-41.18
750千伏	万千伏安	1740	1860	-6.45
500千伏	万千伏安	8275	7715	7.26
330千伏	万千伏安	783	480	63.13
220千伏	万千伏安	10433	9239	12.92
110千伏（含66千伏）	万千伏安	8364	10191	-17.93
**十一、主要电力企业本年完成电力投资**	**亿元**	**8239**	**8840**	**-6.80**
**1、电源投资**	**亿元**	**2900**	**3408**	**-14.92**
水　　电	亿元	622	617	0.73
火　　电	亿元	858	1119	-23.37
核　　电	亿元	454	504	-9.89
风　　电	亿元	681	927	-26.50
太阳能发电	亿元	285	241	18.24
**2、电网投资**	**亿元**	**5339**	**5431**	**-1.70**
送变电	亿元	5135	5282	-2.77
其中：直流	亿元	859	495	73.61
交流	亿元	4276	4787	-10.67
其　他	亿元	204	150	35.82
**十二、单机6000千瓦及以上机组平均单机容量**				
水电	万千瓦/台	6.07	6.42	-0.34
火电	万千瓦/台	13.15	13.19	-0.05
**十三、6000千瓦及以上电厂供电标准煤耗**	**克/千瓦时**	**309**	**312**	**-3**
**十四、6000千瓦及以上电厂厂用电率**	**%**	**4.80**	**4.77**	**0.03**
水　　电	%	0.27	0.29	-0.02
火　　电	%	6.04	6.01	0.03
**十五、6000千瓦及以上电厂发电设备利用小时**	**小时**	**3790**	**3797**	**-7**
水　　电	小时	3597	3619	-22
其中：抽水蓄能	小时	1176	1266	-90
火　　电	小时	4219	4186	33
核　　电	小时	7089	7060	28
风　　电	小时	1949	1745	204
太阳能发电	小时	1205	1129	76
**十六、供、售电量及线损**				
供电量	亿千瓦时	54357	50742	7.12
售电量	亿千瓦时	50835	47451	7.13
线损电量	亿千瓦时	3522	3291	7.00
线路损失率	%	6.48	6.49	-0.01

电力统计基本数据一览

项目	单　位	2018 年	2017 年	比上年增长（%）
**一、发电量**	**亿 kWh**	**69 947**	**64 529**	**8.40**
水电	亿 kWh	12 321	11 947	3.13
其中：抽水蓄能	亿 kWh	329	328	0.44
火电	亿 kWh	49 249	45 877	7.35
其中：燃煤	亿 kWh	44 829	41 782	7.29
燃气	亿 kWh	2155	2032	6.04
燃油	亿 kWh	15	27	-42.53
生物质发电	亿 kWh	936	813	15.23
核电	亿 kWh	2950	2481	18.87
风电	亿 kWh	3658	3046	20.09
太阳能发电	亿 kWh	1769	1178	50.24
其他	亿 kWh	1	1	-4.15
**6000kW 及以上火电厂发电量**	**亿 kWh**	**49 167**	**45 762**	**7.44**
燃煤	亿 kWh	44 821	41 772	7.30
其中：煤矸石发电	亿 kWh	1286	1423	-9.58
燃气	亿 kWh	2134	2016	5.86
其中：常规燃气	亿 kWh	2053	1957	4.93
煤层气发电	亿 kWh	70	52	35.94
燃油	亿 kWh	15	27	-42.45
其他	亿 kWh	2143	1920	11.62
其中：余温、余气、余压发电	亿 kWh	1230	1129	8.96
垃圾焚烧发电	亿 kWh	481	376	28.08
秸秆、蔗渣、林木质发电	亿 kWh	427	413	3.40
**二、全社会用电量**	**亿 kWh**	**69 002**	**63 636**	**8.43**
**1. 全行业用电合计**	**亿 kWh**	**59 310**	**54 849**	**8.13**
第一产业	亿 kWh	746	684	8.99
第二产业	亿 kWh	47 733	44 571	7.09
其中：工业	亿 kWh	46 954	43 874	7.02
第三产业	亿 kWh	10 831	9593	12.90
**2. 城乡居民生活用电合计**	**亿 kWh**	**9692**	**8788**	**10.29**
城镇居民	亿 kWh	5531	5010	10.40
乡村居民	亿 kWh	4162	3778	10.15

(C)1994-2024 China Academic Journal Electronic Publishing House. All rights reserved. http://www.cnki.net

续表

项目	单位	2018 年	2017 年	比上年增长（%）
三、发电装机容量	万 kW	**190 012**	**178 451**	**6.48**
水电	万 kW	35 259	34 411	2.46
其中：抽水蓄能	万 kW	2999	2869	4.52
火电	万 kW	114 408	111 009	3.06
其中：燃煤	万 kW	100 835	98 562	2.31
燃气	万 kW	8375	7580	10.49
燃油	万 kW	173	197	-11.94
生物质发电	万 kW	1947	1651	17.93
核电	万 kW	4466	3582	24.68
风电	万 kW	18 427	16 400	12.35
太阳能发电	万 kW	17 433	13 042	33.66
其他	万 kW	20	7	200.35
**6000kW 及以上火电厂装机容量**	**万 kW**	**114 100**	**110 506**	**3.25**
燃煤	万 kW	100 794	98 517	2.31
其中：煤矸石发电	万 kW	3240	3518	-7.90
燃气	万 kW	8313	7532	10.37
其中：常规燃气发电	万 kW	8139	7400	9.98
煤层气发电	万 kW	151	113	33.32
燃油	万 kW	170	193	-12.11
其他	万 kW	4755	4199	13.24
其中：余温、余气、余发电	万 kW	2854	2626	8.68
垃圾焚烧发电	万 kW	889	722	23.08
秸秆、蔗渣、林木质发电	万 kW	969	850	13.97
**四、35kV 及以上输电线路回路长度**	**km**	**1 892 018**	**1 825 611**	**3.64**
**1. 交流**	**km**	**1 850 631**	**1 788 212**	**3.49**
其中：1000kV	km	11 005	10 073	9.25
750kV	km	20 543	18 830	9.10
500kV	km	187 158	173 772	7.70
330kV	km	30 477	30 183	0.97
220kV	km	434 493	415 311	4.62
110kV	km	652 891	631 361	3.41
35kV	km	514 066	508 682	1.06
**2. 直流**	**km**	**41 995**	**37 399**	**12.29**
其中：±1100kV	km	608		
±800kV	km	21 723	20 874	4.07
±660kV	km	2091	1334	56.75
±500kV	km	15 428	13 552	13.84
±400kV	km	1640	1640	0.00

(C)1994-2024 China Academic Journal Electronic Publishing House. All rights reserved. http://www.cnki.net

续表

项目	单 位	2018 年	2017 年	比上年增长（%）
五、35kV 及以上变电设备容量	万 kVA	699 219	662 928	5.47
**1. 交流**	**万 kVA**	**666 622**	**630 730**	**5.69**
其中：1000kV	万 kVA	14 700	13 800	6.52
750kV	万 kVA	17 030	14 540	17.13
500kV	万 kVA	136 494	125 508	8.75
330kV	万 kVA	13 125	13 029	0.74
220kV	万 kVA	213 127	203 352	4.81
110kV	万 kVA	219 379	209 847	4.54
35kV	万 kVA	52 767	50 654	4.17
**2. 直流**	**万 kVA**	**33 196**	**32 198**	**3.10**
其中：±1100kV	万 kVA	600		
±800kV	万 kVA	17 361	17 841	-2.69
±660kV	万 kVA	947	947	0.00
±500kV	万 kVA	13 353	13 410	-0.43
±400kV	万 kVA	141		
**六、新增发电装机容量**	**万 kW**	**12 785**	**13 019**	**-1.80**
水电	万 kW	859	1287	-33.27
其中：抽水蓄能	万 kW	130	200	-35.00
火电	万 kW	4380	4453	-1.65
其中：燃煤	万 kW	3056	3504	-12.79
燃气	万 kW	884	571	54.87
其中：常规燃气	万 kW	881	571	54.42
煤层气发电		2		
燃油	万 kW			
其他	万 kW	440	379	16.26
其中：余温、余气、余压	万 kW	198	175	13.13
垃圾焚烧发电	万 kW	148	123	20.53
秸秆、蔗渣、林木质发电	万 kW	94	81	16.51
核电	万 kW	884	218	306.44
风电	万 kW	2127	1720	23.64
太阳能发电	万 kW	4525	5341	-15.26
其他	万 kW	10		
**七、火电机组退役和关停容量**	**万 kW**	**1197**	**929**	**28.79**
**八、年底主要发电企业电源项目在建规模**	**万 kW**	**17 890**	**20 804**	**-14.01**
水电	万 kW	7940	7887	0.68
火电	万 kW	6936	8637	-19.70
核电	万 kW	1345	2289	-41.23
风电	万 kW	1564	1909	-18.07

(C)1994-2024 China Academic Journal Electronic Publishing House. All rights reserved. http://www.cnki.net

续表

项目	单　位	2018 年	2017 年	比上年增长（%）
九、新增直流输电线路长度及换流容量				
**1. 线路长度**	**km**	**3325**	**8339**	**-60.13**
其中：±1100kV	km	3325		
±800kV	km		8339	-100.00
±660kV	km			
±500kV	km			
±400kV	km			
**2. 换流容量**	**万 kW**	**3200**	**7900**	**-59.49**
其中：±1100kV	万 kW	1200		
±800kV	万 kW	2000	7700	-74.03
±660kV	万 kW			
±500kV	万 kW		200	-100.00
±400kV	万 kW			
**十、新增交流 110kV 及以上输电线路长度及变电设备容量**				
**1. 线路长度**	**km**	**56 973**	**58 084**	**-1.91**
其中：1000kV	km	129	2846	-95.47
750kV	km	1573	899	74.97
500kV	km	14 540	7999	81.77
330kV	km	828	2521	-67.16
220kV	km	20 697	18 810	10.03
110kV（含 66kV）	km	19 206	25 010	-23.21
**2. 变电设备容量**	**万 kVA**	**31 024**	**32 595**	**-4.82**
其中：1000kV	万 kVA	900	3000	-70.00
750kV	万 kVA	1140	1740	-34.48
500kV	万 kVA	11 160	8275	34.86
330kV	万 kVA	612	783	-21.84
220kV	万 kVA	8402	10 433	-19.47
110kV（含 66kV）	万 kVA	8810	8364	5.33
**十一、本年完成电力投资**	**亿元**	**8161**	**8239**	**-0.94**
**1. 电源投资**	**亿元**	**2787**	**2900**	**-3.89**
水电	亿元	700	622	12.65
火电	亿元	786	858	-8.31
核电	亿元	447	454	-1.55
风电	亿元	646	681	-5.12
太阳能发电	亿元	207	285	-27.38
其他	亿元			
**2. 电网投资**	**亿元**	**5374**	**5339**	**0.65**
送变电	亿元	5133	5135	-0.04
其中：直流	亿元	520	859	-39.41
交流	亿元	4613	4276	7.87

(C)1994-2024 China Academic Journal Electronic Publishing House. All rights reserved.　http://www.cnki.net

续表

项目	单 位	2018 年	2017 年	比上年增长（%）
其他	亿元	241	204	18.10
**十二、单机 6000kW 及以上机组平均单机容量**				
水电：单机容量	万 kW/台	6.10	6.07	0.02
机组台数	台	4894	4816	1.62
机组容量	万 kW	29 830	29 252	1.98
火电：单机容量	万 kW/台	13.38	13.15	0.23
机组台数	台	8070	7796	3.51
机组容量	万 kW	107 969	102 501	5.33
**十三、6000kW 及以上电厂供热量**	**万 GJ**	**480 625**	**421 084**	**14.14**
**十四、6000kW 及以上电厂发电标准煤耗**	**g/kWh**	**289.9**	**291.3**	**-1.3**
**十五、6000kW 及以上电厂供电标准煤耗**	**g/kWh**	**307.6**	**309.4**	**-1.8**
**十六、6000kW 及以上电厂厂用电率**	**%**	**4.69**	**4.80**	**-0.11**
水电	%	0.25	0.27	-0.02
火电	%	5.95	6.04	-0.09
**十七、6000kW 及以上电厂发电设备利用小时**	h	**3880**	**3790**	**90**
水电	h	3607	3597	10
其中：抽水蓄能	h	1102	1176	-74
火电	h	4378	4219	159
核电	h	7543	7089	454
风电	h	2103	1949	155
太阳能发电	h	1230	1205	25
**十八、6000kW 及以上电厂燃料消耗**				
发电消耗标煤量	万 t	130 805	121 811	7.38
发电消耗原煤量	万 t	195 719	177 592	10.21
供热消耗标煤量	万 t	18 104	16 519	9.60
供热消耗原煤量	万 t	27 523	24 248	13.51
**十九、供、售电量及线损**				
供电量	亿 kWh	59 508	54 357	9.48
售电量	亿 kWh	55 777	50 835	9.72
线损电量	亿 kWh	3731	3522	5.93
线路损失率	%	6.27	6.48	-0.21
**二十、发用电设备比**				
发电装机容量：用电设备容量		1:3.96	1:4.05	
**二十一、电力弹性系数**				
电力生产弹性系数		1.27	0.95	34.07
电力消费弹性系数		1.28	0.95	34.41

注 1. 电源投资完成额口径为全国主要发电企业。

2. 从 2018 年 5 月开始，三次产业划分按照《国家统计局关于修订〈三次产业划分规定（2012）〉的通知》（国统设管函〔2018〕74 号）调整，为保证数据可比，同期数据根据新标准重新进行了分类。

(C)1994-2024 China Academic Journal Electronic Publishing House. All rights reserved. http://www.cnki.net

电力统计基本数据一览表

项　　目	单位	2020 年	2019 年	比上年增长（±、%）
**一、发电量**	**亿 kWh**	**76264**	**73269**	**4.09**
水　　电	亿 kWh	13553	13021	4.09
其中：抽水蓄能	亿 kWh	335	319	4.99
火　　电	亿 kWh	51770	50465	2.59
其中：燃煤	亿 kWh	46296	45538	1.66
燃气	亿 kWh	2525	2325	8.61
燃油	亿 kWh	12	13	-5.08
其中：生物质发电	亿 kWh	1355	1126	20.35
核　　电	亿 kWh	3662	3487	5.03
风　　电	亿 kWh	4665	4053	15.08
太阳能发电	亿 kWh	2611	2240	16.56
其　　他	亿 kWh	3	2	12.40
**非化石能源发电量**	**亿 kWh**	**25850**	**23930**	**8.02**
**二、全社会用电量**	**亿 kWh**	**75214**	**72852**	**3.24**
**A. 全行业用电合计**	**亿 kWh**	**64268**	**62607**	**2.65**
第一产业	亿 kWh	859	779	10.15
第二产业	亿 kWh	51318	49963	2.71
其中：工业	亿 kWh	50398	49073	2.70
第三产业	亿 kWh	12091	11865	1.91
**B. 城乡居民生活用电合计**	**亿 kWh**	**10946**	**10245**	**6.84**
城镇居民	亿 kWh	6157	5835	5.52
乡村居民	亿 kWh	4789	4410	8.60
**三、发电装机容量**	**万 kW**	**220204**	**201006**	**9.55**
水　　电	万 kW	37028	35804	3.42
其中：抽水蓄能	万 kW	3149	3029	3.96
火　　电	万 kW	124624	118957	4.76
其中：燃煤	万 kW	107912	104063	3.70
燃气	万 kW	9972	9024	10.51
燃油	万 kW	147	175	-15.99
其中：生物质发电	万 kW	2987	2361	26.51
核　　电	万 kW	4989	4874	2.36

(C)1994-2024 China Academic Journal Electronic Publishing House. All rights reserved.　http://www.cnki.net

续表

项　　目	单位	2020 年	2019 年	比上年增长（±、%）
风　　电	万 kW	28165	20915	34.66
太阳能发电	万 kW	25356	20429	24.12
其　　他	万 kW	41	26	58.02
**非化石能源发电装机容量**	**万 kW**	**98567**	**84410**	**16.77**
**四、35kV 及以上输电线路回路长度**	**km**	**2156170**	**1975312**	**9.16**
**1. 交流**	**km**	**2109846**	**1932947**	**9.15**
其中：1000kV	km	13361	10872	22.89
750kV	km	25046	23256	7.70
500kV	km	203058	195636	3.79
330kV	km	36597	32314	13.25
220kV	km	488543	454585	7.47
110kV	km	752563	684406	9.96
35kV	km	590678	531880	11.05
**2. 直流**	**km**	**46324**	**42364**	**9.35**
其中：±1100kV	km	3295	3295	
±800kV	km	24980	21907	14.03
±660kV	km	1334	1334	
±500kV	km	14783	13733	7.64
±400kV	km	1639	1639	
**五、35kV 及以上变电设备容量**	**万 kVA**	**812893**	**747833**	**8.70**
**1. 交流**	**万 kVA**	**766565**	**708718**	**8.16**
其中：1000kV	万 kVA	18000	15300	17.65
750kV	万 kVA	19785	18515	6.86
500kV	万 kVA	155163	145905	6.35
330kV	万 kVA	15771	14062	12.16
220kV	万 kVA	243736	226101	7.80
110kV	万 kVA	250286	235077	6.47
35kV	万 kVA	63823	53757	18.72
**2. 直流**	**万 kVA**	**46328**	**37706**	**22.87**
其中：±1100kV	万 kVA	2867	2867	
±800kV	万 kVA	27690	22317	24.07
±660kV	万 kVA	947	947	
±500kV	万 kVA	12738	10945	16.38
±400kV	万 kVA	1245	1245	

(C)1994-2024 China Academic Journal Electronic Publishing House. All rights reserved.　http://www.cnki.net

续表

项　　目	单位	2020 年	2019 年	比上年增长（±、%）
**六、新增发电装机容量**	**万 kW**	**19144**	**10500**	**82.31**
水　　电	万 kW	1313	445	195.22
其中：抽水蓄能	万 kW	120	30	300.00
火　　电	万 kW	5660	4423	27.95
其中：燃煤	万 kW	4030	3236	24.52
燃气	万 kW	824	630	30.72
其中：常规燃气	万 kW	811	629	28.89
煤层气发电	万 kW	12	0	
燃油	万 kW			
其他	万 kW	805	557	44.73
其中：余温、余气、余压	万 kW	283	166	70.89
垃圾焚烧发电	万 kW	300	273	9.91
秸秆、蔗渣、林木质发电	万 kW	222	118	88.76
核　　电	万 kW	112	409	-72.64
风　　电	万 kW	7211	2572	180.40
太阳能发电	万 kW	4820	2652	81.76
其　　他	万 kW	28		
**七、火电机组退役和关停容量**	**万 kW**	**1469**	**1024**	**43.44**
**八、年底主要发电企业电源项目在建规模**	**万 kW**	**16137**	**18192**	**-11.29**
水　　电	万 kW	8186	8462	-3.27
火　　电	万 kW	3883	5409	-28.22
核　　电	万 kW	1547	1420	9.00
风　　电	万 kW	1996	2736	-27.04
**九、新增直流输电线路长度及换流容量**				
**1. 线路长度**	**km**	**4444**		
其中：±1100kV	km			
±800kV	km	3389		
±660kV	km			
±500kV	km	1055		
±400kV	km			
**2. 换流容量**	**万 kW**	**5200**	**2200**	**136.36**
其中：±1100kV	万 kW		1200	
±800kV	万 kW	4000		
±660kV	万 kW			

(C)1994-2024 China Academic Journal Electronic Publishing House. All rights reserved.　http://www.cnki.net

续表

项　　目	单位	2020年	2019年	比上年增长（±、%）
±500kV	万kW	1200		
±400kV	万kW		1000	
十、新增交流110kV及以上输电线路长度及变电设备容量				
**1. 线路长度**	**km**	**57237**	**57935**	**-1.20**
其中：1000kV	km	1736	2100	-17.35
750kV	km	1090	4406	-75.26
500kV	km	7424	5595	32.70
330kV	km	1566	3989	-60.73
220kV	km	18768	19822	-5.32
110kV（含66kV）	km	26653	22023	21.02
**2. 变电设备容量**	**万kVA**	**31292**	**31915**	**-1.95**
其中：1000kV	万kVA	1800	1500	20.00
750kV	万kVA	1860	3245	-42.68
500kV	万kVA	8255	8645	-4.51
330kV	万kVA	1098	1263	-13.06
220kV	万kVA	9275	9161	1.24
110kV（含66kV）	万kVA	9004	8100	11.15
**十一、本年完成电力投资**	**亿元**	**10189**	**9097**	**12.00**
**1. 电源投资**	**亿元**	**5292**	**4085**	**29.55**
水　　电	亿元	1067	905	17.90
火　　电	亿元	568	780	-27.26
核　　电	亿元	379	463	-17.95
风　　电	亿元	2653	1552	70.96
太阳能发电	亿元	625	385	62.18
其　　他	亿元			
**2. 电网投资**	**亿元**	**4896**	**5012**	**-2.30**
输变电	亿元	4721	4779	-1.23
其中：直流	亿元	532	249	113.36
交流	亿元	4188	4530	-7.54
其　　他	亿元	176	232	-24.29
十二、单机6000kW及以上机组平均单机容量				
水电：单机容量	万kW/台	6.23	6.04	0.19
机组台数	台	5158	5099	1.16
机组容量	万kW	32137	30788	4.38

(C)1994-2024 China Academic Journal Electronic Publishing House. All rights reserved.　http://www.cnki.net

续表

项　　目	单位	2020 年	2019 年	比上年增长（±、%）
火电：单机容量	万 kW/台	13.55	13.37	0.18
机组台数	台	8776	8430	4.10
机组容量	万 kW	118890	112722	5.47
**十三、6000kW 及以上电厂供热量**	**万 GJ**	**519422**	**492492**	**5.47**
**十四、6000kW 及以上电厂发电标准煤耗**	**g/kWh**	**287.2**	**288.8**	**-1.55**
**十五、6000kW 及以上电厂供电标准煤耗**	**g/kWh**	**304.9**	**306.4**	**-1.50**
**十六、6000kW 及以上电厂厂用电率**	**%**	**4.65**	**4.67**	**-0.02**
水　电	%	0.25	0.24	0.003
火　电	%	5.98	6.01	-0.03
**十七、6000kW 及以上电厂发电设备利用小时**	**h**	**3756**	**3828**	**-72**
水　电	h	3825	3697	128
其中：抽水蓄能	h	1094	1053	40
火　电	h	4211	4307	-97
其中：燃煤发电	h	4323	4429	-106
燃气发电	h	2610	2646	-37
核　电	h	7450	7394	56
风　电	h	2078	2083	-5
太阳能发电	h	1281	1291	-10
**十八、6000kW 及以上电厂燃料消耗**				
发电消耗标煤量	万 t	139561	132007	5.72
发电消耗原煤量	万 t	208088	199443	4.33
供热消耗标煤量	万 t	22678	19463	16.52
供热消耗原煤量	万 t	29710	29227	1.65
**十九、供、售电量及线损**				
供电量	亿 kWh	65232	62835	3.81
售电量	亿 kWh	61581	59111	4.18
线损电量	亿 kWh	3651	3724	-1.97
线损率	%	5.60	5.93	-0.33
**二十、发用电设备比**				
发电装机容量:用电设备容量		1:4.32	1:4.08	
**二十一、电力弹性系数**				
电力生产弹性系数		1.78	0.78	
电力消费弹性系数		1.41	0.73	

注　电源投资完成额口径为全国主要发电企业。

陕西用电量不含陕西省地方电力（集团）有限公司经营区范围内的部分自备电厂用电量，下同。

(C)1994-2024 China Academic Journal Electronic Publishing House. All rights reserved.　http://www.cnki.net
