# Supplementary material for: Prediction analysis of carbon emission in China’s electricity industry based on the dual carbon background
Source: PLoS One. 2024 May 17;19(5):e0302068. doi: 10.1371/journal.pone.0302068 (PMC11101092; doi:10.1371/journal.pone.0302068)
Supplement: S2 File — (PDF) [file pone.0302068.s002.pdf]

4-1 中国能源平衡表(标准量)-2000

(万吨标准煤)

		能源合计 Energy Total	
		(发电煤耗计算法)(coal equivalent calculation)	(电热当量计算法)(calorific value calculation)
**一、可供本地区消费的能源量**	**Total Primary Energy Supply**	**115149.81**	**109495.79**
1.一次能源生产量	Indigenous Production	106988.20	101130.09
水　电	Hydro Power	8181.59	2733.47
核　电	Nuclear Power	615.68	205.70
2.回收能	Recovery of Energy	1759.74	1759.74
3.进口量	Import	13874.40	13836.53
4.我轮、机在外国加油量	China Airplanes&ships Refueling in Abroad	456.77	456.77
5.出口量(-)	Export (-)	-8855.88	-8613.92
6.外轮、机在我国加油量(-)	Foreign Airplanes&ships Refueling in China	-170.09	-170.09
7.库存增(-)、减(+)量	Stock Change	1096.67	1096.67
**二、加工转换投入(-)产出(+)量**	**Input(-) & Output(+) of Transformation**	**-2371.41**	**-30539.25**
1.火力发电	Thermal Power		-27347.84
2.供　热	Heating Supply		-820.00
3.洗选煤	Coal Washing	-916.64	-916.64
4.炼　焦	Coking	-487.29	-487.29
5.炼　油	Petroleum Refineries	-780.55	-780.55
6.制　气	Gas Works	-45.41	-45.41
#焦炭再投入量(-)	Coke Input (-)	-138.78	-138.78
7.煤制品加工	Briquettes	-2.74	-2.74
**三、损失量**	**Loss**	**3893.38**	**1588.99**
**四、终端消费量**	**Total Final Consumption**	**124031.78**	**92517.52**
1.农、林、牧、渔、水利业	Farming, Forestry, Animal Husbandry, Fishery & Water Conservancy	5787.12	4138.36
2.工　业	Industry	83707.35	61700.86
#用作原料、材料	Non-Energy Use	7280.37	7280.37
3.建筑业	Construction	1432.96	1053.09
4.交通运输、仓储及邮电通迅业	Transport, Storage, Postal & Telecommunications Services	9577.29	8884.88
5.批发和零售贸易业、餐饮业	Wholesale, Retail Trade and Catering Service	2893.16	1922.52
6.生活消费	Residential Consumption	14911.83	10685.95
城　镇	Urban	9073.26	6500.76
乡　村	Rural	5838.58	4185.19
7.其　他	Other	5722.04	4131.85
**五、平衡差额**	**Statistical Difference**	**-15146.76**	**-15149.97**
**六、能源消费总量**	**Total Energy Consumption**	**130296.57**	**124645.76**

(C)1994-2024 China Academic Journal Electronic Publishing House. All rights reserved.　http://www.cnki.net

ENERGY BALANCE OF CHINA －2000 (STANDARD QUANTITY)

(10⁴tce)

煤合计 Coal Total	原煤 Raw Coal	洗精煤 Cleaned Coal	其他洗煤 Other Washed Coal	型煤 Briquettes	焦炭 Coke	焦炉煤气 Coke Oven Gas	其他煤气 Other Gas	其他焦化产品 Other Coking Products	油品合计 Petroleum Products Total
**70056.89**	**70444.35**	**-364.07**	**-25.57**	**2.18**	**-1254.76**		**1175.57**	**31.17**	**32448.38**
71287.14	71287.14								23286.18
							1175.57		
161.19	126.96	30.97	3.26					52.53	13603.81
									456.77
-4061.29	-3469.80	-590.45	-0.55	-0.49	-1476.24			-21.36	-2933.63
									-170.09
2669.85	2500.05	195.41	-28.28	2.67	221.48				-1794.66
**-58246.65**	**-61364.90**	**826.91**	**1837.53**	**453.81**	**11022.20**	**1323.37**	**172.05**	**220.22**	**-3056.08**
-38793.19	-37867.35	-187.72	-738.12			-65.67	-201.36		-1690.98
-4734.45	-4572.28	-22.13	-140.04			-59.10	-100.45		-622.22
-916.64	-14758.09	11088.09	2753.36						
-13113.07	-3424.65	-9664.42	-24.00		10943.89	1431.85	43.93	205.41	
									-705.91
-686.56	-293.86	-386.91	-5.79		217.09	16.29	429.93	14.81	-36.97
					-138.78				
-2.74	-448.67		-7.88	453.81					
						**13.03**			**276.12**
**27993.16**	**25206.77**	**966.49**	**1362.74**	**457.16**	**9356.46**	**1336.87**	**1346.95**	**249.50**	**28830.71**
994.53	981.27		13.26		131.01				2183.87
20327.06	18550.44	958.75	813.21	4.66	9029.83	1085.77	1078.33	249.50	12674.95
516.92	501.10	10.28	5.54		567.67			51.43	5193.31
324.94	313.87	3.99	7.08		17.25				505.15
688.65	671.65	3.65	13.33	0.02	10.21	0.12	0.60		7804.46
491.94	482.69	0.09	5.37	3.79	32.45	11.35	7.59		811.28
4718.38	3843.61		428.16	446.61	124.67	220.87	257.24		2087.82
1830.64	1341.35		199.83	289.46	61.39	220.87	254.97		1685.47
2887.74	2502.26		228.33	157.15	63.28		2.27		402.35
447.66	363.24	0.01	82.33	2.08	11.04	18.76	3.19		2763.18
**-16182.92**	**-16127.32**	**-503.65**	**449.22**	**-1.17**	**410.98**	**-26.53**	**0.67**	**1.89**	**285.47**

(C)1994-2024 China Academic Journal Electronic Publishing House. All rights reserved. http://www.cnki.net

(万吨标准煤)

		原油<br>Crude Oil	汽油<br>Gasoline
**一、可供本地区消费的能源量**	**Total Primary Energy Supply**	**30547.73**	**-927.24**
1.一次能源生产量	Indigenous Production	23286.18	
水电	Hydro Power		
核电	Nuclear Power		
2.回收能	Recovery of Energy		
3.进口量	Import	10038.10	0.04
4.我轮、机在外国加油量	China Airplanes&ships Refueling in Abroad		
5.出口量(-)	Export (-)	-1472.32	-669.78
6.外轮、机在我国加油量(-)	Foreign Airplanes&ships Refueling in China		-18.39
7.库存增(-)、减(+)量	Stock Change	-1304.23	-239.11
**二、加工转换投入(-)产出(+)量**	**Input(-) & Output(+) of Transformation**	**-29149.58**	**6081.99**
1.火力发电	Thermal Power	-121.43	-1.47
2.供热	Heating Supply	-20.00	-0.29
3.洗选煤	Coal Washing		
4.炼焦	Coking		
5.炼油	Petroleum Refineries	-29008.15	6083.75
6.制气	Gas Works		
#焦炭再投入量(-)	Coke Input (-)		
7.煤制品加工	Briquettes		
**三、损失量**	**Loss**	**272.73**	
**四、终端消费量**	**Total Final Consumption**	**909.72**	**5155.37**
1.农、林、牧、渔、水利业	Farming, Forestry, Animal Husbandry, Fishery & Water Conservancy		271.49
2.工业	Industry	874.78	883.99
#用作原料、材料	Non-Energy Use	139.40	13.24
3.建筑业	Construction	4.71	170.02
4.交通运输、仓储及邮电通迅业	Transport, Storage, Postal & Telecommunications Services	27.97	2041.98
5.批发和零售贸易业、餐饮业	Wholesale, Retail Trade and Catering Service	0.26	308.76
6.生活消费	Residential Consumption		187.72
城镇	Urban		140.03
乡村	Rural		47.69
7.其他	Other	2.00	1291.41
**五、平衡差额**	**Statistical Difference**	**215.70**	**-0.62**
**六、能源消费总量**	**Total Energy Consumption**		

(C)1994-2024 China Academic Journal Electronic Publishing House. All rights reserved. http://www.cnki.net

Continued(2000)

(10^4 tce)

煤油 Kerosene	柴油 Diesel Oil	燃料油 Fuel Oil	液化石油气 PLG	炼厂干气 Refinery Gas	其他石油制品 Other Petroleum Products	天然气 Natural Gas	热力 Heat	电力 Electricity	其他能源 Other Energy
**12.65**	**-398.00**	**2547.17**	**822.08**		**-156.01**	**3617.60**		**2836.77**	**584.17**
						3617.60		2939.17	
								2733.47	
								205.70	
									584.17
375.90	37.78	2114.50	825.85		211.64			19.00	
98.61	37.88	320.28							
-292.63	-80.84	-47.67	-2.74		-367.65			-121.40	
-84.55	-32.11	-35.04							
-84.68	-360.71	195.10	-1.03						
**1283.49**	**9983.99**	**1317.66**	**1568.10**	**890.23**	**4968.04**	**-391.82**	**4983.24**	**13721.16**	**-286.94**
	-331.72	-1163.19	-3.26	-39.28	-30.63	-202.29		13721.16	-115.51
		-416.05		-160.19	-25.69	-189.53	4983.24		-97.49
									0.70
1283.49	10315.71	2933.87	1571.36	1089.70	5024.36				-74.64
		-36.97							
			**3.39**			**88.58**	**60.04**	**1151.22**	
**1279.55**	**9539.05**	**3916.40**	**2336.23**	**882.36**	**4812.03**	**2778.51**	**4923.04**	**15405.11**	**297.21**
2.21	1909.00	0.57	0.60				1.88	827.07	
123.52	1994.48	2633.95	469.84	882.36	4812.03	2270.18	3974.95	10713.08	297.21
0.24	10.55	141.63	12.86	63.36	4812.03	653.83			297.21
5.89	285.39	23.87	15.27			10.91	4.63	190.21	
788.52	3706.58	1214.31	25.10			13.30	21.95	345.59	
17.66	372.93	16.56	95.11			45.75	38.33	483.83	
106.19	99.60		1694.31			429.86	792.28	2054.83	
9.56	63.86		1472.02			429.86	792.28	1225.28	
96.63	35.74		222.29					829.55	
235.56	1171.07	27.14	36.00			8.51	89.02	790.49	
16.59	46.94	-51.57	50.56	7.87		358.69	0.16	1.60	0.02
							85.87	33.41	

(C)1994-2024 China Academic Journal Electronic Publishing House. All rights reserved. http://www.cnki.net

4-1 中国能源平衡表(实物量)-2000

		煤合计<br>Coal Total<br>万吨<br>10^4 tn	原煤<br>Raw Coal<br>万吨<br>10^4 tn
**一、可供本地区消费的能源量**	**Total Primary Energy Supply**	**98176.09**	**98620.12**
1.一次能源生产量	Indigenous Production	99800.00	99800.00
水电	Hydro Power		
核电	Nuclear Power		
2.回收能	Recovery of Energy		
3.进口量	Import	217.88	177.74
4.我轮、机在外国加油量	China Airplanes&Ships Refueling in Abroad		
5.出口量(-)	Export (-)	-5506.47	-4857.62
6.外轮、机在我国加油量(-)	Foreign Airplanes&Ships Refueling in China		
7.库存增(-)、减(+)量	Stock Change	3664.68	3500.00
**二、加工转换投入(-)产出(+)量**	**Input(-) & Output(+) of Transformation**	**-78450.61**	**-83608.52**
1.火力发电	Thermal Power	-54611.20	-53000.10
2.供热	Heating Supply	-6692.07	-6401.17
3.洗选煤	Coal Washing	-1441.20	-18833.70
4.炼焦	Coking	-15000.40	-4370.41
5.炼油	Petroleum Refineries		
6.制气	Gas Works	-809.99	-375.01
#焦炭再投入量(-)	Coke Input (-)		
7.煤制品加工	Briquettes	104.25	-628.13
**三、损失量**	**Loss**		
**四、终端消费量**	**Total Final Consumption**	**46086.79**	**41675.85**
1.农、林、牧、渔、水利业	Farming, Forestry, Animal Husbandry, Fishery & Water Conservancy	1647.68	1622.39
2.工业	Industry	33279.74	30670.56
#用作原料、材料	Non-Energy Use	850.32	828.50
3.建筑业	Construction	536.82	518.94
4.交通运输、仓储及邮电通迅业	Transport, Storage, Postal & Telecommunications Services	1139.94	1110.48
5.批发和零售贸易业、餐饮业	Wholesale, Retail Trade and Catering Service	814.64	798.06
6.生活消费	Residential Consumption	7906.96	6354.86
城镇	Urban	3075.56	2217.73
乡村	Rural	4831.40	4137.13
7.其他	Other	761.01	600.56
**五、平衡差额**	**Statistical Difference**	**-26361.31**	**-26664.25**

(C)1994-2024 China Academic Journal Electronic Publishing House. All rights reserved. http://www.cnki.net

ENERGY BANLANCE OF CHINA －2000(PHYSICAL QUANTITY)

洗精煤<br>Cleaned Coal<br>万吨<br>10^4 tn	其他洗煤<br>Other Washed Coal<br>万吨<br>10^4 tn	型煤<br>Briquettes<br>万吨<br>10^4 tn	焦炭<br>Coke<br>万吨<br>10^4 tn	焦炉煤气<br>Coke Oven Gas<br>亿立方米<br>10^8 cu.m	其他煤气<br>Other Gas<br>亿立方米<br>10^8 cu.m	其他焦化产品<br>Other Coking Products<br>万吨<br>10^4 tn	油品合计<br>Petroleum Products Total<br>万吨<br>10^4 tn	原油<br>Crude Oil<br>万吨<br>10^4 tn
**-398.93**	**-48.70**	**3.60**	**-1291.70**		**408.78**	**27.01**	**22631.77**	**21382.99**
							16300.00	16300.00
					408.78			
33.94	6.20					45.52	9431.28	7026.53
							317.21	
-647.00	-1.05	-0.80	-1519.70			-18.51	-2055.61	-1030.60
							-116.53	
214.13	-53.85	4.40	228.00				-1244.58	-912.94
**906.10**	**3504.43**	**747.38**	**12041.15**	**221.45**	**59.87**	**190.83**	**-2352.92**	**-20404.30**
-205.70	-1405.40			-10.69	-70.02		-1178.15	-85.00
-24.25	-266.65			-9.62	-34.93		-426.97	-14.00
12150.00	5242.50							
-10589.99	-40.00		11960.54	239.04	15.28	178.00		
							-721.92	-20305.30
-423.96	-11.02		223.48	2.72	149.54	12.83	-25.88	
			-142.87					
	-15.00	747.38						
				**2.18**			**192.89**	**190.91**
**1059.06**	**2598.98**	**752.90**	**10297.14**	**223.71**	**468.42**	**216.20**	**19893.51**	**636.80**
	25.29		144.18				1496.90	
1050.58	1550.93	7.67	9937.68	181.69	375.00	216.20	9016.23	612.34
11.26	10.56		624.74			44.57	3932.00	97.58
4.37	13.51		18.98				344.33	3.30
4.00	25.42	0.04	11.24	0.02	0.21		5351.71	19.58
0.10	10.24	6.24	35.71	1.90	2.64		545.03	0.18
	816.58	735.52	137.20	36.96	89.46		1256.45	
	381.12	476.71	67.56	36.96	88.67		1004.17	
	435.46	258.81	69.64		0.79		252.28	
0.01	157.01	3.43	12.15	3.14	1.11		1882.86	1.40
**-551.89**	**856.75**	**-1.92**	**452.31**	**-4.44**	**0.23**	**1.64**	**192.45**	**150.98**

(C)1994-2024 China Academic Journal Electronic Publishing House. All rights reserved. http://www.cnki.net

续表(2000)

		汽油<br>Gasoline<br>万吨<br>10^4tn	煤油<br>Kerosene<br>万吨<br>10^4tn
**一、可供本地区消费的能源量**	**Total Primary Energy Supply**	**-630.18**	**8.60**
1.一次能源生产量	Indigenous Production		
水 电	Hydro Power		
核 电	Nuclear Power		
2.回 收 能	Recovery of Energy		
3.进 口 量	Import	0.03	255.47
4.我轮、机在外国加油量	China Airplanes&Ships Refueling in Abroad		67.02
5.出 口 量(-)	Export (-)	-455.20	-198.88
6.外轮、机在我国加油量(-)	Foreign Airplanes&ships Refueling in China	-12.50	-57.46
7.库存增(-)、减(+)量	Stock Change	-162.51	-57.55
**二、加工转换投入(-)产出(+)量**	**Input(-) & Output(+) of Transformation**	**4133.47**	**872.29**
1.火力发电	Thermal Power	-1.00	
2.供 热	Heating Supply	-0.20	
3.洗 选 煤	Coal Washing		
4.炼 焦	Coking		
5.炼 油	Petroleum Refineries	4134.67	872.29
6.制 气	Gas Works		
#焦炭再投入量(-)	Coke Input (-)		
7.煤制品加工	Briquettes		
**三、损 失 量**	**Loss**		
**四、终端消费量**	**Total Final Consumption**	**3503.71**	**869.61**
1.农、林、牧、渔、水利业	Farming, Forestry, Animal Husbandry, Fishery & Water Conservancy	184.51	1.50
2.工 业	Industry	600.78	83.95
#用作原料、材料	Non-Energy Use	9.00	0.16
3.建 筑 业	Construction	115.55	4.00
4.交通运输、仓储及邮电通迅业	Transport, Storage, Postal & Telecommunications Services	1387.78	535.90
5.批发和零售贸易业、餐饮业	Wholesale, Retail Trade and Catering Service	209.84	12.00
6.生活消费	Residential Consumption	127.58	72.17
城 镇	Urban	95.17	6.50
乡 村	Rural	32.41	65.67
7.其 他	Other	877.67	160.09
**五、平衡差额**	**Statistical Difference**	**-0.42**	**11.28**

(C)1994-2024 China Academic Journal Electronic Publishing House. All rights reserved. http://www.cnki.net

Continued(2000)

柴油 Diesel Oil 万吨 10^4tn	燃料油 Fuel Oil 万吨 10^4tn	液化石油气 PLG 万吨 10^4tn	炼厂干气 Refinery Gas 万吨 10^4tn	其他石油制品 Other Petroleum Products 万吨 10^4tn	天然气 Natural Gas 亿立方米 10^8 cu. m	热力 Heat 万百万千焦 10^{10} kj	电力 Electricity 亿千瓦小时 10^8kwh	其他能源 Other Energy 万吨标煤 10^4tce
**-273.14**	**1782.98**	**479.54**		**-119.02**	**272.00**		**2308.19**	**584.17**
					272.00		2391.51	
							2224.14	
							167.37	
								584.17
25.93	1480.12	481.74		161.46			15.46	
26.00	224.19							
-55.48	-33.37	-1.60		-280.48			-98.78	
-22.04	-24.53							
-247.55	136.57	-0.60						
**6851.96**	**922.34**	**914.72**	**566.52**	**3790.08**	**-29.46**	**146136.16**	**11164.49**	**-286.94**
-227.66	-814.22	-1.90	-25.00	-23.37	-15.21		11164.49	-115.51
	-291.23		-101.94	-19.60	-14.25	146136.16		-97.49
								0.70
7079.62	2053.67	916.62	693.46	3833.05				-74.64
	-25.88							
		**1.98**			**6.66**	**1760.58**	**936.71**	
**6546.61**	**2741.42**	**1362.79**	**561.51**	**3671.06**	**208.91**	**144370.70**	**12534.67**	**297.21**
1310.14	0.40	0.35				55.08	672.96	
1368.80	1843.72	274.07	561.51	3671.06	170.69	116567.55	8716.91	297.21
7.24	99.14	7.50	40.32	3671.06	49.16			297.21
195.86	16.71	8.91			0.82	135.64	154.77	
2543.81	850.00	14.64			1.00	643.71	281.20	
255.94	11.59	55.48			3.44	1124.12	393.68	
68.36		988.34			32.32	23234.03	1671.95	
43.83		858.67			32.32	23234.03	996.97	
24.53		129.67					674.98	
803.70	19.00	21.00			0.64	2610.57	643.20	
**32.21**	**-36.10**	**29.49**	**5.01**		**26.97**	**4.88**	**1.30**	**0.02**

(C)1994-2024 China Academic Journal Electronic Publishing House. All rights reserved. http://www.cnki.net

4-2 中国能源平衡表(标准量)-2001

(万吨标准煤)

		能源合计 Energy Total	
		(发电煤耗计算法) (coal equivalent calculation)	(电热当量计算法) (calorific value calculation)
**一、可供本地区消费的能源量**	**Total Primary Energy Supply**	**125309.78**	**118615.29**
1.一次能源生产量	Indigenous Production	120900.00	113996.27
水　电	Hydro Power	10325.98	3409.64
核　电	Nuclear Power	650.30	214.73
2.回 收 能	Recovery of Energy	1858.54	1858.54
3.进 口 量	Import	12967.56	12922.73
4.我轮、机在外国加油量	China Airplanes&ships Refueling in Abroad	503.49	503.49
5.出 口 量(-)	Export (-)	-10941.18	-10687.11
6.外轮、机在我国加油量(-)	Foreign Airplanes&ships Refueling in China	-203.53	-203.53
7.库存增(-)、减(+)量	Stock Change	225	225
**二、加工转换投入(-)产出(+)量**	**Input(-) & Output(+) of Transformation**	**-2011.03**	**-32291.30**
1.火力发电	Thermal Power		-29336.21
2.供　热	Heating Supply		-944.61
3.洗 选 煤	Coal Washing	-792.33	-791.88
4.炼　焦	Coking	-386.71	-387.18
5.炼　油	Petroleum Refineries	-635.67	-635.67
6.制　气	Gas Works	-43.11	-43.11
#焦炭再投入量(-)	Coke Input (-)	-149.26	-149.26
7.煤制品加工	Briquettes	-3.82	-3.38
**三、损 失 量**	**Loss**	**3953.09**	**1693.30**
**四、终端消费量**	**Total Final Consumption**	**128950.63**	**94466.57**
1.农、林、牧、渔、水利业	Farming, Forestry, Animal Husbandry, Fishery & Water Conservancy	6232.83	4331.86
2.工　业	Industry	86710.88	62488.43
#用作原料、材料	Non-Energy Use	7594.09	7594.09
3.建 筑 业	Construction	1452.39	1090.17
4.交通运输、仓储及邮电通迅业	Transport, Storage, Postal & Telecommunications Services	9929.20	9128.13
5.批发和零售贸易业、餐饮业	Wholesale, Retail Trade and Catering Service	3164.51	2048.23
6.生活消费	Residential Consumption	15426.56	11076.91
城　镇	Urban	9468.25	6745.85
乡　村	Rural	5958.31	4331.06
7.其　他	Other	6034.25	4302.85
**五、平衡差额**	**Statistical Difference**	**-9604.97**	**-9835.88**
**六、能源消费总量**	**Total Energy Consumption**	**134914.75**	**128451.17**

(C)1994-2024 China Academic Journal Electronic Publishing House. All rights reserved. http://www.cnki.net

ENERGY BALANCE OF CHINA - 2001 (STANDARD QUANTITY)

(10^4tce)

煤合计 Coal Total	原煤 Raw Coal	洗精煤 Cleaned Coal	其他洗煤 Other Washed Coal	型煤 Briquettes	焦炭 Coke	焦炉煤气 Coke Oven Gas	其他煤气 Other Gas	其他焦化产品 Other Coking Products	油品合计 Petroleum Products Total
**77506.12**	**78323**	**-725.38**	**-91.07**	**-0.43**	**-1620.13**		**1242.35**	**40.51**	**33275.67**
82915.00	82915								23423.14
							1242.35		
192.84	159	25.25	8.59					54.81	12652.98
									503.49
-6482.59	-5619	-862.86	-0.55	-0.18	-1345.00			-14.30	-2719.57
									-203.53
881	868	112	-99		-275				-381
**-61687.07**	**-64901**	**944.90**	**1791.16**	**477.87**	**11878.90**	**1145.54**	**135.02**	**235.34**	**-2983.97**
-41438.58	-40598	-176.46	-664.12			-65.24	-241.62	-0.76	-1740.79
-5043.97	-4909	-24.12	-110.85			-43.19	-115.38	-1.03	-637.83
-791.88	-14848	11462.68	2593.44						
-13671.60	-3798	-9858.41	-15.19		11795.50	1235.54	32.77	220.41	
									-572.84
-737.66	-272	-458.79	-6.87		232.66	18.43	459.25	16.72	-32.51
					-149.26				
-3.38	-476		-5.25	477.87					
						**13.48**			**265.73**
**26977.54**	**24247**	**926.36**	**1325.89**	**478.29**	**9838.14**	**1151.52**	**1376.89**	**266.35**	**29499.15**
978.47	965		13.47		126.30				2288.25
19349.04	17671	919.16	754.63	4.25	9510.82	950.30	1123.61	266.35	12723.45
523.16	508	9.91	5.25		703.07			55.23	5229.41
329.42	319	3.59	6.83		21.69				546.04
643.11	627	3.51	12.57	0.03	10.60	0.10	0.72		8079.77
495.46	486	0.09	5.18	4.19	36.04	9.95	8.63		845.19
4720.75	3804		449.07	467.68	121.73	175.90	240.42		2148.43
1831.08	1319		210.10	301.98	61.91	175.90	238.09		1719.29
2889.67	2485		238.97	165.70	59.82		2.33		429.14
461.29	375	0.01	84.14	2.14	10.96	15.27	3.51		2868.02
**-11158.49**	**-10825**	**-706.84**	**374.20**	**-0.85**	**420.63**	**-19.46**	**0.48**	**9.50**	**526.82**

(C)1994-2024 China Academic Journal Electronic Publishing House. All rights reserved. http://www.cnki.net

续表(2001)

(万吨标准煤)

		原油 Crude Oil	汽油 Gasoline
**一、可供本地区消费的能源量**	**Total Primary Energy Supply**	**30767.99**	**-806.04**
1.一次能源生产量	Indigenous Production	23423.14	
水 电	Hydro Power		
核 电	Nuclear Power		
2.回 收 能	Recovery of Energy		
3.进 口 量	Import	8608.74	0.03
4.我轮、机在外国加油量	China Airplanes&ships Refueling in Abroad		
5.出 口 量(-)	Export (-)	-1078.59	-842.32
6.外轮、机在我国加油量(-)	Foreign Airplanes&ships Refueling in China		-19.86
7.库存增(-)、减(+)量	Stock Change	-185.30	56.11
**二、加工转换投入(-)产出(+)量**	**Input(-) & Output(+) of Transformation**	**-29315.89**	**6112.11**
1.火力发电	Thermal Power	-116.57	-0.88
2.供 热	Heating Supply	-17.60	-0.18
3.洗 选 煤	Coal Washing		
4.炼 焦	Coking		
5.炼 油	Petroleum Refineries	-29181.72	6113.17
6.制 气	Gas Works		
#焦炭再投入量(-)	Coke Input (-)		
7.煤制品加工	Briquettes		
**三、损 失 量**	**Loss**	**263.26**	
**四、终端消费量**	**Total Final Consumption**	**916.45**	**5292.67**
1.农、林、牧、渔、水利业	Farming, Forestry, Animal Husbandry, Fishery & Water Conservancy		280.45
2.工 业	Industry	883.81	908.47
#用作原料、材料	Non-Energy Use	142.49	12.45
3.建 筑 业	Construction	4.72	171.71
4.交通运输、仓储及邮电通迅业	Transport, Storage, Postal & Telecommunications Services	26.03	2088.46
5.批发和零售贸易业、餐饮业	Wholesale, Retail Trade and Catering Service	0.21	314.94
6.生活消费	Residential Consumption		198.05
城 镇	Urban		147.02
乡 村	Rural		51.03
7.其 他	Other	1.68	1330.59
**五、平衡差额**	**Statistical Difference**	**272.39**	**13.40**
**六、能源消费总量**	**Total Energy Consumption**		

(C)1994-2024 China Academic Journal Electronic Publishing House. All rights reserved. http://www.cnki.net

Continued(2001)

(10⁴ tce)

煤油 Kerosene	柴油 Diesel Oil	燃料油 Fuel Oil	液化石油气 PLG	炼厂干气 Refinery Gas	其他石油制品 Other Petroleum Products	天然气 Natural Gas	热力 Heat	电力 Electricity	其他能源 Other Energy
**154.32**	**-311.19**	**2817.86**	**815.49**		**-162.76**	**4033.36**		**3521.22**	**616.19**
						4033.76		3624.37	
								3409.64	
								214.73	
									616.19
297.06	40.03	2605.19	838.05		263.88			22.10	
142.28	39.66	321.55							
-268.12	-37.33	-62.99	-3.58		-426.64	-0.40		-125.25	
-94.46	-30.95	-58.26							
77.56	-322.60	12.37	-18.98						
**1161.45**	**10557.00**	**1004.98**	**1630.78**	**864.27**	**5001.33**	**-391.15**	**5224.24**	**14462.29**	**-310.44**
	-350.27	-1197.94	-1.80	-36.14	-37.19	-172.90		14462.29	-138.61
		-428.04		-157.14	-34.87	-218.25	5224.24		-109.20
1161.45	10907.27	2663.47	1632.58	1057.55	5073.39				-62.83
		-32.51							
			**2.47**			**82.73**	**61.23**	**1270.13**	
**1309.95**	**10006.32**	**3841.91**	**2414.87**	**878.41**	**4838.57**	**3174.32**	**5162.84**	**16714.39**	**305.43**
2.24	2004.45	0.60	0.51				1.86	936.98	
126.54	2034.73	2554.90	498.02	878.41	4838.57	2486.31	4206.77	11566.35	305.43
0.26	13.46	136.75	16.29	69.14	4838.57	777.79			305.43
5.15	325.05	23.11	16.30			9.58	5.35	178.09	
825.00	3891.92	1221.45	26.91			15.96	23.43	380.15	
18.35	390.61	17.54	103.54			66.50	39.69	546.77	
110.36	115.36		1724.66			586.66	796.90	2260.41	
10.33	74.49		1487.45			586.66	796.90	1310.31	
100.03	40.87		237.21					950.10	
222.31	1244.20	24.31	44.93			9.31	88.86	845.63	
**5.82**	**239.49**	**-19.07**	**28.93**	**-14.14**		**385.16**	**0.17**	**-1.01**	**0.32**

(C)1994-2024 China Academic Journal Electronic Publishing House. All rights reserved. http://www.cnki.net

4-2 中国能源平衡表(实物量)-2001

		煤合计<br>Coal Total<br>万吨<br>10^4 tn	原煤<br>Raw Coal<br>万吨<br>10^4 tn
**一、可供本地区消费的能源量**	**Total Primary Energy Supply**	**108480.02**	**109648.00**
1.一次能源生产量	Indigenous Production	116078.00	116078.00
水电	Hydro Power		
核电	Nuclear Power		
2.回收能	Recovery of Energy		
3.进口量	Import	266.02	222.00
4.我轮、机在外国加油量	China Airplanes&Ships Refueling in Abroad		
5.出口量(-)	Export (-)	-9012.87	-7867.00
6.外轮、机在我国加油量(-)	Foreign Airplanes&Ships Refueling in China		
7.库存增(-)、减(+)量	Stock Change	1148.87	1215.00
**二、加工转换投入(-)产出(+)量**	**Input(-) & Output(+) of Transformation**	**-82320.07**	**-87755.00**
1.火力发电	Thermal Power	-57687.86	-56230.00
2.供热	Heating Supply	-6961.49	-6724.00
3.洗选煤	Coal Washing	-1450.54	-18949.00
4.炼焦	Coking	-15436.37	-4807.00
5.炼油	Petroleum Refineries		
6.制气	Gas Works	-893.81	-378.00
#焦炭再投入量(-)	Coke Input (-)		
7.煤制品加工	Briquettes	110.00	-667.00
**三、损失量**	**Loss**		
**四、终端消费量**	**Total Final Consumption**	**43891.27**	**39551.00**
1.农、林、牧、渔、水利业	Farming, Forestry, Animal Husbandry, Fishery & Water Conservancy	1599.64	1574.00
2.工业	Industry	31287.92	28824.00
#用作原料、材料	Non-Energy Use	850.00	829.00
3.建筑业	Construction	537.98	521.00
4.交通运输、仓储及邮电通迅业	Transport, Storage, Postal & Telecommunications Services	1050.88	1023.00
5.批发和零售贸易业、餐饮业	Wholesale, Retail Trade and Catering Service	809.87	793.00
6.生活消费	Residential Consumption	7830.25	6205.00
城镇	Urban	3048.36	2151.00
乡村	Rural	4781.89	4054.00
7.其他	Other	774.73	611.00
**五、平衡差额**	**Statistical Difference**	**-17731.32**	**-17658.00**

(C)1994-2024 China Academic Journal Electronic Publishing House. All rights reserved. http://www.cnki.net

ENERGY BANLANCE OF CHINA－2001(PHYSICAL QUANTITY)

洗精煤<br>Cleaned Coal<br>万吨<br>10^4 tn	其他洗煤<br>Other Washed Coal<br>万吨<br>10^4 tn	型煤<br>Briquettes<br>万吨<br>10^4 tn	焦炭<br>Coke<br>万吨<br>10^4 tn	焦炉煤气<br>Coke Oven Gas<br>亿立方米<br>10^8 cu. m	其他煤气<br>Other Gas<br>亿立方米<br>10^8 cu. m	其他焦化产品<br>Other Coking Products<br>万吨<br>10^4 tn	油品合计<br>Petroleum Products Total<br>万吨<br>10^4 tn	原油<br>Crude Oil<br>万吨<br>10^4 tn
**－993.88**	**－173.39**	**－0.71**	**－1667.83**		**432.00**	**35.11**	**23204.67**	**21382.99**
							16395.87	16300.00
					432.00			
27.67	16.35					47.50	8769.15	7026.53
							349.00	
－1144.53	－1.04	－0.30	－1384.60			－12.39	－1906.96	－1030.60
							－139.72	
122.98	－188.70	－0.41	－283.23				－262.67	－912.94
**1237.50**	**3410.43**	**787.00**	**12977.12**	**230.35**	**47.00**	**203.94**	**－2291.99**	**－20404.30**
－193.36	－1264.50			－10.62	－84.02	－0.66	－1213.55	－85.00
－26.43	－211.06			－7.03	－40.12	－0.89	－438.66	－14.00
12560.46	4938.00							
－10600.44	－28.93		12891.26	245.00	11.40	191.00		
							－617.02	－20305.30
－502.73	－13.08		239.51	3.00	159.74	14.49	－22.76	
			－153.65					
	－10.00	787.00						
				**2.71**			**189.34**	**190.91**
**1028.06**	**2524.52**	**787.69**	**10845.58**	**231.55**	**478.83**	**230.82**	**20356.97**	**636.80**
	25.64		139.23				1568.48	
1020.07	1436.85	7.00	10484.75	191.09	390.75	230.82	9059.88	612.34
11.00	10.00		775.07			47.86	3960.11	97.58
3.98	13.00		23.91				372.34	3.30
3.90	23.93	0.05	11.68	0.02	0.25		5540.34	19.58
0.10	9.87	6.90	39.73	2.00	3.00		567.41	0.18
	855.03	770.22	134.20	35.37	83.61		1294.81	
	400.03	497.33	68.25	35.37	82.80		1025.73	
	455.00	272.89	65.95		0.81		269.08	
0.01	160.20	3.52	12.08	3.07	1.22		1953.71	1.40
**－784.44**	**712.52**	**－1.40**	**463.71**	**－3.91**	**0.17**	**8.23**	**366.37**	**150.98**

(C)1994-2024 China Academic Journal Electronic Publishing House. All rights reserved. http://www.cnki.net

		汽油<br>Gasoline<br>万吨<br>10^4tn	煤油<br>Kerosene<br>万吨<br>10^4tn
**一、可供本地区消费的能源量**	**Total Primary Energy Supply**	**-547.80**	**104.88**
1.一次能源生产量	Indigenous Production		
水　电	Hydro Power		
核　电	Nuclear Power		
2.回 收 能	Recovery of Energy		
3.进 口 量	Import	0.02	201.89
4.我轮、机在外国加油量	China Airplanes&Ships Refueling in Abroad		96.70
5.出 口 量(-)	Export (-)	-572.46	-182.22
6.外轮、机在我国加油量(-)	Foreign Airplanes&ships Refueling in China	-13.50	-64.20
7.库存增(-)、减(+)量	Stock Change	38.14	52.71
**二、加工转换投入(-)产出(+)量**	**Input(-) & Output(+) of Transformation**	**4153.94**	**789.35**
1.火力发电	Thermal Power	-0.60	
2.供　热	Heating Supply	-0.12	
3.洗 选 煤	Coal Washing		
4.炼　焦	Coking		
5.炼　油	Petroleum Refineries	4154.66	789.35
6.制　气	Gas Works		
#焦炭再投入量(-)	Coke Input (-)		
7.煤制品加工	Briquettes		
**三、损 失 量**	**Loss**		
**四、终端消费量**	**Total Final Consumption**	**3597.03**	**890.27**
1.农、林、牧、渔、水利业	Farming, Forestry, Animal Husbandry, Fishery & Water Conservancy	190.60	1.52
2.工　业	Industry	617.42	86.00
#用作原料、材料	Non-Energy Use	8.46	0.18
3.建 筑 业	Construction	116.70	3.50
4.交通运输、仓储及邮电通迅业	Transport, Storage, Postal & Telecommunications Services	1419.37	560.69
5.批发和零售贸易业、餐饮业	Wholesale, Retail Trade and Catering Service	214.04	12.47
6.生活消费	Residential Consumption	134.60	75.00
城　镇	Urban	99.92	7.02
乡　村	Rural	34.68	67.98
7.其　他	Other	904.30	151.09
**五、平衡差额**	**Statistical Difference**	**9.11**	**3.96**

(C)1994-2024 China Academic Journal Electronic Publishing House. All rights reserved. http://www.cnki.net

Continued(2001)

柴油<br>Diesel Oil<br>万吨<br>10^4tn	燃料油<br>Fuel Oil<br>万吨<br>10^4tn	液化石油气<br>PLG<br>万吨<br>10^4tn	炼厂干气<br>Refinery Gas<br>万吨<br>10^4tn	其他石油制品<br>Other Petroleum Products<br>万吨<br>10^4tn	天然气<br>Natural Gas<br>亿立方米<br>10^8 cu. m	热力<br>Heat<br>万百万千焦<br>10^{10} kj	电力<br>Electricity<br>亿千瓦小时<br>10^8kwh	其他能源<br>Other Energy<br>万吨标煤<br>10^4tce
**-213.57**	**1972.47**	**475.70**		**-124.17**	**303.26**		**2865.11**	**616.19**
					303.29		2949.04	
							2774.32	
							174.72	
								616.19
27.47	1823.60	488.86		201.31			17.98	
27.22	225.08							
-25.62	-44.09	-2.09		-325.48	-0.03		-101.91	
-21.24	-40.78							
-221.40	8.66	-11.07						
**7245.21**	**703.47**	**951.28**	**550.00**	**3815.48**	**-29.41**	**153203.45**	**11767.53**	**-310.44**
-240.39	-838.54	-1.05	-23.00	-28.37	-13.00		11767.53	-138.61
	-299.62		-100.00	-26.60	-16.41	153203.45		-109.20
								0.20
7485.60	1864.39	952.33	673.00	3870.45				-62.83
	-22.76							
		**1.44**			**6.22**	**1795.58**	**1033.47**	
**6867.27**	**2689.30**	**1408.67**	**559.00**	**3691.31**	**238.67**	**151402.97**	**13599.99**	**305.43**
1375.64	0.42	0.30				54.41	762.39	
1396.42	1788.40	290.51	559.00	3691.31	186.94	123365.60	9411.19	305.43
9.24	95.72	9.50	44.00	3691.31	58.48			305.43
223.08	16.18	9.51			0.72	156.94	144.91	
2671.00	855.00	15.70			1.20	687.06	309.32	
268.07	12.28	60.40			5.00	1163.83	444.89	
79.17		1006.04			44.11	23369.40	1839.23	
51.12		867.67			44.11	23369.40	1066.16	
28.05		138.37					773.07	
853.89	17.02	26.21			0.70	2605.73	688.06	
164.37	-13.36	16.87	-9.00		28.96	4.90	-0.82	0.32

(C)1994-2024 China Academic Journal Electronic Publishing House. All rights reserved. http://www.cnki.net

4－3 中国能源平衡表(标准量)－2002

(万吨标准煤)

		能源合计 Energy Total	
		(发电煤耗计算法)(coal equivalent calculation)	(电热当量计算法)(calorific value calculation)
**一、可供本地区消费的能源量**	**Total Primary Energy Supply**	**144318.71**	**136756.00**
1.一次能源生产量	Indigenous Production	138368.81	130622.94
水　电	Hydro Power	10663.45	3539.20
核电	Nuclear Power	930.43	308.81
2.回 收 能	Recovery of Energy	1907.98	1907.98
3.进 口 量	Import	15197.52	15140.61
4.我轮、机在外国加油量	China Airplanes&ships Refueling in Abroad	571.75	571.75
5.出 口 量(－)	Export (－)	－10796.14	－10556.07
6.外轮、机在我国加油量(－)	Foreign Airplanes&ships Refueling in China	－220.87	－220.87
7.库存增(－)、减(＋)量	Stock Change	－710.34	－710
**二、加工转换投入(－)产出(＋)量**	**Input(－) & Output(＋) of Transformation**	**－2611.91**	**－36326.25**
1.火力发电	Thermal Power	2.99	－32835.28
2.供　热	Heating Supply	－0.37	－879.43
3.洗 选 煤	Coal Washing	－952.50	－952.50
4.炼　焦	Coking	－322.39	－322.39
5.炼　油	Petroleum Refineries	－1015.43	－1015.43
6.制　气	Gas Works	－144.39	－144.39
＃焦炭再投入量(－)	Coke Input (－)	－133.42	－133.42
7.煤制品加工	Briquettes	－43.41	－43.41
**三、损 失 量**	**Loss**	**4763.06**	**1861.15**
**四、终端消费量**	**Total Final Consumption**	**140846.56**	**102466.70**
1.农、林、牧、渔、水利业	Farming, Forestry, Animal Husbandry, Fishery & Water Conservancy	6514.29	4593.60
2.工　业	Industry	95143.39	68161.68
＃用作原料、材料	Non-Energy Use	8687.29	8687.29
3.建 筑 业	Construction	1610.14	1203.09
4.交通运输、仓储及邮电通迅业	Transport, Storage, Postal & Telecommunications Services	10749.73	9909.21
5.批发和零售贸易业、餐饮业	Wholesale, Retail Trade and Catering Service	3464.02	2219.21
6.生活消费	Residential Consumption	17031.76	11937.90
城　镇	Urban	10375.14	7340.78
乡　村	Rural	6656.62	4597.12
7.其　他	Other	6333.27	4442.02
**五、平衡差额**	**Statistical Difference**	**－3902.82**	**－3898.10**
**六、能源消费总量**	**Total Energy Consumption**	**148221.53**	**140654.10**

(C)1994-2024 China Academic Journal Electronic Publishing House. All rights reserved. http://www.cnki.net

ENERGY BALANCE OF CHINA－2002 (STANDARD QUANTITY)

(10^4tce)

煤合计<br>Coal Total	原煤<br>Raw Coal	洗精煤<br>Cleaned Coal	其他洗煤<br>Other Washed Coal	型煤<br>Briquettes	焦炭<br>Coke	焦炉煤气<br>Coke Oven Gas	其他煤气<br>Other Gas	其他焦化产品<br>Other Coking Products	油品合计<br>Petroleum Products Total
**92335.43**	**93464**	**－1121.40**	**－6.68**	**－0.08**	**－1382.12**		**1263.63**	**43.16**	**35750.62**
98573.40	98573								23857.62
							1263.63		
806.23	775	23.34	8.22	0.01				57.33	14248.78
									571.75
－6255.24	－5039	－1213.33	－2.74	－0.50	－1318.21			－14.17	－2849.19
									－220.87
－789	－846	69	－12		－64				143
**－69695.27**	**－72479**	**177.53**	**2102.69**	**503.98**	**13712.27**	**1486.68**	**126.32**	**246.63**	**－3408.46**
－46665.73	－45887	－152.68	－626.42			－84.04	－257.01		－1827.83
－5418.46	－5317	－3.56	－97.65			－29.67	－99.36		－613.88
－952.50	－16355	12549.24	2852.78						
－15809.81	－4090	－11706.21	－13.14		13631.47	1583.50	43.13	229.32	
									－939.74
－805.36	－288	－509.26	－7.63		214.22	16.89	439.56	17.31	－27.01
					－133.42				
－43.41	－542		－5.25	503.98					
**0**									**272.28**
**27685.81**	**25053**	**802.76**	**1322.76**	**507.45**	**11857.45**	**1496.13**	**1389.54**	**288.50**	**31849.14**
1058.46	1046		12.02		136.95				2441.96
19744.83	18182	797.04	761.54	3.86	11502.05	1248.52	1120.22	288.50	13814.03
582.02	566	10.11	5.78		778.50			63.47	6154.50
361.11	353	2.53	5.41		22.71				602.31
688.44	677	3.11	8.15	0.30	11.11	0.12	0.60		8745.14
527.47	517	0.08	5.26	5.45	41.38	10.37	11.13		883.23
4823.81	3879		449.66	495.50	131.26	220.84	257.59		2456.71
1775.76	1256		207.87	312.12	67.27	220.84	254.51		1997.83
3048.05	2623		241.79	183.38	63.99		3.08		458.88
481.69	399		80.72	2.34	11.99	16.28			2905.76
**－5045.65**	**－4069**	**－1746.63**	**773.25**	**－3.55**	**472.70**	**－9.45**	**0.41**	**1.29**	**220.74**

(C)1994-2024 China Academic Journal Electronic Publishing House. All rights reserved. http://www.cnki.net

续表(2002)

(万吨标准煤)

		原油 Crude Oil	汽油 Gasoline
**一、可供本地区消费的能源量**	**Total Primary Energy Supply**	**32527.78**	**-840.28**
1.一次能源生产量	Indigenous Production	23857.62	
水 电	Hydro Power		
核 电	Nuclear Power		
2.回 收 能	Recovery of Energy		0
3.进 口 量	Import	9915.40	
4.我轮、机在外国加油量	China Airplanes&ships Refueling in Abroad		0
5.出 口 量(-)	Export (-)	-1094.96	-900.79
6.外轮、机在我国加油量(-)	Foreign Airplanes&ships Refueling in China		-26.79
7.库存增(-)、减(+)量	Stock Change	-150.28	87.30
**二、加工转换投入(-)产出(+)量**	**Input(-) & Output(+) of Transformation**	**-30959.63**	**6356.48**
1.火力发电	Thermal Power	-111.90	-0.91
2.供 热	Heating Supply	-18.23	-0.18
3.洗 选 煤	Coal Washing		0
4.炼 焦	Coking		0
5.炼 油	Petroleum Refineries	-30829.50	6357.57
6.制 气	Gas Works		0
#焦炭再投入量(-)	Coke Input (-)		0
7.煤制品加工	Briquettes		0
**三、损 失 量**	**Loss**	**269.26**	
**四、终端消费量**	**Total Final Consumption**	**973.24**	**5516.22**
1.农、林、牧、渔、水利业	Farming, Forestry, Animal Husbandry, Fishery & Water Conservancy		276.52
2.工 业	Industry	940.02	928.31
#用作原料、材料	Non-Energy Use	160.19	13.24
3.建 筑 业	Construction	6.00	179.98
4.交通运输、仓储及邮电通迅业	Transport, Storage, Postal & Telecommunications Services	25.21	2212.25
5.批发和零售贸易业、餐饮业	Wholesale, Retail Trade and Catering Service	0.17	329.92
6.生活消费	Residential Consumption		241.01
城 镇	Urban		176.42
乡 村	Rural		64.59
7.其 他	Other	1.84	1348.23
**五、平衡差额**	**Statistical Difference**	**325.65**	**-0.02**
**六、能源消费总量**	**Total Energy Consumption**		

(C)1994-2024 China Academic Journal Electronic Publishing House. All rights reserved. http://www.cnki.net

Continued(2002)

(10[4] tce)

煤油<br>Kerosene	柴油<br>Diesel Oil	燃料油<br>Fuel Oil	液化石油气<br>PLG	炼厂干气<br>Refinery Gas	其他石油制品<br>Other Petroleum Products	天然气<br>Natural Gas	热力<br>Heat	电力<br>Electricity	其他能源<br>Other Energy
**130.47**	**23.64**	**2653.34**	**1074.57**		**181.10**	**4343.91**		**3757.02**	**644.35**
						4343.91		3848.01	
								3539.20	
								308.81	
									644.35
315.66	69.53	2370.99	1073.43		503.77			28.27	
161.56	45.17	365.02							
-249.61	-180.23	-91.30	-9.63		-322.67			-119.26	
-104.91	-30.60	-58.57							
7.77	119.77	67.20	10.77						
**1215.54**	**10899.27**	**924.69**	**1774.73**	**883.80**	**5496.66**	**-373.34**	**5598.91**	**16313.46**	**-333.45**
	-329.29	-1303.83	-2.64	-31.90	-47.36	-146.97		16313.46	-167.16
		-380.95		-177.82	-36.70	-226.37	5598.91		-90.60
1215.54	11228.56	2636.48	1777.37	1093.52	5580.72				-75.69
		-27.01							
**0.00**			**3.02**			**84.19**	**68.40**	**1436.28**	
**1352.52**	**10842.51**	**3822.43**	**2768.76**	**895.70**	**5677.76**	**3423.94**	**5530.32**	**18635.07**	**310.80**
2.06	2162.79	0.59					2.24	953.99	
128.53	2193.43	2503.81	546.47	895.70	5677.76	2632.59	4442.63	13057.51	310.80
3.87	37.88	165.53	25.71	70.32	5677.76	798.00			310.80
	367.17	27.29	21.87			9.04	6.19	201.73	
907.49	4320.02	1245.88	34.29			20.75	27.65	415.40	
19.13	409.14	17.57	107.30			81.13	50.00	614.50	
89.31	122.28		2004.11			680.43	907.51	2459.75	
9.30	86.68		1725.43			680.43	907.51	1436.63	
80.01	35.60		278.68					1023.12	
206.00	1267.68	27.29	54.72				94.10	932.20	
**-6.51**	**80.40**	**-244.40**	**77.52**	**-11.90**		**462.44**	**0.19**	**-0.87**	**0.10**

(C)1994-2024 China Academic Journal Electronic Publishing House. All rights reserved. http://www.cnki.net

4－3 中国能源平衡表(实物量)－2002

		煤合计<br>Coal Total<br>万吨<br>10^4 tn	原煤<br>Raw Coal<br>万吨<br>10^4 tn
**一、可供本地区消费的能源量**	**Total Primary Energy Supply**	**129604.75**	**130846.41**
1.一次能源生产量	Indigenous Production	138000.00	138000.00
水　电	Hydro Power		
核　电	Nuclear Power		
2.回 收 能	Recovery of Energy		
3.进 口 量	Import	1125.74	1084.50
4.我轮、机在外国加油量	China Airplanes&Ships Refueling in Abroad		
5.出 口 量(－)	Export (－)	－8389.56	－7053.99
6.外轮、机在我国加油量(－)	Foreign Airplanes&Ships Refueling in China		
7.库存增(－)、减(＋)量	Stock Change	－1131.43	－1184.10
**二、加工转换投入(－)产出(＋)量**	**Input(－) & Output(＋) of Transformation**	**－93913.12**	**－98761.68**
1.火力发电	Thermal Power	－65600.03	－64240.00
2.供　热	Heating Supply	－7473.73	－7283.90
3.洗 选 煤	Coal Washing	－1717.46	－20900.35
4.炼　焦	Coking	－18209.72	－5177.80
5.炼　油	Petroleum Refineries		
6.制　气	Gas Works	－973.20	－400.65
#焦炭再投入量(－)	Coke Input (－)		
7.煤制品加工	Briquettes	61.02	－758.98
**三、损 失 量**	**Loss**		
**四、终端消费量**	**Total Final Consumption**	**42692.41**	**38305.78**
1.农、林、牧、渔、水利业	Farming, Forestry, Animal Husbandry, Fishery & Water Conservancy	1622.89	1600.00
2.工　业	Industry	30282.23	27800.88
#用作原料、材料	Non-Energy Use	889.61	865.61
3.建 筑 业	Construction	553.55	540.00
4.交通运输、仓储及邮电通迅业	Transport, Storage, Postal & Telecommunications Services	1054.96	1034.95
5.批发和零售贸易业、餐饮业	Wholesale, Retail Trade and Catering Service	809.08	790.00
6.生活消费	Residential Consumption	7602.64	5930.45
城　镇	Urban	2829.89	1920.07
乡　村	Rural	4772.75	4010.38
7.其　他	Other	767.06	609.50
**五、平衡差额**	**Statistical Difference**	**－7000.78**	**－6221.05**

(C)1994-2024 China Academic Journal Electronic Publishing House. All rights reserved. http://www.cnki.net

ENERGY BANLANCE OF CHINA - 2002(PHYSICAL QUANTITY)

洗精煤<br>Cleaned Coal<br>万吨<br>10^4 tn	其他洗煤<br>Other Washed Coal<br>万吨<br>10^4 tn	型煤<br>Briquettes<br>万吨<br>10^4 tn	焦炭<br>Coke<br>万吨<br>10^4 tn	焦炉煤气<br>Coke Oven Gas<br>亿立方米<br>10^8 cu.m	其他煤气<br>Other Gas<br>亿立方米<br>10^8 cu.m	其他焦化产品<br>Other Coking Products<br>万吨<br>10^4 tn	油品合计<br>Petroleum Products Total<br>万吨<br>10^4 tn	原油<br>Crude Oil<br>万吨<br>10^4 tn
**-1228.79**	**-12.73**	**-0.14**	**-1422.81**		**439.40**	**37.40**	**24925.09**	**22768.99**
							16700.00	16700.00
					439.40			
25.58	15.65	0.01				49.68	9873.03	6940.64
							396.31	
-1329.53	-5.22	-0.82	-1357.02			-12.28	-1987.68	-766.46
							-151.51	
75.16	-23.16	0.67	-65.79				94.94	-105.19
**14.96**	**4003.60**	**830.00**	**14115.99**	**243.83**	**43.97**	**213.72**	**-2606.76**	**-21671.31**
-167.30	-1192.73			-13.68	-89.37		-1275.57	-78.33
-3.90	-185.93			-4.83	-34.55		-420.70	-12.76
13751.09	5431.80							
-13006.90	-25.02		14032.81	259.59	15.00	198.72		
							-891.58	-21580.22
-558.03	-14.52		220.53	2.75	152.89	15.00	-18.91	
			-137.35					
	-10.00	830.00						
							**190.24**	**188.48**
**1032.35**	**2518.58**	**835.70**	**12206.56**	**245.38**	**483.23**	**250.00**	**21982.83**	**681.26**
	22.89		140.98				1674.05	
1025.00	1450.00	6.35	11840.70	204.77	389.57	250.00	9854.51	658.00
13.00	11.00		801.42			55.00	4656.90	112.13
3.25	10.30		23.38				410.37	4.20
4.00	15.51	0.50	11.44	0.02	0.21		5994.81	17.65
0.10	10.01	8.97	42.60	1.70	3.87		593.02	0.12
	856.17	816.02	135.12	36.22	89.58		1477.47	
	395.80	514.02	69.25	36.22	88.51		1192.20	
	460.37	302.00	65.87		1.07		285.27	
	153.70	3.86	12.34	2.67			1978.60	1.29
**-2246.18**	**1472.29**	**-5.84**	**486.62**	**-1.55**	**0.14**	**1.12**	**145.26**	**227.94**

(C)1994-2024 China Academic Journal Electronic Publishing House. All rights reserved. http://www.cnki.net

续表(2002)

		汽油<br>Gasoline<br>万吨<br>10^4tn	煤油<br>Kerosene<br>万吨<br>10^4tn
**一、可供本地区消费的能源量**	**Total Primary Energy Supply**	**-571.08**	**88.67**
1.一次能源生产量	Indigenous Production		
水　电	Hydro Power		
核　电	Nuclear Power		
2.回 收 能	Recovery of Energy		
3.进 口 量	Import		214.53
4.我轮、机在外国加油量	China Airplanes&Ships Refueling in Abroad		109.80
5.出 口 量(-)	Export (-)	-612.20	-169.64
6.外轮、机在我国加油量(-)	Foreign Airplanes&ships Refueling in China	-18.21	-71.30
7.库存增(-)、减(+)量	Stock Change	59.33	5.28
**二、加工转换投入(-)产出(+)量**	**Input(-) & Output(+) of Transformation**	**4320.02**	**826.11**
1.火力发电	Thermal Power	-0.62	
2.供　热	Heating Supply	-0.12	
3.洗 选 煤	Coal Washing		
4.炼　焦	Coking		
5.炼　油	Petroleum Refineries	4320.76	826.11
6.制　气	Gas Works		
#焦炭再投入量(-)	Coke Input (-)		
7.煤制品加工	Briquettes		
**三、损 失 量**	**Loss**		
**四、终端消费量**	**Total Final Consumption**	**3748.96**	**919.20**
1.农、林、牧、渔、水利业	Farming, Forestry, Animal Husbandry, Fishery & Water Conservancy	187.93	1.40
2.工　业	Industry	630.90	87.35
#用作原料、材料	Non-Energy Use	9.00	2.63
3.建 筑 业	Construction	122.32	
4.交通运输、仓储及邮电通迅业	Transport, Storage, Postal & Telecommunications Services	1503.50	616.75
5.批发和零售贸易业、餐饮业	Wholesale, Retail Trade and Catering Service	224.22	13.00
6.生活消费	Residential Consumption	163.80	60.70
城　镇	Urban	119.90	6.32
乡　村	Rural	43.90	54.38
7.其　他	Other	916.29	140.00
**五、平衡差额**	**Statistical Difference**	**-0.02**	**-4.42**

(C)1994-2024 China Academic Journal Electronic Publishing House. All rights reserved.   http://www.cnki.net

Continued(2002)

柴油<br>Diesel Oil<br>万吨<br>10^4tn	燃料油<br>Fuel Oil<br>万吨<br>10^4tn	液化石油气<br>PLG<br>万吨<br>10^4tn	炼厂干气<br>Refinery Gas<br>万吨<br>10^4tn	其他石油制品<br>Other Petroleum Products<br>万吨<br>10^4tn	天然气<br>Natural Gas<br>亿立方米<br>10^8 cu. m	热力<br>Heat<br>万百万千焦<br>10^{10} kj	电力<br>Electricity<br>亿千瓦小时<br>10^8kwh	其他能源<br>Other Energy<br>万吨标煤<br>10^4tce
**16.23**	**1857.30**	**626.82**		**138.16**	**326.61**		**3056.97**	**644.35**
					326.61		3131.01	
							2879.74	
							251.27	
								644.35
47.72	1659.66	626.16		384.32			23.00	
31.00	255.51							
-123.69	-63.91	-5.62		-246.16			-97.04	
-21.00	-41.00							
82.20	47.04	6.28						
**7480.11**	**647.27**	**1035.25**	**562.43**	**4193.36**	**-28.07**	**164190.77**	**13273.77**	**-333.45**
-225.99	-912.66	-1.54	-20.30	-36.13	-11.05		13273.77	-167.16
	-266.66		-113.16	-28.00	-17.02	164190.77		-90.60
7706.10	1845.50	1036.79	695.89	4257.49				-75.69
	-18.91							
		**1.76**			**6.33**	**2005.84**	**1168.66**	
**7441.16**	**2675.64**	**1615.09**	**570.00**	**4331.52**	**257.44**	**162179.59**	**15162.79**	**310.80**
1484.31	0.41					65.83	776.23	
1505.34	1752.63	318.77	570.00	4331.52	197.94	130282.41	10624.50	310.80
26.00	115.87	15.00	44.75	4331.52	60.00			310.80
251.99	19.10	12.76			0.68	181.67	164.14	
2964.81	872.10	20.00			1.56	810.73	338.00	
280.79	12.30	62.59			6.10	1466.42	500.00	
83.92		1169.05			51.16	26613.07	2001.42	
59.49		1006.49			51.16	26613.07	1168.94	
24.43		162.56					832.48	
870.00	19.10	31.92				2759.46	758.50	
**55.18**	**-171.07**	**45.22**	**-7.57**		**34.77**	**5.34**	**-0.71**	**0.10**

(C)1994-2024 China Academic Journal Electronic Publishing House. All rights reserved. http://www.cnki.net

4－4 综合能源平衡表

单位：万吨标准煤

项　目	Item	1980	1985
**可供消费的能源总量**	**Total Energy Available for Consumption**	**61557**	**77603**
一次能源生产量	Primary Energy Output	63735	85546
回收能	Recovery of Energy		
进口量	Imports	261	340
出口量(－)	Exports (－)	3058	5774
年初年末库存差额	Stock Changes in the Year	619	－2509
**能源消费总量**	**Total Energy Consumption**	**60275**	**76682**
在总量中：	Consumption by Sector		
1.农、林、牧、渔、水利业	Farming, Forestry, Animal Husbandry, Fishery and Water Conservancy	4692	4045
2.工　业	Industry	38986	51068
3.建筑业	Construction	957	1302
4.交通运输和邮电通讯业	Transportation, Post and Telecommunications Services	2902	3713
5.商业、饮食、物资供销和仓储业	Commerce, Catering Services, Materials Supply, Marketing and Storage	518	766
6.其他	Others	1205	2470
7.生活消费	Residential Consumption	11015	13318
在总量中：	Consumption by Usage		
(一) 终端消费	(I) Final Consumption	57508	73586
＃工业	Industry	38293	48021
(二) 加工转换损失量	(II) Losses in Processing and Transformation	1358	1491
＃炼焦	Coking	644	572
炼油	Petroleum Refining	113	110
(三) 损失量	(III) Other Losses	1409	1605
**平衡差额**	**Balance**	**1282**	**921**

注：1.村办工业包括在工业中(下同)。

2.电力、热力按等价热值折算，因此加工转换损失量中不包括发电、供热损失量。

3.进口量包括我国飞机、轮船在国外加油量；出口量包括外国飞机、轮船在我国加油量。

(C)1994-2024 China Academic Journal Electronic Publishing House. All rights reserved. http://www.cnki.net

OVERALL ENERGY BALANCE

(10 000 tce)

1990	1995	1996	1997	1998	1999	2000	2001	2002
**96138**	**129535**	**134433**	**133724**	**128368.34**	**115829**	**115150**	**125310**	**144319**
103922	129034	132616	132410	124249.57	109126	106988	120900	138369
	2312	1891	467	1919.72	1694	1760	1859	1908
1310	5456	6837	9964	8474.15	9513	14331	13471	15769
5875	6776	7529	7663	7153.44	6477	9026	11145	11017
-3219	-491	618	-1453	878.34	1974	1097	225	-710
**98703**	**131176**	**138948**	**138173**	**132213.92**	**130119**	**130297**	**134915**	**148222**
4852	5505	5717	5905	5790.32	5832	5787	6233	6514
67578	96191	100322	100080	94409.15	90797	89634	92347	102181
1213	1335	1449	1179	1612.09	1381	1433	1453	1610
4541	5863	5994	7543	8245.02	9243	9917	10257	11086
1247	2018	2268	2394	2552.08	2812	2893	3165	3464
3473	4519	5484	4703	5212.56	5502	5722	6034	6333
15799	15745	17714	16368	14392.7	14552	14912	15427	17032
94289	124252	132731	130585	126038.66	124096	124032	128951	140847
63239	89473	94342	92749	88521.91	85095	83707	86711	95143
2264	3634	2903	3915	2629.15	2336	2371	2011	2612
905		919	1297	684.24	544	487	387	322
326		549	626	616.19	648	781	636	1015
2150	3289	3314	3672	3546.11	3688	3893	3953	4763
**-2565**	**-1641**	**-4515**	**-4449**	**-3845.58**	**-14290**	**-15147**	**-9605**	**-3903**

a) Data on industry include the data of village-run industry. (The sane as in the following tables).

b) Electric power and heat are converted on the basic of equal caloric value. Therefore, losses in processing and transformation exclude losses in power generation and heating.

c) Data on imports include the petroleum consumed by the Chinese airplanes and ships in refueling abroad. Data on exports include the petroleum consumed by the foreign airplanes and ships in refueling in China.

(C)1994-2024 China Academic Journal Electronic Publishing House. All rights reserved. http://www.cnki.net

4－5 煤炭平衡表

单位：万吨

项　目	Item	1980	1985
**可供量**	**Total Energy Available for Consumption**	**62601.0**	**82776.6**
生产量	Output	62015.0	87228.4
进口量	Imports	199.0	230.7
出口量（－）	Exports（－）	632.0	777.0
年初年末库存差额	Stock Changes in the Year	1019.0	－3905.5
**消费量**	**Total Energy Consumption**	**61009.5**	**81603.0**
在消费量中：	Consumption by Sector		
1．农、林、牧、渔、水利业	Farming, Forestry, Animal Husbandry, Fishery and Water Conservancy	1550.3	2208.6
2．工　业	Industry	43848.4	58613.3
3．建筑业	Construction	556.0	531.9
4．交通运输和邮电通讯业	Transportation, Postal and Telecommunications Services	1934.4	2307.1
5．商业、饮食、物资供销和仓储业	Commerce, Catering Services, Materi－als Supply, Marketing and Storage	455.2	738.2
6．其他	Other	1091.2	1579.5
7．生活消费	Residential Consumption	11574.0	15624.4
在消费量中：	Consumption by Usage		
（一）终端消费	(1)Final Consumption	38804.2	52704.4
#工业	Industry	21643.1	29715.0
（二）中间消费	(2)Intermediate Consumption		
（用于加工转换）	(Consumed in Transformation)	19461.6	25397.4
发　电	Power Generation	12648.4	16440.7
供　热	Heating		1462.3
炼　焦	Coking	6682.2	7303.8
制　气	Gas Production	131.0	190.6
（三）洗选损耗	(3) Losses in Coal Washing and Dressing	2743.7	3501.2
**平衡差额**	**Balance**	**1591.5**	**1173.6**

注：生产量为原煤产量。

(C)1994-2024 China Academic Journal Electronic Publishing House. All rights reserved. http://www.cnki.net

COAL BALANCE SHEET

(10 000 tons)

1990	1995	1996	1997	1998	1999	2000	2001	2002
**102221.0**	**133461.7**	**137211.9**	**133159.0**	**122810.6**	**103576.1**	**98176.1**	**108480.0**	**129604.8**
107988.3	136073.1	139669.9	137282.0	125000.0	104500.0	99800.0	116078.0	138000.0
200.3	163.5	321.7	201.0	158.6	167.3	217.9	266.0	1125.7
1729.0	2861.7	3648.4	3073.0	3229.7	3743.9	5506.5	9012.9	8389.6
-4238.5	86.8	868.7	-1251.0	881.7	2652.7	3664.7	1148.9	-1131.4
**105523.0**	**137676.5**	**144734.4**	**139248.0**	**129492.2**	**126365.3**	**124537.4**	**126211.3**	**136605.5**
2095.2	1856.7	1917.3	1927.0	1923.3	1735.6	1647.7	1599.6	1622.9
81090.9	117570.7	123885.9	121671.0	114952.4	112757.3	111730.0	113608.0	124195.4
437.6	439.8	446.4	383.0	611.6	522.5	536.8	538.0	553.5
2160.9	1315.1	1175.9	1431.0	1390.6	1294.3	1139.9	11050.9	1055.0
1058.3	977.4	1074.3	863.0	947.6	896.2	814.6	809.9	809.1
1980.4	1986.7	1835.3	735.0	782.7	751.1	761.0	774.7	767.1
16699.7	13530.1	14399.3	12238.0	8884.0	8408.4	7907.0	7830.3	7602.6
60205.9	66156.1	68453.5	61792.0	56347.1	51572.2	46086.8	43891.3	42692.4
35773.8	46050.3	67604.9	44214.0	41807.3	37964.2	33279.7	31287.9	30282.2
41257.8	69487.6	74212.1	77451.0	73145.1	74793.1	78450.6	82320.1	93913.1
27204.3	44440.2	48808.6	48979.0	49489.3	51163.5	54611.2	57687.9	65600.0
2995.5	5887.3	6365.7	6245.0	6319.9	6473.0	6692.1	6961.5	7473.7
10697.6	18396.4	18455.8	19297.0	15628.1	14941.7	15000.4	15436.4	18209.7
360.4	763.7	582.0	733.0	685.1	847.6	810.0	893.8	973.2
4059.3	2032.8	2068.8	2311.0	1159.3	1491.5	1441.2	1450.5	1717.5
**-3302.0**	**-4214.8**	**-7522.5**	**-6089.0**	**-6681.6**	**-22789.2**	**-26361.3**	**-17731.3**	**-7000.8**

a) Data on output refer to the output of raw coal.

(C)1994-2024 China Academic Journal Electronic Publishing House. All rights reserved. http://www.cnki.net

4－6 焦炭平衡表

单位：万吨

项　　目	Item	1980	1985
**可供量**	**Total Energy Available for Consumption**	**4315.3**	**4689.7**
生产量	Output	4343.0	4802.1
进口量	Imports		2.1
出口量(－)	Exports (－)	27.1	36.9
年初年末库存差额	Stock Changes in the Year	－0.6	－77.6
**消费量**	**Total Energy Consumption**	**4303.0**	**4689.7**
在消费量中：	Consumption by Sector		
1. 农、林、牧、渔、水利业	Farming, Forestry, Animal Husbandry, Fishery and Water Conservancy	10.6	20.8
2. 工　业	Industry	4266.7	4627.7
3. 建筑业	Construction	11.9	7.8
4. 交通运输和邮电通讯业	Transportation, Postal and Telecommunications Services	8.2	5.7
5. 商业、饮食、物资供销和仓储业	Commerce, Catering Services, Materials Supply, Marketing and Storage	0.9	2.7
6. 其他	Other	4.7	2.0
7. 生活消费	Residential Consumption		23.0
在消费量中：	Consumption by Usage		
(一) 终端消费	(1) Final Consumption	4294.7	4677.9
＃工业	Industry	4258.4	4615.9
(二) 中间消费	(2) Intermediate Consumption		
(用于加工转换)	(Consumed in Transformation)	8.3	11.8
制气	Gas Production	8.3	11.8
(三) 损失量	(3) Losses in Coal Washing and Dressing		
**平衡差额**	**Balance**	**12.3**	

(C)1994-2024 China Academic Journal Electronic Publishing House. All rights reserved.　http://www.cnki.net

COKE BALANCE SHEET

(10 000 tons)

1990	1995	1996	1997	1998	1999	2000	2001	2002
**7085.8**	**12207.1**	**12726.8**	**12631.2**	**11733.5**	**10970.6**	**10892.3**	**11462.9**	**12830.5**
7328.3	13424.5	13595.6	13653.1	12899.1	12073.7	12184.0	13130.8	14253.3
	0.1	0.1	0.1					
129.0	886.1	768.6	1058.1	1146.4	997.4	1519.7	1384.6	1357.0
-113.5	-331.4	-100.3	36.1	-19.2	-105.7	228.0	-283.2	-65.8
**6914.7**	**10725.3**	**10797.9**	**10927.0**	**11078.2**	**10460.5**	**10440.0**	**10999.2**	**12343.9**
60.1	128.6	119.8	144.7	151.4	145.8	144.2	139.2	141.0
6808.8	10412.0	10491.1	10584.3	10710.3	10100.9	10080.5	10638.4	11978.1
5.2	10.8	13.9	12.5	14.6	17.1	19.0	23.9	23.4
4.1	10.1	6.6	6.5	10.3	10.1	11.2	11.7	11.4
7.7	25.7	28.3	35.2	38.8	36.5	35.7	39.7	42.6
1.9	6.4	12.0	19.2	12.8	13.0	12.2	12.1	12.3
26.9	131.6	126.2	124.7	140.0	143.1	137.2	134.2	135.1
6846.3	10648.0	10750.4	10848.9	11008.6	10302.7	10297.1	10845.6	12206.6
6740.4	10334.7	10443.5	10506.2	10640.6	9937.1	9937.7	10484.8	11840.7
68.4	77.3	47.6	78.1	69.7	157.8	142.9	153.7	137.4
68.4	77.3	47.6	78.1	69.7	157.8	142.9	153.7	137.4
**171.1**	**1481.8**	**1928.9**	**1704.2**	**655.2**	**510.1**	**452.3**	**463.7**	**486.6**

(C)1994-2024 China Academic Journal Electronic Publishing House. All rights reserved.   http://www.cnki.net

4－7 石油平衡表

单位：万吨

项　　目	Item	1980	1985
**可供量**	**Total Energy Available for Consumption**	**8794.5**	**9193.7**
生产量	Output	10594.6	12489.5
进口量	Imports	82.7	90.0
出口量(－)	Exports (－)	1806.2	3630.4
年初年末库存差额	Stock Changes in the Year	－76.6	244.6
**消费量**	**Total Energy Consumption**	**8757.4**	**9168.8**
在消费量中：	Consumption by Sector		
1. 农、林、牧、渔、水利业	Farming, Forestry, Animal Husbandry, Fishery and Water Conservancy	814..9	758.7
2. 工　业	Industry	6203.2	6171.4
3. 建筑业	Construction	175.2	292.2
4. 交通运输和邮电通讯业	Transportation, Postal and Telecommunications Services	911.5	1176.4
5. 商业、饮食、物资供销和仓储业	Commerce, Catering Services, Materi－als Supply, Marketing and Storage	29.0	38.1
6. 其他	Other	481.7	506.1
7. 生活消费	Residential Consumption	141.9	225.9
在消费量中：	Consumption by Usage		
(一) 终端消费	(I)Final Consumption	6311.0	7063.3
#工　业	Industry	3780.3	4462.0
(二) 中间消费	(II)Intermediate Consumption		
(用于加工转换)	(Consumed in Transformation)	2102.1	1745.6
发　电	Power Generation	2065.4	1425.5
供　热	Heating		285.6
制　气	Gas Production	36.7	34.5
(三) 炼油损失量	(III)Losses in Petroleum Refining	81.5	112.9
(四) 损失量	Other Losses	262.8	247.0
**平衡差额**	**Balance**	**37.1**	**24.9**

注：1.生产量为原油产量。

2.进口量包括我国飞机、轮船在国外加油量；出口量包括外国飞机、轮船在我国加油量。

(C)1994-2024 China Academic Journal Electronic Publishing House. All rights reserved.　http://www.cnki.net

PETROLEUM BALANCE

(10 000 tons)

1990	1995	1996	1997	1998	1999	2000	2001	2002
**11435.0**	**16072.7**	**17656.1**	**19653.8**	**19686.1**	**20964.4**	**22631.8**	**23204.7**	**24925.1**
13830.6	15005.0	15733.4	16074.1	16100.0	16000.0	16300.0	16395.9	16700.0
755.6	3673.2	4536.9	6787.0	5738.7	6483.3	9748.5	9118.2	10269.3
3110.4	2454.5	2696.0	2815.2	2326.5	1643.5	2172.1	2046.7	2139.2
-40.8	-151.0	81.8	-392.2	174.0	124.6	-1244.6	-262.7	94.9
**11485.6**	**16064.9**	**17436.2**	**19691.7**	**19817.8**	**21072.9**	**22439.3**	**22838.3**	**24779.8**
1033.6	1203.2	1223.7	1256.3	1294.7	1422.1	1496.9	1568.5	1674.1
7321.6	9349.3	10020.2	11304.2	10870.8	10852.8	11404.8	11388.6	12489.6
327.3	242.8	257.8	285.3	293.9	323.0	344.3	372.3	410.4
1683.2	2863.6	2937.7	3733.0	4245.3	5004.3	5509.5	5692.9	6156.7
77.6	333.9	367.9	416.6	426.0	537.2	545.0	567.4	593.0
757.8	1390.3	1752.3	1758.0	1704.8	1800.3	1882.9	1953.7	1978.6
284.5	682.0	876.6	938.3	983.3	1133.1	1256.5	1294.8	1477.5
9304.7	13676.3	15302.3	17051.9	17514.3	18664.7	19893.5	20357.0	21982.8
5180.4	7095.5	8022.5	8814.1	8717.3	8596.4	9016.2	9059.9	9854.5
1630.4	2230.0	1701.4	2449.7	2106.1	2222.2	2352.9	2292.0	2606.8
1234.4	1358.5	1238.6	1662.1	1304.8	1228.6	1178.2	1213.6	1275.6
356.3	399.9	404.8	356.5	455.5	394.6	427.0	438.7	420.7
39.7	51.6	58.2	49.2	35.4	32.5	25.9	22.8	18.9
295.8	420.1	271.1	382.0	310.4	566.6	721.9	617.0	891.6
254.7	158.6	161.4	190.1	197.3	186.0	192.9	189.3	190.2
**-50.6**	**7.8**	**219.9**	**-37.9**	**-131.5**	**-108.5**	**192.5**	**366.4**	**145.3**

a) Data on output refer to the output of crude oil.

b) Data on imports include the petroleum consumed by the Chinese airplanes and ships in refueling abroad. Data on exports include the petroleum consumed by the foreign airplanes and ships in refueling in China.

(C)1994-2024 China Academic Journal Electronic Publishing House. All rights reserved. http://www.cnki.net

4－8 原油平衡表

单位：万吨

项　　目	Item	1980	1985
**可供量**	**Total Energy Available for Consumption**	**9222.9**	**9516.5**
生产量	Output	10594.6	12489.5
进口量	Imports	36.6	
出口量(－)	Exports (－)	1330.9	3003.0
年初年末库存差额	Stock Changes in the Year	－77.4	30.0
**消费量**	**Total Energy Consumption**	**9205.0**	**9509.5**
在消费量中：	Consumption by Sector		
1. 农、林、牧、渔、水利业	Farming, Forestry, Animal Husbandry, Fishery and Water Conservancy	8.0	0.8
2. 工　业	Industry	9112.0	9389.9
3. 建筑业	Construction	28.8	74.0
4. 交通运输和邮电通讯业	Transportation, Postal and Telecommunications Services	50.1	44.3
5. 商业、饮食、物资供销和仓储业	Commerce, Catering Services, Materi－als Supply, Marketing and Storage		0.1
6. 其他	Other	6.1	0.4
7. 生活消费	Residential Consumption		
在消费量中：	Consumption by Usage		
(一) 终端消费	(I) Final Consumption	499.6	350.4
＃工业	Industry	429.7	254.9
(二) 中间消费	(II) Intermediate Consumption		
(用于加工转换)	(Consumed in Transformation)	8443.0	8929.7
发　电	Power Generation	574.0	279.5
供　热	Heating		61.3
炼　油	Petroleum Refineries	7869.0	8588.9
(三) 油田原油损失量	(III) Losses in Oil Field for Crude Oil	262.4	229.4
**平衡差额**	**Balance**	**17.9**	**7.0**

(C)1994-2024 China Academic Journal Electronic Publishing House. All rights reserved. http://www.cnki.net

CRUDE OIL BALANC

(10 000 tons)

1990	1995	1996	1997	1998	1999	2000	2001	2002
**11770.6**	**14794.9**	**15920.7**	**17499.6**	**17316.9**	**18947.2**	**21383.0**	**21537.2**	**22769.0**
13830.6	15004.4	15733.4	16074.1	16100.0	16000.0	16300.0	16395.9	16700.0
292.3	1709.0	2261.7	3547.0	2732.0	3661.4	7026.5	6026.0	6940.6
2399.0	1822.7	2040.3	1982.9	1560.0	716.7	1030.6	755.0	766.5
46.7	−95.8	−34.1	−138.6	44.9	2.6	−912.9	−129.7	−105.2
**11762.2**	**14886.4**	**15865.0**	**17367.2**	**17395.3**	**18949.5**	**21232.0**	**21342.7**	**22541.1**
0.2	10.1	11.0						
11653.8	14716.3	15690.9	17197.8	17222.5	18775.2	21052.1	21168.2	22357.5
55.2	2.7	2.6	3.0	2.2	3.2	3.3	3.4	4.2
52.1	156.8	159.0	164.7	168.9	169.5	175.0	169.8	177.9
0.3	0.5	0.4	0.3	0.2	0.2	0.2	0.2	0.1
0.6	1390.3	1.2	1.4	1.5	1.4	1.4	1.2	1.3
402.1	309.9	492.2	492.4	518.7	519.4	636.8	654.1	681.3
333.4	274.7	452.9	471.0	495.4	495.4	612.3	630.8	658.0
11106.9	14419.4	15212.9	16686.4	16680.7	18245.7	20404.3	20500.7	21671.3
124.6	61.6	68.2	64.3	74.4	80.2	85.0	81.6	78.3
21.1	4.4	10.8	14.9	24.5	12.9	14.0	12.3	12.8
10961.2	14353.4	15133.9	16607.3	16581.8	18152.6	20305.3	20406.8	21580.2
253.2	157.1	159.9	188.4	196.0	184.4	190.9	187.9	188.5
**8.4**	**−91.5**	**55.7**	**132.4**	**−78.4**	**−2.2**	**151.0**	**194.4**	**227.9**

(C)1994-2024 China Academic Journal Electronic Publishing House. All rights reserved. http://www.cnki.net

4－9 燃料油平衡表

单位：万吨

项　目	Item	1980	1985
**可供量**	**Total Energy Available for Consumption**	**3096.1**	**2848.0**
生产量	Output	3142.0	2835.8
进口量	Imports	39.0	70.0
出口量(－)	Exports (－)	45.4	64.9
年初年末库存差额	Stock Changes in the Year	－39.5	7.1
**消费量**	**Total Energy Consumption**	**3073.7**	**2837.4**
在消费量中：	Consumption by Sector		
1. 农、林、牧、渔、水利业	Farming, Forestry, Animal Husbandry, Fishery and Water Conservancy	2.3	3.1
2. 工　业	Industry	2937.4	2662.2
3. 建筑业	Construction	15.0	18.9
4. 交通运输和邮电通讯业	Transportation, Postal and Telecommunications Services	109.0	144.1
5. 商业、饮食、物资供销和仓储业	Commerce, Catering Services, Materi－als Supply, Marketing and Storage	2.9	3.1
6. 其他	Other	7.1	6.0
7. 生活消费	Residential Consumption		
在消费量中：	Consumption by Usage		
(一) 终端消费	(I) Final Consumption	1617.9	1538.8
#工业	Industry	1481.6	1363.5
(二) 中间消费	(II) Intermediate Consumption		
(用于加工转换)	(Consumed in Transformation)	1455.8	1296.1
发　电	Power Generation	1419.1	1042.3
供　热	Heating		219.3
制　气	Gas Production	36.7	34.5
(三) 损失量	(III) Other Losses		2.5
**平衡差额**	**Balance**	**22.4**	**10.6**

(C)1994-2024 China Academic Journal Electronic Publishing House. All rights reserved. http://www.cnki.net

FUEL OIL BALANCE

(10 000 tons)

1990	1995	1996	1997	1998	1999	2000	2001	2002
**3320.7**	**3717.3**	**3632.3**	**3721.1**	**3828.7**	**3901.1**	**3836.7**	**3836.9**	**3702.8**
3267.9	2960.8	2504.5	2311.2	2100.4	1959.4	2053.7	1864.4	1845.5
167.3	859.1	1192.6	1506.8	1818.3	1963.3	1704.3	2048.7	1915.2
97.2	68.6	70.8	91.2	72.9	38.8	57.9	84.9	104.9
-17.3	-34.0	6.0	-5.7	-17.2	17.2	136.6	8.7	47.0
**3367.8**	**3693.7**	**3564.5**	**3821.3**	**3828.6**	**3934.1**	**3872.8**	**3850.2**	**3873.9**
2.9	8.4	2.5	2.9	0.3	0.4	0.4	0.4	0.4
3091.7	3406.2	3305.5	3223.0	3217.3	3047.7	2975.1	2949.3	2950.9
47.3	14.2	14.0	19.2	16.6	16.2	16.7	16.2	19.0
208.2	227.5	224.5	582.2	565.6	840.0	850.0	855.0	872.1
1.6	6.6	4.3	6.2	7.4	10.5	11.6	12.3	12.3
16.1	30.8	14.5	14.7	21.4	19.4	19.0	17.0	19.1
2042.6	2262.8	2320.6	2651.1	2456.4	2694.0	2741.4	2689.3	2675.6
1766.5	1975.3	1961.8	2025.8	1845.1	1807.6	1843.7	1788.4	1752.6
1325.2	1430.9	1243.9	1170.3	1372.2	1240.1	1131.3	1160.9	1198.2
977.3	1071.5	888.8	839.2	993.8	906.3	814.2	838.5	912.7
308.3	307.8	297.1	282.0	343.0	301.3	291.2	299.6	266.7
39.6	51.6	58.1	49.2	35.4	32.5	25.9	22.8	18.9
**-47.1**	**23.6**	**67.8**	**-100.2**	**0.1**	**-33.0**	**-36.1**	**-13.4**	**-171.1**

(C)1994-2024 China Academic Journal Electronic Publishing House. All rights reserved. http://www.cnki.net

4-10 汽油平衡表

单位：万吨

项 目	Item	1980	1985
**可供量**	**Total Energy Available for Consumption**	**999.4**	**1399.6**
生产量	Output	1079.0	1471.9
进口量	Imports		0.3
出口量(-)	Exports (-)	117.8	129.9
年初年末库存差额	Stock Changes in the Year	38.2	57.3
**消费量**	**Total Energy Consumption**	**998.6**	**1396.3**
在消费量中：	Consumption by Sector		
1. 农、林、牧、渔、水利业	Farming, Forestry, Animal Husbandry, Fishery and Water Conservancy	53.3	122.3
2. 工 业	Industry	273.2	451.3
3. 建筑业	Construction	54.1	73.0
4. 交通运输和邮电通讯业	Transportation, Postal and Telecommunications Services	404.9	477.4
5. 商业、饮食、物资供销和仓储业	Commerce, Catering Services, Materi-als Supply, Marketing and Storage	19.4	23.4
6. 其他	Other	193.7	238.3
7. 生活消费	Residential Consumption		10.6
**平衡差额**	**Balance**	**0.8**	**3.3**

4-11 煤油平衡表

单位：万吨

项 目	Item	1980	1985
**可供量**	**Total Energy Available for Consumption**	**359.0**	**383.2**
生产量	Output	398.5	405.3
进口量	Imports		15.2
出口量(-)	Exports (-)	46.8	46.0
年初年末库存差额	Stock Changes in the Year	2.3	8.7
**消费量**	**Total Energy Consumption**	**365.9**	**385.5**
在消费量中：	Consumption by Sector		
1. 农、林、牧、渔、水利业	Farming, Forestry, Animal Husbandry, Fishery and Water Conservancy	2.3	3.3
2. 工 业	Industry	15.7	20.1
3. 建筑业	Construction	0.8	1.3
4. 交通运输和邮电通讯业	Transportation, Postal and Telecommunications Services	31.4	56.2
5. 商业、饮食、物资供销和仓储业	Commerce, Catering Services, Materi-als Supply, Marketing and Storage	0.2	0.1
6. 其他	Other	216.7	182.9
7. 生活消费	Residential Consumption	98.8	121.6
**平衡差额**	**Balance**	**-6.9**	**-2.3**

(C)1994-2024 China Academic Journal Electronic Publishing House. All rights reserved. http://www.cnki.net

GASOLINE BALANCE

(10 000 tons)

1990	1995	1996	1997	1998	1999	2000	2001	2002
**1884.1**	**2902.0**	**2172.3**	**3284.0**	**3307.6**	**3379.8**	**3504.5**	**3606.9**	**3749.7**
2173.4	3051.6	3274.6	3517.8	3501.0	3741.3	4134.7	4154.7	4320.8
16.9	15.9	7.9	8.4	1.5	0.0	0.0	0.0	0.0
233.8	193.1	139.5	185.9	194.5	425.8	467.7	586.0	630.4
-72.4	27.6	29.3	-56.4	-0.5	64.3	-162.5	38.1	59.3
**1899.5**	**2909.6**	**3182.4**	**3312.0**	**3328.6**	**3380.7**	**3504.9**	**3597.8**	**3749.7**
145.9	179.7	180.2	176.4	172.6	178.1	184.5	190.6	187.9
589.3	812.4	895.7	723.1	677.5	646.5	602.0	618.1	631.6
89.5	103.6	106.2	107.7	112.6	113.8	115.6	116.7	122.3
620.1	982.3	991.3	1183.2	1216.6	1265.5	1387.8	1419.4	1503.5
46.0	197.2	195.8	211.7	216.5	206.3	209.8	214.0	224.2
390.7	570.7	726.8	815.8	825.7	849.4	877.7	904.3	916.3
18.0	63.7	86.4	94.2	107.1	121.1	127.6	134.6	163.8
**-15.4**	**-7.6**	**-10.1**	**-28.0**	**-21.1**	**-0.9**	**-0.4**	**9.1**	**0.0**

KEROSENE BALANCE

(10 000 tons)

1990	1995	1996	1997	1998	1999	2000	2001	2002
**350.9**	**486.4**	**542.1**	**681.7**	**699.9**	**848.6**	**880.9**	**894.2**	**914.8**
392.5	445.8	538.4	577.0	616.1	743.8	872.3	789.4	826.1
26.1	115.7	101.1	218.1	188.0	272.2	322.5	298.6	324.3
55.5	62.4	102.4	98.8	127.6	162.8	256.3	246.4	240.9
-12.2	-12.7	5.0	-14.6	23.3	-4.6	-57.6	52.7	5.3
**350.9**	**512.1**	**555.5**	**681.7**	**671.4**	**824.2**	**870.1**	**890.3**	**919.2**
3.1	3.6	1.7	1.4	1.6	1.4	1.5	1.5	1.4
20.6	44.9	43.1	46.7	62.2	78.4	83.9	86.0	87.4
1.3	3.5	5.0	4.2	3.5	3.9	4.0	3.5	0.0
93.4	250.0	298.9	420.1	390.5	505.6	536.4	560.7	616.7
0.6	8.5	8.3	9.0	9.0	11.5	12.0	12.5	13.0
127.3	137.3	133.9	137.7	141.6	152.7	160.1	151.1	140.0
104.6	64.3	64.7	62.7	63.1	70.8	72.2	75.0	60.7
	**-25.7**	**-13.4**		**28.5**	**24.3**	**11.3**	**4.0**	**-4.4**

(C)1994-2024 China Academic Journal Electronic Publishing House. All rights reserved. http://www.cnki.net

4－12 柴油平衡表

单位：万吨

项 目	Item	1980	1985
**可供量**	**Total Energy Available for Consumption**	**1663.2**	**1944.1**
生产量	Output	1827.8	2023.2
进口量	Imports	2.1	4.5
出口量(－)	Exports (－)	166.5	225.6
年初年末库存差额	Stock Changes in the Year	－0.2	142.0
**消费量**	**Total Energy Consumption**	**1663.2**	**1939.4**
在消费量中:	Consumption by Sector		
1. 农、林、牧、渔、水利业	Farming, Forestry, Animal Husbandry, Fishery and Water Conservancy	749.0	629.2
2. 工 业	Industry	457.4	644.1
3. 建筑业	Construction	76.5	125.0
4. 交通运输和邮电通讯业	Transportation, Postal and Telecommunications Services	316.1	454.4
5. 商业、饮食、物资供销和仓储业	Commerce, Catering Services, Materials Supply, Marketing and Storage	6.5	10.9
6. 其他	Other	57.7	74.0
7. 生活消费	Residential Consumption		
在消费量中:	Consumption by Usage		
(一) 终端消费	(I) Final Consumption	1590.9	1827.4
＃工业	Industry	385.1	532.1
(二) 中间消费	(II) Intermediate Consumption		
(用于加工转换)	(Consumed in Transformation)	72.3	108.6
发 电	Power Generation	72.3	103.6
供 热	Heating		5.0
(三) 损失量	Other Losses		3.4
**平衡差额**	**Balance**		**4.7**

(C)1994-2024 China Academic Journal Electronic Publishing House. All rights reserved. http://www.cnki.net

DIESEL OIL BALANCE

(10 000 tons)

1990	1995	1996	1997	1998	1999	2000	2001	2002
**2689.4**	**4404.2**	**4818.7**	**5271.2**	**5229.8**	**6204.3**	**6806.5**	**7272.0**	**7722.3**
2609.0	3972.6	4419.0	4924.5	4897.7	6172.6	7079.6	7485.6	7706.1
233.8	645.3	512.1	790.2	331.7	56.0	51.9	54.7	78.7
169.8	169.5	192.4	261.5	118.8	70.9	77.5	46.9	144.7
16.4	-44.2	80.0	-182.0	119.2	46.5	-247.6	-221.4	82.2
**2691.7**	**4321.4**	**4691.7**	**5291.2**	**5282.8**	**6231.6**	**6774.3**	**7107.7**	**7667.2**
881.5	1001.4	1028.4	1075.7	1120.2	1241.8	1310.1	1375.6	1484.3
728.1	1189.9	1357.2	1730.4	1346.1	1506.8	1596.5	1637.6	1732.1
133.0	118.2	129.6	146.0	153.5	178.1	195.9	223.1	252.0
709.4	1246.6	1261.1	1379.5	1901.9	2221.7	2543.8	2671.0	2964.8
22.5	103.6	125.3	146.4	153.3	260.9	255.9	268.1	280.8
217.0	645.7	768.1	774.0	564.0	759.7	803.7	853.9	870.0
	16.1	22.1	39.2	43.9	62.7	68.4	79.2	83.9
2564.8	4070.0	4408.5	4549.2	5078.9	6016.2	6546.6	6867.3	7441.2
601.2	938.5	1074.1	988.4	1142.2	1291.4	1368.8	1396.4	1505.3
126.9	251.4	283.1	742.0	203.9	215.5	227.7	240.4	226.0
124.5	204.9	253.3	739.1	203.9	215.5	227.7	240.4	226.0
2.4	46.6	29.9	2.9					
**-2.3**	**82.7**	**127.0**	**-20.0**	**-52.9**	**-27.4**	**32.2**	**164.4**	**55.2**

(C)1994-2024 China Academic Journal Electronic Publishing House. All rights reserved. http://www.cnki.net

4－13 液化石油气平衡表

单位：万吨

项　　目	Item	1980	1985
**可供量**	**Total Energy Available for Consumption**	**122.5**	**157.3**
生产量	Output	122.5	159.7
进口量	Imports		
出口量(－)	Exports (－)		1.9
年初年末库存差额	Stock Changes in the Year		－0.5
**消费量**	**Total Energy Consumption**	**119.6**	**155.7**
在消费量中：	Consumption by Sector		
1. 农、林、牧、渔、水利业	Farming, Forestry, Animal Husbandry, Fishery and Water Conservancy		
2. 工　业	Industry	76.1	59.9
3. 建筑业	Construction		
4. 交通运输和邮电通讯业	Transportation, Postal and Telecommunications Services		
5. 商业、饮食、物资供销和仓储业	Commerce, Catering Services, Materials Supply, Marketing and Storage		0.5
6. 其他	Other	0.4	4.5
7. 生活消费	Residential Consumption	43.1	90.8
**平衡差额**	**Balance**	**2.9**	**1.6**

4－14 天然气平衡表

单位：万吨

项　　目	Item	1980	1985
**可供量**	**Total Energy Available for Consumption**	**142.7**	**129.3**
生产量	Output	142.7	129.3
进口量	Imports		
出口量(－)	Exports (－)		
年初年末库存差额	Stock Changes in the Year		
**消费量**	**Total Energy Consumption**	**140.6**	**129.3**
在消费量中：	Consumption by Sector		
1. 农、林、牧、渔、水利业	Farming, Forestry, Animal Husbandry, Fishery and Water Conservancy		
2. 工　业	Industry	131.4	109.6
3. 建筑业	Construction	6.0	14.1
4. 交通运输和邮电通讯业	Transportation, Postal and Telecommunications Services	0.7	0.8
5. 商业、饮食、物资供销和仓储业	Commerce, Catering Services, Materials Supply, Marketing and Storage		
6. 其他	Other	0.5	0.5
7. 生活消费	Residential Consumption	2.0	4.3
**平衡差额**	**Balance**	**2.1**	

(C)1994-2024 China Academic Journal Electronic Publishing House. All rights reserved.　http://www.cnki.net

LPG BALANCE

(10 000 tons)

1990	1995	1996	1997	1998	1999	2000	2001	2002
**258.5**	**774.3**	**923.1**	**992.1**	**1178.0**	**1146.6**	**1396.16**	**1428.03**	**1663.61**
261.6	540.8	605.9	667.9	747.4	816.6	916.62	952.33	1036.79
	232.6	355.0	358.2	476.6	322.3	481.74	488.86	626.16
1.1	7.1	33.3	39.2	50.2	7.5	1.6	2.09	5.62
-2.0	8.0	-4.5	5.1	4.2	-1.5	-0.6	-11.07	6.28
**254.2**	**750.6**	**931.3**	**1009.7**	**1186.0**	**1208.5**	**1366.67**	**1411.0**	**1618.39**
	0.1						0.3	
82.0	192.5	183.0	203.4	220.5	254.0	276.14	291.67	320.49
1.0	0.5	0.4	5.2	5.7	7.9	8.91	9.51	12.76
	0.5	1.6	1.6	0.5	2.0	16.45	17.03	21.58
6.6	17.4	33.9	42.9	39.5	47.9	55.48	60.4	62.59
6.1	5.7	8.8	14.5	150.6	17.9	21	26.21	31.92
158.5	534.0	703.5	742.2	769.2	878.5	988.34	1006.04	1169.05
**4.3**	**23.7**	**-8.2**	**-17.7**	**-8.0**	**-61.6**	**29.49**	**16.87**	**45.22**

NATURAL GAS BALANCE

(10^8 cu.m)

1990	1995	1996	1997	1998	1999	2000	2001	2002
**153.0**	**179.5**	**201.1**	**226.7**	**232.8**	**252.0**	**272.0**	**303.3**	**326.6**
153.0	179.5	201.1	227.0	232.8	252.0	272.0	303.3	326.6
			0.3					
**152.5**	**177.4**	**184.9**	**195.4**	**202.6**	**214.9**	**245.0**	**274.3**	**291.8**
		0.2						
120.2	154.4	157.2	168.9	171.5	180.2	202.0	217.8	227.5
10.6	0.3	1.5		0.1	0.7	0.8	0.7	0.7
1.9	1.6	4.0	3.7	3.7	4.8	5.8	6.0	6.4
	0.6	1.0	1.0	2.5	2.9	3.4	5.0	6.1
1.2	1.2	1.3	0.6	0.6	0.6	0.6	0.7	
18.6	19.4	19.7	21.2	24.1	25.7	32.3	44.1	51.2
**0.5**	**2.1**	**16.2**	**31.3**	**30.2**	**37.0**	**27.0**	**29.0**	**34.8**

(C)1994-2024 China Academic Journal Electronic Publishing House. All rights reserved. http://www.cnki.net

4－15 电力平衡表

单位：亿千瓦小时

项 目	Item	1980	1985
**可供量**	**Total Energy Available for Consumption**	**3006.3**	**4117.6**
生产量	Output	3006.3	4106.9
水 电	Hydropower	582.1	923.7
火 电	Thermal Power	2424.2	3183.2
核 电	Nuclear Power		
进口量	Imports		11.1
出口量(－)	Exports (－)		0.4
**消费量**	**Total Energy Consumption**	**3006.3**	**4117.6**
在消费量中：	Consumption by Sector		
1. 农、林、牧、渔、水利业	Farming, Forestry, Animal Husbandry, Fishery and Water Conservancy	270	317.4
2. 工 业	Industry	2471:9	3283.4
3. 建筑业	Construction	47.1	71.2
4. 交通运输和邮电通讯业	Transportation, Postal and Telecommunications Services	26.5	63.4
5. 商业、饮食、物资供销和仓储业	Commerce, Catering Services, Materi－als Supply, Marketing and Storage	16.8	38.0
6. 其他	Others	68.8	121.7
7. 生活消费	Residential Consumption	105.2	222.5
在消费量中：	Consumption by Usage		
(一)终端消费	(I)Final Consumption	2763.4	3813.3
#工业	Industry	2229.0	2979.1
(二)输配电损失量	(II)Losses in Transmission	242.9	304.3

(C)1994-2024 China Academic Journal Electronic Publishing House. All rights reserved. http://www.cnki.net

ELECTRICITY BALANCE

(100 million kwh)

1990	1995	1996	1997	1998	1999	2000	2001	2002
**6230.4**	**10023.4**	**10764.3**	**11273.6**	**11590.4**	**12305.2**	**13472.7**	**14632.6**	**16330.7**
6212.0	10077.3	10800.2	11344.7	11662.0	12393.0	13556.0	14716.6	16404.8
1267.2	1905.8	1879.7	1959.8	2080.0	2038.1	2224.1	2774.3	2879.7
4944.8	8043.2	8777.1	9240.7	9441.0	10205.4	11164.5	11767.5	13273.8
	128.3	143.4	144.2	141.0	149.5	167.4	174.7	251.3
19.3	6.4	1.2	0.9	0.2	3.7	15.5	18.0	23.0
0.9	60.3	37.1	72.0	71.7	91.5	98.8	101.9	97.0
**6230.4**	**10023.4**	**10764.3**	**11284.4**	**11598.4**	**12305.2**	**13471.4**	**14633.5**	**16331.5**
426.8	582.4	618.3	639.8	623.5	660.4	673.0	762.4	776.2
4873.3	7659.8	8044.7	8395.7	8406.0	8832.7	9653.6	10444.7	11793.2
65.0	159.6	181.8	117.4	188.8	142.3	154.8	144.9	164.1
105.9	182.3	197.9	255.9	255.6	254.8	281.2	309.3	338.0
76.2	199.5	223.1	265.1	293.4	342.8	393.7	444.9	500.0
202.4	234.2	365.4	357.4	506.7	591.4	643.2	688.1	758.5
480.8	1005.6	1133.1	1253.2	1324.5	1480.8	1672.0	1839.2	2001.4
5795.8	9278.9	9994.8	10486.0	10807.5	11443.3	12534.7	13600.0	15162.8
4438.7	6915.3	7275.2	7597.3	7615.0	7970.8	8716.9	9411.2	10624.5
434.6	744.5	769.5	798.4	790.9	861.9	936.7	1033.5	1168.7

(C)1994-2024 China Academic Journal Electronic Publishing House. All rights reserved. http://www.cnki.net

4-1 中国能源平衡表(标准量) -1999

单位:万吨标准煤

		能源合计 Energy Total	
		(发电煤耗计算法)(coal equivalent calculation)	(电热当量计算法)(calorific value calculation)
**一.可供本地区消费的能源量**	**Total Primary Energy Supply**	**132065.36**	**126628.10**
1.一次能源生产量	Indigenous Production	125934.78	120264.43
水电	Hydro Power	7634.59	2415.97
核电	Nuclear Power	580.58	183.72
2.回收能	Recovery of Energy	1693.52	1693.52
3.进口量	Import	9092.54	9082.82
4.我轮、机在外国加油量	China Airplanes&ships Refueling in Abroad	421.14	421.14
5.出口量(-)	Export (-)	-6943.05	-6700.25
6.外轮、机在我国加油量(-)	Foreign Airplanes&ships Refueling in China	-107.55	-107.55
7.库存增(-)、减(+)量	Stock Change	1973.98	1973.98
**二.加工转换投入(-)产出(+)量**	**Input(-) & Output(+) of Transformation**	**-2227.85**	**-31450.60**
1.火力发电	Thermal Power		-27229.44
2.供热	Heating Supply		-1993.32
3.洗选煤	Coal Washing	-767.05	-767.05
4.炼焦	Coking	-515.62	-515.62
5.炼油	Petroleum Refineries	-647.68	-647.68
6.制气	Gas Works	-105.12	-105.12
#焦炭再投入量(-)	Coke Input (-)	-153.32	-153.32
7.煤制品加工	Briquettes	-39.06	-39.06
**三.损失量**	**Loss**	**3789.44**	**1476.38**
**四.终端消费量**	**Total Final Consumption**	**127813.68**	**95466.10**
1.农、林、牧、渔、水利业	Farming, Forestry, Animal Husbandry, Fishery & Water Conservancy	5993.39	4239.55
2.工业	Industry	87151.21	64390.11
#用作原料、材料	Non-Energy Use	5789.89	5789.89
3.建筑业	Construction	1979.35	1599.52
4.交通运输、仓储及邮电通讯业	Transport, Storage, Postal & Telecommunications Services	9011.78	8325.87
5.批发和零售贸易业、餐饮业	Wholesale, Retail Trade and Catering Service	2901.48	1975.12
6.生活消费	Residential Consumption	15213.92	10984.48
城镇	Urban	8978.58	6392.75
乡村	Rural	6235.33	4591.73
7.其他	Other	5562.54	3951.45
**五.平衡差额**	**Statistical Difference**	**-1765.61**	**-1764.98**
**六.能源消费总量**	**Total Energy Consumption**	**133830.97**	**128393.08**

(C)1994-2024 China Academic Journal Electronic Publishing House. All rights reserved. http://www.cnki.net

ENERGY BALANCE OF CHINA -1999 (STANDARD QUANTITY)

(10 000 tce)

煤合计 Coal Total	原煤 Raw Coal	洗精煤 Cleaned Coal	其他洗煤 Other Washed Coal	型煤 Briquettes	焦炭 Coke	焦炉煤气 Coke Oven Gas	其他煤气 Other Gas	其他焦化产品 Other Coking Products	油品合计 Petroleum Products Total
**90557.06**	**90894.72**	**-379.31**	**47.50**	**-5.85**	**-1071.55**		**1080.73**	**-1.07**	**30059.65**
91430.40	91430.40								22857.60
							1080.67		28.95
124.70	100.72	23.97						31.85	8921.78
									421.14
-2893.18	-2414.30	-478.79		-0.09	-968.89			-32.92	-2243.71
									-107.55
1895.14	1777.90	75.50	47.50	-5.76	-102.66		0.06		181.44
**-58398.08**	**-61918.76**	**1051.26**	**1930.19**	**539.24**	**11575.11**	**1265.68**	**203.67**	**209.16**	**-2923.08**
-37559.30	-36862.69	-82.07	-614.54			-57.01	-188.74		-1765.23
-5658.85	-5573.56	-5.24	-80.05			-47.36	-96.97		-575.15
-768.44	-14460.36	11032.69	2659.23						
-13628.42	-4043.69	-9565.12	-19.60		11520.06	1354.63	42.15	194.86	
									-536.27
-744.01	-408.56	-328.99	-6.46		208.38	15.42	447.24	14.30	-46.43
					-153.32				
-39.06	-569.90		-8.40	539.24					
						**15.55**			**266.20**
**34268.68**	**30779.53**	**951.63**	**1963.50**	**574.02**	**10008.03**	**1290.49**	**1283.02**	**215.12**	**27017.26**
1209.73	1195.33		14.13	0.27	141.64				2074.74
25057.03	22579.16	944.02	1498.93	34.92	9652.86	1046.49	1036.73	215.12	11521.67
1612.20	1499.26	48.52	62.95	1.47	635.38	11.93		54.75	2533.90
364.48	354.44	3.95	6.09		16.61				1029.92
897.93	885.62	3.47	8.70	0.15	9.85	0.12	0.58		7071.82
624.42	612.99	0.09	5.38	5.96	35.48	11.04	8.40		797.93
5669.56	4738.06		400.94	530.55	138.99	213.82	234.18		1879.65
2285.71	1762.84		188.19	334.68	68.00	213.82	231.79		1506.06
3383.85	2975.22		212.75	195.88	70.99		2.39		373.59
445.53	413.93	0.10	29.33	2.16	12.61	19.02	3.13		2641.53
**-2109.70**	**-1803.57**	**-279.68**	**14.20**	**-40.64**	**495.53**	**-40.36**	**1.38**	**-7.03**	**-146.88**

(C)1994-2024 China Academic Journal Electronic Publishing House. All rights reserved. http://www.cnki.net

续表

单位:万吨标准煤

		原油 Crude Oil	汽油 Gasoline
**一.可供本地区消费的能源量**	**Total Primary Energy Supply**	**27068.03**	**-531.88**
1.一次能源生产量	Indigenous Production	22857.60	
水电	Hydro Power		
核电	Nuclear Power		
2.回收能	Recovery of Energy		
3.进口量	Import	5230.63	0.06
4.我轮、机在外国加油量	China Airplanes&ships Refueling in Abroad		
5.出口量(-)	Export (-)	-1023.92	-608.91
6.外轮、机在我国加油量(-)	Foreign Airplanes&ships Refueling in China		-17.66
7.库存增(-)、减(+)量	Stock Change	3.71	94.63
**二.加工转换投入(-)产出(+)量**	**Input(-) & Output(+) of Transformation**	**-26065.75**	**5503.29**
1.火力发电	Thermal Power	-114.52	-1.34
2.供热	Heating Supply	-18.40	-0.28
3.洗选煤	Coal Washing		
4.炼焦	Coking		
5.炼油	Petroleum Refineries	-25932.83	5504.90
6.制气	Gas Works		
#焦炭再投入量(-)	Coke Input (-)		
7.煤制品加工	Briquettes		
**三.损失量**	**Loss**	**263.41**	
**四.终端消费量**	**Total Final Consumption**	**742.03**	**4972.79**
1.农、林、牧、渔、水利业	Farming, Forestry, Animal Husbandry, Fishery & Water Conservancy		262.12
2.工 业	Industry	707.73	949.64
#用作原料、材料	Non-Energy Use	94.13	30.48
3.建筑业	Construction	4.56	167.40
4.交通运输、仓储及邮电通讯业	Transport, Storage, Postal & Telecommunications Services	27.54	1862.09
5.批发和零售贸易业、餐饮业	Wholesale, Retail Trade and Catering Service	0.24	303.58
6.生活消费	Residential Consumption		178.23
城 镇	Urban		133.56
乡 村	Rural		44.67
7.其他	Other	1.96	1249.73
**五.平衡差额**	**Statistical Difference**	**-3.16**	**-1.38**
**六.能源消费总量**	**Total Energy Consumption**		

(C)1994-2024 China Academic Journal Electronic Publishing House. All rights reserved. http://www.cnki.net

Continued

(10 000 tce)

煤油<br>Kerosene	柴油<br>Diesel Oil	燃料油<br>Fuel Oil	液化石油气<br>PLG	炼厂干气<br>Refinery Gas	其他石油制品<br>Other Petroleum Products	天然气<br>Natural Gas	热力<br>Heat	电力<br>Electricity	其他能源<br>Other Energy
**154.20**	**46.13**	**2773.88**	**566.15**		**-16.86**	**2902.19**		**2517.19**	**583.90**
						3351.33		2625.09	
								2415.97	
								183.72	
			28.95						583.90
310.74	44.97	2510.04	552.54		272.80			4.50	
89.81	36.66	294.66							
-183.88	-88.11	-36.37	-12.86		-289.66	-449.14		-112.40	
-55.66	-15.18	-19.04							
-6.81	67.80	24.60	-2.49						
**1094.35**	**8680.12**	**1027.56**	**1396.76**	**807.75**	**4632.84**	**-293.27**	**4584.62**	**12605.90**	**-280.33**
	-313.98	-1294.80	-3.19	-37.42		-168.64		12605.90	-96.42
		-430.39		-126.07		-124.62	4584.62		-74.99
									1.39
									1.10
1094.35	8994.10	2799.18	1399.95	971.24	4632.84				-111.41
		-46.43							
			**2.79**			**78.07**	**57.25**	**1059.31**	
**1212.74**	**8766.13**	**3848.65**	**2065.70**	**793.21**	**4616.01**	**2487.37**	**4528.75**	**14063.79**	**303.59**
2.10	1809.49	0.50	0.53				1.87	811.57	
115.28	1881.66	2582.29	431.95	793.21	4059.90	2078.26	3682.27	9796.10	303.59
13.44	35.56	125.16	47.13	1.75	2186.26	638.13			303.59
5.69	259.45	23.11	13.59		556.11	9.04	4.52	174.94	
743.97	3237.15	1200.02	1.05			10.51	21.94	313.12	
16.85	380.17	15.06	82.03			39.10	37.43	421.33	
104.19	91.30		1505.93			342.08	686.33	1819.88	
9.24	58.68		1304.58			342.08	686.33	1058.97	
94.95	32.62		201.34					760.91	
224.65	1106.92	27.66	30.62			8.38	94.39	726.86	
**35.81**	**-39.88**	**-47.20**	**-105.58**	**14.54**	**-0.03**	**43.49**	**-1.38**	**-0.01**	**-0.02**

(C)1994-2024 China Academic Journal Electronic Publishing House. All rights reserved. http://www.cnki.net

4-1 中国能源平衡表(实物量) -1999

		煤合计<br>(万吨)<br>Coal Total<br>(10^4 tn)	原煤<br>(万吨)<br>Raw Coal<br>(10^4 tn)
**一.可供本地区消费的能源量**	**Total Primary Energy Supply**	**127076.12**	**127410.95**
1.一次能源生产量	Indigenous Production	128000.00	128000.00
水电	Hydro Power		
核电	Nuclear Power		
2.回收能	Recovery of Energy		
3.进口量	Import	167.28	141.01
4.我轮、机在外国加油量	China Airplanes&Ships Refueling in Abroad		
5.出口量(-)	Export (-)	-3743.86	-3219.07
6.外轮、机在我国加油量(-)	Foreign Airplanes&Ships Refueling in China		
7.库存增(-)、减(+)量	Stock Change	2652.70	2489.01
**二.加工转换投入(-)产出(+)量**	**Input(-) & Output(+) of Transformation**	**-80285.78**	**-86000.94**
1.火力发电	Thermal Power	-52458.21	-51198.18
2.供热	Heating Supply	-7960.98	-7802.83
3.洗选煤	Coal Washing	-3091.53	-20244.10
4.炼焦	Coking	-15931.74	-5413.24
5.炼油	Petroleum Refineries		
6.制气	Gas Works	-917.55	-544.75
#焦炭再投入量(-)	Coke Input (-)		
7.煤制品加工	Briquettes	74.23	-797.84
**三.损失量**	**Loss**		
**四.终端消费量**	**Total Final Consumption**	**49714.22**	**43987.52**
1.农、林、牧、渔、水利业	Farming, Forestry, Animal Husbandry, Fishery & Water Conservancy	1735.61	1708.26
2.工业	Industry	36214.19	32268.24
#用作原料、材料	Non-Energy Use	2318.07	2142.61
3.建筑业	Construction	522.46	506.54
4.交通运输、仓储及邮电通迅业	Transport, Storage, Postal & Telecommunications Services	1286.26	1265.65
5.批发和零售贸易业、餐饮业	Wholesale, Retail Trade and Catering Service	896.20	876.03
6.生活消费	Residential Consumption	8408.42	6771.24
城 镇	Urban	3428.81	2519.30
乡 村	Rural	4979.61	4251.94
7.其他	Other	651.08	591.56
**五.平衡差额**	**Statistical Difference**	**-2923.88**	**-2577.51**
**六.能源消费合计**	**Total Energy Consumption**	**130000.00**	**129988.46**

(C)1994-2024 China Academic Journal Electronic Publishing House. All rights reserved. http://www.cnki.net

ENERGY BANLANCE OF CHINA －1999(PHYSICAL QUANTITY)

洗精煤（万吨）Cleaned Coal (10^4 tn)	其他洗煤（万吨）Other Washed Coal (10^4 tn)	型煤（万吨）Briquettes (10^4 tn)	焦炭（万吨）Coke (10^4 tn)	焦炉煤气（亿立方米）Coke Oven Gas (10^8 cu. m)	其他煤气（亿立方米）Other Gas (10^8 cu. m)	其他焦化产品（万吨）Other Coking Products (10^4 tn)	油品合计（万吨）Petroleum Products Total (10^4 tn)	原油（万吨）Crude Oil (10^4 tn)
**-415.64**	**90.45**	**-9.64**	**-1103.10**		**375.80**	**-0.93**	**20981.29**	**18947.24**
							16000.00	16000.00
					375.78		16.89	
26.27						27.60	6190.88	3661.37
							292.46	
-524.64		-0.15	-997.42			-28.53	-1569.94	-716.73
							-73.58	
82.73	90.45	-9.49	-105.68		0.02		124.58	2.60
**1151.94**	**3675.15**	**888.07**	**11915.91**	**207.59**	**70.87**	**181.25**	**-2222.18**	**-18245.66**
-89.93	-1170.10			-9.28	-65.63		-1228.56	-80.16
-5.74	-152.41			-7.71	-33.72		-394.57	-12.88
12089.29	5063.28							
-10481.18	-37.32		11859.23	222.07	14.66	168.86		
							-566.55	-18152.62
-360.50	-12.30		214.51	2.51	155.56	12.39	-32.50	
			-157.83					
	-16.00	888.07						
				**2.55**			**186.01**	**184.38**
**1042.77**	**3738.57**	**945.36**	**10302.69**	**211.66**	**446.19**	**186.41**	**18664.68**	**519.41**
	26.90	0.45	145.81				1422.07	
1034.43	2854.01	57.51	9937.06	171.64	360.54	186.41	8172.11	495.40
53.17	119.87	2.42	654.09	1.96		47.44	1959.67	65.89
4.33	11.59		17.10				747.25	3.19
3.80	16.56	0.25	10.14	0.02	0.20		4852.67	19.28
0.10	10.25	9.82	36.52	1.81	2.92		537.24	0.17
	763.41	873.77	143.08	35.07	81.44		1133.05	
	358.33	551.18	70.00	35.07	80.61		898.32	
	405.08	322.59	73.08		0.83		234.73	
0.11	55.85	3.56	12.98	3.12	1.09		1800.29	1.37
**-306.47**	**27.03**	**-66.93**	**510.12**	**-6.62**	**0.48**	**-6.09**	**-91.58**	**-2.21**
11980.12	5126.70	945.36	10460.52	231.20	545.54	186.41	21072.87	18949.45

(C)1994-2024 China Academic Journal Electronic Publishing House. All rights reserved. http://www.cnki.net

续表

		汽油<br>(万吨)<br>Gasoline<br>(10^4tn)	煤油<br>(万吨)<br>Kerosene<br>(10^4tn)
**一.可供本地区消费的能源量**	**Total Primary Energy Supply**	**-361.48**	**104.80**
1.一次能源生产量	Indigenous Production		
水电	Hydro Power		
核电	Nuclear Power		
2.回收能	Recovery of Energy		
3.进口量	Import	0.04	211.19
4.我轮、机在外国加油量	China Airplanes&Ships Refueling in Abroad		61.04
5.出口量(-)	Export (-)	-413.83	-124.97
6.外轮、机在我国加油量(-)	Foreign Airplanes&ships Refueling in China	-12.00	-37.83
7.库存增(-)、减(+)量	Stock Change	64.31	-4.63
**二.加工转换投入(-)产出(+)量**	**Input(-) & Output(+) of Transformation**	**3740.17**	**743.75**
1.火力发电	Thermal Power	-0.91	
2.供热	Heating Supply	-0.19	
3.洗选煤	Coal Washing		
4.炼焦	Coking		
5.炼油	Petroleum Refineries	3741.27	743.75
6.制气	Gas Works		
#焦炭再投入量(-)	Coke Input (-)		
7.煤制品加工	Briquettes		
**三.损失量**	**Loss**		
**四.终端消费量**	**Total Final Consumption**	**3379.63**	**824.21**
1.农、林、牧、渔、水利业	Farming, Forestry, Animal Husbandry, Fishery & Water Conservancy	178.14	1.43
2.工业	Industry	645.40	78.35
#用作原料、材料	Non-Energy Use	20.72	9.14
3.建筑业	Construction	113.77	3.87
4.交通运输、仓储及邮电通讯业	Transport, Storage, Postal & Telecommunications Services	1265.52	505.62
5.批发和零售贸易业、餐饮业	Wholesale, Retail Trade and Catering Service	206.32	11.45
6.生活消费	Residential Consumption	121.13	70.81
城镇	Urban	90.77	6.28
乡村	Rural	30.36	64.53
7.其他	Other	849.35	152.68
**五.平衡差额**	**Statistical Difference**	**-0.94**	**24.34**
**六.能源消费合计**	**Total Energy Consumption**	**3380.73**	**824.21**

(C)1994-2024 China Academic Journal Electronic Publishing House. All rights reserved. http://www.cnki.net

Continued

柴油<br>(万吨)<br>Diesel Oil<br>(10^4tn)	燃料油<br>(万吨)<br>Fuel Oil<br>(10^4tn)	液化石油气<br>(万吨)<br>PLG<br>(10^4tn)	炼厂干气<br>(万吨)<br>Refinery Gas<br>(10^4tn)	其他石油制品<br>(万吨)<br>Other Petroleum Products<br>(10^4tn)	天然气<br>(亿立方米)<br>Natural Gas<br>(10^8 cu. m)	热力<br>(万百万千焦)<br>Heat<br>(10^{10} kj)	电力<br>(亿千瓦小时)<br>Electricity<br>(10^8 kW · h)	其他能源<br>(万吨标煤)<br>Other Energy<br>(10^4tce)
**31.66**	**1941.68**	**330.25**		**-12.86**	**218.21**		**2048.16**	**583.90**
					251.98		2135.96	
							1965.80	
							149.49	
		16.89						583.90
30.86	1756.99	322.31		208.12			3.66	
25.16	206.26							
-60.47	-25.46	-7.50		-220.98	-33.77		-91.46	
-10.42	-13.33							
46.53	17.22	-1.45						
**5957.12**	**719.28**	**814.77**	**514.03**	**3534.36**	**-22.05**	**134446.33**	**10257.04**	**-280.33**
-215.48	-906.34	-1.86	-23.81		-12.68		10257.04	-96.42
	-301.27		-80.23		-9.37	134446.33		-74.99
								1.39
								1.10
6172.60	1959.39	816.63	618.07	3534.36				-111.41
	-32.50							
		**1.63**			**5.87**	**1678.81**	**861.93**	
**6016.15**	**2694.00**	**1204.98**	**504.78**	**3521.52**	**187.02**	**132808.04**	**11443.28**	**303.59**
1241.84	0.35	0.31				54.90	660.35	
1291.37	1807.57	251.97	504.78	3097.27	156.26	107984.44	7970.79	303.59
24.41	108.45	27.49	35.70	1667.88	47.98			303.59
178.06	16.18	7.93		424.25	0.68	132.65	142.34	
2221.64	840.00	0.61			0.79	643.39	254.78	
260.91	10.54	47.85			2.94	1097.74	342.82	
62.66		878.45			25.72	20126.84	1480.78	
40.27		761.00			25.72	20126.84	861.65	
22.39		117.45					619.13	
759.67	19.36	17.86			0.63	2768.08	591.42	
**-27.37**	**-33.04**	**-61.59**	**9.25**	**-0.02**	**3.27**	**-40.52**	**-0.01**	**-0.02**
**6231.63**	**3934.11**	**1208.47**	**608.82**	**3521.52**	**214.94**	**134486.85**	**12305.21**	**586.41**

(C)1994-2024 China Academic Journal Electronic Publishing House. All rights reserved. http://www.cnki.net

4-2 中国能源平衡表(标准量) -2000

单位:万吨标准煤

		能源合计 Energy Total	
		(发电煤耗计算法)<br>(coal equivalent calculation)	(电热当量计算法)<br>(calorific value calculation)
**一.可供本地区消费的能源量**	**Total Primary Energy Supply**	**136535.10**	**130448.14**
1.一次能源生产量	Indigenous Production	128977.88	122673.33
水电	Hydro Power	8541.84	2733.47
核电	Nuclear Power	642.79	205.70
2.回收能	Recovery of Energy	1759.74	1759.74
3.进口量	Import	13876.91	13836.53
4.我轮、机在外国加油量	China Airplanes&ships Refueling in Abroad	456.78	456.78
5.出口量(-)	Export (-)	-9462.79	-9204.83
6.外轮、机在我国加油量(-)	Foreign Airplanes&ships Refueling in China	-170.10	-170.10
7.库存增(-)、减(+)量	Stock Change	1096.68	1096.68
**二.加工转换投入(-)产出(+)量**	**Input(-) & Output(+) of Transformation**	**-2460.81**	**-33771.49**
1.火力发电	Thermal Power		-29097.10
2.供热	Heating Supply		-2213.59
3.洗选煤	Coal Washing	-861.49	-861.49
4.炼焦	Coking	-524.67	-524.67
5.炼油	Petroleum Refineries	-780.54	-780.54
6.制气	Gas Works	-119.51	-119.51
#焦炭再投入量(-)	Coke Input (-)	-138.78	-138.78
7.煤制品加工	Briquettes	-35.82	-35.82
**三.损失量**	**Loss**	**4062.15**	**1589.25**
**四.终端消费量**	**Total Final Consumption**	**132029.63**	**97108.34**
1.农、林、牧、渔、水利业	Farming, Forestry, Animal Husbandry, Fishery & Water Conservancy	6045.26	4286.98
2.工业	Industry	89265.68	64735.67
#用作原料、材料	Non-Energy Use	5757.44	5757.44
3.建筑业	Construction	2142.53	1736.29
4.交通运输、仓储及邮电通迅业	Transport, Storage, Postal & Telecommunications Services	9721.24	8977.14
5.批发和零售贸易业、餐饮业	Wholesale, Retail Trade and Catering Service	3038.77	1993.65
6.生活消费	Residential Consumption	15964.61	11246.36
城 镇	Urban	9656.09	6700.56
乡 村	Rural	6308.52	4545.80
7.其他	Other	5851.53	4132.27
**五.平衡差额**	**Statistical Difference**	**-2017.48**	**-2020.95**
**六.能源消费总量**	**Total Energy Consumption**	**138552.58**	**132469.09**

(C)1994-2024 China Academic Journal Electronic Publishing House. All rights reserved. http://www.cnki.net

ENERGY BALANCE OF CHINA －2000 (STANDARD QUANTITY)

(10 000 tce)

煤合计 Coal Total	原煤 Raw Coal	洗精煤 Cleaned Coal	其他洗煤 Other Washed Coal	型煤 Briquettes	焦炭 Coke	焦炉煤气 Coke Oven Gas	其他煤气 Other Gas	其他焦化产品 Other Coking Products	油品合计 Petroleum Products Total
**91398.91**	**91786**	**-364.06**	**-25.58**	**2.19**	**-1254.76**		**1175.57**	**31.17**	**32448.38**
92802.57	92803								23286.18
							1175.57		
161.19	127	30.97	3.26					52.53	13603.81
									456.78
-4234.70	-3643	-590.45	-0.55	-0.49	-1476.24			-21.36	-2933.64
									-170.10
2669.85	2500.05	195.42	-28.28	2.67	221.48				-1794.65
**-62152.36**	**-65629**	**980.22**	**2000.19**	**496.31**	**11696.77**	**1350.09**	**172.04**	**220.22**	**-3056.09**
-40514.61	-39785	-96.46	-633.08			-65.67	-201.36		-1691.00
-6128.05	-6035	-5.70	-87.52			-59.10	-100.45		-622.23
-861.49	-14703	11088.09	2753.36						
-13851.32	-4168	-9664.42	-18.91		11618.47	1458.14	43.93	205.41	
									-705.90
-761.07	-414	-341.28	-5.79		217.09	16.71	429.93	14.81	-36.97
					-138.78				
-35.82	-524		-7.88	496.31					
						**13.29**			**276.13**
**31910.82**	**28377**	**1057.76**	**1979.10**	**496.81**	**10002.64**	**1363.87**	**1346.95**	**249.49**	**28830.71**
1134.11	1121		13.28		140.06				2183.87
23353.70	20774	1050.02	1485.39	44.13	9653.46	1107.69	1078.32	249.49	12037.57
1497.62	1379	53.97	62.39	1.86	606.87	12.63		63.50	2625.78
369.59	359	3.99	7.10		18.44				1142.52
780.18	767	3.65	9.15	0.21	10.92	0.12	0.60		7804.47
560.60	551	0.09	5.38	3.79	34.69	11.58	7.59		811.27
5265.71	4390		428.87	446.61	133.28	225.33	257.24		2087.83
2021.74	1532		200.16	289.46	65.63	225.33	254.97		1685.48
3243.98	2858		228.70	157.15	67.65		2.27		402.35
446.93	415	0.01	29.94	2.08	11.80	19.14	3.19		2763.18
**-2664.27**	**-2220**	**-441.60**	**-4.49**	**1.69**	**439.37**	**-27.07**	**0.66**	**1.89**	**285.46**

(C)1994-2024 China Academic Journal Electronic Publishing House. All rights reserved. http://www.cnki.net

续表

单位:万吨标准煤

		原油<br>Crude Oil	汽油<br>Gasoline
**一.可供本地区消费的能源量**	**Total Primary Energy Supply**	**30547.74**	**-927.25**
1.一次能源生产量	Indigenous Production	23286.18	
水电	Hydro Power		
核电	Nuclear Power		
2.回收能	Recovery of Energy		
3.进口量	Import	10038.10	0.04
4.我轮、机在外国加油量	China Airplanes&ships Refueling in Abroad		
5.出口量(-)	Export (-)	-1472.32	-669.78
6.外轮、机在我国加油量(-)	Foreign Airplanes&ships Refueling in China		-18.39
7.库存增(-)、减(+)量	Stock Change	-1304.23	-239.12
**二.加工转换投入(-)产出(+)量**	**Input(-) & Output(+) of Transformation**	**-29149.58**	**6081.99**
1.火力发电	Thermal Power	-121.43	-1.47
2.供热	Heating Supply	-20.00	-0.29
3.洗选煤	Coal Washing		
4.炼焦	Coking		
5.炼油	Petroleum Refineries	-29008.15	6083.75
6.制气	Gas Works		
#焦炭再投入量(-)	Coke Input (-)		
7.煤制品加工	Briquettes		
**三.损失量**	**Loss**	**272.73**	
**四.终端消费量**	**Total Final Consumption**	**909.73**	**5155.36**
1.农、林、牧、渔、水利业	Farming, Forestry, Animal Husbandry, Fishery & Water Conservancy		271.49
2.工业	Industry	874.79	883.99
#用作原料、材料	Non-Energy Use	116.35	28.38
3.建筑业	Construction	4.71	170.02
4.交通运输、仓储及邮电通迅业	Transport, Storage, Postal & Telecommunications Services	27.97	2041.98
5.批发和零售贸易业、餐饮业	Wholesale, Retail Trade and Catering Service	0.26	308.76
6.生活消费	Residential Consumption		187.72
城 镇	Urban		140.03
乡 村	Rural		47.69
7.其他	Other	2.00	1291.40
**五.平衡差额**	**Statistical Difference**	**215.69**	**-0.62**
**六.能源消费总量**	**Total Energy Consumption**		

(C)1994-2024 China Academic Journal Electronic Publishing House. All rights reserved. http://www.cnki.net

Continued

(10 000 tce)

煤油 Kerosene	柴油 Diesel Oil	燃料油 Fuel Oil	液化石油气 PLG	炼厂干气 Refinery Gas	其他石油制品 Other Petroleum Products	天然气 Natural Gas	热力 Heat	电力 Electricity	其他能源 Other Energy
**12.65**	**-397.99**	**2547.17**	**822.08**		**-156.01**	**3200.11**		**2864.58**	**584.17**
						3617.60		2966.98	
								2733.47	
								205.70	
									584.17
375.90	37.78	2114.50	825.85		211.64			19.00	
98.61	37.88	320.28							
-292.63	-80.84	-47.67	-2.74		-367.65	-417.49		-121.40	
-84.55	-32.11	-35.04							
-84.68	-360.71	195.10	-1.03						
**1283.49**	**9983.99**	**1317.65**	**1568.10**	**890.23**	**4968.04**	**-391.82**	**4983.24**	**13693.35**	**-286.94**
	-331.72	-1163.19	-3.26	-39.29	-30.63	-202.29		13693.35	-115.51
		-416.05		-160.19	-25.69	-189.53	4983.24		-97.49
									0.70
1283.49	10315.71	2933.87	1571.36	1089.70	5024.36				-74.64
		-36.97							
			**3.39**			**88.58**	**60.04**	**1151.22**	
**1279.54**	**9539.07**	**3916.39**	**2336.23**	**882.36**	**4812.03**	**2778.50**	**4923.04**	**15405.11**	**297.21**
2.21	1909.00	0.57	0.60				1.88	827.07	
123.52	1994.48	2633.94	469.84	882.36	4174.66	2270.18	3974.95	10713.08	297.21
14.40	37.70	127.66	51.26	1.99	2248.06	653.83			297.21
5.89	285.39	23.87	15.27		637.36	10.91	4.63	190.21	
788.52	3706.59	1214.31	25.10			13.30	21.95	345.59	
17.66	372.93	16.56	95.11			45.75	38.33	483.83	
106.19	99.61		1694.31			429.86	792.28	2054.83	
9.56	63.86		1472.02			429.86	792.28	1225.28	
96.63	35.74		222.29					829.55	
235.56	1171.07	27.14	36.00			8.51	89.02	790.49	
**16.60**	**46.93**	**-51.57**	**50.55**	**7.87**		**-58.79**	**0.17**	**1.60**	**0.02**

(C)1994-2024 China Academic Journal Electronic Publishing House. All rights reserved. http://www.cnki.net

4-2 中国能源平衡表(实物量) -2000

		煤合计 (万吨) Coal Total (10^4 tn)	原煤 (万吨) Raw Coal (10^4 tn)
**一.可供本地区消费的能源量**	**Total Primary Energy Supply**	**128297.09**	**128741.12**
1.一次能源生产量	Indigenous Production	129921.00	129921.00
水电	Hydro Power		
核电	Nuclear Power		
2.回收能	Recovery of Energy		
3.进口量	Import	217.88	177.74
4.我轮、机在外国加油量	China Airplanes&Ships Refueling in Abroad		
5.出口量(-)	Export (-)	-5506.47	-4857.62
6.外轮、机在我国加油量(-)	Foreign Airplanes&Ships Refueling in China		
7.库存增(-)、减(+)量	Stock Change	3664.68	3500.00
**二.加工转换投入(-)产出(+)量**	**Input(-) & Output(+) of Transformation**	**-85178.61**	**-90878.52**
1.火力发电	Thermal Power	-55811.20	-54500.10
2.供热	Heating Supply	-8794.07	-8621.17
3.洗选煤	Coal Washing	-3191.20	-20583.70
4.炼焦	Coking	-16496.40	-5870.41
5.炼油	Petroleum Refineries		
6.制气	Gas Works	-959.99	-575.01
#焦炭再投入量(-)	Coke Input (-)		
7.煤制品加工	Briquettes	74.25	-728.13
**三.损失量**	**Loss**		
**四.终端消费量**	**Total Final Consumption**	**46821.39**	**41075.85**
1.农、林、牧、渔、水利业	Farming, Forestry, Animal Husbandry, Fishery & Water Conservancy	1647.68	1622.39
2.工业	Industry	34122.04	30070.56
#用作原料、材料	Non-Energy Use	2177.67	1996.69
3.建筑业	Construction	536.82	518.94
4.交通运输、仓储及邮电通讯业	Transport, Storage and Post	1132.24	1110.48
5.批发和零售贸易业、餐饮业	Wholesale, Retail Trade and Hotel ,Restaurants	814.64	798.06
6.生活消费	Residential Consumption	7906.96	6354.86
城镇	Urban	3075.56	2217.73
乡村	Rural	4831.40	4137.13
7.其他	Other	661.01	600.56
**五.平衡差额**	**Statistical Difference**	**-3702.91**	**-3213.25**
**六.能源消费合计**	**Total Energy Consumption**	**132000.00**	**131954.37**

(C)1994-2024 China Academic Journal Electronic Publishing House. All rights reserved. http://www.cnki.net

ENERGY BANLANCE OF CHINA -2000(PHYSICAL QUANTITY)

洗精煤<br>(万吨)<br>Cleaned Coal<br>(10^4 tn)	其他洗煤<br>(万吨)<br>Other Washed Coal<br>(10^4 tn)	型煤<br>(万吨)<br>Briquettes<br>(10^4 tn)	焦炭<br>(万吨)<br>Coke<br>(10^4 tn)	焦炉煤气<br>(亿立方米)<br>Coke Oven Gas<br>(10^8 cu. m)	其他煤气<br>(亿立方米)<br>Other Gas<br>(10^8 cu. m)	其他焦化产品<br>(万吨)<br>Other Coking Products<br>(10^4 tn)	油品合计<br>(万吨)<br>Petroleum Products Total<br>(10^4 tn)	原油<br>(万吨)<br>Crude Oil<br>(10^4 tn)
**-398.93**	**-48.70**	**3.60**	**-1291.70**		**408.78**	**27.01**	**22631.77**	**21382.99**
							16300.00	16300.00
					408.78			
33.94	6.20					45.52	9431.28	7026.53
							317.21	
-647.00	-1.05	-0.80	-1519.70			-18.51	-2055.61	-1030.60
							-116.53	
214.13	-53.85	4.40	228.00				-1244.58	-912.94
**1074.10**	**3808.43**	**817.38**	**12041.15**	**221.45**	**59.87**	**190.83**	**-2352.92**	**-20404.30**
-105.70	-1205.40			-10.69	-70.02		-1178.15	-85.00
-6.25	-166.65			-9.62	-34.93		-426.97	-14.00
12150.00	5242.50							
-10589.99	-36.00		11960.54	239.04	15.28	178.00		
							-721.92	-20305.30
-373.96	-11.02		223.48	2.72	149.54	12.83	-25.88	
			-142.87					
	-15.00	817.38						
				**2.18**			**192.89**	**190.91**
**1159.06**	**3768.28**	**818.20**	**10297.14**	**223.71**	**468.42**	**216.20**	**19893.51**	**636.80**
	25.29		144.18				1496.90	
1150.58	2828.23	72.67	9937.68	181.69	375.00	216.20	8529.99	612.34
59.14	118.79	3.06	624.74	2.07		55.02	2032.25	81.44
4.37	13.51		18.98				830.57	3.30
4.00	17.42	0.34	11.24	0.02	0.21		5351.71	19.58
0.10	10.24	6.24	35.71	1.90	2.64		545.03	0.18
	816.58	735.52	137.20	36.96	89.46		1256.45	
	381.12	476.71	67.56	36.96	88.67		1004.17	
	435.46	258.81	69.64		0.79		252.28	
0.01	57.01	3.43	12.15	3.14	1.11		1882.86	1.40
**-483.89**	**-8.55**	**2.78**	**452.31**	**-4.44**	**0.23**	**1.64**	**192.45**	**150.98**
**12234.96**	**5202.35**	**818.20**	**10440.01**	**246.20**	**573.37**	**216.20**	**22439.32**	**21232.01**

(C)1994-2024 China Academic Journal Electronic Publishing House. All rights reserved. http://www.cnki.net

续表

		汽油 (万吨) Gasoline (10^4tn)	煤油 万吨 Kerosene (10^4tn)
**一.可供本地区消费的能源量**	**Total Primary Energy Supply**	**-630.18**	**8.60**
1.一次能源生产量	Indigenous Production		
水电	Hydro Power		
核电	Nuclear Power		
2.回收能	Recovery of Energy		
3.进口量	Import	0.03	255.47
4.我轮、机在外国加油量	China Airplanes&Ships Refueling in Abroad		67.02
5.出口量(-)	Export (-)	-455.20	-198.88
6.外轮、机在我国加油量(-)	Foreign Airplanes&ships Refueling in China	-12.50	-57.46
7.库存增(-)、减(+)量	Stock Change	-162.51	-57.55
**二.加工转换投入(-)产出(+)量**	**Input(-) & Output(+) of Transformation**	**4133.47**	**872.29**
1.火力发电	Thermal Power	-1.00	
2.供热	Heating Supply	-0.20	
3.洗选煤	Coal Washing		
4.炼焦	Coking		
5.炼油	Petroleum Refineries	4134.67	872.29
6.制气	Gas Works		
#焦炭再投入量(-)	Coke Input (-)		
7.煤制品加工	Briquettes		
**三.损失量**	**Loss**		
**四.终端消费量**	**Total Final Consumption**	**3503.71**	**869.61**
1.农、林、牧、渔、水利业	Farming, Forestry, Animal Husbandry, Fishery & Water Conservancy	184.51	1.50
2.工业	Industry	600.78	83.95
#用作原料、材料	Non-Energy Use	19.29	9.79
3.建筑业	Construction	115.55	4.00
4.交通运输、仓储及邮电通迅业	Transport, Storage and Post	1387.78	535.90
5.批发和零售贸易业、餐饮业	Wholesale, Retail Trade and Hotel ,Restaurants	209.84	12.00
6.生活消费	Residential Consumption	127.58	72.17
城 镇	Urban	95.17	6.50
乡 村	Rural	32.41	65.67
7.其他	Other	877.67	160.09
**五.平衡差额**	**Statistical Difference**	**-0.42**	**11.28**
**六.能源消费合计**	**Total Energy Consumption**	**3504.91**	**869.61**

(C)1994-2024 China Academic Journal Electronic Publishing House. All rights reserved. http://www.cnki.net

Continued

柴油 (万吨) Diesel Oil (10⁴tn)	燃料油 (万吨) Fuel Oil (10⁴tn)	液化石油气 (万吨) PLG (10⁴tn)	炼厂干气 (万吨) Refinery Gas (10⁴tn)	其他石油制品 (万吨) Other Petroleum Products (10⁴tn)	天然气 (亿立方米) Natural Gas (10^8 cu. m)	热力 (万百万千焦) Heat (10^{10} kj)	电力 (亿千瓦小时) Electricity (10^8 kW·h)	其他能源 (万吨标煤) Other Energy (10⁴tce)
**-273.14**	**1782.98**	**479.54**		**-119.02**	**240.61**		**2330.82**	**584.17**
					272.00		2414.14	
							2224.14	
							167.37	
								584.17
25.93	1480.12	481.74		161.46			15.46	
26.00	224.19							
-55.48	-33.37	-1.60		-280.48	-31.39		-98.78	
-22.04	-24.53							
-247.55	136.57	-0.60						
**6851.96**	**922.34**	**914.72**	**566.52**	**3790.08**	**-29.46**	**146136.16**	**11141.86**	**-286.94**
-227.66	-814.22	-1.90	-25.00	-23.37	-15.21		11141.86	-115.51
	-291.23		-101.94	-19.60	-14.25	146136.16		-97.49
								0.70
7079.62	2053.67	916.62	693.46	3833.05				-74.64
	-25.88							
		**1.98**			**6.66**	**1760.58**	**936.71**	
**6546.61**	**2741.42**	**1362.79**	**561.51**	**3671.06**	**208.91**	**144370.70**	**12534.67**	**297.21**
1310.14	0.40	0.35				55.08	672.96	
1368.80	1843.72	274.07	561.51	3184.82	170.69	116567.55	8716.91	297.21
25.87	110.62	29.90	40.32	1715.03	49.16			297.21
195.86	16.71	8.91		486.24	0.82	135.64	154.77	
2543.81	850.00	14.64			1.00	643.71	281.20	
255.94	11.59	55.48			3.44	1124.12	393.68	
68.36		988.34			32.32	23234.03	1671.95	
43.83		858.67			32.32	23234.03	996.97	
24.53		129.67					674.98	
803.70	19.00	21.00			0.64	2610.57	643.20	
**32.21**	**-36.10**	**29.49**	**5.01**		**-4.42**	**4.88**	**1.30**	**0.02**
**6774.27**	**3872.75**	**1366.67**	**688.45**	**3714.03**	**245.03**	**146131.28**	**13471.38**	**584.85**

(C)1994-2024 China Academic Journal Electronic Publishing House. All rights reserved. http://www.cnki.net

4-3 中国能源平衡表(标准量) -2001

单位:万吨标准煤

		能源合计 Energy Total	
		(发电煤耗计算法)(coal equivalent calculation)	(电热当量计算法)(calorific value calculation)
**一.可供本地区消费的能源量**	**Total Primary Energy Supply**	**140981.30**	**133545.45**
1.一次能源生产量	Indigenous Production	137445.44	129793.63
水电	Hydro Power	10548.24	3409.64
核电	Nuclear Power	664.30	214.73
2.回收能	Recovery of Energy	1858.54	1858.54
3.进口量	Import	12968.58	12922.31
4.我轮、机在外国加油量	China Airplanes&ships Refueling in Abroad	503.50	503.50
5.出口量(-)	Export (-)	-11816.00	-11553.78
6.外轮、机在我国加油量(-)	Foreign Airplanes&ships Refueling in China	-203.53	-203.53
7.库存增(-)、减(+)量	Stock Change	224.79	224.79
**二.加工转换投入(-)产出(+)量**	**Input(-) & Output(+) of Transformation**	**-2325.33**	**-35089.64**
1.火力发电	Thermal Power		-30450.70
2.供热	Heating Supply		-2313.61
3.洗选煤	Coal Washing	-874.89	-874.89
4.炼焦	Coking	-517.90	-517.90
5.炼油	Petroleum Refineries	-607.12	-607.12
6.制气	Gas Works	-125.41	-125.41
#焦炭再投入量(-)	Coke Input (-)	-149.26	-149.26
7.煤制品加工	Briquettes	-50.75	-50.75
**三.损失量**	**Loss**	**4387.85**	**1701.52**
**四.终端消费量**	**Total Final Consumption**	**136486.03**	**98973.90**
1.农、林、牧、渔、水利业	Farming, Forestry, Animal Husbandry, Fishery & Water Conservancy	6400.29	4437.76
2.工业	Industry	91903.19	65592.66
#用作原料、材料	Non-Energy Use	5924.87	5924.87
3.建筑业	Construction	2234.03	1858.79
4.交通运输、仓储及邮电通迅业	Transport, Storage, Postal & Telecommunications Services	10019.66	9213.38
5.批发和零售贸易业、餐饮业	Wholesale, Retail Trade and Catering Service	3264.99	2102.67
6.生活消费	Residential Consumption	16567.50	11482.06
城 镇	Urban	9992.80	6896.55
乡 村	Rural	6574.70	4585.51
7.其他	Other	6096.37	4286.57
**五.平衡差额**	**Statistical Difference**	**-2217.91**	**-2219.61**
**六.能源消费总量**	**Total Energy Consumption**	**143199.21**	**135765.06**

(C)1994-2024 China Academic Journal Electronic Publishing House. All rights reserved. http://www.cnki.net

ENERGY BALANCE OF CHINA -2001 (STANDARD QUANTITY)

(10 000 tce)

煤合计 Coal Total	原煤 Raw Coal	洗精煤 Cleaned Coal	其他洗煤 Other Washed Coal	型煤 Briquettes	焦炭 Coke	焦炉煤气 Coke Oven Gas	其他煤气 Other Gas	其他焦化产品 Other Coking Products	油品合计 Petroleum Products Total
**92809.66**	**93808**	**-907.01**	**-91.06**	**-0.43**	**-1620.13**		**1242.35**	**40.52**	**33275.68**
98681.97	98682								23423.14
							1242.35		
192.41	159	25.25	8.59					54.82	12652.99
									503.50
-6945.48	-5900	-1044.50	-0.55	-0.18	-1345.00			-14.30	-2719.57
									-203.53
880.75	868	112.23	-99.11	-0.25	-275.13				-380.83
**-65582.01**	**-69419**	**1220.60**	**2095.77**	**520.37**	**12605.97**	**1404.51**	**135.03**	**235.35**	**-2955.42**
-42635.06	-41894	-103.45	-637.86			-65.24	-241.62	-0.76	-1740.80
-6412.99	-6317	-5.87	-89.84			-43.19	-115.38	-1.03	-637.82
-874.89	-15194	11462.68	2856.04						
-14788.36	-5003	-9765.22	-20.45		12522.57	1494.50	32.78	220.41	
									-544.29
-819.96	-446	-367.53	-6.87		232.66	18.43	459.25	16.72	-32.51
					-149.26				
-50.75	-566		-5.25	520.37					
						**16.52**			**270.90**
**30398.67**	**26685**	**1084.22**	**2107.90**	**521.64**	**10535.40**	**1411.82**	**1376.88**	**266.37**	**29517.19**
1075.44	1062		13.47		135.25				2288.24
22172.38	19447	1076.93	1601.26	46.75	10184.89	1165.13	1123.61	266.37	12004.84
1415.89	1291	55.35	67.25	1.97	752.90	13.28		67.79	2591.79
360.40	352	3.63	5.25		23.23				1282.15
701.36	690	3.56	7.32	0.27	11.35	0.12	0.72		8080.29
545.11	535	0.09	5.18	4.80	38.59	12.19	8.63		845.19
5103.23	4186		449.06	467.68	130.36	215.66	240.42		2148.42
1963.35	1451		210.10	301.98	66.30	215.66	238.09		1719.29
3139.88	2735		238.97	165.70	64.06		2.33		429.13
440.75	412	0.01	26.37	2.14	11.73	18.72	3.51		2868.06
**-3171.02**	**-2295**	**-770.64**	**-103.19**	**-1.70**	**450.45**	**-23.84**	**0.49**	**9.50**	**532.17**

(C)1994-2024 China Academic Journal Electronic Publishing House. All rights reserved. http://www.cnki.net

续表

单位:万吨标准煤

		原油 Crude Oil	汽油 Gasoline
**一.可供本地区消费的能源量**	**Total Primary Energy Supply**	**30767.99**	**-806.03**
1.一次能源生产量	Indigenous Production	23423.14	
水电	Hydro Power		
核电	Nuclear Power		
2.回收能	Recovery of Energy		
3.进口量	Import	8608.74	0.03
4.我轮、机在外国加油量	China Airplanes&ships Refueling in Abroad		
5.出口量(-)	Export (-)	-1078.59	-842.32
6.外轮、机在我国加油量(-)	Foreign Airplanes&ships Refueling in China		-19.86
7.库存增(-)、减(+)量	Stock Change	-185.30	56.12
**二.加工转换投入(-)产出(+)量**	**Input(-) & Output(+) of Transformation**	**-29287.33**	**6112.11**
1.火力发电	Thermal Power	-116.57	-0.88
2.供热	Heating Supply	-17.60	-0.18
3.洗选煤	Coal Washing		
4.炼焦	Coking		
5.炼油	Petroleum Refineries	-29153.15	6113.17
6.制气	Gas Works		
#焦炭再投入量(-)	Coke Input (-)		
7.煤制品加工	Briquettes		
**三.损失量**	**Loss**	**268.43**	
**四.终端消费量**	**Total Final Consumption**	**934.48**	**5292.67**
1.农、林、牧、渔、水利业	Farming, Forestry, Animal Husbandry, Fishery & Water Conservancy		280.45
2.工业	Industry	901.19	908.47
#用作原料、材料	Non-Energy Use	119.86	29.16
3.建筑业	Construction	4.81	171.71
4.交通运输、仓储及邮电通迅业	Transport, Storage, Postal & Telecommunications Services	26.54	2088.46
5.批发和零售贸易业、餐饮业	Wholesale, Retail Trade and Catering Service	0.21	314.94
6.生活消费	Residential Consumption		198.05
城 镇	Urban		147.02
乡 村	Rural		51.03
7.其他	Other	1.71	1330.59
**五.平衡差额**	**Statistical Difference**	**277.75**	**13.40**
**六.能源消费总量**	**Total Energy Consumption**		

(C)1994-2024 China Academic Journal Electronic Publishing House. All rights reserved. http://www.cnki.net

Continued

(10 000 tce)

煤油 Kerosene	柴油 Diesel Oil	燃料油 Fuel Oil	液化石油气 PLG	炼厂干气 Refinery Gas	其他石油制品 Other Petroleum Products	天然气 Natural Gas	热力 Heat	电力 Electricity	其他能源 Other Energy
**154.32**	**-311.19**	**2817.87**	**815.49**		**-162.76**	**3629.57**		**3551.61**	**616.19**
						4033.76		3654.76	
								3409.64	
								214.73	
									616.19
297.06	40.03	2605.19	838.05		263.88			22.10	
142.28	39.66	321.55							
-268.12	-37.33	-62.99	-3.58		-426.64	-404.19		-125.25	
-94.46	-30.95	-58.26							
77.56	-322.60	12.37	-18.98						
**1161.45**	**10557.00**	**1004.98**	**1630.78**	**864.27**	**5001.33**	**-391.15**	**5224.24**	**14544.29**	**-310.44**
	-350.27	-1197.94	-1.80	-36.14	-37.19	-172.90		14544.29	-138.61
		-428.04		-157.14	-34.87	-218.25	5224.24		-109.20
									0.20
1161.45	10907.27	2663.47	1632.58	1057.55	5073.39				-62.83
		-32.51							
			**2.47**			**82.73**	**61.23**	**1270.13**	
**1309.94**	**10006.30**	**3841.93**	**2414.88**	**878.41**	**4838.57**	**3174.31**	**5162.84**	**16825.00**	**305.43**
2.24	2004.45	0.60	0.51				1.86	936.98	
126.54	2034.72	2554.91	498.02	878.41	4102.57	2486.30	4206.77	11676.96	305.43
14.75	38.46	123.83	54.33	2.16	2209.23	777.78			305.43
5.15	325.05	23.11	16.30		736.00	9.58	5.35	178.09	
825.00	3891.91	1221.45	26.91			15.96	23.43	380.15	
18.35	390.60	17.54	103.54			66.50	39.69	546.77	
110.36	115.36		1724.65			586.66	796.90	2260.41	
10.33	74.49		1487.45			586.66	796.90	1310.31	
100.03	40.87		237.21					950.10	
222.31	1244.20	24.31	44.93			9.31	88.86	845.63	
**5.83**	**239.50**	**-19.09**	**28.92**	**-14.14**		**-18.62**	**0.17**	**0.77**	**0.32**

(C)1994-2024 China Academic Journal Electronic Publishing House. All rights reserved. http://www.cnki.net

4-3 中国能源平衡表(实物量) -2001

		煤合计 (万吨) Coal Total (10^4 tn)	原煤 (万吨) Raw Coal (10^4 tn)
**一.可供本地区消费的能源量**	**Total Primary Energy Supply**	**130554.02**	**131722.00**
1.一次能源生产量	Indigenous Production	138152.00	138152.00
水电	Hydro Power		
核电	Nuclear Power		
2.回收能	Recovery of Energy		
3.进口量	Import	266.02	222.00
4.我轮.机在外国加油量	China Airplanes&Ships Refueling in Abroad		
5.出口量(-)	Export (-)	-9012.87	-7867.00
6.外轮.机在我国加油量(-)	Foreign Airplanes&Ships Refueling in China		
7.库存增(-)、减(+)量	Stock Change	1148.87	1215.00
**二.加工转换投入(-)产出(+)量**	**Input(-) & Output(+) of Transformation**	**-89388.33**	**-95573.26**
1.火力发电	Thermal Power	-59797.86	-58470.00
2.供热	Heating Supply	-8951.49	-8774.00
3.洗选煤	Coal Washing	-2450.54	-20449.00
4.炼焦	Coking	-17236.37	-6497.00
5.炼油	Petroleum Refineries		
6.制气	Gas Works	-1002.07	-586.26
#焦炭再投入量(-)	Coke Input (-)		
7.煤制品加工	Briquettes	50.00	-797.00
**三.损失量**	**Loss**		
**四.终端消费量**	**Total Final Consumption**	**45611.67**	**39551.00**
1.农、林、牧、渔、水利业	Farming, Forestry, Animal Husbandry, Fishery & Water Conservancy	1599.64	1574.00
2.工业	Industry	33129.92	28824.00
#用作原料、材料	Non-Energy Use	2105.86	1913.91
3.建筑业	Construction	534.98	521.00
4.交通运输、仓储及邮电通迅业	Transport, Storage, Postal & Telecommunications Services	1041.28	1023.00
5.批发和零售贸易业、餐饮业	Wholesale, Retail Trade and Catering Service	810.87	793.00
6.生活消费	Residential Consumption	7830.25	6205.00
城镇	Urban	3048.36	2151.00
乡村	Rural	4781.89	4054.00
7.其他	Other	664.73	611.00
**五.平衡差额**	**Statistical Difference**	**-4445.98**	**-3402.26**
**六.消费量合计**	**Total Final Consumption**	**135000.00**	**135124.26**

(C)1994-2024 China Academic Journal Electronic Publishing House. All rights reserved. http://www.cnki.net

ENERGY BANLANCE OF CHINA －2001(PHYSICAL QUANTITY)

洗精煤（万吨）Cleaned Coal（10^4 tn）	其他洗煤（万吨）Other Washed Coal（10^4 tn）	型煤（万吨）Briquettes（10^4 tn）	焦炭（万吨）Coke（10^4 tn）	焦炉煤气（亿立方米）Coke Oven Gas（10^8 cu. m）	其他煤气（亿立方米）Other Gas（10^8 cu. m）	其他焦化产品（万吨）Other Coking Products（10^4 tn）	油品合计（万吨）Petroleum Products Total（10^4 tn）	原油（万吨）Crude Oil（10^4 tn）
**－993.88**	**－173.39**	**－0.71**	**－1667.83**		**432.00**	**35.11**	**23204.67**	**21537.16**
							16395.87	16395.87
					432.00			
27.67	16.35					47.50	8769.15	6026.00
							349.00	
－1144.53	－1.04	－0.30	－1384.60			－12.39	－1906.96	－755.00
							－139.72	
122.98	－188.70	－0.41	－283.23				－262.67	－129.71
**1337.50**	**3990.43**	**857.00**	**12977.12**	**230.35**	**47.00**	**203.94**	**－2291.99**	**－20500.72**
－113.36	－1214.50			－10.62	－84.02	－0.66	－1213.55	－81.60
－6.43	－171.06			－7.03	－40.12	－0.89	－438.66	－12.32
12560.46	5438.00							
－10700.44	－38.93		12891.26	245.00	11.40	191.00		
							－617.02	－20406.80
－402.73	－13.08		239.51	3.00	159.74	14.49	－22.76	
			－153.65					
	－10.00	857.00						
				**2.71**			**189.34**	**187.90**
**1188.06**	**4013.52**	**859.09**	**10845.58**	**231.55**	**478.83**	**230.82**	**20356.97**	**654.12**
	25.64		139.23				1568.48	
1180.07	3048.85	77.00	10484.75	191.09	390.75	230.82	8498.39	630.82
60.66	128.05	3.24	775.07	2.18		58.74	2008.54	83.90
3.98	10.00		23.91				933.83	3.37
3.90	13.93	0.45	11.68	0.02	0.25		5540.34	18.58
0.10	9.87	7.90	39.73	2.00	3.00		567.41	0.15
	855.03	770.22	134.20	35.37	83.61		1294.81	
	400.03	497.33	68.25	35.37	82.80		1025.73	
	455.00	272.89	65.95		0.81		269.08	
0.01	50.20	3.52	12.08	3.07	1.22		1953.71	1.20
**－844.44**	**－196.48**	**－2.80**	**463.71**	**－3.91**	**0.17**	**8.23**	**366.37**	**194.42**
**12411.02**	**5461.09**	**859.09**	**10999.23**	**251.91**	**602.97**	**232.37**	**22838.30**	**21342.74**

(C)1994-2024 China Academic Journal Electronic Publishing House. All rights reserved. http://www.cnki.net

续表

		汽油<br>(万吨)<br>Gasoline<br>(10^4tn)	煤油<br>(万吨)<br>Kerosene<br>(10^4tn)
**一.可供本地区消费的能源量**	**Total Primary Energy Supply**	**-547.80**	**104.88**
1.一次能源生产量	Indigenous Production		
水电	Hydro Power		
核电	Nuclear Power		
2.回收能	Recovery of Energy		
3.进口量	Import	0.02	201.89
4.我轮.机在外国加油量	China Airplanes&Ships Refueling in Abroad		96.70
5.出口量(-)	Export (-)	-572.46	-182.22
6.外轮.机在我国加油量(-)	Foreign Airplanes&ships Refueling in China	-13.50	-64.20
7.库存增(-)、减(+)量	Stock Change	38.14	52.71
**二.加工转换投入(-)产出(+)量**	**Input(-) & Output(+) of Transformation**	**4153.94**	**789.35**
1.火力发电	Thermal Power	-0.60	
2.供热	Heating Supply	-0.12	
3.洗选煤	Coal Washing		
4.炼焦	Coking		
5.炼油	Petroleum Refineries	4154.66	789.35
6.制气	Gas Works		
#焦炭再投入量(-)	Coke Input (-)		
7.煤制品加工	Briquettes		
**三.损失量**	**Loss**		
**四.终端消费量**	**Total Final Consumption**	**3597.03**	**890.27**
1.农、林、牧、渔、水利业	Farming, Forestry, Animal Husbandry, Fishery & Water Conservancy	190.60	1.52
2.工业	Industry	617.42	86.00
#用作原料、材料	Non-Energy Use	19.82	10.03
3.建筑业	Construction	116.70	3.50
4.交通运输、仓储及邮电通迅业	Transport, Storage, Postal & Telecommunications Services	1419.37	560.69
5.批发和零售贸易业、餐饮业	Wholesale, Retail Trade and Catering Service	214.04	12.47
6.生活消费	Residential Consumption	134.60	75.00
城 镇	Urban	99.92	7.02
乡 村	Rural	34.68	67.98
7.其他	Other	904.30	151.09
**五.平衡差额**	**Statistical Difference**	**9.11**	**3.96**
**六.消费量合计**	**Total Final Consumption**	**3597.75**	**890.27**

(C)1994-2024 China Academic Journal Electronic Publishing House. All rights reserved. http://www.cnki.net

Continued

柴油（万吨）Diesel Oil (10⁴tn)	燃料油（万吨）Fuel Oil (10⁴tn)	液化石油气（万吨）PLG (10⁴tn)	炼厂干气（万吨）Refinery Gas (10⁴tn)	其他石油制品（万吨）Other Petroleum Products (10⁴tn)	天然气（亿立方米）Natural Gas (10^8 cu. m)	热力（万百万千焦）Heat (10^{10} kj)	电力（亿千瓦小时）Electricity (10^8 kW·h)	其他能源（万吨标煤）Other Energy (10⁴tce)
**-213.57**	**1972.47**	**475.70**		**-124.17**	**272.90**		**2889.84**	**616.19**
					303.29		2973.77	
							2774.32	
							174.72	
								616.19
27.47	1823.60	488.86		201.31			17.98	
27.22	225.08							
-25.62	-44.09	-2.09		-325.48	-30.39		-101.91	
-21.24	-40.78							
-221.40	8.66	-11.07						
**7245.21**	**703.47**	**951.28**	**550.00**	**3815.48**	**-29.41**	**153203.45**	**11834.25**	**-310.44**
-240.39	-838.54	-1.05	-23.00	-28.37	-13.00		11834.25	-138.61
	-299.62		-100.00	-26.60	-16.41	153203.45		-109.20
								0.20
7485.60	1864.39	952.33	673.00	3870.45				-62.83
	-22.76							
		**1.44**			**6.22**	**1795.58**	**1033.47**	
**6867.27**	**2689.30**	**1408.67**	**559.00**	**3691.31**	**238.67**	**151402.97**	**13689.99**	**305.43**
1375.64	0.42	0.30				54.41	762.39	
1396.42	1788.40	290.51	559.00	3129.82	186.94	123365.60	9501.19	305.43
26.39	107.30	31.69	44.00	1685.41	58.48			305.43
223.08	16.18	9.51		561.49	0.72	156.94	144.91	
2671.00	855.00	15.70			1.20	687.06	309.32	
268.07	12.28	60.40			5.00	1163.83	444.89	
79.17		1006.04			44.11	23369.40	1839.23	
51.12		867.67			44.11	23369.40	1066.16	
28.05		138.37					773.07	
853.89	17.02	26.21			0.70	2605.73	688.06	
**164.37**	**-13.36**	**16.87**	**-9.00**		**-1.40**	**4.90**	**0.63**	**0.32**
**7107.66**	**3850.22**	**1411.16**	**682.00**	**3746.28**	**274.30**	**153198.55**	**14723.46**	**616.07**

(C)1994-2024 China Academic Journal Electronic Publishing House. All rights reserved. http://www.cnki.net

4-4 中国能源平衡表(标准量) -2002

单位:万吨标准煤

		能源合计 Energy Total	
		(发电煤耗计算法)(coal equivalent calculation)	(电热当量计算法)(calorific value calculation)
**一.可供本地区消费的能源量**	**Total Primary Energy Supply**	**149081.57**	**141437.93**
1.一次能源生产量	Indigenous Production	143809.83	135982.72
水电	Hydro Power	10675.20	3539.20
核电	Nuclear Power	931.46	308.81
2.回收能	Recovery of Energy	1907.98	1907.98
3.进口量	Import	15197.59	15140.60
4.我轮、机在外国加油量	China Airplanes&ships Refueling in Abroad	571.75	571.75
5.出口量(-)	Export (-)	-11474.36	-11233.90
6.外轮、机在我国加油量(-)	Foreign Airplanes&ships Refueling in China	-220.88	-220.88
7.库存增(-)、减(+)量	Stock Change	-710.34	-710.34
**二.加工转换投入(-)产出(+)量**	**Input(-) & Output(+) of Transformation**	**-2787.97**	**-37667.84**
1.火力发电	Thermal Power		-33159.02
2.供热	Heating Supply		-1720.85
3.洗选煤	Coal Washing	-922.72	-922.72
4.炼焦	Coking	-540.52	-540.52
5.炼油	Petroleum Refineries	-1015.44	-1015.44
6.制气	Gas Works	-123.88	-123.88
#焦炭再投入量(-)	Coke Input (-)	-133.42	-133.42
7.煤制品加工	Briquettes	-51.98	-51.98
**三.损失量**	**Loss**	**4778.11**	**1861.15**
**四.终端消费量**	**Total Final Consumption**	**144231.17**	**104625.94**
1.农、林、牧、渔、水利业	Farming, Forestry, Animal Husbandry, Fishery & Water Conservancy	6612.49	4688.30
2.工业	Industry	96864.19	68839.15
#用作原料、材料	Non-Energy Use	6024.56	6024.56
3.建筑业	Construction	2543.66	2135.02
4.交通运输、仓储及邮电通迅业	Transport, Storage, Postal & Telecommunications Services	10829.08	9983.02
5.批发和零售贸易业、餐饮业	Wholesale, Retail Trade and Catering Service	3520.34	2265.97
6.生活消费	Residential Consumption	17527.36	12288.91
城 镇	Urban	10629.98	7454.42
乡 村	Rural	6897.38	4834.49
7.其他	Other	6334.05	4425.56
**五.平衡差额**	**Statistical Difference**	**-2715.68**	**-2717.00**
**六.能源消费总量**	**Total Energy Consumption**	**151797.25**	**144154.93**

(C)1994-2024 China Academic Journal Electronic Publishing House. All rights reserved. http://www.cnki.net

ENERGY BALANCE OF CHINA -2002 (STANDARD QUANTITY)

(10 000 tce)

煤合计 Coal Total	原煤 Raw Coal	洗精煤 Cleaned Coal	其他洗煤 Other Washed Coal	型煤 Briquettes	焦炭 Coke	焦炉煤气 Coke Oven Gas	其他煤气 Other Gas	其他焦化产品 Other Coking Products	油品合计 Petroleum Products Total
**97409.42**	**98537.58**	**-1121.39**	**-6.69**	**-0.09**	**-1382.12**		**1263.63**	**43.16**	**35750.61**
103899.22	103899.22								23857.62
							1263.63		
806.23	774.66	23.34	8.22	0.01				57.33	14248.77
									571.75
-6507.06	-5290.49	-1213.33	-2.74	-0.50	-1318.21			-14.17	-2849.20
									-220.88
-788.97	-845.80	68.59	-12.16	0.41	-63.91				142.53
**-71169.09**	**-74954.59**	**1060.40**	**2172.54**	**552.55**	**13712.27**	**1486.69**	**126.31**	**246.63**	**-3408.47**
-47121.71	-46361.98	-107.05	-652.68			-84.04	-257.01		-1827.83
-6259.89	-6157.51	-6.30	-96.07			-29.67	-99.36		-613.88
-922.72	-16247.27	12366.72	2957.82						
-16027.94	-5138.06	-10866.24	-23.64		13631.47	1583.50	43.13	229.32	
									-939.75
-784.85	-450.49	-326.74	-7.63		214.22	16.89	439.56	17.31	-27.01
					-133.42				
-51.98	-599.28		-5.25	552.55					
									**272.28**
**29668.37**	**26072.15**	**850.86**	**2189.34**	**556.01**	**11857.45**	**1496.14**	**1389.54**	**288.50**	**31861.12**
1153.17	1141.14		12.02		136.95				2441.95
21157.12	18579.89	844.15	1680.64	52.43	11502.06	1248.53	1120.22	288.50	12914.50
1349.89	1233.70	43.39	70.59	2.21	778.50	14.23		73.42	2967.32
393.51	385.14	2.97	5.41		22.71				1501.83
750.24	738.14	3.65	8.15	0.30	11.11	0.12	0.60		8757.14
574.24	563.44	0.09	5.26	5.45	41.38	10.37	11.13		883.23
5174.84	4229.69		449.66	495.49	131.26	220.84	257.59		2456.71
1889.41	1369.42		207.87	312.11	67.27	220.84	254.51		1997.83
3285.43	2860.27		241.79	183.37	63.99		3.08		458.88
465.25	434.70		28.20	2.34	11.99	16.28			2905.75
**-3428.04**	**-2489.16**	**-911.85**	**-23.48**	**-3.55**	**472.70**	**-9.45**	**0.40**	**1.29**	**208.74**

(C)1994-2024 China Academic Journal Electronic Publishing House. All rights reserved. http://www.cnki.net

续表

单位:万吨标准煤

		原油<br>Crude Oil	汽油<br>Gasoline
**一.可供本地区消费的能源量**	**Total Primary Energy Supply**	**32527.78**	**-840.29**
1.一次能源生产量	Indigenous Production	23857.62	
水电	Hydro Power		
核电	Nuclear Power		
2.回收能	Recovery of Energy		
3.进口量	Import	9915.40	
4.我轮、机在外国加油量	China Airplanes&ships Refueling in Abroad		
5.出口量(-)	Export (-)	-1094.96	-900.79
6.外轮、机在我国加油量(-)	Foreign Airplanes&ships Refueling in China		-26.79
7.库存增(-)、减(+)量	Stock Change	-150.27	87.30
**二.加工转换投入(-)产出(+)量**	**Input(-) & Output(+) of Transformation**	**-30959.63**	**6356.48**
1.火力发电	Thermal Power	-111.90	-0.91
2.供热	Heating Supply	-18.23	-0.18
3.洗选煤	Coal Washing		
4.炼焦	Coking		
5.炼油	Petroleum Refineries	-30829.50	6357.57
6.制气	Gas Works		
#焦炭再投入量(-)	Coke Input (-)		
7.煤制品加工	Briquettes		
**三.损失量**	**Loss**	**269.26**	
**四.终端消费量**	**Total Final Consumption**	**973.25**	**5516.22**
1.农、林、牧、渔、水利业	Farming, Forestry, Animal Husbandry, Fishery & Water Conservancy		276.52
2.工业	Industry	940.02	928.31
#用作原料、材料	Non-Energy Use	125.02	29.80
3.建筑业	Construction	6.00	179.98
4.交通运输、仓储及邮电通迅业	Transport, Storage, Postal & Telecommunications Services	25.21	2212.25
5.批发和零售贸易业、餐饮业	Wholesale, Retail Trade and Catering Service	0.17	329.92
6.生活消费	Residential Consumption		241.02
城 镇	Urban		176.42
乡 村	Rural		64.59
7.其他	Other	1.84	1348.23
**五.平衡差额**	**Statistical Difference**	**325.64**	**-0.03**
**六.能源消费总量**	**Total Energy Consumption**		

(C)1994-2024 China Academic Journal Electronic Publishing House. All rights reserved. http://www.cnki.net

Continued

(10 000 tce)

煤油 Kerosene	柴油 Diesel Oil	燃料油 Fuel Oil	液化石油气 PLG	炼厂干气 Refinery Gas	其他石油制品 Other Petroleum Products	天然气 Natural Gas	热力 Heat	电力 Electricity	其他能源 Other Energy
**130.47**	**23.65**	**2653.34**	**1074.56**		**181.10**	**3917.91**		**3790.97**	**644.35**
						4343.91		3881.97	
								3539.20	
								308.81	
									644.35
315.66	69.53	2370.99	1073.43		503.77			28.27	
161.56	45.17	365.02							
-249.61	-180.23	-91.30	-9.63		-322.67	-426.00		-119.26	
-104.91	-30.60	-58.57							
7.77	119.77	67.20	10.77						
**1215.54**	**10899.27**	**924.69**	**1774.73**	**883.80**	**5496.66**	**-373.33**	**5598.91**	**16445.69**	**-333.45**
	-329.29	-1303.83	-2.64	-31.90	-47.36	-146.97		16445.69	-167.16
		-380.95		-177.82	-36.70	-226.37	5598.91		-90.60
1215.54	11228.56	2636.48	1777.37	1093.52	5580.72				-75.69
		-27.01							
			**3.02**			**84.19**	**68.40**	**1436.28**	
**1352.51**	**10842.51**	**3822.42**	**2780.75**	**895.70**	**5677.76**	**3423.95**	**5530.32**	**18799.75**	**310.80**
2.06	2162.79	0.59					2.24	953.99	
128.53	2193.43	2503.81	546.47	895.70	4778.25	2632.60	4442.63	13222.20	310.80
14.99	41.46	121.35	59.62	1.99	2573.09	798.00			43.20
	367.17	27.29	21.87		899.51	9.04	6.19	201.73	
907.49	4320.02	1245.88	46.29			20.75	27.65	415.40	
19.13	409.14	17.57	107.30			81.13	50.00	614.50	
89.31	122.28		2004.10			680.43	907.51	2459.75	
9.30	86.68		1725.43			680.43	907.51	1436.63	
80.01	35.60		278.68					1023.12	
206.00	1267.68	27.29	54.72				94.10	932.20	
**-6.50**	**80.40**	**-244.39**	**65.52**	**-11.90**		**36.44**	**0.18**	**0.63**	**0.10**

(C)1994-2024 China Academic Journal Electronic Publishing House. All rights reserved. http://www.cnki.net

4-4 中国能源平衡表(实物量)-2002

		煤合计<br>(万吨)<br>Coal Total<br>(10^4 tn)	原煤<br>(万吨)<br>Raw Coal<br>(10^4 tn)
**一.可供本地区消费的能源量**	**Total Primary Energy Supply**	**137060.75**	**138302.41**
1.一次能源生产量	Indigenous Production	145456.00	145456.00
水电	Hydro Power		
核电	Nuclear Power		
2.回收能	Recovery of Energy		
3.进口量	Import	1125.74	1084.50
4.我轮、机在外国加油量	China Airplanes&Ships Refueling in Abroad		
5.出口量(-)	Export (-)	-8389.56	-7053.99
6.外轮、机在我国加油量(-)	Foreign Airplanes&Ships Refueling in China		
7.库存增(-)、减(+)量	Stock Change	-1131.43	-1184.10
**二.加工转换投入(-)产出(+)量**	**Input(-) & Output(+) of Transformation**	**-99028.12**	**-105236.68**
1.火力发电	Thermal Power	-68600.03	-67240.00
2.供热	Heating Supply	-8973.73	-8783.90
3.洗选煤	Coal Washing	-1917.46	-21100.35
4.炼焦	Coking	-18624.72	-6672.80
5.炼油	Petroleum Refineries		
6.制气	Gas Works	-973.20	-600.65
#焦炭再投入量(-)	Coke Input (-)		
7.煤制品加工	Briquettes	61.02	-838.98
**三.损失量**	**Loss**		
**四.终端消费量**	**Total Final Consumption**	**42572.41**	**36555.78**
1.农、林、牧、渔、水利业	Farming, Forestry, Animal Husbandry, Fishery & Water Conservancy	1622.89	1600.00
2.工业	Industry	30262.23	26050.88
#用作原料、材料	Non-Energy Use	1915.36	1729.78
3.建筑业	Construction	553.55	540.00
4.交通运输、仓储及邮电通迅业	Transport, Storage, Postal & Telecommunications Services	1054.96	1034.95
5.批发和零售贸易业、餐饮业	Wholesale, Retail Trade and Catering Service	809.08	790.00
6.生活消费	Residential Consumption	7602.64	5930.45
城 镇	Urban	2829.89	1920.07
乡 村	Rural	4772.75	4010.38
7.其他	Other	667.06	609.50
**五.平衡差额**	**Statistical Difference**	**-4539.78**	**-3490.05**
**六.消费量合计**	**Total Final Consumption**	**141600.53**	**141792.46**

(C)1994-2024 China Academic Journal Electronic Publishing House. All rights reserved. http://www.cnki.net

ENERGY BANLANCE OF CHINA －2001(PHYSICAL QUANTITY)

洗精煤<br>(万吨)<br>Cleaned Coal<br>(10^4 tn)	其他洗煤<br>(万吨)<br>Other Washed Coal<br>(10^4 tn)	型煤<br>(万吨)<br>Briquettes<br>(10^4 tn)	焦炭<br>(万吨)<br>Coke<br>(10^4 tn)	焦炉煤气<br>(亿立方米)<br>Coke Oven Gas<br>(10^8 cu. m)	其他煤气<br>(亿立方米)<br>Other Gas<br>(10^8 cu. m)	其他焦化产品<br>(万吨)<br>Other Coking Products<br>(10^4 tn)	油品合计<br>(万吨)<br>Petroleum Products Total<br>(10^4 tn)	原油<br>(万吨)<br>Crude Oil<br>(10^4 tn)
**-1228.79**	**-12.73**	**-0.14**	**-1422.81**		**439.40**	**37.40**	**24925.09**	**22768.99**
							16700.00	16700.00
					439.40			
25.58	15.65	0.01				49.68	9873.03	6940.64
							396.31	
-1329.53	-5.22	-0.82	-1357.02			-12.28	-1987.68	-766.46
							-151.51	
75.16	-23.16	0.67	-65.79				94.94	-105.19
**1161.96**	**4136.60**	**910.00**	**14115.99**	**243.83**	**43.97**	**213.72**	**-2606.76**	**-21671.31**
-117.30	-1242.73			-13.68	-89.37		-1275.57	-78.33
-6.90	-182.93			-4.83	-34.55		-420.70	-12.76
13551.09	5631.80							
-11906.90	-45.02		14032.81	259.59	15.00	198.72		
							-891.58	-21580.22
-358.03	-14.52		220.53	2.75	152.89	15.00	-18.91	
			-137.35					
	-10.00	910.00						
							**190.24**	**188.48**
**932.35**	**4168.58**	**915.70**	**12206.56**	**245.38**	**483.23**	**250.00**	**21989.83**	**681.26**
	22.89		140.98				1674.05	
925.00	3200.00	86.35	11840.70	204.77	389.57	250.00	9168.28	658.00
47.55	134.40	3.64	801.42	2.33		63.63	2294.08	87.51
3.25	10.30		23.38				1096.60	4.20
4.00	15.51	0.50	11.44	0.02	0.21		6001.81	17.65
0.10	10.01	8.97	42.60	1.70	3.87		593.02	0.12
	856.17	816.02	135.12	36.22	89.58		1477.47	
	395.80	514.02	69.25	36.22	88.51		1192.20	
	460.37	302.00	65.87		1.07		285.27	
	53.70	3.86	12.34	2.67			1978.60	1.29
**-999.18**	**-44.71**	**-5.84**	**486.62**	**-1.55**	**0.14**	**1.12**	**138.26**	**227.94**
**13321.48**	**5663.78**	**915.70**	**12343.91**	**263.89**	**607.15**	**250.00**	**24786.83**	**22541.05**

(C)1994-2024 China Academic Journal Electronic Publishing House. All rights reserved. http://www.cnki.net

续表

		汽油<br>(万吨)<br>Gasoline<br>(10^4tn)	煤油<br>(万吨)<br>Kerosene<br>(10^4tn)
**一.可供本地区消费的能源量**	**Total Primary Energy Supply**	**-571.08**	**88.67**
1.一次能源生产量	Indigenous Production		
水电	Hydro Power		
核电	Nuclear Power		
2.回收能	Recovery of Energy		
3.进口量	Import		214.53
4.我轮、机在外国加油量	China Airplanes&Ships Refueling in Abroad		109.80
5.出口量(-)	Export (-)	-612.20	-169.64
6.外轮、机在我国加油量(-)	Foreign Airplanes&ships Refueling in China	-18.21	-71.30
7.库存增(-)、减(+)量	Stock Change	59.33	5.28
**二.加工转换投入(-)产出(+)量**	**Input(-) & Output(+) of Transformation**	**4320.02**	**826.11**
1.火力发电	Thermal Power	-0.62	
2.供热	Heating Supply	-0.12	
3.洗选煤	Coal Washing		
4.炼焦	Coking		
5.炼油	Petroleum Refineries	4320.76	826.11
6.制气	Gas Works		
#焦炭再投入量(-)	Coke Input (-)		
7.煤制品加工	Briquettes		
**三.损失量**	**Loss**		
**四.终端消费量**	**Total Final Consumption**	**3748.96**	**919.20**
1.农、林、牧、渔、水利业	Farming, Forestry, Animal Husbandry, Fishery & Water Conservancy	187.93	1.40
2.工业	Industry	630.90	87.35
#用作原料、材料	Non-Energy Use	20.25	10.19
3.建筑业	Construction	122.32	
4.交通运输、仓储及邮电通迅业	Transport, Storage, Postal & Telecommunications Services	1503.50	616.75
5.批发和零售贸易业、餐饮业	Wholesale, Retail Trade and Catering Service	224.22	13.00
6.生活消费	Residential Consumption	163.80	60.70
城 镇	Urban	119.90	6.32
乡 村	Rural	43.90	54.38
7.其他	Other	916.29	140.00
**五.平衡差额**	**Statistical Difference**	**-0.02**	**-4.42**
**六.消费量合计**	**Total Final Consumption**	**3749.70**	**919.20**

(C)1994-2024 China Academic Journal Electronic Publishing House. All rights reserved. http://www.cnki.net

Continued

柴油<br>(万吨)<br>Diesel Oil<br>(10^4tn)	燃料油<br>(万吨)<br>Fuel Oil<br>(10^4tn)	液化石油气<br>(万吨)<br>PLG<br>(10^4tn)	炼厂干气<br>(万吨)<br>Refinery Gas<br>(10^4tn)	其他石油制品<br>(万吨)<br>Other Petroleum Products<br>(10^4tn)	天然气<br>(亿立方米)<br>Natural Gas<br>(10^8 cu. m)	热力<br>(万百万千焦)<br>Heat<br>(10^{10} kj)	电力<br>(亿千瓦小时)<br>Electricity<br>(10^8 kW · h)	其他能源<br>(万吨标煤)<br>Other Energy<br>(10^4tce)
**16.23**	**1857.30**	**626.82**		**138.16**	**294.58**		**3084.60**	**644.35**
					326.61		3158.64	
							2879.74	
							251.27	
								644.35
47.72	1659.66	626.16		384.32			23.00	
31.00	255.51							
-123.69	-63.91	-5.62		-246.16	-32.03		-97.04	
-21.00	-41.00							
82.20	47.04	6.28						
**7480.11**	**647.27**	**1035.25**	**562.43**	**4193.36**	**-28.07**	**164190.77**	**13381.36**	**-333.45**
-225.99	-912.66	-1.54	-20.30	-36.13	-11.05		13381.36	-167.16
	-266.66		-113.16	-28.00	-17.02	164190.77		-90.60
7706.10	1845.50	1036.79	695.89	4257.49				-75.69
	-18.91							
		**1.76**			**6.33**	**2005.84**	**1168.66**	
**7441.16**	**2675.64**	**1622.09**	**570.00**	**4331.52**	**257.44**	**162179.59**	**15296.79**	**310.80**
1484.31	0.41					65.83	776.23	
1505.34	1752.63	318.77	570.00	3645.29	197.94	130282.41	10758.50	310.80
28.45	105.16	34.78	44.75	1962.99	60.00			43.20
251.99	19.10	12.76		686.23	0.68	181.67	164.14	
2964.81	872.10	27.00			1.56	810.73	338.00	
280.79	12.30	62.59			6.10	1466.42	500.00	
83.92		1169.05			51.16	26613.07	2001.42	
59.49		1006.49			51.16	26613.07	1168.94	
24.43		162.56					832.48	
870.00	19.10	31.92				2759.46	758.50	
**55.18**	**-171.07**	**38.22**	-7.57		**2.74**	**5.34**	**0.51**	**0.10**
**7667.15**	**3873.87**	**1625.39**	**703.46**	**4395.65**	**291.84**	**164185.43**	**16465.45**	**644.25**

(C)1994-2024 China Academic Journal Electronic Publishing House. All rights reserved. http://www.cnki.net

4-5 中国能源平衡表(标准量)-2003

单位:万吨标准煤

		能源合计 Energy Total	
		(发电煤耗计算法)(coal equivalent calculation)	(电热当量计算法)(calorific value calculation)
**一.可供本地区消费的能源量**	**Total Primary Energy Supply**	**172128.74**	**164409.96**
1.一次能源生产量	Indigenous Production	163841.53	155946.81
水电	Hydro Power	10268.65	3486.44
核电	Nuclear Power	1568.89	532.67
2.回收能	Recovery of Energy	2042.76	2042.76
3.进口量	Import	19573.99	19502.75
4.我轮、机在外国加油量	China Airplanes&ships Refueling in Abroad	474.14	474.14
5.出口量(-)	Export (-)	-12689.70	-12442.52
6.外轮、机在我国加油量(-)	Foreign Airplanes&ships Refueling in China	-299.76	-299.76
7.库存增(-)、减(+)量	Stock Change	-814.22	-814.22
**二.加工转换投入(-)产出(+)量**	**Input(-) & Output(+) of Transformation**	**-3377.59**	**-43505.36**
1.火力发电	Thermal Power		-37783.07
2.供热	Heating Supply		-2344.71
3.洗选煤	Coal Washing	-1191.50	-1191.50
4.炼焦	Coking	-768.61	-768.61
5.炼油	Petroleum Refineries	-1091.65	-1091.65
6.制气	Gas Works	-93.62	-93.62
#焦炭再投入量(-)	Coke Input (-)	-172.76	-172.76
7.煤制品加工	Briquettes	-59.44	-59.44
**三.损失量**	**Loss**	**4979.33**	**1938.52**
**四.终端消费量**	**Total Final Consumption**	**166633.39**	**121829.04**
1.农、林、牧、渔、水利业	Farming, Forestry, Animal Husbandry, Fishery & Water Conservancy	6715.97	4866.60
2.工业	Industry	113724.87	81710.69
#用作原料、材料	Non-Energy Use	6998.38	6998.38
3.建筑业	Construction	2859.57	2402.99
4.交通运输、仓储及邮电通迅业	Transport, Storage and Post	12507.57	11547.28
5.批发和零售贸易业、餐饮业	Wholesale, Retail Trade and Hotel ,Restaurants	4179.55	2669.75
6.生活消费	Residential Consumption	19827.16	14031.27
城 镇	Urban	12152.41	8462.01
乡 村	Rural	7674.76	5569.26
7.其他	Other	6818.69	4600.46
**五.平衡差额**	**Statistical Difference**	**-2861.57**	**-2862.96**
**六.能源消费总量**	**Total Energy Consumption**	**174990.30**	**167272.92**

(C)1994-2024 China Academic Journal Electronic Publishing House. All rights reserved. http://www.cnki.net

ENERGY BALANCE OF CHINA -2003 (STANDARD QUANTITY)

(10 000 tce)

煤合计 Coal Total	原煤 Raw Coal	洗精煤 Cleaned Coal	其他洗煤 Other Washed Coal	型煤 Briquettes	焦炭 Coke	焦炉煤气 Coke Oven Gas	其他煤气 Other Gas	其他焦化产品 Other Coking Products	油品合计 Petroleum Products Total
**116221.75**	**117331.58**	**-1051.30**	**-55.93**	**-2.60**	**-1792.07**		**1381.53**	**88.75**	**39472.99**
123002.46	123002.46								24229.03
							1381.53		
839.35	587.75	237.69	13.91		0.17			110.30	18516.31
									474.14
-7263.81	-6059.40	-1198.73	-3.09	-2.59	-1430.01			-21.55	-3350.98
									-299.76
-356.25	-199.23	-90.26	-66.75	-0.01	-362.23				-95.75
**-83655.99**	**-88237.26**	**1092.44**	**2822.73**	**666.10**	**17094.56**	**1680.73**	**150.45**	**263.59**	**-3792.25**
-54344.30	-53416.97	-112.98	-814.35			-69.72	-288.36		-2135.50
-7373.87	-7241.40	-7.30	-125.17			-29.06	-105.31		-611.20
-1191.50	-20801.90	15805.32	3805.07						
-19861.34	-5567.10	-14264.98	-29.26		17039.14	1759.85	48.62	245.12	
									-1015.65
-825.53	-489.60	-327.62	-8.30		228.18	19.66	495.51	18.46	-29.90
					-172.76				
-59.44	-720.29		-5.25	666.10					
									**231.61**
**37304.75**	**32858.37**	**1025.98**	**2754.96**	**665.44**	**13916.19**	**1694.05**	**1532.82**	**350.82**	**34845.86**
1324.35	1312.09		12.25		136.95				2452.69
27441.57	24149.63	1019.60	2208.39	63.95	13572.40	1426.93	1254.65	350.82	14010.25
1751.39	1603.54	52.41	92.75	2.69	837.53	16.27		80.78	3372.58
453.45	444.53	2.76	6.16		20.20				1679.44
840.17	828.74	3.50	7.62	0.31	10.48	0.09	0.89		10155.44
675.33	663.52	0.12	5.84	5.86	46.10	12.20	12.77		1013.97
6034.41	4961.34		479.56	593.51	119.00	238.55	264.52		2719.59
2131.30	1569.26		205.16	356.89	60.44	238.55	261.67		2197.18
3903.11	3392.09		274.40	236.62	58.56		2.85		522.41
535.47	498.52		35.14	1.81	11.06	16.28			2814.48
**-4738.99**	**-3764.05**	**-984.84**	**11.84**	**-1.94**	**1386.30**	**-13.32**	**-0.83**	**1.52**	**603.27**

(C)1994-2024 China Academic Journal Electronic Publishing House. All rights reserved. http://www.cnki.net

续表

单位:万吨标准煤

		原油 Crude Oil	汽油 Gasoline
**一. 可供本地区消费的能源量**	**Total Primary Energy Supply**	**35982.28**	**-1057.30**
1. 一次能源生产量	Indigenous Production	24229.03	
水电	Hydro Power		
核电	Nuclear Power		
2. 回收能	Recovery of Energy		
3. 进口量	Import	13003.13	
4. 我轮、机在外国加油量	China Airplanes&ships Refueling in Abroad		
5. 出口量(-)	Export (-)	-1161.92	-1109.79
6. 外轮、机在我国加油量(-)	Foreign Airplanes&ships Refueling in China		
7. 库存增(-)、减(+)量	Stock Change	-87.96	52.48
**二. 加工转换投入(-)产出(+)量**	**Input(-) & Output(+) of Transformation**	**-34213.54**	**7048.57**
1. 火力发电	Thermal Power	-134.27	-0.59
2. 供热	Heating Supply	-15.73	-0.12
3. 洗选煤	Coal Washing		
4. 炼焦	Coking		
5. 炼油	Petroleum Refineries	-34063.54	7049.27
6. 制气	Gas Works		
#焦炭再投入量(-)	Coke Input (-)		
7. 煤制品加工	Briquettes		
**三. 损失量**	**Loss**	**229.69**	
**四. 终端消费量**	**Total Final Consumption**	**1160.34**	**5990.86**
1. 农、林、牧、渔、水利业	Farming, Forestry, Animal Husbandry, Fishery & Water Conservancy		286.92
2. 工业	Industry	1132.88	908.44
#用作原料、材料	Non-Energy Use	150.67	29.16
3. 建筑业	Construction	5.71	181.95
4. 交通运输、仓储及邮电通迅业	Transport, Storage and Post	19.90	2739.22
5. 批发和零售贸易业、餐饮业	Wholesale, Retail Trade and Hotel ,Restaurants	0.13	350.33
6. 生活消费	Residential Consumption		292.44
城镇	Urban		211.71
乡村	Rural		80.74
7. 其他	Other	1.71	1231.56
**五. 平衡差额**	**Statistical Difference**	**378.71**	**0.40**
**六. 能源消费总量**	**Total Energy Consumption**		

(C)1994-2024 China Academic Journal Electronic Publishing House. All rights reserved. http://www.cnki.net

Continued

(10 000 tce)

煤油 Kerosene	柴油 Diesel Oil	燃料油 Fuel Oil	液化石油气 PLG	炼厂干气 Refinery Gas	其他石油制品 Other Petroleum Products	天然气 Natural Gas	热力 Heat	电力 Electricity	其他能源 Other Energy
**87.12**	**-95.09**	**3266.19**	**1070.49**		**219.30**	**4407.89**		**3967.89**	**661.23**
						4657.00		4058.33	
								3486.44	
								532.67	
									661.23
309.39	123.63	3422.14	1091.56		566.45			36.62	
157.59	39.04	277.52							
-296.77	-326.39	-108.77	-4.11		-343.22	-249.11		-127.07	
-109.18	-29.72	-160.86							
26.09	98.35	-163.83	-16.95		-3.93				
**1258.49**	**12037.83**	**952.00**	**2074.59**	**903.79**	**6146.01**	**-362.29**	**6045.93**	**19422.64**	**-352.73**
	-395.28	-1509.63	-2.67	-26.64	-66.42	-176.09		19422.64	-191.73
		-372.58		-188.69	-34.08	-186.20	6045.93		-85.00
1258.49	12433.11	2864.11	2077.27	1119.12	6246.51				-76.00
		-29.90							
			**1.92**			**88.45**	**69.09**	**1549.38**	
**1356.06**	**11858.58**	**4117.34**	**3075.28**	**920.84**	**6366.56**	**4059.03**	**5976.70**	**21840.46**	**308.37**
1.99	2162.92	0.86					2.42	950.20	
129.14	2271.95	2711.93	617.15	920.84	5317.92	3178.70	4633.68	15533.33	308.37
15.06	42.94	131.44	67.33	72.28	2863.70	888.17			43.15
	402.49	25.43	15.21		1048.64	9.31	7.36	233.24	
914.74	5078.28	1343.30	60.00			23.28	29.10	487.84	
16.54	518.04	18.57	110.37			91.11	52.64	765.63	
82.96	128.06		2216.13			756.64	1148.01	2750.55	
8.83	92.31		1884.34			756.64	1148.01	1668.21	
74.13	35.76		331.79					1082.34	
210.69	1296.82	17.26	56.43				103.49	1119.67	
**-10.45**	**84.16**	**100.86**	**67.89**	**-17.05**	**-1.25**	**-101.88**	**0.15**	**0.69**	**0.13**

(C)1994-2024 China Academic Journal Electronic Publishing House. All rights reserved. http://www.cnki.net

4－5　中国能源平衡表(实物量)－2003

		煤合计 (万吨) Coal Total (10^4 tn)	原煤 (万吨) Raw Coal (10^4 tn)
**一.可供本地区消费的能源量**	**Total Primary Energy Supply**	**163401.95**	**164664.72**
1.一次能源生产量	Indigenous Production	172200.00	172200.00
水电	Hydro Power		
核电	Nuclear Power		
2.回收能	Recovery of Energy		
3.进口量	Import	1109.77	822.83
4.我轮、机在外国加油量	China Airplanes&Ships Refueling in Abroad		
5.出口量(－)	Export (－)	－9402.89	－8079.20
6.外轮、机在我国加油量(－)	Foreign Airplanes&Ships Refueling in China		
7.库存增(－)、减(＋)量	Stock Change	－504.93	－278.91
**二.加工转换投入(－)产出(＋)量**	**Input(－) & Output(＋) of Transformation**	**－120187.27**	**－127855.92**
1.火力发电	Thermal Power	－81976.47	－80302.12
2.供热	Heating Supply	－10895.45	－10649.12
3.洗选煤	Coal Washing	－2699.30	－27263.30
4.炼焦	Coking	－23639.86	－7953.00
5.炼油	Petroleum Refineries		
6.制气	Gas Works	－1054.81	－680.00
#焦炭再投入量(－)	Coke Input (－)		
7.煤制品加工	Briquettes	78.62	－1008.38
**三.损失量**	**Loss**		
**四.终端消费量**	**Total Final Consumption**	**49044.77**	**41570.90**
1.农、林、牧、渔、水利业	Farming, Forestry, Animal Husbandry, Fishery & Water Conservancy	1683.33	1660.00
2.工业	Industry	35981.21	30553.00
#用作原料、材料	Non-Energy Use	2267.22	2028.72
3.建筑业	Construction	577.15	562.40
4.交通运输、仓储及邮电通迅业	Transport, Storage and Post	1067.33	1048.48
5.批发和零售贸易业、餐饮业	Wholesale, Retail Trade and Hotel ,Restaurants	860.42	839.45
6.生活消费	Residential Consumption	8174.71	6276.86
城 镇	Urban	2968.13	1985.35
乡 村	Rural	5206.58	4291.51
7.其他	Other	700.62	630.71
**五.平衡差额**	**Statistical Difference**	**－5830.09**	**－4762.10**
**六.能源消费合计**	**Total Energy Consumption**	**169232.04**	**169426.82**

(C)1994-2024 China Academic Journal Electronic Publishing House. All rights reserved.　http://www.cnki.net

ENERGY BANLANCE OF CHINA －2003(PHYSICAL QUANTITY)

洗精煤<br>(万吨)<br>Cleaned Coal<br>(10^4 tn)	其他洗煤<br>(万吨)<br>Other Washed Coal<br>(10^4 tn)	型煤<br>(万吨)<br>Briquettes<br>(10^4 tn)	焦炭<br>(万吨)<br>Coke<br>(10^4 tn)	焦炉煤气<br>(亿立方米)<br>Coke Oven Gas<br>(10^8 cu. m)	其他煤气<br>(亿立方米)<br>Other Gas<br>(10^8 cu. m)	其他焦化产品<br>(万吨)<br>Other Coking Products<br>(10^4 tn)	油品合计<br>(万吨)<br>Petroleum Products Total<br>(10^4 tn)	原油<br>(万吨)<br>Crude Oil<br>(10^4 tn)
**-1151.98**	**-106.50**	**-4.29**	**-1844.83**		**480.40**	**76.91**	**27540.51**	**25187.09**
							16959.98	16959.98
					480.40			
260.45	26.49		0.17			95.58	12861.46	9102.01
							328.15	
-1313.53	-5.89	-4.27	-1472.11			-18.67	-2333.64	-813.33
							-207.20	
-98.90	-127.10	-0.02	-372.89				-68.24	-61.57
**1197.06**	**5374.59**	**1097.00**	**17597.86**	**275.62**	**52.37**	**228.41**	**-2901.36**	**-23949.00**
-123.80	-1550.55			-11.35	-100.27		-1491.57	-93.99
-8.00	-238.33			-4.73	-36.62		-417.97	-11.01
17319.00	7245.00							
-15631.14	-55.72		17540.81	288.50	16.91	212.41		
							-970.89	-23844.00
-359.00	-15.81		234.90	3.20	172.35	16.00	-20.93	
			-177.85					
	-10.00	1097.00						
							**161.90**	**160.78**
**1124.24**	**5245.54**	**1104.09**	**14325.91**	**277.81**	**533.06**	**304.00**	**24062.83**	**812.22**
	23.33		140.98				1681.35	
1117.25	4204.86	106.10	13972.00	234.00	436.32	304.00	9958.71	793.00
57.43	176.60	4.47	862.19	2.67		77.37	5280.77	105.47
3.02	11.73		20.79				1230.56	4.00
3.84	14.50	0.51	10.79	0.02	0.31		6957.74	13.93
0.13	11.12	9.72	47.46	2.00	4.44		682.33	0.09
	913.10	984.75	122.50	39.12	91.99		1635.75	
	390.63	592.15	62.22	39.12	91.00		1312.42	
	522.47	392.60	60.28		0.99		323.33	
	66.90	3.01	11.39	2.67			1916.39	1.20
**-1079.16**	**22.55**	**-11.38**	**1427.12**	**-2.18**	**-0.29**	**1.32**	**414.42**	**265.09**
**17246.18**	**7115.95**	**1104.09**	**14503.76**	**293.89**	**669.95**	**304.00**	**27126.09**	**24922.00**

(C)1994-2024 China Academic Journal Electronic Publishing House. All rights reserved. http://www.cnki.net

续表

		汽油（万吨）Gasoline (10^4tn)	煤油（万吨）Kerosene (10^4tn)
**一.可供本地区消费的能源量**	**Total Primary Energy Supply**	**-718.57**	**59.21**
1.一次能源生产量	Indigenous Production		
水电	Hydro Power		
核电	Nuclear Power		
2.回收能	Recovery of Energy		
3.进口量	Import		210.27
4.我轮、机在外国加油量	China Airplanes&Ships Refueling in Abroad		107.10
5.出口量(-)	Export (-)	-754.24	-201.69
6.外轮、机在我国加油量(-)	Foreign Airplanes&ships Refueling in China		-74.20
7.库存增(-)、减(+)量	Stock Change	35.67	17.73
**二.加工转换投入(-)产出(+)量**	**Input(-) & Output(+) of Transformation**	**4790.38**	**855.30**
1.火力发电	Thermal Power	-0.40	
2.供热	Heating Supply	-0.08	
3.洗选煤	Coal Washing		
4.炼焦	Coking		
5.炼油	Petroleum Refineries	4790.86	855.30
6.制气	Gas Works		
#焦炭再投入量(-)	Coke Input (-)		
7.煤制品加工	Briquettes		
**三.损失量**	**Loss**		
**四.终端消费量**	**Total Final Consumption**	**4071.54**	**921.61**
1.农、林、牧、渔、水利业	Farming, Forestry, Animal Husbandry, Fishery & Water Conservancy	195.00	1.35
2.工业	Industry	617.40	87.77
#用作原料、材料	Non-Energy Use	19.82	10.23
3.建筑业	Construction	123.66	
4.交通运输、仓储及邮电通迅业	Transport, Storage and Post	1861.64	621.68
5.批发和零售贸易业、餐饮业	Wholesale, Retail Trade and Hotel, Restaurants	238.09	11.24
6.生活消费	Residential Consumption	198.75	56.38
城镇	Urban	143.88	6.00
乡村	Rural	54.87	50.38
7.其他	Other	837.00	143.19
**五.平衡差额**	**Statistical Difference**	**0.27**	**-7.10**
**六.能源消费合计**	**Total Energy Consumption**	**4072.02**	**921.61**

(C)1994-2024 China Academic Journal Electronic Publishing House. All rights reserved. http://www.cnki.net

Continued

柴油（万吨）Diesel Oil (10^4 tn)	燃料油（万吨）Fuel Oil (10^4 tn)	液化石油气（万吨）PLG (10^4 tn)	炼厂干气（万吨）Refinery Gas (10^4 tn)	其他石油制品（万吨）Other Petroleum Products (10^4 tn)	天然气（亿立方米）Natural Gas (10^8 cu. m)	热力（万百万千焦）Heat (10^10 kj)	电力（亿千瓦小时）Electricity (10^8 kW·h)	其他能源（万吨标煤）Other Energy (10^4 tce)
**-65.26**	**2286.29**	**624.45**		**167.30**	**331.42**		**3228.55**	**661.23**
					350.15		3302.14	
							2836.81	
							433.42	
								661.23
84.85	2395.45	636.74		432.14			29.80	
26.79	194.26							
-224.00	-76.14	-2.40		-261.84	-18.73		-103.39	
-20.40	-112.60							
67.50	-114.68	-9.89		-3.00				
**8261.50**	**666.39**	**1210.17**	**575.15**	**4688.75**	**-27.24**	**177300.00**	**15803.61**	**-352.73**
-271.28	-1056.72	-1.56	-16.95	-50.67	-13.24		15803.61	-191.73
	-260.80		-120.08	-26.00	-14.00	177300.00		-85.00
8532.78	2004.84	1211.73	712.18	4765.42				-76.00
	-20.93							
		**1.12**			**6.65**	**2026.06**	**1260.68**	
**8138.48**	**2882.08**	**1793.90**	**586.00**	**4857.00**	**305.19**	**175269.66**	**17770.92**	**308.37**
1484.40	0.60					70.83	773.15	
1559.23	1898.31	360.00	586.00	4057.00	239.00	135885.00	12639.00	308.37
29.47	113.90	39.28	46.00	2184.69	66.78			43.15
276.23	17.80	8.87		800.00	0.70	215.93	189.78	
3485.20	940.29	35.00			1.75	853.31	396.94	
355.53	13.00	64.38			6.85	1543.69	622.97	
87.89		1292.73			56.89	33666.00	2238.04	
63.35		1099.19			56.89	33666.00	1357.37	
24.54		193.54					880.67	
890.00	12.08	32.92				3034.90	911.04	
**57.76**	**70.60**	**39.60**	**-10.85**	**-0.95**	**-7.66**	**4.28**	**0.56**	**0.13**
**8409.76**	**4220.53**	**1796.58**	**723.03**	**4933.67**	**339.08**	**177295.72**	**19031.60**	**661.10**

(C)1994-2024 China Academic Journal Electronic Publishing House. All rights reserved. http://www.cnki.net

4－6 中国能源平衡表(标准量)－2004

单位:万吨标准煤

		能源合计 Energy Total	
		(发电煤耗计算法)(coal equivalent calculation)	(电热当量计算法)(calorific value calculation)
**一.可供本地区消费的能源量**	**Total Primary Energy Supply**	**203343.73**	**194104.38**
1.一次能源生产量	Indigenous Production	187341.15	177962.03
水电	Hydro Power	12477.88	4345.06
核电	Nuclear Power	1781.24	620.26
2.回收能	Recovery of Energy	2507.99	2507.99
3.进口量	Import	26045.95	25967.73
4.我轮、机在外国加油量	China Airplanes&ships Refueling in Abroad	546.74	546.74
5.出口量(－)	Export (－)	－11159.24	－10941.26
6.外轮、机在我国加油量(－)	Foreign Airplanes&ships Refueling in China	－486.79	－486.79
7.库存增(－)、减(＋)量	Stock Change	－1452.07	－1452.07
**二.加工转换投入(－)产出(＋)量**	**Input(－) & Output(＋) of Transformation**	**－3683.59**	**－47622.04**
1.火力发电	Thermal Power		－41305.20
2.供热	Heating Supply		－2633.25
3.洗选煤	Coal Washing	－1326.65	－1326.65
4.炼焦	Coking	－526.88	－526.88
5.炼油	Petroleum Refineries	－1415.60	－1415.60
6.制气	Gas Works	－162.31	－162.31
#焦炭再投入量(－)	Coke Input (－)	－196.36	－196.36
7.煤制品加工	Briquettes	－55.78	－55.78
**三.损失量**	**Loss**	**5439.16**	**2140.34**
**四.终端消费量**	**Total Final Consumption**	**194103.93**	**144227.22**
1.农、林、牧、渔、水利业	Farming, Forestry, Animal Husbandry, Fishery & Water Conservancy	7679.89	5818.18
2.工业	Industry	134442.37	98384.39
#用作原料、材料	Non-Energy Use	8412.26	8412.26
3.建筑业	Construction	3258.61	2741.89
4.交通运输、仓储及邮电通迅业	Transport, Storage, Postal & Telecommunications Services	14783.26	13732.18
5.批发和零售贸易业、餐饮业	Wholesale, Retail Trade and Catering Service	4820.32	3098.56
6.生活消费	Residential Consumption	21280.66	15045.43
城 镇	Urban	12973.12	8994.90
乡 村	Rural	8307.54	6050.52
7.其他	Other	7838.84	5406.60
**五.平衡差额**	**Statistical Difference**	**117.05**	**114.77**
**六.能源消费总量**	**Total Energy Consumption**	**203226.68**	**193989.61**

(C)1994-2024 China Academic Journal Electronic Publishing House. All rights reserved.   http://www.cnki.net

ENERGY BALANCE OF CHINA －2004 (STANDARD QUANTITY)

(10 000 tce)

煤合计 Coal Total	原煤 Raw Coal	洗精煤 Cleaned Coal	其他洗煤 Other Washed Coal	型煤 Briquettes	焦炭 Coke	焦炉煤气 Coke Oven Gas	其他煤气 Other Gas	其他焦化产品 Other Coking Products	油品合计 Petroleum Products Total
**137316.09**	**137335.12**	**-36.23**	**20.29**	**-3.10**	**-2015.08**		**1640.36**	**127.27**	**46042.22**
142311.70	142311.70								25125.25
							1640.36		
1444.68	811.03	614.66	18.99		0.52			160.56	24320.19
									546.74
-6296.41	-5774.32	-518.09	-0.91	-3.10	-1458.27			-33.28	-2712.32
									-486.79
-143.89	-13.29	-132.81	2.21		-557.34				-750.84
**-92017.76**	**-97151.22**	**1638.95**	**2810.85**	**683.66**	**19178.62**	**1838.78**	**16.13**	**328.50**	**-4662.64**
-59662.31	-58504.17	-140.92	-1017.22			-154.07	-333.01		-2671.73
-7936.42	-7820.14	-10.58	-105.71			-87.35	-229.58		-609.83
-1326.65	-26280.83	20312.69	4641.49						
-22021.99	-3841.50	-18146.40	-34.09		19068.37	2054.22	58.29	314.23	
									-1366.06
-1014.60	-629.66	-375.84	-9.10		299.00	25.98	520.43	21.89	-15.01
					-188.74			-7.6164	
-55.78	-74.92		-664.52	683.66					
									**214.13**
**46348.21**	**40957.00**	**1798.16**	**2904.90**	**688.15**	**16584.43**	**1827.69**	**1656.61**	**455.22**	**40507.03**
1805.16	1792.13		13.02		95.86				2920.55
35990.13	31732.11	1795.37	2376.61	86.05	16306.73	1550.00	1358.80	455.22	15855.57
2302.73	2107.01	92.28	99.82	3.62	799.46	17.67		115.85	4123.16
483.23	478.06	2.79	2.38		16.31				1936.62
668.35	665.60		2.75		1.74		0.60		12400.29
696.11	682.47		5.79	7.84	51.83	11.24	18.52		1219.78
6134.59	5069.99		472.10	592.50	102.15	252.93	278.68		2942.63
1902.82	1395.15		180.19	327.48	53.08	252.93	275.44		2386.01
4231.77	3674.84		291.91	265.01	49.07		3.25		556.61
570.64	536.63		32.25	1.77	9.80	13.51			3231.60
**-1049.88**	**-773.10**	**-195.43**	**-73.76**	**-7.59**	**579.11**	**11.10**	**-0.12**	**0.56**	**658.42**

(C)1994-2024 China Academic Journal Electronic Publishing House. All rights reserved. http://www.cnki.net

续表

单位:万吨标准煤

		原油 Crude Oil	汽油 Gasoline
**一.可供本地区消费的能源量**	**Total Primary Energy Supply**	**41446.99**	**-835.87**
1.一次能源生产量	Indigenous Production	25125.25	
水电	Hydro Power		
核电	Nuclear Power		
2.回收能	Recovery of Energy		
3.进口量	Import	17531.78	
4.我轮、机在外国加油量	China Airplanes&ships Refueling in Abroad		
5.出口量(-)	Export (-)	-784.53	-795.60
6.外轮、机在我国加油量(-)	Foreign Airplanes&ships Refueling in China		
7.库存增(-)、减(+)量	Stock Change	-425.51	-40.27
**二.加工转换投入(-)产出(+)量**	**Input(-) & Output(+) of Transformation**	**-39651.59**	**7746.63**
1.火力发电	Thermal Power	-25.77	-0.44
2.供热	Heating Supply	-0.36	-0.22
3.洗选煤	Coal Washing		
4.炼焦	Coking		
5.炼油	Petroleum Refineries	-39625.46	7747.29
6.制气	Gas Works		
#焦炭再投入量(-)	Coke Input (-)		
7.煤制品加工	Briquettes		
**三.损失量**	**Loss**	**212.59**	
**四.终端消费量**	**Total Final Consumption**	**1207.08**	**6908.68**
1.农、林、牧、渔、水利业	Farming, Forestry, Animal Husbandry, Fishery & Water Conservancy		323.90
2.工业	Industry	1207.08	745.93
#用作原料、材料	Non-Energy Use	160.54	23.94
3.建筑业	Construction		230.26
4.交通运输、仓储及邮电通迅业	Transport, Storage, Postal & Telecommunications Services		3396.67
5.批发和零售贸易业、餐饮业	Wholesale, Retail Trade and Catering Service		411.70
6.生活消费	Residential Consumption		421.61
城 镇	Urban		329.48
乡 村	Rural		92.14
7.其他	Other		1378.61
**五.平衡差额**	**Statistical Difference**	**375.72**	**2.07**
**六.能源消费总量**	**Total Energy Consumption**		

(C)1994-2024 China Academic Journal Electronic Publishing House. All rights reserved. http://www.cnki.net

Continued

(10 000 tce)

煤油 Kerosene	柴油 Diesel Oil	燃料油 Fuel Oil	液化石油气 PLG	炼厂干气 Refinery Gas	其他石油制品 Other Petroleum Products	天然气 Natural Gas	热力 Heat	电力 Electricity	其他能源 Other Energy
**135.68**	**152.75**	**4024.81**	**1087.03**		**30.84**	**5189.66**		**4936.23**	**867.63**
						5514.18		5010.90	
								4345.06	
								620.26	
									867.63
414.93	400.59	4370.40	1098.87		503.62			41.79	
204.52	42.20	300.02							
-301.64	-92.74	-259.56	-5.47		-472.78	-324.52		-116.46	
-186.13	-33.51	-267.15							
3.99	-163.78	-118.90	-6.37						
**1415.84**	**13855.10**	**537.51**	**2413.01**	**1060.13**	**7960.74**	**-524.02**	**6567.20**	**22067.78**	**-414.64**
	-488.01	-2041.74	-3.77	-47.58	-64.41	-253.10		22067.78	-298.75
		-304.76	-12.34	-200.23	-91.91	-270.92	6567.20		-66.35
1415.84	14343.11	2899.03	2429.13	1307.94	8117.06				-49.54
		-15.01							
			**1.54**			**103.21**	**77.08**	**1745.92**	
**1560.95**	**13930.22**	**4472.15**	**3438.49**	**1050.72**	**7938.74**	**4649.13**	**6489.96**	**25256.91**	**452.04**
1.59	2585.34	0.94	8.78				2.51	994.10	
89.59	2433.21	2739.15	826.65	1050.72	6763.24	3359.58	4825.72	18230.61	452.04
10.45	45.99	132.76	90.19	17.29	3642.00	1015.06			38.33
	485.40	30.51	14.93		1175.51	18.49	14.24	273.01	
1206.12	6093.94	1643.53	60.03			66.90	41.67	552.62	
5.34	610.51	35.69	156.53			122.08	75.25	903.75	
40.26	165.63		2315.13			894.03	1411.56	3028.86	
3.03	121.98		1931.52			890.04	1411.56	1823.02	
37.22	43.64		383.61			3.99		1205.84	
218.05	1556.18	22.33	56.43			188.06	119.01	1273.96	
**-9.43**	**77.63**	**90.17**	**60.00**	**9.41**	**52.84**	**-86.70**	**0.16**	**1.18**	**0.95**

(C)1994-2024 China Academic Journal Electronic Publishing House. All rights reserved. http://www.cnki.net

4-6 中国能源平衡表(实物量) -2004

		煤合计 (万吨) Coal Total (10^4 tn)	原煤 (万吨) Raw Coal (10^4 tn)
**一.可供本地区消费的能源量**	**Total Primary Energy Supply**	**192265.48**	**192272.20**
1.一次能源生产量	Indigenous Production	199232.40	199232.40
水电	Hydro Power		
核电	Nuclear Power		
2.回收能	Recovery of Energy		
3.进口量	Import	1861.40	1142.29
4.我轮、机在外国加油量	China Airplanes&Ships Refueling in Abroad		
5.出口量(-)	Export (-)	-8666.36	-8083.88
6.外轮、机在我国加油量(-)	Foreign Airplanes&Ships Refueling in China		
7.库存增(-)、减(+)量	Stock Change	-161.96	-18.61
**二.加工转换投入(-)产出(+)量**	**Input(-) & Output(+) of Transformation**	**-134052.25**	**-142351.19**
1.火力发电	Thermal Power	-91961.56	-89868.15
2.供热	Heating Supply	-11546.56	-11333.54
3.洗选煤	Coal Washing	-3633.89	-35041.11
4.炼焦	Coking	-25349.58	-5122.00
5.炼油	Petroleum Refineries		
6.制气	Gas Works	-1316.43	-881.51
#焦炭再投入量(-)	Coke Input (-)		
7.煤制品加工	Briquettes	-244.23	-104.88
**三.损失量**	**Loss**		
**四.终端消费量**	**Total Final Consumption**	**59543.75**	**50881.44**
1.农、林、牧、渔、水利业	Farming, Forestry, Animal Husbandry, Fishery & Water Conservancy	2251.19	2226.39
2.工业	Industry	46082.95	39421.24
#用作原料、材料	Non-Energy Use	2916.13	2617.57
3.建筑业	Construction	601.53	593.89
4.交通运输、仓储及邮电通迅业	Transport, Storage, Postal & Telecommunications Services	832.12	826.89
5.批发和零售贸易业、餐饮业	Wholesale, Retail Trade and Catering Service	871.79	847.84
6.生活消费	Residential Consumption	8173.20	6298.52
城镇	Urban	2615.63	1733.21
乡村	Rural	5557.57	4565.31
7.其他	Other	730.97	666.66
**五.平衡差额**	**Statistical Difference**	**-1330.52**	**-960.43**
**六.消费量合计**	**Total Final Consumption**	**193596.00**	**193232.63**

(C)1994-2024 China Academic Journal Electronic Publishing House. All rights reserved. http://www.cnki.net

ENERGY BANLANCE OF CHINA －2004(PHYSICAL QUANTITY)

洗精煤 (万吨) Cleaned Coal (10⁴ tn)	其他洗煤 (万吨) Other Washed Coal (10⁴ tn)	型煤 (万吨) Briquettes (10⁴ tn)	焦炭 (万吨) Coke (10⁴ tn)	焦炉煤气 (亿立方米) Coke Oven Gas (10⁸ cu. m)	其他煤气 (亿立方米) Other Gas (10⁸ cu. m)	其他焦化产品 (万吨) Other Coking Products (10⁴ tn)	油品合计 (万吨) Petroleum Products Total (10⁴ tn)	原油 (万吨) Crude Oil (10⁴ tn)
**-40.25**	**38.64**	**-5.10**	**-2074.41**		**570.40**	**110.29**	**32116.21**	**29012.31**
							17587.32	17587.32
					570.40			
682.96	36.15		0.54			139.13	16913.35	12272.00
							377.97	
-575.65	-1.73	-5.10	-1501.20			-28.84	-1904.08	-549.16
							-336.50	
-147.56	4.22		-573.75				-521.85	-297.85
**1821.06**	**5351.96**	**1125.92**	**19743.28**	**299.33**	**5.61**	**284.67**	**-3488.18**	**-27755.56**
-156.58	-1936.83			-25.08	-115.80		-1864.07	-18.04
-11.75	-201.27			-14.22	-79.83		-418.47	-0.25
22569.66	8837.56							
-20162.67	-64.91		19629.78	334.40	20.27	272.30		
							-1195.13	-27737.27
-417.60	-17.32		307.80	4.23	180.97	18.97	-10.51	
			-194.30			-6.60		
	-1265.27	1125.92						
							**149.71**	**148.81**
**1997.95**	**5531.04**	**1133.32**	**17072.71**	**297.52**	**576.05**	**394.47**	**28062.02**	**844.94**
	24.80		98.69				2001.29	
1994.85	4525.15	141.71	16786.83	252.32	472.49	394.47	11344.10	844.94
102.54	190.06	5.97	823.00	2.88		100.39	3142.49	112.38
3.10	4.54		16.79				1422.31	
	5.23		1.79		0.21		8495.88	
	11.03	12.91	53.36	1.83	6.44		818.72	
	898.89	975.78	105.16	41.17	96.91		1778.05	
	343.09	539.33	54.65	41.17	95.78		1436.41	
	555.80	436.45	50.51		1.13		341.64	
	61.40	2.91	10.09	2.20			2201.68	
**-217.14**	**-140.45**	**-12.50**	**596.16**	**1.81**	**-0.04**	**0.49**	**416.30**	**263.00**
**22746.55**	**9016.64**	**1133.32**	**17267.01**	**336.82**	**771.68**	**401.07**	**31699.91**	**28749.31**

(C)1994-2024 China Academic Journal Electronic Publishing House. All rights reserved. http://www.cnki.net

续表

		汽油 (万吨) Gasoline (10^4 tn)	煤油 (万吨) Kerosene (10^4 tn)
**一.可供本地区消费的能源量**	**Total Primary Energy Supply**	**-568.08**	**92.21**
1.一次能源生产量	Indigenous Production		
水电	Hydro Power		
核电	Nuclear Power		
2.回收能	Recovery of Energy		
3.进口量	Import		282.00
4.我轮、机在外国加油量	China Airplanes&Ships Refueling in Abroad		139.00
5.出口量(-)	Export (-)	-540.71	-205.00
6.外轮、机在我国加油量(-)	Foreign Airplanes&ships Refueling in China		-126.50
7.库存增(-)、减(+)量	Stock Change	-27.37	2.71
**二.加工转换投入(-)产出(+)量**	**Input(-) & Output(+) of Transformation**	**5264.80**	**962.24**
1.火力发电	Thermal Power	-0.30	
2.供热	Heating Supply	-0.15	
3.洗选煤	Coal Washing		
4.炼焦	Coking		
5.炼油	Petroleum Refineries	5265.25	962.24
6.制气	Gas Works		
#焦炭再投入量(-)	Coke Input (-)		
7.煤制品加工	Briquettes		
**三.损失量**	**Loss**		
**四.终端消费量**	**Total Final Consumption**	**4695.31**	**1060.86**
1.农、林、牧、渔、水利业	Farming, Forestry, Animal Husbandry, Fishery & Water Conservancy	220.13	1.08
2.工业	Industry	506.95	60.89
#用作原料、材料	Non-Energy Use	16.27	7.10
3.建筑业	Construction	156.49	
4.交通运输、仓储及邮电通迅业	Transport, Storage, Postal & Telecommunications Services	2308.46	819.71
5.批发和零售贸易业、餐饮业	Wholesale, Retail Trade and Catering Service	279.80	3.63
6.生活消费	Residential Consumption	286.54	27.36
城 镇	Urban	223.92	2.06
乡 村	Rural	62.62	25.30
7.其他	Other	936.94	148.19
**五.平衡差额**	**Statistical Difference**	**1.41**	**-6.41**
**六.消费量合计**	**Total Final Consumption**	**4695.76**	**1060.86**

(C)1994-2024 China Academic Journal Electronic Publishing House. All rights reserved. http://www.cnki.net

Continued

柴油（万吨）Diesel Oil (10⁴tn)	燃料油（万吨）Fuel Oil (10⁴tn)	液化石油气（万吨）PLG (10⁴tn)	炼厂干气（万吨）Refinery Gas (10⁴tn)	其他石油制品（万吨）Other Petroleum Products (10⁴tn)	天然气（亿立方米）Natural Gas (10^8 cu. m)	热力（万百万千焦）Heat (10^{10} kj)	电力（亿千瓦小时）Electricity (10^8 kW·h)	其他能源（万吨标煤）Other Energy (10⁴tce)
**104.83**	**2817.31**	**634.09**		**23.53**	**390.20**		**4016.46**	**867.63**
					414.60		4077.22	
							3535.44	
							504.69	
								867.63
274.92	3059.22	641.00		384.21			34.00	
28.96	210.01							
-63.65	-181.69	-3.19		-360.68	-24.40		-94.76	
-23.00	-187.00							
-112.40	-83.23	-3.72						
**9508.68**	**376.25**	**1407.58**	**674.64**	**6073.19**	**-39.40**	**192586.50**	**17955.88**	**-414.64**
-334.92	-1429.19	-2.20	-30.28	-49.14	-19.03		17955.88	-298.75
	-213.33	-7.20	-127.42	-70.12	-20.37	192586.50		-66.35
9843.60	2029.28	1416.98	832.34	6192.45				-49.54
	-10.51							
		**0.90**			**7.76**	**2260.48**	**1420.60**	
**9560.23**	**3130.44**	**2005.77**	**668.65**	**6095.81**	**349.56**	**190321.46**	**20550.78**	**452.04**
1774.30	0.66	5.12				73.60	808.87	
1669.90	1917.37	482.21	668.65	5193.19	252.60	141516.66	14833.69	452.04
31.56	115.04	52.61	11.00	2796.53	76.32			38.33
333.13	21.36	8.71		902.62	1.39	417.58	222.14	
4182.24	1150.45	35.02			5.03	1221.92	449.65	
418.99	24.98	91.31			9.18	2206.70	735.35	
113.67		1350.48			67.22	41394.86	2464.49	
83.72		1126.71			66.92	41394.86	1483.33	
29.95		223.77			0.30		981.15	
1068.00	15.63	32.92			14.14	3490.14	1036.58	
**53.28**	**63.12**	**35.00**	**5.99**	**0.91**	**-6.52**	**4.57**	**0.96**	**0.95**
**9895.15**	**4783.47**	**2016.07**	**826.35**	**6215.07**	**396.72**	**192581.93**	**21971.38**	**866.68**

(C)1994-2024 China Academic Journal Electronic Publishing House. All rights reserved. http://www.cnki.net

4-7 综合能源平衡表

单位：万吨标准煤

项　目	Item	1980	1985
**可供消费的能源总量**	**Total Energy Available for Consumption**	**61557**	**77603**
一次能源生产量	Primary Energy Output	63735	85546
回收能	Recovery of Energy		
进口量	Imports	261	340
出口量(-)	Exports (-)	3058	5774
年初年末库存差额	Stock Changes in the Year	619	-2509
**能源消费总量**	**Total Energy Consumption**	**60275**	**76682**
在总量中:	Consumption by Sector		
1.农、林、牧、渔业	Farming, Forestry, Animal Husbandry, Fishery Conservancy	4692	4045
2.工 业	Industry	38986	51068
3.建筑业	Construction	957	1302
4.交通运输、仓储和邮政业	Transport, Storage and Post	2902	3713
5.批发、零售业和住宿、餐饮业	Wholesale, Retail Trade and Hotel, Restaurants	518	766
6.其他	Others	1205	2470
7.生活消费	Residential Consumption	11015	13318
在总量中:	Consumption by Usage		
(一) 终端消费	(I) Final Consumption	57508	73586
#工业	Industry	38293	48021
(二)加工转换损失量	(II) Losses in Processing and	1358	1491
#炼焦	Coking	644	572
炼油	Petroleum Refining	113	110
(三) 损失量	(III) Other Losses	1409	1605
**平衡差额**	**Balance**	**1282**	**921**

注:1. 村办工业包括在工业中(下同)。

2. 电力、热力按等价热值折算,因此加工转换损失量中不包括发电、供热损失量。

3. 进口量包括我国飞机、轮船在国外加油量;出口量包括外国飞机、轮船在我国加油量。

(C)1994-2024 China Academic Journal Electronic Publishing House. All rights reserved. http://www.cnki.net

OVERALL ENERGY BALANCE SHEET

(10 000 tce)

1990	1995	1998	1999	2000	2001	2002	2003	2004
**96138**	**129535**	**128368**	**132065**	**136535**	**140981**	**149082**	**172129**	**203344**
103922	129034	124250	125935	128978	137445	143810	163842	187341
	2312	1920	1694	1760	1859	1908	2043	2508
1310	5456	8474	9514	14334	13472	15769	20048	26593
5875	6776	7153	7051	9633	12020	11695	12989	11646
-3219	-491	878	1974	1097	225	-710	-814	-1452
**98703**	**131176**	**132214**	**133831**	**138553**	**143199**	**151797**	**174990**	**203227**
4852	5505	5790	5993	6045	6400	6612	6716	7680
67578	96191	94409	92840	95443	98273	104088	121771	143244
1213	1335	1612	1979	2143	2234	2544	2860	3259
4541	5863	8245	9340	10067	10363	11171	12819	15104
1247	2018	2552	2901	3039	3265	3520	4180	4820
3473	4519	5213	5563	5852	6096	6334	6819	7839
15799	15745	14393	15214	15965	16568	17527	19827	21281
94289	124252	126039	127814	132030	136486	144231	166633	194104
63239	89473	88522	87151	89266	91903	96864	113725	134442
2264	3634	2629	2228	2461	2325	2788	3378	3684
905		684	516	525	518	541	769	527
326		616	648	781	607	1015	1092	1416
2150	3289	3546	3789	4062	4388	4778	4979	5439
**-2565**	**-1641**	**-3846**	**-1766**	**-2017**	**-2218**	**-2716**	**-2862**	**117**

a) Data on industry include the data of village - run industry. (The sane as in the following tables).

b) Electric power and heat are converted on the basic of equal caloric value. Therefore, losses in processing and transformation exclude losses in power generation and heating.

c) Data on imports include the petroleum consumed by the Chinese airplanes and ships in refueling abroad. Data on exports include the petroleum consumed by the foreign airplanes and ships in refueling in China.

(C)1994-2024 China Academic Journal Electronic Publishing House. All rights reserved. http://www.cnki.net

4-8 煤炭平衡表

单位：万吨

项　目	Item	1980	1985
**可供量**	**Total Energy Available for Consumption**	**62601.0**	**82776.6**
生产量	Output	62015.0	87228.4
进口量	Imports	199.0	230.7
出口量(-)	Exports (-)	632.0	777.0
年初年末库存差额	Stock Changes in the Year	1019.0	-3905.5
**消费量**	**Total Energy Consumption**	**61009.5**	**81603.0**
在消费量中：	Consumption by Sector		
1. 农、林、牧、渔业	Farming, Forestry, Animal Husbandry, Fishery Conservancy	1550.3	2208.6
2. 工业	Industry	43848.4	58613.3
3. 建筑业	Construction	556.0	531.9
4. 交通运输、仓储和邮政业	Transport, Storage and Post	1934.4	2307.1
5. 批发、零售业和住宿、餐饮业	Wholesale, Retail Trade and Hotel, Restaurants	455.2	738.2
6. 其他	Other	1091.2	1579.5
7. 生活消费	Residential Consumption	11574.0	15624.4
在消费量中：	Consumption by Usage		
(一)终端消费	(1) Final Consumption	38804.2	52704.4
#工业	Industry	21643.1	29715.0
(二)中间消费	(2) Intermediate Consumption		
(用于加工转换)	(Consumed in Transformation)	19461.6	25397.4
发电	Power Generation	12648.4	16440.7
供热	Heating		1462.3
炼焦	Coking	6682.2	7303.8
制气	Gas Production	131.0	190.6
(三)洗选损耗	(3) Losses in Coal Washing and Dressing	2743.7	3501.2
**平衡差额**	**Balance**	**1591.5**	**1173.6**

注：生产量为原煤产量。

(C)1994-2024 China Academic Journal Electronic Publishing House. All rights reserved. http://www.cnki.net

COAL BALANCE SHEET

(10 000 ton)

1990	1995	1998	1999	2000	2001	2002	2003	2004
**102221.0**	**133461.7**	**122810.6**	**127076.1**	**128297.1**	**130554.0**	**137060.8**	**163402.0**	**192265.5**
107988.3	136073.1	125000.0	128000.0	129921.0	138152.0	145456.0	172200.0	199232.4
200.3	163.5	158.6	167.3	217.9	266.0	1125.7	1109.8	1861.4
1729.0	2861.7	3229.7	-3743.9	-5506.5	-9012.9	-8389.6	-9402.9	-8666.4
-4238.5	86.8	881.7	2652.7	3664.7	1148.9	-1131.4	-504.9	-162.0
**105523.0**	**137676.5**	**129492.2**	**130000.0**	**132000.0**	**135000.0**	**141600.5**	**169232.0**	**193596.0**
2095.2	1856.7	1923.3	1735.6	1647.7	1599.6	1622.9	1683.3	2251.2
81090.9	117570.7	114952.4	116500.0	119300.7	122518.3	129290.4	156168.5	180135.2
437.6	439.8	611.6	522.5	536.8	535.0	553.6	577.2	601.5
2160.9	1315.1	1390.6	1286.3	1132.2	1041.3	1055.0	1067.3	832.1
1058.3	977.4	947.6	896.2	814.6	810.9	809.1	860.4	871.8
1980.4	1986.7	782.7	651.1	661.0	664.7	667.1	700.6	731.0
16699.7	13530.1	8884.0	8408.4	7907.0	7830.3	7602.6	8174.7	8173.2
60205.9	66156.1	56347.1	49714.2	46821.4	45611.7	42572.4	49044.8	59543.8
35773.8	46050.3	41807.3	36214.2	34122.0	33129.9	30262.2	35981.2	46083.0
41257.8	69487.6	73145.1	80285.8	85178.6	89388.3	99028.1	120187.3	134052.3
27204.3	44440.2	49489.3	52458.2	55811.2	59797.9	68600.0	81976.5	91961.6
2995.5	5887.3	6319.9	7961.0	8794.1	8951.5	8973.7	10895.5	11546.6
10697.6	18396.4	15628.1	15931.7	16496.4	17236.4	18624.7	2699.3	25349.6
360.4	763.7	685.1	917.6	960.0	1002.1	973.2	23639.9	1316.4
4059.3	2032.8	1159.3	3091.5	3191.2	2450.5	1917.5	1054.8	3633.9
**-3302.0**	**-4214.8**	**-6681.6**	**-2923.9**	**-3702.9**	**-4446.0**	**-4539.8**	**-5830.1**	**-1330.5**

a) Data on output refer to the output of raw coal.

(C)1994-2024 China Academic Journal Electronic Publishing House. All rights reserved. http://www.cnki.net

4-9 焦炭平衡表

单位：万吨

项　　目	Item	1980	1985
**可供量**	**Total Energy Available for Consumption**	**4315.3**	**4689.7**
生产量	Output	4343.0	4802.1
进口量	Imports		2.1
出口量(-)	Exports (-)	27.1	36.9
年初年末库存差额	Stock Changes in the Year	-0.6	-77.6
**消费量**	**Total Energy Consumption**	**4303.0**	**4689.7**
在消费量中：	Consumption by Sector		
1. 农、林、牧、渔 业	Farming, Forestry, Animal Husbandry, Fishery Conservancy	10.6	20.8
2. 工 业	Industry	4266.7	4627.7
3. 建筑业	Construction	11.9	7.8
4. 交通运输、仓储和邮政业	Transport, Storage and Post	8.2	5.7
5. 批发、零售业和住宿、餐饮业	Wholesale, Retail Trade and Hotel, Restaurants	0.9	2.7
6. 其他	Other	4.7	2.0
7. 生活消费	Residential Consumption		23.0
在消费量中：	Consumption by Usage		
(一)终端消费	(1) Final Consumption	4294.7	4677.9
#工 业	Industry	4258.4	4615.9
(二)中间消费	(2) Intermediate Consumption		
(用于加工转换)	(Consumed in Transformation)	8.3	11.8
制 气	Gas Production	8.3	11.8
(三) 损失量	(3) Losses in Coal Washing and Dressing		
**平衡差额**	**Balance**	**12.3**	

(C)1994-2024 China Academic Journal Electronic Publishing House. All rights reserved. http://www.cnki.net

COKE BALANCE SHEET

(10 000 ton)

1990	1995	1998	1999	2000	2001	2002	2003	2004
**7085.8**	**12207.1**	**11733.5**	**10970.6**	**10892.3**	**11462.9**	**12830.5**	**15930.9**	**17863.2**
7328.3	13424.5	12899.1	12073.7	12184.0	13130.8	14253.3	17775.7	19937.6
	0.1						0.2	0.5
129.0	886.1	1146.4	997.4	1519.7	1384.6	1357.0	1472.1	1501.2
-113.5	-331.4	-19.2	-105.7	228.0	-283.2	-65.8	-372.9	-573.8
**6914.7**	**10725.3**	**11078.2**	**10460.5**	**10440.0**	**10999.2**	**12343.9**	**14503.8**	**17267.0**
60.1	128.6	151.4	145.8	144.2	139.2	141.0	141.0	98.7
6808.8	10412.0	10710.3	10094.9	10080.5	10638.4	11978.1	14149.8	16981.1
5.2	10.8	14.6	17.1	19.0	23.9	23.4	20.8	16.8
4.1	10.1	10.3	10.1	11.2	11.7	11.4	10.8	1.8
7.7	25.7	38.8	36.5	35.7	39.7	42.6	47.5	53.4
1.9	6.4	12.8	13.0	12.2	12.1	12.3	11.4	10.1
26.9	131.6	140.0	143.1	137.2	134.2	135.1	122.5	105.2
6846.3	10648.0	11008.6	10302.7	10297.1	10845.6	12206.6	14325.9	17072.7
6740.4	10334.7	10640.6	9937.1	9937.7	10484.8	11840.7	13972.0	16786.8
68.4	77.3	69.7	157.8	142.9	153.7	137.4	177.9	194.3
68.4	77.3	69.7	157.8	142.9	153.7	137.4	177.9	194.3
**171.1**	**1481.8**	**655.2**	**510.1**	**452.3**	**463.7**	**486.6**	**1427.1**	**596.2**

(C)1994-2024 China Academic Journal Electronic Publishing House. All rights reserved. http://www.cnki.net

4－10 石油平衡表

单位：万吨

项　　目	Item	1980	1985
**可供量**	**Total Energy Available for Consumption**	**8794.5**	**9193.7**
生产量	Output	10594.6	12489.5
进口量	Imports	82.7	90.0
出口量(－)	Exports (－)	1806.2	3630.4
年初年末库存差额	Stock Changes in the Year	－76.6	244.6
**消费量**	**Total Energy Consumption**	**8757.4**	**9168.8**
在消费量中：	Consumption by Sector		
1. 农、林、牧、渔 业	Farming, Forestry, Animal Husbandry, Fishery Conservancy	814..9	758.7
2. 工 业	Industry	6203.2	6171.4
3. 建筑业	Construction	175.2	292.2
4. 交通运输、仓储和邮政业	Transport, Storage and Post	911.5	1176.4
5. 批发、零售业和住宿 、餐饮业	Wholesale, Retail Trade and Hotel , Restaurants	29.0	38.1
6. 其他	Other	481.7	506.1
7. 生活消费	Residential Consumption	141.9	225.9
在消费量中：	Consumption by Usage	8757.4	
(一)终端消费	(I) Final Consumption	6311.0	7063.3
#工 业	Industry	3780.3	4462.0
(二) 中间消费	(II) Intermediate Consumption		
(用于加工转换)	(Consumed in Transformation)	2102.1	1745.6
发 电	Power Generation	2065.4	1425.5
供 热	Heating		285.6
制 气	Gas Production	36.7	34.5
(三) 炼油损失量	(III) Losses in Petroleum Refining	81.5	112.9
(四) 损失量	Other Losses	262.8	247.0
**平衡差额**	**Balance**	**37.1**	**24.9**

注：1. 生产量为原油产量。

2. 进口量包括我国飞机、轮船在国外加油量；出口量包括外国飞机、轮船在我国加油量。

(C)1994-2024 China Academic Journal Electronic Publishing House. All rights reserved. http://www.cnki.net

PETROLEUM BALANCE

(10 000 ton)

1990	1995	1998	1999	2000	2001	2002	2003	2004
**11435.0**	**16072.7**	**19686.1**	**20981.3**	**22631.8**	**23204.7**	**24925.1**	**27540.5**	**32116.2**
13830.6	15005.0	16100.0	16000.0	16300.0	16395.9	16700.0	16960.0	17587.3
755.6	3673.2	5738.7	6483.3	9748.5	9118.2	10269.3	13189.6	17291.3
3110.4	2454.5	2326.5	1643.5	2172.1	2046.7	2139.2	2540.8	2240.6
-40.8	-151.0	174.0	124.6	-1244.6	-262.7	94.9	-68.2	-521.9
**11485.6**	**16064.9**	**19817.8**	**21072.9**	**22439.3**	**22838.3**	**24786.8**	**27126.1**	**31699.9**
1033.6	1203.2	1294.7	1422.1	1496.9	1568.5	1674.1	1681.4	2001.3
7321.6	9349.3	10870.8	10428.7	10918.5	10827.2	11803.4	12886.5	14857.3
327.3	242.8	293.9	747.3	830.6	933.8	1096.6	1230.6	1422.3
1683.2	2863.6	4245.3	5004.3	5509.0	5692.9	6163.7	7093.2	8620.6
77.6	333.9	426.0	537.2	545.0	567.4	593.0	682.3	818.7
757.8	1390.3	1704.8	1800.3	1882.9	1953.7	1978.6	1916.4	2201.7
284.5	682.0	983.3	1133.1	1256.5	1294.8	1477.5	1635.8	1778.0
9304.7	13676.3	17514.3	18664.7	19893.5	20357.0	21989.8	24062.8	28062.0
5180.4	7095.5	8717.3	8172.1	8530.0	8498.4	9168.3	9958.7	11344.1
1630.4	2230.0	2106.1	2222.2	2352.9	2292.0	2606.8	2901.4	3488.2
1234.4	1358.5	1304.8	1228.6	1178.2	1213.6	1275.6	1491.6	1864.1
356.3	399.9	455.5	394.6	427.0	438.7	420.7	418.0	418.5
39.7	51.6	35.4	32.5	25.9	22.8	18.9	20.9	10.5
295.8	420.1	310.4	566.6	721.9	617.0	891.6	970.9	1195.1
254.7	158.6	197.3	186.0	192.9	189.3	190.2	161.9	149.7
**-50.6**	**7.8**	**-131.5**	**-108.5**	**192.5**	**366.4**	**138.3**	**414.4**	**416.3**

a) Data on output refer to the output of crude oil.

b) Data on imports include the petroleum consumed by the Chinese airplanes and ships in refueling abroad. Data on exports include the petroleum consumed by the foreign airplanes and ships in refueling in China.

(C)1994-2024 China Academic Journal Electronic Publishing House. All rights reserved. http://www.cnki.net

4-11 原油平衡表

单位：万吨

项 目	Item	1980	1985
**可供量**	**Total Energy Available for Consumption**	**9222.9**	**9516.5**
生产量	Output	10594.6	12489.5
进口量	Imports	36.6	
出口量(-)	Exports (-)	1330.9	3003.0
年初年末库存差额	Stock Changes in the Year	-77.4	30.0
**消费量**	**Total Energy Consumption**	**9205.0**	**9509.5**
在消费量中：	Consumption by Sector		
1. 农、林、牧、渔 业	Farming, Forestry, Animal Husbandry, Fishery Conservancy	8.0	0.8
2. 工 业	Industry	9112.0	9389.9
3.. 建筑业	Construction	28.8	74.0
4. 交通运输、仓储和邮政业	Transport, Storage and Post	50.1	44.3
5. 批发、零售业和住宿 、餐饮业	Wholesale, Retail Trade and Hotel , Restaurants		0.1
6. 其他	Other	6.1	0.4
7. 生活消费	Residential Consumption		
在消费量中：	Consumption by Usage		
(一)终端消费	(I) Final Consumption	499.6	350.4
#工 业	Industry	429.7	254.9
(二)中间消费	(II) Intermediate Consumption		
(用于加工转换)	(Consumed in Transformation)	8443.0	8929.7
发 电	Power Generation	574.0	279.5
供 热	Heating		61.3
炼油	Petroleum Refineries	7869.0	8588.9
(三) 油田原油损失量	(III) Losses in Oil Field for Crude Oil	262.4	229.4
**平衡差额**	**Balance**	**17.9**	**7.0**

(C)1994-2024 China Academic Journal Electronic Publishing House. All rights reserved. http://www.cnki.net

KEROSENE BALANCE SHEET

(10 000 ton)

1990	1995	1998	1999	2000	2001	2002	2003	2004
**11770.6**	**14794.9**	**17316.9**	**18947.2**	**21383.0**	**21537.2**	**22769.0**	**25187.1**	**29012.3**
13830.6	15004.4	16100.0	16000.0	16300.0	16395.9	16700.0	16960.0	17587.3
292.3	1709.0	2732.0	3661.4	7026.5	6026.0	6940.6	9102.0	12272.0
2399.0	1822.7	1560.0	716.7	1030.6	755.0	766.5	813.3	549.2
46.7	-95.8	44.9	2.6	-912.9	-129.7	-105.2	-61.6	-297.9
**11762.2**	**14886.4**	**17395.3**	**18949.5**	**21232.0**	**21342.7**	**22541.1**	**24922.0**	**28749.3**
0.2	10.1							
11653.8	14716.3	17222.5	18775.2	21052.1	21168.2	22357.5	24768.4	28625.5
55.2	2.7	2.2	3.2	3.3	3.4	4.2	4.0	
52.1	156.8	168.9	169.5	175.0	169.8	177.9	148.3	123.8
0.3	0.5	0.2	0.2	0.2	0.2	0.1	0.1	
0.6	1390.3	1.5	1.4	1.4	1.2	1.3	1.2	
402.1	309.9	518.7	519.4	636.8	654.1	681.3	812.2	844.9
333.4	274.7	495.4	495.4	612.3	630.8	658.0	793.0	844.9
11106.9	14419.4	16680.7	18245.7	20404.3	20500.7	21671.3	23949.0	27755.6
124.6	61.6	74.4	80.2	85.0	81.6	78.3	94.0	18.0
21.1	4.4	24.5	12.9	14.0	12.3	12.8	11.0	0.3
10961.2	14353.4	16581.8	18152.6	20305.3	20406.8	21580.2	23844.0	27737.3
253.2	157.1	196.0	184.4	190.9	187.9	188.5	160.8	148.8
**8.4**	**-91.5**	**-78.4**	**-2.2**	**151.0**	**194.4**	**227.9**	**265.1**	**263.0**

(C)1994-2024 China Academic Journal Electronic Publishing House. All rights reserved. http://www.cnki.net

4-12 燃料油平衡表

单位：万吨

项　目	Item	1980	1985
**可供量**	**Total Energy Available for Consumption**	**3096.1**	**2848.0**
生产量	Output	3142.0	2835.8
进口量	Imports	39.0	70.0
出口量(-)	Exports (-)	45.4	64.9
年初年末库存差额	Stock Changes in the Year	-39.5	7.1
**消费量**	**Total Energy Consumption**	**3073.7**	**2837.4**
在消费量中:	Consumption by Sector		
1. 农、林、牧、渔业	Farming, Forestry, Animal Husbandry, Fishery Conservancy	2.3	3.1
2. 工业	Industry	2937.4	2662.2
3. 建筑业	Construction	15.0	18.9
4. 交通运输、仓储和邮政业	Transport, Storage and Post	109.0	144.1
5. 批发、零售业和住宿、餐饮业	Wholesale, Retail Trade and Hotel, Restaurants	2.9	3.1
6. 其他	Other	7.1	6.0
7. 生活消费	Residential Consumption		
在消费量中:	Consumption by Usage		
(一)终端消费	(I) Final Consumption	1617.9	1538.8
#工业	Industry	1481.6	1363.5
(二)中间消费	(II) Intermediate Consumption		
(用于加工转换)	(Consumed in Transformation)	1455.8	1296.1
发电	Power Generation	1419.1	1042.3
供热	Heating		219.3
制气	Gas Production	36.7	34.5
(三)损失量	(III) Other Losses		2.5
**平衡差额**	**Balance**	**22.4**	**10.6**

(C)1994-2024 China Academic Journal Electronic Publishing House. All rights reserved. http://www.cnki.net

DIESEL OIL BALANCE SHEET

(10 000 ton)

1990	1995	1998	1999	2000	2001	2002	2003	2004
**3320.7**	**3717.3**	**3828.7**	**3901.1**	**3836.7**	**3836.9**	**3702.8**	**4291.1**	**4846.6**
3267.9	2960.8	2100.4	1959.4	2053.7	1864.4	1845.5	2004.8	2029.3
167.3	859.1	1818.3	1963.3	1704.3	2048.7	1915.2	2589.7	3269.2
97.2	68.6	72.9	38.8	57.9	84.9	104.9	188.7	368.7
-17.3	-34.0	-17.2	17.2	136.6	8.7	47.0	-114.7	-83.2
**3367.8**	**3693.7**	**3828.6**	**3934.1**	**3872.8**	**3850.2**	**3873.9**	**4220.5**	**4783.5**
2.9	8.4	0.3	0.4	0.4	0.4	0.4	0.6	0.7
3091.7	3406.2	3217.3	3047.7	2975.1	2949.3	2950.9	3236.8	3570.4
47.3	14.2	16.6	16.2	16.7	16.2	19.0	17.8	21.4
208.2	227.5	565.6	840.0	850.0	855.0	872.1	940.3	1150.4
1.6	6.6	7.4	10.5	11.6	12.3	12.3	13.0	25.0
16.1	30.8	21.4	19.4	19.0	17.0	19.1	12.1	15.6
2042.6	2262.8	2456.4	2694.0	2741.4	2689.3	2675.6	2882.1	3130.4
1766.5	1975.3	1845.1	1807.6	1843.7	1788.4	1752.6	1898.3	1917.4
1325.2	1430.9	1372.2	1240.1	1131.3	1160.9	1198.2	1338.5	1653.0
977.3	1071.5	993.8	906.3	814.2	838.5	912.7	1056.7	1429.2
308.3	307.8	343.0	301.3	291.2	299.6	266.7	260.8	213.3
39.6	51.6	35.4	32.5	25.9	22.8	18.9	20.9	10.5
**-47.1**	**23.6**	**0.1**	**-33.0**	**-36.1**	**-13.4**	**-171.1**	**70.6**	**63.1**

(C)1994-2024 China Academic Journal Electronic Publishing House. All rights reserved. http://www.cnki.net

4-13 汽油平衡表

单位：万吨

项　目	Item	1980	1985
**可供量**	**Total Energy Available for Consumption**	**999.4**	**1399.6**
生产量	Output	1079.0	1471.9
进口量	Imports		0.3
出口量(-)	Exports (-)	117.8	129.9
年初年末库存差额	Stock Changes in the Year	38.2	57.3
**消费量**	**Total Energy Consumption**	**998.6**	**1396.3**
在消费量中：	Consumption by Sector		
1. 农、林、牧、渔 业	Farming, Forestry, Animal Husbandry, Fishery Conservancy	53.3	122.3
2. 工 业	Industry	273.2	451.3
3. 建筑业	Construction	54.1	73.0
4. 交通运输、仓储和邮政业	Transport, Storage and Post	404.9	477.4
5. 批发、零售业和住宿、餐饮业	Wholesale, Retail Trade and Hotel, Restaurants	19.4	23.4
6. 其他	Other	193.7	238.3
7. 生活消费	Residential Consumption		10.6
**平衡差额**	**Balance**	**0.8**	**3.3**

4-14 煤油平衡表

单位：万吨

项　目	Item	1980	1985
**可供量**	**Total Energy Available for Consumption**	**359.0**	**383.2**
生产量	Output	398.5	405.3
进口量	Imports		15.2
出口量(-)	Exports (-)	46.8	46.0
年初年末库存差额	Stock Changes in the Year	2.3	8.7
**消费量**	**Total Energy Consumption**	**365.9**	**385.5**
在消费量中：	Consumption by Sector		
1. 农、林、牧、渔 业	Farming, Forestry, Animal Husbandry, Fishery Conservancy	2.3	3.3
2. 工 业	Industry	15.7	20.1
3. 建筑业	Construction	0.8	1.3
4. 交通运输、仓储和邮政业	Transport, Storage and Post	31.4	56.2
5. 批发、零售业和住宿、餐饮业	Wholesale, Retail Trade and Hotel, Restaurants	0.2	0.1
6. 其他	Other	216.7	182.9
7. 生活消费	Residential Consumption	98.8	121.6
**平衡差额**	**Balance**	**-6.9**	**-2.3**

(C)1994-2024 China Academic Journal Electronic Publishing House. All rights reserved.　http://www.cnki.net

GASOLINE BALANCE SHEET

(10 000 ton)

1990	1995	1998	1999	2000	2001	2002	2003	2004
**1884.1**	**2902.0**	**3307.6**	**3379.8**	**3504.5**	**3606.9**	**3749.7**	**4072.3**	**4697.2**
2173.4	3051.6	3501.0	3741.3	4134.7	4154.7	4320.8	4790.9	5265.3
16.9	15.9	1.5						
233.8	193.1	194.5	425.8	467.7	586.0	630.4	754.2	540.7
-72.4	27.6	-0.5	64.3	-162.5	38.1	59.3	35.7	-27.4
**1899.5**	**2909.6**	**3328.6**	**3380.7**	**3504.9**	**3597.8**	**3749.7**	**4072.0**	**4695.8**
145.9	179.7	172.6	178.1	184.5	190.6	187.9	195.0	220.1
589.3	812.4	677.5	646.5	602.0	618.1	631.6	617.9	507.4
89.5	103.6	112.6	113.8	115.6	116.7	122.3	123.7	156.5
620.1	982.3	1216.6	1265.5	1387.8	1419.4	1503.5	1861.6	2308.5
46.0	197.2	216.5	206.3	209.8	214.0	224.2	238.1	279.8
390.7	570.7	825.7	849.4	877.7	904.3	916.3	837.0	936.9
18.0	63.7	107.1	121.1	127.6	134.6	163.8	198.8	286.5
**-15.4**	**-7.6**	**-21.1**	**-0.9**	**-0.4**	**9.1**		**0.3**	**1.4**

KEROSENE BALANCE SHEET

(10 000 ton)

1990	1995	1998	1999	2000	2001	2002	2003	2004
**350.9**	**486.4**	**699.9**	**848.6**	**880.9**	**894.2**	**914.8**	**914.5**	**1054.5**
392.5	445.8	616.1	743.8	872.3	789.4	826.1	855.3	962.2
26.1	115.7	188.0	272.2	322.5	298.6	324.3	317.4	421.0
55.5	62.4	127.6	162.8	256.3	246.4	240.9	275.9	331.5
-12.2	-12.7	23.3	-4.6	-57.6	52.7	5.3	17.7	2.7
**350.9**	**512.1**	**671.4**	**824.2**	**869.6**	**890.3**	**919.2**	**921.6**	**1060.9**
3.1	3.6	1.6	1.4	1.5	1.5	1.4	1.4	1.1
20.6	44.9	62.2	78.4	84.0	86.0	87.4	87.8	60.9
1.3	3.5	3.5	3.9	4.0	3.5			
93.4	250.0	390.5	505.6	535.9	560.7	616.7	621.7	819.7
0.6	8.5	9.0	11.5	12.0	12.5	13.0	11.2	3.6
127.3	137.3	141.6	152.7	160.1	151.1	140.0	143.2	148.2
104.6	64.3	63.1	70.8	72.2	75.0	60.7	56.4	27.4
	**-25.7**	**28.5**	**24.3**	**11.3**	**4.0**	**-4.4**	**-7.1**	**-6.4**

(C)1994-2024 China Academic Journal Electronic Publishing House. All rights reserved. http://www.cnki.net

4－15 柴油平衡表

单位：万吨

项　目	Item	1980	1985
可供量	Total Energy Available for Consumption	1663.2	1944.1
生产量	Output	1827.8	2023.2
进口量	Imports	2.1	4.5
出口量(－)	Exports (－)	166.5	225.6
年初年末库存差额	Stock Changes in the Year	-0.2	142.0
消费量	Total Energy Consumption	1663.2	1939.4
在消费量中：	Consumption by Sector		
1. 农、林、牧、渔 业	Farming, Forestry, Animal Husbandry, Fishery Conservancy	749.0	629.2
2. 工 业	Industry	457.4	644.1
3. 建筑业	Construction	76.5	125.0
4. 交通运输、仓储和邮政业	Transport, Storage and Post	316.1	454.4
5. 批发、零售业和住宿 、餐饮业	Wholesale, Retail Trade and Hotel , Restaurants	6.5	10.9
6. 其他	Other	57.7	74.0
7. 生活消费	Residential Consumption		
在消费量中：	Consumption by Usage		
(一)终端消费	(I) Final Consumption	1590.9	1827.4
#工 业	Industry	385.1	532.1
(二) 中间消费	(II) Intermediate Consumption		
(用于加工转换)	(Consumed in Transformation)	72.3	108.6
发 电	Power Generation	72.3	103.6
供 热	Heating		5.0
(三) 损失量	Other Losses		3.4
平衡差额	Balance		4.7

(C)1994-2024 China Academic Journal Electronic Publishing House. All rights reserved. http://www.cnki.net

DIESEL OIL BALANCE SHEET

(10 000 ton)

1990	1995	1998	1999	2000	2001	2002	2003	2004
**2689.4**	**4404.2**	**5229.8**	**6204.3**	**6806.5**	**7272.0**	**7722.3**	**8467.5**	**9948.4**
2609.0	3972.6	4897.7	6172.6	7079.6	7485.6	7706.1	8532.8	9843.6
233.8	645.3	331.7	56.0	51.9	54.7	78.7	111.6	303.9
169.8	169.5	118.8	70.9	77.5	46.9	144.7	244.4	86.7
16.4	-44.2	119.2	46.5	-247.6	-221.4	82.2	67.5	-112.4
**2691.7**	**4321.4**	**5282.8**	**6231.6**	**6774.3**	**7107.7**	**7667.2**	**8409.8**	**9895.2**
881.5	1001.4	1120.2	1241.8	1310.1	1375.6	1484.3	1484.4	1774.3
728.1	1189.9	1346.1	1506.8	1596.5	1636.8	1731.3	1830.5	2004.8
133.0	118.2	153.5	178.1	195.9	223.1	252.0	276.2	333.1
709.4	1246.6	1901.9	2221.7	2543.8	2671.0	2964.8	3485.2	4182.2
22.5	103.6	153.3	260.9	255.9	268.1	280.8	355.5	419.0
217.0	645.7	564.0	759.7	803.7	853.9	870.0	890.0	1068.0
	16.1	43.9	62.7	68.4	79.2	83.9	87.9	113.7
2564.8	4070.0	5078.9	6016.2	6546.6	6867.3	7441.2	8138.5	9560.2
601.2	938.5	1142.2	1291.4	1368.8	1396.4	1505.3	1559.2	1669.9
126.9	251.4	203.9	215.5	227.7	240.4	226.0	271.3	334.9
124.5	204.9	203.9	215.5	227.7	240.4	226.0	271.3	334.9
2.4	46.6							
**-2.3**	**82.7**	**-52.9**	**-27.4**	**32.2**	**164.4**	**55.2**	**57.8**	**53.3**

(C)1994-2024 China Academic Journal Electronic Publishing House. All rights reserved. http://www.cnki.net

4-16 液化石油气平衡表

单位：万吨

项　目	Item	1980	1985
**可供量**	**Total Energy Available for Consumption**	**122.5**	**157.3**
生产量	Output	122.5	159.7
进口量	Imports		
出口量(-)	Exports (-)		1.9
年初年末库存差额	Stock Changes in the Year		-0.5
**消费量**	**Total Energy Consumption**	**119.6**	**155.7**
在消费量中：	Consumption by Sector		
1. 农、林、牧、渔 业	Farming, Forestry, Animal Husbandry, Fishery Conservancy		
2. 工 业	Industry	76.1	59.9
3. 建筑业	Construction		
4. 交通运输、仓储和邮政业	Transport, Storage and Post		
5. 批发、零售业和住宿 、餐饮业	Wholesale, Retail Trade and Hotel ,Restaurants		0.5
6. 其他	Other	0.4	4.5
7. 生活消费	Residential Consumption	43.1	90.8
**平衡差额**	**Balance**	**2.9**	**1.6**

4-17 天然气平衡表

单位：万吨

项　目	Item	1980	1985
**可供量**	**Total Energy Available for Consumption**	**142.7**	**129.3**
生产量	Output	142.7	129.3
进口量	Imports		
出口量(-)	Exports (-)		
年初年末库存差额	Stock Changes in the Year		
**消费量**	**Total Energy Consumption**	**140.6**	**129.3**
在消费量中：	Consumption by Sector		
1. 农、林、牧、渔 业	Farming, Forestry, Animal Husbandry, Fishery Conservancy		
2. 工 业	Industry	131.4	109.6
3. 建筑业	Construction	6.0	14.1
4. 交通运输、仓储和邮政业	Transport, Storage and Post	0.7	0.8
5. 批发、零售业和住宿 、餐饮业	Wholesale, Retail Trade and Hotel ,Restaurants		
6. 其他	Other	0.5	0.5
7. 生活消费	Residential Consumption	2.0	4.3
**平衡差额**	**Balance**	**2.1**	

(C)1994-2024 China Academic Journal Electronic Publishing House. All rights reserved.　http://www.cnki.net

LPG BALANCE SHEET

(10 000 ton)

1990	1995	1998	1999	2000	2001	2002	2003	2004
**258.5**	**774.3**	**1178.0**	**1146.6**	**1396.2**	**1428.0**	**1663.6**	**1836.2**	**2051.1**
261.6	540.8	747.4	816.6	916.6	952.3	1036.8	1211.7	1417.0
	232.6	476.6	322.3	481.7	488.9	626.2	636.7	641.0
1.1	7.1	50.2	7.5	1.6	2.1	5.6	2.4	3.2
-2.0	8.0	4.2	-1.5	-0.6	-11.1	6.3	-9.9	-3.7
**254.2**	**750.6**	**1186.0**	**1208.5**	**1366.7**	**1411.0**	**1625.4**	**1796.6**	**2016.1**
	0.1			0.4	0.3			5.1
82.0	192.5	220.5	254.0	276.1	291.7	320.5	361.6	491.6
1.0	0.5	5.7	7.9	8.9	9.5	12.8	8.9	8.7
	0.5	0.5	2.0	16.5	17.0	28.6	36.1	35.9
6.6	17.4	39.5	47.9	55.5	60.4	62.6	64.4	91.3
6.1	5.7	150.6	17.9	21.0	26.2	31.9	32.9	32.9
158.5	534.0	769.2	878.5	988.3	1006.0	1169.1	1292.7	1350.5
**4.3**	**23.7**	**-8.0**	**-61.6**	**29.5**	**16.9**	**38.2**	**39.6**	**35.0**

NATURAL GAS BALANCE SHEET

(100 million cu. m)

1990	1995	1998	1999	2000	2001	2002	2003	2004
**153.0**	**179.5**	**232.8**	**218.2**	**240.6**	**272.9**	**294.6**	**331.42**	**390.2**
153.0	179.5	232.8	252.0	272.0	303.3	326.6	350.15	414.6
			33.8	31.4	30.4	32.0	18.73	24.4
**152.5**	**177.4**	**202.6**	**214.9**	**245.0**	**274.3**	**291.8**	**339.1**	**396.7**
120.2	154.4	171.5	180.2	202.0	217.8	227.5	267.8	293.6
10.6	0.3	0.1	0.7	0.8	0.7	0.7	0.7	1.4
1.9	1.6	3.7	4.8	5.8	6.0	6.4	6.8	11.2
	0.6	2.5	2.9	3.4	5.0	6.1	6.9	9.2
1.2	1.2	0.6	0.6	0.6	0.7			14.1
18.6	19.4	24.1	25.7	32.3	44.1	51.2	56.9	67.2
**0.5**	**2.1**	**30.2**	**3.3**	**-4.4**	**-1.4**	**2.7**	**-7.7**	**-6.5**

(C)1994-2024 China Academic Journal Electronic Publishing House. All rights reserved. http://www.cnki.net

4-18 电力平衡表

单位：亿千瓦小时

项　目	Item	1980	1985
**可供量**	**Total Energy Available for Consumption**	**3006.3**	**4117.6**
生产量	Output	3006.3	4106.9
水电	Hydropower	582.1	923.7
火电	Thermal Power	2424.2	3183.2
核电	Nuclear Power		
进口量	Imports		11.1
出口量(-)	Exports (-)		0.4
**消费量**	**Total Energy Consumption**	**3006.3**	**4117.6**
在消费量中：	Consumption by Sector		
1. 农、林、牧、渔业	Farming, Forestry, Animal Husbandry, Fishery Conservancy	270	317.4
2. 工业	Industry	2471.9	3283.4
3. 建筑业	Construction	47.1	71.2
4. 交通运输、仓储和邮政业	Transport, Storage and Post	26.5	63.4
5. 批发、零售业和住宿、餐饮业	Wholesale, Retail Trade and Hotel, Restaurants	16.8	38.0
6. 其他	Others	68.8	121.7
7. 生活消费	Residential Consumption	105.2	222.5
在消费量中：	Consumption by Usage		
(一)终端消费	(I) Final Consumption	2763.4	3813.3
#工业	Industry	2229.0	2979.1
(二)输配电损失量	(II) Losses in Transmission	242.9	304.3

(C)1994-2024 China Academic Journal Electronic Publishing House. All rights reserved.   http://www.cnki.net

ELECTRICITY BALANCE

(100 million kW · h)

1990	1995	1998	1999	2000	2001	2002	2003	2004
**6230.4**	**10023.4**	**11590.4**	**12305.2**	**13472.7**	**14724.1**	**16466.0**	**19032.2**	**21972.3**
6212.0	10077.3	11662.0	12393.0	13556.0	14808.0	16540.0	19105.8	22033.1
1267.2	1905.8	2080.0	2038.1	2224.1	2774.3	2879.7	2836.8	3535.4
4944.8	8043.2	9441.0	10205.4	11141.9	11834.3	13381.4	15803.6	17955.9
	128.3	141.0	149.5	167.4	174.7	251.3	433.4	504.7
19.3	6.4	0.2	3.7	15.5	18.0	23.0	29.8	34.0
0.9	60.3	71.7	91.5	98.8	101.9	97.0	103.4	94.8
**6230.4**	**10023.4**	**11598.4**	**12305.2**	**13471.4**	**14723.5**	**16465.5**	**19031.6**	**21971.4**
426.8	582.4	623.5	660.4	673.0	762.4	776.2	773.2	808.9
4873.3	7659.8	8406.0	8832.7	9653.6	10534.7	11927.2	13899.7	16254.3
65.0	159.6	188.8	142.3	154.8	144.9	164.1	189.8	222.1
105.9	182.3	255.6	254.8	281.2	309.3	338.0	396.9	449.6
76.2	199.5	293.4	342.8	393.7	444.9	500.0	623.0	735.4
202.4	234.2	506.7	591.4	643.2	688.1	758.5	911.0	1036.6
480.8	1005.6	1324.5	1480.8	1672.0	1839.2	2001.4	2238.0	2464.5
5795.8	9278.9	10807.5	11443.3	12534.7	13690.0	15296.8	17770.9	20550.8
4438.7	6915.3	7615.0	7970.8	8716.9	9501.2	10758.5	12639.0	14833.7
434.6	744.5	790.9	861.9	936.7	1033.5	1168.7	1260.7	1420.6

(C)1994-2024 China Academic Journal Electronic Publishing House. All rights reserved. http://www.cnki.net

5-1 中国能源平衡表(实物量) -2010

项 目	Item	煤合计 (万吨) Coal Total (10^4 tn)	原煤 (万吨) Raw Coal (10^4 tn)
**一.可供本地区消费的能源量**	**Total Primary Energy Supply**	**319771.98**	**322524.47**
1.一次能源生产量	Indigenous Production	323500.00	323500.00
水电	Hydro Power		
核电	Nuclear Power		
风电	Wind Power		
2.回收能	Recovery of Energy		
3.进口量	Import	16309.52	16306.50
4.境内轮船和飞机在境外的加油量	Domestic Airplanes&Ships Refueling in Abroad		
5.出口量(-)	Export (-)	-1910.37	-1903.00
6.境外轮船和飞机在境内的加油量(-)	Oversea Airplanes&Ships Refueling in China		
7.库存增(-)、减(+)量	Stock Change	-18127.17	-15379.03
**二.加工转换投入(-)产出(+)量**	**Input(-) & Output(+) of Transformation**	**-227885.57**	**-242393.85**
1.火力发电	Thermal Power	-154542.48	-151307.09
2.供 热	Heating Supply	-15253.08	-14565.47
3.洗 选 煤	Coal Washing	-9484.62	-69758.46
4.炼 焦	Coking	-47150.37	-5842.10
5.炼油及煤制油	Petroleum Refineries	-213.42	-114.30
#油品再投入量(-)	Petroleum Products Input (-)		
6.制 气	Gas Works	-1040.06	-603.38
#焦炭再投入量(-)	Coke Input (-)		
7.天然气液化	Natural Gas Liquefaction		
8.煤制品加工	Briquettes	-201.54	-203.05
**三.损 失 量**	**Loss**		
**四.终端消费量**	**Total Final Consumption**	**84350.93**	**73864.92**
1.农、林、牧、渔业	Farming, Forestry, Animal Husbandry, Fishery Conservancy	1711.10	1671.35
2.工业	Industry	68146.05	59538.89
#用作原料、材料	Non-Energy Use	4289.25	3915.52
3.建筑业	Construction	718.92	707.13
4.交通运输、仓储和邮政业	Transport, Storage and Post	639.23	612.99
5.批发、零售业和住宿、餐饮业	Wholesale, Retail Trade and Hotel, Restaurants	1969.87	1897.89
6.其他	Others	2006.59	1818.03
7.生活消费	Residential Consumption	9159.17	7618.64
城 镇	Urban	1902.68	1403.17
乡 村	Rural	7256.49	6215.47
**五.平衡差额**	**Statistical Difference**	**7535.48**	**6265.71**
**六.消费量合计**	**Total Final Consumption**	**312236.50**	**316258.76**

?1994-2018 China Academic Journal Electronic Publishing House. All rights reserved. http://www.cnki.net

ENERGY BANLANCE OF CHINA -2010(PHYSICAL QUANTITY)

洗精煤 (万吨) Cleaned Coal (10^4 tn)	其他洗煤 (万吨) Other Washed Coal (10^4 tn)	型煤 (万吨) Briquettes (10^4 tn)	煤矸石 (万吨) Gangue (10^4 tn)	焦炭 (万吨) Coke (10^4 tn)	焦炉煤气 (亿立方米) Coke Oven Gas (10^8 cu.m)	高炉煤气 (亿立方米) Blast Furnace Gas (10^8 cu.m)	转炉煤气 (亿立方米) Converter Gas (10^8 cu.m)	其他煤气 (亿立方米) Other Gas (10^8 cu.m)
**-938.55**	**-1805.06**	**-8.88**		**-1950.74**		**2270.61**	**255.86**	
						2270.61	255.86	
		3.02		10.95				
		-7.37		-335.00				
-938.55	-1805.06	-4.53		-1626.69				
**4152.31**	**9268.46**	**1087.51**	**-0.08**	**36329.59**	**563.84**	**-1152.33**	**-78.45**	**174.50**
-4.69	-3230.70		-2226.54		-105.48	-792.00	-46.15	
-10.53	-677.08		-470.96		-72.83	-360.33	-32.30	
45949.21	14324.62		2697.42					
-41251.77	-56.49			36209.60	718.38			
-93.23	-5.89							
-436.68				248.23	23.77			174.50
				-128.24				
	-1086.00	1087.51						
**2541.78**	**6905.45**	**1038.79**		**33559.56**	**551.29**	**1110.00**	**179.02**	**168.61**
	39.75			46.82				
2529.37	5667.70	410.09		33455.45	494.55	1110.00	179.02	55.00
130.01	243.71			1338.45	4.95			
1.36	10.43			5.81				
11.05	15.19			0.12				
	39.13	32.86		5.10	0.80			
	177.11	11.46		2.77	2.68			
	956.15	584.38		43.49	53.26			113.61
	322.85	176.65		23.72	53.26			113.17
	633.30	407.73		19.77				0.44
**671.98**	**557.95**	**39.84**	**-0.08**	**819.29**	**12.55**	**8.28**	**-1.61**	**5.89**
**44338.68**	**11961.61**	**1038.79**	**2697.50**	**33687.80**	**705.83**	**2262.33**	**257.47**	**168.61**

?1994-2018 China Academic Journal Electronic Publishing House. All rights reserved. http://www.cnki.net

续表 1

项　　目	Item	其他焦化产品 (万吨) Other Coking Products (10^4 tn)	油品合计 (万吨) Petroleum Products Total (10^4 tn)
**一.可供本地区消费的能源量**	**Total Primary Energy Supply**		**44178.43**
1.一次能源生产量	Indigenous Production		20301.40
水电	Hydro Power		
核电	Nuclear Power		
风电	Wind Power		
2.回收能	Recovery of Energy		
3.进口量	Import		28791.77
4.境内轮船和飞机在境外的加油量	Domestic Airplanes&Ships Refueling in Abroad		645.45
5.出口量(−)	Export (-)		-3357.54
6.境外轮船和飞机在境内的加油量(−)	Oversea Airplanes&Ships Refueling in China		-721.46
7.库存增(−)、减(+)量	Stock Change		-1481.19
**二.加工转换投入(−)产出(+)量**	**Input(-) & Output(+) of Transformation**	**682.68**	**-2657.06**
1.火力发电	Thermal Power		-459.14
2.供　　热	Heating Supply		-593.12
3.洗 选 煤	Coal Washing		
4.炼　　焦	Coking	693.31	
5.炼油及煤制油	Petroleum Refineries	-44.25	2264.01
#油品再投入量(−)	Petroleum Products Input (-)		-3868.81
6.制　　气	Gas Works	44.85	
#焦炭再投入量(−)	Coke Input (-)	-11.23	
7.天然气液化	Natural Gas Liquefaction		
8.煤制品加工	Briquettes		
**三.损 失 量**	**Loss**		**194.40**
**四.终端消费量**	**Total Final Consumption**	**673.00**	**40393.70**
1.农、林、牧、渔业	Farming,Forestry,Animal Husbandry, Fishery Conservancy		1382.50
2.工业	Industry	673.00	14757.76
#用作原料、材料	Non-Energy Use		4246.75
3.建筑业	Construction		3045.11
4.交通运输、仓储和邮政业	Transport, Storage and Post		14709.86
5.批发、零售业和住宿、餐饮业	Wholesale, Retail Trade and Hotel, Restaurants		481.01
6.其他	Others		2556.66
7.生活消费	Residential Consumption		3460.80
城 镇	Urban		2517.38
乡 村	Rural		943.41
**五.平衡差额**	**Statistical Difference**	**9.68**	**933.28**
**六.消费量合计**	**Total Final Consumption**	**728.48**	**43245.15**

?1994-2018 China Academic Journal Electronic Publishing House. All rights reserved. http://www.cnki.net

Continued 1

原油 (万吨) Crude Oil (10^4 tn)	汽油 (万吨) Gasoline (10^4 tn)	煤油 (万吨) Kerosene (10^4 tn)	柴油 (万吨) Diesel Oil (10^4 tn)	燃料油 (万吨) Fuel Oil (10^4 tn)	石脑油 (万吨) Naphtha (10^4 tn)	润滑油 (万吨) Lubricants (10^4 tn)	石蜡 (万吨) Petroleum Waxes (10^4 tn)	溶剂油 (万吨) White Spirit (10^4 tn)
**42876.63**	**-446.17**	**-156.77**	**-222.52**	**1278.47**	**3.70**	**22.95**	**-51.10**	**-0.17**
20301.40								
23768.18	0.01	487.00	180.00	2299.00	290.70	34.20	0.90	3.36
		239.06	10.19	396.20				
-303.00	-517.00	-605.00	-464.00	-990.00	-87.00	-11.25	-52.00	-0.53
		-265.50	-26.24	-429.72				
-889.95	70.82	-12.33	77.53	2.99	-200.00			-3.00
**-41876.43**	**7360.38**	**1921.73**	**14806.74**	**1182.13**	**1931.57**	**218.94**	**160.11**	**74.33**
-3.71	-0.09		-113.86	-123.89				
-3.28			-3.78	-201.34				
-41869.44	7360.47	1924.39	14924.38	2536.97	1940.65	218.94	160.11	74.33
		-2.66		-1029.61	-9.08			
**192.00**								
**806.12**	**6886.12**	**1741.41**	**14516.17**	**2403.18**	**1841.02**	**225.00**	**103.00**	**70.00**
	169.07	0.90	1206.73	1.14				
806.12	689.37	37.54	2046.16	1022.48	1841.02	225.00	103.00	70.00
107.21	22.13				290.00	218.66	101.08	67.23
	274.70	8.77	490.20	30.76				
	3204.93	1601.08	8518.56	1326.65				
	168.18	34.98	196.60	8.62				
	1166.22	38.73	1287.19	13.53				
	1213.65	19.41	770.73					
	844.66	1.69	573.44					
	369.00	17.72	197.29					
**2.08**	**28.09**	**23.55**	**68.06**	**57.42**	**94.25**	**16.89**	**6.01**	**4.16**
**42874.55**	**6886.21**	**1744.07**	**14633.80**	**3758.02**	**1850.10**	**225.00**	**103.00**	**70.00**

?1994-2018 China Academic Journal Electronic Publishing House. All rights reserved. http://www.cnki.net

续表 2

项　目	Item	石油沥青(万吨) Bitumen Asphalt (10^4 tn)	石油焦(万吨) Petroleum Coke (10^4 tn)
**一.可供本地区消费的能源量**	**Total Primary Energy Supply**	**392.70**	**443.66**
1.一次能源生产量	Indigenous Production		
水电	Hydro Power		
核电	Nuclear Power		
风电	Wind Power		
2.回收能	Recovery of Energy		
3.进口量	Import	407.70	773.86
4.境内轮船和飞机在境外的加油量	Domestic Airplanes&Ships Refueling in Abroad		
5.出口量(-)	Export (-)	-15.00	-210.20
6.境外轮船和飞机在境内的加油量(-)	Oversea Airplanes&Ships Refueling in China		
7.库存增(-)、减(+)量	Stock Change		-120.00
**二.加工转换投入(-)产出(+)量**	**Input(-) & Output(+) of Transformation**	**1963.90**	**1399.46**
1.火力发电	Thermal Power		-109.47
2.供　热	Heating Supply		-115.58
3.洗 选 煤	Coal Washing		
4.炼　焦	Coking		
5.炼油及煤制油	Petroleum Refineries	1963.90	1624.51
#油品再投入量(-)	Petroleum Products Input (-)		
6.制　气	Gas Works		
#焦炭再投入量(-)	Coke Input (-)		
7.天然气液化	Natural Gas Liquefaction		
8.煤制品加工	Briquettes		
**三.损 失 量**	**Loss**		
**四.终端消费量**	**Total Final Consumption**	**2218.37**	**1800.00**
1.农、林、牧、渔业	Farming, Forestry, Animal Husbandry, Fishery Conservancy		
2.工业	Industry	44.87	1800.00
#用作原料、材料	Non-Energy Use		1582.71
3.建筑业	Construction	2173.50	
4.交通运输、仓储和邮政业	Transport, Storage and Post		
5.批发、零售业和住宿、餐饮业	Wholesale, Retail Trade and Hotel, Restaurants		
6.其他	Others		
7.生活消费	Residential Consumption		
城 镇	Urban		
乡 村	Rural		
**五.平衡差额**	**Statistical Difference**	**138.23**	**43.12**
**六.消费量合计**	**Total Final Consumption**	**2218.37**	**2025.05**

?1994-2018 China Academic Journal Electronic Publishing House. All rights reserved.   http://www.cnki.net

Continued 2

液化石油气<br>(万吨)<br>LPG<br>(10^4 tn)	炼厂干气<br>(万吨)<br>Refinery Gas<br>(10^4 tn)	其他石油制品<br>(万吨)<br>Other Petroleum Products<br>(10^4 tn)	天然气<br>(亿立方米)<br>Natural Gas<br>(10^8 cu.m)	液化天然气<br>(万吨)<br>LNG<br>(10^4 tn)	热力<br>(万百万千焦)<br>Heat<br>(10^{10} kJ)	电力<br>(亿千瓦小时)<br>Electricity<br>(10^8 kW•h)	其他能源<br>(万吨标煤)<br>Other Energy<br>(10^4tce)
**231.55**		**-194.50**	**943.98**	**934.30**	**28465.63**	**8617.18**	**558.00**
			948.48			8752.32	
						7221.72	
						738.80	
						446.22	
					28465.63		558.00
327.00		219.86	35.80	934.30		55.45	
-93.00		-9.56	-40.30			-190.59	
-2.45		-404.80					
**2044.57**	**1123.96**	**5031.55**	**-200.99**	**-130.52**	**269355.59**	**33319.28**	**-556.89**
	-74.01	-34.11	-161.78	-167.69	-24067.15	33319.28	-378.85
-0.88	-175.78	-92.48	-27.96	-8.78	293422.74		-174.28
2102.27	1460.71	7841.82					-3.76
-56.82	-86.96	-2683.68					
			-3.92				
			-7.33	45.95			
**2.40**			**17.11**	**14.14**	**3400.00**	**2568.24**	
**2180.20**	**1102.44**	**4500.68**	**723.57**	**781.00**	**294418.28**	**39366.25**	
4.66			0.50		91.01	976.49	
529.08	1102.44	4440.68	362.07	655.00	213189.00	28303.53	
57.72		1800.00	90.52	163.75			
7.18		60.00	1.16		661.74	483.24	
58.64			79.71	126.00	1637.94	734.53	
72.63			27.24		3902.19	1292.00	
51.00			26.00		7526.18	2451.83	
1457.01			226.90		67410.23	5124.63	
1097.60			226.22		67410.23	2988.06	
359.41			0.68			2136.57	
**93.52**	**21.52**	**336.37**	**2.31**	**8.64**	**2.94**	**1.97**	**1.11**
**2240.30**	**1439.19**	**7310.95**	**941.67**	**971.61**	**321885.43**	**41934.49**	**556.89**

?1994-2018 China Academic Journal Electronic Publishing House. All rights reserved. http://www.cnki.net

5-2 中国能源平衡表(标准量) -2010

单位：万吨标准煤

项 目	Item	能源合计 Energy Total	
		(发电煤耗计算法) (coal equivalent calculation)	(电热当量计算法) (calorific value calculation)
**一.可供本地区消费的能源量**	**Total Primary Energy Supply**	**332703.37**	**315747.30**
1.一次能源生产量	Indigenous Production	296915.72	279693.73
水电	Hydro Power	23085.71	8875.49
核电	Nuclear Power	2361.73	907.99
风电	Wind Power	1426.43	548.40
2.回收能	Recovery of Energy	5143.08	5143.08
3.进口量	Import	54863.59	54754.48
4.境内轮船和飞机在境外的加油量	Domestic Airplanes&Ships Refueling in Abroad	932.61	932.61
5.出口量(-)	Export (-)	-7802.37	-7427.34
6.境外轮船和飞机在境内的加油量(-)	Oversea Airplanes&Ships Refueling in China	-1042.79	-1042.79
7.库存增(-)、减(+)量	Stock Change	-16306.47	-16306.47
**二.加工转换投入(-)产出(+)量**	**Input(-) & Output(+) of Transformation**	**-11072.83**	**-76635.35**
1.火力发电	Thermal Power	0.00	-65562.52
2.供 热	Heating Supply	-3580.18	-3580.18
3.洗 选 煤	Coal Washing	-3530.81	-3530.81
4.炼 焦	Coking	-1479.64	-1479.64
5.炼油及煤制油	Petroleum Refineries	3149.76	3149.76
#油品再投入量(-)	Petroleum Products Input (-)	-5291.78	-5291.78
6.制 气	Gas Works	-135.77	-135.77
#焦炭再投入量(-)	Coke Input (-)	-137.53	-137.53
7.天然气液化	Natural Gas Liquefaction	-16.75	-16.75
8.煤制品加工	Briquettes	-50.13	-50.13
**三.损 失 量**	**Loss**	**8856.66**	**3803.12**
**四.终端消费量**	**Total Final Consumption**	**305009.67**	**227548.49**
1.农、林、牧、渔业	Farming, Forestry, Animal Husbandry, Fishery Conservancy	6477.30	4555.85
2.工业	Industry	211626.09	155933.09
#用作原料、材料	Non-Energy Use	9811.58	9811.58
3.建筑业	Construction	6226.30	5275.42
4.交通运输、仓储和邮政业	Transport, Storage and Post	25614.71	24169.37
5.批发、零售业和住宿、餐饮业	Wholesale, Retail Trade and Hotel, Restaurants	6826.82	4284.55
6.其他	Others	13680.50	8856.03
7.生活消费	Residential Consumption	34557.94	24474.18
城 镇	Urban	20706.84	14827.22
乡 村	Rural	13851.10	9646.96
**五.平衡差额**	**Statistical Difference**	**7764.22**	**7760.34**
**六.消费量合计**	**Total Final Consumption**	**324939.15**	**307986.96**

?1994-2018 China Academic Journal Electronic Publishing House. All rights reserved. http://www.cnki.net

ENERGY BALANCE OF CHINA -2010 (STANDARD QUANTITY)

(10 000 tce)

煤合计<br>Coal Total	原煤<br>Raw Coal	洗精煤<br>Cleaned Coal	其他洗煤<br>Other Washed Coal	型煤<br>Briquettes	煤矸石<br>Gangue	焦炭<br>Coke	焦炉煤气<br>Coke Oven Gas	高炉煤气<br>Blast Furnace Gas
**224756.84**	**226554.94**	**-844.70**	**-948.02**	**-5.39**		**-1894.95**		**2920.00**
227319.77	227319.77							
								2920.00
11649.15	11647.32			1.83		10.64		
-1526.85	-1522.38			-4.48		-325.42		
-12685.23	-10889.77	-844.70	-948.02	-2.75		-1580.17		
**-157863.96**	**-167129.16**	**3737.08**	**4867.80**	**660.33**	**-0.02**	**35290.56**	**3221.78**	**-1481.90**
-100049.33	-98348.34	-4.22	-1696.76		-445.31		-602.71	-1018.51
-11143.02	-10777.94	-9.48	-355.60		-94.19		-416.15	-463.38
-4070.29	-52947.87	41354.29	7523.29		539.48			
-41558.55	-4402.28	-37126.60	-29.67			35174.01	4104.82	
-168.64	-81.64	-83.91	-3.09					
-823.993238	-430.98	-393.012				241.1268903	135.82178	
						-124.57		
-50.13	-140.10		-570.37	660.33				
**61385.13**	**54840.04**	**2287.60**	**3626.74**	**630.75**		**32599.76**	**3150.07**	**1427.46**
1282.47	1261.60		20.88			45.48		
49318.47	43816.36	2276.43	2976.68	249.01		32498.63	2825.86	1427.46
3161.78	2916.77	117.01	128.00			1300.17	28.26	
523.15	516.45	1.22	5.48			5.64		
449.45	431.53	9.95	7.98			0.12		
1469.58	1429.08		20.55	19.95		4.95	4.57	
1467.00	1367.02		93.02	6.96		2.69	15.31	
6875.02	6018.01		502.17	354.84		42.24	304.33	
1355.50	1078.67		169.56	107.26		23.04	304.33	
5519.52	4939.34		332.61	247.57		19.21		
**5507.75**	**4585.74**	**604.78**	**293.04**	**24.19**	**-0.02**	**795.86**	**71.71**	**10.64**

?1994-2018 China Academic Journal Electronic Publishing House. All rights reserved. http://www.cnki.net

续表 1

单位: 万吨标准煤

项　目	Item	转炉煤气 Converter Gas	其他煤气 Gasoline
**一.可供本地区消费的能源量**	**Total Primary Energy Supply**	**694.40**	
1.一次能源生产量	Indigenous Production		
水电	Hydro Power		
核电	Nuclear Power		
风电	Wind Power		
2.回收能	Recovery of Energy	694.40	
3.进口量	Import		
4.境内轮船和飞机在境外的加油量	Domestic Airplanes&Ships Refueling in Abroad		
5.出口量(-)	Export (-)		
6.境外轮船和飞机在境内的加油量(-)	Oversea Airplanes&Ships Refueling in China		
7.库存增(-)、减(+)量	Stock Change		
**二.加工转换投入(-)产出(+)量**	**Input(-) & Output(+) of Transformation**	**-212.91**	**311.66**
1.火力发电	Thermal Power	-125.25	
2.供　热	Heating Supply	-87.66	
3.洗 选 煤	Coal Washing		
4.炼　焦	Coking		
5.炼油及煤制油	Petroleum Refineries		
#油品再投入量(-)	Petroleum Products Input (-)		
6.制　气	Gas Works		311.66
#焦炭再投入量(-)	Coke Input (-)		
7.天然气液化	Natural Gas Liquefaction		
8.煤制品加工	Briquettes		
**三.损 失 量**	**Loss**		
**四.终端消费量**	**Total Final Consumption**	**485.86**	**301.14**
1.农、林、牧、渔业	Farming, Forestry, Animal Husbandry, Fishery Conservancy		
2.工业	Industry	485.86	98.23
#用作原料、材料	Non-Energy Use		
3.建筑业	Construction		
4.交通运输、仓储和邮政业	Transport, Storage and Post		
5.批发、零售业和住宿、餐饮业	Wholesale, Retail Trade and Hotel, Restaurants		
6.其他	Others		
7.生活消费	Residential Consumption		202.91
城 镇	Urban		202.13
乡 村	Rural		0.79
**五.平衡差额**	**Statistical Difference**	**-4.37**	**10.51**
**六.消费量合计**	**Total Final Consumption**		

?1994-2018 China Academic Journal Electronic Publishing House. All rights reserved. http://www.cnki.net

Continued 1

(10 000 tce)

其他焦化产品 Other Coking Products	油品合计 Petroleum Products Total	原油 Crude Oil	汽油 Gasoline	煤油 Kerosene	柴油 Diesel Oil	燃料油 Fuel Oil	石脑油 Naphtha	润滑油 Lubricants	石蜡 Petroleum Waxes
	**63233.54**	**61527.84**	**-656.49**	**-230.67**	**-324.23**	**1826.42**	**5.55**	**32.46**	**-69.74**
	29002.58	29002.58							
	40908.64	33955.22	0.01	716.57	262.28	3284.35	436.05	48.37	1.23
	932.61			351.75	14.85	566.01			
	-4804.85	-432.87	-760.71	-890.20	-676.09	-1414.31	-130.50	-15.91	-70.97
	-1042.79			-390.66	-38.23	-613.90			
	-2041.07	-1271.38	104.20	-18.14	112.97	4.27	-300.00		
**787.81**	**-3363.39**	**-59824.67**	**10830.06**	**2827.63**	**21574.91**	**1688.79**	**2897.36**	**309.65**	**218.52**
	-624.93	-5.30	-0.13		-165.90	-176.99			
	-819.91	-4.69			-5.51	-287.63			
800.08									
-51.06	3373.23	-59814.68	10830.20	2831.55	21746.31	3624.32	2910.98	309.65	218.52
	-5291.78			-3.91		-1470.90	-13.62		
51.76									
-12.96									
	**278.41**	**274.29**							
**776.64**	**58005.74**	**1151.62**	**10132.23**	**2562.31**	**21151.51**	**3433.18**	**2761.53**	**318.22**	**140.57**
	2018.04		248.77	1.32	1758.33	1.63			
776.64	20480.66	1151.62	1014.34	55.24	2981.46	1460.71	2761.53	318.22	140.57
	5321.37	153.17	32.56				435.00	309.25	137.95
	4114.71		404.19	12.90	714.27	43.94			
	21479.73		4715.73	2355.83	12412.39	1895.26			
	722.22		247.45	51.47	286.47	12.31			
	3755.28		1715.98	56.98	1875.56	19.33			
	5435.10		1785.77	28.56	1123.02				
	3962.49		1242.83	2.49	835.55				
	1472.62		542.94	26.07	287.47				
**11.17**	**1307.60**	**2.97**	**41.34**	**34.66**	**99.17**	**82.04**	**141.38**	**23.89**	**8.20**

?1994-2018 China Academic Journal Electronic Publishing House. All rights reserved. http://www.cnki.net

续表 2

单位: 万吨标准煤

项目	Item	溶剂油<br>White spirit	石油沥青<br>Bitumen Asphalt
**一.可供本地区消费的能源量**	**Total Primary Energy Supply**	**-0.25**	**514.44**
1.一次能源生产量	Indigenous Production		
水电	Hydro Power		
核电	Nuclear Power		
风电	Wind Power		
2.回收能	Recovery of Energy		
3.进口量	Import	4.93	534.09
4.境内轮船和飞机在境外的加油量	Domestic Airplanes&Ships Refueling in Abroad		
5.出口量(-)	Export (-)	-0.78	-19.65
6.境外轮船和飞机在境内的加油量(-)	Oversea Airplanes&Ships Refueling in China		
7.库存增(-)、减(+)量	Stock Change	-4.40	
**二.加工转换投入(-)产出(+)量**	**Input(-) & Output(+) of Transformation**	**109.06**	**2572.71**
1.火力发电	Thermal Power		
2.供　热	Heating Supply		
3.洗 选 煤	Coal Washing		
4.炼　焦	Coking		
5.炼油及煤制油	Petroleum Refineries	109.06	2572.71
#油品再投入量(-)	Petroleum Products Input (-)		
6.制　气	Gas Works		
#焦炭再投入量(-)	Coke Input (-)		
7.天然气液化	Natural Gas Liquefaction		
8.煤制品加工	Briquettes		
**三.损 失 量**	**Loss**		
**四.终端消费量**	**Total Final Consumption**	**102.70**	**2906.06**
1.农、林、牧、渔业	Farming, Forestry, Animal Husbandry, Fishery Conservancy		
2.工业	Industry	102.70	58.78
#用作原料、材料	Non-Energy Use	98.64	
3.建筑业	Construction		2847.29
4.交通运输、仓储和邮政业	Transport, Storage and Post		
5.批发、零售业和住宿、餐饮业	Wholesale, Retail Trade and Hotel, Restaurants		
6.其他	Others		
7.生活消费	Residential Consumption		
城 镇	Urban		
乡 村	Rural		
**五.平衡差额**	**Statistical Difference**	**6.10**	**181.08**
**六.消费量合计**	**Total Final Consumption**		

?1994-2018 China Academic Journal Electronic Publishing House. All rights reserved.   http://www.cnki.net

Continued 2

(10 000 tce)

石油焦 Petroleum Coke	液化石油气 LPG	炼厂干气 Refinery Gas	其他石油制品 Other Petroleum Products	天然气 Natural Gas	液化天然气 LNG	热力 Heat	电力 Electricity	其他能源 Other Energy
**465.84**	**401.06**		**-258.69**	**12554.93**	**1641.75**	**970.68**	**10590.51**	**558.00**
				12614.78			10756.60	
							8875.49	
							907.99	
							548.40	
						970.68		558.00
812.55	560.58		292.41	476.14	1641.75		68.15	
-220.71	-159.43		-12.71	-535.99			-234.24	
-126.00	-4.20		-538.38					
**1469.43**	**3505.01**	**1766.19**	**6691.96**	**-2673.17**	**-229.35**	**9185.03**	**40949.40**	**-556.89**
-114.94		-116.30	-45.37	-2151.67	-294.66	-820.69	40949.40	-378.85
-121.36	-1.51	-276.22	-123.00	-371.87	-15.43	10005.72		-174.28
1705.74	3603.92	2295.36	10429.62					-3.76
	-97.41	-136.65	-3569.29					
				-52.14				
				-97.49	80.74			
	**4.11**			**227.56**	**24.85**	**115.94**	**3156.37**	
**1890.00**	**3737.52**	**1732.37**	**5985.90**	**9623.53**	**1372.37**	**10039.66**	**48381.12**	
	7.99			6.65		3.10	1200.11	
1890.00	907.00	1732.37	5906.10	4815.53	1150.97	7269.74	34785.04	
1661.85	98.95		2394.00	1203.88	287.74			
	12.31		79.80	15.46		22.57	593.90	
	100.53			1060.08	221.41	55.85	902.74	
	124.52			362.30		133.06	1587.87	
	87.43			345.80		256.64	3013.30	
	2497.74			3017.71		2298.69	6298.17	
	1881.61			3008.72		2298.69	3672.33	
	616.13			8.99			2625.84	
**45.28**	**160.32**	**33.82**	**447.37**	**30.67**	**15.18**	**0.10**	**2.42**	**1.11**

?1994-2018 China Academic Journal Electronic Publishing House. All rights reserved. http://www.cnki.net

5-3 综合能源平衡表

单位：万吨标准煤

项目	Item	1980	1985
**可供消费的能源总量**	**Total Energy Available for Consumption**	**61557**	**77603**
一次能源生产量	Primary Energy Output	63735	85546
回收能	Recovery of Energy		
进口量	Imports	261	340
出口量(−)	Exports (-)	3058	5774
年初年末库存差额	Stock Changes in the Year	619	-2509
**能源消费总量**	**Total Energy Consumption**	**60275**	**76682**
在总量中：	Consumption by Sector		
1.农、林、牧、渔业	Farming, Forestry, Animal Husbandry, Fishery Conservancy	4692	4045
2.工业	Industry	38986	51068
3.建筑业	Construction	957	1302
4.交通运输、仓储和邮政业	Transport, Storage and Post	2902	3713
5.批发、零售业和住宿、餐饮业	Wholesale, Retail Trade and Hotel, Restaurants	518	766
6.其他	Others	1205	2470
7.生活消费	Residential Consumption	11015	13318
在总量中：	Consumption by Usage		
(一) 终端消费	(I)Final Consumption	57508	73586
#工业	Industry	38293	48021
(二) 加工转换损失量	(II)Losses in Processing and	1358	1491
#炼焦	Coking	644	572
炼油	Petroleum Refining	113	110
(三) 损失量	(III)Other Losses	1409	1605
**平衡差额**	**Balance**	**1282**	**921**

注：1.村办工业包括在工业中(下同)。
2.电力、热力按等价热值折算，因此加工转换损失量中不包括发电、供热损失量。
3.进口量包括境内轮船和飞机在境外的加油量；出口量包括境外轮船和飞机在境内的加油量。

?1994-2018 China Academic Journal Electronic Publishing House. All rights reserved. http://www.cnki.net

OVERALL ENERGY BALANCE SHEET

(10 000 tce)

1990	1995	2000	2005	2006	2007	2008	2009	2010
**96138**	**129535**	**142605**	**232225**	**256034**	**274800**	**287011**	**311277**	**332703**
103922	129034	135048	216219	232167	247279	260552	274619	296916
	2312	1760	2939	3725	6166	6511	7627	5143
1310	5456	14334	26952	31171	35062	36764	47313	55796
5875	6776	9633	11448	10925	9995	9955	8440	-8845
-3219	-491	1097	-2436	-104	-3711	-6860	-9841	-16306
**98703**	**131176**	**145531**	**235997**	**258676**	**280508**	**291448**	**306647**	**324939**
4852	5505	3914	6071	6331	6228	6013	6251	6477
67578	96191	103774	168724	184945	200531	209302	219197	231102
1213	1335	2179	3403	3761	4128	3813	4562	6226
4541	5863	11242	18391	20284	21959	22917	23692	26068
1247	2018	3048	4848	5314	5689	5734	6412	6827
3473	4519	5762	9255	10276	11158	11771	12690	13681
15799	15745	15614	25305	27765	30814	31898	33843	34558
94289	124252	139008	225690	247520	268610	278546	292299	305010
63239	89473	97597	158767	174225	189032	196832	205322	211626
2264	3634	2461	3823	4056	4241	5166	6283	11073
905		525	702	734	854	819	1010	1480
326		781	1305	1391	1325	1380	1784	2142
2150	3289	4062	6483	7100	7657	7736	8065	8857
**-2565**	**-1641**	**-2926**	**-3772**	**-2642**	**-5708**	**-4437**	**4630**	**7764**

a) Data on industry include the data of village-run industry.(The sane as in the following tables).

b) Electric power and heat are converted on the basic of equal caloric value. Therefore, losses in processing and transformation exclude losses in powei generation and heating.

c) Data on imports include the petroleum consumed by the domestic airplanes and ships in refueling abroad. Data on exports include the petroleum consumed by the oversea airplanes and ships in refueling in China.

?1994-2018 China Academic Journal Electronic Publishing House. All rights reserved. http://www.cnki.net

5-4 煤炭平衡表

单位: 万吨

项目	Item	1980	1985
**可供量**	**Total Energy Available for Consumption**	**62601.0**	**82776.6**
生产量	Output	62015.0	87228.4
进口量	Imports	199.0	230.7
出口量(-)	Exports (-)	632.0	777.0
年初年末库存差额	Stock Changes in the Year	1019.0	-3905.5
**消费量**	**Total Energy Consumption**	**61009.5**	**81603.0**
在消费量中:	Consumption by Sector		
1.农、林、牧、渔业	Farming, Forestry, Animal Husbandry, Fishery Conservancy	1550.3	2208.6
2.工业	Industry	43848.4	58613.3
3.建筑业	Construction	556.0	531.9
4.交通运输、仓储和邮政业	Transport, Storage and Post	1934.4	2307.1
5.批发、零售业和住宿、餐饮业	Wholesale, Retail Trade and Hotel, Restaurants	455.2	738.2
6.其他	Others	1091.2	1579.5
7.生活消费	Residential Consumption	11574.0	15624.4
在消费量中:	Consumption by Usage		
(一) 终端消费	(I)Final Consumption	38804.2	52704.4
#工 业	Industry	21643.1	29715.0
(二) 中间消费	(2)Intermediate Consumption		
(用于加工转换)	(Consumed in Transformation)	19461.6	25397.4
发 电	Power Generation	12648.4	16440.7
供 热	Heating		1462.3
炼 焦	Coking	6682.2	7303.8
炼油及煤制油			
制 气	Gas Production	131.0	190.6
(三) 洗选损耗	(3) Losses in Coal Washing and Dressing	2743.7	3501.2
**平衡差额**	**Balance**	**1591.5**	**1173.6**

注: 生产量为原煤产量。

?1994-2018 China Academic Journal Electronic Publishing House. All rights reserved. http://www.cnki.net

COAL BALANCE SHEET

(10 000 ton)

1990	1995	2000	2005	2006	2007	2008	2009	2010
**102221.0**	**133461.7**	**136794.5**	**226941.0**	**251336.3**	**265543.5**	**275061.1**	**301283.8**	**319772.0**
107988.3	136073.1	138418.5	234951.8	252855.1	269164.3	280200.0	297300.0	323500.0
200.3	163.5	217.9	2617.1	3810.5	5101.6	4034.1	12584.0	16309.5
1729.0	2861.7	5506.5	7172.4	6327.3	5318.7	4543.4	2239.6	1910.4
-4238.5	86.8	3664.7	-3455.4	997.9	-3403.6	-4629.6	-6360.6	-18127.2
**105523.0**	**137676.5**	**141091.7**	**231851.1**	**255065.5**	**272745.9**	**281095.9**	**295833.1**	**312236.5**
2095.2	1856.7	933.4	1513.8	1502.6	1519.6	1522.6	1582.1	1711.1
81090.9	117570.7	127806.7	215493.3	238510.2	256202.8	265574.2	279888.5	296031.6
437.6	439.8	536.8	603.6	652.0	615.3	603.2	635.6	718.9
2160.9	1315.1	882.2	811.2	769.9	735.9	665.4	640.9	639.2
1058.3	977.4	1314.6	1674.4	1791.5	1868.3	1791.4	1977.9	1969.9
1980.4	1986.7	1161.0	1715.9	1802.9	2043.4	1791.6	1986.1	2006.6
16699.7	13530.1	8457.0	10039.0	10036.3	9760.6	9147.6	9121.9	9159.2
60205.9	66156.1	55913.1	75382.7	77532.6	79676.6	81089.2	83700.5	84350.9
35773.8	46050.3	42628.0	59024.9	60977.4	63133.5	65567.5	67755.9	68146.1
41257.8	69487.6	85178.6	156468.4	177532.8	193069.3	200006.7	212132.6	227885.6
27204.3	44440.2	55811.2	103263.5	118763.9	130548.8	135351.7	143967.3	154542.5
2995.5	5887.3	8794.1	13542.0	14561.4	15394.2	15029.2	15359.7	15253.1
10697.6	18396.4	16496.4	33167.1	37450.1	39659.0	41461.7	43691.7	47150.4
								213.4
360.4	763.7	960.0	1277.0	1257.1	1491.8	1227.2	1150.7	1040.1
4059.3	2032.8	3191.2	4982.1	5279.3	5754.6	6757.8	7765.5	9484.6
**-3302.0**	**-4214.8**	**-4297.2**	**-4910.0**	**-3729.2**	**-7202.4**	**-6034.9**	**5450.7**	**7535.5**

a) Data on output refer to the output of raw coal.

?1994-2018 China Academic Journal Electronic Publishing House. All rights reserved. http://www.cnki.net

5-5 焦炭平衡表

单位: 万吨

项目	Item	1980	1985
**可供量**	**Total Energy Available for Consumption**	**4315.3**	**4689.7**
生产量	Output	4343.0	4802.1
进口量	Imports		2.1
出口量(-)	Exports (-)	27.1	36.9
年初年末库存差额	Stock Changes in the Year	-0.6	-77.6
**消费量**	**Total Energy Consumption**	**4303.0**	**4689.7**
在消费量中:	Consumption by Sector		
1.农、林、牧、渔业	Farming, Forestry, Animal Husbandry, Fishery Conservancy	10.6	20.8
2.工业	Industry	4266.7	4627.7
3.建筑业	Construction	11.9	7.8
4.交通运输、仓储和邮政业	Transport, Storage and Post	8.2	5.7
5.批发、零售业和住宿、餐饮业	Wholesale, Retail Trade and Hotel, Restaurants	0.9	2.7
6.其他	Others	4.7	2.0
7.生活消费	Residential Consumption		23.0
在消费量中:	Consumption by Usage		
(一) 终端消费	(1)Final Consumption	4294.7	4677.9
#工　业	Industry	4258.4	4615.9
(二) 中间消费	(2)Intermediate Consumption		
(用于加工转换)	(Consumed in Transformation)	8.3	11.8
制　气	Gas Production	8.3	11.8
(三) 损失量	(3) Losses in Coal Washing and Dressing		
**平衡差额**	**Balance**	**12.3**	

?1994-2018 China Academic Journal Electronic Publishing House. All rights reserved. http://www.cnki.net

COKE BALANCE SHEET

(10 000 ton)

1990	1995	2000	2005	2006	2007	2008	2009	2010
**7085.8**	**12207.1**	**10892.3**	**25184.4**	**27990.5**	**29090.1**	**29994.0**	**31961.3**	**34507.1**
7328.3	13424.5	12184.0	26611.7	29768.3	31305.3	32313.9	34244.1	36457.8
	0.1		0.5				15.9	11.0
129.0	886.1	1519.7	1276.4	1446.8	1529.9	1221.3	54.5	335.0
-113.5	-331.4	228.0	-151.4	-331.1	-685.3	-1098.6	-2244.1	-1626.7
**6914.7**	**10725.3**	**10840.8**	**25105.8**	**27892.8**	**29168.1**	**29900.2**	**31850.0**	**33687.8**
60.1	128.6	70.9	63.5	55.7	57.2	53.1	44.6	46.8
6808.8	10412.0	10554.6	24860.9	27653.6	28932.2	29756.7	31743.3	33583.7
5.2	10.8	19.0	18.4	18.5	17.5	10.7	5.7	5.8
4.1	10.1	11.2	1.1	0.9	0.6	0.3	0.1	0.1
7.7	25.7	35.7	64.1	65.4	71.0	7.5	3.9	5.1
1.9	6.4	12.2	7.6	8.3	8.2	6.9	3.5	2.8
26.9	131.6	137.2	90.3	90.4	81.4	64.9	48.8	43.5
6846.3	10648.0	10697.9	24877.9	27664.9	28900.1	29712.6	31689.7	33559.6
6740.4	10334.7	10411.7	24633.0	27425.6	28664.2	29569.1	31583.0	33455.5
68.4	77.3	142.9	227.9	227.9	268.0	187.6	160.3	128.2
68.4	77.3	142.9	227.9	227.9	268.0	187.6	160.3	128.2
**171.1**	**1481.8**	**51.6**	**78.6**	**97.7**	**-78.1**	**93.8**	**111.4**	**819.3**

?1994-2018 China Academic Journal Electronic Publishing House. All rights reserved. http://www.cnki.net

5-6 石油平衡表

单位: 万吨

项　　目	Item	1980	1985
**可供量**	**Total Energy Available for Consumption**	**8794.5**	**9193.7**
生产量	Output	10594.6	12489.5
进口量	Imports	82.7	90.0
出口量(-)	Exports (-)	1806.2	3630.4
年初年末库存差额	Stock Changes in the Year	-76.6	244.6
**消费量**	**Total Energy Consumption**	**8757.4**	**9168.8**
在消费量中:	Consumption by Sector		
1.农、林、牧、渔业	Farming, Forestry, Animal Husbandry, Fishery Conservancy	814..9	758.7
2.工业	Industry	6203.2	6171.4
3.建筑业	Construction	175.2	292.2
4.交通运输、仓储和邮政业	Transport, Storage and Post	911.5	1176.4
5.批发、零售业和住宿、餐饮业	Wholesale, Retail Trade and Hotel, Restaurants	29.0	38.1
6.其他	Others	481.7	506.1
7.生活消费	Residential Consumption	141.9	225.9
在消费量中:	Consumption by Usage	8838.9	
(一) 终端消费	(I)Final Consumption	6311.0	7063.3
#工　业	Industry	3780.3	4462.0
(二) 中间消费	(II)Intermediate Consumption		
(用于加工转换)	(Consumed in Transformation)	2183.6	1858.5
发　电	Power Generation	2065.4	1425.5
供　热	Heating		285.6
制　气	Gas Production	36.7	34.5
炼油损失量	Losses in Petroleum Refining	81.5	112.9
(三)损失量	(III) Other Losses	262.8	247.0
**平衡差额**	**Balance**	**37.1**	**24.9**

注: 1.生产量为原油产量。
2.进口量包括境内轮船和飞机在境外的加油量; 出口量包括境外轮船和飞机在境内的加油量。

?1994-2018 China Academic Journal Electronic Publishing House. All rights reserved.　http://www.cnki.net

PETROLEUM BALANCE

(10 000 ton)

1990	1995	2000	2005	2006	2007	2008	2009	2010
**11435.0**	**16072.7**	**22631.8**	**32539.1**	**34930.0**	**36648.9**	**37318.8**	**38462.8**	**44178.4**
13830.6	15005.0	16300.0	18135.3	18476.6	18631.8	19044.0	18949.0	20301.4
755.6	3673.2	9748.5	17163.2	19453.0	21139.4	23015.5	25642.4	29437.2
3110.4	2454.5	2172.1	2888.1	2626.2	2664.3	2945.7	3916.6	4079.0
-40.8	-151.0	-1244.6	128.8	-373.3	-458.0	-1795.0	-2211.9	-1481.2
**11485.6**	**16064.9**	**22495.9**	**32537.7**	**34876.2**	**36658.7**	**37302.9**	**38384.5**	**43245.2**
1033.6	1203.2	788.5	1451.7	1540.2	1399.9	1265.8	1308.1	1382.5
7321.6	9349.3	11248.5	14245.1	14804.3	14905.1	15603.1	15692.9	17448.8
327.3	242.8	840.6	1502.2	1648.5	1823.1	1517.5	1942.3	3045.1
1683.2	2863.6	6399.0	10709.5	11849.2	12906.7	13279.4	13548.5	14870.3
77.6	333.9	247.0	375.6	392.2	426.9	366.4	429.7	481.0
757.8	1390.3	1635.9	1969.2	2077.6	2215.9	2353.8	2296.3	2556.7
284.5	682.0	1336.5	2284.4	2564.2	2981.1	2916.9	3166.8	3460.8
9304.7	13676.3	19950.1	29191.6	31614.1	33857.6	34702.9	35689.9	40393.7
5180.4	7095.5	8860.0	11027.5	11707.5	12269.2	13170.8	13153.7	14757.8
1630.4	2230.0	2352.9	3190.7	3062.1	2601.2	2397.6	2505.9	2657.1
1234.4	1358.5	1178.2	1602.0	1343.7	884.0	618.1	501.2	459.1
356.3	399.9	427.0	407.6	427.8	430.1	413.8	383.3	593.1
39.7	51.6	25.9	14.4	13.4	5.0	2.0	0.3	
295.8	420.1	721.9	1166.7	1277.3	1282.1	1363.7	1621.1	1604.8
254.7	158.6	192.9	155.4	200.0	200.0	202.4	188.7	194.4
**-50.6**	**7.8**	**135.8**	**1.4**	**53.8**	**-9.9**	**15.9**	**78.3**	**933.3**

a) Data on output refer to the output of crude oil.

b) Data on imports include the petroleum consumed by the domestic airplanes and ships in refueling abroad.
Data on exports include the petroleum consumed by the oversea airplanes and ships in refueling in China.

?1994-2018 China Academic Journal Electronic Publishing House. All rights reserved. http://www.cnki.net

5-7 原油平衡表

单位: 万吨

项 目	Item	1980	1985
**可供量**	**Total Energy Available for Consumption**	**9222.9**	**9516.5**
生产量	Output	10594.6	12489.5
进口量	Imports	36.6	
出口量(-)	Exports (-)	1330.9	3003.0
年初年末库存差额	Stock Changes in the Year	-77.4	30.0
**消费量**	**Total Energy Consumption**	**9205.0**	**9509.5**
在消费量中:	Consumption by Sector		
1.农、林、牧、渔业	Farming, Forestry, Animal Husbandry, Fishery Conservancy	8.0	0.8
2.工业	Industry	9112.0	9389.9
3.建筑业	Construction	28.8	74.0
4.交通运输、仓储和邮政业	Transport, Storage and Post	50.1	44.3
5.批发、零售业和住宿、餐饮业	Wholesale, Retail Trade and Hotel, Restaurants		0.1
6.其他	Others	6.1	0.4
7.生活消费	Residential Consumption		
在消费量中:	Consumption by Usage		
(一) 终端消费	(I)Final Consumption	499.6	350.4
#工 业	Industry	429.7	254.9
(二) 中间消费	(II)Intermediate Consumption		
(用于加工转换)	(Consumed in Transformation)	8443.0	8929.7
发 电	Power Generation	574.0	279.5
供 热	Heating		61.3
炼 油	Petroleum Refineries	7869.0	8588.9
(三) 油田原油损失量	(III)Losses in Oil Field for Crude Oil	262.4	229.4
**平衡差额**	**Balance**	**17.9**	**7.0**

?1994-2018 China Academic Journal Electronic Publishing House. All rights reserved. http://www.cnki.net

CRUDE OIL BALANC SHEET

(10 000 ton)

1990	1995	2000	2005	2006	2007	2008	2009	2010
**11770.6**	**14794.9**	**21383.0**	**30089.2**	**32249.2**	**34035.1**	**35498.6**	**38130.5**	**42876.6**
13830.6	15004.4	16300.0	18135.3	18476.6	18631.8	19044.0	18949.0	20301.4
292.3	1709.0	7026.5	12681.7	14517.5	16316.0	17888.5	20365.3	23768.2
2399.0	1822.7	1030.6	806.7	633.7	388.4	423.8	507.3	303.0
46.7	-95.8	-912.9	78.8	-111.1	-524.4	-1010.1	-676.5	-890.0
**11762.2**	**14886.4**	**21232.0**	**30086.2**	**32245.2**	**34031.6**	**35498.2**	**38128.6**	**42874.6**
0.2	10.1							
11653.8	14716.3	21052.1	29959.4	32081.5	33867.9	35332.6	37975.2	42716.6
55.2	2.7	3.3						
52.1	156.8	175.1	126.9	163.7	163.7	165.7	153.4	158.0
0.3	0.5	0.2						
0.6	1390.3	1.4						
402.1	309.9	636.8	870.4	977.1	984.1	1192.8	826.2	806.1
333.4	274.7	612.3	870.4	977.1	984.1	1192.8	826.2	806.1
11106.9	14419.4	20404.3	29062.1	31069.8	32849.1	34105.1	37115.8	41876.4
124.6	61.6	85.0	21.3	22.9	15.9	8.9	4.4	3.7
21.1	4.4	14.0	0.3	0.8	0.6	1.0	2.0	3.3
10961.2	14353.4	20305.3	29040.5	31046.0	32832.6	34095.3	37109.4	41869.4
253.2	157.1	190.9	153.8	198.4	198.4	200.4	186.7	192.0
**8.4**	**-91.5**	**151.0**	**2.9**	**4.0**	**3.5**	**0.4**	**1.9**	**2.1**

?1994-2018 China Academic Journal Electronic Publishing House. All rights reserved. http://www.cnki.net

5-8 燃料油平衡表

单位：万吨

项目	Item	1980	1985
**可供量**	**Total Energy Available for Consumption**	**3096.1**	**2848.0**
生产量	Output	3142.0	2835.8
进口量	Imports	39.0	70.0
出口量(-)	Exports (-)	45.4	64.9
年初年末库存差额	Stock Changes in the Year	-39.5	7.1
**消费量**	**Total Energy Consumption**	**3073.7**	**2837.4**
在消费量中:	Consumption by Sector		
1.农、林、牧、渔业	Farming, Forestry, Animal Husbandry, Fishery Conservancy	2.3	3.1
2.工业	Industry	2937.4	2662.2
3.建筑业	Construction	15.0	18.9
4.交通运输、仓储和邮政业	Transport, Storage and Post	109.0	144.1
5.批发、零售业和住宿、餐饮业	Wholesale, Retail Trade and Hotel, Restaurants	2.9	3.1
6.其他	Others	7.1	6.0
7.生活消费	Residential Consumption		
在消费量中:	Consumption by Usage		
(一) 终端消费	(I)Final Consumption	1617.9	1538.8
#工　业	Industry	1481.6	1363.5
(二) 中间消费	(II)Intermediate Consumption		
(用于加工转换)	(Consumed in Transformation)	1455.8	1296.1
发　电	Power Generation	1419.1	1042.3
供　热	Heating		219.3
炼油再投入量	Petroleum Production		
制　气	Gas Production	36.7	34.5
(三)损失量	(III)Other Losses		2.5
**平衡差额**	**Balance**	**22.4**	**10.6**

?1994-2018 China Academic Journal Electronic Publishing House. All rights reserved.   http://www.cnki.net

FUEL OIL BALANCE SHEET

(10 000 ton)

1990	1995	2000	2005	2006	2007	2008	2009	2010
**3320.7**	**3717.3**	**3836.7**	**4237.3**	**4371.8**	**4158.4**	**3242.4**	**2829.3**	**3815.4**
3267.9	2960.8	2053.7	1767.4	1784.7	1967.2	1737.4	1353.4	2537.0
167.3	859.1	1704.3	2883.9	3138.1	2807.6	2533.2	2723.9	2695.2
97.2	68.6	57.9	427.6	466.9	631.4	1036.4	1222.4	1419.7
-17.3	-34.0	136.6	13.5	-84.1	14.9	8.3	-25.5	3.0
**3367.8**	**3693.7**	**3872.8**	**4242.1**	**4368.3**	**4157.5**	**3237.1**	**2827.8**	**3758.0**
2.9	8.4	0.4	0.7	0.7	1.0	1.5	1.1	1.1
3091.7	3406.2	2975.1	2924.8	2836.1	2329.2	2039.5	1521.5	2377.3
47.3	14.2	16.7	14.2	16.3	30.7	37.7	34.2	30.8
208.2	227.5	850.0	1261.0	1480.6	1760.0	1142.8	1250.6	1326.7
1.6	6.6	11.6	27.5	21.4	24.8	6.3	8.1	8.6
16.1	30.8	19.0	13.9	13.1	11.8	9.5	12.3	13.5
2042.6	2262.8	2741.4	2949.9	3267.7	3455.5	2757.8	2533.7	2403.2
1766.5	1975.3	1843.7	1632.6	1735.5	1627.2	1560.1	1227.5	1022.5
1325.2	1430.9	1131.3	1292.2	1100.6	702.0	479.3	294.1	1354.8
977.3	1071.5	814.2	1106.1	928.0	565.6	347.5	193.0	123.9
308.3	307.8	291.2	171.7	159.2	131.5	129.8	100.7	201.3
								1029.6
39.6	51.6	25.9	14.4	13.4	5.0	2.0	0.3	
**-47.1**	**23.6**	**-36.1**	**-4.8**	**3.5**	**0.9**	**5.3**	**1.5**	**57.4**

?1994-2018 China Academic Journal Electronic Publishing House. All rights reserved. http://www.cnki.net

5-9 汽油平衡表

单位：万吨

项　目	Item	1980	1985
**可供量**	**Total Energy Available for Consumption**	**999.4**	**1399.6**
生产量	Output	1079.0	1471.9
进口量	Imports		0.3
出口量(-)	Exports (-)	117.8	129.9
年初年末库存差额	Stock Changes in the Year	38.2	57.3
**消费量**	**Total Energy Consumption**	**998.6**	**1396.3**
在消费量中:	Consumption by Sector		
1.农、林、牧、渔业	Farming, Forestry, Animal Husbandry, Fishery Conservancy	53.3	122.3
2.工业	Industry	273.2	451.3
3.建筑业	Construction	54.1	73.0
4.交通运输、仓储和邮政业	Transport, Storage and Post	404.9	477.4
5.批发、零售业和住宿、餐饮业	Wholesale, Retail Trade and Hotel, Restaurants	19.4	23.4
6.其他	Others	193.7	238.3
7.生活消费	Residential Consumption		10.6
**平衡差额**	**Balance**	**0.8**	**3.3**

5-10 煤油平衡表

单位：万吨

项　目	Item	1980	1985
**可供量**	**Total Energy Available for Consumption**	**359.0**	**383.2**
生产量	Output	398.5	405.3
进口量	Imports		15.2
出口量(-)	Exports (-)	46.8	46.0
年初年末库存差额	Stock Changes in the Year	2.3	8.7
**消费量**	**Total Energy Consumption**	**365.9**	**385.5**
在消费量中:	Consumption by Sector		
1.农、林、牧、渔业	Farming, Forestry, Animal Husbandry, Fishery Conservancy	2.3	3.3
2.工业	Industry	15.7	20.1
3.建筑业	Construction	0.8	1.3
4.交通运输、仓储和邮政业	Transport, Storage and Post	31.4	56.2
5.批发、零售业和住宿、餐饮业	Wholesale, Retail Trade and Hotel, Restaurants	0.2	0.1
6.其他	Others	216.7	182.9
7.生活消费	Residential Consumption	98.8	121.6
**平衡差额**	**Balance**	**-6.9**	**-2.3**

?1994-2018 China Academic Journal Electronic Publishing House. All rights reserved.   http://www.cnki.net

GASOLINE BALANCE SHEET

(10 000 ton)

1990	1995	2000	2005	2006	2007	2008	2009	2010
**1884.1**	**2902.0**	**3504.5**	**4854.7**	**5243.0**	**5519.3**	**6147.6**	**6181.8**	**6914.3**
2173.4	3051.6	4134.7	5433.0	5595.0	5917.9	6347.2	7320.7	7360.5
16.9	15.9			6.1	22.7	198.7	4.4	
233.8	193.1	467.7	559.7	350.5	464.3	203.4	491.9	517.0
-72.4	27.6	-162.5	-18.6	-7.5	42.9	-194.9	-651.4	70.8
**1899.5**	**2909.6**	**3504.6**	**4854.9**	**5242.5**	**5519.1**	**6145.5**	**6172.7**	**6886.2**
145.9	179.7	89.2	159.6	167.7	172.8	160.4	168.1	169.1
589.3	812.4	682.0	441.7	498.5	524.5	586.1	671.1	689.5
89.5	103.6	115.6	172.1	180.7	178.8	196.2	235.4	274.7
620.1	982.3	1527.8	2430.1	2592.4	2613.2	3090.4	2881.6	3204.9
46.0	197.2	69.8	129.4	123.3	131.7	135.3	147.5	168.2
390.7	570.7	792.7	998.2	1064.1	1119.7	1121.9	1069.9	1166.2
18.0	63.7	227.6	523.8	615.7	778.4	855.1	999.1	1213.7
**-15.4**	**-7.6**	**-0.1**	**-0.2**	**0.5**	**0.2**	**2.1**	**9.1**	**28.1**

KEROSENE BALANCE SHEET

(10 000 ton)

1990	1995	2000	2005	2006	2007	2008	2009	2010
**350.9**	**486.4**	**880.9**	**1070.0**	**1117.9**	**1239.2**	**1290.4**	**1448.2**	**1767.6**
392.5	445.8	872.3	1006.5	975.5	1153.3	1158.9	1480.3	1924.4
26.1	115.7	322.5	476.1	731.7	725.0	836.8	795.1	726.1
55.5	62.4	256.3	447.6	584.4	637.1	706.5	826.1	870.5
-12.2	-12.7	-57.6	35.0	-4.9	-2.0	1.3	-1.1	-12.3
**350.9**	**512.1**	**871.6**	**1076.8**	**1124.7**	**1243.7**	**1294.0**	**1439.4**	**1744.1**
3.1	3.6	1.5	1.6	1.5	0.9	1.3	0.8	0.9
20.6	44.9	84.0	57.5	48.2	45.2	49.1	32.0	40.2
1.3	3.5	4.0				9.7	10.4	8.8
93.4	250.0	535.9	952.4	1010.5	1130.0	1174.6	1314.3	1601.1
0.6	8.5	14.0	3.7	3.8	4.9	20.8	29.1	35.0
127.3	137.3	160.1	36.2	38.0	43.2	25.9	33.7	38.7
104.6	64.3	72.2	25.5	22.7	19.5	12.7	19.2	19.4
	**-25.7**	**9.3**	**-6.8**	**-6.9**	**-4.5**	**-3.6**	**8.8**	**23.6**

?1994-2018 China Academic Journal Electronic Publishing House. All rights reserved. http://www.cnki.net

5-11 柴油平衡表

单位：万吨

项目	Item	1980	1985
**可供量**	**Total Energy Available for Consumption**	**1663.2**	**1944.1**
生产量	Output	1827.8	2023.2
进口量	Imports	2.1	4.5
出口量(-)	Exports (-)	166.5	225.6
年初年末库存差额	Stock Changes in the Year	-0.2	142.0
**消费量**	**Total Energy Consumption**	**1663.2**	**1939.4**
在消费量中:	Consumption by Sector		
1.农、林、牧、渔业	Farming, Forestry, Animal Husbandry, Fishery Conservancy	749.0	629.2
2.工业	Industry	457.4	644.1
3.建筑业	Construction	76.5	125.0
4.交通运输、仓储和邮政业	Transport, Storage and Post	316.1	454.4
5.批发、零售业和住宿、餐饮业	Wholesale, Retail Trade and Hotel, Restaurants	6.5	10.9
6.其他	Others	57.7	74.0
7.生活消费	Residential Consumption		
在消费量中:	Consumption by Usage		
(一) 终端消费	(I)Final Consumption	1590.9	1827.4
#工 业	Industry	385.1	532.1
(二) 中间消费	(II)Intermediate Consumption		
(用于加工转换)	(Consumed in Transformation)	72.3	108.6
发 电	Power Generation	72.3	103.6
供 热	Heating		5.0
(三) 损失量	(III)Other Losses		3.4
**平衡差额**	**Balance**		**4.7**

5-12 液化石油气平衡表

单位：万吨

项目	Item	1980	1985
**可供量**	**Total Energy Available for Consumption**	**122.5**	**157.3**
生产量	Output	122.5	159.7
进口量	Imports		
出口量(-)	Exports (-)		1.9
年初年末库存差额	Stock Changes in the Year		-0.5
**消费量**	**Total Energy Consumption**	**119.6**	**155.7**
在消费量中:	Consumption by Sector		
1.农、林、牧、渔业	Farming, Forestry, Animal Husbandry, Fishery Conservancy		
2.工业	Industry	76.1	59.9
3.建筑业	Construction		
4.交通运输、仓储和邮政业	Transport, Storage and Post		
5.批发、零售业和住宿、餐饮业	Wholesale, Retail Trade and Hotel, Restaurants		0.5
6.其他	Others	0.4	4.5
7.生活消费	Residential Consumption	43.1	90.8
**平衡差额**	**Balance**	**2.9**	**1.6**

?1994-2018 China Academic Journal Electronic Publishing House. All rights reserved. http://www.cnki.net

DIESEL OIL BALANCE SHEET

(10 000 ton)

1990	1995	2000	2005	2006	2007	2008	2009	2010
**2689.4**	**4404.2**	**6806.5**	**10972.6**	**11836.1**	**12494.2**	**13543.1**	**13768.1**	**14701.9**
2609.0	3972.6	7079.6	11090.2	11762.4	12359.1	13409.2	14288.6	14924.4
233.8	645.3	51.9	61.0	80.7	173.7	633.1	192.5	190.2
169.8	169.5	77.5	170.9	102.6	93.3	89.1	478.7	490.2
16.4	-44.2	-247.6	-7.7	95.6	54.7	-410.0	-234.3	77.5
**2691.7**	**4321.4**	**6806.2**	**10972.2**	**11835.4**	**12496.7**	**13532.6**	**13756.6**	**14633.8**
881.5	1001.4	697.1	1286.3	1365.5	1219.0	1098.9	1134.1	1206.7
728.1	1189.9	1696.5	1991.3	1961.6	1972.8	2517.0	2348.8	2163.8
133.0	118.2	205.9	386.6	428.7	433.8	370.8	415.3	490.2
709.4	1246.6	3293.8	5890.4	6547.3	7184.4	7649.3	7892.0	8518.6
22.5	103.6	95.9	116.0	129.8	133.9	152.7	181.7	196.6
217.0	645.7	638.7	895.1	933.0	1007.5	1151.8	1131.8	1287.2
	16.1	178.4	406.4	469.6	545.3	592.1	652.9	770.7
2564.8	4070.0	6578.6	10605.4	11522.7	12265.3	13347.5	13604.8	14516.2
601.2	938.5	1468.8	1624.5	1648.9	1741.4	2331.9	2197.0	2046.2
126.9	251.4	227.7	366.7	312.7	231.4	185.1	151.8	117.6
124.5	204.9	227.7	366.7	312.7	231.4	185.1	151.8	113.9
2.4	46.6							3.8
**-2.3**	**82.7**	**0.3**	**0.4**	**0.7**	**-2.4**	**10.6**	**11.4**	**68.1**

LPG BALANCE SHEET

(10 000 ton)

1990	1995	2000	2005	2006	2007	2008	2009	2010
**258.5**	**774.3**	**1396.2**	**2052.2**	**2252.5**	**2320.2**	**2114.3**	**2157.7**	**2333.8**
261.6	540.8	916.6	1432.7	1745.3	1944.7	1914.8	1831.7	2102.3
	232.6	481.7	617.0	535.6	405.4	259.2	408.0	327.0
1.1	7.1	1.6	2.7	15.1	33.8	67.9	84.9	93.0
-2.0	8.0	-0.6	5.2	-13.3	3.8	8.1	2.9	-2.5
**254.2**	**750.6**	**1389.7**	**2046.5**	**2207.6**	**2327.9**	**2118.9**	**2153.1**	**2240.3**
	0.1	0.4	3.5	4.7	6.2	3.7	4.1	4.7
82.0	192.5	426.1	534.4	541.2	455.6	499.4	478.5	586.8
1.0	0.5	8.9	6.3	7.5	7.2	6.2	6.5	7.2
	0.5	16.5	48.7	54.7	55.5	56.7	56.6	61.0
6.6	17.4	55.5	99.0	113.9	131.6	51.4	63.2	72.6
6.1	5.7	24.0	25.8	29.4	33.8	44.7	48.6	51.0
158.5	534.0	858.3	1328.7	1456.2	1637.9	1457.0	1495.7	1457.0
**4.3**	**23.7**	**6.5**	**5.7**	**44.9**	**-7.6**	**-4.7**	**4.6**	**93.5**

?1994-2018 China Academic Journal Electronic Publishing House. All rights reserved. http://www.cnki.net

5-13 天然气平衡表

单位：亿立方米

项 目	Item	1980	1985
**可供量**	**Total Energy Available for Consumption**	**142.7**	**129.3**
生产量	Output	142.7	129.3
进口量	Imports		
出口量(-)	Exports (-)		
年初年末库存差额	Stock Changes in the Year		
**消费量**	**Total Energy Consumption**	**140.6**	**129.3**
在消费量中:	Consumption by Sector		
1.农、林、牧、渔业	Farming, Forestry, Animal Husbandry, Fishery Conservancy		
2.工业	Industry	131.4	109.6
3.建筑业	Construction	6.0	14.1
4.交通运输、仓储和邮政业	Transport, Storage and Post	0.7	0.8
5.批发、零售业和住宿、餐饮业	Wholesale, Retail Trade and Hotel, Restaurants		
6.其他	Others	0.5	0.5
7.生活消费	Residential Consumption	2.0	4.3
**平衡差额**	**Balance**	**2.1**	

注：从2010年起包括液化天然气数据。

5-14 电力平衡表

单位：亿千瓦小时

项 目	Item	1980	1985
**可供量**	**Total Energy Available for Consumption**	**3006.3**	**4117.6**
生产量	Output	3006.3	4106.9
水 电	Hydropower	582.1	923.7
火 电	Thermal Power	2424.2	3183.2
核 电	Nuclear Power		
风 电	Wind Power		
进口量	Imports		11.1
出口量(-)	Exports (-)		0.4
**消费量**	**Total Energy Consumption**	**3006.3**	**4117.6**
在消费量中:	Consumption by Sector		
1.农、林、牧、渔业	Farming, Forestry, Animal Husbandry, Fishery Conservancy	270	317.4
2.工业	Industry	2471.9	3283.4
3.建筑业	Construction	47.1	71.2
4.交通运输、仓储和邮政业	Transport, Storage and Post	26.5	63.4
5.批发、零售业和住宿、餐饮业	Wholesale, Retail Trade and Hotel, Restaurants	16.8	38.0
6.其他	Others	68.8	121.7
7.生活消费	Residential Consumption	105.2	222.5
在消费量中:	Consumption by Usage		
(一) 终端消费	(I)Final Consumption	2763.4	3813.3
#工 业	Industry	2229.0	2979.1
(二) 输配电损失量	(II)Losses in Transmission	242.9	304.3

?1994-2018 China Academic Journal Electronic Publishing House. All rights reserved. http://www.cnki.net

NATURAL GAS BALANCE SHEET

(100 million cu.m)

1990	1995	2000	2005	2006	2007	2008	2009	2010
**153.0**	**179.5**	**240.6**	**463.5**	**566.1**	**706.6**	**816.6**	**896.9**	**1072.9**
153.0	179.5	272.0	493.2	585.5	692.4	803.0	852.7	948.5
				9.5	40.2	46.0	76.3	164.7
		31.4	29.7	29.0	26.0	32.5	32.1	40.3
**152.5**	**177.4**	**245.0**	**467.6**	**561.4**	**705.2**	**812.9**	**895.2**	**1075.8**
								0.5
120.2	154.4	199.0	328.8	384.0	479.7	531.6	577.9	687.3
10.6	0.3	0.8	1.5	1.7	2.1	1.0	1.0	1.2
1.9	1.6	8.8	38.0	47.2	46.9	71.6	91.1	106.7
	0.6	3.4	10.8	13.2	17.1	17.8	24.0	27.2
1.2	1.2	0.6	9.1	12.8	16.1	20.9	23.6	26.0
18.6	19.4	32.3	79.4	102.6	143.4	170.1	177.7	226.9
**0.5**	**2.1**	**-4.4**	**-4.1**	**4.6**	**1.4**	**3.7**	**1.7**	**3.5**

a) Include the data of LNG since 2010.

ELECTRICITY BALANCE

(100 million kW•h)

1990	1995	2000	2005	2006	2007	2008	2009	2010
**6230.4**	**10023.4**	**13472.7**	**24940.8**	**28588.4**	**32712.4**	**34540.8**	**37032.7**	**41936.5**
6212.0	10077.3	13556.0	25002.6	28657.3	32815.5	34668.8	37146.5	42071.6
1267.2	1905.8	2224.1	3970.2	4357.9	4852.6	5851.9	6156.4	7221.7
4944.8	8043.2	11141.9	20473.4	23696.0	27229.3	27900.8	29827.8	33319.3
	128.3	167.4	530.9	548.4	621.3	683.9	701.3	738.8
								446.2
19.3	6.4	15.5	50.1	53.9	42.5	38.4	60.1	55.5
0.9	60.3	98.8	111.9	122.7	145.7	166.4	173.9	190.6
**6230.4**	**10023.4**	**13472.4**	**24940.3**	**28588.0**	**32711.8**	**34541.4**	**37032.2**	**41934.5**
426.8	582.4	533.0	776.3	827.0	879.0	887.1	939.9	976.5
4873.3	7659.8	10004.6	18521.7	21267.7	24290.8	25388.6	26854.5	30871.8
65.0	159.6	159.8	233.9	271.0	309.0	367.3	421.9	483.2
105.9	182.3	281.2	430.3	467.4	531.9	571.8	617.0	734.5
76.2	199.5	418.7	752.3	847.3	929.8	1017.4	1136.8	1292.0
202.4	234.2	623.2	1340.9	1555.9	1708.6	1913.0	2189.9	2451.8
480.8	1005.6	1452.0	2884.8	3351.6	4062.7	4396.1	4872.2	5124.6
5795.8	9278.9	12535.7	23233.8	26729.1	30650.1	32403.5	34773.9	39366.3
4438.7	6915.3	9067.9	16815.2	19408.9	22229.1	23250.8	24596.3	28303.5
434.6	744.5	936.7	1706.5	1858.8	2061.7	2137.9	2258.2	2568.2

?1994-2018 China Academic Journal Electronic Publishing House. All rights reserved. http://www.cnki.net

5-1 中国能源平衡表(实物量)-2015

项　目	Item	煤合计 (万吨) Coal Total (10^4 tons)	原煤 (万吨) Raw Coal (10^4 tons)
**一.可供本地区消费的能源量**	**Total Primary Energy Supply**	**397073.75**	**396371.63**
1.一次能源生产量	Indigenous Production	374654.16	374654.16
水电	Hydro Power		
核电	Nuclear Power		
风电	Wind Power		
2.进口量	Import	20406.48	20401.30
3.境内轮船和飞机在境外的加油量	Domestic Airplanes&Ships Refueling in Abroad		
4.出口量(-)	Export (-)	533.80	519.73
5.境外轮船和飞机在境内的加油量(-)	Oversea Airplanes&Ships Refueling in China (-)		
6.库存增(-)、减(+)量	Stock Change	2546.91	1835.90
**二.加工转换投入(-)产出(+)量**	**Input(-) & Output(+) of Transformation**	**-284818.72**	**-302290.15**
1.火力发电	Thermal Power	-179318.38	-176643.54
2.供热	Heating Supply	-24095.38	-23370.24
3.洗选煤	Coal Washing	-18337.66	-93395.97
4.炼焦	Coking	-60643.56	-6891.22
5.炼油及煤制油	Petroleum Refineries	-679.03	-518.32
#油品再投入量(-)	Petroleum Products Input (-)		
6.制气	Gas Works	-1270.45	-1132.67
#焦炭再投入量(-)	Coke Input (-)		
7.天然气液化	Natural Gas Liquefaction		
8.煤制品加工	Briquettes	-474.26	-338.19
9.回收能	Recovery of Energy		
**三.损失量**	**Loss**		
**四.终端消费量**	**Total Final Consumption**	**112195.35**	**94027.49**
1.农、林、牧、渔业	Agriculture, Forestry, Animal Husbandry and Fishery	2625.00	2581.03
2.工业	Industry	90831.25	74393.53
#用作原料、材料	Non-Energy Use	9451.26	8354.15
3.建筑业	Construction	878.06	851.29
4.交通运输、仓储和邮政业	Transport, Storage and Post	491.60	460.97
5.批发、零售业和住宿、餐饮业	Wholesale, Retail Trade and Hotel, Restaurants	3863.65	3767.18
6.其他	Others	4158.66	4043.84
7.生活消费	Residential Consumption	9347.13	7929.65
城镇	Urban	1361.50	990.12
乡村	Rural	7985.63	6939.53
**五.平衡差额**	**Statistical Difference**	**59.68**	**53.99**
**六.消费量合计**	**Total Energy Consumption**	**397014.07**	**396317.64**

?1994-2018 China Academic Journal Electronic Publishing House. All rights reserved. http://www.cnki.net

Energy Balance of China (Physical Quantity) -2015

洗精煤 (万吨) Cleaned Coal (10^4 tons)	其他洗煤 (万吨) Other Washed Coal (10^4 tons)	型煤 (万吨) Briquettes (10^4 tons)	煤矸石 (万吨) Gangue (10^4 tons)	焦炭 (万吨) Coke (10^4 tons)	焦炉煤气 (亿立方米) Coke Oven Gas (10^8 cu.m)	高炉煤气 (亿立方米) Blast Furnace Gas (10^8 cu.m)	转炉煤气 (亿立方米) Converter Gas (10^8 cu.m)	其他煤气 (亿立方米) Other Gas (10^8 cu.m)
**614.85**	**99.26**	**-11.99**		**-803.63**				
		5.18		0.38				
		14.07		964.82				
614.85	99.26	-3.10		160.81				
**5261.27**	**10284.23**	**1925.93**	**-11.50**	**44538.75**	**590.74**	**5520.91**	**360.31**	**153.19**
-31.82	-2643.02		-2709.60	-5.92	-169.63	-1465.75	-113.46	-2.80
-56.03	-669.11		-725.34	-276.03	-60.85	-620.23	-45.40	-0.51
59304.68	15753.63		3423.44					
-53674.91	-77.43			44633.92	817.32			
-149.89	-10.82							
-130.76	-7.02			188.62	3.90			156.50
				-1.84				
	-2062.00	1925.93						
						7606.89	519.17	
**5854.30**	**10408.35**	**1905.21**		**43774.95**	**588.51**	**5522.55**	**356.90**	**153.09**
	43.97			49.49				
5837.17	9281.06	1319.49		43639.19	566.70	5522.55	356.90	86.29
663.69	433.43			1745.83	14.53			
8.09	18.68			6.68				
9.04	21.59			3.02				
	62.08	34.39		40.06	0.91			6.14
	102.25	12.57		5.35	1.18			
	878.72	538.77		31.16	19.72			60.66
	203.44	167.94		9.17	19.72			60.46
	675.28	370.82		21.99				0.20
**21.82**	**-24.86**	**8.73**	**-11.50**	**-39.83**	**2.23**	**-1.64**	**3.41**	**0.10**
**59897.71**	**15877.75**	**1905.21**	**3434.94**	**44058.74**	**818.99**	**7608.53**	**515.76**	**156.40**

?1994-2018 China Academic Journal Electronic Publishing House. All rights reserved. http://www.cnki.net

5-1 续表 1

项 目	Item	其他焦化产品 (万吨) Other Coking Products (10^4 tons)	油品合计 (万吨) Petroleum Products Total (10^4 tons)
**一.可供本地区消费的能源量**	**Total Primary Energy Supply**		**55188.00**
1.一次能源生产量	Indigenous Production		21455.58
水电	Hydro Power		
核电	Nuclear Power		
风电	Wind Power		
2.进口量	Import		38824.29
3.境内轮船和飞机在境外的加油量	Domestic Airplanes&Ships Refueling in Abroad		924.33
4.出口量(-)	Export (-)		4373.46
5.境外轮船和飞机在境内的加油量(-)	Oversea Airplanes&Ships Refueling in China (-)		754.69
6.库存增(-)、减(+)量	Stock Change		-888.05
**二.加工转换投入(-)产出(+)量**	**Input(-) & Output(+) of Transformation**	**1074.33**	**-2626.94**
1.火力发电	Thermal Power		-265.54
2.供热	Heating Supply		-493.21
3.洗选煤	Coal Washing		
4.炼焦	Coking	1183.11	
5.炼油及煤制油	Petroleum Refineries	-121.43	7133.09
#油品再投入量(-)	Petroleum Products Input (-)		-9001.28
6.制气	Gas Works	21.87	
#焦炭再投入量(-)	Coke Input (-)	-9.22	
7.天然气液化	Natural Gas Liquefaction		
8.煤制品加工	Briquettes		
9.回收能	Recovery of Energy		
**三.损失量**	**Loss**		**87.56**
**四.终端消费量**	**Total Final Consumption**	**1077.23**	**52445.68**
1.农、林、牧、渔业	Agriculture, Forestry, Animal Husbandry and Fishery		1733.41
2.工业	Industry	1077.23	16229.74
#用作原料、材料	Non-Energy Use	392.67	8254.95
3.建筑业	Construction		3507.50
4.交通运输、仓储和邮政业	Transport, Storage and Post		20513.75
5.批发、零售业和住宿、餐饮业	Wholesale, Retail Trade and Hotel, Restaurants		615.70
6.其他	Others		3683.34
7.生活消费	Residential Consumption		6162.25
城镇	Urban		4305.58
乡村	Rural		1856.67
**五.平衡差额**	**Statistical Difference**	**-2.90**	**27.82**
**六.消费量合计**	**Total Energy Consumption**	**1207.88**	**55160.18**

?1994-2018 China Academic Journal Electronic Publishing House. All rights reserved. http://www.cnki.net

Continued 1

原油 (万吨) Crude Oil (10^4 tons)	汽油 (万吨) Gasoline (10^4 tons)	煤油 (万吨) Kerosene (10^4 tons)	柴油 (万吨) Diesel Oil (10^4 tons)	燃料油 (万吨) Fuel Oil (10^4 tons)	石脑油 (万吨) Naphtha (10^4 tons)	润滑油 (万吨) Lubricants (10^4 tons)	石蜡 (万吨) Paraffin Waxes (10^4 tons)	溶剂油 (万吨) White Spirit (10^4 tons)
**54093.50**	**-718.52**	**-926.01**	**-654.27**	**669.77**	**632.72**	**20.72**	**-53.86**	**-7.06**
21455.58								
33548.28	17.03	348.45	42.80	1540.40	664.72	32.56	8.49	2.21
		367.93	28.65	527.75				
286.56	589.29	1237.33	716.25	1051.69		11.84	62.35	0.42
		389.29	15.00	350.40				
-623.80	-146.26	-15.77	5.53	3.71	-32.00			-8.85
**-53218.37**	**12103.01**	**3658.62**	**17928.02**	**1424.73**	**3934.63**	**113.58**	**161.33**	**147.26**
-12.46	-0.17		-22.36	-31.52				
-6.69	-0.07		-6.22	-165.13				
-53199.22	12103.56	3658.62	18007.89	3963.01	4585.04	117.17	161.35	147.26
	-0.31		-51.29	-2341.63	-650.41	-3.59	-0.02	
**87.22**								
**782.69**	**11367.91**	**2663.71**	**17280.44**	**2123.73**	**4573.66**	**133.80**	**108.20**	**142.00**
	231.33	1.10	1492.88	0.94				
782.69	476.53	21.16	1436.50	594.75	4573.66	133.80	108.20	142.00
135.66	9.33	1.94	27.50	131.74	4249.48	116.67	105.54	136.46
	408.57	12.50	555.71	53.51				
	5306.59	2504.88	11162.80	1439.49				
	243.29	11.68	257.74	18.95				
	2108.47	83.27	1384.15	16.08				
	2593.11	29.13	990.66					
	1804.08	3.74	549.70					
	789.03	25.39	440.96					
**5.22**	**16.58**	**68.90**	**-6.69**	**-29.23**	**-6.31**	**0.50**	**-0.73**	**-1.80**
**54088.28**	**11368.46**	**2663.71**	**17360.31**	**4662.01**	**5224.07**	**137.39**	**108.22**	**142.00**

?1994-2018 China Academic Journal Electronic Publishing House. All rights reserved. http://www.cnki.net

5-1 续表 2

项 目	Item	石油沥青 (万吨) Bitumen Asphalt (10^4 tons)	石油焦 (万吨) Petroleum Coke (10^4 tons)
**一.可供本地区消费的能源量**	**Total Primary Energy Supply**	**441.89**	**361.84**
1.一次能源生产量	Indigenous Production		
水电	Hydro Power		
核电	Nuclear Power		
风电	Wind Power		
2.进口量	Import	470.59	588.76
3.境内轮船和飞机在境外的加油量	Domestic Airplanes&Ships Refueling in Abroad		
4.出口量(-)	Export (-)	28.70	241.92
5.境外轮船和飞机在境内的加油量(-)	Oversea Airplanes&Ships Refueling in China (-)		
6.库存增(-)、减(+)量	Stock Change		15.00
**二.加工转换投入(-)产出(+)量**	**Input(-) & Output(+) of Transformation**	**2007.80**	**1613.62**
1.火力发电	Thermal Power		-136.27
2.供热	Heating Supply		-150.51
3.洗选煤	Coal Washing		
4.炼焦	Coking		
5.炼油及煤制油	Petroleum Refineries	2016.52	1900.40
#油品再投入量(-)	Petroleum Products Input (-)	-8.72	
6.制气	Gas Works		
#焦炭再投入量(-)	Coke Input (-)		
7.天然气液化	Natural Gas Liquefaction		
8.煤制品加工	Briquettes		
9.回收能	Recovery of Energy		
**三.损失量**	**Loss**		
**四.终端消费量**	**Total Final Consumption**	**2451.09**	**1975.82**
1.农、林、牧、渔业	Agriculture, Forestry, Animal Husbandry and Fishery		
2.工业	Industry	68.79	1975.82
#用作原料、材料	Non-Energy Use	39.58	1351.11
3.建筑业	Construction	2382.30	
4.交通运输、仓储和邮政业	Transport, Storage and Post		
5.批发、零售业和住宿、餐饮业	Wholesale, Retail Trade and Hotel, Restaurants		
6.其他	Others		
7.生活消费	Residential Consumption		
城镇	Urban		
乡村	Rural		
**五.平衡差额**	**Statistical Difference**	**-1.40**	**-0.36**
**六.消费量合计**	**Total Energy Consumption**	**2459.81**	**2262.60**

?1994-2018 China Academic Journal Electronic Publishing House. All rights reserved. http://www.cnki.net

Continued 2

液化石油气 (万吨) LPG (10^4 tons)	炼厂干气 (万吨) Refinery Gas (10^4 tons)	其他石油制品 (万吨) Other Petroleum Products (10^4 tons)	天然气 (亿立方米) Natural Gas (10^8 cu.m)	液化天然气 (万吨) LNG (10^4 tons)	热力 (万百万千焦) Heat (10^{10} kJ)	电力 (亿千瓦小时) Electricity (10^8 kW•h)	其他能源 (万吨标煤) Other Energy (10^4 tce)
**1073.80**		**253.48**	**1654.29**	**1961.87**		**15179.41**	**5751.10**
			1346.10			15303.85	5751.10
						11302.70	
						1707.89	
						1857.66	
1243.95		316.05	340.64	1961.87		62.10	
144.15		2.96	32.45			186.54	
-26.00		-59.61					
**2552.12**	**1570.69**	**3376.02**	**-430.66**	**345.57**	**401604.52**	**42841.88**	**-223.42**
	-50.42	-12.34	-291.59	-163.12	-59452.63	42841.88	-1058.97
-3.27	-131.71	-29.61	-61.70	-9.84	399029.66		-310.67
2934.42	1853.65	8883.42	-3.75				-38.86
-379.03	-100.83	-5465.45					
			3.24				
			-76.86	518.53			
					62027.49		1185.08
**0.34**			**20.38**	**13.00**	**4875.29**	**2987.86**	
**3578.56**	**1576.01**	**3688.06**	**1209.66**	**2296.70**	**396778.72**	**55032.12**	**5526.78**
7.16			0.95		106.70	1039.83	492.73
731.57	1576.01	3608.26	559.36	2040.00	280611.96	38562.13	1231.53
233.99	34.20	1681.75	96.94	182.17			
15.10		79.80	2.16		903.67	698.67	35.65
99.98			190.65	256.70	2809.72	1125.61	1171.85
84.04			51.29		6113.91	2122.04	92.52
91.36			45.44		12391.77	3918.63	298.37
2549.35			359.81		93840.97	7565.21	2204.13
1948.06			358.38		93840.97	4103.94	249.53
601.29			1.43			3461.27	1954.60
**47.02**	**-5.32**	**-58.56**	**-6.41**	**-2.26**	**-49.49**	**1.31**	**0.90**
**3961.20**	**1858.97**	**9195.46**	**1589.14**	**2482.66**	**461106.64**	**58019.98**	**6935.28**

?1994-2018 China Academic Journal Electronic Publishing House. All rights reserved.   http://www.cnki.net

5-2 中国能源平衡表(标准量) -2015

单位：万吨标准煤

项目	Item	能源合计 (发电煤耗计算法) (coal equivalent calculation)	Energy Total (电热当量计算法) (calorific value calculation)
**一.可供本地区消费的能源量**	**Total Primary Energy Supply**	**429960.16**	**402217.03**
1.一次能源生产量	Indigenous Production	361475.51	333504.95
水电	Hydro Power	34548.76	13891.02
核电	Nuclear Power	5220.48	2099.00
风电	Wind Power	5678.28	2283.06
2.进口量	Import	76114.18	76000.68
3.境内轮船和飞机在境外的加油量	Domestic Airplanes&Ships Refueling in Abroad	1337.06	1337.06
4.出口量(-)	Export (-)	8688.77	8347.84
5.境外轮船和飞机在境内的加油量(-)	Oversea Airplanes&Ships Refueling in China (-)	1095.24	1095.24
6.库存增(-)、减(+)量	Stock Change	817.41	817.41
**二.加工转换投入(-)产出(+)量**	**Input(-) & Output(+) of Transformation**	**-2698.85**	**-81000.17**
1.火力发电	Thermal Power		-78301.32
2.供热	Heating Supply	-5291.00	-5291.00
3.洗选煤	Coal Washing	-4996.49	-4996.49
4.炼焦	Coking	-4098.80	-4098.80
5.炼油及煤制油	Petroleum Refineries	10259.45	10259.45
#油品再投入量(-)	Petroleum Products Input (-)	-12489.85	-12489.85
6.制气	Gas Works	-318.65	-318.65
#焦炭再投入量(-)	Coke Input (-)	-12.43	-12.43
7.天然气液化	Natural Gas Liquefaction	-88.02	-88.02
8.煤制品加工	Briquettes	-154.77	-154.77
9.回收能	Recovery of Energy	14491.71	14491.71
**三.损失量**	**Loss**	**9712.15**	**4251.30**
**四.终端消费量**	**Total Final Consumption**	**417494.08**	**316912.88**
1.农、林、牧、渔业	Agriculture, Forestry, Animal Husbandry and Fishery	8231.66	6331.18
2.工业	Industry	280205.66	209726.35
#用作原料、材料	Non-Energy Use	22100.70	22100.70
3.建筑业	Construction	7696.41	6419.47
4.交通运输、仓储和邮政业	Transport, Storage and Post	37976.93	35919.67
5.批发、零售业和住宿、餐饮业	Wholesale, Retail Trade and Hotel, Restaurants	11403.69	7525.28
6.其他	Others	21880.78	14718.77
7.生活消费	Residential Consumption	50098.96	36272.17
城镇	Urban	28668.14	21167.44
乡村	Rural	21430.83	15104.73
**五.平衡差额**	**Statistical Difference**	**55.07**	**52.68**
**六.消费量合计**	**Total Energy Consumption**	**429905.09**	**402164.35**

?1994-2018 China Academic Journal Electronic Publishing House. All rights reserved. http://www.cnki.net

Energy Balance of China (Standard Quantity) -2015

(10 000 tce)

煤合计 Coal Total	原煤 Raw Coal	洗精煤 Cleaned Coal	其他洗煤 Other Washed Coal	型煤 Briquettes	煤矸石 Gangue	焦炭 Coke	焦炉煤气 Coke Oven Gas	高炉煤气 Blast Furnace Gas	转炉煤气 Converter Gas
**274744.93**	**274146.24**	**553.37**	**52.61**	**-7.28**		**-780.65**			
260794.67	260794.67								
12474.98	12471.83			3.15		0.37			
471.22	462.68			8.54		937.23			
1946.50	1342.41	553.37	52.61	-1.88		156.21			
**-195909.46**	**-207264.67**	**4735.14**	**5450.64**	**1169.43**	**66.17**	**43264.94**	**3375.49**	**7099.89**	**977.88**
-119741.17	-118311.73	-28.64	-1400.80		-541.92	-5.75	-969.27	-1884.95	-307.93
-15420.58	-15015.53	-50.43	-354.63		-145.07	-268.14	-347.70	-797.62	-123.22
-5749.65	-67473.28	53374.21	8349.42		753.16				
-53491.67	-5143.21	-48307.42	-41.04			43357.39	4670.17		
-480.58	-339.95	-134.90	-5.73						
-871.03	-749.62	-117.68	-3.7206			183.23	22.28		
						-1.79			
-154.77	-231.34		-1092.86	1169.43					
								9782.46	1409.03
**78801.98**	**66859.84**	**5268.87**	**5516.42**	**1156.85**		**42522.99**	**3362.75**	**7102.00**	**968.63**
1965.56	1942.26		23.31			48.07			
62686.28	51712.67	5253.45	4918.96	801.19		42391.11	3238.12	7102.00	968.63
6804.08	5977.05	597.32	229.72			1695.90	83.02		
701.15	683.97	7.28	9.90			6.49			
349.13	329.55	8.14	11.44			2.93			
2972.64	2918.86		32.90	20.88		38.91	5.21		
3157.79	3095.96		54.19	7.63		5.20	6.74		
6969.44	6176.58		465.72	327.14		30.27	112.68		
985.17	775.37		107.82	101.98		8.91	112.68		
5984.27	5401.21		357.90	225.16		21.36			
**33.49**	**21.73**	**19.64**	**-13.17**	**5.30**	**66.17**	**-38.69**	**12.74**	**-2.11**	**9.25**

?1994-2018 China Academic Journal Electronic Publishing House. All rights reserved. http://www.cnki.net

5-2 续表 1

单位：万吨标准煤

项　目	Item	其他煤气 Gasoline	其他焦化产品 Other Coking Products
**一.可供本地区消费的能源量**	**Total Primary Energy Supply**		
1.一次能源生产量	Indigenous Production		
水电	Hydro Power		
核电	Nuclear Power		
风电	Wind Power		
2.进口量	Import		
3.境内轮船和飞机在境外的加油量	Domestic Airplanes&Ships Refueling in Abroad		
4.出口量(-)	Export (-)		
5.境外轮船和飞机在境内的加油量(-)	Oversea Airplanes&Ships Refueling in China (-)		
6.库存增(-)、减(+)量	Stock Change		
**二.加工转换投入(-)产出(+)量**	**Input(-) & Output(+) of Transformation**	**273.60**	**1239.78**
1.火力发电	Thermal Power	-5.00	
2.供热	Heating Supply	-0.91	
3.洗选煤	Coal Washing		
4.炼焦	Coking		1365.31
5.炼油及煤制油	Petroleum Refineries		-140.13
#油品再投入量(-)	Petroleum Products Input (-)		
6.制气	Gas Works	279.51	25.24
#焦炭再投入量(-)	Coke Input (-)		-10.64
7.天然气液化	Natural Gas Liquefaction		
8.煤制品加工	Briquettes		
9.回收能	Recovery of Energy		
**三.损失量**	**Loss**		
**四.终端消费量**	**Total Final Consumption**	**273.41**	**1243.12**
1.农、林、牧、渔业	Agriculture, Forestry, Animal Husbandry and Fishery		
2.工业	Industry	154.11	1243.12
#用作原料、材料	Non-Energy Use		453.14
3.建筑业	Construction		
4.交通运输、仓储和邮政业	Transport, Storage and Post		
5.批发、零售业和住宿、餐饮业	Wholesale, Retail Trade and Hotel, Restaurants	10.97	
6.其他	Others		
7.生活消费	Residential Consumption	108.33	
城镇	Urban	107.98	
乡村	Rural	0.36	
**五.平衡差额**	**Statistical Difference**	**0.19**	**-3.35**
**六.消费量合计**	**Total Energy Consumption**		

?1994-2018 China Academic Journal Electronic Publishing House. All rights reserved. http://www.cnki.net

Continued 1

(10 000 tce)

油品合计 Petroleum Products Total	原油 Crude Oil	汽油 Gasoline	煤油 Kerosene	柴油 Diesel Oil	燃料油 Fuel Oil	石脑油 Naphtha	润滑油 Lubricants	石蜡 Paraffin Waxes
**78892.98**	**77277.97**	**-1057.23**	**-1362.53**	**-953.34**	**956.83**	**949.08**	**29.30**	**-73.51**
30651.44	30651.44							
55573.30	47927.07	25.06	512.71	62.36	2200.62	997.08	46.05	11.59
1337.06			541.37	41.75	753.94			
6288.28	409.38	867.08	1820.61	1043.65	1502.44		16.75	85.10
1095.24			572.80	21.86	500.58			
-1285.30	-891.16	-215.21	-23.20	8.06	5.30	-48.00		
**-2521.08**	**-76027.76**	**17808.37**	**5383.29**	**26122.92**	**2035.37**	**5901.95**	**160.64**	**220.18**
-334.39	-17.80	-0.25		-32.58	-45.03			
-664.62	-9.56	-0.10		-9.06	-235.90			
10967.77	-76000.41	17809.18	5383.29	26239.30	5661.56	6877.56	165.71	220.21
-12489.85		-0.46		-74.73	-3345.25	-975.615	-5.077337	-0.027296
**125.19**	**124.60**							
**76185.23**	**1118.15**	**16726.74**	**3919.39**	**25179.33**	**3033.96**	**6860.49**	**189.23**	**147.67**
2530.88		340.38	1.62	2175.27	1.34			
22893.36	1118.15	701.17	31.13	2093.12	849.66	6860.49	189.23	147.67
11484.26	193.80	13.73	2.85	40.07	188.20	6374.22	165.01	144.04
4758.57		601.18	18.39	809.72	76.45			
29986.98		7808.12	3685.68	16265.32	2056.46			
921.85		357.98	17.18	375.55	27.07			
5421.37		3102.41	122.52	2016.85	22.97			
9672.20		3815.51	42.86	1443.49				
6800.55		2654.53	5.50	800.96				
2871.66		1160.98	37.36	642.53				
**61.48**	**7.46**	**24.40**	**101.37**	**-9.75**	**-41.75**	**-9.46**	**0.71**	**-1.00**

?1994-2018 China Academic Journal Electronic Publishing House. All rights reserved. http://www.cnki.net

5-2 续表 2

单位: 万吨标准煤

项目	Item	溶剂油 White spirit	石油沥青 Bitumen Asphalt
**一.可供本地区消费的能源量**	**Total Primary Energy Supply**	**-10.36**	**578.88**
1.一次能源生产量	Indigenous Production		
水电	Hydro Power		
核电	Nuclear Power		
风电	Wind Power		
2.进口量	Import	3.24	616.47
3.境内轮船和飞机在境外的加油量	Domestic Airplanes&Ships Refueling in Abroad		
4.出口量(-)	Export (-)	0.62	37.60
5.境外轮船和飞机在境内的加油量(-)	Oversea Airplanes&Ships Refueling in China (-)		
6.库存增(-)、减(+)量	Stock Change	-12.98	
**二.加工转换投入(-)产出(+)量**	**Input(-) & Output(+) of Transformation**	**216.06**	**2630.22**
1.火力发电	Thermal Power		
2.供热	Heating Supply		
3.洗选煤	Coal Washing		
4.炼焦	Coking		
5.炼油及煤制油	Petroleum Refineries	216.06	2641.64
#油品再投入量(-)	Petroleum Products Input (-)		-11.4232
6.制气	Gas Works		
#焦炭再投入量(-)	Coke Input (-)		
7.天然气液化	Natural Gas Liquefaction		
8.煤制品加工	Briquettes		
9.回收能	Recovery of Energy		
**三.损失量**	**Loss**		
**四.终端消费量**	**Total Final Consumption**	**208.34**	**3210.93**
1.农、林、牧、渔业	Agriculture, Forestry, Animal Husbandry and Fishery		
2.工业	Industry	208.34	90.12
#用作原料、材料	Non-Energy Use	200.21	51.85
3.建筑业	Construction		3120.81
4.交通运输、仓储和邮政业	Transport, Storage and Post		
5.批发、零售业和住宿、餐饮业	Wholesale, Retail Trade and Hotel, Restaurants		
6.其他	Others		
7.生活消费	Residential Consumption		
城镇	Urban		
乡村	Rural		
**五.平衡差额**	**Statistical Difference**	**-2.64**	**-1.83**
**六.消费量合计**	**Total Energy Consumption**		

?1994-2018 China Academic Journal Electronic Publishing House. All rights reserved. http://www.cnki.net

Continued 2

(10 000 tce)

石油焦 Petroleum Coke	液化石油气 LPG	炼厂干气 Refinery Gas	其他石油制品 Other Petroleum Products	天然气 Natural Gas	液化天然气 LNG	热力 Heat	电力 Electricity	其他能源 Other Energy
**379.93**	**1840.82**		**337.13**	**21505.77**	**3447.40**		**18655.49**	**5751.10**
				17499.30			18808.43	5751.10
							13891.02	
							2099.00	
							2283.06	
618.20	2132.50		420.35	4428.32	3447.40		76.32	
254.02	247.12		3.94	421.85			229.26	
15.75	-44.57		-79.28					
**1694.30**	**4375.10**	**2468.18**	**4490.11**	**-5598.58**	**607.24**	**13694.71**	**52652.67**	**-223.42**
-143.08		-79.23	-16.41	-3790.67	-286.63	-2027.33	52652.67	-1058.97
-158.04	-5.61	-206.97	-39.38	-802.10	-17.29	13606.91		-310.67
1995.42	5030.48	2912.83	11814.95	-48.75				-38.86
	-649.77	-158.44	-7269.05					
				42.12				
				-999.18	911.16			
						2115.14		1185.08
	**0.58**			**264.94**	**22.84**	**166.25**	**3672.08**	
**2074.61**	**6134.72**	**2476.54**	**4905.12**	**15725.59**	**4035.77**	**13530.15**	**67634.48**	**5526.78**
	12.27			12.34		3.64	1277.95	492.73
2074.61	1254.13	2476.54	4798.99	7271.68	3584.69	9568.87	47392.86	1231.53
1418.67	401.13	53.74	2236.73	1260.18	320.11			
	25.89		106.13	28.12		30.82	858.67	35.65
	171.40			2478.51	451.08	95.81	1383.37	1171.85
	144.07			666.71		208.48	2607.99	92.52
	156.62			590.74		422.56	4816.00	298.37
	4370.35			4677.49		3199.98	9297.64	2204.13
	3339.56			4658.91		3199.98	5043.74	249.53
	1030.79			18.58			4253.90	1954.60
**-0.38**	**80.61**	**-8.36**	**-77.88**	**-83.34**	**-3.98**	**-1.69**	**1.61**	**0.90**

?1994-2018 China Academic Journal Electronic Publishing House. All rights reserved. http://www.cnki.net

5-3 综合能源平衡表

单位：万吨标准煤

项　　目	Item	1980	1985	1990
**可供消费的能源总量**	**Total Energy Available for Consumption**	**61557**	**77603**	**96138**
一次能源生产量	Primary Energy Output	63735	85546	103922
回收能	Recovery of Energy			
进口量	Imports	261	340	1310
出口量(-)	Exports (-)	3058	5774	5875
年初年末库存差额	Stock Changes in the Year	619	-2509	-3219
**能源消费总量**	**Total Energy Consumption**	**60275**	**76682**	**98703**
在总量中:	Consumption by Sector			
1.农、林、牧、渔业	Agriculture, Forestry, Animal Husbandry and Fishery	4692	4045	4852
2.工业	Industry	38986	51068	67578
3.建筑业	Construction	957	1302	1213
4.交通运输、仓储和邮政业	Transport, Storage and Post	2902	3713	4541
5.批发、零售业和住宿、餐饮业	Wholesale, Retail Trade and Hotel, Restaurants	518	766	1247
6.其他	Others	1205	2470	3473
7.生活消费	Residential Consumption	11015	13318	15799
在总量中:	Consumption by Usage			
(一) 终端消费	(I) Final Consumption	57508	73586	94289
#工业	Industry	38293	48021	63239
(二) 加工转换损失量	(II) Losses in Processing and	1358	1491	2264
#炼焦	Coking	644	572	905
炼油	Petroleum Refining	113	110	326
(三) 回收能(-)	(III) Recovery of Energy(-)			
(四) 损失量	(IV) Other Losses	1409	1605	2150
**平衡差额**	**Balance**	**1282**	**921**	**-2565**

注：1.村办工业包括在工业中(下同)。
2.电力按等价热值折算，因此加工转换损失量中不包括发电损失量。
3.进口量包括境内轮船和飞机在境外的加油量；出口量包括境外轮船和飞机在境内的加油量。

?1994-2018 China Academic Journal Electronic Publishing House. All rights reserved. http://www.cnki.net

Overall Energy Balance Sheet

(10 000 tce)

1995	2000	2005	2010	2011	2012	2013	2014	2015
**129535**	**144234**	**254619**	**365588**	**390394**	**407594**	**417415**	**426095**	**429960**
129034	138570	229037	312125	340178	351041	358784	361866	361476
2312	3087	7452	8958					
5456	14327	26823	57671	65437	68701	73420	77325	77451
6776	9327	11257	8803	8449	7374	8005	8271	9784
-491	-2424	2564	-4363	-6772	-4773	-6784	-4825	817
**131176**	**146964**	**261369**	**360648**	**387043**	**402138**	**416913**	**425806**	**429905**
5505	4233	6860	7266	7675	7804	8055	8094	8232
96191	103014	187914	261377	278048	284712	291130	295686	292276
1335	2207	3486	5533	6052	6337	7017	7520	7696
5863	11447	19136	27102	29694	32561	34819	36336	38318
2018	3251	5917	7847	9147	10012	10598	10873	11404
4519	6118	10484	15052	16843	18407	19763	20084	21881
15745	16695	27573	36470	39584	42306	45531	47212	50099
124252	140476	250877	337469	373296	386888	403814	413162	417494
89473	96871	177775	238652	264698	269900	278514	283420	280206
3634	2472	3882	14294	15412	16763	15994	17020	17191
	526	855	1595	1833	2179	2433	2731	4099
	781	1273	1960	1792	2153	1899	2115	2230
				10864	11239	13333	14578	14492
3289	4016	6610	8885	9199	9726	10439	10201	9712
**-1641**	**-2730**	**-6751**	**4940**	**3350**	**5456**	**502**	**289**	**55**

a) Data on industry include the data of village-run industry.(The same as in the following tables).
b) Electric power is converted on the basic of equal caloric value. Therefore, losses in processing and transformation exclude losses in power generation.
c) Data on imports include the petroleum consumed by the domestic airplanes and ships in refueling abroad. Data on exports include the petroleum consumed by the oversea airplanes and ships in refueling in China.

?1994-2018 China Academic Journal Electronic Publishing House. All rights reserved. http://www.cnki.net

5-4 煤炭平衡表

单位：万吨

项目	Item	1980	1985	1990
**可供量**	**Total Energy Available for Consumption**	**62601**	**82777**	**102221**
生产量	Output	62015	87228	107988
进口量	Imports	199	231	200
出口量(-)	Exports (-)	632	777	1729
年初年末库存差额	Stock Changes in the Year	1019	-3906	-4239
**消费量**	**Total Energy Consumption**	**61010**	**81603**	**105523**
在消费量中:	Consumption by Sector			
1.农、林、牧、渔业	Agriculture, Forestry, Animal Husbandry and Fishery	1550	2209	2095
2.工业	Industry	43848	58613	81091
3.建筑业	Construction	556	532	438
4.交通运输、仓储和邮政业	Transport, Storage and Post	1934	2307	2161
5.批发、零售业和住宿、餐饮业	Wholesale, Retail Trade and Hotel, Restaurants	455	738	1058
6.其他	Others	1091	1580	1980
7.生活消费	Residential Consumption	11574	15624	16700
在消费量中:	Consumption by Usage			
(一) 终端消费	(I) Final Consumption	38804	52704	60206
#工业	Industry	21643	29715	35774
(二) 中间消费	(II) Intermediate Consumption			
(用于加工转换)	(Consumed in Transformation)	22205	28899	45317
#发电	Power Generation	12648	16441	27204
供热	Heating		1462	2996
炼焦	Coking	6682	7304	10698
炼油及煤制油	Petroleum Refineries			
制气	Gas Production	131	191	360
(三)洗选损耗	(III) Losses in Coal Washing and Dressing	2744	3501	4059
**平衡差额**	**Balance**	**1592**	**1174**	**-3302**

注：生产量为原煤产量。

?1994-2018 China Academic Journal Electronic Publishing House. All rights reserved. http://www.cnki.net

Coal Balance Sheet

(10 000 tons)

1995	2000	2005	2010	2011	2012	2013	2014	2015
**133462**	**131895**	**235508**	**355578**	**393058**	**418654**	**425015**	**411834**	**397074**
136073	138418	236515	342845	376444	394513	397432	387392	374654
164	218	2622	18307	22236	28841	32702	29122	20406
2862	5506	7173	1911	1467	927	751	574	534
87	-1235	3545	-3663	-4155	-3772	-4368	-4106	2547
**137677**	**135690**	**243375**	**349008**	**388961**	**411727**	**424426**	**411613**	**397014**
1857	1051	1802	2147	2207	2266	2451	2579	2625
117571	121807	224766	329728	368916	391191	403157	390497	375650
440	537	604	731	797	767	811	914	878
1315	882	811	639	646	614	615	558	492
977	1461	2627	3192	3572	3752	3966	3767	3864
1987	1495	2727	3412	3612	3883	4136	4046	4159
13530	8457	10039	9159	9212	9253	9290	9253	9347
66156	50511	86386	114826	120647	118957	119491	116044	112195
46050	36628	67776	95546	100602	98421	98222	94928	90831
71520	81987	152208	222948	252691	266016	282355	272194	266481
44440	55811	103663	153742	175579	183531	195177	184525	179318
5887	8794	13542	17553	19334	23780	22710	22445	24095
18396	16496	33446	49950	56060	56768	62536	62894	60644
			213	346	378	459	650	679
764	960	1277	1040	870	849	846	948	1270
2033	3191	4782	11235	15623	26754	22579	23375	18338
**-4215**	**-3795**	**-7868**	**6569**	**4097**	**6928**	**589**	**220**	**60**

a) Data on output refer to the output of raw coal.

?1994-2018 China Academic Journal Electronic Publishing House. All rights reserved. http://www.cnki.net

5-5 焦炭平衡表

单位: 万吨

项目	Item	1980	1985	1990
**可供量**	**Total Energy Available for Consumption**	**4315.3**	**4689.7**	**7085.8**
生产量	Output	4343.0	4802.1	7328.3
进口量	Imports		2.1	
出口量(-)	Exports (-)	27.1	36.9	129.0
年初年末库存差额	Stock Changes in the Year	-0.6	-77.6	-113.5
**消费量**	**Total Energy Consumption**	**4303.0**	**4689.7**	**6914.7**
在消费量中:	Consumption by Sector			
1.农、林、牧、渔业	Agriculture, Forestry, Animal Husbandry and Fishery	10.6	20.8	60.1
2.工业	Industry	4266.7	4627.7	6808.8
3.建筑业	Construction	11.9	7.8	5.2
4.交通运输、仓储和邮政业	Transport, Storage and Post	8.2	5.7	4.1
5.批发、零售业和住宿、餐饮业	Wholesale, Retail Trade and Hotel, Restaurants	0.9	2.7	7.7
6.其他	Others	4.7	2.0	1.9
7.生活消费	Residential Consumption		23.0	26.9
在消费量中:	Consumption by Usage			
(一) 终端消费	(I) Final Consumption	4294.7	4677.9	6846.3
#工业	Industry	4258.4	4615.9	6740.4
(二) 中间消费	(II) Intermediate Consumption			
(用于加工转换)	(Consumed in Transformation)	8.3	11.8	68.4
制气	Gas Production	8.3	11.8	68.4
(三) 损失量	(III) Losses in Coal Washing and Dressing			
**平衡差额**	**Balance**	**12.3**		**171.1**

?1994-2018 China Academic Journal Electronic Publishing House. All rights reserved. http://www.cnki.net

Coke Balance Sheet

(10 000 tons)

1995	2000	2005	2010	2011	2012	2013	2014	2015
**12207.1**	**10892.3**	**25084.4**	**38707.1**	**42085.8**	**44813.7**	**45850.1**	**46894.3**	**44018.9**
13424.5	12184.0	26511.7	38657.8	43433.0	43831.4	48347.8	47980.9	44822.5
0.1		0.5	11.0	11.6	7.6	3.5	0.2	0.4
886.1	1519.7	1276.4	335.0	329.7	102.0	467.3	850.7	964.8
-331.4	228.0	-151.4	373.3	-1029.0	1076.7	-2034.0	-236.0	160.8
**10725.3**	**10840.8**	**25105.8**	**38702.8**	**42063.3**	**44805.2**	**45851.9**	**46884.9**	**44058.7**
128.6	70.9	63.5	46.8	54.1	57.5	69.2	34.9	49.5
10412.0	10554.6	24860.9	38598.7	41952.1	44694.8	45694.0	46749.6	43923.0
10.8	19.0	18.4	5.8	4.8	6.3	7.7	9.7	6.7
10.1	11.2	1.1	0.1	0.1	0.1	2.2	2.7	3.0
25.7	35.7	64.1	5.1	9.2	6.7	35.8	46.6	40.1
6.4	12.2	7.6	2.8	1.9	1.9	5.0	5.1	5.4
131.6	137.2	90.3	43.5	41.1	37.9	38.0	36.4	31.2
10648.0	10697.9	24877.9	38574.6	41954.3	44738.5	45817.5	46589.1	43775.0
10334.7	10411.7	24633.0	38470.5	41843.1	44628.1	45659.6	46453.8	43639.2
77.3	142.9	227.9	128.2	109.0	66.7	34.4	295.8	283.8
77.3	142.9	227.9	128.2	109.0	66.7	34.4	32.8	1.8
**1481.8**	**51.6**	**-21.4**	**4.3**	**22.6**	**8.5**	**-1.8**	**9.4**	**-39.8**

?1994-2018 China Academic Journal Electronic Publishing House. All rights reserved. http://www.cnki.net

5-6 石油平衡表

单位：万吨

项　　目	Item	1980	1985	1990
**可供量**	**Total Energy Available for Consumption**	**8794.5**	**9193.7**	**11435.0**
生产量	Output	10594.6	12489.5	13830.6
进口量	Imports	82.7	90.0	755.6
出口量(-)	Exports (-)	1806.2	3630.4	3110.4
年初年末库存差额	Stock Changes in the Year	-76.6	244.6	-40.8
**消费量**	**Total Energy Consumption**	**8757.4**	**9168.8**	**11485.6**
在消费量中:	Consumption by Sector			
1.农、林、牧、渔业	Agriculture, Forestry, Animal Husbandry and Fishery	814..9	758.7	1033.6
2.工业	Industry	6203.2	6171.4	7321.6
3.建筑业	Construction	175.2	292.2	327.3
4.交通运输、仓储和邮政业	Transport, Storage and Post	911.5	1176.4	1683.2
5.批发、零售业和住宿、餐饮业	Wholesale, Retail Trade and Hotel, Restaurants	29.0	38.1	77.6
6.其他	Others	481.7	506.1	757.8
7.生活消费	Residential Consumption	141.9	225.9	284.5
在消费量中:	Consumption by Usage			
(一) 终端消费	(I) Final Consumption	6311.0	7063.3	9304.7
#工业	Industry	3780.3	4462.0	5180.4
(二) 中间消费	(II) Intermediate Consumption			
(用于加工转换)	(Consumed in Transformation)	2183.6	1858.5	1630.4
发电	Power Generation	2065.4	1425.5	1234.4
供热	Heating		285.6	356.3
制气	Gas Production	36.7	34.5	39.7
炼油损失量	Losses in Petroleum Refining	81.5	112.9	295.8
(三) 损失量	(III) Other Losses	262.8	247.0	254.7
**平衡差额**	**Balance**	**37.1**	**24.9**	**-50.6**

注：1.生产量为原油产量。
2.进口量包括境内轮船和飞机在境外的加油量；出口量包括境外轮船和飞机在境内的加油量。

?1994-2018 China Academic Journal Electronic Publishing House. All rights reserved. http://www.cnki.net

Petroleum Balance Sheet

(10 000 tons)

1995	2000	2005	2010	2011	2012	2013	2014	2015
**16072.7**	**22631.4**	**32539.1**	**44178.4**	**45659.2**	**47864.7**	**49993.9**	**51861.8**	**55188.0**
15005.0	16300.0	18135.3	20301.4	20287.6	20747.8	20991.9	21142.9	21455.6
3673.2	9748.5	17163.2	29437.2	31593.7	33088.8	34264.8	36179.6	39748.6
2454.5	2172.1	2888.1	4079.0	4117.0	3884.3	4176.7	4213.9	5128.2
-151.0	-1245.0	128.8	-1481.2	-2105.0	-2087.6	-1086.1	-1246.8	-888.1
**16064.9**	**22495.9**	**32547.0**	**44101.0**	**45619.5**	**47797.3**	**49970.6**	**51814.4**	**55160.2**
1203.2	788.5	1451.7	1382.5	1466.3	1537.9	1650.3	1717.7	1733.4
9349.3	11248.5	14030.4	18555.0	17986.0	17753.2	17594.6	18217.5	18908.1
242.8	840.6	1502.2	2483.1	2581.8	2740.7	3090.6	3311.9	3507.5
2863.6	6399.0	10928.5	15079.3	16221.1	17863.6	18967.6	19546.9	20549.9
333.9	247.0	375.6	481.0	500.0	542.4	565.4	563.2	615.7
1390.3	1635.9	1974.2	2578.2	2880.5	3067.8	3349.7	3152.0	3683.3
682.0	1336.5	2284.4	3541.9	3983.9	4291.6	4752.4	5305.2	6162.2
13676.3	19950.1	29495.6	41243.4	43103.3	45080.7	47458.8	49134.0	52445.7
7095.5	8860.0	11107.5	15857.8	15579.9	15160.4	15235.4	15584.5	16229.7
2230.0	2352.9	2896.0	2663.3	2334.5	2534.6	2295.7	2570.0	2626.9
1358.5	1178.2	1306.4	385.3	319.8	292.4	265.1	254.1	265.5
399.9	427.0	429.1	593.1	525.7	493.5	448.2	521.3	493.2
51.6	25.9	14.4						
420.1	721.9	1146.1	1684.8	1489.1	1748.7	1582.4	1794.6	1868.2
158.6	192.9	155.4	194.4	181.7	182.0	216.1	110.3	87.6
**7.8**	**135.4**	**-7.9**	**77.4**	**39.7**	**67.4**	**23.3**	**47.4**	**27.8**

a) Data on output refer to the output of crude oil.

b) Data on imports include the petroleum consumed by the domestic airplanes and ships in refueling abroad.
Data on exports include the petroleum consumed by the oversea airplanes and ships in refueling in China.

?1994-2018 China Academic Journal Electronic Publishing House. All rights reserved. http://www.cnki.net

5-7 原油平衡表

单位: 万吨

项 目	Item	1980	1985	1990
**可供量**	**Total Energy Available for Consumption**	**9222.9**	**9516.5**	**11770.6**
生产量	Output	10594.6	12489.5	13830.6
进口量	Imports	36.6		292.3
出口量(-)	Exports (-)	1330.9	3003.0	2399.0
年初年末库存差额	Stock Changes in the Year	-77.4	30.0	46.7
**消费量**	**Total Energy Consumption**	**9205.0**	**9509.5**	**11762.2**
在消费量中:	Consumption by Sector			
1.农、林、牧、渔业	Agriculture, Forestry, Animal Husbandry and Fishery	8.0	0.8	0.2
2.工业	Industry	9112.0	9389.9	11653.8
3.建筑业	Construction	28.8	74.0	55.2
4.交通运输、仓储和邮政业	Transport, Storage and Post	50.1	44.3	52.1
5.批发、零售业和住宿、餐饮业	Wholesale, Retail Trade and Hotel, Restaurants		0.1	0.3
6.其他	Others	6.1	0.4	0.6
7.生活消费	Residential Consumption			
在消费量中:	Consumption by Usage			
(一) 终端消费	(I) Final Consumption	499.6	350.4	402.1
#工 业	Industry	429.7	254.9	333.4
(二) 中间消费	(II) Intermediate Consumption			
(用于加工转换)	(Consumed in Transformation)	8443.0	8929.7	11106.9
发 电	Power Generation	574.0	279.5	124.6
供 热	Heating		61.3	21.1
炼油	Petroleum Refineries	7869.0	8588.9	10961.2
(三) 油田原油损失量	(III) Losses in Oil Field for Crude Oil	262.4	229.4	253.2
**平衡差额**	**Balance**	**17.9**	**7.0**	**8.4**

?1994-2018 China Academic Journal Electronic Publishing House. All rights reserved. http://www.cnki.net

Crude Oil Balanc Sheet

(10 000 tons)

	1995	2000	2005	2010	2011	2012	2013	2014	2015
	**14794.9**	**21383.0**	**30089.2**	**42876.6**	**43961.0**	**46684.7**	**48670.9**	**51544.6**	**54093.5**
	15004.4	16300.0	18135.3	20301.4	20287.6	20747.8	20991.9	21142.9	21455.6
	1709.0	7026.5	12681.7	23768.2	25377.9	27102.7	28174.2	30837.4	33548.3
	1822.7	1030.6	806.7	303.0	251.4	243.2	161.7	60.0	286.6
	-95.8	-912.9	78.8	-890.0	-1453.0	-922.6	-333.4	-375.7	-623.8
	**14886.4**	**21232.0**	**30088.9**	**42874.6**	**43965.8**	**46678.9**	**48652.2**	**51547.0**	**54088.3**
	10.1								
	14716.3	21052.1	29962.1	42716.6	43860.4	46559.5	48503.4	51502.1	54052.4
	2.7	3.3							
	156.8	175.1	126.9	158.0	105.4	119.4	148.7	44.9	35.9
	0.5	0.2							
	1390.3	1.4							
	309.9	636.8	850.4	806.1	522.7	555.5	629.4	855.9	782.7
	274.7	612.3	850.4	806.1	522.7	555.5	629.4	855.9	782.7
	14419.4	20404.3	29084.8	41876.4	43266.1	45945.7	47810.5	50583.3	53218.4
	61.6	85.0	41.3	3.7	11.3	10.9	10.4	8.9	12.5
	4.4	14.0	3.0	3.3	4.6	1.4	3.4	7.0	6.7
	14353.4	20305.3	29040.5	41869.4	43250.2	45933.5	47796.7	50567.4	53199.2
	157.1	190.9	153.8	192.0	177.0	177.7	212.2	107.8	87.2
	**-91.5**	**151.0**	**0.2**	**2.1**	**-4.8**	**5.8**	**18.7**	**-2.3**	**5.2**

?1994-2018 China Academic Journal Electronic Publishing House. All rights reserved. http://www.cnki.net

5-8 燃料油平衡表

单位: 万吨

项目	Item	1980	1985	1990
**可供量**	**Total Energy Available for Consumption**	**3096.1**	**2848.0**	**3320.7**
生产量	Output	3142.0	2835.8	3267.9
进口量	Imports	39.0	70.0	167.3
出口量(-)	Exports (-)	45.4	64.9	97.2
年初年末库存差额	Stock Changes in the Year	-39.5	7.1	-17.3
**消费量**	**Total Energy Consumption**	**3073.7**	**2837.4**	**3367.8**
在消费量中:	Consumption by Sector			
1.农、林、牧、渔业	Agriculture, Forestry, Animal Husbandry and Fishery	2.3	3.1	2.9
2.工业	Industry	2937.4	2662.2	3091.7
3.建筑业	Construction	15.0	18.9	47.3
4.交通运输、仓储和邮政业	Transport, Storage and Post	109.0	144.1	208.2
5.批发、零售业和住宿、餐饮业	Wholesale, Retail Trade and Hotel, Restaurants	2.9	3.1	1.6
6.其他	Others	7.1	6.0	16.1
7.生活消费	Residential Consumption			
在消费量中:	Consumption by Usage			
(一) 终端消费	(I) Final Consumption	1617.9	1538.8	2042.6
#工 业	Industry	1481.6	1363.5	1766.5
(二) 中间消费	(II) Intermediate Consumption			
(用于加工转换)	(Consumed in Transformation)	1455.8	1296.1	1325.2
发 电	Power Generation	1419.1	1042.3	977.3
供 热	Heating		219.3	308.3
炼油再投入量	Petroleum Production			
制 气	Gas Production	36.7	34.5	39.6
(三)损失量	(III) Other Losses		2.5	
**平衡差额**	**Balance**	**22.4**	**10.6**	**-47.1**

?1994-2018 China Academic Journal Electronic Publishing House. All rights reserved. http://www.cnki.net

Fuel Oil Balance Sheet

(10 000 ton)

1995	2000	2005	2010	2011	2012	2013	2014	2015
**3717.3**	**3836.7**	**4237.3**	**3765.4**	**3667.6**	**3691.0**	**3926.1**	**4401.9**	**4632.8**
2960.8	2053.7	1767.4	2487.0	2281.8	2253.2	2775.9	3541.7	3963.0
859.1	1704.3	2883.9	2695.2	3097.4	3102.4	2734.3	2146.3	2068.2
68.6	57.9	427.6	1419.7	1685.6	1601.5	1493.7	1279.9	1402.1
-34.0	136.6	13.5	3.0	-26.0	-63.1	-90.4	-6.2	3.7
**3693.7**	**3872.8**	**4244.2**	**3758.0**	**3662.8**	**3683.3**	**3954.0**	**4400.5**	**4662.0**
8.4	0.4	0.7	1.1	1.3	2.0	2.0	1.3	0.9
3406.2	2975.1	2986.9	2377.3	2260.2	2241.7	2421.1	2835.7	3133.0
14.2	16.7	14.2	30.8	30.6	27.1	59.5	44.6	53.5
227.5	850.0	1201.0	1326.7	1345.2	1383.9	1429.0	1486.4	1439.5
6.6	11.6	27.5	8.6	9.3	8.7	19.1	17.4	19.0
30.8	19.0	13.9	13.5	16.2	19.9	23.4	15.1	16.1
2262.8	2741.4	2989.9	2403.2	2230.2	2072.8	2101.8	2103.0	2123.7
1975.3	1843.7	1732.6	1022.5	827.6	631.2	568.9	538.2	594.8
1430.9	1131.3	1254.3	1354.8	1432.6	1610.5	1852.1	2297.5	2538.3
1071.5	814.2	1068.7	123.9	60.9	43.1	47.3	35.6	31.5
307.8	291.2	171.1	201.3	183.8	187.3	167.2	164.9	165.1
			1029.6	1188.0	1380.0	1637.7	2097.0	2341.6
51.6	25.9	14.4						
**23.6**	**-36.1**	**-6.9**	**7.4**	**4.8**	**7.7**	**-27.8**	**1.4**	**-29.2**

?1994-2018 China Academic Journal Electronic Publishing House. All rights reserved. http://www.cnki.net

5-9 汽油平衡表

单位：万吨

项目	Item	1980	1985	1990
**可供量**	**Total Energy Available for Consumption**	**999.4**	**1399.6**	**1884.1**
生产量	Output	1079.0	1471.9	2173.4
进口量	Imports		0.3	16.9
出口量(-)	Exports (-)	117.8	129.9	233.8
年初年末库存差额	Stock Changes in the Year	38.2	57.3	-72.4
**消费量**	**Total Energy Consumption**	**998.6**	**1396.3**	**1899.5**
在消费量中:	Consumption by Sector			
1.农、林、牧、渔业	Agriculture, Forestry, Animal Husbandry and Fishery	53.3	122.3	145.9
2.工业	Industry	273.2	451.3	589.3
3.建筑业	Construction	54.1	73.0	89.5
4.交通运输、仓储和邮政业	Transport, Storage and Post	404.9	477.4	620.1
5.批发、零售业和住宿、餐饮业	Wholesale, Retail Trade and Hotel, Restaurants	19.4	23.4	46.0
6.其他	Others	193.7	238.3	390.7
7.生活消费	Residential Consumption		10.6	18.0
**平衡差额**	**Balance**	**0.8**	**3.3**	**-15.4**

5-10 煤油平衡表

单位：万吨

项目	Item	1980	1985	1990
**可供量**	**Total Energy Available for Consumption**	**359.0**	**383.2**	**350.9**
生产量	Output	398.5	405.3	392.5
进口量	Imports		15.2	26.1
出口量(-)	Exports (-)	46.8	46.0	55.5
年初年末库存差额	Stock Changes in the Year	2.3	8.7	-12.2
**消费量**	**Total Energy Consumption**	**365.9**	**385.5**	**350.9**
在消费量中:	Consumption by Sector			
1.农、林、牧、渔业	Agriculture, Forestry, Animal Husbandry and Fishery	2.3	3.3	3.1
2.工业	Industry	15.7	20.1	20.6
3.建筑业	Construction	0.8	1.3	1.3
4.交通运输、仓储和邮政业	Transport, Storage and Post	31.4	56.2	93.4
5.批发、零售业和住宿、餐饮业	Wholesale, Retail Trade and Hotel, Restaurants	0.2	0.1	0.6
6.其他	Others	216.7	182.9	127.3
7.生活消费	Residential Consumption	98.8	121.6	104.6
**平衡差额**	**Balance**	**-6.9**	**-2.3**	

?1994-2018 China Academic Journal Electronic Publishing House. All rights reserved.   http://www.cnki.net

Gasoline Balance Sheet

(10 000 tons)

1995	2000	2005	2010	2011	2012	2013	2014	2015
**2902.0**	**3504.5**	**4855.3**	**6964.3**	**7597.9**	**8164.5**	**9369.5**	**9770.7**	**11385.0**
3051.6	4134.7	5433.6	7410.5	8117.9	8976.1	9834.0	11029.9	12103.6
15.9				2.9	0.5		3.4	17.0
193.1	467.7	559.7	517.0	406.0	291.7	468.7	507.5	589.3
27.6	-162.5	-18.6	70.8	-117.0	-520.3	4.2	-755.0	-146.3
**2909.6**	**3504.6**	**4854.9**	**6956.2**	**7595.9**	**8165.9**	**9366.4**	**9776.4**	**11368.5**
179.7	89.2	159.6	169.1	186.0	192.9	198.7	216.6	231.3
812.4	682.0	441.7	689.5	604.8	581.1	523.4	489.0	477.1
103.6	115.6	172.1	274.7	282.8	286.9	326.5	331.0	408.6
982.3	1527.8	2430.1	3274.9	3573.5	3778.0	4381.8	4665.0	5306.6
197.2	69.8	129.4	168.2	177.1	200.1	220.9	217.8	243.3
570.7	792.7	998.2	1166.2	1313.2	1460.5	1818.7	1738.1	2108.5
63.7	227.6	523.8	1213.7	1458.6	1666.5	1896.4	2118.8	2593.1
**-7.6**	**-0.1**	**0.4**	**8.1**	**1.9**	**-1.4**	**3.2**	**-5.7**	**16.6**

Kerosene Balance Sheet

(10 000 ton)

1995	2000	2005	2010	2011	2012	2013	2014	2015
**486.4**	**880.9**	**1070.0**	**1767.6**	**1821.7**	**1959.1**	**2189.1**	**2336.4**	**2732.6**
445.8	872.3	1006.5	1924.4	1922.4	2164.0	2523.9	3081.0	3658.6
115.7	322.5	476.1	726.1	875.1	877.3	945.2	721.0	716.4
62.4	256.3	447.6	870.5	966.8	1085.9	1280.6	1455.8	1626.6
-12.7	-57.6	35.0	-12.3	-9.0	3.7	0.6	-9.7	-15.8
**512.1**	**871.6**	**1076.8**	**1765.2**	**1816.7**	**1956.6**	**2164.1**	**2335.4**	**2663.7**
3.6	1.5	1.6	0.9	1.5	1.2	1.2	0.8	1.1
44.9	84.0	57.5	40.2	34.2	32.0	27.4	17.4	21.2
3.5	4.0		8.8	10.8	7.9	11.4	10.4	12.5
250.0	535.9	952.4	1601.1	1646.4	1787.1	1998.2	2216.0	2504.9
8.5	14.0	3.7	35.0	32.2	28.6	13.4	11.3	11.7
137.3	160.1	36.2	58.7	68.2	74.2	84.6	50.7	83.3
64.3	72.2	25.5	20.5	23.5	25.6	27.9	28.9	29.1
**-25.7**	**9.3**	**-6.8**	**2.4**	**5.0**	**2.4**	**25.0**	**1.0**	**68.9**

?1994-2018 China Academic Journal Electronic Publishing House. All rights reserved. http://www.cnki.net

5-11 柴油平衡表

单位: 万吨

项 目	Item	1980	1985	1990
**可供量**	**Total Energy Available for Consumption**	**1663.2**	**1944.1**	**2689.4**
生产量	Output	1827.8	2023.2	2609.0
进口量	Imports	2.1	4.5	233.8
出口量(-)	Exports (-)	166.5	225.6	169.8
年初年末库存差额	Stock Changes in the Year	-0.2	142.0	16.4
**消费量**	**Total Energy Consumption**	**1663.2**	**1939.4**	**2691.7**
在消费量中:	Consumption by Sector			
1.农、林、牧、渔业	Agriculture, Forestry, Animal Husbandry and Fishery	749.0	629.2	881.5
2.工业	Industry	457.4	644.1	728.1
3.建筑业	Construction	76.5	125.0	133.0
4.交通运输、仓储和邮政业	Transport, Storage and Post	316.1	454.4	709.4
5.批发、零售业和住宿、餐饮业	Wholesale, Retail Trade and Hotel, Restaurants	6.5	10.9	22.5
6.其他	Others	57.7	74.0	217.0
7.生活消费	Residential Consumption			
在消费量中:	Consumption by Usage			
(一) 终端消费	(I) Final Consumption	1590.9	1827.4	2564.8
#工 业	Industry	385.1	532.1	601.2
(二) 中间消费	(II) Intermediate Consumption			
(用于加工转换)	(Consumed in Transformation)	72.3	108.6	126.9
发 电	Power Generation	72.3	103.6	124.5
供 热	Heating		5.0	2.4
(三) 损失量	(III) Other Losses		3.4	
**平衡差额**	**Balance**		**4.7**	**-2.3**

5-12 液化石油气平衡表

单位: 万吨

项 目	Item	1980	1985	1990
**可供量**	**Total Energy Available for Consumption**	**122.5**	**157.3**	**258.5**
生产量	Output	122.5	159.7	261.6
进口量	Imports			
出口量(-)	Exports (-)		1.9	1.1
年初年末库存差额	Stock Changes in the Year		-0.5	-2.0
**消费量**	**Total Energy Consumption**	**119.6**	**155.7**	**254.2**
在消费量中:	Consumption by Sector			
1.农、林、牧、渔业	Agriculture, Forestry, Animal Husbandry and Fishery			
2.工业	Industry	76.1	59.9	82.0
3.建筑业	Construction			1.0
4.交通运输、仓储和邮政业	Transport, Storage and Post			
5.批发、零售业和住宿、餐饮业	Wholesale, Retail Trade and Hotel, Restaurants		0.5	6.6
6.其他	Others	0.4	4.5	6.1
7.生活消费	Residential Consumption	43.1	90.8	158.5
**平衡差额**	**Balance**	**2.9**	**1.6**	**4.3**

?1994-2018 China Academic Journal Electronic Publishing House. All rights reserved. http://www.cnki.net

Diesel Oil Balance Sheet

(10 000 ton)

1995	2000	2005	2010	2011	2012	2013	2014	2015
**4404.2**	**6806.5**	**10972.6**	**14701.9**	**15626.2**	**16966.9**	**17105.9**	**17172.9**	**17353.6**
3972.6	7079.6	11090.2	14924.4	15689.7	17063.8	17275.7	17635.3	18007.9
645.3	51.9	61.0	190.2	243.3	99.9	35.3	55.0	71.5
169.5	77.5	170.9	490.2	228.8	205.7	294.4	423.9	731.3
-44.2	-247.6	-7.7	77.5	-78.0	8.9	89.4	-93.7	5.5
**4321.4**	**6806.2**	**10974.9**	**14699.0**	**15635.1**	**16966.0**	**17150.6**	**17165.3**	**17360.3**
1001.4	697.1	1286.3	1206.7	1271.9	1335.5	1441.5	1492.0	1492.9
1189.9	1696.5	1710.0	2090.0	1824.3	1747.7	1675.9	1595.3	1516.4
118.2	205.9	386.6	490.2	518.6	518.0	557.0	552.0	555.7
1246.6	3293.8	6169.4	8657.6	9485.2	10727.0	10920.5	11042.8	11162.8
103.6	95.9	116.0	196.6	212.3	229.0	233.5	230.1	257.7
645.7	638.7	900.1	1287.2	1428.1	1444.7	1339.8	1268.7	1384.2
16.1	178.4	406.4	770.7	894.7	964.1	982.5	984.4	990.7
4070.0	6578.6	10889.4	14655.2	15593.5	16900.7	17106.8	17127.0	17280.4
938.5	1468.8	1624.5	2046.2	1782.7	1682.3	1632.0	1557.0	1436.5
251.4	227.7	85.5	43.8	41.6	65.4	43.9	38.3	79.9
204.9	227.7	81.9	40.1	39.2	35.6	35.6	25.8	22.4
46.6		3.6	3.8	2.4	2.4	2.6	4.7	6.2
**82.7**	**0.3**	**-2.4**	**2.9**	**-8.9**	**0.9**	**-44.7**	**7.6**	**-6.7**

LPG Balance Sheet

(10 000 tons)

1995	2000	2005	2010	2011	2012	2013	2014	2015
**774.3**	**1396.2**	**2052.2**	**2323.8**	**2474.2**	**2496.0**	**2836.1**	**3292.7**	**4008.2**
540.8	916.6	1432.7	2092.3	2240.8	2268.7	2513.3	2705.8	2934.4
232.6	481.7	617.0	327.0	349.6	358.5	451.7	739.4	1244.0
7.1	1.6	2.7	93.0	119.1	128.2	126.9	144.4	144.2
8.0	-0.6	5.2	-2.5	3.0	-3.0	-2.0	-8.0	-26.0
**750.6**	**1389.7**	**2046.5**	**2321.9**	**2470.2**	**2482.2**	**2823.4**	**3289.8**	**3961.2**
0.1	0.4	3.5	4.7	5.6	6.4	6.8	7.1	7.2
192.5	426.1	534.4	586.8	661.1	621.0	705.1	835.0	1113.9
0.5	8.9	6.3	7.2	7.2	6.8	14.7	16.8	15.1
0.5	16.5	48.7	61.0	65.5	68.1	89.4	91.8	100.3
17.4	55.5	99.0	72.6	69.0	76.0	78.5	86.6	84.0
5.7	24.0	25.8	52.6	54.8	68.5	83.4	79.4	91.4
534.0	858.3	1328.7	1537.0	1607.2	1635.4	1845.6	2173.1	2549.3
**23.7**	**6.5**	**5.7**	**1.9**	**4.0**	**13.8**	**12.7**	**2.9**	**47.0**

?1994-2018 China Academic Journal Electronic Publishing House. All rights reserved. http://www.cnki.net

5-13 天然气平衡表

单位: 亿立方米

项目	Item	1980	1985	1990
**可供量**	**Total Energy Available for Consumption**	**142.7**	**129.3**	**153.0**
生产量	Output	142.7	129.3	153.0
进口量	Imports			
出口量(-)	Exports (-)			
年初年末库存差额	Stock Changes in the Year			
**消费量**	**Total Energy Consumption**	**140.6**	**129.3**	**152.5**
在消费量中:	Consumption by Sector			
1.农、林、牧、渔业	Agriculture, Forestry, Animal Husbandry and Fishery			
2.工业	Industry	131.4	109.6	120.2
3.建筑业	Construction	6.0	14.1	10.6
4.交通运输、仓储和邮政业	Transport, Storage and Post	0.7	0.8	1.9
5.批发、零售业和住宿、餐饮业	Wholesale, Retail Trade and Hotel, Restaurants			
6.其他	Others	0.5	0.5	1.2
7.生活消费	Residential Consumption	2.0	4.3	18.6
**平衡差额**	**Balance**	**2.1**		**0.5**

注: 从2010年起包括液化天然气数据。

5-14 电力平衡表

单位: 亿千瓦小时

项目	Item	1980	1985	1990
**可供量**	**Total Energy Available for Consumption**	**3006.3**	**4117.6**	**6230.4**
生产量	Output	3006.3	4106.9	6212.0
水电	Hydropower	582.1	923.7	1267.2
火电	Thermal Power	2424.2	3183.2	4944.8
核电	Nuclear Power			
风电	Wind Power			
进口量	Imports		11.1	19.3
出口量(-)	Exports (-)		0.4	0.9
**消费量**	**Total Energy Consumption**	**3006.3**	**4117.6**	**6230.4**
在消费量中:	Consumption by Sector			
1.农、林、牧、渔业	Agriculture, Forestry, Animal Husbandry and Fishery	270	317.4	426.8
2.工业	Industry	2471.9	3283.4	4873.3
3.建筑业	Construction	47.1	71.2	65.0
4.交通运输、仓储和邮政业	Transport, Storage and Post	26.5	63.4	105.9
5.批发、零售业和住宿、餐饮业	Wholesale, Retail Trade and Hotel, Restaurants	16.8	38.0	76.2
6.其他	Others	68.8	121.7	202.4
7.生活消费	Residential Consumption	105.2	222.5	480.8
在消费量中:	Consumption by Usage			
(一) 终端消费	(I) Final Consumption	2763.4	3813.3	5795.8
#工业	Industry	2229.0	2979.1	4438.7
(二) 输配电损失量	(II) Losses in Transmission	242.9	304.3	434.6

?1994-2018 China Academic Journal Electronic Publishing House. All rights reserved. http://www.cnki.net

Natural Gas Balance Sheet

(100 million cu.m)

1995	2000	2005	2010	2011	2012	2013	2014	2015
**179.5**	**240.6**	**463.5**	**1082.3**	**1333.0**	**1497.8**	**1706.6**	**1866.8**	**1925.0**
179.5	272.0	493.2	957.9	1053.4	1106.1	1208.6	1301.6	1346.1
			164.7	311.5	420.6	525.4	591.3	611.4
	31.4	29.7	40.3	31.9	28.9	27.5	26.1	32.5
**177.4**	**245.0**	**466.1**	**1080.2**	**1341.1**	**1497.0**	**1705.4**	**1868.9**	**1931.7**
			0.5	0.6	0.6	0.7	0.8	0.9
154.4	199.0	327.2	691.8	875.7	980.7	1129.1	1221.3	1234.5
0.3	0.8	1.5	1.2	1.3	1.3	2.0	1.9	2.2
1.6	8.8	38.0	106.7	138.3	154.5	175.8	214.4	237.6
0.6	3.4	10.8	27.2	33.6	38.7	39.3	46.6	51.3
1.2	0.6	9.1	26.0	27.1	32.9	35.6	41.3	45.4
19.4	32.3	79.4	226.9	264.4	288.3	322.9	342.6	359.8
**2.1**	**-4.4**	**-2.6**	**2.1**	**0.7**	**0.8**	**1.2**	**-2.1**	**-6.7**

a) Include the data of LNG since 2010.

Electricity Balance Sheet

(100 million kW•h)

1995	2000	2005	2010	2011	2012	2013	2014	2015
**10023.4**	**13472.7**	**24940.8**	**41936.5**	**47002.7**	**49767.7**	**54204.1**	**56381.8**	**58021.3**
10077.3	13556.0	25002.6	42071.6	47130.2	49875.5	54316.4	56495.8	58145.7
1905.8	2224.1	3970.2	7221.7	6989.5	8721.1	9202.9	10643.4	11302.7
8043.2	11141.9	20473.4	33319.3	38337.0	38928.1	42470.1	42686.5	42841.9
128.3	167.4	530.9	738.8	863.5	973.9	1116.1	1325.4	1707.9
			446.2	703.3	959.8	1412.0	1560.8	1857.7
6.4	15.5	50.1	55.5	65.6	68.7	74.4	67.5	62.1
60.3	98.8	111.9	190.6	193.1	176.5	186.7	181.6	186.5
**10023.4**	**13472.4**	**24940.3**	**41934.5**	**47000.9**	**49762.6**	**54203.4**	**56383.7**	**58020.0**
582.4	533.0	776.3	976.5	1012.9	1012.6	1026.9	1013.4	1039.8
7659.8	10004.6	18521.7	30871.8	34691.6	36232.2	39236.9	40802.7	41550.0
159.6	159.8	233.9	483.2	571.8	608.4	675.1	721.7	698.7
182.3	281.2	430.3	734.5	848.4	915.4	1000.9	1059.2	1125.6
199.5	418.7	752.3	1292.0	1503.1	1691.5	1876.9	1995.6	2122.0
234.2	623.2	1340.9	2451.8	2753.1	3083.6	3397.6	3615.0	3918.6
1005.6	1452.0	2884.8	5124.6	5620.1	6219.0	6989.2	7176.1	7565.2
9278.9	12535.7	23233.8	39366.3	44300.2	46866.5	51062.7	53283.8	55032.1
6915.3	9067.9	16815.2	28303.5	31990.9	33336.1	36096.2	37702.8	38562.1
744.5	936.7	1706.5	2568.2	2700.7	2896.2	3140.7	3099.9	2987.9

?1994-2018 China Academic Journal Electronic Publishing House. All rights reserved. http://www.cnki.net

5-1 全国能源平衡表(实物量) -2020

项 目	Item	煤合计 (万吨) Coal Total (10^4 tons)	原煤 (万吨) Raw Coal (10^4 tons)
**一.可供本地区消费的能源量**	**Total Primary Energy Supply**	**414518.97**	**415805.42**
1.一次能源生产量	Indigenous Production	390157.71	390157.71
水电	Hydro Power		
核电	Nuclear Power		
风电	Wind Power		
2.进口量	Import	30361.09	30361.09
3.境内飞机和轮船在境外的加油量	Domestic Airplanes&Ships Refueling Abroad		
4.出口量(-)	Export (-)	319.46	316.78
5.境外飞机和轮船在境内的加油量(-)	Oversea Airplanes&Ships Refueling Domestically (-)		
6.库存增(-)、减(+)量	Stock Change	-5680.37	-4396.61
**二.加工转换投入(-)产出(+)量**	**Input(-) & Output(+) of Transformation**	**-332434.09**	**-349916.36**
1.火力发电	Thermal Power	-211634.87	-206845.78
2.供热	Heating Supply	-36933.28	-35434.18
3.煤炭洗选	Coal Washing	-11301.20	-90163.69
4.炼焦	Coking	-65967.64	-10960.01
5.炼油及煤制油	Petroleum Refining and Coal-to-liquids	-3047.29	-2914.28
#油品再投入量(-)	Petroleum Products Input (-)		
6.制气	Gas Works	-3308.95	-3150.59
#再投入量(-)	Input (-)		
7.天然气液化	Natural Gas Liquefaction		
8.煤制品加工	Briquettes	-240.86	-447.82
9.回收能	Recovery of Energy		
**三.损失量**	**Loss**		
**四.终端消费量**	**Total Final Consumption**	**72425.57**	**57206.52**
1.农、林、牧、渔业	Agriculture, Forestry, Animal Husbandry and Fishery	2253.90	2172.28
2.工业	Industry	58457.16	44258.69
#用作原料、材料	Non-Energy Use	13945.34	11477.50
3.建筑业	Construction	638.85	597.02
4.交通运输、仓储和邮政业	Transport, Storage and Post	241.01	224.31
5.批发和零售业、住宿和餐饮业	Wholesale and Retail Trades, Hotels and Catering Services	1981.18	1926.62
6.其他	Others	2570.80	2488.06
7.居民生活	Residential	6282.66	5539.54
城镇	Urban	611.84	482.44
乡村	Rural	5670.81	5057.10
**五.平衡差额**	**Statistical Difference**	**9659.31**	**8682.54**
**六.消费量合计**	**Total Energy Consumption**	**404859.66**	**407122.88**

(C)1994-2024 China Academic Journal Electronic Publishing House. All rights reserved. http://www.cnki.net

Energy Balance of China (Physical Quantity) -2020

洗精煤 (万吨) Cleaned Coal (10^4 tons)	其他洗煤 (万吨) Other Washed Coal (10^4 tons)	煤制品 (万吨) Briquettes (10^4 tons)	煤矸石 (万吨) Gangue (10^4 tons)	焦炭 (万吨) Coke (10^4 tons)	焦炉煤气 (亿立方米) Coke Oven Gas (10^8 cu.m)	高炉煤气 (亿立方米) Blast Furnace Gas (10^8 cu.m)	转炉煤气 (亿立方米) Converter Gas (10^8 cu.m)	其他煤气 (亿立方米) Other Gas (10^8 cu.m)
**-747.63**	**-531.00**	**-7.81**		**123.79**				
				297.91				
		2.68		349.21				
-747.63	-531.00	-5.13		175.09				
**1117.80**	**15008.33**	**1356.13**	**-60.08**	**46640.33**	**711.53**	**8481.93**	**661.85**	**145.40**
	-4789.09		-2087.61	-250.03	-217.85	-2932.52	-360.91	-3.48
	-1499.10		-762.82	-280.28	-57.17	-500.05	-49.18	-1.60
56125.43	22737.06		2790.35					
-55007.63				47188.23	1032.53			
	-133.01							
	-158.35							150.47
				-17.59	-45.99			
	-1149.18	1356.13						
						11914.49	1071.94	
	**13932.63**	**1286.41**		**47762.51**	**727.21**	**8638.81**	**651.37**	**149.80**
	81.62			23.24				
	13300.72	897.76		47724.27	718.58	8638.81	651.37	97.63
	2302.54	165.30		1818.29	105.48			
	41.83			3.57				
	16.70							
	37.37	17.18		0.02	0.68			9.54
	76.57	6.17			0.88			
	377.83	365.29		11.41	7.07			42.63
	52.23	77.18		2.82	5.12			42.41
	325.60	288.11		8.59	1.95			0.22
**370.17**	**544.69**	**61.91**	**-60.08**	**-998.40**	**-15.67**	**-156.88**	**10.48**	**-4.40**
**55007.63**	**21661.36**	**1286.41**	**2850.43**	**48310.41**	**1048.21**	**12071.38**	**1061.46**	**154.88**

(C)1994-2024 China Academic Journal Electronic Publishing House. All rights reserved. http://www.cnki.net

5-1 续表 1

项　目	Item	其他焦化产品 (万吨) Other Coking Products (10^4 tons)	油品合计 (万吨) Petroleum Products Total (10^4 tons)
**一.可供本地区消费的能源量**	**Total Primary Energy Supply**		**67553.72**
1.一次能源生产量	Indigenous Production		19476.86
水电	Hydro Power		
核电	Nuclear Power		
风电	Wind Power		
2.进口量	Import		60497.54
3.境内飞机和轮船在境外的加油量	Domestic Airplanes&Ships Refueling Abroad		774.14
4.出口量(−)	Export (-)		6738.13
5.境外飞机和轮船在境内的加油量(−)	Oversea Airplanes&Ships Refueling Domestically (-)		812.90
6.库存增(−)、减(+)量	Stock Change		-5643.79
**二.加工转换投入(−)产出(+)量**	**Input(-) & Output(+) of Transformation**	**933.70**	**-3265.64**
1.火力发电	Thermal Power		-321.54
2.供热	Heating Supply		-677.70
3.煤炭洗选	Coal Washing		
4.炼焦	Coking	1350.21	-4.60
5.炼油及煤制油	Petroleum Refining and Coal-to-liquids	-412.06	12495.07
#油品再投入量(−)	Petroleum Products Input (-)		-14728.00
6.制气	Gas Works	2.17	-28.86
#再投入量(−)	Input (-)	-6.63	
7.天然气液化	Natural Gas Liquefaction		
8.煤制品加工	Briquettes		
9.回收能	Recovery of Energy		
**三.损失量**	**Loss**		**18.26**
**四.终端消费量**	**Total Final Consumption**	**1001.96**	**62085.22**
1.农、林、牧、渔业	Agriculture, Forestry, Animal Husbandry and Fishery		1773.10
2.工业	Industry	1001.96	24428.39
#用作原料、材料	Non-Energy Use	369.98	14394.21
3.建筑业	Construction		4180.29
4.交通运输、仓储和邮政业	Transport, Storage and Post		20480.16
5.批发和零售业、住宿和餐饮业	Wholesale and Retail Trades, Hotels and Catering Services		583.27
6.其他	Others		3469.89
7.居民生活	Residential		7170.11
城镇	Urban		4884.76
乡村	Rural		2285.35
**五.平衡差额**	**Statistical Difference**	**-68.26**	**2184.60**
**六.消费量合计**	**Total Energy Consumption**	**1420.65**	**65369.11**

(C)1994-2024 China Academic Journal Electronic Publishing House. All rights reserved. http://www.cnki.net

Continued 1

原油 (万吨) Crude Oil (10^4 tons)	汽油 (万吨) Gasoline (10^4 tons)	煤油 (万吨) Kerosene (10^4 tons)	柴油 (万吨) Diesel Oil (10^4 tons)	燃料油 (万吨) Fuel Oil (10^4 tons)	石脑油 (万吨) Naphtha (10^4 tons)	润滑油 (万吨) Lubricants (10^4 tons)	石蜡 (万吨) Paraffin Waxes (10^4 tons)	溶剂油 (万吨) White Spirit (10^4 tons)
**69969.65**	**-1427.66**	**-717.04**	**-2070.17**	**-436.41**	**772.46**	**16.30**	**-49.71**	**5.10**
19476.86								
54200.67	48.05	265.73	119.10	1253.47	788.70	28.95	11.34	3.69
		248.32	18.37	507.45				
163.81	1600.00	997.48	1975.95	1583.31		13.43	66.00	0.31
		228.78	35.21	548.91				
-3544.07	124.29	-4.82	-196.48	-65.10	-16.24	0.79	4.94	1.72
**-69040.48**	**14233.10**	**4129.42**	**16383.32**	**2749.06**	**5621.91**	**258.93**	**239.24**	**37.64**
-16.55			-27.58	-29.43				
			-2.91	-231.38				
-69023.93	14256.69	4129.42	16529.85	5780.70	7190.72	263.38	249.42	39.97
	-23.59		-116.04	-2770.83	-1568.81	-4.45	-10.18	-2.32
**17.63**								
**419.03**	**12743.57**	**3352.10**	**14136.16**	**2332.96**	**6178.69**	**267.78**	**198.21**	**44.16**
	257.35	11.00	1497.15	1.13				
419.03	160.38	9.39	879.58	230.69	6178.69	267.78	198.21	44.16
	2.47	1.82	20.75	37.99	5750.38	245.96	194.33	41.56
	508.39	10.81	503.92	40.75				
	5573.57	3110.76	9531.98	2042.01				
	273.23	14.76	197.61	11.80				
	2253.09	182.96	946.15	6.59				
	3717.56	12.42	579.78					
	2510.98	0.36	265.29					
	1206.59	12.06	314.49					
**492.50**	**61.87**	**60.28**	**176.98**	**-20.31**	**215.68**	**7.46**	**-8.68**	**-1.41**
**69477.14**	**12767.16**	**3352.10**	**14282.70**	**5364.60**	**7747.49**	**272.23**	**208.39**	**46.48**

(C)1994-2024 China Academic Journal Electronic Publishing House. All rights reserved. http://www.cnki.net

5-1 续表 2

项 目	Item	石油沥青(万吨) Bitumen Asphalt (10^4 tons)	石油焦(万吨) Petroleum Coke (10^4 tons)
**一.可供本地区消费的能源量**	**Total Primary Energy Supply**	**228.14**	**847.55**
1.一次能源生产量	Indigenous Production		
水电	Hydro Power		
核电	Nuclear Power		
风电	Wind Power		
2.进口量	Import	475.92	1027.66
3.境内飞机和轮船在境外的加油量	Domestic Airplanes&Ships Refueling Abroad		
4.出口量(-)	Export (-)	56.74	178.36
5.境外飞机和轮船在境内的加油量(-)	Oversea Airplanes&Ships Refueling Domestically (-)		
6.库存增(-)、减(+)量	Stock Change	-191.04	-1.76
**二.加工转换投入(-)产出(+)量**	**Input(-) & Output(+) of Transformation**	**3321.53**	**2454.29**
1.火力发电	Thermal Power		-189.88
2.供热	Heating Supply		-256.55
3.煤炭洗选	Coal Washing		
4.炼焦	Coking		-4.60
5.炼油及煤制油	Petroleum Refining and Coal-to-liquids	3950.47	2956.90
#油品再投入量(-)	Petroleum Products Input (-)	-628.94	-42.34
6.制气	Gas Works		-9.25
#再投入量(-)	Input (-)		
7.天然气液化	Natural Gas Liquefaction		
8.煤制品加工	Briquettes		
9.回收能	Recovery of Energy		
**三.损失量**	**Loss**		
**四.终端消费量**	**Total Final Consumption**	**3479.59**	**3163.41**
1.农、林、牧、渔业	Agriculture, Forestry, Animal Husbandry and Fishery		
2.工业	Industry	349.52	3163.41
#用作原料、材料	Non-Energy Use	304.14	2423.03
3.建筑业	Construction	3018.82	
4.交通运输、仓储和邮政业	Transport, Storage and Post	111.24	
5.批发和零售业、住宿和餐饮业	Wholesale and Retail Trades, Hotels and Catering Services		
6.其他	Others		
7.居民生活	Residential		
城镇	Urban		
乡村	Rural		
**五.平衡差额**	**Statistical Difference**	**70.08**	**138.42**
**六.消费量合计**	**Total Energy Consumption**	**4108.53**	**3666.03**

(C)1994-2024 China Academic Journal Electronic Publishing House. All rights reserved. http://www.cnki.net

Continued 2

液化石油气 (万吨) Liquefied Petroleum Gas (10^4 tons)	炼厂干气 (万吨) Refinery Gas (10^4 tons)	其他石油制品 (万吨) Other Petroleum Products (10^4 tons)	天然气 (亿立方米) Natural Gas (10^8 cu.m)	液化天然气 (万吨) Liquefied Natural Gas (10^4 tons)	热力 (万百万千焦) Heat (10^{10} kJ)	电力 (亿千瓦时) Electricity (10^8 kW•h)	其他能源 (万吨标准煤) Other Energy (10^4 tce)
**1892.07**		**-1476.56**	**2350.63**	**6663.86**		**24317.69**	**7214.43**
			1924.95			24488.12	7214.43
						13552.09	
						3662.55	
						4664.74	
2004.63		269.65	476.53	6669.73		47.51	
94.89		7.86	50.85	5.87		217.93	
-17.68		-1738.35					
**3920.55**	**1896.13**	**10529.72**	**-755.92**	**931.47**	**596841.75**	**53302.48**	**-1325.91**
-0.14	-50.19	-7.78	-411.37	-352.62	-127833.91	53302.48	-2017.84
-7.71	-165.20	-13.95	-169.66	-48.78	613906.59		-689.73
4489.00	2348.61	19333.88	-31.42				-415.24
-556.88	-221.19	-8782.42					
-3.72	-15.90		69.94				768.20
			-15.17				
			-198.25	1332.86			
					110769.08		1028.70
**0.63**			**26.13**	**1.47**	**5204.80**	**3233.48**	
**5652.07**	**1863.57**	**8253.91**	**1568.80**	**7590.07**	**591129.64**	**74386.69**	**5590.70**
6.47			1.28		111.89	1422.11	666.23
2496.33	1863.57	8167.65	614.97	7042.49	412216.71	49119.96	990.53
866.76	127.15	4377.86	87.44	162.98			105.78
11.34		86.26	2.64		1933.11	1011.10	28.55
110.61			272.25	547.58	3904.98	1750.98	1208.26
85.87			62.14		10405.72	3169.04	64.00
81.10			55.55		21207.86	6517.02	245.50
2860.35			559.97		141349.38	11396.48	2387.64
2108.13			553.67		141349.38	6157.21	238.00
752.22			6.30			5239.27	2149.64
**159.91**	**32.56**	**799.25**	**-0.22**	**3.79**	**507.31**		**297.82**
**6221.15**	**2316.04**	**17058.07**	**2236.86**	**7992.93**	**724168.35**	**77620.17**	**8713.51**

(C)1994-2024 China Academic Journal Electronic Publishing House. All rights reserved. http://www.cnki.net

5-2 全国能源平衡表(标准量) -2020

单位：万吨标准煤

项 目	Item	能源合计 (发电煤耗计算法) (Coal Equivalent Calculation)	Energy Total (电热当量计算法) (Calorific Value Calculation)
**一.可供本地区消费的能源量**	**Total Primary Energy Supply**	**507478.68**	**464901.27**
1.一次能源生产量	Indigenous Production	407295.20	364419.41
水电	Hydro Power	40383.62	16655.52
核电	Nuclear Power	10913.96	4501.27
风电	Wind Power	13900.37	5732.96
2.进口量	Import	123687.97	123604.79
3.境内飞机和轮船在境外的加油量	Domestic Airplanes&Ships Refueling Abroad	1117.09	1117.09
4.出口量(-)	Export (-)	11665.68	11284.11
5.境外飞机和轮船在境内的加油量(-)	Oversea Airplanes&Ships Refueling Domestically (-)	1172.11	1172.11
6.库存增(-)、减(+)量	Stock Change	-11783.80	-11783.80
**二.加工转换投入(-)产出(+)量**	**Input(-) & Output(+) of Transformation**	**16.87**	**-93309.47**
1.火力发电	Thermal Power		-93326.33
2.供热	Heating Supply	-7202.87	-7202.87
3.煤炭洗选	Coal Washing	-4548.04	-4548.04
4.炼焦	Coking	-3937.65	-3937.65
5.炼油及煤制油	Petroleum Refining and Coal-to-liquids	14258.47	14258.47
#油品再投入量(-)	Petroleum Products Input (-)	-20390.25	-20390.25
6.制气	Gas Works	-425.68	-425.68
#再投入量(-)	Input (-)	-481.10	-481.10
7.天然气液化	Natural Gas Liquefaction	-182.70	-182.70
8.煤制品加工	Briquettes	-110.54	-110.54
9.回收能	Recovery of Energy	23037.21	23037.21
**三.损失量**	**Loss**	**10175.19**	**4513.74**
**四.终端消费量**	**Total Final Consumption**	**488155.77**	**357913.48**
1.农、林、牧、渔业	Agriculture, Forestry, Animal Husbandry and Fishery	9262.85	6772.91
2.工业	Industry	322677.35	236674.13
#用作原料、材料	Non-Energy Use	34557.13	34557.13
3.建筑业	Construction	9320.36	7550.03
4.交通运输、仓储和邮政业	Transport, Storage and Post	41098.69	38032.92
5.批发和零售业、住宿和餐饮业	Wholesale and Retail Trades, Hotels and Catering Services	13171.11	7622.50
6.其他	Others	28245.06	16834.53
7.居民生活	Residential	64380.34	44426.45
城镇	Urban	38731.64	27951.09
乡村	Rural	25648.71	16475.36
**五.平衡差额**	**Statistical Difference**	**9164.59**	**9164.59**
**六.消费量合计**	**Total Energy Consumption**	**498314.09**	**455736.68**

(C)1994-2024 China Academic Journal Electronic Publishing House. All rights reserved. http://www.cnki.net

Energy Balance of China (Standard Quantity) -2020

(10^4 tce)

煤合计 Coal Total	原煤 Raw Coal	洗精煤 Cleaned Coal	其他洗煤 Other Washed Coal	煤制品 Briquettes	煤矸石 Gangue	焦炭 Coke	焦炉煤气 Coke Oven Gas	高炉煤气 Blast Furnace Gas	转炉煤气 Converter Gas
**289194.95**	**290159.30**	**-672.87**	**-286.74**	**-4.74**		**120.25**			
274719.77	274719.77								
18830.69	18830.69					289.39			
276.58	274.95			1.63		339.22			
-4078.93	-3116.21	-672.87	-286.74	-3.11		170.08			
**-229296.52**	**-239230.49**	**1006.02**	**8104.50**	**823.44**	**43.79**	**45306.41**	**4065.70**	**10907.76**	**1796.27**
-139530.78	-136944.67		-2586.11		-417.52	-242.87	-1244.77	-3771.22	-979.52
-22770.06	-21960.55		-809.52		-152.56	-272.27	-326.65	-643.06	-133.46
-5161.92	-67952.82	50512.89	12278.01		613.88				
-57229.50	-7722.64	-49506.87				45838.64	5899.90		
-2177.10	-2105.27		-71.82						
-2316.63	-2231.12		-85.51						
						-17.09	-262.78		
-110.54	-313.42		-620.56	823.44					
								15322.04	2909.25
**52835.06**	**44530.33**		**7523.62**	**781.11**		**46396.50**	**4155.26**	**11109.51**	**1767.82**
1727.13	1683.05		44.07			22.58			
42043.42	34315.91		7182.39	545.12		46359.35	4105.95	11109.51	1767.82
10411.24	9067.50		1243.37	100.37		1766.29	602.71		
530.50	507.91		22.59			3.47			
181.28	172.26		9.02						
1619.29	1588.67		20.18	10.43		0.02	3.88		
2031.00	1985.90		41.35	3.75			5.03		
4702.44	4276.61		204.03	221.80		11.09	40.40		
456.92	381.85		28.20	46.86		2.74	29.26		
4245.52	3894.76		175.82	174.94		8.34	11.14		
**7063.37**	**6398.49**	**333.16**	**294.13**	**37.59**	**43.79**	**-969.84**	**-89.56**	**-201.75**	**28.45**

(C)1994-2024 China Academic Journal Electronic Publishing House. All rights reserved. http://www.cnki.net

5-2 续表 1

单位：万吨标准煤

项　目	Item	其他煤气 Other Gas	其他焦化产品 Other Coking Products
**一.可供本地区消费的能源量**	**Total Primary Energy Supply**		
1.一次能源生产量	Indigenous Production		
水电	Hydro Power		
核电	Nuclear Power		
风电	Wind Power		
2.进口量	Import		
3.境内飞机和轮船在境外的加油量	Domestic Airplanes&Ships Refueling Abroad		
4.出口量(-)	Export (-)		
5.境外飞机和轮船在境内的加油量(-)	Oversea Airplanes&Ships Refueling Domestically (-)		
6.库存增(-)、减(+)量	Stock Change		
**二.加工转换投入(-)产出(+)量**	**Input(-) & Output(+) of Transformation**	**259.68**	**1077.48**
1.火力发电	Thermal Power	-6.22	
2.供热	Heating Supply	-2.85	
3.煤炭洗选	Coal Washing		
4.炼焦	Coking		1558.15
5.炼油及煤制油	Petroleum Refining and Coal-to-liquids		-475.52
#油品再投入量(-)	Petroleum Products Input (-)		
6.制气	Gas Works	268.75	2.51
#再投入量(-)	Input (-)		-7.65
7.天然气液化	Natural Gas Liquefaction		
8.煤制品加工	Briquettes		
9.回收能	Recovery of Energy		
**三.损失量**	**Loss**		
**四.终端消费量**	**Total Final Consumption**	**267.55**	**1156.26**
1.农、林、牧、渔业	Agriculture, Forestry, Animal Husbandry and Fishery		
2.工业	Industry	174.36	1156.26
#用作原料、材料	Non-Energy Use		426.95
3.建筑业	Construction		
4.交通运输、仓储和邮政业	Transport, Storage and Post		
5.批发和零售业、住宿和餐饮业	Wholesale and Retail Trades, Hotels and Catering Services	17.04	
6.其他	Others		
7.居民生活	Residential	76.14	
城镇	Urban	75.75	
乡村	Rural	0.39	
**五.平衡差额**	**Statistical Difference**	**-7.87**	**-78.77**
**六.消费量合计**	**Total Energy Consumption**		

(C)1994-2024 China Academic Journal Electronic Publishing House. All rights reserved. http://www.cnki.net

Continued 1

(10[4] tce)

油品合计 Petroleum Products Total	原油 Crude Oil	汽油 Gasoline	煤油 Kerosene	柴油 Diesel Oil	燃料油 Fuel Oil	石脑油 Naphtha	润滑油 Lubricants	石蜡 Paraffin Waxes
**96752.95**	**99958.64**	**-2100.65**	**-1055.05**	**-3016.44**	**-623.46**	**1158.68**	**23.06**	**-67.85**
27824.64	27824.64							
86599.55	77431.08	70.69	390.99	173.53	1790.70	1183.04	40.94	15.48
1117.09			365.38	26.77	724.94			
9741.26	234.02	2354.23	1467.70	2879.16	2261.91		19.00	90.07
1172.11			336.63	51.30	784.18			
-7874.95	-5063.06	182.89	-7.09	-286.28	-93.01	-24.36	1.12	6.74
**-3999.05**	**-98631.23**	**20942.58**	**6076.02**	**23872.13**	**3927.31**	**8432.87**	**366.21**	**326.52**
-394.70	-23.64			-40.19	-42.04			
-895.53				-4.24	-330.55			
-4.83								
17727.32	-98607.59	20977.29	6076.02	24085.65	8258.31	10786.07	372.50	340.41
-20390.25		-34.71		-169.09	-3958.41	-2353.21	-6.30	-13.89
-41.07								
**26.27**	**25.19**							
**89669.83**	**598.63**	**18750.89**	**4932.28**	**20597.81**	**3332.87**	**9268.03**	**378.72**	**270.52**
2589.05		378.66	16.19	2181.50	1.62			
34291.99	598.63	235.98	13.82	1281.64	329.56	9268.03	378.72	270.52
19841.31		3.64	2.68	30.23	54.27	8625.57	347.87	265.22
5645.25		748.05	15.91	734.26	58.21			
29919.72		8200.94	4577.17	13889.04	2917.22			
875.74		402.04	21.72	287.94	16.85			
5111.49		3315.20	269.20	1378.64	9.41			
11236.59		5470.02	18.27	844.79				
7695.71		3694.65	0.53	386.55				
3540.88		1775.37	17.75	458.24				
**3057.80**	**703.59**	**91.03**	**88.70**	**257.88**	**-29.02**	**323.52**	**10.55**	**-11.85**

(C)1994-2024 China Academic Journal Electronic Publishing House. All rights reserved. http://www.cnki.net

5-2 续表 2

单位: 万吨标准煤

项　目	Item	溶剂油 White Spirit	石油沥青 Bitumen Asphalt
**一.可供本地区消费的能源量**	**Total Primary Energy Supply**	**7.49**	**298.87**
1.一次能源生产量	Indigenous Production		
水电	Hydro Power		
核电	Nuclear Power		
风电	Wind Power		
2.进口量	Import	5.41	623.46
3.境内飞机和轮船在境外的加油量	Domestic Airplanes&Ships Refueling Abroad		
4.出口量(-)	Export (-)	0.45	74.33
5.境外飞机和轮船在境内的加油量(-)	Oversea Airplanes&Ships Refueling Domestically (-)		
6.库存增(-)、减(+)量	Stock Change	2.52	-250.26
**二.加工转换投入(-)产出(+)量**	**Input(-) & Output(+) of Transformation**	**55.23**	**4351.20**
1.火力发电	Thermal Power		
2.供热	Heating Supply		
3.煤炭洗选	Coal Washing		
4.炼焦	Coking		
5.炼油及煤制油	Petroleum Refining and Coal-to-liquids	58.64	5175.12
#油品再投入量(-)	Petroleum Products Input (-)	-3.41	-823.91
6.制气	Gas Works		
#再投入量(-)	Input (-)		
7.天然气液化	Natural Gas Liquefaction		
8.煤制品加工	Briquettes		
9.回收能	Recovery of Energy		
**三.损失量**	**Loss**		
**四.终端消费量**	**Total Final Consumption**	**64.79**	**4558.26**
1.农、林、牧、渔业	Agriculture, Forestry, Animal Husbandry and Fishery		
2.工业	Industry	64.79	457.88
#用作原料、材料	Non-Energy Use	60.97	398.42
3.建筑业	Construction		3954.66
4.交通运输、仓储和邮政业	Transport, Storage and Post		145.72
5.批发和零售业、住宿和餐饮业	Wholesale and Retail Trades, Hotels and Catering Services		
6.其他	Others		
7.居民生活	Residential		
城镇	Urban		
乡村	Rural		
**五.平衡差额**	**Statistical Difference**	**-2.07**	**91.81**
**六.消费量合计**	**Total Energy Consumption**		

(C)1994-2024 China Academic Journal Electronic Publishing House. All rights reserved. http://www.cnki.net

Continued 2

(10⁴ tce)

石油焦 Petroleum Coke	液化石油气 Liquefied Petroleum Gas	炼厂干气 Refinery Gas	其他石油制品 Other Petroleum Products	天然气 Natural Gas	液化天然气 Liquefied Natural Gas	热力 Heat	电力 Electricity	其他能源 Other Energy
**889.92**	**3243.57**		**-1963.83**	**29996.88**	**11735.36**		**29886.45**	**7214.43**
				24564.68			30095.89	7214.43
							16655.52	
							4501.27	
							5732.96	
1079.05	3436.54		358.63	6081.08	11745.69		58.39	
187.27	162.66		10.46	648.88	10.33		267.84	
-1.85	-30.31		-2312.01					
**2577.00**	**6720.99**	**2979.58**	**14004.53**	**-9646.49**	**1640.36**	**20352.30**	**65508.75**	**-1325.91**
-199.37	-0.24	-78.86	-10.35	-5249.55	-620.97	-4359.14	65508.75	-2017.84
-269.38	-13.21	-259.59	-18.55	-2165.00	-85.90	20934.21		-689.73
-4.83								
3104.75	7695.49	3690.60	25714.06	-401.00				-415.24
-44.45	-954.67	-347.58	-11680.62					
-9.71	-6.38	-24.98		892.58				768.20
				-193.58				
				-2529.93	2347.23			
						3777.23		1028.70
	**1.08**			**333.45**	**2.59**	**177.48**	**3973.95**	
**3321.58**	**9689.34**	**2928.41**	**10977.70**	**20019.77**	**13366.45**	**20157.52**	**91421.24**	**5590.70**
	11.09			16.34		3.82	1747.77	666.23
3321.58	4279.46	2928.41	10862.97	7847.77	12402.15	14056.59	60368.43	990.53
2544.19	1485.89	199.80	5822.55	1115.83	287.02			105.78
	19.43		114.73	33.70		65.92	1242.65	28.55
	189.63			3474.24	964.31	133.16	2151.96	1208.26
	147.20			792.95		354.83	3894.74	64.00
	139.03			708.91		723.19	8009.42	245.50
	4903.50			7145.86		4820.01	14006.28	2387.64
	3613.97			7065.49		4820.01	7567.21	238.00
	1289.53			80.37			6439.07	2149.64
**145.34**	**274.14**	**51.17**	**1063.00**	**-2.83**	**6.67**	**17.30**		**297.82**

(C)1994-2024 China Academic Journal Electronic Publishing House. All rights reserved. http://www.cnki.net

5-3 综合能源平衡表

单位: 万吨标准煤

项　　目	Item	1980	1985	1990
**可供消费的能源总量**	**Total Energy Available for Consumption**	**61557**	**77603**	**96138**
一次能源生产总量	Total Primary Energy Production	63735	85546	103922
回收能	Recovery of Energy			
进口量	Imports	261	340	1310
出口量(-)	Exports (-)	3058	5774	5875
年初年末库存差额	Stock Changes in the Year	619	-2509	-3219
**能源消费总量**	**Total Energy Consumption**	**60275**	**76682**	**98703**
在总量中:	Consumption by Sector			
1.农、林、牧、渔业	Agriculture, Forestry, Animal Husbandry and Fishery	4692	4045	4852
2.工业	Industry	38986	51068	67578
3.建筑业	Construction	957	1302	1213
4.交通运输、仓储和邮政业	Transport, Storage and Post	2902	3713	4541
5.批发和零售业、住宿和餐饮业	Wholesale and Retail Trades, Hotels and Catering Services	518	766	1247
6.其他	Others	1205	2470	3473
7.居民生活	Residential	11015	13318	15799
在总量中:	Consumption by Usage			
(一) 终端消费	(I) Final Consumption	57508	73586	94289
#工业	Industry	38293	48021	63239
(二) 加工转换损失量	(II) Losses During the Process of Energy Transformation	1358	1491	2264
#炼焦	Coking	644	572	905
炼油及煤制油	Petroleum Refining and Coal-to-liquids	113	110	326
(三) 回收能(-)	(III) Recovery of Energy(-)			
(四) 损失量	(IV) Other Losses	1409	1605	2150
**平衡差额**	**Balance**	**1282**	**921**	**-2565**

注: 1.电力按等价热值折算，因此加工转换损失量中不包括发电损失量。
2.进口量包括境内飞机和轮船在境外的加油量；出口量包括境外飞机和轮船在境内的加油量。

(C)1994-2024 China Academic Journal Electronic Publishing House. All rights reserved. http://www.cnki.net

Overall Energy Balance Sheet

(10[4] tce)

1995	2000	2005	2010	2015	2016	2017	2018	2019	2020
**129535**	**144234**	**254619**	**365588**	**431636**	**434121**	**450444**	**471686**	**493178**	**507479**
129034	138570	229037	312125	362193	345954	358867	378859	397317	407295
2312	3087	7452	8958						
5456	14327	26823	57671	77695	90235	100039	110787	119064	124805
6776	9327	11257	8803	9785	11956	12669	13337	14151	12838
-491	-2424	2564	-4363	1532	9888	4206	-4623	-9052	-11784
**131176**	**146964**	**261369**	**360648**	**434113**	**441492**	**455827**	**471925**	**487488**	**498314**
5505	4233	6860	7266	8271	8585	8945	8781	9018	9263
96191	103014	187914	261377	295953	295615	302308	311151	322503	332625
1335	2207	3486	5533	7545	7847	8243	8685	9142	9320
5863	11447	19136	27102	38510	39883	42140	43617	43909	41309
2018	3251	5917	7847	11447	12042	12456	12994	13624	13171
4519	6118	10484	15052	21925	23185	24277	26262	27582	28245
15745	16695	27573	36470	50461	54336	57459	60436	61709	64380
124252	140476	250877	337469	420110	428342	442255	461020	476219	488156
89473	96871	177775	238652	282291	282809	289098	300558	311542	322677
3634	2472	3882	14294	18770	18674	19279	20803	22156	23020
	526	855	1595	4261	3887	3721	4129	4209	3938
	781	1273	1960	2866	3543	4280	5027	5895	6132
				14492	15373	15921	20465	21350	23037
3289	4016	6610	8885	9724	9849	10213	10567	10462	10175
**-1641**	**-2730**	**-6751**	**4940**	**-2477**	**-7371**	**-5383**	**-239**	**5690**	**9165**

Note: a) Electric power is converted on the basic of equal caloric value. Therefore, losses during the process of energy transformation exclude losses in power generation.

b) Data on imports include the domestic airplanes and ships refueling abroad. Data on exports include the oversea airplanes and ships refueling domestically.

(C)1994-2024 China Academic Journal Electronic Publishing House. All rights reserved. http://www.cnki.net

5-4 煤炭平衡表

单位: 万吨

项　目	Item	1980	1985	1990
**可供量**	**Total Energy Available for Consumption**	**62601**	**82777**	**102221**
生产量	Production	62015	87228	107988
进口量	Imports	199	231	200
出口量(−)	Exports (-)	632	777	1729
年初年末库存差额	Stock Changes in the Year	1019	-3906	-4239
**消费量**	**Total Energy Consumption**	**61010**	**81603**	**105523**
在消费量中:	Consumption by Sector			
1.农、林、牧、渔业	Agriculture, Forestry, Animal Husbandry and Fishery	1550	2209	2095
2.工业	Industry	43848	58613	81091
3.建筑业	Construction	556	532	438
4.交通运输、仓储和邮政业	Transport, Storage and Post	1934	2307	2161
5.批发和零售业、住宿和餐饮业	Wholesale and Retail Trades, Hotels and Catering Services	455	738	1058
6.其他	Others	1091	1580	1980
7.居民生活	Residential	11574	15624	16700
在消费量中:	Consumption by Usage			
(一) 终端消费	(I) Final Consumption	38804	52704	60206
#工业	Industry	21643	29715	35774
(二) 中间消费	(II) Intermediate Consumption			
(用于加工转换)	(Consumed in Transformation)	22205	28899	41258
#火力发电	Thermal Power	12648	16441	27204
供热	Heating		1462	2996
炼焦	Coking	6682	7304	10698
煤制油	Coal-to-liquids			
制气	Gas Production	131	191	360
(三)洗选损耗	(III) Losses in Coal Washing and Dressing	2744	3501	4059
**平衡差额**	**Balance**	**1592**	**1174**	**-3302**

注：生产量为原煤产量。

(C)1994-2024 China Academic Journal Electronic Publishing House. All rights reserved.　http://www.cnki.net

Coal Balance Sheet

(10^4 tons)

1995	2000	2005	2010	2015	2016	2017	2018	2019	2020
**133462**	**131895**	**235508**	**355578**	**397074**	**378494**	**383480**	**394848**	**405537**	**414519**
136073	138418	236515	342845	374654	341060	352356	369774	384633	390158
164	218	2622	18307	20406	25555	27093	28210	29977	30361
2862	5506	7173	1911	534	879	809	494	603	319
87	-1235	3545	-3663	2547	12758	4839	-2642	-8470	-5680
**137677**	**135690**	**243375**	**349008**	**399834**	**388820**	**391403**	**397452**	**401915**	**404860**
1857	1051	1802	2147	2625	2778	2834	2363	2202	2254
117571	121807	224766	329728	378190	367435	371160	380696	387268	390891
440	537	604	731	878	805	733	650	640	639
1315	882	811	639	492	404	353	321	283	241
977	1461	2627	3192	3864	3826	3461	2686	2378	1981
1987	1495	2727	3412	4159	4081	3580	3021	2598	2571
13530	8457	10039	9159	9627	9492	9283	7714	6547	6283
66156	50511	86386	114826	112975	101569	92841	81171	73449	72426
46050	36628	67776	95546	91331	80183	72598	64415	58802	58457
69488	81987	152208	222948	267061	272512	285325	303986	316252	321133
44440	55811	103663	153742	179568	182666	193925	205197	210159	211635
5887	8794	13542	17553	24115	26577	28983	32388	34442	36933
18396	16496	33446	49950	60874	60649	58910	61603	65673	65968
			213	679	1105	1568	2497	3240	3047
764	960	1277	1040	1320	1212	1663	2010	2459	3309
2033	3191	4782	11235	19798	14740	13237	12295	12213	11301
**-4215**	**-3795**	**-7868**	**6569**	**-2760**	**-10326**	**-7924**	**-2604**	**3623**	**9659**

Note: Data on production refer to the raw coal production.

(C)1994-2024 China Academic Journal Electronic Publishing House. All rights reserved. http://www.cnki.net

5-5 焦炭平衡表

单位：万吨

项 目	Item	1980	1985	1990
**可供量**	**Total Energy Available for Consumption**	**4315.3**	**4689.7**	**7085.8**
生产量	Production	4343.0	4802.1	7328.3
进口量	Imports		2.1	
出口量(-)	Exports (-)	27.1	36.9	129.0
年初年末库存差额	Stock Changes in the Year	-0.6	-77.6	-113.5
**消费量**	**Total Energy Consumption**	**4303.0**	**4689.7**	**6914.7**
在消费量中:	Consumption by Sector			
1.农、林、牧、渔业	Agriculture, Forestry, Animal Husbandry and Fishery	10.6	20.8	60.1
2.工业	Industry	4266.7	4627.7	6808.8
3.建筑业	Construction	11.9	7.8	5.2
4.交通运输、仓储和邮政业	Transport, Storage and Post	8.2	5.7	4.1
5.批发和零售业、住宿和餐饮业	Wholesale and Retail Trades, Hotels and Catering Services	0.9	2.7	7.7
6.其他	Others	4.7	2.0	1.9
7.居民生活	Residential		23.0	26.9
在消费量中:	Consumption by Usage			
(一) 终端消费	(I) Final Consumption	4294.7	4677.9	6846.3
#工业	Industry	4258.4	4615.9	6740.4
(二) 中间消费	(II) Intermediate Consumption			
(用于加工转换)	(Consumed in Transformation)	8.3	11.8	68.4
制气	Gas Production	8.3	11.8	68.4
(三) 损失量	(III) Losses in Coal Washing and Dressing			
**平衡差额**	**Balance**	**12.3**		**171.1**

(C)1994-2024 China Academic Journal Electronic Publishing House. All rights reserved. http://www.cnki.net

Coke Balance Sheet

(10[4] tons)

1995	2000	2005	2010	2015	2016	2017	2018	2019	2020
**12207.1**	**10892.3**	**25084.4**	**38707.1**	**44018.9**	**45428.6**	**43739.5**	**43257.9**	**46533.7**	**47312.0**
13424.5	12184.0	26511.7	38657.8	44822.5	44911.5	43168.4	44751.4	47295.8	47188.2
0.1		0.5	11.0	0.4	0.1	1.0	9.1	52.3	297.9
886.1	1519.7	1276.4	335.0	964.8	1011.9	807.9	975.8	652.3	349.2
-331.4	228.0	-151.4	373.3	160.8	1529.0	1378.0	-526.8	-162.2	175.1
**10725.3**	**10840.8**	**25105.8**	**38702.8**	**44058.7**	**45462.4**	**43743.1**	**43716.6**	**46426.0**	**48310.4**
128.6	70.9	63.5	46.8	49.5	53.1	38.4	103.0	59.7	23.2
10412.0	10554.6	24860.9	38598.7	43923.0	45324.7	43609.1	43560.9	46320.2	48272.2
10.8	19.0	18.4	5.8	6.7	7.1	12.6	10.6	9.7	3.6
10.1	11.2	1.1	0.1	3.0	3.2	6.0	0.4	0.4	
25.7	35.7	64.1	5.1	40.1	41.3	49.4	19.0	16.9	
6.4	12.2	7.6	2.8	5.4	5.6	5.9	6.3	6.3	
131.6	137.2	90.3	43.5	31.2	27.4	21.8	16.4	12.8	11.4
10648.0	10697.9	24877.9	38574.6	43775.0	45454.4	43741.5	43693.7	46336.6	47762.5
10334.7	10411.7	24633.0	38470.5	43639.2	45316.7	43607.4	43538.0	46230.9	47724.3
77.3	142.9	227.9	128.2	283.8	8.0	1.6	22.8	89.3	547.9
77.3	142.9	227.9	128.2	1.8	1.9	1.6	1.6	16.5	17.6
**1481.8**	**51.6**	**-21.4**	**4.3**	**-39.8**	**-33.8**	**-3.6**	**-458.6**	**107.7**	**-998.4**

(C)1994-2024 China Academic Journal Electronic Publishing House. All rights reserved. http://www.cnki.net

5-6 石油平衡表

单位: 万吨

项　　目	Item	1980	1985	1990
**可供量**	**Total Energy Available for Consumption**	**8794.5**	**9193.7**	**11435.0**
生产量	Production	10594.6	12489.5	13830.6
进口量	Imports	82.7	90.0	755.6
出口量(-)	Exports (-)	1806.2	3630.4	3110.4
年初年末库存差额	Stock Changes in the Year	-76.6	244.6	-40.8
**消费量**	**Total Energy Consumption**	**8757.4**	**9168.8**	**11485.6**
在消费量中:	Consumption by Sector			
1.农、林、牧、渔业	Agriculture, Forestry, Animal Husbandry and Fishery	814..9	758.7	1033.6
2.工业	Industry	6203.2	6171.4	7321.6
3.建筑业	Construction	175.2	292.2	327.3
4.交通运输、仓储和邮政业	Transport, Storage and Post	911.5	1176.4	1683.2
5.批发和零售业、住宿和餐饮业	Wholesale and Retail Trades, Hotels and Catering Services	29.0	38.1	77.6
6.其他	Others	481.7	506.1	757.8
7.居民生活	Residential	141.9	225.9	284.5
在消费量中:	Consumption by Usage			
(一) 终端消费	(I) Final Consumption	6311.0	7063.3	9304.7
#工业	Industry	3780.3	4462.0	5180.4
(二) 中间消费	(II) Intermediate Consumption			
(用于加工转换)	(Consumed in Transformation)	2183.6	1858.5	1630.4
火力发电	Thermal Power	2065.4	1425.5	1234.4
供热	Heating		285.6	356.3
制气	Gas Production	36.7	34.5	39.7
炼油损失量	Losses in Petroleum Refining	81.5	112.9	295.8
(三) 损失量	(III) Other Losses	262.8	247.0	254.7
**平衡差额**	**Balance**	**37.1**	**24.9**	**-50.6**

注：1.生产量为原油产量。
2.进口量包括境内飞机和轮船在境外的加油量；出口量包括境外飞机和轮船在境内的加油量。

(C)1994-2024 China Academic Journal Electronic Publishing House. All rights reserved. http://www.cnki.net

Petroleum Balance Sheet

(10^4 tons)

1995	2000	2005	2010	2015	2016	2017	2018	2019	2020
**16072.7**	**22631.4**	**32539.1**	**44178.4**	**55688.0**	**57710.6**	**60810.8**	**63726.6**	**66900.9**	**67553.7**
15004.4	16300.0	18135.3	20301.4	21455.6	19968.5	19150.6	18932.4	19101.4	19476.9
3673.2	9748.5	17163.2	29437.2	39748.6	44502.9	49141.2	54094.3	58102.2	61271.7
2454.5	2172.1	2888.1	4079.0	5128.2	6382.9	7026.7	7557.4	8211.4	7551.0
-151.0	-1245.0	128.8	-1481.2	-388.1	-377.8	-454.3	-1742.7	-2091.4	-5643.8
**16064.9**	**22495.9**	**32547.0**	**44101.0**	**55960.2**	**57692.9**	**60395.9**	**62245.1**	**64506.5**	**65369.1**
1203.2	788.5	1451.7	1382.5	1733.4	1730.3	1786.4	1724.9	1748.2	1773.1
9349.3	11248.5	14030.4	18555.0	19718.0	20382.5	21486.7	22460.3	25210.6	27711.1
242.8	840.6	1502.2	2483.1	3384.3	3599.1	3803.5	3935.7	4055.1	4180.3
2863.6	6399.0	10928.5	15079.3	20663.1	21146.1	22075.8	22738.6	22109.6	20481.4
333.9	247.0	375.6	481.0	615.7	584.9	601.1	599.0	608.4	583.3
1390.3	1635.9	1974.2	2578.2	3683.3	3537.1	3502.7	3458.2	3460.6	3469.9
682.0	1336.5	2284.4	3541.9	6162.2	6712.8	7139.7	7328.4	7314.0	7170.1
13676.3	19950.1	29495.6	41243.4	52945.7	54387.0	56880.0	58623.0	61018.5	62085.2
7095.5	8860.0	11107.5	15857.8	16739.7	17100.2	17980.5	18847.6	21732.4	24428.4
2230.0	2352.9	2896.0	2663.3	2926.9	3264.3	3468.9	3593.3	3453.6	3265.6
1358.5	1178.2	1306.4	385.3	265.5	284.6	280.6	309.3	308.3	321.5
399.9	427.0	429.1	593.1	493.2	517.8	522.6	590.2	653.8	677.7
51.6	25.9	14.4			4.8	4.7	4.7	3.7	28.9
420.1	721.9	1146.1	1684.8	2168.2	2457.2	2661.0	2689.0	2487.9	2232.9
158.6	192.9	155.4	194.4	87.6	41.6	47.0	28.8	34.5	18.3
**7.8**	**135.4**	**-7.9**	**77.4**	**-272.2**	**17.7**	**414.9**	**1481.5**	**2394.3**	**2184.6**

Note: a) Data on production refer to the crude oil production.
b) Data on imports include the domestic airplanes and ships refueling abroad. Data on exports include the oversea airplanes and ships refueling domestically.

(C)1994-2024 China Academic Journal Electronic Publishing House. All rights reserved. http://www.cnki.net

5-7 原油平衡表

单位: 万吨

项　　目	Item	1980	1985	1990
**可供量**	**Total Energy Available for Consumption**	**9222.9**	**9516.5**	**11770.6**
生产量	Production	10594.6	12489.5	13830.6
进口量	Imports	36.6		292.3
出口量(-)	Exports (-)	1330.9	3003.0	2399.0
年初年末库存差额	Stock Changes in the Year	-77.4	30.0	46.7
**消费量**	**Total Energy Consumption**	**9205.0**	**9509.5**	**11762.2**
在消费量中:	Consumption by Sector			
1.农、林、牧、渔业	Agriculture, Forestry, Animal Husbandry and Fishery	8.0	0.8	0.2
2.工业	Industry	9112.0	9389.9	11653.8
3.建筑业	Construction	28.8	74.0	55.2
4.交通运输、仓储和邮政业	Transport, Storage and Post	50.1	44.3	52.1
5.批发和零售业、住宿和餐饮业	Wholesale and Retail Trades, Hotels and Catering Services		0.1	0.3
6.其他	Others	6.1	0.4	0.6
7.居民生活	Residential			
在消费量中:	Consumption by Usage			
(一) 终端消费	(I) Final Consumption	499.6	350.4	402.1
#工业	Industry	429.7	254.9	333.4
(二) 中间消费	(II) Intermediate Consumption			
(用于加工转换)	(Consumed in Transformation)	8443.0	8929.7	11106.9
火力发电	Thermal Power	574.0	279.5	124.6
供热	Heating		61.3	21.1
炼油	Petroleum Refineries	7869.0	8588.9	10961.2
(三) 油田原油损失量	(III) Losses in Oil Field for Crude Oil	262.4	229.4	253.2
**平衡差额**	**Balance**	**17.9**	**7.0**	**8.4**

(C)1994-2024 China Academic Journal Electronic Publishing House. All rights reserved. http://www.cnki.net

Crude Oil Balance Sheet

(10[4] tons)

1995	2000	2005	2010	2015	2016	2017	2018	2019	2020
**14794.9**	**21383.0**	**30089.2**	**42876.6**	**54593.5**	**57332.3**	**59969.0**	**63849.2**	**68007.5**	**69969.6**
15004.4	16300.0	18135.3	20301.4	21455.6	19968.5	19150.6	18932.4	19101.4	19476.9
1709.0	7026.5	12681.7	23768.2	33548.3	38100.7	41946.2	46188.5	50567.6	54200.7
1822.7	1030.6	806.7	303.0	286.6	294.1	486.1	262.7	81.0	163.8
-95.8	-912.9	78.8	-890.0	-123.8	-442.8	-641.8	-1009.1	-1580.5	-3544.1
**14886.4**	**21232.0**	**30088.9**	**42874.6**	**54788.3**	**57125.9**	**59402.2**	**63004.3**	**67268.3**	**69477.1**
10.1									
14716.3	21052.1	29962.1	42716.6	54752.4	57103.6	59393.5	62995.5	67259.1	69476.5
2.7	3.3								
156.8	175.1	126.9	158.0	35.9	22.3	8.7	8.8	9.2	0.6
0.5	0.2								
1390.3	1.4								
309.9	636.8	850.4	806.1	782.7	630.1	364.7	348.3	326.5	419.0
274.7	612.3	850.4	806.1	782.7	630.1	364.7	348.3	326.5	419.0
14419.4	20404.3	29084.8	41876.4	53918.4	56455.4	58991.5	62627.7	66907.9	69040.5
61.6	85.0	41.3	3.7	12.5	13.1	14.2	15.3	16.0	16.5
4.4	14.0	3.0	3.3	6.7					
14353.4	20305.3	29040.5	41869.4	53899.2	56442.3	58977.3	62612.4	66891.9	69023.9
157.1	190.9	153.8	192.0	87.2	40.4	46.0	28.3	33.8	17.6
**-91.5**	**151.0**	**0.2**	**2.1**	**-194.8**	**206.4**	**566.8**	**844.8**	**739.2**	**492.5**

(C)1994-2024 China Academic Journal Electronic Publishing House. All rights reserved. http://www.cnki.net

5-8 燃料油平衡表

单位: 万吨

项　　目	Item	1980	1985	1990
**可供量**	**Total Energy Available for Consumption**	**3096.1**	**2848.0**	**3320.7**
生产量	Production	3142.0	2835.8	3267.9
进口量	Imports	39.0	70.0	167.3
出口量(-)	Exports (-)	45.4	64.9	97.2
年初年末库存差额	Stock Changes in the Year	-39.5	7.1	-17.3
**消费量**	**Total Energy Consumption**	**3073.7**	**2837.4**	**3367.8**
在消费量中:	Consumption by Sector			
1.农、林、牧、渔业	Agriculture, Forestry, Animal Husbandry and Fishery	2.3	3.1	2.9
2.工业	Industry	2937.4	2662.2	3091.7
3.建筑业	Construction	15.0	18.9	47.3
4.交通运输、仓储和邮政业	Transport, Storage and Post	109.0	144.1	208.2
5.批发和零售业、住宿和餐饮业	Wholesale and Retail Trades, Hotels and Catering Services	2.9	3.1	1.6
6.其他	Others	7.1	6.0	16.1
7.居民生活	Residential			
在消费量中:	Consumption by Usage			
(一) 终端消费	(I) Final Consumption	1617.9	1538.8	2042.6
#工　业	Industry	1481.6	1363.5	1766.5
(二) 中间消费	(II) Intermediate Consumption			
(用于加工转换)	(Consumed in Transformation)	1455.8	1296.1	1325.2
火力发电	Thermal Power	1419.1	1042.3	977.3
供　热	Heating		219.3	308.3
炼油再投入量	Petroleum Production			
制　气	Gas Production	36.7	34.5	39.6
(三)损失量	(III) Other Losses		2.5	
**平衡差额**	**Balance**	**22.4**	**10.6**	**-47.1**

(C)1994-2024 China Academic Journal Electronic Publishing House. All rights reserved. http://www.cnki.net

Fuel Oil Balance Sheet

(10[4] tons)

	1995	2000	2005	2010	2015	2016	2017	2018	2019	2020
	**3717.3**	**3836.7**	**4237.3**	**3765.4**	**4632.8**	**4644.5**	**4895.8**	**4585.2**	**4851.0**	**5344.3**
	2960.8	2053.7	1767.4	2487.0	3963.0	4236.9	4563.5	3899.7	4506.0	5780.7
	859.1	1704.3	2883.9	2695.2	2068.2	1743.2	1915.3	2271.3	2028.5	1760.9
	68.6	57.9	427.6	1419.7	1402.1	1342.0	1516.7	1673.2	1548.5	2132.2
	-34.0	136.6	13.5	3.0	3.7	6.4	-66.2	87.4	-135.0	-65.1
	**3693.7**	**3872.8**	**4244.2**	**3758.0**	**4662.0**	**4631.0**	**4887.3**	**4536.1**	**4690.3**	**5364.6**
	8.4	0.4	0.7	1.1	0.9	1.0	1.3	1.3	1.2	1.1
	3406.2	2975.1	2986.9	2377.3	3133.0	3035.4	3043.7	2688.2	2612.5	3262.3
	14.2	16.7	14.2	30.8	53.5	51.9	43.2	31.8	31.8	40.7
	227.5	850.0	1201.0	1326.7	1439.5	1511.4	1771.3	1795.7	2025.3	2042.0
	6.6	11.6	27.5	8.6	19.0	17.2	15.1	10.1	10.2	11.8
	30.8	19.0	13.9	13.5	16.1	14.1	12.5	9.0	9.2	6.6
	2262.8	2741.4	2989.9	2403.2	2123.7	2060.4	2196.9	2155.9	2295.4	2333.0
	1975.3	1843.7	1732.6	1022.5	594.8	464.8	353.4	308.0	217.6	230.7
	1430.9	1131.3	1254.3	1354.8	2538.3	2570.7	2690.4	2380.2	2395.0	3031.6
	1071.5	814.2	1068.7	123.9	31.5	31.1	16.6	15.1	13.9	29.4
	307.8	291.2	171.1	201.3	165.1	158.5	67.0	59.5	60.2	231.4
				1029.6	2341.6	2381.1	2606.7	2305.5	2320.8	2770.8
	51.6	25.9	14.4							
	**23.6**	**-36.1**	**-6.9**	**7.4**	**-29.2**	**13.5**	**8.5**	**49.1**	**160.7**	**-20.3**

(C)1994-2024 China Academic Journal Electronic Publishing House. All rights reserved. http://www.cnki.net

5-9 汽油平衡表

单位：万吨

项　　目	Item	1980	1985	1990
**可供量**	**Total Energy Available for Consumption**	**999.4**	**1399.6**	**1884.1**
生产量	Production	1079.0	1471.9	2173.4
进口量	Imports		0.3	16.9
出口量(-)	Exports (-)	117.8	129.9	233.8
年初年末库存差额	Stock Changes in the Year	38.2	57.3	-72.4
**消费量**	**Total Energy Consumption**	**998.6**	**1396.3**	**1899.5**
在消费量中:	Consumption by Sector			
1.农、林、牧、渔业	Agriculture, Forestry, Animal Husbandry and Fishery	53.3	122.3	145.9
2.工业	Industry	273.2	451.3	589.3
3.建筑业	Construction	54.1	73.0	89.5
4.交通运输、仓储和邮政业	Transport, Storage and Post	404.9	477.4	620.1
5.批发和零售业、住宿和餐饮业	Wholesale and Retail Trades, Hotels and Catering Services	19.4	23.4	46.0
6.其他	Others	193.7	238.3	390.7
7.居民生活	Residential		10.6	18.0
**平衡差额**	**Balance**	**0.8**	**3.3**	**-15.4**

5-10 煤油平衡表

单位：万吨

项　　目	Item	1980	1985	1990
**可供量**	**Total Energy Available for Consumption**	**359.0**	**383.2**	**350.9**
生产量	Production	398.5	405.3	392.5
进口量	Imports		15.2	26.1
出口量(-)	Exports (-)	46.8	46.0	55.5
年初年末库存差额	Stock Changes in the Year	2.3	8.7	-12.2
**消费量**	**Total Energy Consumption**	**365.9**	**385.5**	**350.9**
在消费量中:	Consumption by Sector			
1.农、林、牧、渔业	Agriculture, Forestry, Animal Husbandry and Fishery	2.3	3.3	3.1
2.工业	Industry	15.7	20.1	20.6
3.建筑业	Construction	0.8	1.3	1.3
4.交通运输、仓储和邮政业	Transport, Storage and Post	31.4	56.2	93.4
5.批发和零售业、住宿和餐饮业	Wholesale and Retail Trades, Hotels and Catering Services	0.2	0.1	0.6
6.其他	Others	216.7	182.9	127.3
7.居民生活	Residential	98.8	121.6	104.6
**平衡差额**	**Balance**	**-6.9**	**-2.3**	

(C)1994-2024 China Academic Journal Electronic Publishing House. All rights reserved. http://www.cnki.net

Gasoline Balance Sheet

(10⁴ tons)

1995	2000	2005	2010	2015	2016	2017	2018	2019	2020
**2902.0**	**3504.5**	**4855.3**	**6964.3**	**11385.0**	**11829.4**	**12200.5**	**13035.3**	**13690.3**	**12829.0**
3051.6	4134.7	5433.6	7410.5	12103.6	12932.0	13276.2	14264.7	14880.7	14256.7
15.9				17.0	20.8	1.6	44.5	33.3	48.0
193.1	467.7	559.7	517.0	589.3	969.3	1051.4	1287.9	1637.1	1600.0
27.6	-162.5	-18.6	70.8	-146.3	-154.2	-26.0	13.9	413.3	124.3
**2909.6**	**3504.6**	**4854.9**	**6956.2**	**11368.5**	**11866.0**	**12296.3**	**13055.3**	**13628.0**	**12767.2**
179.7	89.2	159.6	169.1	231.3	224.4	229.6	242.9	253.2	257.3
812.4	682.0	441.7	689.5	477.1	436.3	382.1	296.5	262.0	184.0
103.6	115.6	172.1	274.7	408.6	437.3	452.3	505.0	499.9	508.4
982.3	1527.8	2430.1	3274.9	5306.6	5511.1	5698.5	6067.6	6244.9	5573.6
197.2	69.8	129.4	168.2	243.3	240.9	244.5	275.5	287.9	273.2
570.7	792.7	998.2	1166.2	2108.5	2046.4	2075.0	2163.6	2240.9	2253.1
63.7	227.6	523.8	1213.7	2593.1	2969.7	3214.2	3504.2	3839.1	3717.6
**-7.6**	**-0.1**	**0.4**	**8.1**	**16.6**	**-36.7**	**-95.8**	**-20.0**	**62.3**	**61.9**

Kerosene Balance Sheet

(10⁴ tons)

1995	2000	2005	2010	2015	2016	2017	2018	2019	2020
**486.4**	**880.9**	**1070.0**	**1767.6**	**2732.6**	**3020.7**	**3332.8**	**3714.3**	**4002.8**	**3412.4**
445.8	872.3	1006.5	1924.4	3658.6	3983.9	4230.9	4770.3	5322.6	4129.4
115.7	322.5	476.1	726.1	716.4	776.7	852.3	942.3	930.0	514.0
62.4	256.3	447.6	870.5	1626.6	1721.7	1765.2	1947.0	2239.8	1226.3
-12.7	-57.6	35.0	-12.3	-15.8	-18.2	14.8	-51.3	-10.0	-4.8
**512.1**	**871.6**	**1076.8**	**1765.2**	**2663.7**	**2970.7**	**3326.4**	**3653.5**	**3950.2**	**3352.1**
3.6	1.5	1.6	0.9	1.1	2.2	1.5	4.9	11.0	11.0
44.9	84.0	57.5	40.2	21.2	20.0	14.5	24.9	11.0	9.4
3.5	4.0		8.8	12.5	10.0	9.7	17.3	16.0	10.8
250.0	535.9	952.4	1601.1	2504.9	2814.9	3173.3	3462.5	3689.2	3110.8
8.5	14.0	3.7	35.0	11.7	11.2	11.3	15.5	15.5	14.8
137.3	160.1	36.2	58.7	83.3	85.9	88.4	103.8	184.2	183.0
64.3	72.2	25.5	20.5	29.1	26.4	27.6	24.6	23.4	12.4
**-25.7**	**9.3**	**-6.8**	**2.4**	**68.9**	**50.0**	**6.4**	**60.8**	**52.6**	**60.3**

(C)1994-2024 China Academic Journal Electronic Publishing House. All rights reserved. http://www.cnki.net

5-11 柴油平衡表

单位：万吨

项　　目	Item	1980	1985	1990
**可供量**	**Total Energy Available for Consumption**	**1663.2**	**1944.1**	**2689.4**
生产量	Production	1827.8	2023.2	2609.0
进口量	Imports	2.1	4.5	233.8
出口量(－)	Exports (-)	166.5	225.6	169.8
年初年末库存差额	Stock Changes in the Year	-0.2	142.0	16.4
**消费量**	**Total Energy Consumption**	**1663.2**	**1939.4**	**2691.7**
在消费量中:	Consumption by Sector			
1.农、林、牧、渔业	Agriculture, Forestry, Animal Husbandry and Fishery	749.0	629.2	881.5
2.工业	Industry	457.4	644.1	728.1
3.建筑业	Construction	76.5	125.0	133.0
4.交通运输、仓储和邮政业	Transport, Storage and Post	316.1	454.4	709.4
5.批发和零售业、住宿和餐饮业	Wholesale and Retail Trades, Hotels and Catering Services	6.5	10.9	22.5
6.其他	Others	57.7	74.0	217.0
7.居民生活	Residential			
在消费量中:	Consumption by Usage			
(一) 终端消费	(I) Final Consumption	1590.9	1827.4	2564.8
#工　业	Industry	385.1	532.1	601.2
(二) 中间消费	(II) Intermediate Consumption			
(用于加工转换)	(Consumed in Transformation)	72.3	108.6	126.9
火力发电	Thermal Power	72.3	103.6	124.5
供　热	Heating		5.0	2.4
(三) 损失量	(III) Other Losses		3.4	
**平衡差额**	**Balance**		**4.7**	**-2.3**

(C)1994-2024 China Academic Journal Electronic Publishing House. All rights reserved. http://www.cnki.net

Diesel Oil Balance Sheet

(10^4 tons)

1995	2000	2005	2010	2015	2016	2017	2018	2019	2020
**4404.2**	**6806.5**	**10972.6**	**14701.9**	**17353.6**	**16765.2**	**16994.5**	**16567.3**	**15359.6**	**14459.7**
3972.6	7079.6	11090.2	14924.4	18007.9	17917.7	18667.9	18360.1	17308.3	16529.9
645.3	51.9	61.0	190.2	71.5	116.1	107.6	89.0	144.5	137.5
169.5	77.5	170.9	490.2	731.3	1556.9	1736.8	1872.8	2189.1	2011.2
-44.2	-247.6	-7.7	77.5	5.5	288.3	-44.2	-9.0	95.9	-196.5
**4321.4**	**6806.2**	**10974.9**	**14699.0**	**17360.3**	**16839.0**	**16916.5**	**16409.6**	**14917.9**	**14282.7**
1001.4	697.1	1286.3	1206.7	1492.9	1495.9	1546.8	1468.2	1475.1	1497.2
1189.9	1696.5	1710.0	2090.0	1516.4	1412.9	1459.9	1259.5	1290.6	1026.1
118.2	205.9	386.6	490.2	555.7	561.3	596.1	543.4	530.3	503.9
1246.6	3293.8	6169.4	8657.6	11162.8	11068.5	11173.7	11166.9	9867.3	9532.0
103.6	95.9	116.0	196.6	257.7	232.0	233.8	211.8	203.9	197.6
645.7	638.7	900.1	1287.2	1384.2	1307.2	1233.3	1107.4	954.0	946.2
16.1	178.4	406.4	770.7	990.7	761.3	673.0	652.3	596.8	579.8
4070.0	6578.6	10889.4	14655.2	17280.4	16736.4	16722.4	16340.0	14805.6	14136.2
938.5	1468.8	1624.5	2046.2	1436.5	1310.3	1265.8	1189.9	1178.3	879.6
251.4	227.7	85.5	43.8	79.9	102.7	194.1	69.6	112.3	146.5
204.9	227.7	81.9	40.1	22.4	29.2	23.9	26.4	24.2	27.6
46.6		3.6	3.8	6.2	6.1	5.9	5.2	5.3	2.9
**82.7**	**0.3**	**-2.4**	**2.9**	**-6.7**	**-73.9**	**78.0**	**157.8**	**441.6**	**177.0**

(C)1994-2024 China Academic Journal Electronic Publishing House. All rights reserved. http://www.cnki.net

5-12　液化石油气平衡表

单位：万吨

项　　目	Item	1980	1985	1990
**可供量**	**Total Energy Available for Consumption**	**122.5**	**157.3**	**258.5**
生产量	Production	122.5	159.7	261.6
进口量	Imports			
出口量(−)	Exports (-)		1.9	1.1
年初年末库存差额	Stock Changes in the Year		-0.5	-2.0
**消费量**	**Total Energy Consumption**	**119.6**	**155.7**	**254.2**
在消费量中:	Consumption by Sector			
1.农、林、牧、渔业	Agriculture, Forestry, Animal Husbandry and Fishery			
2.工业	Industry	76.1	59.9	82.0
3.建筑业	Construction			1.0
4.交通运输、仓储和邮政业	Transport, Storage and Post			
5.批发和零售业、住宿和餐饮业	Wholesale and Retail Trades, Hotels and Catering Services		0.5	6.6
6.其他	Others	0.4	4.5	6.1
7.居民生活	Residential	43.1	90.8	158.5
**平衡差额**	**Balance**	**2.9**	**1.6**	**4.3**

5-13　天然气平衡表

单位：亿立方米

项　　目	Item	1980	1985	1990
**可供量**	**Total Energy Available for Consumption**	**142.7**	**129.3**	**153.0**
生产量	Production	142.7	129.3	153.0
进口量	Imports			
出口量(−)	Exports (-)			
年初年末库存差额	Stock Changes in the Year			
**消费量**	**Total Energy Consumption**	**140.6**	**129.3**	**152.5**
在消费量中:	Consumption by Sector			
1.农、林、牧、渔业	Agriculture, Forestry, Animal Husbandry and Fishery			
2.工业	Industry	131.4	109.6	120.2
3.建筑业	Construction	6.0	14.1	10.6
4.交通运输、仓储和邮政业	Transport, Storage and Post	0.7	0.8	1.9
5.批发和零售业、住宿和餐饮业	Wholesale and Retail Trades, Hotels and Catering Services			
6.其他	Others	0.5	0.5	1.2
7.居民生活	Residential	2.0	4.3	18.6
**平衡差额**	**Balance**	**2.1**		**0.5**

注：从2010年起包括液化天然气数据。

(C)1994-2024 China Academic Journal Electronic Publishing House. All rights reserved.　http://www.cnki.net

Liquefied Petroleum Gas Balance Sheet

(10^4 tons)

1995	2000	2005	2010	2015	2016	2017	2018	2019	2020
**774.3**	**1396.2**	**2052.2**	**2323.8**	**4008.2**	**5034.0**	**5472.9**	**5733.4**	**6160.6**	**6381.1**
540.8	916.6	1432.7	2092.3	2934.4	3503.9	3677.3	3915.6	4210.0	4489.0
232.6	481.7	617.0	327.0	1244.0	1678.5	1921.9	1966.4	2109.3	2004.6
7.1	1.6	2.7	93.0	144.2	132.3	132.2	113.5	140.9	94.9
8.0	-0.6	5.2	-2.5	-26.0	-16.0	5.9	-35.1	-17.8	-17.7
**750.6**	**1389.7**	**2046.5**	**2321.9**	**3961.2**	**5015.1**	**5457.8**	**5673.1**	**6066.4**	**6221.2**
0.1	0.4	3.5	4.7	7.2	6.8	7.1	7.6	7.8	6.5
192.5	426.1	534.4	586.8	1113.9	1766.8	1896.3	2215.5	2870.3	3064.8
0.5	8.9	6.3	7.2	15.1	14.8	15.8	17.0	14.0	11.3
0.5	16.5	48.7	61.0	100.3	104.2	123.7	125.1	156.5	111.2
17.4	55.5	99.0	72.6	84.0	83.6	96.4	86.2	90.9	85.9
5.7	24.0	25.8	52.6	91.4	83.5	93.5	74.4	72.2	81.1
534.0	858.3	1328.7	1537.0	2549.3	2955.4	3225.0	3147.3	2854.7	2860.4
**23.7**	**6.5**	**5.7**	**1.9**	**47.0**	**18.9**	**15.1**	**60.3**	**94.2**	**159.9**

Natural Gas Balance Sheet

(10^8 cu.m)

1995	2000	2005	2010	2015	2016	2017	2018	2019	2020
**179.5**	**240.6**	**463.5**	**1082.3**	**1925.0**	**2080.5**	**2390.7**	**2814.3**	**3057.5**	**3270.2**
179.5	*272.0*	*493.2*	*957.9*	*1346.1*	*1368.7*	*1480.4*	*1601.6*	*1761.7*	*1925.0*
			164.7	611.4	745.6	945.6	1246.4	1331.8	1397.0
	31.4	29.7	40.3	32.5	33.8	35.3	33.6	36.1	51.7
**177.4**	**245.0**	**466.1**	**1080.2**	**1931.8**	**2078.1**	**2393.7**	**2817.1**	**3059.7**	**3339.9**
			0.5	0.9	1.1	1.1	1.3	1.2	1.3
154.4	199.0	327.2	691.8	1234.5	1338.6	1575.2	1940.1	2092.1	2304.0
0.3	0.8	1.5	1.2	2.2	1.9	1.8	2.5	2.8	2.6
1.6	8.8	38.0	106.7	237.6	254.8	284.7	286.2	341.5	354.3
0.6	3.4	10.8	27.2	51.3	53.7	57.6	60.8	62.5	62.1
1.2	0.6	9.1	26.0	45.4	48.2	52.9	57.9	57.3	55.6
19.4	32.3	79.4	226.9	359.8	379.7	420.3	468.4	502.3	560.0
**2.1**	**-4.4**	**-2.6**	**2.1**	**-6.7**	**2.4**	**-3.0**	**-2.8**	**-2.2**	**0.3**

Note: Include the data of Liquefied Natural Gas since 2010.

(C)1994-2024 China Academic Journal Electronic Publishing House. All rights reserved. http://www.cnki.net

5-14 电力平衡表

单位：亿千瓦时

项　　目	Item	1980	1985	1990
**可供量**	**Total Energy Available for Consumption**	**3006.3**	**4117.6**	**6230.4**
生产量	Production	3006.3	4106.9	6212.0
#水电	#Hydro Power	582.1	923.7	1267.2
火电	Thermal Power	2424.2	3183.2	4944.8
核电	Nuclear Power			
风电	Wind Power			
进口量	Imports		11.1	19.3
出口量(-)	Exports (-)		0.4	0.9
**消费量**	**Total Energy Consumption**	**3006.3**	**4117.6**	**6230.4**
在消费量中:	Consumption by Sector			
1.农、林、牧、渔业	Agriculture, Forestry, Animal Husbandry and Fishery	270.0	317.4	426.8
2.工业	Industry	2471.9	3283.4	4873.3
3.建筑业	Construction	47.1	71.2	65.0
4.交通运输、仓储和邮政业	Transport, Storage and Post	26.5	63.4	105.9
5.批发和零售业、住宿和餐饮业	Wholesale and Retail Trades, Hotels and Catering Services	16.8	38.0	76.2
6.其他	Others	68.8	121.7	202.4
7.居民生活	Residential	105.2	222.5	480.8
在消费量中:	Consumption by Usage			
(一) 终端消费	(I) Final Consumption	2763.4	3813.3	5795.8
#工业	#Industry	2229.0	2979.1	4438.7
(二) 输配电损失量	(II) Losses in Transmission	242.9	304.3	434.6

(C)1994-2024 China Academic Journal Electronic Publishing House. All rights reserved. http://www.cnki.net

Electricity Balance Sheet

(10^{8} kW•h)

1995	2000	2005	2010	2015	2016	2017	2018	2019	2020
**10023.4**	**13472.7**	**24940.8**	**41936.5**	**58021.3**	**61204.4**	**65914.0**	**71509.2**	**74866.3**	**77620.2**
10077.3	13556.0	25002.6	42071.6	58145.7	61331.6	66044.5	71661.3	75034.3	77790.6
1905.8	2224.1	3970.2	7221.7	11302.7	11840.5	11978.7	12317.9	13044.4	13552.1
8043.2	11141.9	20473.4	33319.3	42841.9	44370.7	47546.0	50963.2	52201.5	53302.5
128.3	167.4	530.9	738.8	1707.9	2132.9	2480.7	2943.6	3483.5	3662.5
			446.2	1857.7	2370.7	2972.3	3659.7	4060.3	4664.7
6.4	15.5	50.1	55.5	62.1	61.9	64.2	56.9	48.6	47.5
60.3	98.8	111.9	190.6	186.5	189.1	194.7	209.1	216.5	217.9
**10023.4**	**13472.4**	**24940.3**	**41934.5**	**58020.0**	**61205.1**	**65914.0**	**71508.2**	**74866.1**	**77620.2**
582.4	533.0	776.3	976.5	1039.8	1091.9	1175.1	1242.5	1336.2	1422.1
7659.8	10004.6	18521.7	30871.8	41550.0	42996.9	46052.8	49094.9	50698.3	52353.4
159.6	159.8	233.9	483.2	698.7	725.6	789.2	887.8	991.2	1011.1
182.3	281.2	430.3	734.5	1125.6	1251.5	1418.0	1608.5	1752.3	1751.0
199.5	418.7	752.3	1292.0	2122.0	2323.8	2526.6	2900.4	3187.1	3169.0
234.2	623.2	1340.9	2451.8	3918.6	4394.8	4880.6	5716.5	6263.8	6517.0
1005.6	1452.0	2884.8	5124.6	7565.2	8420.6	9071.6	10057.6	10637.2	11396.5
9278.9	12535.7	23233.8	39366.3	55032.1	58142.2	62718.1	68156.5	71536.0	74386.7
6915.3	9067.9	16815.2	28303.5	38562.1	39934.0	42857.0	45743.2	47368.2	49120.0
744.5	936.7	1706.5	2568.2	2987.9	3062.9	3195.8	3351.7	3330.1	3233.5

(C)1994-2024 China Academic Journal Electronic Publishing House. All rights reserved. http://www.cnki.net
