# Supplementary material for: Prediction analysis of carbon emission in China’s electricity industry based on the dual carbon background
Source: PLoS One. 2024 May 17;19(5):e0302068. doi: 10.1371/journal.pone.0302068 (PMC11101092; doi:10.1371/journal.pone.0302068)
Supplement: S3 File — (ZIP) [file pone.0302068.s003.zip › China Electric Power Yearbook 2001-2021/统计资料-2001.pdf]

全 国 部 分

国民经济主要指标

	单　位	2000 年	1999 年	比 1999 年增长（%）
全国总人口	万人	126583	125909	0.54
城镇人口	万人	45844	38892	17.88
乡村人口	万人	80739	87017	-7.21
年末从业人员	万人	71150	70586	0.80
国内生产总值	亿元	89404	82067	8.00
第一产业	亿元	14212	14472	2.40
第二产业	亿元	45488	40558	9.60
第三产业	亿元	29704	27038	7.80
工业增加值	亿元	39570	35087	9.90
全社会固定资产投资总额	亿元	32619	29855	9.30
国有经济投资总额	亿元	23284	21320	9.20
基本建设投资	亿元	13215	12455	6.10
更新改造投资	亿元	5077	4485	13.20
房地产	亿元	4902	4103	19.50
一次能源生产总量(标准煤)	万 t	109000	109126	-0.12
原　煤	万 t	99800	104500	-4.50
原　油	万 t	16300	16000	1.88
天然气	亿 m^3	277	252	9.92

注　国内生产总值按现价计算，增长率按可比价计算；工业总产值按当年价格计算。

2000 年电力生产基本情况

	单　位	2000 年	1999 年	比 1999 年增长（%）
**一、发电装机容量**	**万 kW**	**31932.09**	**29876.79**	**6.88**
水　电	万 kW	7935.22	7297.08	8.75
火　电	万 kW	23754.02	22343.40	6.31
核　电	万 kW	210.00	210.00	—

?1994-2018 China Academic Journal Electronic Publishing House. All rights reserved. http://www.cnki.net

续表

	单 位	2000年	1999年	比1999年增长(%)
**二、单机6000kW及以上机组**	**台**	**5235**	**5107**	**128**
	万kW	28288.29	26585.13	6.41
水 电	台	1062	984	78
	万kW	5747.13	5202.35	10.47
火 电	台	4170	4120	50
	万kW	22331.15	21172.77	5.47
其中：供热	台	1493	1313	180
	万kW	2986.41	2815.91	6.05
其中：国外机组	台	1051	1061	-10
	万kW	7773.75	7064.81	10.03
水 电	台	125	115	10
	万kW	1444.28	1274.78	13.30
火 电	台	924	944	-20
	万kW	6149.47	5610.04	9.62
平均单机容量	万kW/台	5.4	5.21	0.19
**三、35kV及以上输电线路长度**	**km**	**726167**	**686084**	**5.84**
其中：500kV	km	26837	22927	17.05
330kV	km	8669	7949	9.06
220kV	km	128114	121790	5.19
110kV	km	201230	190961	5.38
**四、35kV及以上变电设备容量**	**万kV·A**	**99612**	**91775**	**8.54**
其中：500kV	万kV·A	9447	8012	17.91
330kV	万kV·A	1410	1248	12.98
220kV	万kV·A	30632	28027	9.29
110kV	万kV·A	35384	32406	9.19
**五、发电量**	**亿kW·h**	**13684.82**	**12331.41**	**10.98**
水 电	亿kW·h	2431.34	2129.27	14.19
火 电	亿kW·h	11079.36	10047.37	10.27
核 电	亿kW·h	167.37	148.33	12.84
**六、6000kW及以上电厂供热量**	**万百万kJ**	**120434.27**	**108907**	**10.58**
**七、6000kW及以上电厂供电煤耗**	**g/(kW·h)**	**392**	**399**	**-7**
**八、6000kW及以上电厂发电煤耗**	**g/(kW·h)**	**363**	**369**	**-6**
**九、6000kW及以上电厂厂用电率**	**%**	**6.28**	**6.5**	**-0.22**
水 电	%	0.49	0.55	-0.06
火 电	%	7.31	7.51	-0.2
**十、6000kW及以上电厂利用小时**	**h**	**4517**	**4393**	**124**
水 电	h	3258	3198	60
火 电	h	4848	4719	129
**十一、供电量**	**亿kW·h**	**11365.77**	**10336.01**	**9.96**
售电量	亿kW·h	10490.4	9498.47	10.44
线损电量	亿kW·h	875.38	837.54	4.52
线路损失率	%	7.7	8.10	-0.4

?1994-2018 China Academic Journal Electronic Publishing House. All rights reserved. http://www.cnki.net

续表

	单 位	2000 年	1999 年	比 1999 年增长（%）
十二、6000kW 及以上电厂燃料消耗				
发电消耗标准煤量	万 t	39789.37	36539	8.89
发电消耗原煤量	万 t	52810.45	48187	9.60
发电消耗燃油量	万 t	1041.56	1159	-10.14
发电消耗燃气量	万 m^3	1538163	1290668	19.18
供热消耗标准煤量	万 t	4780.64	4489	6.50
供热消耗原煤量	万 t	6382.3	5689	12.18
供热消耗燃油量	万 t	175.6	201	-12.77
供热消耗燃气量	万 m^3	873543	965192	-9.50
十三、6000kW 及以上电厂热效率				
电厂发电热效率	%	33.84	33.46	0.38
电厂供热效率	%	85.96	82.78	3.18
能源转换总效率	%	39.43	38.85	0.58
十四、发用电设备比				
发电设备容量：用电设备容量		1:2.28	1:2.16	
十五、电力弹性系数				
电力生产弹性系数		1.37	0.92	0.45
电力消费弹性系数		1.42	0.92	0.5
十六、电力消费能源占一次能源的比重	%	41.72	40.07	1.65

2000 年电力工业建设项目投资完成情况

	计算单位	2000 年	1999 年	比 1999 年增长（%）
一、固定资产投资完成额	亿元	2125.53	1837.06	15.70
基本建设投资	亿元	953.66	1153.70	-17.34
“大代小”投资	亿元	43.39	33.77	28.49
城乡电网投资	亿元	1128.48	649.59	73.72
（一）基本建设投资完成额	亿元	953.67	1153.70	-17.34
1. 按资金来源分				
非经营基金	亿元	1.06	1.45	-26.90
开行软贷	亿元		12.85	
开行贷款	亿元	216.36	300.93	-28.10
商行贷款	亿元	170.81	198.36	-13.89
利用外资	亿元	210.81	220.55	-4.42
中央专项	亿元	25.66	25.55	0.43
三峡基金	亿元	14.77	9.20	60.54
煤代油	亿元		0.60	

?1994-2018 China Academic Journal Electronic Publishing House. All rights reserved. http://www.cnki.net

续表

	计算单位	2000年	1999年	比1999年增长(%)
企业自有	亿元	138.34	154.64	-10.54
中央债券	亿元	0.19		
地方债券	亿元		4.30	
地方专项	亿元	62.11	67.96	-8.61
地方其他	亿元	33.01	54.73	-39.69
其　他	亿元	80.55	102.58	-21.48
2.按类型分				
电　源	亿元	642.38	787.47	-18.42
电　网	亿元	260.08	300.98	-13.59
其　他	亿元	51.21	65.25	-21.52
3.按隶属关系分				
国家电力公司全资	亿元	327.76	375.85	-12.79
国家电力公司控股	亿元	308.50	378.69	-18.53
国家电力公司参股	亿元	158.69	222.79	-28.77
地方电力企业	亿元	112.33	97.71	14.96
其他电力企业	亿元	46.38	78.66	-41.04
4.按构成分				
建筑工程	亿元	246.75	314.00	-21.42
安装工程	亿元	134.67	148.22	-9.14
设备工器具购置	亿元	316.10	393.81	-19.73
其他工程	亿元	256.14	297.66	-13.95
**(二)"大代小"投资完成额**	**亿元**	**43.40**	**33.77**	**28.52**
1.按资金来源分				
开行贷款	亿元		2.29	
商行贷款	亿元	19.43	9.37	107.36
利用外资	亿元	4.19	5.75	-27.13
企业自有	亿元	7.27	11.22	-35.20
中央专项	亿元	0.20		
地方专项	亿元	0.81	0.45	80.00
地方其他	亿元	2.27	1.06	114.15
其　他	亿元	9.23	3.63	154.27
2.按构成分				
建筑工程	亿元	12.35	8.00	54.38
安装工程	亿元	5.65	6.69	-15.55
设备工器具购置	亿元	15.62	10.66	46.53
其他工程	亿元	8.78	8.42	4.28
**(三)城乡电网建设与改造投资完成额**	**亿元**	**1128.48**	**649.59**	**73.72**
1.按电网构成分				
城网部分	亿元	354.21	254.04	39.43
农网部分	亿元	774.27	395.55	95.75

?1994-2018 China Academic Journal Electronic Publishing House. All rights reserved. http://www.cnki.net

续表

	计算单位	2000年	1999年	比1999年增长(%)
其中：农网直供部分	亿元	376.57	226.93	65.94
农网趸售部分	亿元	397.60	168.62	135.86
**2. 按电压等级分**				
220kV	亿元	21.76	20.11	8.20
110kV	亿元	174.72	125.42	39.31
35kV	亿元	113.00	51.37	119.97
10kV及以下	亿元	749.45	412.19	81.82
其 他	亿元	69.56	40.50	71.75
(1) 城网部分				
220kV	亿元	20.34	18.55	9.65
110kV	亿元	91.07	74.43	22.36
35kV	亿元	23.87	15.40	55.00
10kV及以下	亿元	167.07	110.56	51.11
其 他	亿元	51.86	35.10	47.75
(2) 农网部分				
220kV	亿元	1.42	1.56	-8.97
110kV	亿元	83.64	50.99	64.03
35kV	亿元	89.12	35.98	147.69
10kV及以下	亿元	582.38	301.63	93.08
其 他	亿元	17.70	5.40	227.78
**二、年新增固定资产**	**亿元**	**865.42**	**1034.98**	**-16.38**
基本建设新增	亿元	838.22	969.10	-13.51
"大代小"新增	亿元	27.20	65.88	-58.71
**三、基建新增生产能力**				
**1. 新增单机500kW及以上机组**	**万kW**	**2012.04**	**2052.30**	**-1.96**
水 电	万kW	452.41	628.66	-28.04
火 电	万kW	1559.63	1423.64	9.55
其中：新增大中型机组	万kW	1934.03	1890.88	2.28
水 电	万kW	430.30	587.65	-26.78
火 电	万kW	1503.73	1303.23	15.38
**2. 新增110kV及以上线路**	**km**	**15778.27**	**13470.74**	**17.13**
500kV	km	6205.21	3057.62	102.94
330kV	km	706.00	757.35	-6.78
220kV	km	6585.26	6912.05	-4.73
110kV	km	2281.80	2743.72	-16.84
**3. 新增110kV及以上变电设备**	**万kV·A**	**4284.55**	**3935.40**	**8.87**
500kV	万kV·A	1974.40	1135.20	73.93
330kV	万kV·A	147.00	207.00	-28.99
220kV	万kV·A	2037.00	2278.20	-10.59
110kV	万kV·A	126.15	315.00	-59.95

?1994-2018 China Academic Journal Electronic Publishing House. All rights reserved. http://www.cnki.net

续表

	计算单位	2000年	1999年	比1999年增长(%)
**四、"大代小"投产能力**				
**1. 新增单机500kW及以上机组**	**万kW**	**40.90**	**132.20**	**-69.06**
火　电	万kW	40.90	132.20	-69.06
**2. 新增110kV及以上线路**	**km**	**0.80**		
220kV	km	0.80		
**3. 新增110kV及以上变电设备**	**万kV·A**	**18.00**		
220kV	万kV·A	18.00		
**五、城乡电网建设与改造投产能力**				
**1. 投产线路**	**km**	**1923711.57**	**758581.81**	**153.59**
220kV	km	1193.86	1028.34	16.10
110kV	km	11169.00	7580.65	47.34
35kV	km	24977.07	15464.76	61.51
1～10kV	km	458643.41	287569.90	59.49
低压线路	km	1427728.23	446938.16	219.45
**2. 投产变电设备**	**万kV·A**	**8791.34**	**6149.73**	**42.95**
220kV	万kV·A	336.00	551.30	-39.05
110kV	万kV·A	3420.71	2257.77	51.51
35kV	万kV·A	1852.43	877.15	111.19
1～10kV	万kV·A	3182.20	2463.51	29.17
**六、施工、竣工房屋建筑面积**				
**1. 施工房屋面积**	**万m^2**	**902.64**	**997.80**	**-9.54**
其中：住宅	万m^2	393.94	591.06	-33.35
**2. 竣工面积**	**万m^2**	**373.31**	**595.68**	**-37.33**
其中：住宅	万m^2	208.92	393.79	-46.95
**七、基本建设规模**				
**1. 上年结转规模**	**万kW**	**6273.04**	**7105.20**	**-11.71**
水　电	万kW	2600.19	3155.84	-17.61
火　电	万kW	3672.85	3949.36	-7.00
**2. 当年新开工规模**	**万kW**	**593.68**	**582.65**	**1.89**
水　电	万kW	95.70	46.95	103.83
火　电	万kW	497.98	535.70	-7.04
**3. 当年在建规模**	**万kW**	**7462.65**	**8163.92**	**-8.59**
水　电	万kW	3167.92	3187.84	-0.62
火　电	万kW	4294.73	4976.08	-13.69
**4. 当年投产规模**	**万kW**	**1934.03**	**1890.88**	**2.28**
水　电	万kW	430.30	587.65	-26.78
火　电	万kW	1503.73	1303.23	15.38
**5. 年末建设规模**	**万kW**	**5528.62**	**6273.04**	**-11.87**
水　电	万kW	2737.62	2600.19	5.29
火　电	万kW	2791.00	3672.85	-24.01
**6. 当年投产容量与建设规模比**		**1:3.9**	**1:4.3**	
水　电		1:7.4	1:5.4	
火　电		1:2.9	1:3.8	

注　在建规模中含三峡、万家寨、小浪底的规模。

?1994-2018 China Academic Journal Electronic Publishing House. All rights reserved.　http://www.cnki.net
