# Supplementary material for: Prediction analysis of carbon emission in China’s electricity industry based on the dual carbon background
Source: PLoS One. 2024 May 17;19(5):e0302068. doi: 10.1371/journal.pone.0302068 (PMC11101092; doi:10.1371/journal.pone.0302068)
Supplement: S3 File — (ZIP) [file pone.0302068.s003.zip › China Electric Power Yearbook 2001-2021/统计资料-2003.pdf]

国民经济主要指标

	单 位	2002 年	比 2001 年增长 (%)
全国总人口	万人	128453	0.65
城镇人口	万人	50212	4.47
乡村人口	万人	78241	-1.66
年末从业人员	万人	73740	0.98
国内生产总值	亿元	102398	8.00
第一产业	亿元	14883	2.90
第二产业	亿元	52982	9.90
第三产业	亿元	34533	7.30
工业增加值	亿元	45935	10.20
全社会固定资产投资总额	亿元	43202	16.10
国有经济投资总额	亿元	31020	17.00
基本建设投资	亿元	17251	16.40
更新改造投资	亿元	6584	11.10
房地产	亿元	7736	21.90
一次能源生产总量（标准煤）	亿 t	13.87	18.50
原 煤	万 t	13.80	18.90
原 油	万 t	1.67	1.80

注 国内生产总值、工业增加值绝对数按现价计算，增长速度按可比价格计算。

2002 年电力生产基本情况

	单 位	2002 年	2001 年	比 2001 年增长 (%)
**一、发电装机容量**	**万 kW**	**35657.09**	**33848.69**	**5.34**
水 电	万 kW	8607.46	8300.64	3.70
火 电	万 kW	26554.67	25301.2	4.95
核 电	万 kW	446.80	210.00	112.76
**二、单机 6000kW 及以上机组**	**台**	**6100**	**5860**	**240**
	万 kW	32570.57	30924.65	5.32
水 电	台	1424	1371	53
	万 kW	6458.22	6307.14	2.40

?1994-2018 China Academic Journal Electronic Publishing House. All rights reserved. http://www.cnki.net

续表

	单　位	2002年	2001年	比2001年增长(%)
火　电	台	4670	4486	184
	万kW	25665.55	24407.51	5.15
其中：供　热	台	1937	1827	110
	万kW	3743.67	3478.07	7.64
其中：国外机组	台	1125	1094	31
	万kW	9318.07	8569.66	8.73
水　电	台	151	144	7
	万kW	1624.92	1594.38	1.92
火　电	台	974	950	24
	万kW	7693.15	6975.28	10.29
平均单机容量	万kW/台	5.34	5.28	0.06
**三、35kV及以上输电线路长度**	**km**	**803505**	**781854**	**2.77**
其中：500kV	km	36745	31486	16.70
330kV	km	9612	9177	4.74
220kV	km	142362	135935	4.73
110kV	km	226567	220051	2.96
**四、35kV及以上变电设备容量**	**万kVA**	**124481**	**111771**	**11.37**
其中：500kV	万kVA	13750	11731	17.21
330kV	万kVA	1755	1527	14.93
220kV	万kVA	37209	34026	9.35
110kV	万kVA	44762	40238	11.24
**五、发电量**	**亿kWh**	**16541.64**	**14838.56**	**11.48**
水　电	亿kWh	2745.65	2611.08	5.15
火　电	亿kWh	13522.04	12044.78	12.26
核　电	亿kWh	264.89	174.72	51.61
**六、6000kW及以上电厂供热量**	**万百万kJ**	**139150.48**	**128743.69**	**8.08**
**七、6000kW及以上电厂供电煤耗**	**g/(kWh)**	**383**	**385**	**-2**
**八、6000kW及以上电厂发电煤耗**	**g/(kWh)**	**356**	**357**	**-1**
**九、6000kW及以上电厂厂用电率**	**%**	**6.15**	**6.24**	**-0.09**
水　电	%	0.49	0.46	0.03
火　电	%	7.10	7.25	-0.15
**十、6000kW及以上电厂利用小时**	**h**	**4860**	**4588**	**272**
水　电	h	3289	3129	160
火　电	h	5272	4900	372
**十一、供电量**	**亿kWh**	**14032.27**	**12556.45**	**11.75**
售电量	亿kWh	12976.89	11608.11	11.79
线损电量	亿kWh	1055.38	948.34	11.29
线路损失量	%	7.52	7.55	-0.03
**十二、6000kW及以上电厂燃料消耗**				
发电消耗标准煤量	万t	47290.08	42161.79	12.16
发电消耗燃煤量	万t	65594.55	57637.06	13.81

?1994-2018 China Academic Journal Electronic Publishing House. All rights reserved. http://www.cnki.net

续表

	单 位	2002 年	2001 年	比 2001 年增长 (%)
发电消耗燃油量	万 t	1089.12	1022.81	6.48
发电消耗燃气量	万 m^3	2114728	1811165	16.76
供热消耗标准煤量	万 t	5633.19	5160.31	9.16
供热消耗原煤量	万 t	7689.50	6924.35	11.05
供热消耗燃油量	万 t	153.75	148.27	3.70
供热消耗燃气量	万 m^3	1042125	915449	13.84
**十三、6000kW 及以上电厂热效率**				
电厂热效率	%	35.12	34.81	0.31
电厂供热效率	%	84.28	85.13	−0.85
能源转换总效率	%	40.36	40.55	−0.19
**十四、发用电设备比**				
发电设备容量：用电设备容量		1:2.68	1:2.46	
**十五、电力弹性系数**				
电力生产弹性系数		1.43	1.15	0.28
电力消费弹性系数		1.45	1.19	0.26
**十六、电力消费能源占一次能源的比重**	%	**43.56**	**42.90**	**0.66**

2002 年电力工业建设项目投资完成情况

	计算单位	2002 年	2001 年	比 2001 年增长 (%)
**一、固定资产投资完成额**	**亿元**	**2296.92**	**1944.55**	**18.12**
基本建设投资	亿元	1238.81	1010.71	22.57
"大代小"投资	亿元	70.31	58.85	19.47
城乡电网投资	亿元	987.80	874.99	12.89
**(一) 基本建设投资完成额**	**亿元**	**1238.81**	**1010.70**	**22.57**
**1. 按资金来源分**				
非经营基金	亿元	0.95	0.52	82.69
开行软贷	亿元	2.09	0.58	260.34

?1994-2018 China Academic Journal Electronic Publishing House. All rights reserved. http://www.cnki.net

续表

	计算单位	2002 年	2001 年	比 2001 年增长（%）
开行贷款	亿元	238.34	207.05	15.11
商行贷款	亿元	435.19	270.03	61.16
利用外资	亿元	172.46	172.43	0.02
中央专项	亿元	2.43	19.10	-87.28
三峡基金	亿元	16.81	36.94	-54.49
煤代油	亿元			
企业自有	亿元	179.53	161.13	11.42
中央债券	亿元	19.25	0.19	10031.58
地方债券	亿元			
地方专项	亿元	24.15	24.32	-0.70
地方其他	亿元	35.63	42.00	-15.17
其他	亿元	111.67	76.60	45.78
**2. 按类型分**				
电源	亿元	677.12	592.69	14.25
电网	亿元	519.68	361.20	43.88
其他	亿元	42.01	56.82	-26.06
**3. 按隶属关系分**				
国家电力公司全资	亿元	504.25	394.51	27.82
国家电力公司控股	亿元	261.36	326.65	-19.99
国家电力公司参股	亿元	232.64	193.14	20.45
地方电力企业	亿元	32.22	67.29	-52.12
其他电力企业	亿元	208.34	29.12	615.45
**4. 按构成分**				
建筑工程	亿元	398.72	289.94	37.52
安装工程	亿元	161.92	146.03	10.88
设备工器具购置	亿元	381.50	324.72	17.49
其他工程	亿元	296.67	250.02	18.66
**（二）"大代小"投资完成额**	**亿元**	**70.31**	**58.85**	**19.47**
**1. 按资金来源分**				
开行贷款	亿元	13.24	3.36	294.05

?1994-2018 China Academic Journal Electronic Publishing House. All rights reserved. http://www.cnki.net

续表

	计算单位	2002 年	2001 年	比 2001 年增长 (%)
商行贷款	亿元	23.19	38.98	-40.51
利用外资	亿元	1.08	2.52	-57.14
企业自有	亿元	13.65	7.35	85.71
中央专项	亿元			
地方专项	亿元	3.88		
地方其他	亿元	1.42	4.85	-70.72
其 他	亿元	13.87	1.79	674.86
**2. 按构成分**				
建筑工程	亿元	13.91	11.91	16.79
安装工程	亿元	13.77	11.60	18.71
设备工器具购置	亿元	29.43	24.98	17.81
其他工程	亿元	13.19	10.36	27.32
**(三) 城乡电网建设与改造投资完成额**	**亿元**	**987.80**	**874.99**	**12.89**
**按电网构成分**				
城网部分	亿元	173.86	372.52	-53.33
农网部分	亿元	813.94	502.47	61.99
其中：农网直供部分	亿元	454.99	254.99	78.43
农网趸售部分	亿元	358.95	247.48	45.04
**二、年新增固定资产**	**亿元**	**911.92**	**898.10**	**1.54**
基本建设新增	亿元	833.05	841.24	-0.97
“大代小”新增	亿元	78.87	56.86	38.71
**三、基建新增生产能力**				
**1. 新增单机 500kW 及以上机组**	**万 kW**	**1193.01**	**1586.87**	**-24.82**
水 电	万 kW	148.22	289.55	-48.81
火 电	万 kW	1044.79	1297.32	-19.47
其中：新增大中型机组	万 kW	1130.36	1516.36	-25.45
水 电	万 kW	123.62	266.51	-53.62
火 电	万 kW	1006.74	1249.85	-19.45
**2. 新增 110kV 及以上线路**	**km**	**17797.95**	**14302.46**	**24.44**
500kV	km	5728.14	4335.01	32.14

?1994-2018 China Academic Journal Electronic Publishing House. All rights reserved. http://www.cnki.net

续表

	计算单位	2002 年	2001 年	比 2001 年增长（%）
330kV	km	537.12	418.00	28.50
220kV	km	7385.10	7178.08	2.88
110kV	km	4147.59	2371.37	74.90
**3. 新增 110kV 及以上变电设备**	**万 kVA**	**5872.00**	**5319.30**	**10.39**
500kV	万 kVA	2233.00	2133.00	4.69
330kV	万 kVA	129.00	153.00	-15.69
220kV	万 kVA	2596.00	2621.00	-0.95
110kV	万 kVA	914.00	412.30	121.68
**四、"大代小"投产能力**				
**新增单机 500kW 及以上机组**	**万 kW**	**240.25**	**132.20**	**81.73**
火　电	万 kW	240.25	131.50	82.70
**五、城乡电网建设与改造投产能力**				
**1. 投产线路**	**km**	**2069595.59**	**1452278.93**	**42.51**
220kV	km	862.01	1294.76	-33.42
110kV	km	10998.62	16093.12	-31.66
35kV	km	26536.79	34203.81	-22.42
1～10kV	km	608380.16	331669.73	83.43
低压线路	km	1422818.01	1069017.51	33.10
**2. 投产变电设备**	**万 kVA**	**22660.65**	**12643.06**	**79.23**
220kV	万 kVA	824.10	1197.71	-31.19
110kV	万 kVA	3338.40	4627.26	-27.85
35kV	万 kVA	1734.87	2454.81	-29.33
1～10kV	万 kVA	16763.28	4363.28	284.19
**六、基本建设规模**				
**1. 上年结转规模**	**万 kW**	**6354.06**	**5528.62**	**14.93**
水　电	万 kW	3585.01	2737.62	30.95
火　电	万 kW	2769.05	2791.00	-0.79
**2. 当年新开工规模**	**万 kW**	**2077.50**	**2058.94**	**0.90**
水　电	万 kW	963.50	963.40	0.01
火　电	万 kW	1114.00	1095.54	1.69
**3. 当年在建规模**	**万 kW**	**8431.56**	**7870.42**	**7.13**

?1994-2018 China Academic Journal Electronic Publishing House. All rights reserved. http://www.cnki.net
