# Supplementary material for: Prediction analysis of carbon emission in China’s electricity industry based on the dual carbon background
Source: PLoS One. 2024 May 17;19(5):e0302068. doi: 10.1371/journal.pone.0302068 (PMC11101092; doi:10.1371/journal.pone.0302068)
Supplement: S3 File — (ZIP) [file pone.0302068.s003.zip › China Electric Power Yearbook 2001-2021/统计资料-2005.pdf]

# 电力行业统计资料

## 2004年发电技术经济指标

| 地区 | 发电设备平均利用小时（h） | | | 发电厂用电率（%） | | | 标准煤耗（g/kWh） | |
|---|---|---|---|---|---|---|---|---|
| | 合计 | 水电 | 火电 | 合计 | 水电 | 火电 | 发电 | 供电 |
| 全国总计 | 5455 | 3462 | 5991 | 5.95 | 0.47 | 6.85 | 349 | 376 |
| 北京市 | 5379 | 330 | 7322 | 7.84 | 1.93 | 7.94 | 317 | 348 |
| 天津市 | 5656 | | 5656 | 6.35 | | 6.35 | 323 | 344 |
| 河北省 | 6086 | 444 | 6350 | 6.49 | 2.70 | 6.50 | 350 | 375 |
| 山西省 | 6450 | 2667 | 6622 | 7.57 | 0.42 | 7.70 | 361 | 391 |
| 内蒙古自治区 | 6436 | 3939 | 6712 | 7.08 | 0.91 | 7.17 | 336 | 369 |
| 辽宁省 | 5460 | 2931 | 5715 | 6.94 | 1.33 | 7.21 | 349 | 376 |
| 吉林省 | 4147 | 1655 | 5633 | 6.64 | 0.75 | 7.68 | 362 | 392 |
| 黑龙江省 | 4612 | 1528 | 4837 | 7.69 | 1.27 | 7.84 | 376 | 408 |
| 上海市 | 6243 | | 6243 | 5.22 | | 5.22 | 329 | 347 |
| 江苏省 | 6385 | 2242 | 6402 | 5.92 | 1.03 | 5.93 | 346 | 368 |
| 浙江省 | 5701 | 1284 | 6923 | 5.69 | 0.62 | 5.68 | 340 | 361 |
| 安徽省 | 6155 | 1684 | 6450 | 5.94 | 0.23 | 6.03 | 341 | 369 |
| 福建省 | 4768 | 2093 | 6469 | 5.09 | 0.30 | 6.07 | 334 | 359 |
| 江西省 | 4637 | 1506 | 5634 | 6.58 | 1.20 | 7.04 | 353 | 380 |
| 山东省 | 5145 | | 5148 | 7.32 | | 7.32 | 337 | 364 |
| 河南省 | 5487 | 2862 | 5819 | 7.72 | 0.43 | 8.19 | 411 | 377 |
| 湖北省 | 5228 | 5407 | 4968 | 2.51 | 0.12 | 6.58 | 345 | 376 |
| 湖南省 | 4761 | 3620 | 5778 | 4.98 | 0.51 | 7.47 | 362 | 391 |
| 广东省 | 5503 | 2026 | 5908 | 5.00 | 0.60 | 5.42 | 336 | 358 |
| 广西自治区 | 4894 | 3914 | 6104 | 4.53 | 0.43 | 8.33 | 361 | 394 |
| 海南省 | 3623 | 1887 | 4155 | 5.62 | 1.06 | 6.24 | 334 | 358 |
| 重庆市 | 5065 | 4909 | 5123 | 8.70 | 2.09 | 11.06 | 386 | 434 |
| 四川省 | 4946 | 4671 | 5412 | 4.05 | 0.39 | 9.41 | 407 | 455 |
| 贵州省 | 5403 | 3419 | 6970 | 5.17 | 0.30 | 7.06 | 339 | 370 |
| 云南省 | 5153 | 4428 | 6238 | 3.82 | 0.30 | 7.56 | 368 | 398 |
| 西藏自治区 | 2908 | 3034 | | 4.91 | 2.52 | 22.57 | | |
| 陕西省 | 5515 | 2607 | 6087 | 7.01 | 0.60 | 7.50 | 358 | 387 |
| 甘肃省 | 5520 | 3449 | 7126 | 4.73 | 0.70 | 6.21 | 348 | 368 |
| 青海省 | 3804 | 3109 | 6124 | 3.61 | 1.05 | 7.96 | 396 | 430 |
| 宁夏自治区 | 7276 | 3211 | 7732 | 5.25 | 0.41 | 5.45 | 336 | 358 |
| 新疆自治区 | 4420 | 3772 | 4588 | 8.03 | 1.50 | 9.07 | 416 | 460 |

?1994-2018 China Academic Journal Electronic Publishing House. All rights reserved. http://www.cnki.net

# 2004年全国分地区发电设备容量

| 地区 | 装机容量（万kW） | | | | | 比2003年增减（%） | | | | |
|---|---|---|---|---|---|---|---|---|---|---|
| | 合计 | 水电 | 火电 | 核电 | 其他 | 合计 | 水电 | 火电 | 核电 | 其他 |
| 全国总计 | 44238.73 | 10524.16 | 32948.30 | 683.60 | 81.97 | 13.02 | 10.90 | 13.70 | 10.51 | 47.83 |
| 北京市 | 451.44 | 105.59 | 345.85 | | | 2.47 | −0.21 | 3.32 | | |
| 天津市 | 601.35 | 0.50 | 600.85 | | | 0.00 | 0.00 | 0.00 | | |
| 河北省 | 2073.00 | 78.38 | 1993.27 | | 1.35 | 12.20 | 2.55 | 12.62 | | |
| 山西省 | 1848.05 | 78.73 | 1769.33 | | | 16.73 | −1.06 | 17.67 | | |
| 内蒙古自治区 | 1432.12 | 56.79 | 1364.15 | | 11.17 | 18.45 | −4.08 | 19.43 | | 45.87 |
| 辽宁省 | 1650.64 | 140.41 | 1496.03 | | 14.20 | 0.96 | 0.57 | 0.97 | | 3.26 |
| 吉林省 | 959.59 | 360.12 | 595.87 | | 3.61 | 1.99 | 0.43 | 2.87 | | 19.96 |
| 黑龙江省 | 1214.30 | 84.46 | 1125.91 | | 3.93 | 2.14 | 1.20 | 1.85 | | |
| 上海市 | 1201.83 | 0.00 | 1201.49 | | 0.34 | 8.34 | | 8.31 | | |
| 江苏省 | 2843.36 | 12.65 | 2828.95 | | 1.75 | 27.03 | −8.17 | 27.17 | | |
| 浙江省 | 3095.39 | 641.84 | 2143.98 | 305.60 | 3.97 | 29.94 | 6.01 | 39.94 | 27.02 | −0.13 |
| 安徽省 | 1005.73 | 69.28 | 936.45 | | | 1.24 | 6.73 | 0.86 | | |
| 福建省 | 1550.75 | 718.01 | 831.54 | | 1.20 | 11.84 | 6.20 | 17.24 | | |
| 江西省 | 804.59 | 254.99 | 549.60 | | | 4.29 | 10.51 | 1.63 | | |
| 山东省 | 3292.36 | 5.08 | 3286.04 | | 1.23 | 7.79 | 0.09 | 7.76 | | |
| 河南省 | 2422.65 | 243.80 | 2178.85 | | | 20.69 | 0.00 | 23.55 | | |
| 湖北省 | 2462.44 | 1511.51 | 950.93 | | | 24.93 | 31.01 | 16.35 | | |
| 湖南省 | 1422.78 | 744.82 | 677.95 | | | 9.03 | 12.80 | 5.16 | | |
| 广东省 | 4262.10 | 858.46 | 3017.29 | 378.00 | 8.34 | 8.72 | 5.89 | 10.80 | | 0.05 |
| 广西自治区 | 941.85 | 504.04 | 437.81 | | | 22.08 | 11.39 | 37.24 | | |
| 海南省 | 217.04 | 56.22 | 159.95 | | 0.87 | 23.35 | 1.97 | 33.35 | | |
| 重庆市 | 467.90 | 140.79 | 327.11 | | | 5.00 | 5.87 | 4.64 | | |
| 四川省 | 2028.32 | 1338.29 | 690.03 | | | 9.96 | 8.44 | 13.05 | | |
| 贵州省 | 1469.83 | 689.65 | 780.18 | | | 15.74 | 10.63 | 20.66 | | |
| 云南省 | 1136.55 | 705.86 | 430.69 | | | 12.53 | 7.88 | 21.09 | | |
| 西藏自治区 | 46.92 | 40.35 | 3.46 | | 2.42 | 24.72 | 29.58 | 0.00 | | |
| 陕西省 | 951.69 | 187.65 | 764.04 | | | 8.29 | 28.32 | 4.29 | | |
| 甘肃省 | 867.99 | 356.61 | 497.56 | | 13.82 | 7.86 | 8.70 | 4.86 | | |
| 青海省 | 494.32 | 405.34 | 88.98 | | | 16.39 | 21.32 | −1.77 | | |
| 宁夏自治区 | 419.07 | 36.62 | 378.20 | | 4.25 | 22.53 | 18.82 | 21.92 | | |
| 新疆自治区 | 602.80 | 97.30 | 495.97 | | 9.53 | 9.70 | −1.70 | 12.38 | | 4.38 |

?1994-2018 China Academic Journal Electronic Publishing House. All rights reserved. http://www.cnki.net

# 2004 年全国分地区发电量

| 地　　区 | 发电量（亿 kWh） | | | | | 比 2003 年增减（%） | | | | |
|---|---|---|---|---|---|---|---|---|---|---|
| | 合计 | 水电 | 火电 | 核电 | 其他 | 合计 | 水电 | 火电 | 核电 | 其他 |
| 全国总计 | 21943.52 | 3309.90 | 18103.80 | 504.69 | 25.13 | 15.18 | 17.65 | 14.66 | 15.08 | 137.84 |
| 北京市 | 189.26 | 3.47 | 185.79 | | | −1.87 | −48.84 | −0.16 | | |
| 天津市 | 339.52 | 0.00 | 339.52 | | | 5.44 | −100.00 | 5.47 | | |
| 河北省 | 1255.35 | 5.25 | 1249.70 | | 0.40 | 15.38 | 4.08 | 15.43 | | 8.43 |
| 山西省 | 1069.58 | 20.32 | 1049.26 | | | 11.59 | 7.50 | 11.67 | | |
| 内蒙古自治区 | 814.58 | 8.13 | 804.27 | | 2.18 | 23.52 | 16.63 | 23.53 | | 51.66 |
| 辽宁省 | 887.54 | 39.47 | 845.43 | | 2.64 | 7.79 | 65.65 | 6.01 | | 30.48 |
| 吉林省 | 394.70 | 61.47 | 332.42 | | 0.81 | 16.49 | 50.67 | 11.78 | | |
| 黑龙江省 | 548.66 | 13.38 | 534.82 | | 0.46 | 10.62 | 21.13 | 10.29 | | |
| 上海市 | 711.34 | 0.00 | 711.27 | | 0.07 | 2.43 | | 2.42 | | |
| 江苏省 | 1639.01 | 3.27 | 1635.45 | | 0.29 | 22.61 | −18.24 | 22.71 | | |
| 浙江省 | 1258.83 | 85.45 | 952.55 | 219.88 | 0.95 | 15.26 | −23.18 | 14.64 | 47.34 | 11.29 |
| 安徽省 | 611.02 | 12.27 | 598.75 | | | 9.67 | −21.37 | 10.56 | | |
| 福建省 | 659.66 | 154.57 | 504.90 | | 0.19 | 8.01 | −18.21 | 19.80 | | −20.21 |
| 江西省 | 340.17 | 38.90 | 301.27 | | | 9.63 | 0.67 | 10.90 | | |
| 山东省 | 1639.75 | 0.41 | 1639.18 | | 0.16 | 17.49 | 114.95 | 17.46 | | |
| 河南省 | 1162.36 | 68.84 | 1093.52 | | | 15.11 | 26.14 | 14.48 | | |
| 湖北省 | 1125.46 | 695.12 | 430.34 | | | 43.72 | 79.27 | 8.86 | | |
| 湖南省 | 614.23 | 242.36 | 371.86 | | | 13.95 | −0.67 | 26.05 | | |
| 广东省 | 2121.33 | 141.14 | 1693.89 | 284.81 | 1.49 | 11.90 | −17.64 | 18.16 | −1.55 | |
| 广西自治区 | 373.72 | 172.29 | 201.43 | | | 2.76 | −10.68 | 17.94 | | |
| 海南省 | 68.74 | 11.77 | 56.87 | | 0.10 | 15.69 | −18.88 | 26.97 | | −18.71 |
| 重庆市 | 229.14 | 56.70 | 165.20 | | 7.25 | 12.92 | 43.50 | 1.09 | | |
| 四川省 | 935.29 | 589.02 | 346.27 | | | 12.98 | 17.80 | 5.63 | | |
| 贵州省 | 731.00 | 233.79 | 497.20 | | | 15.47 | 16.84 | 14.84 | | |
| 云南省 | 536.72 | 293.50 | 243.22 | | | 16.96 | 9.37 | 27.64 | | |
| 西藏自治区 | 12.06 | 10.88 | 0.06 | | 1.12 | 19.99 | 19.21 | 11.02 | | |
| 陕西省 | 514.81 | 70.43 | 444.39 | | | 20.55 | 54.44 | 16.50 | | |
| 甘肃省 | 475.26 | 120.47 | 332.42 | | 4.38 | 16.23 | 22.77 | 12.71 | | |
| 青海省 | 172.78 | 110.71 | 62.08 | | | 27.22 | 55.14 | −3.70 | | |
| 宁夏自治区 | 263.27 | 9.84 | 252.98 | | 0.46 | 31.64 | 19.68 | 31.93 | | |
| 新疆自治区 | 266.41 | 36.68 | 227.52 | | 2.21 | 12.84 | 2.77 | 14.71 | | 6.20 |

?1994-2018 China Academic Journal Electronic Publishing House. All rights reserved. http://www.cnki.net
