# Supplementary material for: Prediction analysis of carbon emission in China’s electricity industry based on the dual carbon background
Source: PLoS One. 2024 May 17;19(5):e0302068. doi: 10.1371/journal.pone.0302068 (PMC11101092; doi:10.1371/journal.pone.0302068)
Supplement: S3 File — (ZIP) [file pone.0302068.s003.zip › China Electric Power Yearbook 2001-2021/统计资料-2007.pdf]

电力行业统计资料

2006年全国各省（市、自治区）发电设备容量

单位：万 kW

地区	全部		水电		火电		核电		风电		其他	
	2006	同比(%)	2006	同比(%)	2006	同比(%)	2006	同比(%)	2006	同比(%)	2006	同比(%)
全国总计	62369.8	20.6	13029.2	11.0	48382.2	23.6	684.6		207.2	96.3	66.5	28.1
北京市	506.1	3.1	105.3		398.4	3.9					2.4	
天津市	654.1	5.9	0.5		651.2	5.5					2.4	
河北省	2709.0	16.9	78.5	0.1	2608.7	16.8			21.8	354.1		
山西省	2745.1	19.0	79.0	0.9	2666.1	19.6						
内蒙古自治区	3028.1	51.8	81.8	44.0	2889.9	50.7			54.8	162.2	1.7	
辽宁省	1833.8	4.6	140.1	−0.2	1672.1	4.5			20.7	63.4	0.9	
吉林省	1113.1	9.5	387.2	4.1	703.9	10.7			20.8	162.4	1.3	108.3
黑龙江省	1342.5	7.6	85.3	0.8	1245.6	7.6			11.5	119.7		
上海市	1478.0	10.6	0.0		1452.6	10.8			2.4		22.9	
江苏省	5207.3	31.9	13.6	−4.9	5177.6	21.8			1.5		14.7	149.4
浙江省	4687.0	34.2	836.9	20.4	3539.1	27.9	306.6		3.7		0.6	
安徽省	1513.5	23.6	100.1	35.4	1413.4	22.8						
福建省	2204.7	25.1	895.7	8.9	1300.1	39.1			8.9	70.8		
江西省	985.6	10.4	328.8	8.9	656.8	11.2						
山东省	5005.5	33.7	55.3	973.4	4939.5	32.3			10.6	191.2		
河南省	3515.6	22.0	255.3	0.5	3260.3	24.1						
湖北省	2994.4	9.2	1832.1	2.4	1162.3	22.0						
湖南省	1938.0	28.7	864.8	10.2	1071.5	48.6					1.7	
广东省	5389.9	12.1	932.0	3.1	4061.5	15.4	378		18.3	119.9		
广西自治区	1305.8	18.5	762.4	25.3	543.4	10.2						
海南省	258.0	22.0	58.9	2.6	198.2	29.4			0.9			
重庆市	759.7	33.8	197.9	3.5	559.4	49.6					2.4	
四川省	2728.5	31.0	1773.0	18.0	955.5	27.1						
贵州省	2188.3	29.7	753.4	4.2	1435.0	48.9						
云南省	1826.1	43.2	969.8	21.2	856.4	80.2						
西藏自治区	37.9	−21.4	34.3	−18.2	1.2	−63.3					2.4	−20.3
陕西省	1193.4	8.3	216.5	9.5	972.3	8.1					4.6	
甘肃省	1093.9	11.0	429.1	7.1	644.8	12.9			11.3	117.2	8.6	
青海省	694.0	21.5	542.3	12.4	151.7	71.0						
宁夏自治区	644.1	24.3	42.9	−0.2	600.2	29.3			1.1	−90.6		
新疆自治区	789.2	20.7	176.6	29.9	593.7	17.6			18.9	41.5		

?1994-2018 China Academic Journal Electronic Publishing House. All rights reserved.   http://www.cnki.net

2006年全国各省（市、自治区）发电量

单位：亿kWh

地　　区	发电量	增长率（%）
全国总计	28598	14.1
北京市	211	−1.2
天津市	363	−2.6
河北省	1461	9.1
山西省	1526	16.3
内蒙古自治区	1416	34.0
辽宁省	1011	11.8
吉林省	456	5.1
黑龙江省	646	6.5
上海市	727	−2.0
江苏省	2536	19.6
浙江省	1766	21.3
安徽省	734	13.1
福建省	904	16.2
江西省	436	16.7
山东省	2273	18.9
河南省	1583	14.5
湖北省	1308	1.4
湖南省	748	16.1
广东省	2472	8.5
广西自治区	523	17.3
海南省	95	15.3
重庆市	289	13.8
四川省	1227	20.4
贵州省	980	22.9
云南省	757	25.2
西藏自治区	13	−5.7
陕西省	582	6.0
甘肃省	529	4.6
青海省	280	25.9
宁夏自治区	391	26.6
新疆自治区	356	14.8

2006年全国各省（市、自治区）发电设备利用小时

地　　区	合计（h）	水电（h）	火电（h）
全国总计	5198	3393	5612
北京市	4039	404	4924
天津市	5843		5843
河北省	5939	666	6099
山西省	6275	3186	6369
内蒙古自治区	5785	2261	5916
辽宁省	5746	3362	5978
吉林省	4360	1321	6151
黑龙江省	5147	1648	5404
上海市	5102		5079
江苏省	5398	1926	5373
浙江省	5339	1765	5961
安徽省	5279	1240	5483
福建省	4669	4015	5042
江西省	4797	2730	5415
山东省	5324	94	5364
河南省	5152	3129	5331
湖北省	4774	4245	5712
湖南省	4367	3313	5117
广东省	5121	2727	5161
广西自治区	4577	3691	5643
海南省	3936	2192	4303
重庆市	4729	2799	5341
四川省	4409	4105	4901
贵州省	5342	1669	6660
云南省	4942	3871	6275
西藏自治区	3547	3531	
陕西省	4924	2221	5356
甘肃省	5202	4089	6021
青海省	4198	3760	6219
宁夏自治区	7099	3892	7499
新疆自治区	4939	3120	5578

?1994-2018 China Academic Journal Electronic Publishing House. All rights reserved.   http://www.cnki.net

2006年全国各省（市、自治区）厂用电率情况

地　区	厂用电率（%）
全国总计	5.93
北京市	7.51
天津市	6.86
河北省	6.63
山西省	7.45
内蒙古自治区	7.58
辽宁省	6.62
吉林省	6.78
黑龙江省	7.85
上海市	5.06
江苏省	5.69
浙江省	5.62
安徽省	6.05
福建省	4.51
江西省	6.17
山东省	7.12
河南省	7.06
湖北省	2.75
湖南省	4.95
广东省	5.27
广西自治区	4.45
海南省	7.56
重庆市	8.45
四川省	4.51
贵州省	6.06
云南省	4.12
西藏自治区	
陕西省	6.97
甘肃省	4.29
青海省	2.57
宁夏自治区	
新疆自治区	8.02

2006年全国各省（市、自治区）煤耗情况

地　区	发电（g/kWh）	供电（g/kWh）
全国总计	342	367
北京市	303	333
天津市	319	342
河北省	345	370
山西省	344	371
内蒙古自治区	338	369
辽宁省	346	371
吉林省	353	382
黑龙江省	362	393
上海市	321	338
江苏省	334	354
浙江省	327	347
安徽省	335	357
福建省	328	352
江西省	350	380
山东省	355	379
河南省	348	377
湖北省	339	363
湖南省	349	375
广东省	332	352
广西自治区	345	374
海南省	296	324
重庆市	364	403
四川省	387	426
贵州省	343	368
云南省	348	373
西藏自治区		
陕西省	342	368
甘肃省	340	361
青海省	387	418
宁夏自治区	337	365
新疆自治区	425	469

?1994-2018 China Academic Journal Electronic Publishing House. All rights reserved. http://www.cnki.net

2006年全国各省（市、自治区）供热情况

地　区	供热容量（万kW）	供热量（GJ）
全国总计	8311	2275657476
北京市	304	60700224
天津市	182	58384021
河北省	616	136001157
山西省	354	55120078
内蒙古自治区	405	57091587
辽宁省	563	209382110
吉林省	404	114942841
黑龙江省	536	110995803
上海市	366	56156945
江苏省	1270	381183009
浙江省	458	313650852
安徽省	132	41598982
福建省	59	24312497
江西省		
山东省	1467	356367894
河南省	401	50894701
湖北省	113	3487398
湖南省	45	42706488
广东省	175	47862012
广西自治区		
海南省		
重庆市	16	23271522
四川省	31	36463934
贵州省		
云南省		
西藏自治区		
陕西省	72	15427858
甘肃省	122	38192041
青海省		
宁夏自治区	47	3518968
新疆自治区	173	37944554

2006年全国各省（市、自治区）全社会用电量情况

地　区	全年累计（万kWh）	同比（%）
全国合计	283678849	14.16
北京市	6115719	7.19
天津市	4336501	12.68
河北省	17348320	15.51
山西省	10976771	15.99
内蒙古自治区	8849083	32.53
辽宁省	12282742	10.60
吉林省	4124577	9.05
黑龙江	5970466	7.41
上海市	9901450	7.31
江苏省	25697523	17.16
浙江省	19092315	16.25
安徽省	6621832	13.75
福建省	8668444	14.57
江西省	4461968	13.83
山东省	22720720	15.24
河南省	15235026	12.63
湖北省	8767570	11.14
湖南省	7687729	13.99
广东省	30040334	12.36
广西区	5794618	13.59
海南省	976759	20.06
重庆市	4051961	15.25
四川省	10594386	12.40
贵州省	5819780	16.09
云南省	6456136	15.87
西藏自治区	130845	11.69
陕西省	5807287	12.36
甘肃省	5363349	9.57
青海省	2444149	18.33
宁夏自治区	3778500	24.75
新疆自治区	3561989	14.85

?1994-2018 China Academic Journal Electronic Publishing House. All rights reserved. http://www.cnki.net
