# Supplementary material for: Prediction analysis of carbon emission in China’s electricity industry based on the dual carbon background
Source: PLoS One. 2024 May 17;19(5):e0302068. doi: 10.1371/journal.pone.0302068 (PMC11101092; doi:10.1371/journal.pone.0302068)
Supplement: S3 File — (ZIP) [file pone.0302068.s003.zip › China Electric Power Yearbook 2001-2021/统计资料-2009.pdf]

电力行业统计资料

2008年全国分地区发电设备容量

装机容量（万 kW）

地区	全部			水电			火电			核电			风电			其他
	2008年	2007年	同比(%)	2008年	2007年	同比(%)	2008年	2007年	同比(%)	2008年	2007年	同比(%)	2008年	2007年	同比(%)	2008年
**全国**	**79 273**	**71 822**	**10.4**	**17 260**	**14 823**	**16.4**	**60 286**	**55 607**	**8.4**	**885**	**885**		**839**	**420**	**99.8**	**3.53**
北京市	581	495	17.4	105	105		476	390	22.1							
天津市	749	693	8.0		1		749	692	8.1							
河北省	3211	3021	6.3	154	78	96.7	2987	2902	2.9				70	41	73.1	
山西省	3604	3174	13.5	79	79	−0.1	3525	3095	13.9							
内蒙古	4886	4179	16.9	83	83		4574	3987	14.7				230	108	113.1	
辽宁省	2219	2150	3.2	143	141	1.1	1990	1972	0.9				85	35	143.6	0.90
吉林省	1300	1198	8.5	389	389	0.2	835	758	10.1				76	47	62.1	
黑龙江省	1813	1518	19.4	94	87	8.3	1657	1408	17.7				62	23	168.2	
上海市	1682	1442	16.6				1678	1415	18.6				4	2	61.5	0.22
江苏省	5442	5599	−2.8	114	14		5068	5334	−5.0	200	200		61	25	145.9	
浙江省	5317	5112	4.0	896	852	5.2	4099	3949	3.8	307	307		15	4	286.5	
安徽省	2638	1927	36.9	156	151	3.4	2482	1776	39.7							
福建省	2627	2398	9.5	1058	980	7.9	1543	1391	10.9				26	23	12.5	
江西省	1308	1284	1.8	371	357	4.0	934	927	0.7				3			
山东省	5736	5540	3.5	105	105		5593	5414	3.3				37	21	71.7	
河南省	4572	4129	10.7	302	274	10.1	4268	3854	10.7				3			
湖北省	4328	3708	16.7	2905	2402	20.9	1421	1304	8.9				1	1		
湖南省	2508	2260	10.9	1065	922	15.4	1443	1336	8.0							
广东省	6008	5886	2.1	1028	1011	1.7	4573	4471	2.3	378	378		29	25	16.2	
广西区	2424	1975	22.7	1397	1044	33.9	1027	931	10.3							
海南省	279	302	−7.5	41	59	−30.3	237	240	−1.4				1	1		
重庆市	1073	863	24.3	406	224	81.6	666	637	4.6							
四川省	3501	3186	9.9	2224	1986	12.0	1277	1200	6.4							
贵州省	2664	2417	10.2	947	821	15.3	1717	1596	7.6							
云南省	2585	2221	16.4	1574	1158	35.8	1003	1063	−5.6				8			
西藏区	54	42	27.5	43	39	12.6	8	1								2.41
陕西省	1966	1416	38.9	181	179	1.1	1785	1229	45.2							
甘肃省	1502	1259	19.3	544	440	23.6	898	784	14.5				60	26	129.8	
青海省	791	774	2.2	591	583	1.3	200	190	5.2							
宁夏区	814	751	8.5	43	43		754	703	7.3				17	5	248.5	
新疆区	1090	903	20.7	219	214	2.1	820	656	25.0				51	33	57.1	

注 表中其他部分的统计口径与2007年不同。

?1994-2018 China Academic Journal Electronic Publishing House. All rights reserved. http://www.cnki.net

2008 年全国分地区发电量

地区	全部			水电			火电			核电			风电			其他
	2008年	2007年	同比(%)	2008年	2007年	同比(%)	2008年	2007年	同比(%)	2008年	2007年	同比(%)	2008年	2007年	同比(%)	2008年
**全国**	**34 510**	**32 644**	**5.7**	**5655**	**4714**	**20.0**	**28 030**	**27 207**	**3.0**	**692**	**629**	**10.1**	**130.8**	**57.1**	**129.0**	**1.7**
北京市	247	227	9.0	5	4	5.5	243	223	9.1							
天津市	397	399	−0.4		0.1		397	399	−0.4							
河北省	1601	1646	−2.7	7	6	19.6	1580	1633	−3.2				13.6	7.1	90.4	
山西省	1786	1759	1.5	23	26	−10.0	1762	1734	1.7							
内蒙区	2057	1830	12.4	11	14	−21.9	2008	1801	11.5				37.4	14.4	160.5	
辽宁省	1139	1113	2.4	42	44	−3.2	1085	1065	1.9				10.8	3.3	231.5	0.2
吉林省	526	501	4.9	48	56	−14.5	464	437	6.2				13.7	6.4	113.1	
黑龙江省	739	699	5.8	14	12	16.2	715	684	4.6				10.9	3.6	203.4	
上海市	795	742	7.2				794	726	9.4				0.6	0.4	39.8	0.01
江苏省	2887	2825	2.2	3.3	3.2	3.4	2735	2709	1.0	141	100	40.5	7.8	2.1	266.0	
浙江省	2134	2080	2.6	147	130	12.7	1748	1723	1.4	238	227	5.1	1.3	0.5	162.2	
安徽省	1103	868	27.0	28	20	41.0	1074	848	26.6							
福建省	1085	1039	4.4	332	312	6.4	748	723	3.4				5.9	4.0	45.2	
江西省	494	494	−0.1	89	73	21.5	405	421	−3.9				0.01			
山东省	2697	2596	3.9	2.0	2.0	13.7	2689	2591	3.8				5.4	2.7	104.2	
河南省	1972	1864	5.8	81	91	−10.4	1890	1773	6.6				0.2			
湖北省	1752	1541	13.7	1199	933	28.6	553	609	−9.2				0.2	0.04	425.8	
湖南省	850	837	1.5	312	294	6.1	537	542	−0.8				0.01			
广东省	2682	2695	−0.5	256	232	10.0	2107	2157	−2.3	313	302	3.8	6.0	3.9	55.9	
广西区	855	685	24.9	513	324	58.4	342	361	−5.2							
海南省	118	114	3.5	11	12	−11.2	107	101	6.1				0.14	0.13	14.6	
重庆市	403	366	10.0	116	77	52.0	286	288	−0.7							
四川省	1236	1226	0.8	836	775	7.8	401	451	−11.1							
贵州省	1179	1138	3.7	366	295	24.3	813	843	−3.5							
云南省	1040	905	14.9	622	431	44.2	418	474	−11.8				0.3			
西藏区	16	15	6.8	14.2	13.6	4.5	0.13	0.01								1.4
陕西省	769	645	19.3	54	52	5.4	715	591	21.0							
甘肃省	691	620	11.5	217	189	14.7	468	424	10.3				6.3	3.1	103.8	
青海省	322	302	6.6	216	205	5.1	107	97	9.8							
宁夏区	459	453	1.4	16	17	−7.2	440	435	1.3				2.4	0.5	370.7	
新疆区	479	420	14.0	74	70	6.6	397	346	14.9				7.8	5.0	56.5	

注　表中其他部分的统计口径与 2007 年不同。

?1994-2018 China Academic Journal Electronic Publishing House. All rights reserved.　http://www.cnki.net

2008年全国新增发电机组设备能力

万 kW

地区	合计	其中									
		水电		火电					核电	风电	其他
			其中：抽水蓄能		燃煤	燃气	燃油	其他			
**全国**	**9202**	**2148**	**175**	**6555**	**6040**	**321**	**8**	**185**		**499**	
北京市	145			145	16	129					
天津市	60			60	60						
河北省	365	75	75	261	257	2		2		30	
山西省	459			450	418			32		10	
内蒙古	773			611	515			96		162	
辽宁省	290			240	240					50	
吉林省	112			70	69			1		42	
黑龙江	219	1		189	184			6		29	
上海市	267			266	266					1	
江苏省	343	100	100	192	179			13		50	
浙江省	417	55		347	269	78				14	
安徽省	750			750	744			6			
福建省	372	41		316	246	70				15	
江西省	73			70	70					3	
山东省	326			309	307			2		17	
河南省	460	4		457	418	39					
湖北省	608	488		120	120						
湖南省	241	115		126	126						
广东省	300			300	300						
广西区	403	342		62	60			2			
海南省	17	0		12			2	10		5	
重庆市	218	185		33	33						
四川省	292	200		92	92						
贵州省	306	126		180	180						
云南省	316	306		3				3		8	
西藏区	11	3		8			7	2			
陕西省	535			535	522	3		10			
甘肃省	231	77		120	120					34	
青海省	22	9		14	14						
宁夏区	57			43	43	0				13	
新疆区	214	23		175	174			1		17	

?1994-2018 China Academic Journal Electronic Publishing House. All rights reserved. http://www.cnki.net

2008年全社会用电量情况

亿 kWh

地　　区	2008年	同比（%）
**全国合计**	**34 379.69**	**5.49**
北京市	689.72	3.40
天津市	515.88	4.24
河北省	2095.02	4.04
山西省	1314.33	−2.56
内蒙古	1220.57	5.20
辽宁省	1412.00	3.86
吉林省	496.49	7.32
黑龙江	669.90	6.51
上海市	1138.22	6.14
江苏省	3118.32	5.63
浙江省	2322.87	6.10
安徽省	858.88	11.67
福建省	1073.55	7.32
江西省	546.77	6.98
山东省	2726.97	5.04
河南省	1970.77	9.00
湖北省	1058.53	7.01
湖南省	916.79	2.94
广东省	3506.78	3.32
广西区	760.79	11.69
海南省	122.97	8.58
重庆市	487.69	8.56
四川省	1213.39	3.05
贵州省	679.18	1.51
云南省	829.44	7.76
西藏区	15.87	6.85
陕西省	708.03	8.25
甘肃省	677.76	10.25
青海省	313.23	9.74
宁夏区	439.62	−0.04
新疆区	479.37	15.98

?1994-2018 China Academic Journal Electronic Publishing House. All rights reserved. http://www.cnki.net
