# Supplementary material for: Prediction analysis of carbon emission in China’s electricity industry based on the dual carbon background
Source: PLoS One. 2024 May 17;19(5):e0302068. doi: 10.1371/journal.pone.0302068 (PMC11101092; doi:10.1371/journal.pone.0302068)
Supplement: S3 File — (ZIP) [file pone.0302068.s003.zip › China Electric Power Yearbook 2001-2021/统计资料-2011.pdf]

电力行业统计资料

2010 年电力统计基本数据一览表

统计口径	单 位	2010 年	2009 年	比 2009 年增长(%)
**一、发电装机容量**	**万 kW**	**96 641.30**	**87 409.72**	**10.56**
水 电	万 kW	21 605.72	19 629.02	10.07
火 电	万 kW	70 967.21	65 107.63	9.00
核 电	万 kW	1082.40	907.82	19.23
风 电	万 kW	2957.55	1759.94	68.05
地热、潮汐	万 kW	2.81	2.81	
太阳能	万 kW	25.62	2.50	925.14
**6000 千瓦及以上火电厂设备容量**	**万 kW**	**70 391.13**	**64 522.91**	**9.09**
其中：燃 煤	万 kW	64 660.64	59 215.17	9.20
燃 油	万 kW	877.99	823.00	6.68
燃 气	万 kW	2642.40	2402.96	9.96
煤矸石	万 kW	836.80	674.00	24.15
生物质	万 kW	170.45	108.80	56.66
垃 圾	万 kW	170.94	130.29	31.20
余温、余压、余气等	万 kW	1007.13	1168.70	—13.82
**二、关停小火电机组容量**	**万 kW**	**1305.24**	**1812.79**	**—28.00**
**三、新增发电设备能力**	**万 kW**	**9124.00**	**9667.35**	**—5.62**
水 电	万 kW	1642.85	2105.70	—21.98
火 电	万 kW	5830.56	6585.76	—11.47
其中：燃 煤	万 kW	5425.84	6186.71	—12.30
燃 气	万 kW	123.50	127.34	—3.02
煤矸石	万 kW	130.14	155.00	—16.04
生物质	万 kW	43.11	23.20	85.82
垃 圾	万 kW	14.03	11.34	23.72
余温、余压、余气等	万 kW	81.77	68.25	19.81
核 电	万 kW	173.69		
风 电	万 kW	1457.31	973.00	49.77
太阳能及地热	万 kW	19.59	2.79	602.15
**四、年底电源在建规模**	**万 kW**	**18 338.94**	**18 355.56**	**—0.09**
水 电	万 kW	6551.05	6724.80	—2.58

?1994-2018 China Academic Journal Electronic Publishing House. All rights reserved. http://www.cnki.net

续表

统计口径	单 位	2010年	2009年	比2009年增长(%)
火 电	万kW	7400.13	7749.10	-4.50
核 电	万kW	3395.42	3140.00	8.13
风 电	万kW	966.54	720.75	34.10
**五、基建新增110kV及以上输电线路长度**	**km**	**76 574**	**69 217**	**10.63**
其中：1000kV	km		640	-100.00
±800kV	km	1907	1375	38.69
750kV	km	4491	2021	122.24
±660kV	km	1335		
500kV	km	10 793	12 959	-16.71
其中：±500kV	km	3189	574	455.61
330kV	km	1699	1766	-3.78
220kV	km	24 499	22 697	7.94
110kV	km	31 850	27 760	14.73
**六、基建新增110kV及以上变电设备容量**	**万kVA**	**35 335**	**36 155**	**-2.27**
其中：1000kV	万kVA		600	-100.00
750kV	万kVA	1920	1080	77.78
500kV	万kVA	9495	11 545	-17.76
330kV	万kVA	960	780	23.08
220kV	万kVA	13 438	12 801	4.98
110kV	万kVA	9522	9349	1.85
**七、单机6000kW及以上机组平均单机容量**				
水电：单机容量	万kW/台	5.61	5.51	0.10
机组台数	台	3097	2860	237
机组容量	万kW	17 386.83	15 768.11	1619
火电：单机容量	万kW/台	10.88	10.31	0.57
机组台数	台	6373	6221	152
机组容量	万kW	69 349.28	64 133.47	5216
**八、35kV及以上输电线路长度**	**km**	**1 336 772**	**1 231 883**	**8.51**
其中：1000kV	km	1006	640	57.25
±800kV	km	3334	1375	142.45
750kV	km	6685	2640	153.22
±660kV	km	1095		
500kV	km	135 180	124 559	8.53
其中：±500kV	km	8081	6901	17.10
330kV	km	20 338	19 156	6.17
220kV	km	277 988	253 573	9.63
110kV	km	458 477	422 863	8.42
35kV	km	432 668	407 077	6.29
**九、35kV及以上变电设备容量(交流)**	**万kVA**	**361 742**	**319 542**	**13.21**
其中：1000kV	万kVA	600	600	
750kV	万kVA	3870	1740	122.41

?1994-2018 China Academic Journal Electronic Publishing House. All rights reserved. http://www.cnki.net

续表

统计口径	单位	2010年	2009年	比2009年增长(%)
500kV	万kVA	69 843	60 114	16.18
330kV	万kVA	6457	5523	16.91
220kV	万kVA	118 247	103 040	14.76
110kV	万kVA	125 224	112 958	10.86
35kV	万kVA	37 501	35 567	5.44
**十、电力投资当年完成**	**亿元**	**7417.47**	**7701.61**	**-3.69**
**1. 电源投资**	**亿元**	**3969.36**	**3803.31**	**4.37**
其中：水电	亿元	819.18	867.19	-5.54
火电	亿元	1426.14	1543.56	-7.61
核电	亿元	647.57	584.01	10.88
风电	亿元	1037.55	781.78	32.72
**2. 电网投资**	**亿元**	**3448.10**	**3898.30**	**-11.55**
送变电	亿元	3338.45	3776.91	-11.61
其中：直流	亿元	283.54	276.76	2.45
±800kV	亿元	116.05	189.44	-38.74
±660kV	亿元	71.40	9.08	686.41
±500kV	亿元	81.46	78.24	4.11
±400kV	亿元	14.62		
其中：交流	亿元	3054.92	3099.79	-1.45
1000kV	亿元	1.97	4.88	-59.65
750kV	亿元	173.85	125.46	38.57
500kV	亿元	444.13	699.74	-36.53
330kV	亿元	37.25	56.93	-34.57
220kV	亿元	1083.16	891.28	21.53
110kV及以下	亿元	1314.55	1321.50	-0.53
其他(含小型基建)	亿元	109.65	121.39	-9.67
**十一、发电量**	**亿kWh**	**42 277.71**	**36 811.86**	**14.85**
水电	亿kWh	6867.36	5716.82	20.13
火电	亿kWh	34 166.28	30 116.87	13.45
核电	亿kWh	747.42	700.50	6.70
风电	亿kWh	494.00	276.15	78.89
地热、潮汐、太阳能等	亿kWh	2.65	1.52	73.71
**6000kW及以上火电厂发电量**	**亿kWh**	**34 086.11**	**30 050.07**	**13.43**
其中：燃煤	亿kWh	32 162.69	28 347.71	13.46
燃油	亿kWh	161.89	170.76	-5.20
燃气	亿kWh	776.27	565.59	37.25
煤矸石	亿kWh	361.00	317.76	13.61
生物质	亿kWh	74.25	52.17	42.33
垃圾	亿kWh	86.84	67.48	28.70
余温、余压、余气等	亿kWh	455.99	528.60	-13.74

?1994-2018 China Academic Journal Electronic Publishing House. All rights reserved. http://www.cnki.net

续表

统计口径	单 位	2010年	2009年	比2009年增长(%)
**十二、6000kW及以上电厂供热量**	**万GJ**	**280 759.99**	**258 198.07**	**8.74**
**十三、6000kW及以上电厂供电煤耗**	**g/kWh**	**333**	**340**	**−7**
**十四、6000kW及以上电厂发电煤耗**	**g/kWh**	**312**	**320**	**−8**
**十五、6000kW及以上电厂厂用电率**	**%**	**5.43**	**5.76**	**−0.32**
水 电	%	0.33	0.40	−0.07
火 电	%	6.33	6.62	−0.29
**十六、6000kW及以上电厂利用小时数**	**h**	**4650**	**4546**	**104**
水 电	h	3404	3328	76
火 电	h	5031	4865	166
核 电	h	7840	7716	124
风 电	h	2047	2077	−30
**十七、供、售电量及线损**				
供电量	亿kWh	38 041.72	32 613.74	6.52
售电量	亿kWh	35 556.32	30 423.09	16.87
线损电量	亿kWh	2485.39	2190.65	13.45
线路损失率	%	6.53	6.72	−0.19
**十八、全社会用电量**	**亿kWh**	**41 998.82**	**36 598.42**	**14.76**
**A. 全行业用电合计**	**亿kWh**	**36 904.87**	**32 023.23**	**15.24**
第一产业	亿kWh	976.49	939.90	3.89
第二产业	亿kWh	31 450.01	27 139.60	15.88
其中：工业	亿kWh	30 966.77	26 757.69	15.73
1. 轻工业	亿kWh	5336.23	4635.56	15.12
2. 重工业	亿kWh	25 630.53	22 122.13	15.86
第三产业	亿kWh	4478.36	3943.73	13.56
**B. 城乡居民生活用电合计**	**亿kWh**	**5093.96**	**4575.19**	**11.34**
其中：城镇居民	亿kWh	2959.87	2670.03	10.86
乡村居民	亿kWh	2134.09	1905.16	12.02
**十九、6000kW及以上电厂燃料消耗**				
发电消耗标准煤量	万t	102 006.24	91 478.17	11.51
发电消耗原煤量	万t	158 970.92	139 669.62	13.82
供热消耗标准煤量	万t	11 171.65	10 199.06	9.54
供热消耗原煤量	万t	16 769.41	14 959.97	12.10
**二十、6000kW及以上电厂热效率**				
电厂热效率	%	39.42	38.44	0.99
电厂供热效率	%	85.75	86.38	−0.63
能源转换总效率	%	44.00	43.25	0.75
**二十一、发用电设备比**				
发电设备容量：用电设备容量		1∶3.18	1∶3.16	
**二十二、电力弹性系数**				
电力生产弹性系数		1.44	0.72	0.72
电力消费弹性系数		1.43	0.70	0.73

?1994-2018 China Academic Journal Electronic Publishing House. All rights reserved. http://www.cnki.net
