# Supplementary material for: Prediction analysis of carbon emission in China’s electricity industry based on the dual carbon background
Source: PLoS One. 2024 May 17;19(5):e0302068. doi: 10.1371/journal.pone.0302068 (PMC11101092; doi:10.1371/journal.pone.0302068)
Supplement: S3 File — (ZIP) [file pone.0302068.s003.zip › China Electric Power Yearbook 2001-2021/统计资料-2013.pdf]

电力行业统计资料[1]

2012 年电力统计基本数据一览表

	单 位	2012 年	2011 年	同比增长（%）
一、发电量	亿 kWh	49 865	47 306	5.41
水电	亿 kWh	8556	6681	28.06
其中：抽水蓄能	亿 kWh	93	109	－14.56
火电	亿 kWh	39 255	39 003	0.65
核电	亿 kWh	983	872	12.75
风电	亿 kWh	1030	741	39.15
太阳能发电	亿 kWh	36	6	494.22
其他	亿 kWh	5	2	117.20
6000kW 及以上火电厂发电量	亿 kWh	39 160	38 893	0.69
燃煤	亿 kWh	37 104	36 961	0.38
其中：煤矸石发电	亿 kWh	746	672	11.02
燃油	亿 kWh	54	59	－9.01
燃气	亿 kWh	1092	1088	0.39
其中：煤层气发电	亿 kWh	15	18	－16.74
其他	亿 kWh	911	785	16.09
其中：余温、余气、余压发电	亿 kWh	594	552	7.70
垃圾焚烧发电	亿 kWh	120	97	23.78
秸秆、蔗渣、林木质发电	亿 kWh	196	135	44.78
二、全社会用电量	亿 kWh	49 657	47 022	5.60
A. 全行业用电合计	亿 kWh	43 429	41 401	4.90
第一产业	亿 kWh	1003	1014	1.12
第二产业	亿 kWh	36 733	35 282	4.11
其中：工业	亿 kWh	36 122	34 710	4.07
1. 轻工业	亿 kWh	6114	5825	4.96
2. 重工业	亿 kWh	30 008	28 885	3.89
第三产业	亿 kWh	5693	5105	11.52
B. 城乡居民生活用电合计	亿 kWh	6228	5621	10.79

[1] 本统计资料未包含香港、澳门地区及台湾省资料。

?1994-2018 China Academic Journal Electronic Publishing House. All rights reserved. http://www.cnki.net

续表

	单　位	2012年	2011年	同比增长(%)
其中：城镇居民	亿kWh	3562	3202	11.25
乡村居民	亿kWh	2666	2419	10.18
三、发电装机容量	万kW	114 676	106 253	7.93
水电	万kW	24 947	23 298	7.08
其中：抽水蓄能	万kW	2033	1838	10.61
火电	万kW	81 968	76 834	6.68
核电	万kW	1257	1257	
风电	万kW	6142	4623	32.86
太阳能发电	万kW	341	212	60.63
其他	万kW	20.5	19.0	7.84
6000kW及以上火电厂装机容量	万kW	81 426	76 302	6.71
燃煤	万kW	75 382	70 929	6.28
其中：煤矸石发电	万kW	1574	1295	21.54
燃油	万kW	301	328	−8.23
燃气	万kW	3717	3415	8.84
其中：煤层气发电	万kW	29	38	−23.92
其他	万kW	2025	1631	24.19
其中：余温、余气、余发电	万kW	1256	1072	17.23
垃圾焚烧发电	万kW	251	210	19.91
秸秆、蔗渣、林木质发电	万kW	518	349	48.12
四、35kV及以上输电线路回路长度	km	1 479 963	1 409 698	4.98
1000kV	km	639	639	
±800kV	km	5466	3334	63.96
750kV	km	10 088	10 005	0.83
±660kV	km	1400	1400	
500kV	km	146 250	140 263	4.27
其中：±500kV	km	9145	8837	3.49
±400kV	km	1051	1051	
330kV	km	22 701	22 267	1.95
220kV	km	318 217	295 978	7.51
110kV(含66kV)	km	517 983	491 322	5.43
35kV	km	456 168	443 440	2.87
五、35kV及以上变电设备容量	万kVA	445 899	408 398	9.18
1000kV	万kVA	1800	1800	
±800kV	万kVA	4360	2669	63.34
750kV	万kVA	5320	5320	
±660kV	万kVA	946	946	

?1994-2018 China Academic Journal Electronic Publishing House. All rights reserved. http://www.cnki.net

续表

	单 位	2012年	2011年	同比增长(%)
500kV	万kVA	90 625	82 109	10.37
其中：±500kV	万kVA	7230	6011	20.27
±400kV	万kVA	141	71	98.86
330kV	万kVA	7714	7424	3.91
220kV	万kVA	144 228	131 060	10.05
110kV(含66kV)	万kVA	149 231	137 776	8.31
35kV	万kVA	41 534	39 223	5.89
六、新增发电装机容量	万kW	8315	9436	−11.88
水电	万kW	1676	1283	30.63
其中：抽水蓄能	万kW	165	175	−5.71
火电	万kW	5236	6241	−16.10
其中：燃煤	万kW	4788	5837	−17.97
其中：煤矸石发电	万kW	156	183	−14.75
燃油	万kW			
燃气	万kW	247	237	4.38
其中：煤层气发电	万kW	1		
其他	万kW	201	168	19.57
其中：余温、余气、余压	万kW	105	77	35.72
垃圾焚烧发电	万kW	20	26	−22.04
秸秆、蔗渣、林木质发电	万kW	75	65	14.98
核电	万kW		175	
风电	万kW	1296	1528	−15.18
太阳能发电	万kW	107	196	−45.23
其他	万kW		13.1	
七、火电机组退役和关停容量	万kW	616	955	−35.52
八、年底主要发电企业电源在建规模	万kW	16 235	17 084	−4.97
水电	万kW	6648	7121	−6.64
火电	万kW	5166	5558	−7.05
核电	万kW	3383	3347	1.08
风电	万kW	971	1047	−7.23
九、新增110kV及以上输电线路长度	km	66 269	66 903	−0.95
1000kV	km		1:4	
±800kV	km	2090		
750kV	km	741	2740	−72.96
±660kV	km			
500kV	km	4747	7331	−35.24
其中：±500kV	km			

?1994-2018 China Academic Journal Electronic Publishing House. All rights reserved. http://www.cnki.net

续表

	单位	2012年	2011年	同比增长(%)
±400kV	km		1038	
330kV	km	219	965	−77.32
220kV	km	26 431	24 129	9.54
110kV(含66kV)	km	32 040	30 698	4.37
十、新增110kV及以上变电设备容量	万kVA	28 835	31 713	−9.07
1000kV	万kVA		1200	
±800kV	万kVA	1440		
750kV	万kVA		1660	
±660kV	万kVA		400	
500kV	万kVA	7650	6465	18.33
其中：±500kV	万kVA	450	600	−25.00
±400kV	万kVA		120	
330kV	万kVA	372	614	−39.41
220kV	万kVA	11 269	12 032	−6.34
110kV(含66kV)	万kVA	9994	9222	8.37
十一、电力投资当年完成	亿元	7393	7614	−2.90
1. 电源投资	亿元	3732	3927	−4.98
水电	亿元	1239	971	27.64
火电	亿元	1002	1133	−11.55
核电	亿元	784	764	2.69
风电	亿元	607	902	−32.70
太阳能发电	亿元	99	155	−36.18
其他	亿元		2.5	
2. 电网投资	亿元	3661	3687	−0.69
送变电	亿元	3458	3498	−1.13
其中：直流	亿元	278	222	25.19
交流	亿元	3180	3275	−2.90
其他	亿元	203	189	7.38
十二、单机6000kW及以上机组平均单机容量				
水电：单机容量	万kW/台	5.77	5.66	0.11
机组台数	台	3530	3328	202
机组容量	万kW	20 377	18 834	1543
火电：单机容量	万kW/台	11.80	11.40	0.40
机组台数	台	6805	6595	210
机组容量	万kW	80 302	75 215	5087
十三、6000kW及以上电厂供热量	万GJ	307 749	297 859	3.32
十四、6000kW及以上电厂发电标准煤耗	g/kWh	305	308	−3

?1994-2018 China Academic Journal Electronic Publishing House. All rights reserved. http://www.cnki.net

续表

	单 位	2012年	2011年	同比增长（%）
十五、6000kW及以上电厂供电标准煤耗	g/kWh	325	329	－4
十六、6000kW及以上电厂厂用电率	%	5.10	5.39	－0.29
水电	%	0.33	0.36	－0.03
火电	%	6.08	6.23	－0.15
十七、6000kW及以上电厂发电设备利用小时	h	4579	4730	－151
水电	h	3591	3019	572
其中：抽水蓄能	h	592	619	－27
火电	h	4982	5305	－323
核电	h	7855	7759	96
风电	h	1929	1875	54
十八、6000kW及以上电厂燃料消耗				
发电消耗标准煤量	万t	114 770	114 400	0.32
发电消耗原煤量	万t	178 968	182 382	－1.87
供热消耗标准煤量	万t	12 247	11 854	3.32
供热消耗原煤量	万t	18 447	18 262	1.01
十九、6000kW及以上火电厂热效率				
电厂热效率	%	41.91	41.76	0.15
电厂供热效率	%	85.74	85.74	
电厂能源转换总效率	%	46.10	44.37	1.73
二十、供、售电量及线损				
供电量	亿kWh	44 798	42 768	4.75
售电量	亿kWh	41 781	39 980	4.51
线损电量	亿kWh	3018	2788	8.23
线路损失率	%	6.74	6.52	0.22
二十一、发用电设备比				
发电装机容量：用电设备容量		1∶3.47	1∶3.31	
二十二、电力弹性系数				
电力生产弹性系数		0.69	1.28	－0.58
电力消费弹性系数		0.72	1.29	－0.57

注 1. 2012年，全国基建新增生物质发电装机容量99万kW，同比增长8.51%；年底6000kW及以上电厂生物质装机容量769万kW，同比增长37.57%；全年6000kW及以上电厂生物质发电量316亿kWh，同比增长35.62%；

2. 本年35kV及以上变电设备容量包含换流站两端变压器容量，2011年同期数据相应调整。

?1994-2018 China Academic Journal Electronic Publishing House. All rights reserved. http://www.cnki.net
