# Supplementary material for: Prediction analysis of carbon emission in China’s electricity industry based on the dual carbon background
Source: PLoS One. 2024 May 17;19(5):e0302068. doi: 10.1371/journal.pone.0302068 (PMC11101092; doi:10.1371/journal.pone.0302068)
Supplement: S3 File — (ZIP) [file pone.0302068.s003.zip › China Electric Power Yearbook 2001-2021/统计资料-2015.pdf]

电力统计基本数据一览表

基本数据项目	单位	2014 年	2013 年	比 2013 年增长（±、%）
**一、发电量**	**亿 kWh**	**56 045**	**53 721**	**4.33**
水电	亿 kWh	10 601	8921	18.83
其中：抽水蓄能	亿 kWh	132	107	23.51
火电	亿 kWh	42 274	42 216	0.14
其中：燃煤	亿 kWh	39 510	39 805	−0.74
燃气	亿 kWh	1333	1164	14.52
燃油	亿 kWh	44	52	−15.10
核电	亿 kWh	1332	1115	19.48
风电	亿 kWh	1598	1383	15.55
太阳能发电	亿 kWh	235	84	180.78
其他	亿 kWh	5	3	92.22
**6000kW 及以上火电厂发电量**	**亿 kWh**	**42 169**	**42 134**	**0.08**
燃煤	亿 kWh	39 449	39 776	−0.82
其中：煤矸石发电	亿 kWh	1587	1042	52.22
燃气	亿 kWh	1322	1156	14.36
其中：常规燃气	亿 kWh	1288	1129	14.07
煤层气发电	亿 kWh	21	27	−19.86
燃油	亿 kWh	44	52	−15.08
其他	亿 kWh	1354	1120	20.82
其中：余温、余气、余压发电	亿 kWh	892	737	21.02
垃圾焚烧发电	亿 kWh	245	176	38.98
秸秆、蔗渣、林木质发电	亿 kWh	216	207	4.62
**二、全社会用电量**	**亿 kWh**	**55 637**	**53 423**	**4.14**
**1. 全行业用电合计**	**亿 kWh**	**48 701**	**46 630**	**4.44**
第一产业	亿 kWh	1013	1026	−1.22
第二产业	亿 kWh	41 017	39 335	4.28
其中：工业	亿 kWh	40 296	38 660	4.23
其中：轻工业	亿 kWh	6693	6427	4.15
重工业	亿 kWh	33 603	32 233	4.25
第三产业	亿 kWh	6670	6269	6.39
**2. 城乡居民生活用电合计**	**亿 kWh**	**6936**	**6793**	**2.10**
其中：城镇居民	亿 kWh	3933	3860	1.89

?1994-2018 China Academic Journal Electronic Publishing House. All rights reserved. http://www.cnki.net

续表

基本数据项目	单位	2014年	2013年	比2013年增长（±、%）
乡村居民	亿kWh	3003	2933	2.39
**三、发电装机容量**	**万kW**	**137 018**	**125 768**	**8.95**
水电	万kW	30 486	28 044	8.71
其中：抽水蓄能	万kW	2211	2153	2.68
火电	万kW	92 363	87 009	6.15
其中：燃煤	万kW	83 233	79 578	4.59
燃气	万kW	5697	4277	33.19
燃油	万kW	512	590	−13.33
核电	万kW	2008	1466	36.97
风电	万kW	9657	7652	26.20
太阳能发电	万kW	2486	1589	56.50
其他	万kW	19	8	128.45
**6000kW及以上火电厂装机容量**	**万kW**	**91 861**	**86 473**	**6.23**
燃煤	万kW	83 106	79 450	4.60
其中：煤矸石发电	万kW	3349	2062	62.42
燃气	万kW	5666	4252	33.26
其中：常规燃气	万kW	5526	4157	32.93
煤层气发电	万kW	91	95	−4.64
燃油	万kW	270	293	−7.70
其他	万kW	2819	2478	13.74
其中：余温、余气、余压发电	万kW	1838	1610	14.15
垃圾焚烧发电	万kW	469	359	30.58
秸秆、蔗渣、林木质发电	万kW	512	509	0.55
**四、35kV及以上输电线路回路长度**	**km**	**1 628 472**	**1 554 236**	**4.78**
**1. 交流**	**km**	**1 603 488**	**1 534 248**	**4.51**
其中：1000kV	km	3111	1936	60.70
750kV	km	13 881	12 666	9.59
500kV	km	152 107	146 166	4.06
330kV	km	25 146	24 065	4.49
220kV	km	358 377	339 075	5.69
110kV	km	566 571	545 815	3.80
35kV	km	484 296	464 525	4.26
**2. 直流**	**km**	**24 984**	**19 988**	**24.99**
其中：±800kV	km	10 132	6904	46.75
±660kV	km	1336	1400	−4.54
±500kV	km	11 875	10 653	11.48
±400kV	km	1640	1031	59.00
**五、35kV及以上变电设备容量**	**万kVA**	**526 685**	**483 427**	**8.95**
**1. 交流**	**万kVA**	**509 134**	**470 047**	**8.32**
其中：1000kV	万kVA	5700	3900	46.15

?1994-2018 China Academic Journal Electronic Publishing House. All rights reserved. http://www.cnki.net

续表

基本数据项目	单位	2014年	2013年	比2013年增长（±、%）
750kV	万kVA	8090	6500	24.47
500kV	万kVA	100 011	90 112	10.99
330kV	万kVA	10 493	8575	22.36
220kV	万kVA	167 342	155 699	7.48
110kV	万kVA	171 588	161 661	6.14
35kV	万kVA	45 909	43 600	5.30
**2. 直流**	**万kVA**	**17 551**	**13 380**	**31.17**
其中：±800kV	万kVA	3180	4654	−31.67
±660kV	万kVA		948	−100.00
±500kV	万kVA	14 230	7637	86.32
±400kV	万kVA	141	141	0.00
**六、新增发电装机容量**	**万kW**	**10 443**	**10 222**	**2.16**
水电	万kW	2180	3096	−29.59
其中：抽水蓄能	万kW	60	120	−50.00
火电	万kW	4791	4175	14.74
其中：燃煤	万kW	3498	3447	1.47
其中：煤矸石发电	万kW	30	61	−50.50
燃气	万kW	946	440	115.18
其中：常规燃气	万kW	938	439	113.91
煤层气发电		8		
燃油	万kW		15	−100.00
其他	万kW	346	273	26.83
其中：余温、余气、余压	万kW	254	182	39.43
垃圾焚烧发电	万kW	21	34	−39.36
秸秆、蔗渣、林木质发电	万kW	71	56	26.32
核电	万kW	547	221	147.84
风电	万kW	2101	1487	41.25
太阳能发电	万kW	825	1243	−33.63
其他	万kW			
**七、火电机组退役和关停容量**	**万kW**	**909**	**803**	**13.15**
**八、年底主要发电企业电源项目在建规模**	**万kW**	**14 500**	**15 950**	**−9.10**
水电	万kW	4328	5129	−15.61
火电	万kW	5524	5953	−7.21
核电	万kW	2863	3387	−15.49
风电	万kW	1676	1457	15.04
**九、新增直流输电线路长度及换流容量**				
**1. 线路长度**	**km**	**2876**	**4846**	**−40.65**
其中：±800kV	km	1653	3623	−54.37
±660kV	km			
±500kV	km	1223	1223	0.00

?1994-2018 China Academic Journal Electronic Publishing House. All rights reserved. http://www.cnki.net

续表

基本数据项目	单位	2014 年	2013 年	比 2013 年增长（±、%）
±400kV	km			
**2. 换流容量**	**万 kW**	**3860**	**1370**	**181.75**
其中：±800kV	万 kW	2900	1050	176.19
±660kV	万 kW			
±500kV	万 kW	960	320	200.00
±400kV	万 kW			
**十、新增交流 110kV 及以上输电线路长度及变电设备容量**				
**1. 线路长度**	**km**	**59 799**	**59 604**	**0.33**
其中：1000kV	km	1206	1298	−7.09
750kV	km	1314	2218	−40.78
500kV	km	7272	7469	−2.64
330kV	km	1202	1598	−24.77
220kV	km	22 098	21 498	2.79
110kV（含 66kV）	km	26 708	25 523	4.64
**2. 变电设备容量**	**万 kVA**	**30 853**	**28 577**	**7.97**
其中：1000kV	万 kVA	1800	2100	−14.29
750kV	万 kVA	660	1180	−44.07
500kV	万 kVA	7555	5580	35.39
330kV	万 kVA	741	573	29.32
220kV	万 kVA	11 602	10 398	11.58
110kV（含 66kV）	万 kVA	8495	8746	−2.87
**十一、本年完成电力投资**	**亿元**	**7805**	**7728**	**1.00**
**1. 电源投资**	**亿元**	**3686**	**3872**	**−4.80**
水电	亿元	943	1223	−22.92
火电	亿元	1145	1016	12.67
核电	亿元	533	660	−19.31
风电	亿元	915	650	40.93
太阳能发电	亿元	150	323	−53.43
其他	亿元			
**2. 电网投资**	**亿元**	**4119**	**3856**	**6.82**
送变电	亿元	3993	3768	5.98
其中：直流	亿元	168	354	−52.68
交流	亿元	3825	3413	12.07
其他	亿元	126	89	42.52
**十二、单机 6000kW 及以上机组平均单机容量**				
水电：单机容量	万 kW/台	6.34	5.91	7.36
机组台数	台	3945	3998	−1.33
机组容量	万 kW	25 020	23 617	5.94
火电：单机容量	万 kW/台	12.53	11.75	6.59
机组台数	台	7162	7223	−0.84

?1994-2018 China Academic Journal Electronic Publishing House. All rights reserved. http://www.cnki.net

续表

基本数据项目	单位	2014年	2013年	比2013年增长（±、%）
机组容量	万 kW	89 723	84 891	5.69
**十三、6000kW及以上电厂供热量**	**万 GJ**	**318 362**	**324 128**	**−1.78**
**十四、6000kW及以上电厂发电标准煤耗**	**g/kWh**	**300**	**302**	**−2**
**十五、6000kW及以上电厂供电标准煤耗**	**g/kWh**	**319**	**321**	**−2**
**十六、6000kW及以上电厂厂用电率**	**%**	**4.83**	**5.05**	**−0.23**
水电	%	0.50	0.33	0.17
火电	%	5.84	6.01	−0.18
**十七、6000kW及以上电厂发电设备利用小时**	**h**	**4318**	**4521**	**−204**
水电	h	3669	3359	310
其中：抽水蓄能	h	609	513	97
火电	h	4739	5021	−282
核电	h	7787	7874	−87
风电	h	1900	2025	−124
太阳能发电	h	1235	1342	−107
**十八、6000kW及以上电厂燃料消耗**				
发电消耗标煤量	万 t	117 737	122 127	−3.59
发电消耗原煤量	万 t	175 777	185 782	−5.39
供热消耗标煤量	万 t	12 310	12 834	−4.08
供热消耗原煤量	万 t	18 631	19 168	−2.80
**十九、6000kW及以上火电厂热效率**				
电厂热效率	%	43.99	42.38	1.62
电厂供热效率	%	88.24	86.17	2.07
电厂能源转换总效率	%	48.18	45.42	2.76
**二十、供、售电量及线损**				
供电量	亿 kWh	48 676	47 075	3.40
售电量	亿 kWh	45 442	43 768	3.82
线损电量	亿 kWh	3234	3306	−2.19
线路损失率	%	6.64	7.02	−0.38
**二十一、发用电设备比**				
发电装机容量：用电设备容量		1：3.71	1：3.51	
**二十二、电力弹性系数**				
电力生产弹性系数		0.58	1.00	−0.42
电力消费弹性系数		0.56	0.98	−0.43

注 1. 风电、太阳能发电装机容量和发电量等指标均为并网口径。
2. 变电设备容量包含换流变容量，2014年国家电网公司调整±800kV和±660kV换流容量至转换对应电压等级。
3. 2014年起国家电网公司供售电统计中剔除了自发自用电量，相应按照新口径调整了2013年供售电量、线损数值。
4. 生物质发电2014年底装机容量1030万kW，2014年发电量474亿kWh。

?1994-2018 China Academic Journal Electronic Publishing House. All rights reserved. http://www.cnki.net
