# Supplementary material for: Prediction analysis of carbon emission in China’s electricity industry based on the dual carbon background
Source: PLoS One. 2024 May 17;19(5):e0302068. doi: 10.1371/journal.pone.0302068 (PMC11101092; doi:10.1371/journal.pone.0302068)
Supplement: S3 File — (ZIP) [file pone.0302068.s003.zip › China Electric Power Yearbook 2001-2021/统计资料-2019.pdf]

电力统计基本数据一览

项目	单 位	2018 年	2017 年	比上年增长（%）
**一、发电量**	**亿 kWh**	**69 947**	**64 529**	**8.40**
水电	亿 kWh	12 321	11 947	3.13
其中：抽水蓄能	亿 kWh	329	328	0.44
火电	亿 kWh	49 249	45 877	7.35
其中：燃煤	亿 kWh	44 829	41 782	7.29
燃气	亿 kWh	2155	2032	6.04
燃油	亿 kWh	15	27	-42.53
生物质发电	亿 kWh	936	813	15.23
核电	亿 kWh	2950	2481	18.87
风电	亿 kWh	3658	3046	20.09
太阳能发电	亿 kWh	1769	1178	50.24
其他	亿 kWh	1	1	-4.15
**6000kW 及以上火电厂发电量**	**亿 kWh**	**49 167**	**45 762**	**7.44**
燃煤	亿 kWh	44 821	41 772	7.30
其中：煤矸石发电	亿 kWh	1286	1423	-9.58
燃气	亿 kWh	2134	2016	5.86
其中：常规燃气	亿 kWh	2053	1957	4.93
煤层气发电	亿 kWh	70	52	35.94
燃油	亿 kWh	15	27	-42.45
其他	亿 kWh	2143	1920	11.62
其中：余温、余气、余压发电	亿 kWh	1230	1129	8.96
垃圾焚烧发电	亿 kWh	481	376	28.08
秸秆、蔗渣、林木质发电	亿 kWh	427	413	3.40
**二、全社会用电量**	**亿 kWh**	**69 002**	**63 636**	**8.43**
**1. 全行业用电合计**	**亿 kWh**	**59 310**	**54 849**	**8.13**
第一产业	亿 kWh	746	684	8.99
第二产业	亿 kWh	47 733	44 571	7.09
其中：工业	亿 kWh	46 954	43 874	7.02
第三产业	亿 kWh	10 831	9593	12.90
**2. 城乡居民生活用电合计**	**亿 kWh**	**9692**	**8788**	**10.29**
城镇居民	亿 kWh	5531	5010	10.40
乡村居民	亿 kWh	4162	3778	10.15

(C)1994-2024 China Academic Journal Electronic Publishing House. All rights reserved. http://www.cnki.net

续表

项目	单 位	2018 年	2017 年	比上年增长（%）
**三、发电装机容量**	**万 kW**	**190 012**	**178 451**	**6.48**
水电	万 kW	35 259	34 411	2.46
其中：抽水蓄能	万 kW	2999	2869	4.52
火电	万 kW	114 408	111 009	3.06
其中：燃煤	万 kW	100 835	98 562	2.31
燃气	万 kW	8375	7580	10.49
燃油	万 kW	173	197	-11.94
生物质发电	万 kW	1947	1651	17.93
核电	万 kW	4466	3582	24.68
风电	万 kW	18 427	16 400	12.35
太阳能发电	万 kW	17 433	13 042	33.66
其他	万 kW	20	7	200.35
**6000kW 及以上火电厂装机容量**	**万 kW**	**114 100**	**110 506**	**3.25**
燃煤	万 kW	100 794	98 517	2.31
其中：煤矸石发电	万 kW	3240	3518	-7.90
燃气	万 kW	8313	7532	10.37
其中：常规燃气发电	万 kW	8139	7400	9.98
煤层气发电	万 kW	151	113	33.32
燃油	万 kW	170	193	-12.11
其他	万 kW	4755	4199	13.24
其中：余温、余气、余发电	万 kW	2854	2626	8.68
垃圾焚烧发电	万 kW	889	722	23.08
秸秆、蔗渣、林木质发电	万 kW	969	850	13.97
**四、35kV 及以上输电线路回路长度**	**km**	**1 892 018**	**1 825 611**	**3.64**
**1. 交流**	**km**	**1 850 631**	**1 788 212**	**3.49**
其中：1000kV	km	11 005	10 073	9.25
750kV	km	20 543	18 830	9.10
500kV	km	187 158	173 772	7.70
330kV	km	30 477	30 183	0.97
220kV	km	434 493	415 311	4.62
110kV	km	652 891	631 361	3.41
35kV	km	514 066	508 682	1.06
**2. 直流**	**km**	**41 995**	**37 399**	**12.29**
其中：±1100kV	km	608		
±800kV	km	21 723	20 874	4.07
±660kV	km	2091	1334	56.75
±500kV	km	15 428	13 552	13.84
±400kV	km	1640	1640	0.00

(C)1994-2024 China Academic Journal Electronic Publishing House. All rights reserved. http://www.cnki.net

续表

项目	单 位	2018 年	2017 年	比上年增长（%）
五、35kV 及以上变电设备容量	万 kVA	699 219	662 928	5.47
**1. 交流**	**万 kVA**	**666 622**	**630 730**	**5.69**
其中：1000kV	万 kVA	14 700	13 800	6.52
750kV	万 kVA	17 030	14 540	17.13
500kV	万 kVA	136 494	125 508	8.75
330kV	万 kVA	13 125	13 029	0.74
220kV	万 kVA	213 127	203 352	4.81
110kV	万 kVA	219 379	209 847	4.54
35kV	万 kVA	52 767	50 654	4.17
**2. 直流**	**万 kVA**	**33 196**	**32 198**	**3.10**
其中：±1100kV	万 kVA	600		
±800kV	万 kVA	17 361	17 841	-2.69
±660kV	万 kVA	947	947	0.00
±500kV	万 kVA	13 353	13 410	-0.43
±400kV	万 kVA	141		
**六、新增发电装机容量**	**万 kW**	**12 785**	**13 019**	**-1.80**
水电	万 kW	859	1287	-33.27
其中：抽水蓄能	万 kW	130	200	-35.00
火电	万 kW	4380	4453	-1.65
其中：燃煤	万 kW	3056	3504	-12.79
燃气	万 kW	884	571	54.87
其中：常规燃气	万 kW	881	571	54.42
煤层气发电		2		
燃油	万 kW			
其他	万 kW	440	379	16.26
其中：余温、余气、余压	万 kW	198	175	13.13
垃圾焚烧发电	万 kW	148	123	20.53
秸秆、蔗渣、林木质发电	万 kW	94	81	16.51
核电	万 kW	884	218	306.44
风电	万 kW	2127	1720	23.64
太阳能发电	万 kW	4525	5341	-15.26
其他	万 kW	10		
**七、火电机组退役和关停容量**	**万 kW**	**1197**	**929**	**28.79**
**八、年底主要发电企业电源项目在建规模**	**万 kW**	**17 890**	**20 804**	**-14.01**
水电	万 kW	7940	7887	0.68
火电	万 kW	6936	8637	-19.70
核电	万 kW	1345	2289	-41.23
风电	万 kW	1564	1909	-18.07

(C)1994-2024 China Academic Journal Electronic Publishing House. All rights reserved. http://www.cnki.net

续表

项目	单位	2018年	2017年	比上年增长（%）
九、新增直流输电线路长度及换流容量				
**1. 线路长度**	**km**	**3325**	**8339**	**-60.13**
其中：±1100kV	km	3325		
±800kV	km		8339	-100.00
±660kV	km			
±500kV	km			
±400kV	km			
**2. 换流容量**	**万kW**	**3200**	**7900**	**-59.49**
其中：±1100kV	万kW	1200		
±800kV	万kW	2000	7700	-74.03
±660kV	万kW			
±500kV	万kW		200	-100.00
±400kV	万kW			
**十、新增交流110kV及以上输电线路长度及变电设备容量**				
**1. 线路长度**	**km**	**56 973**	**58 084**	**-1.91**
其中：1000kV	km	129	2846	-95.47
750kV	km	1573	899	74.97
500kV	km	14 540	7999	81.77
330kV	km	828	2521	-67.16
220kV	km	20 697	18 810	10.03
110kV（含66kV）	km	19 206	25 010	-23.21
**2. 变电设备容量**	**万kVA**	**31 024**	**32 595**	**-4.82**
其中：1000kV	万kVA	900	3000	-70.00
750kV	万kVA	1140	1740	-34.48
500kV	万kVA	11 160	8275	34.86
330kV	万kVA	612	783	-21.84
220kV	万kVA	8402	10 433	-19.47
110kV（含66kV）	万kVA	8810	8364	5.33
**十一、本年完成电力投资**	**亿元**	**8161**	**8239**	**-0.94**
**1. 电源投资**	**亿元**	**2787**	**2900**	**-3.89**
水电	亿元	700	622	12.65
火电	亿元	786	858	-8.31
核电	亿元	447	454	-1.55
风电	亿元	646	681	-5.12
太阳能发电	亿元	207	285	-27.38
其他	亿元			
**2. 电网投资**	**亿元**	**5374**	**5339**	**0.65**
送变电	亿元	5133	5135	-0.04
其中：直流	亿元	520	859	-39.41
交流	亿元	4613	4276	7.87

(C)1994-2024 China Academic Journal Electronic Publishing House. All rights reserved. http://www.cnki.net

续表

项目	单 位	2018 年	2017 年	比上年增长（%）
其他	亿元	241	204	18.10
**十二、单机 6000kW 及以上机组平均单机容量**				
水电：单机容量	万 kW/台	6.10	6.07	0.02
机组台数	台	4894	4816	1.62
机组容量	万 kW	29 830	29 252	1.98
火电：单机容量	万 kW/台	13.38	13.15	0.23
机组台数	台	8070	7796	3.51
机组容量	万 kW	107 969	102 501	5.33
**十三、6000kW 及以上电厂供热量**	**万 GJ**	**480 625**	**421 084**	**14.14**
**十四、6000kW 及以上电厂发电标准煤耗**	**g/kWh**	**289.9**	**291.3**	**-1.3**
**十五、6000kW 及以上电厂供电标准煤耗**	**g/kWh**	**307.6**	**309.4**	**-1.8**
**十六、6000kW 及以上电厂厂用电率**	**%**	**4.69**	**4.80**	**-0.11**
水电	%	0.25	0.27	-0.02
火电	%	5.95	6.04	-0.09
**十七、6000kW 及以上电厂发电设备利用小时**	h	**3880**	**3790**	**90**
水电	h	3607	3597	10
其中：抽水蓄能	h	1102	1176	-74
火电	h	4378	4219	159
核电	h	7543	7089	454
风电	h	2103	1949	155
太阳能发电	h	1230	1205	25
**十八、6000kW 及以上电厂燃料消耗**				
发电消耗标煤量	万 t	130 805	121 811	7.38
发电消耗原煤量	万 t	195 719	177 592	10.21
供热消耗标煤量	万 t	18 104	16 519	9.60
供热消耗原煤量	万 t	27 523	24 248	13.51
**十九、供、售电量及线损**				
供电量	亿 kWh	59 508	54 357	9.48
售电量	亿 kWh	55 777	50 835	9.72
线损电量	亿 kWh	3731	3522	5.93
线路损失率	%	6.27	6.48	-0.21
**二十、发用电设备比**				
发电装机容量：用电设备容量		1:3.96	1:4.05	
**二十一、电力弹性系数**				
电力生产弹性系数		1.27	0.95	34.07
电力消费弹性系数		1.28	0.95	34.41

注 1. 电源投资完成额口径为全国主要发电企业。

2. 从 2018 年 5 月开始，三次产业划分按照《国家统计局关于修订〈三次产业划分规定（2012）〉的通知》（国统设管函〔2018〕74 号）调整，为保证数据可比，同期数据根据新标准重新进行了分类。

(C)1994-2024 China Academic Journal Electronic Publishing House. All rights reserved. http://www.cnki.net
