# Supplementary material for: Prediction analysis of carbon emission in China’s electricity industry based on the dual carbon background
Source: PLoS One. 2024 May 17;19(5):e0302068. doi: 10.1371/journal.pone.0302068 (PMC11101092; doi:10.1371/journal.pone.0302068)
Supplement: S3 File — (ZIP) [file pone.0302068.s003.zip › China Electric Power Yearbook 2001-2021/统计资料-2021.pdf]

电力统计基本数据一览表

项　　目	单位	2020 年	2019 年	比上年增长（±、%）
**一、发电量**	**亿 kWh**	**76264**	**73269**	**4.09**
水　　电	亿 kWh	13553	13021	4.09
其中：抽水蓄能	亿 kWh	335	319	4.99
火　　电	亿 kWh	51770	50465	2.59
其中：燃煤	亿 kWh	46296	45538	1.66
燃气	亿 kWh	2525	2325	8.61
燃油	亿 kWh	12	13	-5.08
其中：生物质发电	亿 kWh	1355	1126	20.35
核　　电	亿 kWh	3662	3487	5.03
风　　电	亿 kWh	4665	4053	15.08
太阳能发电	亿 kWh	2611	2240	16.56
其　　他	亿 kWh	3	2	12.40
**非化石能源发电量**	**亿 kWh**	**25850**	**23930**	**8.02**
**二、全社会用电量**	**亿 kWh**	**75214**	**72852**	**3.24**
**A. 全行业用电合计**	**亿 kWh**	**64268**	**62607**	**2.65**
第一产业	亿 kWh	859	779	10.15
第二产业	亿 kWh	51318	49963	2.71
其中：工业	亿 kWh	50398	49073	2.70
第三产业	亿 kWh	12091	11865	1.91
**B. 城乡居民生活用电合计**	**亿 kWh**	**10946**	**10245**	**6.84**
城镇居民	亿 kWh	6157	5835	5.52
乡村居民	亿 kWh	4789	4410	8.60
**三、发电装机容量**	**万 kW**	**220204**	**201006**	**9.55**
水　　电	万 kW	37028	35804	3.42
其中：抽水蓄能	万 kW	3149	3029	3.96
火　　电	万 kW	124624	118957	4.76
其中：燃煤	万 kW	107912	104063	3.70
燃气	万 kW	9972	9024	10.51
燃油	万 kW	147	175	-15.99
其中：生物质发电	万 kW	2987	2361	26.51
核　　电	万 kW	4989	4874	2.36

(C)1994-2024 China Academic Journal Electronic Publishing House. All rights reserved.　http://www.cnki.net

续表

项　　目	单位	2020年	2019年	比上年增长（±、%）
风　　电	万kW	28165	20915	34.66
太阳能发电	万kW	25356	20429	24.12
其　　他	万kW	41	26	58.02
**非化石能源发电装机容量**	**万kW**	**98567**	**84410**	**16.77**
**四、35kV及以上输电线路回路长度**	**km**	**2156170**	**1975312**	**9.16**
**1. 交流**	**km**	**2109846**	**1932947**	**9.15**
其中：1000kV	km	13361	10872	22.89
750kV	km	25046	23256	7.70
500kV	km	203058	195636	3.79
330kV	km	36597	32314	13.25
220kV	km	488543	454585	7.47
110kV	km	752563	684406	9.96
35kV	km	590678	531880	11.05
**2. 直流**	**km**	**46324**	**42364**	**9.35**
其中：±1100kV	km	3295	3295	
±800kV	km	24980	21907	14.03
±660kV	km	1334	1334	
±500kV	km	14783	13733	7.64
±400kV	km	1639	1639	
**五、35kV及以上变电设备容量**	**万kVA**	**812893**	**747833**	**8.70**
**1. 交流**	**万kVA**	**766565**	**708718**	**8.16**
其中：1000kV	万kVA	18000	15300	17.65
750kV	万kVA	19785	18515	6.86
500kV	万kVA	155163	145905	6.35
330kV	万kVA	15771	14062	12.16
220kV	万kVA	243736	226101	7.80
110kV	万kVA	250286	235077	6.47
35kV	万kVA	63823	53757	18.72
**2. 直流**	**万kVA**	**46328**	**37706**	**22.87**
其中：±1100kV	万kVA	2867	2867	
±800kV	万kVA	27690	22317	24.07
±660kV	万kVA	947	947	
±500kV	万kVA	12738	10945	16.38
±400kV	万kVA	1245	1245	

(C)1994-2024 China Academic Journal Electronic Publishing House. All rights reserved.　http://www.cnki.net

续表

项　　目	单位	2020年	2019年	比上年增长（±、%）
**六、新增发电装机容量**	**万kW**	**19144**	**10500**	**82.31**
水　　电	万kW	1313	445	195.22
其中：抽水蓄能	万kW	120	30	300.00
火　　电	万kW	5660	4423	27.95
其中：燃煤	万kW	4030	3236	24.52
燃气	万kW	824	630	30.72
其中：常规燃气	万kW	811	629	28.89
煤层气发电	万kW	12	0	
燃油	万kW			
其他	万kW	805	557	44.73
其中：余温、余气、余压	万kW	283	166	70.89
垃圾焚烧发电	万kW	300	273	9.91
秸秆、蔗渣、林木质发电	万kW	222	118	88.76
核　　电	万kW	112	409	-72.64
风　　电	万kW	7211	2572	180.40
太阳能发电	万kW	4820	2652	81.76
其　　他	万kW	28		
**七、火电机组退役和关停容量**	**万kW**	**1469**	**1024**	**43.44**
**八、年底主要发电企业电源项目在建规模**	**万kW**	**16137**	**18192**	**-11.29**
水　　电	万kW	8186	8462	-3.27
火　　电	万kW	3883	5409	-28.22
核　　电	万kW	1547	1420	9.00
风　　电	万kW	1996	2736	-27.04
**九、新增直流输电线路长度及换流容量**				
**1. 线路长度**	**km**	**4444**		
其中：±1100kV	km			
±800kV	km	3389		
±660kV	km			
±500kV	km	1055		
±400kV	km			
**2. 换流容量**	**万kW**	**5200**	**2200**	**136.36**
其中：±1100kV	万kW		1200	
±800kV	万kW	4000		
±660kV	万kW			

(C)1994-2024 China Academic Journal Electronic Publishing House. All rights reserved.　http://www.cnki.net

续表

项　　目	单位	2020 年	2019 年	比上年增长（±、%）
±500kV	万 kW	1200		
±400kV	万 kW		1000	
十、新增交流 110kV 及以上输电线路长度及变电设备容量				
**1. 线路长度**	**km**	**57237**	**57935**	**-1.20**
其中：1000kV	km	1736	2100	-17.35
750kV	km	1090	4406	-75.26
500kV	km	7424	5595	32.70
330kV	km	1566	3989	-60.73
220kV	km	18768	19822	-5.32
110kV（含 66kV）	km	26653	22023	21.02
**2. 变电设备容量**	**万 kVA**	**31292**	**31915**	**-1.95**
其中：1000kV	万 kVA	1800	1500	20.00
750kV	万 kVA	1860	3245	-42.68
500kV	万 kVA	8255	8645	-4.51
330kV	万 kVA	1098	1263	-13.06
220kV	万 kVA	9275	9161	1.24
110kV（含 66kV）	万 kVA	9004	8100	11.15
十一、本年完成电力投资	亿元	**10189**	**9097**	**12.00**
**1. 电源投资**	**亿元**	**5292**	**4085**	**29.55**
水　　电	亿元	1067	905	17.90
火　　电	亿元	568	780	-27.26
核　　电	亿元	379	463	-17.95
风　　电	亿元	2653	1552	70.96
太阳能发电	亿元	625	385	62.18
其　　他	亿元			
**2. 电网投资**	**亿元**	**4896**	**5012**	**-2.30**
输变电	亿元	4721	4779	-1.23
其中：直流	亿元	532	249	113.36
交流	亿元	4188	4530	-7.54
其　　他	亿元	176	232	-24.29
十二、单机 6000kW 及以上机组平均单机容量				
水电：单机容量	万 kW/台	6.23	6.04	0.19
机组台数	台	5158	5099	1.16
机组容量	万 kW	32137	30788	4.38

(C)1994-2024 China Academic Journal Electronic Publishing House. All rights reserved.　http://www.cnki.net

续表

项　　目	单位	2020年	2019年	比上年增长（±、%）
火电：单机容量	万kW/台	13.55	13.37	0.18
机组台数	台	8776	8430	4.10
机组容量	万kW	118890	112722	5.47
**十三、6000kW及以上电厂供热量**	**万GJ**	**519422**	**492492**	**5.47**
**十四、6000kW及以上电厂发电标准煤耗**	**g/kWh**	**287.2**	**288.8**	**-1.55**
**十五、6000kW及以上电厂供电标准煤耗**	**g/kWh**	**304.9**	**306.4**	**-1.50**
**十六、6000kW及以上电厂厂用电率**	**%**	**4.65**	**4.67**	**-0.02**
水　　电	%	0.25	0.24	0.003
火　　电	%	5.98	6.01	-0.03
**十七、6000kW及以上电厂发电设备利用小时**	**h**	**3756**	**3828**	**-72**
水　　电	h	3825	3697	128
其中：抽水蓄能	h	1094	1053	40
火　　电	h	4211	4307	-97
其中：燃煤发电	h	4323	4429	-106
燃气发电	h	2610	2646	-37
核　　电	h	7450	7394	56
风　　电	h	2078	2083	-5
太阳能发电	h	1281	1291	-10
**十八、6000kW及以上电厂燃料消耗**				
发电消耗标煤量	万t	139561	132007	5.72
发电消耗原煤量	万t	208088	199443	4.33
供热消耗标煤量	万t	22678	19463	16.52
供热消耗原煤量	万t	29710	29227	1.65
**十九、供、售电量及线损**				
供电量	亿kWh	65232	62835	3.81
售电量	亿kWh	61581	59111	4.18
线损电量	亿kWh	3651	3724	-1.97
线损率	%	5.60	5.93	-0.33
**二十、发用电设备比**				
发电装机容量:用电设备容量		1:4.32	1:4.08	
**二十一、电力弹性系数**				
电力生产弹性系数		1.78	0.78	
电力消费弹性系数		1.41	0.73	

注　电源投资完成额口径为全国主要发电企业。

陕西用电量不含陕西省地方电力（集团）有限公司经营区范围内的部分自备电厂用电量，下同。

(C)1994-2024 China Academic Journal Electronic Publishing House. All rights reserved.　http://www.cnki.net
