# Supplementary material for: Prediction analysis of carbon emission in China’s electricity industry based on the dual carbon background
Source: PLoS One. 2024 May 17;19(5):e0302068. doi: 10.1371/journal.pone.0302068 (PMC11101092; doi:10.1371/journal.pone.0302068)
Supplement: S4 File — (ZIP) [file pone.0302068.s004.zip › China Energy Statistic Yearbook 2001-2021/中国能源统计年鉴_林贤郁_四、全国能源平衡表-2000-2002.pdf]

4-1 中国能源平衡表(标准量)-2000

(万吨标准煤)

		能源合计 Energy Total	
		(发电煤耗计算法) (coal equivalent calculation)	(电热当量计算法) (calorific value calculation)
**一、可供本地区消费的能源量**	**Total Primary Energy Supply**	**115149.81**	**109495.79**
1.一次能源生产量	Indigenous Production	106988.20	101130.09
水 电	Hydro Power	8181.59	2733.47
核 电	Nuclear Power	615.68	205.70
2.回 收 能	Recovery of Energy	1759.74	1759.74
3.进 口 量	Import	13874.40	13836.53
4.我轮、机在外国加油量	China Airplanes&ships Refueling in Abroad	456.77	456.77
5.出 口 量(-)	Export (-)	-8855.88	-8613.92
6.外轮、机在我国加油量(-)	Foreign Airplanes&ships Refueling in China	-170.09	-170.09
7.库存增(-)、减(+)量	Stock Change	1096.67	1096.67
**二、加工转换投入(-)产出(+)量**	**Input(-) & Output(+) of Transformation**	**-2371.41**	**-30539.25**
1.火力发电	Thermal Power		-27347.84
2.供 热	Heating Supply		-820.00
3.洗 选 煤	Coal Washing	-916.64	-916.64
4.炼 焦	Coking	-487.29	-487.29
5.炼 油	Petroleum Refineries	-780.55	-780.55
6.制 气	Gas Works	-45.41	-45.41
#焦炭再投入量(-)	Coke Input (-)	-138.78	-138.78
7.煤制品加工	Briquettes	-2.74	-2.74
**三、损 失 量**	**Loss**	**3893.38**	**1588.99**
**四、终端消费量**	**Total Final Consumption**	**124031.78**	**92517.52**
1.农、林、牧、渔、水利业	Farming, Forestry, Animal Husbandry, Fishery & Water Conservancy	5787.12	4138.36
2.工 业	Industry	83707.35	61700.86
#用作原料、材料	Non-Energy Use	7280.37	7280.37
3.建 筑 业	Construction	1432.96	1053.09
4.交通运输、仓储及邮电通迅业	Transport, Storage, Postal & Telecommunications Services	9577.29	8884.88
5.批发和零售贸易业、餐饮业	Wholesale, Retail Trade and Catering Service	2893.16	1922.52
6.生活消费	Residential Consumption	14911.83	10685.95
城 镇	Urban	9073.26	6500.76
乡 村	Rural	5838.58	4185.19
7.其 他	Other	5722.04	4131.85
**五、平衡差额**	**Statistical Difference**	**-15146.76**	**-15149.97**
**六、能源消费总量**	**Total Energy Consumption**	**130296.57**	**124645.76**

(C)1994-2024 China Academic Journal Electronic Publishing House. All rights reserved. http://www.cnki.net

ENERGY BALANCE OF CHINA －2000 (STANDARD QUANTITY)

(10^4tce)

煤合计<br>Coal Total	原煤<br>Raw Coal	洗精煤<br>Cleaned Coal	其他洗煤<br>Other Washed Coal	型煤<br>Briquettes	焦炭<br>Coke	焦炉煤气<br>Coke Oven Gas	其他煤气<br>Other Gas	其他焦化产品<br>Other Coking Products	油品合计<br>Petroleum Products Total
**70056.89**	**70444.35**	**-364.07**	**-25.57**	**2.18**	**-1254.76**		**1175.57**	**31.17**	**32448.38**
71287.14	71287.14								23286.18
							1175.57		
161.19	126.96	30.97	3.26					52.53	13603.81
									456.77
-4061.29	-3469.80	-590.45	-0.55	-0.49	-1476.24			-21.36	-2933.63
									-170.09
2669.85	2500.05	195.41	-28.28	2.67	221.48				-1794.66
**-58246.65**	**-61364.90**	**826.91**	**1837.53**	**453.81**	**11022.20**	**1323.37**	**172.05**	**220.22**	**-3056.08**
-38793.19	-37867.35	-187.72	-738.12			-65.67	-201.36		-1690.98
-4734.45	-4572.28	-22.13	-140.04			-59.10	-100.45		-622.22
-916.64	-14758.09	11088.09	2753.36						
-13113.07	-3424.65	-9664.42	-24.00		10943.89	1431.85	43.93	205.41	
									-705.91
-686.56	-293.86	-386.91	-5.79		217.09	16.29	429.93	14.81	-36.97
					-138.78				
-2.74	-448.67		-7.88	453.81					
						**13.03**			**276.12**
**27993.16**	**25206.77**	**966.49**	**1362.74**	**457.16**	**9356.46**	**1336.87**	**1346.95**	**249.50**	**28830.71**
994.53	981.27		13.26		131.01				2183.87
20327.06	18550.44	958.75	813.21	4.66	9029.83	1085.77	1078.33	249.50	12674.95
516.92	501.10	10.28	5.54		567.67			51.43	5193.31
324.94	313.87	3.99	7.08		17.25				505.15
688.65	671.65	3.65	13.33	0.02	10.21	0.12	0.60		7804.46
491.94	482.69	0.09	5.37	3.79	32.45	11.35	7.59		811.28
4718.38	3843.61		428.16	446.61	124.67	220.87	257.24		2087.82
1830.64	1341.35		199.83	289.46	61.39	220.87	254.97		1685.47
2887.74	2502.26		228.33	157.15	63.28		2.27		402.35
447.66	363.24	0.01	82.33	2.08	11.04	18.76	3.19		2763.18
**-16182.92**	**-16127.32**	**-503.65**	**449.22**	**-1.17**	**410.98**	**-26.53**	**0.67**	**1.89**	**285.47**

(C)1994-2024 China Academic Journal Electronic Publishing House. All rights reserved. http://www.cnki.net

续表(2000)

(万吨标准煤)

		原油 Crude Oil	汽油 Gasoline
**一、可供本地区消费的能源量**	**Total Primary Energy Supply**	**30547.73**	**-927.24**
1.一次能源生产量	Indigenous Production	23286.18	
水电	Hydro Power		
核电	Nuclear Power		
2.回收能	Recovery of Energy		
3.进口量	Import	10038.10	0.04
4.我轮、机在外国加油量	China Airplanes&ships Refueling in Abroad		
5.出口量(-)	Export (-)	-1472.32	-669.78
6.外轮、机在我国加油量(-)	Foreign Airplanes&ships Refueling in China		-18.39
7.库存增(-)、减(+)量	Stock Change	-1304.23	-239.11
**二、加工转换投入(-)产出(+)量**	**Input(-) & Output(+) of Transformation**	**-29149.58**	**6081.99**
1.火力发电	Thermal Power	-121.43	-1.47
2.供热	Heating Supply	-20.00	-0.29
3.洗选煤	Coal Washing		
4.炼焦	Coking		
5.炼油	Petroleum Refineries	-29008.15	6083.75
6.制气	Gas Works		
#焦炭再投入量(-)	Coke Input (-)		
7.煤制品加工	Briquettes		
**三、损失量**	**Loss**	**272.73**	
**四、终端消费量**	**Total Final Consumption**	**909.72**	**5155.37**
1.农、林、牧、渔、水利业	Farming, Forestry, Animal Husbandry, Fishery & Water Conservancy		271.49
2.工业	Industry	874.78	883.99
#用作原料、材料	Non-Energy Use	139.40	13.24
3.建筑业	Construction	4.71	170.02
4.交通运输、仓储及邮电通迅业	Transport, Storage, Postal & Telecommunications Services	27.97	2041.98
5.批发和零售贸易业、餐饮业	Wholesale, Retail Trade and Catering Service	0.26	308.76
6.生活消费	Residential Consumption		187.72
城镇	Urban		140.03
乡村	Rural		47.69
7.其他	Other	2.00	1291.41
**五、平衡差额**	**Statistical Difference**	**215.70**	**-0.62**
**六、能源消费总量**	**Total Energy Consumption**		

(C)1994-2024 China Academic Journal Electronic Publishing House. All rights reserved. http://www.cnki.net

Continued(2000)

(10^4 tce)

煤油 Kerosene	柴油 Diesel Oil	燃料油 Fuel Oil	液化石油气 PLG	炼厂干气 Refinery Gas	其他石油制品 Other Petroleum Products	天然气 Natural Gas	热力 Heat	电力 Electricity	其他能源 Other Energy
**12.65**	**-398.00**	**2547.17**	**822.08**		**-156.01**	**3617.60**		**2836.77**	**584.17**
						3617.60		2939.17	
								2733.47	
								205.70	
									584.17
375.90	37.78	2114.50	825.85		211.64			19.00	
98.61	37.88	320.28							
-292.63	-80.84	-47.67	-2.74		-367.65			-121.40	
-84.55	-32.11	-35.04							
-84.68	-360.71	195.10	-1.03						
**1283.49**	**9983.99**	**1317.66**	**1568.10**	**890.23**	**4968.04**	**-391.82**	**4983.24**	**13721.16**	**-286.94**
	-331.72	-1163.19	-3.26	-39.28	-30.63	-202.29		13721.16	-115.51
		-416.05		-160.19	-25.69	-189.53	4983.24		-97.49
									0.70
1283.49	10315.71	2933.87	1571.36	1089.70	5024.36				-74.64
		-36.97							
			**3.39**			**88.58**	**60.04**	**1151.22**	
**1279.55**	**9539.05**	**3916.40**	**2336.23**	**882.36**	**4812.03**	**2778.51**	**4923.04**	**15405.11**	**297.21**
2.21	1909.00	0.57	0.60				1.88	827.07	
123.52	1994.48	2633.95	469.84	882.36	4812.03	2270.18	3974.95	10713.08	297.21
0.24	10.55	141.63	12.86	63.36	4812.03	653.83			297.21
5.89	285.39	23.87	15.27			10.91	4.63	190.21	
788.52	3706.58	1214.31	25.10			13.30	21.95	345.59	
17.66	372.93	16.56	95.11			45.75	38.33	483.83	
106.19	99.60		1694.31			429.86	792.28	2054.83	
9.56	63.86		1472.02			429.86	792.28	1225.28	
96.63	35.74		222.29					829.55	
235.56	1171.07	27.14	36.00			8.51	89.02	790.49	
16.59	46.94	-51.57	50.56	7.87		358.69	0.16	1.60	0.02
							85.87	33.41	

(C)1994-2024 China Academic Journal Electronic Publishing House. All rights reserved. http://www.cnki.net

4-1 中国能源平衡表(实物量)-2000

		煤合计<br>Coal Total<br>万吨<br>10^4 tn	原煤<br>Raw Coal<br>万吨<br>10^4 tn
**一、可供本地区消费的能源量**	**Total Primary Energy Supply**	**98176.09**	**98620.12**
1.一次能源生产量	Indigenous Production	99800.00	99800.00
水 电	Hydro Power		
核 电	Nuclear Power		
2.回 收 能	Recovery of Energy		
3.进 口 量	Import	217.88	177.74
4.我轮、机在外国加油量	China Airplanes&Ships Refueling in Abroad		
5.出 口 量(-)	Export (-)	-5506.47	-4857.62
6.外轮、机在我国加油量(-)	Foreign Airplanes&Ships Refueling in China		
7.库存增(-)、减(+)量	Stock Change	3664.68	3500.00
**二、加工转换投入(-)产出(+)量**	**Input(-) & Output(+) of Transformation**	**-78450.61**	**-83608.52**
1.火力发电	Thermal Power	-54611.20	-53000.10
2.供 热	Heating Supply	-6692.07	-6401.17
3.洗 选 煤	Coal Washing	-1441.20	-18833.70
4.炼 焦	Coking	-15000.40	-4370.41
5.炼 油	Petroleum Refineries		
6.制 气	Gas Works	-809.99	-375.01
#焦炭再投入量(-)	Coke Input (-)		
7.煤制品加工	Briquettes	104.25	-628.13
**三、损 失 量**	**Loss**		
**四、终端消费量**	**Total Final Consumption**	**46086.79**	**41675.85**
1.农、林、牧、渔、水利业	Farming, Forestry, Animal Husbandry, Fishery & Water Conservancy	1647.68	1622.39
2.工 业	Industry	33279.74	30670.56
#用作原料、材料	Non-Energy Use	850.32	828.50
3.建 筑 业	Construction	536.82	518.94
4.交通运输、仓储及邮电通迅业	Transport, Storage, Postal & Telecommunications Services	1139.94	1110.48
5.批发和零售贸易业、餐饮业	Wholesale, Retail Trade and Catering Service	814.64	798.06
6.生活消费	Residential Consumption	7906.96	6354.86
城 镇	Urban	3075.56	2217.73
乡 村	Rural	4831.40	4137.13
7.其 他	Other	761.01	600.56
**五、平衡差额**	**Statistical Difference**	**-26361.31**	**-26664.25**

(C)1994-2024 China Academic Journal Electronic Publishing House. All rights reserved. http://www.cnki.net

ENERGY BANLANCE OF CHINA -2000(PHYSICAL QUANTITY)

洗精煤<br>Cleaned Coal<br>万吨<br>10^4 tn	其他洗煤<br>Other Washed Coal<br>万吨<br>10^4 tn	型煤<br>Briquettes<br>万吨<br>10^4 tn	焦炭<br>Coke<br>万吨<br>10^4 tn	焦炉煤气<br>Coke Oven Gas<br>亿立方米<br>10^8 cu.m	其他煤气<br>Other Gas<br>亿立方米<br>10^8 cu.m	其他焦化产品<br>Other Coking Products<br>万吨<br>10^4 tn	油品合计<br>Petroleum Products Total<br>万吨<br>10^4 tn	原油<br>Crude Oil<br>万吨<br>10^4 tn
**-398.93**	**-48.70**	**3.60**	**-1291.70**		**408.78**	**27.01**	**22631.77**	**21382.99**
							16300.00	16300.00
					408.78			
33.94	6.20					45.52	9431.28	7026.53
							317.21	
-647.00	-1.05	-0.80	-1519.70			-18.51	-2055.61	-1030.60
							-116.53	
214.13	-53.85	4.40	228.00				-1244.58	-912.94
**906.10**	**3504.43**	**747.38**	**12041.15**	**221.45**	**59.87**	**190.83**	**-2352.92**	**-20404.30**
-205.70	-1405.40			-10.69	-70.02		-1178.15	-85.00
-24.25	-266.65			-9.62	-34.93		-426.97	-14.00
12150.00	5242.50							
-10589.99	-40.00		11960.54	239.04	15.28	178.00		
							-721.92	-20305.30
-423.96	-11.02		223.48	2.72	149.54	12.83	-25.88	
			-142.87					
	-15.00	747.38						
				**2.18**			**192.89**	**190.91**
**1059.06**	**2598.98**	**752.90**	**10297.14**	**223.71**	**468.42**	**216.20**	**19893.51**	**636.80**
	25.29		144.18				1496.90	
1050.58	1550.93	7.67	9937.68	181.69	375.00	216.20	9016.23	612.34
11.26	10.56		624.74			44.57	3932.00	97.58
4.37	13.51		18.98				344.33	3.30
4.00	25.42	0.04	11.24	0.02	0.21		5351.71	19.58
0.10	10.24	6.24	35.71	1.90	2.64		545.03	0.18
	816.58	735.52	137.20	36.96	89.46		1256.45	
	381.12	476.71	67.56	36.96	88.67		1004.17	
	435.46	258.81	69.64		0.79		252.28	
0.01	157.01	3.43	12.15	3.14	1.11		1882.86	1.40
**-551.89**	**856.75**	**-1.92**	**452.31**	**-4.44**	**0.23**	**1.64**	**192.45**	**150.98**

(C)1994-2024 China Academic Journal Electronic Publishing House. All rights reserved. http://www.cnki.net

续表(2000)

		汽油 Gasoline 万吨 10^4tn	煤油 Kerosene 万吨 10^4tn
**一、可供本地区消费的能源量**	**Total Primary Energy Supply**	**-630.18**	**8.60**
1.一次能源生产量	Indigenous Production		
水　电	Hydro Power		
核　电	Nuclear Power		
2.回 收 能	Recovery of Energy		
3.进 口 量	Import	0.03	255.47
4.我轮、机在外国加油量	China Airplanes&Ships Refueling in Abroad		67.02
5.出 口 量(-)	Export (-)	-455.20	-198.88
6.外轮、机在我国加油量(-)	Foreign Airplanes&ships Refueling in China	-12.50	-57.46
7.库存增(-)、减(+)量	Stock Change	-162.51	-57.55
**二、加工转换投入(-)产出(+)量**	**Input(-) & Output(+) of Transformation**	**4133.47**	**872.29**
1.火力发电	Thermal Power	-1.00	
2.供　热	Heating Supply	-0.20	
3.洗 选 煤	Coal Washing		
4.炼　焦	Coking		
5.炼　油	Petroleum Refineries	4134.67	872.29
6.制　气	Gas Works		
#焦炭再投入量(-)	Coke Input (-)		
7.煤制品加工	Briquettes		
**三、损 失 量**	**Loss**		
**四、终端消费量**	**Total Final Consumption**	**3503.71**	**869.61**
1.农、林、牧、渔、水利业	Farming, Forestry, Animal Husbandry, Fishery & Water Conservancy	184.51	1.50
2.工　业	Industry	600.78	83.95
#用作原料、材料	Non-Energy Use	9.00	0.16
3.建 筑 业	Construction	115.55	4.00
4.交通运输、仓储及邮电通迅业	Transport, Storage, Postal & Telecommunications Services	1387.78	535.90
5.批发和零售贸易业、餐饮业	Wholesale, Retail Trade and Catering Service	209.84	12.00
6.生活消费	Residential Consumption	127.58	72.17
城　镇	Urban	95.17	6.50
乡　村	Rural	32.41	65.67
7.其　他	Other	877.67	160.09
**五、平衡差额**	**Statistical Difference**	**-0.42**	**11.28**

(C)1994-2024 China Academic Journal Electronic Publishing House. All rights reserved.   http://www.cnki.net

Continued(2000)

柴油<br>Diesel Oil<br>万吨<br>10^4tn	燃料油<br>Fuel Oil<br>万吨<br>10^4tn	液化石油气<br>PLG<br>万吨<br>10^4tn	炼厂干气<br>Refinery Gas<br>万吨<br>10^4tn	其他石油制品<br>Other Petroleum Products<br>万吨<br>10^4tn	天然气<br>Natural Gas<br>亿立方米<br>10^8 cu.m	热力<br>Heat<br>万百万千焦<br>10^{10} kj	电力<br>Electricity<br>亿千瓦小时<br>10^8kwh	其他能源<br>Other Energy<br>万吨标煤<br>10^4tce
**-273.14**	**1782.98**	**479.54**		**-119.02**	**272.00**		**2308.19**	**584.17**
					272.00		2391.51	
							2224.14	
							167.37	
								584.17
25.93	1480.12	481.74		161.46			15.46	
26.00	224.19							
-55.48	-33.37	-1.60		-280.48			-98.78	
-22.04	-24.53							
-247.55	136.57	-0.60						
**6851.96**	**922.34**	**914.72**	**566.52**	**3790.08**	**-29.46**	**146136.16**	**11164.49**	**-286.94**
-227.66	-814.22	-1.90	-25.00	-23.37	-15.21		11164.49	-115.51
	-291.23		-101.94	-19.60	-14.25	146136.16		-97.49
								0.70
7079.62	2053.67	916.62	693.46	3833.05				-74.64
	-25.88							
		**1.98**			**6.66**	**1760.58**	**936.71**	
**6546.61**	**2741.42**	**1362.79**	**561.51**	**3671.06**	**208.91**	**144370.70**	**12534.67**	**297.21**
1310.14	0.40	0.35				55.08	672.96	
1368.80	1843.72	274.07	561.51	3671.06	170.69	116567.55	8716.91	297.21
7.24	99.14	7.50	40.32	3671.06	49.16			297.21
195.86	16.71	8.91			0.82	135.64	154.77	
2543.81	850.00	14.64			1.00	643.71	281.20	
255.94	11.59	55.48			3.44	1124.12	393.68	
68.36		988.34			32.32	23234.03	1671.95	
43.83		858.67			32.32	23234.03	996.97	
24.53		129.67					674.98	
803.70	19.00	21.00			0.64	2610.57	643.20	
**32.21**	**-36.10**	**29.49**	**5.01**		**26.97**	**4.88**	**1.30**	**0.02**

(C)1994-2024 China Academic Journal Electronic Publishing House. All rights reserved. http://www.cnki.net

4－2 中国能源平衡表(标准量)－2001

(万吨标准煤)

		能源合计 Energy Total	
		(发电煤耗计算法)(coal equivalent calculation)	(电热当量计算法)(calorific value calculation)
**一、可供本地区消费的能源量**	**Total Primary Energy Supply**	**125309.78**	**118615.29**
1.一次能源生产量	Indigenous Production	120900.00	113996.27
水　电	Hydro Power	10325.98	3409.64
核　电	Nuclear Power	650.30	214.73
2.回 收 能	Recovery of Energy	1858.54	1858.54
3.进 口 量	Import	12967.56	12922.73
4.我轮、机在外国加油量	China Airplanes&ships Refueling in Abroad	503.49	503.49
5.出 口 量(－)	Export (－)	－10941.18	－10687.11
6.外轮、机在我国加油量(－)	Foreign Airplanes&ships Refueling in China	－203.53	－203.53
7.库存增(－)、减(＋)量	Stock Change	225	225
**二、加工转换投入(－)产出(＋)量**	**Input(－) & Output(＋) of Transformation**	**－2011.03**	**－32291.30**
1.火力发电	Thermal Power		－29336.21
2.供　热	Heating Supply		－944.61
3.洗 选 煤	Coal Washing	－792.33	－791.88
4.炼　焦	Coking	－386.71	－387.18
5.炼　油	Petroleum Refineries	－635.67	－635.67
6.制　气	Gas Works	－43.11	－43.11
＃焦炭再投入量(－)	Coke Input (－)	－149.26	－149.26
7.煤制品加工	Briquettes	－3.82	－3.38
**三、损 失 量**	**Loss**	**3953.09**	**1693.30**
**四、终端消费量**	**Total Final Consumption**	**128950.63**	**94466.57**
1.农、林、牧、渔、水利业	Farming, Forestry, Animal Husbandry, Fishery & Water Conservancy	6232.83	4331.86
2.工　业	Industry	86710.88	62488.43
＃用作原料、材料	Non-Energy Use	7594.09	7594.09
3.建 筑 业	Construction	1452.39	1090.17
4.交通运输、仓储及邮电通迅业	Transport, Storage, Postal & Telecommunications Services	9929.20	9128.13
5.批发和零售贸易业、餐饮业	Wholesale, Retail Trade and Catering Service	3164.51	2048.23
6.生活消费	Residential Consumption	15426.56	11076.91
城　镇	Urban	9468.25	6745.85
乡　村	Rural	5958.31	4331.06
7.其　他	Other	6034.25	4302.85
**五、平衡差额**	**Statistical Difference**	**－9604.97**	**－9835.88**
**六、能源消费总量**	**Total Energy Consumption**	**134914.75**	**128451.17**

(C)1994-2024 China Academic Journal Electronic Publishing House. All rights reserved.　http://www.cnki.net

ENERGY BALANCE OF CHINA - 2001 (STANDARD QUANTITY)

(10⁴tce)

煤合计<br>Coal Total	原煤<br>Raw Coal	洗精煤<br>Cleaned Coal	其他洗煤<br>Other Washed Coal	型煤<br>Briquettes	焦炭<br>Coke	焦炉煤气<br>Coke Oven Gas	其他煤气<br>Other Gas	其他焦化产品<br>Other Coking Products	油品合计<br>Petroleum Products Total
**77506.12**	**78323**	**-725.38**	**-91.07**	**-0.43**	**-1620.13**		**1242.35**	**40.51**	**33275.67**
82915.00	82915								23423.14
							1242.35		
192.84	159	25.25	8.59					54.81	12652.98
									503.49
-6482.59	-5619	-862.86	-0.55	-0.18	-1345.00			-14.30	-2719.57
									-203.53
881	868	112	-99		-275				-381
**-61687.07**	**-64901**	**944.90**	**1791.16**	**477.87**	**11878.90**	**1145.54**	**135.02**	**235.34**	**-2983.97**
-41438.58	-40598	-176.46	-664.12			-65.24	-241.62	-0.76	-1740.79
-5043.97	-4909	-24.12	-110.85			-43.19	-115.38	-1.03	-637.83
-791.88	-14848	11462.68	2593.44						
-13671.60	-3798	-9858.41	-15.19		11795.50	1235.54	32.77	220.41	
									-572.84
-737.66	-272	-458.79	-6.87		232.66	18.43	459.25	16.72	-32.51
					-149.26				
-3.38	-476		-5.25	477.87					
						**13.48**			**265.73**
**26977.54**	**24247**	**926.36**	**1325.89**	**478.29**	**9838.14**	**1151.52**	**1376.89**	**266.35**	**29499.15**
978.47	965		13.47		126.30				2288.25
19349.04	17671	919.16	754.63	4.25	9510.82	950.30	1123.61	266.35	12723.45
523.16	508	9.91	5.25		703.07			55.23	5229.41
329.42	319	3.59	6.83		21.69				546.04
643.11	627	3.51	12.57	0.03	10.60	0.10	0.72		8079.77
495.46	486	0.09	5.18	4.19	36.04	9.95	8.63		845.19
4720.75	3804		449.07	467.68	121.73	175.90	240.42		2148.43
1831.08	1319		210.10	301.98	61.91	175.90	238.09		1719.29
2889.67	2485		238.97	165.70	59.82		2.33		429.14
461.29	375	0.01	84.14	2.14	10.96	15.27	3.51		2868.02
**-11158.49**	**-10825**	**-706.84**	**374.20**	**-0.85**	**420.63**	**-19.46**	**0.48**	**9.50**	**526.82**

(C)1994-2024 China Academic Journal Electronic Publishing House. All rights reserved. http://www.cnki.net

续表(2001)

(万吨标准煤)

		原油 Crude Oil	汽油 Gasoline
**一、可供本地区消费的能源量**	**Total Primary Energy Supply**	**30767.99**	**-806.04**
1.一次能源生产量	Indigenous Production	23423.14	
水　电	Hydro Power		
核　电	Nuclear Power		
2.回收能	Recovery of Energy		
3.进口量	Import	8608.74	0.03
4.我轮、机在外国加油量	China Airplanes&ships Refueling in Abroad		
5.出口量(-)	Export (-)	-1078.59	-842.32
6.外轮、机在我国加油量(-)	Foreign Airplanes&ships Refueling in China		-19.86
7.库存增(-)、减(+)量	Stock Change	-185.30	56.11
**二、加工转换投入(-)产出(+)量**	**Input(-) & Output(+) of Transformation**	**-29315.89**	**6112.11**
1.火力发电	Thermal Power	-116.57	-0.88
2.供　热	Heating Supply	-17.60	-0.18
3.洗选煤	Coal Washing		
4.炼　焦	Coking		
5.炼　油	Petroleum Refineries	-29181.72	6113.17
6.制　气	Gas Works		
#焦炭再投入量(-)	Coke Input (-)		
7.煤制品加工	Briquettes		
**三、损失量**	**Loss**	**263.26**	
**四、终端消费量**	**Total Final Consumption**	**916.45**	**5292.67**
1.农、林、牧、渔、水利业	Farming, Forestry, Animal Husbandry, Fishery & Water Conservancy		280.45
2.工　业	Industry	883.81	908.47
#用作原料、材料	Non-Energy Use	142.49	12.45
3.建筑业	Construction	4.72	171.71
4.交通运输、仓储及邮电通迅业	Transport, Storage, Postal & Telecommunications Services	26.03	2088.46
5.批发和零售贸易业、餐饮业	Wholesale, Retail Trade and Catering Service	0.21	314.94
6.生活消费	Residential Consumption		198.05
城　镇	Urban		147.02
乡　村	Rural		51.03
7.其　他	Other	1.68	1330.59
**五、平衡差额**	**Statistical Difference**	**272.39**	**13.40**
**六、能源消费总量**	**Total Energy Consumption**		

(C)1994-2024 China Academic Journal Electronic Publishing House. All rights reserved. http://www.cnki.net

Continued(2001)

(10^4 tce)

煤油 Kerosene	柴油 Diesel Oil	燃料油 Fuel Oil	液化石油气 PLG	炼厂干气 Refinery Gas	其他石油制品 Other Petroleum Products	天然气 Natural Gas	热力 Heat	电力 Electricity	其他能源 Other Energy
**154.32**	**-311.19**	**2817.86**	**815.49**		**-162.76**	**4033.36**		**3521.22**	**616.19**
						4033.76		3624.37	
								3409.64	
								214.73	
									616.19
297.06	40.03	2605.19	838.05		263.88			22.10	
142.28	39.66	321.55							
-268.12	-37.33	-62.99	-3.58		-426.64	-0.40		-125.25	
-94.46	-30.95	-58.26							
77.56	-322.60	12.37	-18.98						
**1161.45**	**10557.00**	**1004.98**	**1630.78**	**864.27**	**5001.33**	**-391.15**	**5224.24**	**14462.29**	**-310.44**
	-350.27	-1197.94	-1.80	-36.14	-37.19	-172.90		14462.29	-138.61
		-428.04		-157.14	-34.87	-218.25	5224.24		-109.20
1161.45	10907.27	2663.47	1632.58	1057.55	5073.39				-62.83
		-32.51							
			**2.47**			**82.73**	**61.23**	**1270.13**	
**1309.95**	**10006.32**	**3841.91**	**2414.87**	**878.41**	**4838.57**	**3174.32**	**5162.84**	**16714.39**	**305.43**
2.24	2004.45	0.60	0.51				1.86	936.98	
126.54	2034.73	2554.90	498.02	878.41	4838.57	2486.31	4206.77	11566.35	305.43
0.26	13.46	136.75	16.29	69.14	4838.57	777.79			305.43
5.15	325.05	23.11	16.30			9.58	5.35	178.09	
825.00	3891.92	1221.45	26.91			15.96	23.43	380.15	
18.35	390.61	17.54	103.54			66.50	39.69	546.77	
110.36	115.36		1724.66			586.66	796.90	2260.41	
10.33	74.49		1487.45			586.66	796.90	1310.31	
100.03	40.87		237.21					950.10	
222.31	1244.20	24.31	44.93			9.31	88.86	845.63	
**5.82**	**239.49**	**-19.07**	**28.93**	**-14.14**		**385.16**	**0.17**	**-1.01**	**0.32**

(C)1994-2024 China Academic Journal Electronic Publishing House. All rights reserved.   http://www.cnki.net

4-2 中国能源平衡表(实物量)-2001

		煤合计<br>Coal Total<br>万吨<br>10^4 tn	原煤<br>Raw Coal<br>万吨<br>10^4 tn
**一、可供本地区消费的能源量**	**Total Primary Energy Supply**	**108480.02**	**109648.00**
1.一次能源生产量	Indigenous Production	116078.00	116078.00
水 电	Hydro Power		
核 电	Nuclear Power		
2.回 收 能	Recovery of Energy		
3.进 口 量	Import	266.02	222.00
4.我轮、机在外国加油量	China Airplanes&Ships Refueling in Abroad		
5.出 口 量(-)	Export (-)	-9012.87	-7867.00
6.外轮、机在我国加油量(-)	Foreign Airplanes&Ships Refueling in China		
7.库存增(-)、减(+)量	Stock Change	1148.87	1215.00
**二、加工转换投入(-)产出(+)量**	**Input(-) & Output(+) of Transformation**	**-82320.07**	**-87755.00**
1.火力发电	Thermal Power	-57687.86	-56230.00
2.供 热	Heating Supply	-6961.49	-6724.00
3.洗 选 煤	Coal Washing	-1450.54	-18949.00
4.炼 焦	Coking	-15436.37	-4807.00
5.炼 油	Petroleum Refineries		
6.制 气	Gas Works	-893.81	-378.00
#焦炭再投入量(-)	Coke Input (-)		
7.煤制品加工	Briquettes	110.00	-667.00
**三、损 失 量**	**Loss**		
**四、终端消费量**	**Total Final Consumption**	**43891.27**	**39551.00**
1.农、林、牧、渔、水利业	Farming, Forestry, Animal Husbandry, Fishery & Water Conservancy	1599.64	1574.00
2.工 业	Industry	31287.92	28824.00
#用作原料、材料	Non-Energy Use	850.00	829.00
3.建 筑 业	Construction	537.98	521.00
4.交通运输、仓储及邮电通迅业	Transport, Storage, Postal & Telecommunications Services	1050.88	1023.00
5.批发和零售贸易业、餐饮业	Wholesale, Retail Trade and Catering Service	809.87	793.00
6.生活消费	Residential Consumption	7830.25	6205.00
城 镇	Urban	3048.36	2151.00
乡 村	Rural	4781.89	4054.00
7.其 他	Other	774.73	611.00
**五、平衡差额**	**Statistical Difference**	**-17731.32**	**-17658.00**

(C)1994-2024 China Academic Journal Electronic Publishing House. All rights reserved. http://www.cnki.net

ENERGY BANLANCE OF CHINA - 2001(PHYSICAL QUANTITY)

洗精煤<br>Cleaned Coal<br>万吨<br>10^4 tn	其他洗煤<br>Other Washed Coal<br>万吨<br>10^4 tn	型煤<br>Briquettes<br>万吨<br>10^4 tn	焦炭<br>Coke<br>万吨<br>10^4 tn	焦炉煤气<br>Coke Oven Gas<br>亿立方米<br>10^8 cu. m	其他煤气<br>Other Gas<br>亿立方米<br>10^8 cu. m	其他焦化产品<br>Other Coking Products<br>万吨<br>10^4 tn	油品合计<br>Petroleum Products Total<br>万吨<br>10^4 tn	原油<br>Crude Oil<br>万吨<br>10^4 tn
**-993.88**	**-173.39**	**-0.71**	**-1667.83**		**432.00**	**35.11**	**23204.67**	**21382.99**
							16395.87	16300.00
					432.00			
27.67	16.35					47.50	8769.15	7026.53
							349.00	
-1144.53	-1.04	-0.30	-1384.60			-12.39	-1906.96	-1030.60
							-139.72	
122.98	-188.70	-0.41	-283.23				-262.67	-912.94
**1237.50**	**3410.43**	**787.00**	**12977.12**	**230.35**	**47.00**	**203.94**	**-2291.99**	**-20404.30**
-193.36	-1264.50			-10.62	-84.02	-0.66	-1213.55	-85.00
-26.43	-211.06			-7.03	-40.12	-0.89	-438.66	-14.00
12560.46	4938.00							
-10600.44	-28.93		12891.26	245.00	11.40	191.00		
							-617.02	-20305.30
-502.73	-13.08		239.51	3.00	159.74	14.49	-22.76	
			-153.65					
	-10.00	787.00						
				**2.71**			**189.34**	**190.91**
**1028.06**	**2524.52**	**787.69**	**10845.58**	**231.55**	**478.83**	**230.82**	**20356.97**	**636.80**
	25.64		139.23				1568.48	
1020.07	1436.85	7.00	10484.75	191.09	390.75	230.82	9059.88	612.34
11.00	10.00		775.07			47.86	3960.11	97.58
3.98	13.00		23.91				372.34	3.30
3.90	23.93	0.05	11.68	0.02	0.25		5540.34	19.58
0.10	9.87	6.90	39.73	2.00	3.00		567.41	0.18
	855.03	770.22	134.20	35.37	83.61		1294.81	
	400.03	497.33	68.25	35.37	82.80		1025.73	
	455.00	272.89	65.95		0.81		269.08	
0.01	160.20	3.52	12.08	3.07	1.22		1953.71	1.40
**-784.44**	**712.52**	**-1.40**	**463.71**	**-3.91**	**0.17**	**8.23**	**366.37**	**150.98**

(C)1994-2024 China Academic Journal Electronic Publishing House. All rights reserved. http://www.cnki.net

续表(2001)

		汽油<br>Gasoline<br>万吨<br>10^4tn	煤油<br>Kerosene<br>万吨<br>10^4tn
**一、可供本地区消费的能源量**	**Total Primary Energy Supply**	**-547.80**	**104.88**
1.一次能源生产量	Indigenous Production		
水　电	Hydro Power		
核　电	Nuclear Power		
2.回 收 能	Recovery of Energy		
3.进 口 量	Import	0.02	201.89
4.我轮、机在外国加油量	China Airplanes&Ships Refueling in Abroad		96.70
5.出 口 量(-)	Export (-)	-572.46	-182.22
6.外轮、机在我国加油量(-)	Foreign Airplanes&ships Refueling in China	-13.50	-64.20
7.库存增(-)、减(+)量	Stock Change	38.14	52.71
**二、加工转换投入(-)产出(+)量**	**Input(-) & Output(+) of Transformation**	**4153.94**	**789.35**
1.火力发电	Thermal Power	-0.60	
2.供　热	Heating Supply	-0.12	
3.洗 选 煤	Coal Washing		
4.炼　焦	Coking		
5.炼　油	Petroleum Refineries	4154.66	789.35
6.制　气	Gas Works		
#焦炭再投入量(-)	Coke Input (-)		
7.煤制品加工	Briquettes		
**三、损 失 量**	**Loss**		
**四、终端消费量**	**Total Final Consumption**	**3597.03**	**890.27**
1.农、林、牧、渔、水利业	Farming, Forestry, Animal Husbandry, Fishery & Water Conservancy	190.60	1.52
2.工　业	Industry	617.42	86.00
#用作原料、材料	Non-Energy Use	8.46	0.18
3.建 筑 业	Construction	116.70	3.50
4.交通运输、仓储及邮电通迅业	Transport, Storage, Postal & Telecommunications Services	1419.37	560.69
5.批发和零售贸易业、餐饮业	Wholesale, Retail Trade and Catering Service	214.04	12.47
6.生活消费	Residential Consumption	134.60	75.00
城　镇	Urban	99.92	7.02
乡　村	Rural	34.68	67.98
7.其　他	Other	904.30	151.09
**五、平衡差额**	**Statistical Difference**	**9.11**	**3.96**

(C)1994-2024 China Academic Journal Electronic Publishing House. All rights reserved. http://www.cnki.net

Continued(2001)

柴油<br>Diesel Oil<br>万吨<br>10^4tn	燃料油<br>Fuel Oil<br>万吨<br>10^4tn	液化石油气<br>PLG<br>万吨<br>10^4tn	炼厂干气<br>Refinery Gas<br>万吨<br>10^4tn	其他石油制品<br>Other Petroleum Products<br>万吨<br>10^4tn	天然气<br>Natural Gas<br>亿立方米<br>10^8 cu. m	热力<br>Heat<br>万百万千焦<br>10^{10} kj	电力<br>Electricity<br>亿千瓦小时<br>10^8kwh	其他能源<br>Other Energy<br>万吨标煤<br>10^4tce
**-213.57**	**1972.47**	**475.70**		**-124.17**	**303.26**		**2865.11**	**616.19**
					303.29		2949.04	
							2774.32	
							174.72	
								616.19
27.47	1823.60	488.86		201.31			17.98	
27.22	225.08							
-25.62	-44.09	-2.09		-325.48	-0.03		-101.91	
-21.24	-40.78							
-221.40	8.66	-11.07						
**7245.21**	**703.47**	**951.28**	**550.00**	**3815.48**	**-29.41**	**153203.45**	**11767.53**	**-310.44**
-240.39	-838.54	-1.05	-23.00	-28.37	-13.00		11767.53	-138.61
	-299.62		-100.00	-26.60	-16.41	153203.45		-109.20
								0.20
7485.60	1864.39	952.33	673.00	3870.45				-62.83
	-22.76							
		**1.44**			**6.22**	**1795.58**	**1033.47**	
**6867.27**	**2689.30**	**1408.67**	**559.00**	**3691.31**	**238.67**	**151402.97**	**13599.99**	**305.43**
1375.64	0.42	0.30				54.41	762.39	
1396.42	1788.40	290.51	559.00	3691.31	186.94	123365.60	9411.19	305.43
9.24	95.72	9.50	44.00	3691.31	58.48			305.43
223.08	16.18	9.51			0.72	156.94	144.91	
2671.00	855.00	15.70			1.20	687.06	309.32	
268.07	12.28	60.40			5.00	1163.83	444.89	
79.17		1006.04			44.11	23369.40	1839.23	
51.12		867.67			44.11	23369.40	1066.16	
28.05		138.37					773.07	
853.89	17.02	26.21			0.70	2605.73	688.06	
164.37	-13.36	16.87	-9.00		28.96	4.90	-0.82	0.32

(C)1994-2024 China Academic Journal Electronic Publishing House. All rights reserved. http://www.cnki.net

4-3 中国能源平衡表(标准量)-2002

(万吨标准煤)

		能源合计 Energy Total	
		(发电煤耗计算法)(coal equivalent calculation)	(电热当量计算法)(calorific value calculation)
**一、可供本地区消费的能源量**	**Total Primary Energy Supply**	**144318.71**	**136756.00**
1.一次能源生产量	Indigenous Production	138368.81	130622.94
水电	Hydro Power	10663.45	3539.20
核电	Nuclear Power	930.43	308.81
2.回收能	Recovery of Energy	1907.98	1907.98
3.进口量	Import	15197.52	15140.61
4.我轮、机在外国加油量	China Airplanes&ships Refueling in Abroad	571.75	571.75
5.出口量(-)	Export (-)	-10796.14	-10556.07
6.外轮、机在我国加油量(-)	Foreign Airplanes&ships Refueling in China	-220.87	-220.87
7.库存增(-)、减(+)量	Stock Change	-710.34	-710
**二、加工转换投入(-)产出(+)量**	**Input(-) & Output(+) of Transformation**	**-2611.91**	**-36326.25**
1.火力发电	Thermal Power	2.99	-32835.28
2.供热	Heating Supply	-0.37	-879.43
3.洗选煤	Coal Washing	-952.50	-952.50
4.炼焦	Coking	-322.39	-322.39
5.炼油	Petroleum Refineries	-1015.43	-1015.43
6.制气	Gas Works	-144.39	-144.39
#焦炭再投入量(-)	Coke Input (-)	-133.42	-133.42
7.煤制品加工	Briquettes	-43.41	-43.41
**三、损失量**	**Loss**	**4763.06**	**1861.15**
**四、终端消费量**	**Total Final Consumption**	**140846.56**	**102466.70**
1.农、林、牧、渔、水利业	Farming, Forestry, Animal Husbandry, Fishery & Water Conservancy	6514.29	4593.60
2.工业	Industry	95143.39	68161.68
#用作原料、材料	Non-Energy Use	8687.29	8687.29
3.建筑业	Construction	1610.14	1203.09
4.交通运输、仓储及邮电通迅业	Transport, Storage, Postal & Telecommunications Services	10749.73	9909.21
5.批发和零售贸易业、餐饮业	Wholesale, Retail Trade and Catering Service	3464.02	2219.21
6.生活消费	Residential Consumption	17031.76	11937.90
城镇	Urban	10375.14	7340.78
乡村	Rural	6656.62	4597.12
7.其他	Other	6333.27	4442.02
**五、平衡差额**	**Statistical Difference**	**-3902.82**	**-3898.10**
**六、能源消费总量**	**Total Energy Consumption**	**148221.53**	**140654.10**

(C)1994-2024 China Academic Journal Electronic Publishing House. All rights reserved. http://www.cnki.net

ENERGY BALANCE OF CHINA－2002 (STANDARD QUANTITY)

(10^4tce)

煤合计 Coal Total	原煤 Raw Coal	洗精煤 Cleaned Coal	其他洗煤 Other Washed Coal	型煤 Briquettes	焦炭 Coke	焦炉煤气 Coke Oven Gas	其他煤气 Other Gas	其他焦化产品 Other Coking Products	油品合计 Petroleum Products Total
**92335.43**	**93464**	**-1121.40**	**-6.68**	**-0.08**	**-1382.12**		**1263.63**	**43.16**	**35750.62**
98573.40	98573								23857.62
							1263.63		
806.23	775	23.34	8.22	0.01				57.33	14248.78
									571.75
-6255.24	-5039	-1213.33	-2.74	-0.50	-1318.21			-14.17	-2849.19
									-220.87
-789	-846	69	-12		-64				143
**-69695.27**	**-72479**	**177.53**	**2102.69**	**503.98**	**13712.27**	**1486.68**	**126.32**	**246.63**	**-3408.46**
-46665.73	-45887	-152.68	-626.42			-84.04	-257.01		-1827.83
-5418.46	-5317	-3.56	-97.65			-29.67	-99.36		-613.88
-952.50	-16355	12549.24	2852.78						
-15809.81	-4090	-11706.21	-13.14		13631.47	1583.50	43.13	229.32	
									-939.74
-805.36	-288	-509.26	-7.63		214.22	16.89	439.56	17.31	-27.01
					-133.42				
-43.41	-542		-5.25	503.98					
**0**									**272.28**
**27685.81**	**25053**	**802.76**	**1322.76**	**507.45**	**11857.45**	**1496.13**	**1389.54**	**288.50**	**31849.14**
1058.46	1046		12.02		136.95				2441.96
19744.83	18182	797.04	761.54	3.86	11502.05	1248.52	1120.22	288.50	13814.03
582.02	566	10.11	5.78		778.50			63.47	6154.50
361.11	353	2.53	5.41		22.71				602.31
688.44	677	3.11	8.15	0.30	11.11	0.12	0.60		8745.14
527.47	517	0.08	5.26	5.45	41.38	10.37	11.13		883.23
4823.81	3879		449.66	495.50	131.26	220.84	257.59		2456.71
1775.76	1256		207.87	312.12	67.27	220.84	254.51		1997.83
3048.05	2623		241.79	183.38	63.99		3.08		458.88
481.69	399		80.72	2.34	11.99	16.28			2905.76
**-5045.65**	**-4069**	**-1746.63**	**773.25**	**-3.55**	**472.70**	**-9.45**	**0.41**	**1.29**	**220.74**

(C)1994-2024 China Academic Journal Electronic Publishing House. All rights reserved. http://www.cnki.net

续表(2002)

(万吨标准煤)

		原油<br>Crude Oil	汽油<br>Gasoline
**一、可供本地区消费的能源量**	**Total Primary Energy Supply**	**32527.78**	**-840.28**
1.一次能源生产量	Indigenous Production	23857.62	
水 电	Hydro Power		
核 电	Nuclear Power		
2.回收能	Recovery of Energy		0
3.进口量	Import	9915.40	
4.我轮、机在外国加油量	China Airplanes&ships Refueling in Abroad		0
5.出口量(-)	Export (-)	-1094.96	-900.79
6.外轮、机在我国加油量(-)	Foreign Airplanes&ships Refueling in China		-26.79
7.库存增(-)、减(+)量	Stock Change	-150.28	87.30
**二、加工转换投入(-)产出(+)量**	**Input(-) & Output(+) of Transformation**	**-30959.63**	**6356.48**
1.火力发电	Thermal Power	-111.90	-0.91
2.供 热	Heating Supply	-18.23	-0.18
3.洗选煤	Coal Washing		0
4.炼 焦	Coking		0
5.炼 油	Petroleum Refineries	-30829.50	6357.57
6.制 气	Gas Works		0
#焦炭再投入量(-)	Coke Input (-)		0
7.煤制品加工	Briquettes		0
**三、损失量**	**Loss**	**269.26**	
**四、终端消费量**	**Total Final Consumption**	**973.24**	**5516.22**
1.农、林、牧、渔、水利业	Farming, Forestry, Animal Husbandry, Fishery & Water Conservancy		276.52
2.工 业	Industry	940.02	928.31
#用作原料、材料	Non-Energy Use	160.19	13.24
3.建筑业	Construction	6.00	179.98
4.交通运输、仓储及邮电通迅业	Transport, Storage, Postal & Telecommunications Services	25.21	2212.25
5.批发和零售贸易业、餐饮业	Wholesale, Retail Trade and Catering Service	0.17	329.92
6.生活消费	Residential Consumption		241.01
城 镇	Urban		176.42
乡 村	Rural		64.59
7.其 他	Other	1.84	1348.23
**五、平衡差额**	**Statistical Difference**	**325.65**	**-0.02**
**六、能源消费总量**	**Total Energy Consumption**		

(C)1994-2024 China Academic Journal Electronic Publishing House. All rights reserved. http://www.cnki.net

Continued(2002)

(10^4 tce)

煤油<br>Kerosene	柴油<br>Diesel Oil	燃料油<br>Fuel Oil	液化石油气<br>PLG	炼厂干气<br>Refinery Gas	其他石油制品<br>Other Petroleum Products	天然气<br>Natural Gas	热力<br>Heat	电力<br>Electricity	其他能源<br>Other Energy
**130.47**	**23.64**	**2653.34**	**1074.57**		**181.10**	**4343.91**		**3757.02**	**644.35**
						4343.91		3848.01	
								3539.20	
								308.81	
									644.35
315.66	69.53	2370.99	1073.43		503.77			28.27	
161.56	45.17	365.02							
-249.61	-180.23	-91.30	-9.63		-322.67			-119.26	
-104.91	-30.60	-58.57							
7.77	119.77	67.20	10.77						
**1215.54**	**10899.27**	**924.69**	**1774.73**	**883.80**	**5496.66**	**-373.34**	**5598.91**	**16313.46**	**-333.45**
	-329.29	-1303.83	-2.64	-31.90	-47.36	-146.97		16313.46	-167.16
		-380.95		-177.82	-36.70	-226.37	5598.91		-90.60
1215.54	11228.56	2636.48	1777.37	1093.52	5580.72				-75.69
		-27.01							
**0.00**			**3.02**			**84.19**	**68.40**	**1436.28**	
**1352.52**	**10842.51**	**3822.43**	**2768.76**	**895.70**	**5677.76**	**3423.94**	**5530.32**	**18635.07**	**310.80**
2.06	2162.79	0.59					2.24	953.99	
128.53	2193.43	2503.81	546.47	895.70	5677.76	2632.59	4442.63	13057.51	310.80
3.87	37.88	165.53	25.71	70.32	5677.76	798.00			310.80
	367.17	27.29	21.87			9.04	6.19	201.73	
907.49	4320.02	1245.88	34.29			20.75	27.65	415.40	
19.13	409.14	17.57	107.30			81.13	50.00	614.50	
89.31	122.28		2004.11			680.43	907.51	2459.75	
9.30	86.68		1725.43			680.43	907.51	1436.63	
80.01	35.60		278.68					1023.12	
206.00	1267.68	27.29	54.72				94.10	932.20	
**-6.51**	**80.40**	**-244.40**	**77.52**	**-11.90**		**462.44**	**0.19**	**-0.87**	**0.10**

(C)1994-2024 China Academic Journal Electronic Publishing House. All rights reserved. http://www.cnki.net

4-3 中国能源平衡表(实物量)-2002

		煤合计<br>Coal Total<br>万吨<br>10^4 tn	原煤<br>Raw Coal<br>万吨<br>10^4 tn
**一、可供本地区消费的能源量**	**Total Primary Energy Supply**	**129604.75**	**130846.41**
1.一次能源生产量	Indigenous Production	138000.00	138000.00
水　电	Hydro Power		
核　电	Nuclear Power		
2.回 收 能	Recovery of Energy		
3.进 口 量	Import	1125.74	1084.50
4.我轮、机在外国加油量	China Airplanes&Ships Refueling in Abroad		
5.出 口 量(-)	Export (-)	-8389.56	-7053.99
6.外轮、机在我国加油量(-)	Foreign Airplanes&Ships Refueling in China		
7.库存增(-)、减(+)量	Stock Change	-1131.43	-1184.10
**二、加工转换投入(-)产出(+)量**	**Input(-) & Output(+) of Transformation**	**-93913.12**	**-98761.68**
1.火力发电	Thermal Power	-65600.03	-64240.00
2.供　热	Heating Supply	-7473.73	-7283.90
3.洗 选 煤	Coal Washing	-1717.46	-20900.35
4.炼　焦	Coking	-18209.72	-5177.80
5.炼　油	Petroleum Refineries		
6.制　气	Gas Works	-973.20	-400.65
#焦炭再投入量(-)	Coke Input (-)		
7.煤制品加工	Briquettes	61.02	-758.98
**三、损 失 量**	**Loss**		
**四、终端消费量**	**Total Final Consumption**	**42692.41**	**38305.78**
1.农、林、牧、渔、水利业	Farming, Forestry, Animal Husbandry, Fishery & Water Conservancy	1622.89	1600.00
2.工　业	Industry	30282.23	27800.88
#用作原料、材料	Non-Energy Use	889.61	865.61
3.建 筑 业	Construction	553.55	540.00
4.交通运输、仓储及邮电通迅业	Transport, Storage, Postal & Telecommunications Services	1054.96	1034.95
5.批发和零售贸易业、餐饮业	Wholesale, Retail Trade and Catering Service	809.08	790.00
6.生活消费	Residential Consumption	7602.64	5930.45
城　镇	Urban	2829.89	1920.07
乡　村	Rural	4772.75	4010.38
7.其　他	Other	767.06	609.50
**五、平衡差额**	**Statistical Difference**	**-7000.78**	**-6221.05**

(C)1994-2024 China Academic Journal Electronic Publishing House. All rights reserved. http://www.cnki.net

ENERGY BANLANCE OF CHINA－2002(PHYSICAL QUANTITY)

洗精煤 Cleaned Coal 万吨 10^4 tn	其他洗煤 Other Washed Coal 万吨 10^4 tn	型煤 Briquettes 万吨 10^4 tn	焦炭 Coke 万吨 10^4 tn	焦炉煤气 Coke Oven Gas 亿立方米 10^8 cu.m	其他煤气 Other Gas 亿立方米 10^8 cu.m	其他焦化产品 Other Coking Products 万吨 10^4 tn	油品合计 Petroleum Products Total 万吨 10^4 tn	原油 Crude Oil 万吨 10^4 tn
**－1228.79**	**－12.73**	**－0.14**	**－1422.81**		**439.40**	**37.40**	**24925.09**	**22768.99**
							16700.00	16700.00
					439.40			
25.58	15.65	0.01				49.68	9873.03	6940.64
							396.31	
－1329.53	－5.22	－0.82	－1357.02			－12.28	－1987.68	－766.46
							－151.51	
75.16	－23.16	0.67	－65.79				94.94	－105.19
**14.96**	**4003.60**	**830.00**	**14115.99**	**243.83**	**43.97**	**213.72**	**－2606.76**	**－21671.31**
－167.30	－1192.73			－13.68	－89.37		－1275.57	－78.33
－3.90	－185.93			－4.83	－34.55		－420.70	－12.76
13751.09	5431.80							
－13006.90	－25.02		14032.81	259.59	15.00	198.72		
							－891.58	－21580.22
－558.03	－14.52		220.53	2.75	152.89	15.00	－18.91	
			－137.35					
	－10.00	830.00						
							**190.24**	**188.48**
**1032.35**	**2518.58**	**835.70**	**12206.56**	**245.38**	**483.23**	**250.00**	**21982.83**	**681.26**
	22.89		140.98				1674.05	
1025.00	1450.00	6.35	11840.70	204.77	389.57	250.00	9854.51	658.00
13.00	11.00		801.42			55.00	4656.90	112.13
3.25	10.30		23.38				410.37	4.20
4.00	15.51	0.50	11.44	0.02	0.21		5994.81	17.65
0.10	10.01	8.97	42.60	1.70	3.87		593.02	0.12
	856.17	816.02	135.12	36.22	89.58		1477.47	
	395.80	514.02	69.25	36.22	88.51		1192.20	
	460.37	302.00	65.87		1.07		285.27	
	153.70	3.86	12.34	2.67			1978.60	1.29
**－2246.18**	**1472.29**	**－5.84**	**486.62**	**－1.55**	**0.14**	**1.12**	**145.26**	**227.94**

(C)1994-2024 China Academic Journal Electronic Publishing House. All rights reserved. http://www.cnki.net

续表(2002)

		汽油<br>Gasoline<br>万吨<br>10^4tn	煤油<br>Kerosene<br>万吨<br>10^4tn
**一、可供本地区消费的能源量**	**Total Primary Energy Supply**	**-571.08**	**88.67**
1.一次能源生产量	Indigenous Production		
水　电	Hydro Power		
核　电	Nuclear Power		
2.回 收 能	Recovery of Energy		
3.进 口 量	Import		214.53
4.我轮、机在外国加油量	China Airplanes&Ships Refueling in Abroad		109.80
5.出 口 量(-)	Export (-)	-612.20	-169.64
6.外轮、机在我国加油量(-)	Foreign Airplanes&ships Refueling in China	-18.21	-71.30
7.库存增(-)、减(+)量	Stock Change	59.33	5.28
**二、加工转换投入(-)产出(+)量**	**Input(-) & Output(+) of Transformation**	**4320.02**	**826.11**
1.火力发电	Thermal Power	-0.62	
2.供　热	Heating Supply	-0.12	
3.洗 选 煤	Coal Washing		
4.炼　焦	Coking		
5.炼　油	Petroleum Refineries	4320.76	826.11
6.制　气	Gas Works		
#焦炭再投入量(-)	Coke Input (-)		
7.煤制品加工	Briquettes		
**三、损 失 量**	**Loss**		
**四、终端消费量**	**Total Final Consumption**	**3748.96**	**919.20**
1.农、林、牧、渔、水利业	Farming, Forestry, Animal Husbandry, Fishery & Water Conservancy	187.93	1.40
2.工　业	Industry	630.90	87.35
#用作原料、材料	Non-Energy Use	9.00	2.63
3.建 筑 业	Construction	122.32	
4.交通运输、仓储及邮电通迅业	Transport, Storage, Postal & Telecommunications Services	1503.50	616.75
5.批发和零售贸易业、餐饮业	Wholesale, Retail Trade and Catering Service	224.22	13.00
6.生活消费	Residential Consumption	163.80	60.70
城　镇	Urban	119.90	6.32
乡　村	Rural	43.90	54.38
7.其　他	Other	916.29	140.00
**五、平衡差额**	**Statistical Difference**	**-0.02**	**-4.42**

(C)1994-2024 China Academic Journal Electronic Publishing House. All rights reserved. http://www.cnki.net

Continued(2002)

柴油<br>Diesel Oil<br>万吨<br>10^4tn	燃料油<br>Fuel Oil<br>万吨<br>10^4tn	液化石油气<br>PLG<br>万吨<br>10^4tn	炼厂干气<br>Refinery Gas<br>万吨<br>10^4tn	其他石油制品<br>Other Petroleum Products<br>万吨<br>10^4tn	天然气<br>Natural Gas<br>亿立方米<br>10^8 cu. m	热力<br>Heat<br>万百万千焦<br>10^{10} kj	电力<br>Electricity<br>亿千瓦小时<br>10^8kwh	其他能源<br>Other Energy<br>万吨标煤<br>10^4tce
**16.23**	**1857.30**	**626.82**		**138.16**	**326.61**		**3056.97**	**644.35**
					326.61		3131.01	
							2879.74	
							251.27	
								644.35
47.72	1659.66	626.16		384.32			23.00	
31.00	255.51							
-123.69	-63.91	-5.62		-246.16			-97.04	
-21.00	-41.00							
82.20	47.04	6.28						
**7480.11**	**647.27**	**1035.25**	**562.43**	**4193.36**	**-28.07**	**164190.77**	**13273.77**	**-333.45**
-225.99	-912.66	-1.54	-20.30	-36.13	-11.05		13273.77	-167.16
	-266.66		-113.16	-28.00	-17.02	164190.77		-90.60
7706.10	1845.50	1036.79	695.89	4257.49				-75.69
	-18.91							
		**1.76**			**6.33**	**2005.84**	**1168.66**	
**7441.16**	**2675.64**	**1615.09**	**570.00**	**4331.52**	**257.44**	**162179.59**	**15162.79**	**310.80**
1484.31	0.41					65.83	776.23	
1505.34	1752.63	318.77	570.00	4331.52	197.94	130282.41	10624.50	310.80
26.00	115.87	15.00	44.75	4331.52	60.00			310.80
251.99	19.10	12.76			0.68	181.67	164.14	
2964.81	872.10	20.00			1.56	810.73	338.00	
280.79	12.30	62.59			6.10	1466.42	500.00	
83.92		1169.05			51.16	26613.07	2001.42	
59.49		1006.49			51.16	26613.07	1168.94	
24.43		162.56					832.48	
870.00	19.10	31.92				2759.46	758.50	
**55.18**	**-171.07**	**45.22**	**-7.57**		**34.77**	**5.34**	**-0.71**	**0.10**

(C)1994-2024 China Academic Journal Electronic Publishing House. All rights reserved. http://www.cnki.net

4-4 综合能源平衡表

单位：万吨标准煤

项目	Item	1980	1985
**可供消费的能源总量**	**Total Energy Available for Consumption**	**61557**	**77603**
一次能源生产量	Primary Energy Output	63735	85546
回收能	Recovery of Energy		
进口量	Imports	261	340
出口量(-)	Exports (-)	3058	5774
年初年末库存差额	Stock Changes in the Year	619	-2509
**能源消费总量**	**Total Energy Consumption**	**60275**	**76682**
在总量中：	Consumption by Sector		
1.农、林、牧、渔、水利业	Farming, Forestry, Animal Husbandry, Fishery and Water Conservancy	4692	4045
2.工业	Industry	38986	51068
3.建筑业	Construction	957	1302
4.交通运输和邮电通讯业	Transportation, Post and Telecommunications Services	2902	3713
5.商业、饮食、物资供销和仓储业	Commerce, Catering Services, Materials Supply, Marketing and Storage	518	766
6.其他	Others	1205	2470
7.生活消费	Residential Consumption	11015	13318
在总量中：	Consumption by Usage		
(一)终端消费	(I)Final Consumption	57508	73586
#工业	Industry	38293	48021
(二)加工转换损失量	(II)Losses in Processing and Transformation	1358	1491
#炼焦	Coking	644	572
炼油	Petroleum Refining	113	110
(三)损失量	(III)Other Losses	1409	1605
**平衡差额**	**Balance**	**1282**	**921**

注：1.村办工业包括在工业中(下同)。

2.电力、热力按等价热值折算，因此加工转换损失量中不包括发电、供热损失量。

3.进口量包括我国飞机、轮船在国外加油量；出口量包括外国飞机、轮船在我国加油量。

(C)1994-2024 China Academic Journal Electronic Publishing House. All rights reserved. http://www.cnki.net

OVERALL ENERGY BALANCE

(10 000 tce)

1990	1995	1996	1997	1998	1999	2000	2001	2002
**96138**	**129535**	**134433**	**133724**	**128368.34**	**115829**	**115150**	**125310**	**144319**
103922	129034	132616	132410	124249.57	109126	106988	120900	138369
	2312	1891	467	1919.72	1694	1760	1859	1908
1310	5456	6837	9964	8474.15	9513	14331	13471	15769
5875	6776	7529	7663	7153.44	6477	9026	11145	11017
-3219	-491	618	-1453	878.34	1974	1097	225	-710
**98703**	**131176**	**138948**	**138173**	**132213.92**	**130119**	**130297**	**134915**	**148222**
4852	5505	5717	5905	5790.32	5832	5787	6233	6514
67578	96191	100322	100080	94409.15	90797	89634	92347	102181
1213	1335	1449	1179	1612.09	1381	1433	1453	1610
4541	5863	5994	7543	8245.02	9243	9917	10257	11086
1247	2018	2268	2394	2552.08	2812	2893	3165	3464
3473	4519	5484	4703	5212.56	5502	5722	6034	6333
15799	15745	17714	16368	14392.7	14552	14912	15427	17032
94289	124252	132731	130585	126038.66	124096	124032	128951	140847
63239	89473	94342	92749	88521.91	85095	83707	86711	95143
2264	3634	2903	3915	2629.15	2336	2371	2011	2612
905		919	1297	684.24	544	487	387	322
326		549	626	616.19	648	781	636	1015
2150	3289	3314	3672	3546.11	3688	3893	3953	4763
**-2565**	**-1641**	**-4515**	**-4449**	**-3845.58**	**-14290**	**-15147**	**-9605**	**-3903**

a) Data on industry include the data of village-run industry. (The sane as in the following tables).

b) Electric power and heat are converted on the basic of equal caloric value. Therefore, losses in processing and transformation exclude losses in power generation and heating.

c) Data on imports include the petroleum consumed by the Chinese airplanes and ships in refueling abroad. Data on exports include the petroleum consumed by the foreign airplanes and ships in refueling in China.

(C)1994-2024 China Academic Journal Electronic Publishing House. All rights reserved. http://www.cnki.net

4－5 煤炭平衡表

单位：万吨

项　目	Item	1980	1985
**可供量**	**Total Energy Available for Consumption**	**62601.0**	**82776.6**
生产量	Output	62015.0	87228.4
进口量	Imports	199.0	230.7
出口量(－)	Exports (－)	632.0	777.0
年初年末库存差额	Stock Changes in the Year	1019.0	－3905.5
**消费量**	**Total Energy Consumption**	**61009.5**	**81603.0**
在消费量中：	Consumption by Sector		
1. 农、林、牧、渔、水利业	Farming, Forestry, Animal Husbandry, Fishery and Water Conservancy	1550.3	2208.6
2. 工　业	Industry	43848.4	58613.3
3. 建筑业	Construction	556.0	531.9
4. 交通运输和邮电通讯业	Transportation, Postal and Telecommunications Services	1934.4	2307.1
5. 商业、饮食、物资供销和仓储业	Commerce, Catering Services, Materi－als Supply, Marketing and Storage	455.2	738.2
6. 其他	Other	1091.2	1579.5
7. 生活消费	Residential Consumption	11574.0	15624.4
在消费量中：	Consumption by Usage		
(一) 终端消费	(1)Final Consumption	38804.2	52704.4
#工业	Industry	21643.1	29715.0
(二) 中间消费	(2)Intermediate Consumption		
(用于加工转换)	(Consumed in Transformation)	19461.6	25397.4
发　电	Power Generation	12648.4	16440.7
供　热	Heating		1462.3
炼　焦	Coking	6682.2	7303.8
制　气	Gas Production	131.0	190.6
(三) 洗选损耗	(3) Losses in Coal Washing and Dressing	2743.7	3501.2
**平衡差额**	**Balance**	**1591.5**	**1173.6**

注：生产量为原煤产量。

(C)1994-2024 China Academic Journal Electronic Publishing House. All rights reserved.　http://www.cnki.net

COAL BALANCE SHEET

(10 000 tons)

1990	1995	1996	1997	1998	1999	2000	2001	2002
**102221.0**	**133461.7**	**137211.9**	**133159.0**	**122810.6**	**103576.1**	**98176.1**	**108480.0**	**129604.8**
107988.3	136073.1	139669.9	137282.0	125000.0	104500.0	99800.0	116078.0	138000.0
200.3	163.5	321.7	201.0	158.6	167.3	217.9	266.0	1125.7
1729.0	2861.7	3648.4	3073.0	3229.7	3743.9	5506.5	9012.9	8389.6
-4238.5	86.8	868.7	-1251.0	881.7	2652.7	3664.7	1148.9	-1131.4
**105523.0**	**137676.5**	**144734.4**	**139248.0**	**129492.2**	**126365.3**	**124537.4**	**126211.3**	**136605.5**
2095.2	1856.7	1917.3	1927.0	1923.3	1735.6	1647.7	1599.6	1622.9
81090.9	117570.7	123885.9	121671.0	114952.4	112757.3	111730.0	113608.0	124195.4
437.6	439.8	446.4	383.0	611.6	522.5	536.8	538.0	553.5
2160.9	1315.1	1175.9	1431.0	1390.6	1294.3	1139.9	11050.9	1055.0
1058.3	977.4	1074.3	863.0	947.6	896.2	814.6	809.9	809.1
1980.4	1986.7	1835.3	735.0	782.7	751.1	761.0	774.7	767.1
16699.7	13530.1	14399.3	12238.0	8884.0	8408.4	7907.0	7830.3	7602.6
60205.9	66156.1	68453.5	61792.0	56347.1	51572.2	46086.8	43891.3	42692.4
35773.8	46050.3	67604.9	44214.0	41807.3	37964.2	33279.7	31287.9	30282.2
41257.8	69487.6	74212.1	77451.0	73145.1	74793.1	78450.6	82320.1	93913.1
27204.3	44440.2	48808.6	48979.0	49489.3	51163.5	54611.2	57687.9	65600.0
2995.5	5887.3	6365.7	6245.0	6319.9	6473.0	6692.1	6961.5	7473.7
10697.6	18396.4	18455.8	19297.0	15628.1	14941.7	15000.4	15436.4	18209.7
360.4	763.7	582.0	733.0	685.1	847.6	810.0	893.8	973.2
4059.3	2032.8	2068.8	2311.0	1159.3	1491.5	1441.2	1450.5	1717.5
**-3302.0**	**-4214.8**	**-7522.5**	**-6089.0**	**-6681.6**	**-22789.2**	**-26361.3**	**-17731.3**	**-7000.8**

a) Data on output refer to the output of raw coal.

(C)1994-2024 China Academic Journal Electronic Publishing House. All rights reserved. http://www.cnki.net

4－6 焦炭平衡表

单位：万吨

项　　目	Item	1980	1985
**可供量**	**Total Energy Available for Consumption**	**4315.3**	**4689.7**
生产量	Output	4343.0	4802.1
进口量	Imports		2.1
出口量(－)	Exports (－)	27.1	36.9
年初年末库存差额	Stock Changes in the Year	－0.6	－77.6
**消费量**	**Total Energy Consumption**	**4303.0**	**4689.7**
在消费量中：	Consumption by Sector		
1．农、林、牧、渔、水利业	Farming, Forestry, Animal Husbandry, Fishery and Water Conservancy	10.6	20.8
2．工　业	Industry	4266.7	4627.7
3．建筑业	Construction	11.9	7.8
4．交通运输和邮电通讯业	Transportation, Postal and Telecommunications Services	8.2	5.7
5．商业、饮食、物资供销和仓储业	Commerce, Catering Services, Materi－als Supply, Marketing and Storage	0.9	2.7
6．其他	Other	4.7	2.0
7．生活消费	Residential Consumption		23.0
在消费量中：	Consumption by Usage		
(一)终端消费	(1)Final Consumption	4294.7	4677.9
＃工业	Industry	4258.4	4615.9
(二)中间消费	(2)Intermediate Consumption		
(用于加工转换)	(Consumed in Transformation)	8.3	11.8
制气	Gas Production	8.3	11.8
(三)损失量	(3) Losses in Coal Washing and Dressing		
**平衡差额**	**Balance**	**12.3**	

(C)1994-2024 China Academic Journal Electronic Publishing House. All rights reserved.   http://www.cnki.net

COKE BALANCE SHEET

(10 000 tons)

1990	1995	1996	1997	1998	1999	2000	2001	2002
**7085.8**	**12207.1**	**12726.8**	**12631.2**	**11733.5**	**10970.6**	**10892.3**	**11462.9**	**12830.5**
7328.3	13424.5	13595.6	13653.1	12899.1	12073.7	12184.0	13130.8	14253.3
	0.1	0.1	0.1					
129.0	886.1	768.6	1058.1	1146.4	997.4	1519.7	1384.6	1357.0
-113.5	-331.4	-100.3	36.1	-19.2	-105.7	228.0	-283.2	-65.8
**6914.7**	**10725.3**	**10797.9**	**10927.0**	**11078.2**	**10460.5**	**10440.0**	**10999.2**	**12343.9**
60.1	128.6	119.8	144.7	151.4	145.8	144.2	139.2	141.0
6808.8	10412.0	10491.1	10584.3	10710.3	10100.9	10080.5	10638.4	11978.1
5.2	10.8	13.9	12.5	14.6	17.1	19.0	23.9	23.4
4.1	10.1	6.6	6.5	10.3	10.1	11.2	11.7	11.4
7.7	25.7	28.3	35.2	38.8	36.5	35.7	39.7	42.6
1.9	6.4	12.0	19.2	12.8	13.0	12.2	12.1	12.3
26.9	131.6	126.2	124.7	140.0	143.1	137.2	134.2	135.1
6846.3	10648.0	10750.4	10848.9	11008.6	10302.7	10297.1	10845.6	12206.6
6740.4	10334.7	10443.5	10506.2	10640.6	9937.1	9937.7	10484.8	11840.7
68.4	77.3	47.6	78.1	69.7	157.8	142.9	153.7	137.4
68.4	77.3	47.6	78.1	69.7	157.8	142.9	153.7	137.4
**171.1**	**1481.8**	**1928.9**	**1704.2**	**655.2**	**510.1**	**452.3**	**463.7**	**486.6**

(C)1994-2024 China Academic Journal Electronic Publishing House. All rights reserved. http://www.cnki.net

4－7 石油平衡表

单位：万吨

项　　目	Item	1980	1985
**可供量**	**Total Energy Available for Consumption**	**8794.5**	**9193.7**
生产量	Output	10594.6	12489.5
进口量	Imports	82.7	90.0
出口量(－)	Exports (－)	1806.2	3630.4
年初年末库存差额	Stock Changes in the Year	－76.6	244.6
**消费量**	**Total Energy Consumption**	**8757.4**	**9168.8**
在消费量中：	Consumption by Sector		
1. 农、林、牧、渔、水利业	Farming, Forestry, Animal Husbandry, Fishery and Water Conservancy	814..9	758.7
2. 工　业	Industry	6203.2	6171.4
3. 建筑业	Construction	175.2	292.2
4. 交通运输和邮电通讯业	Transportation, Postal and Telecommunications Services	911.5	1176.4
5. 商业、饮食、物资供销和仓储业	Commerce, Catering Services, Materi－als Supply, Marketing and Storage	29.0	38.1
6. 其他	Other	481.7	506.1
7. 生活消费	Residential Consumption	141.9	225.9
在消费量中：	Consumption by Usage		
(一) 终端消费	(I)Final Consumption	6311.0	7063.3
#工　业	Industry	3780.3	4462.0
(二) 中间消费	(II)Intermediate Consumption		
(用于加工转换)	(Consumed in Transformation)	2102.1	1745.6
发　电	Power Generation	2065.4	1425.5
供　热	Heating		285.6
制　气	Gas Production	36.7	34.5
(三) 炼油损失量	(III)Losses in Petroleum Refining	81.5	112.9
(四) 损失量	Other Losses	262.8	247.0
**平衡差额**	**Balance**	**37.1**	**24.9**

注：1.生产量为原油产量。

2.进口量包括我国飞机、轮船在国外加油量；出口量包括外国飞机、轮船在我国加油量。

(C)1994-2024 China Academic Journal Electronic Publishing House. All rights reserved. http://www.cnki.net

PETROLEUM BALANCE

(10 000 tons)

1990	1995	1996	1997	1998	1999	2000	2001	2002
**11435.0**	**16072.7**	**17656.1**	**19653.8**	**19686.1**	**20964.4**	**22631.8**	**23204.7**	**24925.1**
13830.6	15005.0	15733.4	16074.1	16100.0	16000.0	16300.0	16395.9	16700.0
755.6	3673.2	4536.9	6787.0	5738.7	6483.3	9748.5	9118.2	10269.3
3110.4	2454.5	2696.0	2815.2	2326.5	1643.5	2172.1	2046.7	2139.2
-40.8	-151.0	81.8	-392.2	174.0	124.6	-1244.6	-262.7	94.9
**11485.6**	**16064.9**	**17436.2**	**19691.7**	**19817.8**	**21072.9**	**22439.3**	**22838.3**	**24779.8**
1033.6	1203.2	1223.7	1256.3	1294.7	1422.1	1496.9	1568.5	1674.1
7321.6	9349.3	10020.2	11304.2	10870.8	10852.8	11404.8	11388.6	12489.6
327.3	242.8	257.8	285.3	293.9	323.0	344.3	372.3	410.4
1683.2	2863.6	2937.7	3733.0	4245.3	5004.3	5509.5	5692.9	6156.7
77.6	333.9	367.9	416.6	426.0	537.2	545.0	567.4	593.0
757.8	1390.3	1752.3	1758.0	1704.8	1800.3	1882.9	1953.7	1978.6
284.5	682.0	876.6	938.3	983.3	1133.1	1256.5	1294.8	1477.5
9304.7	13676.3	15302.3	17051.9	17514.3	18664.7	19893.5	20357.0	21982.8
5180.4	7095.5	8022.5	8814.1	8717.3	8596.4	9016.2	9059.9	9854.5
1630.4	2230.0	1701.4	2449.7	2106.1	2222.2	2352.9	2292.0	2606.8
1234.4	1358.5	1238.6	1662.1	1304.8	1228.6	1178.2	1213.6	1275.6
356.3	399.9	404.8	356.5	455.5	394.6	427.0	438.7	420.7
39.7	51.6	58.2	49.2	35.4	32.5	25.9	22.8	18.9
295.8	420.1	271.1	382.0	310.4	566.6	721.9	617.0	891.6
254.7	158.6	161.4	190.1	197.3	186.0	192.9	189.3	190.2
**-50.6**	**7.8**	**219.9**	**-37.9**	**-131.5**	**-108.5**	**192.5**	**366.4**	**145.3**

a) Data on output refer to the output of crude oil.

b) Data on imports include the petroleum consumed by the Chinese airplanes and ships in refueling abroad. Data on exports include the petroleum consumed by the foreign airplanes and ships in refueling in China.

(C)1994-2024 China Academic Journal Electronic Publishing House. All rights reserved. http://www.cnki.net

4－8 原油平衡表

单位：万吨

项　　目	Item	1980	1985
**可供量**	**Total Energy Available for Consumption**	**9222.9**	**9516.5**
生产量	Output	10594.6	12489.5
进口量	Imports	36.6	
出口量(－)	Exports (－)	1330.9	3003.0
年初年末库存差额	Stock Changes in the Year	－77.4	30.0
**消费量**	**Total Energy Consumption**	**9205.0**	**9509.5**
在消费量中：	Consumption by Sector		
1. 农、林、牧、渔、水利业	Farming, Forestry, Animal Husbandry, Fishery and Water Conservancy	8.0	0.8
2. 工　业	Industry	9112.0	9389.9
3. 建筑业	Construction	28.8	74.0
4. 交通运输和邮电通讯业	Transportation, Postal and Telecommunications Services	50.1	44.3
5. 商业、饮食、物资供销和仓储业	Commerce, Catering Services, Materi－als Supply, Marketing and Storage		0.1
6. 其他	Other	6.1	0.4
7. 生活消费	Residential Consumption		
在消费量中：	Consumption by Usage		
(一) 终端消费	(I)Final Consumption	499.6	350.4
#工业	Industry	429.7	254.9
(二) 中间消费	(II)Intermediate Consumption		
(用于加工转换)	(Consumed in Transformation)	8443.0	8929.7
发　电	Power Generation	574.0	279.5
供　热	Heating		61.3
炼　油	Petroleum Refineries	7869.0	8588.9
(三) 油田原油损失量	(III)Losses in Oil Field for Crude Oil	262.4	229.4
**平衡差额**	**Balance**	**17.9**	**7.0**

(C)1994-2024 China Academic Journal Electronic Publishing House. All rights reserved. http://www.cnki.net

CRUDE OIL BALANC

(10 000 tons)

1990	1995	1996	1997	1998	1999	2000	2001	2002
**11770.6**	**14794.9**	**15920.7**	**17499.6**	**17316.9**	**18947.2**	**21383.0**	**21537.2**	**22769.0**
13830.6	15004.4	15733.4	16074.1	16100.0	16000.0	16300.0	16395.9	16700.0
292.3	1709.0	2261.7	3547.0	2732.0	3661.4	7026.5	6026.0	6940.6
2399.0	1822.7	2040.3	1982.9	1560.0	716.7	1030.6	755.0	766.5
46.7	-95.8	-34.1	-138.6	44.9	2.6	-912.9	-129.7	-105.2
**11762.2**	**14886.4**	**15865.0**	**17367.2**	**17395.3**	**18949.5**	**21232.0**	**21342.7**	**22541.1**
0.2	10.1	11.0						
11653.8	14716.3	15690.9	17197.8	17222.5	18775.2	21052.1	21168.2	22357.5
55.2	2.7	2.6	3.0	2.2	3.2	3.3	3.4	4.2
52.1	156.8	159.0	164.7	168.9	169.5	175.0	169.8	177.9
0.3	0.5	0.4	0.3	0.2	0.2	0.2	0.2	0.1
0.6	1390.3	1.2	1.4	1.5	1.4	1.4	1.2	1.3
402.1	309.9	492.2	492.4	518.7	519.4	636.8	654.1	681.3
333.4	274.7	452.9	471.0	495.4	495.4	612.3	630.8	658.0
11106.9	14419.4	15212.9	16686.4	16680.7	18245.7	20404.3	20500.7	21671.3
124.6	61.6	68.2	64.3	74.4	80.2	85.0	81.6	78.3
21.1	4.4	10.8	14.9	24.5	12.9	14.0	12.3	12.8
10961.2	14353.4	15133.9	16607.3	16581.8	18152.6	20305.3	20406.8	21580.2
253.2	157.1	159.9	188.4	196.0	184.4	190.9	187.9	188.5
**8.4**	**-91.5**	**55.7**	**132.4**	**-78.4**	**-2.2**	**151.0**	**194.4**	**227.9**

(C)1994-2024 China Academic Journal Electronic Publishing House. All rights reserved. http://www.cnki.net

4－9 燃料油平衡表

单位：万吨

项 目	Item	1980	1985
**可供量**	**Total Energy Available for Consumption**	**3096.1**	**2848.0**
生产量	Output	3142.0	2835.8
进口量	Imports	39.0	70.0
出口量(－)	Exports (－)	45.4	64.9
年初年末库存差额	Stock Changes in the Year	－39.5	7.1
**消费量**	**Total Energy Consumption**	**3073.7**	**2837.4**
在消费量中：	Consumption by Sector		
1. 农、林、牧、渔、水利业	Farming, Forestry, Animal Husbandry, Fishery and Water Conservancy	2.3	3.1
2. 工 业	Industry	2937.4	2662.2
3. 建筑业	Construction	15.0	18.9
4. 交通运输和邮电通讯业	Transportation, Postal and Telecommunications Services	109.0	144.1
5. 商业、饮食、物资供销和仓储业	Commerce, Catering Services, Materials Supply, Marketing and Storage	2.9	3.1
6. 其他	Other	7.1	6.0
7. 生活消费	Residential Consumption		
在消费量中：	Consumption by Usage		
(一) 终端消费	(I) Final Consumption	1617.9	1538.8
#工业	Industry	1481.6	1363.5
(二) 中间消费	(II) Intermediate Consumption		
(用于加工转换)	(Consumed in Transformation)	1455.8	1296.1
发 电	Power Generation	1419.1	1042.3
供 热	Heating		219.3
制 气	Gas Production	36.7	34.5
(三) 损失量	(III) Other Losses		2.5
**平衡差额**	**Balance**	**22.4**	**10.6**

(C)1994-2024 China Academic Journal Electronic Publishing House. All rights reserved. http://www.cnki.net

FUEL OIL BALANCE

(10 000 tons)

	1990	1995	1996	1997	1998	1999	2000	2001	2002
	**3320.7**	**3717.3**	**3632.3**	**3721.1**	**3828.7**	**3901.1**	**3836.7**	**3836.9**	**3702.8**
	3267.9	2960.8	2504.5	2311.2	2100.4	1959.4	2053.7	1864.4	1845.5
	167.3	859.1	1192.6	1506.8	1818.3	1963.3	1704.3	2048.7	1915.2
	97.2	68.6	70.8	91.2	72.9	38.8	57.9	84.9	104.9
	-17.3	-34.0	6.0	-5.7	-17.2	17.2	136.6	8.7	47.0
	**3367.8**	**3693.7**	**3564.5**	**3821.3**	**3828.6**	**3934.1**	**3872.8**	**3850.2**	**3873.9**
	2.9	8.4	2.5	2.9	0.3	0.4	0.4	0.4	0.4
	3091.7	3406.2	3305.5	3223.0	3217.3	3047.7	2975.1	2949.3	2950.9
	47.3	14.2	14.0	19.2	16.6	16.2	16.7	16.2	19.0
	208.2	227.5	224.5	582.2	565.6	840.0	850.0	855.0	872.1
	1.6	6.6	4.3	6.2	7.4	10.5	11.6	12.3	12.3
	16.1	30.8	14.5	14.7	21.4	19.4	19.0	17.0	19.1
	2042.6	2262.8	2320.6	2651.1	2456.4	2694.0	2741.4	2689.3	2675.6
	1766.5	1975.3	1961.8	2025.8	1845.1	1807.6	1843.7	1788.4	1752.6
	1325.2	1430.9	1243.9	1170.3	1372.2	1240.1	1131.3	1160.9	1198.2
	977.3	1071.5	888.8	839.2	993.8	906.3	814.2	838.5	912.7
	308.3	307.8	297.1	282.0	343.0	301.3	291.2	299.6	266.7
	39.6	51.6	58.1	49.2	35.4	32.5	25.9	22.8	18.9
	**-47.1**	**23.6**	**67.8**	**-100.2**	**0.1**	**-33.0**	**-36.1**	**-13.4**	**-171.1**

(C)1994-2024 China Academic Journal Electronic Publishing House. All rights reserved. http://www.cnki.net

4－10 汽油平衡表

单位：万吨

项 目	Item	1980	1985
**可供量**	**Total Energy Available for Consumption**	**999.4**	**1399.6**
生产量	Output	1079.0	1471.9
进口量	Imports		0.3
出口量(－)	Exports (－)	117.8	129.9
年初年末库存差额	Stock Changes in the Year	38.2	57.3
**消费量**	**Total Energy Consumption**	**998.6**	**1396.3**
在消费量中：	Consumption by Sector		
1. 农、林、牧、渔、水利业	Farming, Forestry, Animal Husbandry, Fishery and Water Conservancy	53.3	122.3
2. 工 业	Industry	273.2	451.3
3. 建筑业	Construction	54.1	73.0
4. 交通运输和邮电通讯业	Transportation, Postal and Telecommunications Services	404.9	477.4
5. 商业、饮食、物资供销和仓储业	Commerce, Catering Services, Materi－als Supply, Marketing and Storage	19.4	23.4
6. 其他	Other	193.7	238.3
7. 生活消费	Residential Consumption		10.6
**平衡差额**	**Balance**	**0.8**	**3.3**

4－11 煤油平衡表

单位：万吨

项 目	Item	1980	1985
**可供量**	**Total Energy Available for Consumption**	**359.0**	**383.2**
生产量	Output	398.5	405.3
进口量	Imports		15.2
出口量(－)	Exports (－)	46.8	46.0
年初年末库存差额	Stock Changes in the Year	2.3	8.7
**消费量**	**Total Energy Consumption**	**365.9**	**385.5**
在消费量中：	Consumption by Sector		
1. 农、林、牧、渔、水利业	Farming, Forestry, Animal Husbandry, Fishery and Water Conservancy	2.3	3.3
2. 工 业	Industry	15.7	20.1
3. 建筑业	Construction	0.8	1.3
4. 交通运输和邮电通讯业	Transportation, Postal and Telecommunications Services	31.4	56.2
5. 商业、饮食、物资供销和仓储业	Commerce, Catering Services, Materi－als Supply, Marketing and Storage	0.2	0.1
6. 其他	Other	216.7	182.9
7. 生活消费	Residential Consumption	98.8	121.6
**平衡差额**	**Balance**	**－6.9**	**－2.3**

(C)1994-2024 China Academic Journal Electronic Publishing House. All rights reserved. http://www.cnki.net

GASOLINE BALANCE

(10 000 tons)

1990	1995	1996	1997	1998	1999	2000	2001	2002
**1884.1**	**2902.0**	**2172.3**	**3284.0**	**3307.6**	**3379.8**	**3504.5**	**3606.9**	**3749.7**
2173.4	3051.6	3274.6	3517.8	3501.0	3741.3	4134.7	4154.7	4320.8
16.9	15.9	7.9	8.4	1.5	0.0	0.0	0.0	0.0
233.8	193.1	139.5	185.9	194.5	425.8	467.7	586.0	630.4
-72.4	27.6	29.3	-56.4	-0.5	64.3	-162.5	38.1	59.3
**1899.5**	**2909.6**	**3182.4**	**3312.0**	**3328.6**	**3380.7**	**3504.9**	**3597.8**	**3749.7**
145.9	179.7	180.2	176.4	172.6	178.1	184.5	190.6	187.9
589.3	812.4	895.7	723.1	677.5	646.5	602.0	618.1	631.6
89.5	103.6	106.2	107.7	112.6	113.8	115.6	116.7	122.3
620.1	982.3	991.3	1183.2	1216.6	1265.5	1387.8	1419.4	1503.5
46.0	197.2	195.8	211.7	216.5	206.3	209.8	214.0	224.2
390.7	570.7	726.8	815.8	825.7	849.4	877.7	904.3	916.3
18.0	63.7	86.4	94.2	107.1	121.1	127.6	134.6	163.8
**-15.4**	**-7.6**	**-10.1**	**-28.0**	**-21.1**	**-0.9**	**-0.4**	**9.1**	**0.0**

KEROSENE BALANCE

(10 000 tons)

1990	1995	1996	1997	1998	1999	2000	2001	2002
**350.9**	**486.4**	**542.1**	**681.7**	**699.9**	**848.6**	**880.9**	**894.2**	**914.8**
392.5	445.8	538.4	577.0	616.1	743.8	872.3	789.4	826.1
26.1	115.7	101.1	218.1	188.0	272.2	322.5	298.6	324.3
55.5	62.4	102.4	98.8	127.6	162.8	256.3	246.4	240.9
-12.2	-12.7	5.0	-14.6	23.3	-4.6	-57.6	52.7	5.3
**350.9**	**512.1**	**555.5**	**681.7**	**671.4**	**824.2**	**870.1**	**890.3**	**919.2**
3.1	3.6	1.7	1.4	1.6	1.4	1.5	1.5	1.4
20.6	44.9	43.1	46.7	62.2	78.4	83.9	86.0	87.4
1.3	3.5	5.0	4.2	3.5	3.9	4.0	3.5	0.0
93.4	250.0	298.9	420.1	390.5	505.6	536.4	560.7	616.7
0.6	8.5	8.3	9.0	9.0	11.5	12.0	12.5	13.0
127.3	137.3	133.9	137.7	141.6	152.7	160.1	151.1	140.0
104.6	64.3	64.7	62.7	63.1	70.8	72.2	75.0	60.7
	**-25.7**	**-13.4**		**28.5**	**24.3**	**11.3**	**4.0**	**-4.4**

(C)1994-2024 China Academic Journal Electronic Publishing House. All rights reserved. http://www.cnki.net

4－12 柴油平衡表

单位：万吨

项目	Item	1980	1985
**可供量**	**Total Energy Available for Consumption**	**1663.2**	**1944.1**
生产量	Output	1827.8	2023.2
进口量	Imports	2.1	4.5
出口量(－)	Exports (－)	166.5	225.6
年初年末库存差额	Stock Changes in the Year	－0.2	142.0
**消费量**	**Total Energy Consumption**	**1663.2**	**1939.4**
在消费量中：	Consumption by Sector		
1. 农、林、牧、渔、水利业	Farming, Forestry, Animal Husbandry, Fishery and Water Conservancy	749.0	629.2
2. 工业	Industry	457.4	644.1
3. 建筑业	Construction	76.5	125.0
4. 交通运输和邮电通讯业	Transportation, Postal and Telecommunications Services	316.1	454.4
5. 商业、饮食、物资供销和仓储业	Commerce, Catering Services, Materials Supply, Marketing and Storage	6.5	10.9
6. 其他	Other	57.7	74.0
7. 生活消费	Residential Consumption		
在消费量中：	Consumption by Usage		
(一) 终端消费	(I) Final Consumption	1590.9	1827.4
#工业	Industry	385.1	532.1
(二) 中间消费	(II) Intermediate Consumption		
(用于加工转换)	(Consumed in Transformation)	72.3	108.6
发电	Power Generation	72.3	103.6
供热	Heating		5.0
(三) 损失量	Other Losses		3.4
**平衡差额**	**Balance**		**4.7**

(C)1994-2024 China Academic Journal Electronic Publishing House. All rights reserved. http://www.cnki.net

DIESEL OIL BALANCE

(10 000 tons)

1990	1995	1996	1997	1998	1999	2000	2001	2002
**2689.4**	**4404.2**	**4818.7**	**5271.2**	**5229.8**	**6204.3**	**6806.5**	**7272.0**	**7722.3**
2609.0	3972.6	4419.0	4924.5	4897.7	6172.6	7079.6	7485.6	7706.1
233.8	645.3	512.1	790.2	331.7	56.0	51.9	54.7	78.7
169.8	169.5	192.4	261.5	118.8	70.9	77.5	46.9	144.7
16.4	-44.2	80.0	-182.0	119.2	46.5	-247.6	-221.4	82.2
**2691.7**	**4321.4**	**4691.7**	**5291.2**	**5282.8**	**6231.6**	**6774.3**	**7107.7**	**7667.2**
881.5	1001.4	1028.4	1075.7	1120.2	1241.8	1310.1	1375.6	1484.3
728.1	1189.9	1357.2	1730.4	1346.1	1506.8	1596.5	1637.6	1732.1
133.0	118.2	129.6	146.0	153.5	178.1	195.9	223.1	252.0
709.4	1246.6	1261.1	1379.5	1901.9	2221.7	2543.8	2671.0	2964.8
22.5	103.6	125.3	146.4	153.3	260.9	255.9	268.1	280.8
217.0	645.7	768.1	774.0	564.0	759.7	803.7	853.9	870.0
	16.1	22.1	39.2	43.9	62.7	68.4	79.2	83.9
2564.8	4070.0	4408.5	4549.2	5078.9	6016.2	6546.6	6867.3	7441.2
601.2	938.5	1074.1	988.4	1142.2	1291.4	1368.8	1396.4	1505.3
126.9	251.4	283.1	742.0	203.9	215.5	227.7	240.4	226.0
124.5	204.9	253.3	739.1	203.9	215.5	227.7	240.4	226.0
2.4	46.6	29.9	2.9					
**-2.3**	**82.7**	**127.0**	**-20.0**	**-52.9**	**-27.4**	**32.2**	**164.4**	**55.2**

(C)1994-2024 China Academic Journal Electronic Publishing House. All rights reserved. http://www.cnki.net

4－13 液化石油气平衡表

单位：万吨

项　目	Item	1980	1985
**可供量**	**Total Energy Available for Consumption**	**122.5**	**157.3**
生产量	Output	122.5	159.7
进口量	Imports		
出口量(－)	Exports (－)		1.9
年初年末库存差额	Stock Changes in the Year		－0.5
**消费量**	**Total Energy Consumption**	**119.6**	**155.7**
在消费量中：	Consumption by Sector		
1. 农、林、牧、渔、水利业	Farming, Forestry, Animal Husbandry, Fishery and Water Conservancy		
2. 工　业	Industry	76.1	59.9
3. 建筑业	Construction		
4. 交通运输和邮电通讯业	Transportation, Postal and Telecommunications Services		
5. 商业、饮食、物资供销和仓储业	Commerce, Catering Services, Materials Supply, Marketing and Storage		0.5
6. 其他	Other	0.4	4.5
7. 生活消费	Residential Consumption	43.1	90.8
**平衡差额**	**Balance**	**2.9**	**1.6**

4－14 天然气平衡表

单位：万吨

项　目	Item	1980	1985
**可供量**	**Total Energy Available for Consumption**	**142.7**	**129.3**
生产量	Output	142.7	129.3
进口量	Imports		
出口量(－)	Exports (－)		
年初年末库存差额	Stock Changes in the Year		
**消费量**	**Total Energy Consumption**	**140.6**	**129.3**
在消费量中：	Consumption by Sector		
1. 农、林、牧、渔、水利业	Farming, Forestry, Animal Husbandry, Fishery and Water Conservancy		
2. 工　业	Industry	131.4	109.6
3. 建筑业	Construction	6.0	14.1
4. 交通运输和邮电通讯业	Transportation, Postal and Telecommunications Services	0.7	0.8
5. 商业、饮食、物资供销和仓储业	Commerce, Catering Services, Materials Supply, Marketing and Storage		
6. 其他	Other	0.5	0.5
7. 生活消费	Residential Consumption	2.0	4.3
**平衡差额**	**Balance**	**2.1**	

(C)1994-2024 China Academic Journal Electronic Publishing House. All rights reserved. http://www.cnki.net

LPG BALANCE

(10 000 tons)

1990	1995	1996	1997	1998	1999	2000	2001	2002
**258.5**	**774.3**	**923.1**	**992.1**	**1178.0**	**1146.6**	**1396.16**	**1428.03**	**1663.61**
261.6	540.8	605.9	667.9	747.4	816.6	916.62	952.33	1036.79
	232.6	355.0	358.2	476.6	322.3	481.74	488.86	626.16
1.1	7.1	33.3	39.2	50.2	7.5	1.6	2.09	5.62
-2.0	8.0	-4.5	5.1	4.2	-1.5	-0.6	-11.07	6.28
**254.2**	**750.6**	**931.3**	**1009.7**	**1186.0**	**1208.5**	**1366.67**	**1411.0**	**1618.39**
	0.1						0.3	
82.0	192.5	183.0	203.4	220.5	254.0	276.14	291.67	320.49
1.0	0.5	0.4	5.2	5.7	7.9	8.91	9.51	12.76
	0.5	1.6	1.6	0.5	2.0	16.45	17.03	21.58
6.6	17.4	33.9	42.9	39.5	47.9	55.48	60.4	62.59
6.1	5.7	8.8	14.5	150.6	17.9	21	26.21	31.92
158.5	534.0	703.5	742.2	769.2	878.5	988.34	1006.04	1169.05
**4.3**	**23.7**	**-8.2**	**-17.7**	**-8.0**	**-61.6**	**29.49**	**16.87**	**45.22**

NATURAL GAS BALANCE

(10^8 cu.m)

1990	1995	1996	1997	1998	1999	2000	2001	2002
**153.0**	**179.5**	**201.1**	**226.7**	**232.8**	**252.0**	**272.0**	**303.3**	**326.6**
153.0	179.5	201.1	227.0	232.8	252.0	272.0	303.3	326.6
			0.3					
**152.5**	**177.4**	**184.9**	**195.4**	**202.6**	**214.9**	**245.0**	**274.3**	**291.8**
		0.2						
120.2	154.4	157.2	168.9	171.5	180.2	202.0	217.8	227.5
10.6	0.3	1.5		0.1	0.7	0.8	0.7	0.7
1.9	1.6	4.0	3.7	3.7	4.8	5.8	6.0	6.4
	0.6	1.0	1.0	2.5	2.9	3.4	5.0	6.1
1.2	1.2	1.3	0.6	0.6	0.6	0.6	0.7	
18.6	19.4	19.7	21.2	24.1	25.7	32.3	44.1	51.2
**0.5**	**2.1**	**16.2**	**31.3**	**30.2**	**37.0**	**27.0**	**29.0**	**34.8**

(C)1994-2024 China Academic Journal Electronic Publishing House. All rights reserved. http://www.cnki.net

4－15 电力平衡表

单位：亿千瓦小时

项　目	Item	1980	1985
**可供量**	**Total Energy Available for Consumption**	**3006.3**	**4117.6**
生产量	Output	3006.3	4106.9
水　电	Hydropower	582.1	923.7
火　电	Thermal Power	2424.2	3183.2
核　电	Nuclear Power		
进口量	Imports		11.1
出口量(－)	Exports (－)		0.4
**消费量**	**Total Energy Consumption**	**3006.3**	**4117.6**
在消费量中：	Consumption by Sector		
1. 农、林、牧、渔、水利业	Farming, Forestry, Animal Husbandry, Fishery and Water Conservancy	270	317.4
2. 工　业	Industry	2471:9	3283.4
3. 建筑业	Construction	47.1	71.2
4. 交通运输和邮电通讯业	Transportation, Postal and Telecommunications Services	26.5	63.4
5. 商业、饮食、物资供销和仓储业	Commerce, Catering Services, Materi－als Supply, Marketing and Storage	16.8	38.0
6. 其他	Others	68.8	121.7
7. 生活消费	Residential Consumption	105.2	222.5
在消费量中：	Consumption by Usage		
(一) 终端消费	(I)Final Consumption	2763.4	3813.3
＃工业	Industry	2229.0	2979.1
(二) 输配电损失量	(II)Losses in Transmission	242.9	304.3

(C)1994-2024 China Academic Journal Electronic Publishing House. All rights reserved.　http://www.cnki.net

ELECTRICITY BALANCE

(100 million kwh)

1990	1995	1996	1997	1998	1999	2000	2001	2002
**6230.4**	**10023.4**	**10764.3**	**11273.6**	**11590.4**	**12305.2**	**13472.7**	**14632.6**	**16330.7**
6212.0	10077.3	10800.2	11344.7	11662.0	12393.0	13556.0	14716.6	16404.8
1267.2	1905.8	1879.7	1959.8	2080.0	2038.1	2224.1	2774.3	2879.7
4944.8	8043.2	8777.1	9240.7	9441.0	10205.4	11164.5	11767.5	13273.8
	128.3	143.4	144.2	141.0	149.5	167.4	174.7	251.3
19.3	6.4	1.2	0.9	0.2	3.7	15.5	18.0	23.0
0.9	60.3	37.1	72.0	71.7	91.5	98.8	101.9	97.0
**6230.4**	**10023.4**	**10764.3**	**11284.4**	**11598.4**	**12305.2**	**13471.4**	**14633.5**	**16331.5**
426.8	582.4	618.3	639.8	623.5	660.4	673.0	762.4	776.2
4873.3	7659.8	8044.7	8395.7	8406.0	8832.7	9653.6	10444.7	11793.2
65.0	159.6	181.8	117.4	188.8	142.3	154.8	144.9	164.1
105.9	182.3	197.9	255.9	255.6	254.8	281.2	309.3	338.0
76.2	199.5	223.1	265.1	293.4	342.8	393.7	444.9	500.0
202.4	234.2	365.4	357.4	506.7	591.4	643.2	688.1	758.5
480.8	1005.6	1133.1	1253.2	1324.5	1480.8	1672.0	1839.2	2001.4
5795.8	9278.9	9994.8	10486.0	10807.5	11443.3	12534.7	13600.0	15162.8
4438.7	6915.3	7275.2	7597.3	7615.0	7970.8	8716.9	9411.2	10624.5
434.6	744.5	769.5	798.4	790.9	861.9	936.7	1033.5	1168.7

(C)1994-2024 China Academic Journal Electronic Publishing House. All rights reserved. http://www.cnki.net
