# Supplementary material for: Prediction analysis of carbon emission in China’s electricity industry based on the dual carbon background
Source: PLoS One. 2024 May 17;19(5):e0302068. doi: 10.1371/journal.pone.0302068 (PMC11101092; doi:10.1371/journal.pone.0302068)
Supplement: S4 File — (ZIP) [file pone.0302068.s004.zip › China Energy Statistic Yearbook 2001-2021/中国能源统计年鉴_胡汉舟_总编_五、全国能源平衡表-2021.pdf]

5-1 全国能源平衡表(实物量) -2020

项　目	Item	煤合计 (万吨) Coal Total (10^4 tons)	原煤 (万吨) Raw Coal (10^4 tons)
**一.可供本地区消费的能源量**	**Total Primary Energy Supply**	**414518.97**	**415805.42**
1.一次能源生产量	Indigenous Production	390157.71	390157.71
水电	Hydro Power		
核电	Nuclear Power		
风电	Wind Power		
2.进口量	Import	30361.09	30361.09
3.境内飞机和轮船在境外的加油量	Domestic Airplanes&Ships Refueling Abroad		
4.出口量(-)	Export (-)	319.46	316.78
5.境外飞机和轮船在境内的加油量(-)	Oversea Airplanes&Ships Refueling Domestically (-)		
6.库存增(-)、减(+)量	Stock Change	-5680.37	-4396.61
**二.加工转换投入(-)产出(+)量**	**Input(-) & Output(+) of Transformation**	**-332434.09**	**-349916.36**
1.火力发电	Thermal Power	-211634.87	-206845.78
2.供热	Heating Supply	-36933.28	-35434.18
3.煤炭洗选	Coal Washing	-11301.20	-90163.69
4.炼焦	Coking	-65967.64	-10960.01
5.炼油及煤制油	Petroleum Refining and Coal-to-liquids	-3047.29	-2914.28
#油品再投入量(-)	Petroleum Products Input (-)		
6.制气	Gas Works	-3308.95	-3150.59
#再投入量(-)	Input (-)		
7.天然气液化	Natural Gas Liquefaction		
8.煤制品加工	Briquettes	-240.86	-447.82
9.回收能	Recovery of Energy		
**三.损失量**	**Loss**		
**四.终端消费量**	**Total Final Consumption**	**72425.57**	**57206.52**
1.农、林、牧、渔业	Agriculture, Forestry, Animal Husbandry and Fishery	2253.90	2172.28
2.工业	Industry	58457.16	44258.69
#用作原料、材料	Non-Energy Use	13945.34	11477.50
3.建筑业	Construction	638.85	597.02
4.交通运输、仓储和邮政业	Transport, Storage and Post	241.01	224.31
5.批发和零售业、住宿和餐饮业	Wholesale and Retail Trades, Hotels and Catering Services	1981.18	1926.62
6.其他	Others	2570.80	2488.06
7.居民生活	Residential	6282.66	5539.54
城镇	Urban	611.84	482.44
乡村	Rural	5670.81	5057.10
**五.平衡差额**	**Statistical Difference**	**9659.31**	**8682.54**
**六.消费量合计**	**Total Energy Consumption**	**404859.66**	**407122.88**

(C)1994-2024 China Academic Journal Electronic Publishing House. All rights reserved. http://www.cnki.net

Energy Balance of China (Physical Quantity) -2020

洗精煤 (万吨) Cleaned Coal (10^4 tons)	其他洗煤 (万吨) Other Washed Coal (10^4 tons)	煤制品 (万吨) Briquettes (10^4 tons)	煤矸石 (万吨) Gangue (10^4 tons)	焦炭 (万吨) Coke (10^4 tons)	焦炉煤气 (亿立方米) Coke Oven Gas (10^8 cu.m)	高炉煤气 (亿立方米) Blast Furnace Gas (10^8 cu.m)	转炉煤气 (亿立方米) Converter Gas (10^8 cu.m)	其他煤气 (亿立方米) Other Gas (10^8 cu.m)
**-747.63**	**-531.00**	**-7.81**		**123.79**				
				297.91				
		2.68		349.21				
-747.63	-531.00	-5.13		175.09				
**1117.80**	**15008.33**	**1356.13**	**-60.08**	**46640.33**	**711.53**	**8481.93**	**661.85**	**145.40**
	-4789.09		-2087.61	-250.03	-217.85	-2932.52	-360.91	-3.48
	-1499.10		-762.82	-280.28	-57.17	-500.05	-49.18	-1.60
56125.43	22737.06		2790.35					
-55007.63				47188.23	1032.53			
	-133.01							
	-158.35							150.47
				-17.59	-45.99			
	-1149.18	1356.13						
						11914.49	1071.94	
	**13932.63**	**1286.41**		**47762.51**	**727.21**	**8638.81**	**651.37**	**149.80**
	81.62			23.24				
	13300.72	897.76		47724.27	718.58	8638.81	651.37	97.63
	2302.54	165.30		1818.29	105.48			
	41.83			3.57				
	16.70							
	37.37	17.18		0.02	0.68			9.54
	76.57	6.17			0.88			
	377.83	365.29		11.41	7.07			42.63
	52.23	77.18		2.82	5.12			42.41
	325.60	288.11		8.59	1.95			0.22
**370.17**	**544.69**	**61.91**	**-60.08**	**-998.40**	**-15.67**	**-156.88**	**10.48**	**-4.40**
**55007.63**	**21661.36**	**1286.41**	**2850.43**	**48310.41**	**1048.21**	**12071.38**	**1061.46**	**154.88**

(C)1994-2024 China Academic Journal Electronic Publishing House. All rights reserved. http://www.cnki.net

5-1 续表 1

项　目	Item	其他焦化产品 (万吨) Other Coking Products (10^4 tons)	油品合计 (万吨) Petroleum Products Total (10^4 tons)
**一.可供本地区消费的能源量**	**Total Primary Energy Supply**		**67553.72**
1.一次能源生产量	Indigenous Production		19476.86
水电	Hydro Power		
核电	Nuclear Power		
风电	Wind Power		
2.进口量	Import		60497.54
3.境内飞机和轮船在境外的加油量	Domestic Airplanes&Ships Refueling Abroad		774.14
4.出口量(-)	Export (-)		6738.13
5.境外飞机和轮船在境内的加油量(-)	Oversea Airplanes&Ships Refueling Domestically (-)		812.90
6.库存增(-)、减(+)量	Stock Change		-5643.79
**二.加工转换投入(-)产出(+)量**	**Input(-) & Output(+) of Transformation**	**933.70**	**-3265.64**
1.火力发电	Thermal Power		-321.54
2.供热	Heating Supply		-677.70
3.煤炭洗选	Coal Washing		
4.炼焦	Coking	1350.21	-4.60
5.炼油及煤制油	Petroleum Refining and Coal-to-liquids	-412.06	12495.07
#油品再投入量(-)	Petroleum Products Input (-)		-14728.00
6.制气	Gas Works	2.17	-28.86
#再投入量(-)	Input (-)	-6.63	
7.天然气液化	Natural Gas Liquefaction		
8.煤制品加工	Briquettes		
9.回收能	Recovery of Energy		
**三.损失量**	**Loss**		**18.26**
**四.终端消费量**	**Total Final Consumption**	**1001.96**	**62085.22**
1.农、林、牧、渔业	Agriculture, Forestry, Animal Husbandry and Fishery		1773.10
2.工业	Industry	1001.96	24428.39
#用作原料、材料	Non-Energy Use	369.98	14394.21
3.建筑业	Construction		4180.29
4.交通运输、仓储和邮政业	Transport, Storage and Post		20480.16
5.批发和零售业、住宿和餐饮业	Wholesale and Retail Trades, Hotels and Catering Services		583.27
6.其他	Others		3469.89
7.居民生活	Residential		7170.11
城镇	Urban		4884.76
乡村	Rural		2285.35
**五.平衡差额**	**Statistical Difference**	**-68.26**	**2184.60**
**六.消费量合计**	**Total Energy Consumption**	**1420.65**	**65369.11**

(C)1994-2024 China Academic Journal Electronic Publishing House. All rights reserved. http://www.cnki.net

Continued 1

原油 (万吨) Crude Oil (10^4 tons)	汽油 (万吨) Gasoline (10^4 tons)	煤油 (万吨) Kerosene (10^4 tons)	柴油 (万吨) Diesel Oil (10^4 tons)	燃料油 (万吨) Fuel Oil (10^4 tons)	石脑油 (万吨) Naphtha (10^4 tons)	润滑油 (万吨) Lubricants (10^4 tons)	石蜡 (万吨) Paraffin Waxes (10^4 tons)	溶剂油 (万吨) White Spirit (10^4 tons)
**69969.65**	**-1427.66**	**-717.04**	**-2070.17**	**-436.41**	**772.46**	**16.30**	**-49.71**	**5.10**
19476.86								
54200.67	48.05	265.73	119.10	1253.47	788.70	28.95	11.34	3.69
		248.32	18.37	507.45				
163.81	1600.00	997.48	1975.95	1583.31		13.43	66.00	0.31
		228.78	35.21	548.91				
-3544.07	124.29	-4.82	-196.48	-65.10	-16.24	0.79	4.94	1.72
**-69040.48**	**14233.10**	**4129.42**	**16383.32**	**2749.06**	**5621.91**	**258.93**	**239.24**	**37.64**
-16.55			-27.58	-29.43				
			-2.91	-231.38				
-69023.93	14256.69	4129.42	16529.85	5780.70	7190.72	263.38	249.42	39.97
	-23.59		-116.04	-2770.83	-1568.81	-4.45	-10.18	-2.32
**17.63**								
**419.03**	**12743.57**	**3352.10**	**14136.16**	**2332.96**	**6178.69**	**267.78**	**198.21**	**44.16**
	257.35	11.00	1497.15	1.13				
419.03	160.38	9.39	879.58	230.69	6178.69	267.78	198.21	44.16
	2.47	1.82	20.75	37.99	5750.38	245.96	194.33	41.56
	508.39	10.81	503.92	40.75				
	5573.57	3110.76	9531.98	2042.01				
	273.23	14.76	197.61	11.80				
	2253.09	182.96	946.15	6.59				
	3717.56	12.42	579.78					
	2510.98	0.36	265.29					
	1206.59	12.06	314.49					
**492.50**	**61.87**	**60.28**	**176.98**	**-20.31**	**215.68**	**7.46**	**-8.68**	**-1.41**
**69477.14**	**12767.16**	**3352.10**	**14282.70**	**5364.60**	**7747.49**	**272.23**	**208.39**	**46.48**

(C)1994-2024 China Academic Journal Electronic Publishing House. All rights reserved. http://www.cnki.net

5-1 续表 2

项　目	Item	石油沥青(万吨) Bitumen Asphalt (10^4 tons)	石油焦(万吨) Petroleum Coke (10^4 tons)
**一.可供本地区消费的能源量**	**Total Primary Energy Supply**	**228.14**	**847.55**
1.一次能源生产量	Indigenous Production		
水电	Hydro Power		
核电	Nuclear Power		
风电	Wind Power		
2.进口量	Import	475.92	1027.66
3.境内飞机和轮船在境外的加油量	Domestic Airplanes&Ships Refueling Abroad		
4.出口量(−)	Export (-)	56.74	178.36
5.境外飞机和轮船在境内的加油量(−)	Oversea Airplanes&Ships Refueling Domestically (-)		
6.库存增(−)、减(+)量	Stock Change	-191.04	-1.76
**二.加工转换投入(−)产出(+)量**	**Input(-) & Output(+) of Transformation**	**3321.53**	**2454.29**
1.火力发电	Thermal Power		-189.88
2.供热	Heating Supply		-256.55
3.煤炭洗选	Coal Washing		
4.炼焦	Coking		-4.60
5.炼油及煤制油	Petroleum Refining and Coal-to-liquids	3950.47	2956.90
#油品再投入量(−)	Petroleum Products Input (-)	-628.94	-42.34
6.制气	Gas Works		-9.25
#再投入量(−)	Input (-)		
7.天然气液化	Natural Gas Liquefaction		
8.煤制品加工	Briquettes		
9.回收能	Recovery of Energy		
**三.损失量**	**Loss**		
**四.终端消费量**	**Total Final Consumption**	**3479.59**	**3163.41**
1.农、林、牧、渔业	Agriculture, Forestry, Animal Husbandry and Fishery		
2.工业	Industry	349.52	3163.41
#用作原料、材料	Non-Energy Use	304.14	2423.03
3.建筑业	Construction	3018.82	
4.交通运输、仓储和邮政业	Transport, Storage and Post	111.24	
5.批发和零售业、住宿和餐饮业	Wholesale and Retail Trades, Hotels and Catering Services		
6.其他	Others		
7.居民生活	Residential		
城镇	Urban		
乡村	Rural		
**五.平衡差额**	**Statistical Difference**	**70.08**	**138.42**
**六.消费量合计**	**Total Energy Consumption**	**4108.53**	**3666.03**

(C)1994-2024 China Academic Journal Electronic Publishing House. All rights reserved. http://www.cnki.net

Continued 2

液化石油气 (万吨) Liquefied Petroleum Gas (10^4 tons)	炼厂干气 (万吨) Refinery Gas (10^4 tons)	其他石油制品 (万吨) Other Petroleum Products (10^4 tons)	天然气 (亿立方米) Natural Gas (10^8 cu.m)	液化天然气 (万吨) Liquefied Natural Gas (10^4 tons)	热力 (万百万千焦) Heat (10^{10} kJ)	电力 (亿千瓦时) Electricity (10^8 kW•h)	其他能源 (万吨标准煤) Other Energy (10^4 tce)
**1892.07**		**-1476.56**	**2350.63**	**6663.86**		**24317.69**	**7214.43**
			1924.95			24488.12	7214.43
						13552.09	
						3662.55	
						4664.74	
2004.63		269.65	476.53	6669.73		47.51	
94.89		7.86	50.85	5.87		217.93	
-17.68		-1738.35					
**3920.55**	**1896.13**	**10529.72**	**-755.92**	**931.47**	**596841.75**	**53302.48**	**-1325.91**
-0.14	-50.19	-7.78	-411.37	-352.62	-127833.91	53302.48	-2017.84
-7.71	-165.20	-13.95	-169.66	-48.78	613906.59		-689.73
4489.00	2348.61	19333.88	-31.42				-415.24
-556.88	-221.19	-8782.42					
-3.72	-15.90		69.94				768.20
			-15.17				
			-198.25	1332.86			
					110769.08		1028.70
**0.63**			**26.13**	**1.47**	**5204.80**	**3233.48**	
**5652.07**	**1863.57**	**8253.91**	**1568.80**	**7590.07**	**591129.64**	**74386.69**	**5590.70**
6.47			1.28		111.89	1422.11	666.23
2496.33	1863.57	8167.65	614.97	7042.49	412216.71	49119.96	990.53
866.76	127.15	4377.86	87.44	162.98			105.78
11.34		86.26	2.64		1933.11	1011.10	28.55
110.61			272.25	547.58	3904.98	1750.98	1208.26
85.87			62.14		10405.72	3169.04	64.00
81.10			55.55		21207.86	6517.02	245.50
2860.35			559.97		141349.38	11396.48	2387.64
2108.13			553.67		141349.38	6157.21	238.00
752.22			6.30			5239.27	2149.64
**159.91**	**32.56**	**799.25**	**-0.22**	**3.79**	**507.31**		**297.82**
**6221.15**	**2316.04**	**17058.07**	**2236.86**	**7992.93**	**724168.35**	**77620.17**	**8713.51**

(C)1994-2024 China Academic Journal Electronic Publishing House. All rights reserved. http://www.cnki.net

5-2 全国能源平衡表(标准量) -2020

单位：万吨标准煤

项　目	Item	能源合计 (发电煤耗计算法) (Coal Equivalent Calculation)	Energy Total (电热当量计算法) (Calorific Value Calculation)
**一.可供本地区消费的能源量**	**Total Primary Energy Supply**	**507478.68**	**464901.27**
1.一次能源生产量	Indigenous Production	407295.20	364419.41
水电	Hydro Power	40383.62	16655.52
核电	Nuclear Power	10913.96	4501.27
风电	Wind Power	13900.37	5732.96
2.进口量	Import	123687.97	123604.79
3.境内飞机和轮船在境外的加油量	Domestic Airplanes&Ships Refueling Abroad	1117.09	1117.09
4.出口量(-)	Export (-)	11665.68	11284.11
5.境外飞机和轮船在境内的加油量(-)	Oversea Airplanes&Ships Refueling Domestically (-)	1172.11	1172.11
6.库存增(-)、减(+)量	Stock Change	-11783.80	-11783.80
**二.加工转换投入(-)产出(+)量**	**Input(-) & Output(+) of Transformation**	**16.87**	**-93309.47**
1.火力发电	Thermal Power		-93326.33
2.供热	Heating Supply	-7202.87	-7202.87
3.煤炭洗选	Coal Washing	-4548.04	-4548.04
4.炼焦	Coking	-3937.65	-3937.65
5.炼油及煤制油	Petroleum Refining and Coal-to-liquids	14258.47	14258.47
#油品再投入量(-)	Petroleum Products Input (-)	-20390.25	-20390.25
6.制气	Gas Works	-425.68	-425.68
#再投入量(-)	Input (-)	-481.10	-481.10
7.天然气液化	Natural Gas Liquefaction	-182.70	-182.70
8.煤制品加工	Briquettes	-110.54	-110.54
9.回收能	Recovery of Energy	23037.21	23037.21
**三.损失量**	**Loss**	**10175.19**	**4513.74**
**四.终端消费量**	**Total Final Consumption**	**488155.77**	**357913.48**
1.农、林、牧、渔业	Agriculture, Forestry, Animal Husbandry and Fishery	9262.85	6772.91
2.工业	Industry	322677.35	236674.13
#用作原料、材料	Non-Energy Use	34557.13	34557.13
3.建筑业	Construction	9320.36	7550.03
4.交通运输、仓储和邮政业	Transport, Storage and Post	41098.69	38032.92
5.批发和零售业、住宿和餐饮业	Wholesale and Retail Trades, Hotels and Catering Services	13171.11	7622.50
6.其他	Others	28245.06	16834.53
7.居民生活	Residential	64380.34	44426.45
城镇	Urban	38731.64	27951.09
乡村	Rural	25648.71	16475.36
**五.平衡差额**	**Statistical Difference**	**9164.59**	**9164.59**
**六.消费量合计**	**Total Energy Consumption**	**498314.09**	**455736.68**

(C)1994-2024 China Academic Journal Electronic Publishing House. All rights reserved. http://www.cnki.net

Energy Balance of China (Standard Quantity) -2020

(10^4 tce)

煤合计 Coal Total	原煤 Raw Coal	洗精煤 Cleaned Coal	其他洗煤 Other Washed Coal	煤制品 Briquettes	煤矸石 Gangue	焦炭 Coke	焦炉煤气 Coke Oven Gas	高炉煤气 Blast Furnace Gas	转炉煤气 Converter Gas
**289194.95**	**290159.30**	**-672.87**	**-286.74**	**-4.74**		**120.25**			
274719.77	274719.77								
18830.69	18830.69					289.39			
276.58	274.95			1.63		339.22			
-4078.93	-3116.21	-672.87	-286.74	-3.11		170.08			
**-229296.52**	**-239230.49**	**1006.02**	**8104.50**	**823.44**	**43.79**	**45306.41**	**4065.70**	**10907.76**	**1796.27**
-139530.78	-136944.67		-2586.11		-417.52	-242.87	-1244.77	-3771.22	-979.52
-22770.06	-21960.55		-809.52		-152.56	-272.27	-326.65	-643.06	-133.46
-5161.92	-67952.82	50512.89	12278.01		613.88				
-57229.50	-7722.64	-49506.87				45838.64	5899.90		
-2177.10	-2105.27		-71.82						
-2316.63	-2231.12		-85.51						
						-17.09	-262.78		
-110.54	-313.42		-620.56	823.44					
								15322.04	2909.25
**52835.06**	**44530.33**		**7523.62**	**781.11**		**46396.50**	**4155.26**	**11109.51**	**1767.82**
1727.13	1683.05		44.07			22.58			
42043.42	34315.91		7182.39	545.12		46359.35	4105.95	11109.51	1767.82
10411.24	9067.50		1243.37	100.37		1766.29	602.71		
530.50	507.91		22.59			3.47			
181.28	172.26		9.02						
1619.29	1588.67		20.18	10.43		0.02	3.88		
2031.00	1985.90		41.35	3.75			5.03		
4702.44	4276.61		204.03	221.80		11.09	40.40		
456.92	381.85		28.20	46.86		2.74	29.26		
4245.52	3894.76		175.82	174.94		8.34	11.14		
**7063.37**	**6398.49**	**333.16**	**294.13**	**37.59**	**43.79**	**-969.84**	**-89.56**	**-201.75**	**28.45**

(C)1994-2024 China Academic Journal Electronic Publishing House. All rights reserved. http://www.cnki.net

5-2 续表 1

单位：万吨标准煤

项　目	Item	其他煤气 Other Gas	其他焦化产品 Other Coking Products
**一.可供本地区消费的能源量**	**Total Primary Energy Supply**		
1.一次能源生产量	Indigenous Production		
水电	Hydro Power		
核电	Nuclear Power		
风电	Wind Power		
2.进口量	Import		
3.境内飞机和轮船在境外的加油量	Domestic Airplanes&Ships Refueling Abroad		
4.出口量(-)	Export (-)		
5.境外飞机和轮船在境内的加油量(-)	Oversea Airplanes&Ships Refueling Domestically (-)		
6.库存增(-)、减(+)量	Stock Change		
**二.加工转换投入(-)产出(+)量**	**Input(-) & Output(+) of Transformation**	**259.68**	**1077.48**
1.火力发电	Thermal Power	-6.22	
2.供热	Heating Supply	-2.85	
3.煤炭洗选	Coal Washing		
4.炼焦	Coking		1558.15
5.炼油及煤制油	Petroleum Refining and Coal-to-liquids		-475.52
#油品再投入量(-)	Petroleum Products Input (-)		
6.制气	Gas Works	268.75	2.51
#再投入量(-)	Input (-)		-7.65
7.天然气液化	Natural Gas Liquefaction		
8.煤制品加工	Briquettes		
9.回收能	Recovery of Energy		
**三.损失量**	**Loss**		
**四.终端消费量**	**Total Final Consumption**	**267.55**	**1156.26**
1.农、林、牧、渔业	Agriculture, Forestry, Animal Husbandry and Fishery		
2.工业	Industry	174.36	1156.26
#用作原料、材料	Non-Energy Use		426.95
3.建筑业	Construction		
4.交通运输、仓储和邮政业	Transport, Storage and Post		
5.批发和零售业、住宿和餐饮业	Wholesale and Retail Trades, Hotels and Catering Services	17.04	
6.其他	Others		
7.居民生活	Residential	76.14	
城镇	Urban	75.75	
乡村	Rural	0.39	
**五.平衡差额**	**Statistical Difference**	**-7.87**	**-78.77**
**六.消费量合计**	**Total Energy Consumption**		

(C)1994-2024 China Academic Journal Electronic Publishing House. All rights reserved. http://www.cnki.net

Continued 1

(10⁴ tce)

油品合计 Petroleum Products Total	原油 Crude Oil	汽油 Gasoline	煤油 Kerosene	柴油 Diesel Oil	燃料油 Fuel Oil	石脑油 Naphtha	润滑油 Lubricants	石蜡 Paraffin Waxes
**96752.95**	**99958.64**	**-2100.65**	**-1055.05**	**-3016.44**	**-623.46**	**1158.68**	**23.06**	**-67.85**
27824.64	27824.64							
86599.55	77431.08	70.69	390.99	173.53	1790.70	1183.04	40.94	15.48
1117.09			365.38	26.77	724.94			
9741.26	234.02	2354.23	1467.70	2879.16	2261.91		19.00	90.07
1172.11			336.63	51.30	784.18			
-7874.95	-5063.06	182.89	-7.09	-286.28	-93.01	-24.36	1.12	6.74
**-3999.05**	**-98631.23**	**20942.58**	**6076.02**	**23872.13**	**3927.31**	**8432.87**	**366.21**	**326.52**
-394.70	-23.64			-40.19	-42.04			
-895.53				-4.24	-330.55			
-4.83								
17727.32	-98607.59	20977.29	6076.02	24085.65	8258.31	10786.07	372.50	340.41
-20390.25		-34.71		-169.09	-3958.41	-2353.21	-6.30	-13.89
-41.07								
**26.27**	**25.19**							
**89669.83**	**598.63**	**18750.89**	**4932.28**	**20597.81**	**3332.87**	**9268.03**	**378.72**	**270.52**
2589.05		378.66	16.19	2181.50	1.62			
34291.99	598.63	235.98	13.82	1281.64	329.56	9268.03	378.72	270.52
19841.31		3.64	2.68	30.23	54.27	8625.57	347.87	265.22
5645.25		748.05	15.91	734.26	58.21			
29919.72		8200.94	4577.17	13889.04	2917.22			
875.74		402.04	21.72	287.94	16.85			
5111.49		3315.20	269.20	1378.64	9.41			
11236.59		5470.02	18.27	844.79				
7695.71		3694.65	0.53	386.55				
3540.88		1775.37	17.75	458.24				
**3057.80**	**703.59**	**91.03**	**88.70**	**257.88**	**-29.02**	**323.52**	**10.55**	**-11.85**

(C)1994-2024 China Academic Journal Electronic Publishing House. All rights reserved. http://www.cnki.net

5-2 续表 2

单位：万吨标准煤

项目	Item	溶剂油 White Spirit	石油沥青 Bitumen Asphalt
**一.可供本地区消费的能源量**	**Total Primary Energy Supply**	**7.49**	**298.87**
1.一次能源生产量	Indigenous Production		
水电	Hydro Power		
核电	Nuclear Power		
风电	Wind Power		
2.进口量	Import	5.41	623.46
3.境内飞机和轮船在境外的加油量	Domestic Airplanes&Ships Refueling Abroad		
4.出口量(-)	Export (-)	0.45	74.33
5.境外飞机和轮船在境内的加油量(-)	Oversea Airplanes&Ships Refueling Domestically (-)		
6.库存增(-)、减(+)量	Stock Change	2.52	-250.26
**二.加工转换投入(-)产出(+)量**	**Input(-) & Output(+) of Transformation**	**55.23**	**4351.20**
1.火力发电	Thermal Power		
2.供热	Heating Supply		
3.煤炭洗选	Coal Washing		
4.炼焦	Coking		
5.炼油及煤制油	Petroleum Refining and Coal-to-liquids	58.64	5175.12
#油品再投入量(-)	Petroleum Products Input (-)	-3.41	-823.91
6.制气	Gas Works		
#再投入量(-)	Input (-)		
7.天然气液化	Natural Gas Liquefaction		
8.煤制品加工	Briquettes		
9.回收能	Recovery of Energy		
**三.损失量**	**Loss**		
**四.终端消费量**	**Total Final Consumption**	**64.79**	**4558.26**
1.农、林、牧、渔业	Agriculture, Forestry, Animal Husbandry and Fishery		
2.工业	Industry	64.79	457.88
#用作原料、材料	Non-Energy Use	60.97	398.42
3.建筑业	Construction		3954.66
4.交通运输、仓储和邮政业	Transport, Storage and Post		145.72
5.批发和零售业、住宿和餐饮业	Wholesale and Retail Trades, Hotels and Catering Services		
6.其他	Others		
7.居民生活	Residential		
城镇	Urban		
乡村	Rural		
**五.平衡差额**	**Statistical Difference**	**-2.07**	**91.81**
**六.消费量合计**	**Total Energy Consumption**		

(C)1994-2024 China Academic Journal Electronic Publishing House. All rights reserved. http://www.cnki.net

Continued 2

(10⁴ tce)

石油焦<br>Petroleum Coke	液化石油气<br>Liquefied Petroleum Gas	炼厂干气<br>Refinery Gas	其他石油制品<br>Other Petroleum Products	天然气<br>Natural Gas	液化天然气<br>Liquefied Natural Gas	热力<br>Heat	电力<br>Electricity	其他能源<br>Other Energy
**889.92**	**3243.57**		**-1963.83**	**29996.88**	**11735.36**		**29886.45**	**7214.43**
				24564.68			30095.89	7214.43
							16655.52	
							4501.27	
							5732.96	
1079.05	3436.54		358.63	6081.08	11745.69		58.39	
187.27	162.66		10.46	648.88	10.33		267.84	
-1.85	-30.31		-2312.01					
**2577.00**	**6720.99**	**2979.58**	**14004.53**	**-9646.49**	**1640.36**	**20352.30**	**65508.75**	**-1325.91**
-199.37	-0.24	-78.86	-10.35	-5249.55	-620.97	-4359.14	65508.75	-2017.84
-269.38	-13.21	-259.59	-18.55	-2165.00	-85.90	20934.21		-689.73
-4.83								
3104.75	7695.49	3690.60	25714.06	-401.00				-415.24
-44.45	-954.67	-347.58	-11680.62					
-9.71	-6.38	-24.98		892.58				768.20
				-193.58				
				-2529.93	2347.23			
						3777.23		1028.70
	**1.08**			**333.45**	**2.59**	**177.48**	**3973.95**	
**3321.58**	**9689.34**	**2928.41**	**10977.70**	**20019.77**	**13366.45**	**20157.52**	**91421.24**	**5590.70**
	11.09			16.34		3.82	1747.77	666.23
3321.58	4279.46	2928.41	10862.97	7847.77	12402.15	14056.59	60368.43	990.53
2544.19	1485.89	199.80	5822.55	1115.83	287.02			105.78
	19.43		114.73	33.70		65.92	1242.65	28.55
	189.63			3474.24	964.31	133.16	2151.96	1208.26
	147.20			792.95		354.83	3894.74	64.00
	139.03			708.91		723.19	8009.42	245.50
	4903.50			7145.86		4820.01	14006.28	2387.64
	3613.97			7065.49		4820.01	7567.21	238.00
	1289.53			80.37			6439.07	2149.64
**145.34**	**274.14**	**51.17**	**1063.00**	**-2.83**	**6.67**	**17.30**		**297.82**

(C)1994-2024 China Academic Journal Electronic Publishing House. All rights reserved. http://www.cnki.net

5-3 综合能源平衡表

单位：万吨标准煤

项　　目	Item	1980	1985	1990
**可供消费的能源总量**	**Total Energy Available for Consumption**	**61557**	**77603**	**96138**
一次能源生产总量	Total Primary Energy Production	63735	85546	103922
回收能	Recovery of Energy			
进口量	Imports	261	340	1310
出口量(-)	Exports (-)	3058	5774	5875
年初年末库存差额	Stock Changes in the Year	619	-2509	-3219
**能源消费总量**	**Total Energy Consumption**	**60275**	**76682**	**98703**
在总量中:	Consumption by Sector			
1.农、林、牧、渔业	Agriculture, Forestry, Animal Husbandry and Fishery	4692	4045	4852
2.工业	Industry	38986	51068	67578
3.建筑业	Construction	957	1302	1213
4.交通运输、仓储和邮政业	Transport, Storage and Post	2902	3713	4541
5.批发和零售业、住宿和餐饮业	Wholesale and Retail Trades, Hotels and Catering Services	518	766	1247
6.其他	Others	1205	2470	3473
7.居民生活	Residential	11015	13318	15799
在总量中:	Consumption by Usage			
(一) 终端消费	(I) Final Consumption	57508	73586	94289
#工业	Industry	38293	48021	63239
(二) 加工转换损失量	(II) Losses During the Process of Energy Transformation	1358	1491	2264
#炼焦	Coking	644	572	905
炼油及煤制油	Petroleum Refining and Coal-to-liquids	113	110	326
(三) 回收能(-)	(III) Recovery of Energy(-)			
(四) 损失量	(IV) Other Losses	1409	1605	2150
**平衡差额**	**Balance**	**1282**	**921**	**-2565**

注：1.电力按等价热值折算，因此加工转换损失量中不包括发电损失量。
2.进口量包括境内飞机和轮船在境外的加油量；出口量包括境外飞机和轮船在境内的加油量。

(C)1994-2024 China Academic Journal Electronic Publishing House. All rights reserved. http://www.cnki.net

Overall Energy Balance Sheet

(10[4] tce)

1995	2000	2005	2010	2015	2016	2017	2018	2019	2020
**129535**	**144234**	**254619**	**365588**	**431636**	**434121**	**450444**	**471686**	**493178**	**507479**
129034	138570	229037	312125	362193	345954	358867	378859	397317	407295
2312	3087	7452	8958						
5456	14327	26823	57671	77695	90235	100039	110787	119064	124805
6776	9327	11257	8803	9785	11956	12669	13337	14151	12838
-491	-2424	2564	-4363	1532	9888	4206	-4623	-9052	-11784
**131176**	**146964**	**261369**	**360648**	**434113**	**441492**	**455827**	**471925**	**487488**	**498314**
5505	4233	6860	7266	8271	8585	8945	8781	9018	9263
96191	103014	187914	261377	295953	295615	302308	311151	322503	332625
1335	2207	3486	5533	7545	7847	8243	8685	9142	9320
5863	11447	19136	27102	38510	39883	42140	43617	43909	41309
2018	3251	5917	7847	11447	12042	12456	12994	13624	13171
4519	6118	10484	15052	21925	23185	24277	26262	27582	28245
15745	16695	27573	36470	50461	54336	57459	60436	61709	64380
124252	140476	250877	337469	420110	428342	442255	461020	476219	488156
89473	96871	177775	238652	282291	282809	289098	300558	311542	322677
3634	2472	3882	14294	18770	18674	19279	20803	22156	23020
	526	855	1595	4261	3887	3721	4129	4209	3938
	781	1273	1960	2866	3543	4280	5027	5895	6132
				14492	15373	15921	20465	21350	23037
3289	4016	6610	8885	9724	9849	10213	10567	10462	10175
**-1641**	**-2730**	**-6751**	**4940**	**-2477**	**-7371**	**-5383**	**-239**	**5690**	**9165**

Note: a) Electric power is converted on the basic of equal caloric value. Therefore, losses during the process of energy transformation exclude losses in power generation.

b) Data on imports include the domestic airplanes and ships refueling abroad. Data on exports include the oversea airplanes and ships refueling domestically.

(C)1994-2024 China Academic Journal Electronic Publishing House. All rights reserved. http://www.cnki.net

5-4 煤炭平衡表

单位: 万吨

项　　目	Item	1980	1985	1990
**可供量**	**Total Energy Available for Consumption**	**62601**	**82777**	**102221**
生产量	Production	62015	87228	107988
进口量	Imports	199	231	200
出口量(–)	Exports (-)	632	777	1729
年初年末库存差额	Stock Changes in the Year	1019	-3906	-4239
**消费量**	**Total Energy Consumption**	**61010**	**81603**	**105523**
在消费量中:	Consumption by Sector			
1.农、林、牧、渔业	Agriculture, Forestry, Animal Husbandry and Fishery	1550	2209	2095
2.工业	Industry	43848	58613	81091
3.建筑业	Construction	556	532	438
4.交通运输、仓储和邮政业	Transport, Storage and Post	1934	2307	2161
5.批发和零售业、住宿和餐饮业	Wholesale and Retail Trades, Hotels and Catering Services	455	738	1058
6.其他	Others	1091	1580	1980
7.居民生活	Residential	11574	15624	16700
在消费量中:	Consumption by Usage			
(一) 终端消费	(I) Final Consumption	38804	52704	60206
#工业	Industry	21643	29715	35774
(二) 中间消费	(II) Intermediate Consumption			
(用于加工转换)	(Consumed in Transformation)	22205	28899	41258
#火力发电	Thermal Power	12648	16441	27204
供热	Heating		1462	2996
炼焦	Coking	6682	7304	10698
煤制油	Coal-to-liquids			
制气	Gas Production	131	191	360
(三)洗选损耗	(III) Losses in Coal Washing and Dressing	2744	3501	4059
**平衡差额**	**Balance**	**1592**	**1174**	**-3302**

注：生产量为原煤产量。

(C)1994-2024 China Academic Journal Electronic Publishing House. All rights reserved. http://www.cnki.net

Coal Balance Sheet

(10^4 tons)

1995	2000	2005	2010	2015	2016	2017	2018	2019	2020
**133462**	**131895**	**235508**	**355578**	**397074**	**378494**	**383480**	**394848**	**405537**	**414519**
136073	138418	236515	342845	374654	341060	352356	369774	384633	390158
164	218	2622	18307	20406	25555	27093	28210	29977	30361
2862	5506	7173	1911	534	879	809	494	603	319
87	-1235	3545	-3663	2547	12758	4839	-2642	-8470	-5680
**137677**	**135690**	**243375**	**349008**	**399834**	**388820**	**391403**	**397452**	**401915**	**404860**
1857	1051	1802	2147	2625	2778	2834	2363	2202	2254
117571	121807	224766	329728	378190	367435	371160	380696	387268	390891
440	537	604	731	878	805	733	650	640	639
1315	882	811	639	492	404	353	321	283	241
977	1461	2627	3192	3864	3826	3461	2686	2378	1981
1987	1495	2727	3412	4159	4081	3580	3021	2598	2571
13530	8457	10039	9159	9627	9492	9283	7714	6547	6283
66156	50511	86386	114826	112975	101569	92841	81171	73449	72426
46050	36628	67776	95546	91331	80183	72598	64415	58802	58457
69488	81987	152208	222948	267061	272512	285325	303986	316252	321133
44440	55811	103663	153742	179568	182666	193925	205197	210159	211635
5887	8794	13542	17553	24115	26577	28983	32388	34442	36933
18396	16496	33446	49950	60874	60649	58910	61603	65673	65968
			213	679	1105	1568	2497	3240	3047
764	960	1277	1040	1320	1212	1663	2010	2459	3309
2033	3191	4782	11235	19798	14740	13237	12295	12213	11301
**-4215**	**-3795**	**-7868**	**6569**	**-2760**	**-10326**	**-7924**	**-2604**	**3623**	**9659**

Note: Data on production refer to the raw coal production.

(C)1994-2024 China Academic Journal Electronic Publishing House. All rights reserved. http://www.cnki.net

5-5 焦炭平衡表

单位: 万吨

项　　目	Item	1980	1985	1990
**可供量**	**Total Energy Available for Consumption**	**4315.3**	**4689.7**	**7085.8**
生产量	Production	4343.0	4802.1	7328.3
进口量	Imports		2.1	
出口量(-)	Exports (-)	27.1	36.9	129.0
年初年末库存差额	Stock Changes in the Year	-0.6	-77.6	-113.5
**消费量**	**Total Energy Consumption**	**4303.0**	**4689.7**	**6914.7**
在消费量中:	Consumption by Sector			
1.农、林、牧、渔业	Agriculture, Forestry, Animal Husbandry and Fishery	10.6	20.8	60.1
2.工业	Industry	4266.7	4627.7	6808.8
3.建筑业	Construction	11.9	7.8	5.2
4.交通运输、仓储和邮政业	Transport, Storage and Post	8.2	5.7	4.1
5.批发和零售业、住宿和餐饮业	Wholesale and Retail Trades, Hotels and Catering Services	0.9	2.7	7.7
6.其他	Others	4.7	2.0	1.9
7.居民生活	Residential		23.0	26.9
在消费量中:	Consumption by Usage			
(一) 终端消费	(I) Final Consumption	4294.7	4677.9	6846.3
#工业	Industry	4258.4	4615.9	6740.4
(二) 中间消费	(II) Intermediate Consumption			
(用于加工转换)	(Consumed in Transformation)	8.3	11.8	68.4
制气	Gas Production	8.3	11.8	68.4
(三) 损失量	(III) Losses in Coal Washing and Dressing			
**平衡差额**	**Balance**	**12.3**		**171.1**

(C)1994-2024 China Academic Journal Electronic Publishing House. All rights reserved. http://www.cnki.net

Coke Balance Sheet

(10⁴ tons)

1995	2000	2005	2010	2015	2016	2017	2018	2019	2020
**12207.1**	**10892.3**	**25084.4**	**38707.1**	**44018.9**	**45428.6**	**43739.5**	**43257.9**	**46533.7**	**47312.0**
13424.5	12184.0	26511.7	38657.8	44822.5	44911.5	43168.4	44751.4	47295.8	47188.2
0.1		0.5	11.0	0.4	0.1	1.0	9.1	52.3	297.9
886.1	1519.7	1276.4	335.0	964.8	1011.9	807.9	975.8	652.3	349.2
-331.4	228.0	-151.4	373.3	160.8	1529.0	1378.0	-526.8	-162.2	175.1
**10725.3**	**10840.8**	**25105.8**	**38702.8**	**44058.7**	**45462.4**	**43743.1**	**43716.6**	**46426.0**	**48310.4**
128.6	70.9	63.5	46.8	49.5	53.1	38.4	103.0	59.7	23.2
10412.0	10554.6	24860.9	38598.7	43923.0	45324.7	43609.1	43560.9	46320.2	48272.2
10.8	19.0	18.4	5.8	6.7	7.1	12.6	10.6	9.7	3.6
10.1	11.2	1.1	0.1	3.0	3.2	6.0	0.4	0.4	
25.7	35.7	64.1	5.1	40.1	41.3	49.4	19.0	16.9	
6.4	12.2	7.6	2.8	5.4	5.6	5.9	6.3	6.3	
131.6	137.2	90.3	43.5	31.2	27.4	21.8	16.4	12.8	11.4
10648.0	10697.9	24877.9	38574.6	43775.0	45454.4	43741.5	43693.7	46336.6	47762.5
10334.7	10411.7	24633.0	38470.5	43639.2	45316.7	43607.4	43538.0	46230.9	47724.3
77.3	142.9	227.9	128.2	283.8	8.0	1.6	22.8	89.3	547.9
77.3	142.9	227.9	128.2	1.8	1.9	1.6	1.6	16.5	17.6
**1481.8**	**51.6**	**-21.4**	**4.3**	**-39.8**	**-33.8**	**-3.6**	**-458.6**	**107.7**	**-998.4**

(C)1994-2024 China Academic Journal Electronic Publishing House. All rights reserved. http://www.cnki.net

5-6 石油平衡表

单位：万吨

项　目	Item	1980	1985	1990
**可供量**	**Total Energy Available for Consumption**	**8794.5**	**9193.7**	**11435.0**
生产量	Production	10594.6	12489.5	13830.6
进口量	Imports	82.7	90.0	755.6
出口量(-)	Exports (-)	1806.2	3630.4	3110.4
年初年末库存差额	Stock Changes in the Year	-76.6	244.6	-40.8
**消费量**	**Total Energy Consumption**	**8757.4**	**9168.8**	**11485.6**
在消费量中:	Consumption by Sector			
1.农、林、牧、渔业	Agriculture, Forestry, Animal Husbandry and Fishery	814..9	758.7	1033.6
2.工业	Industry	6203.2	6171.4	7321.6
3.建筑业	Construction	175.2	292.2	327.3
4.交通运输、仓储和邮政业	Transport, Storage and Post	911.5	1176.4	1683.2
5.批发和零售业、住宿和餐饮业	Wholesale and Retail Trades, Hotels and Catering Services	29.0	38.1	77.6
6.其他	Others	481.7	506.1	757.8
7.居民生活	Residential	141.9	225.9	284.5
在消费量中:	Consumption by Usage			
(一) 终端消费	(I) Final Consumption	6311.0	7063.3	9304.7
#工业	Industry	3780.3	4462.0	5180.4
(二) 中间消费	(II) Intermediate Consumption			
(用于加工转换)	(Consumed in Transformation)	2183.6	1858.5	1630.4
火力发电	Thermal Power	2065.4	1425.5	1234.4
供热	Heating		285.6	356.3
制气	Gas Production	36.7	34.5	39.7
炼油损失量	Losses in Petroleum Refining	81.5	112.9	295.8
(三) 损失量	(III) Other Losses	262.8	247.0	254.7
**平衡差额**	**Balance**	**37.1**	**24.9**	**-50.6**

注：1.生产量为原油产量。
2.进口量包括境内飞机和轮船在境外的加油量；出口量包括境外飞机和轮船在境内的加油量。

(C)1994-2024 China Academic Journal Electronic Publishing House. All rights reserved. http://www.cnki.net

Petroleum Balance Sheet

(10^4 tons)

1995	2000	2005	2010	2015	2016	2017	2018	2019	2020
**16072.7**	**22631.4**	**32539.1**	**44178.4**	**55688.0**	**57710.6**	**60810.8**	**63726.6**	**66900.9**	**67553.7**
15004.4	16300.0	18135.3	20301.4	21455.6	19968.5	19150.6	18932.4	19101.4	19476.9
3673.2	9748.5	17163.2	29437.2	39748.6	44502.9	49141.2	54094.3	58102.2	61271.7
2454.5	2172.1	2888.1	4079.0	5128.2	6382.9	7026.7	7557.4	8211.4	7551.0
-151.0	-1245.0	128.8	-1481.2	-388.1	-377.8	-454.3	-1742.7	-2091.4	-5643.8
**16064.9**	**22495.9**	**32547.0**	**44101.0**	**55960.2**	**57692.9**	**60395.9**	**62245.1**	**64506.5**	**65369.1**
1203.2	788.5	1451.7	1382.5	1733.4	1730.3	1786.4	1724.9	1748.2	1773.1
9349.3	11248.5	14030.4	18555.0	19718.0	20382.5	21486.7	22460.3	25210.6	27711.1
242.8	840.6	1502.2	2483.1	3384.3	3599.1	3803.5	3935.7	4055.1	4180.3
2863.6	6399.0	10928.5	15079.3	20663.1	21146.1	22075.8	22738.6	22109.6	20481.4
333.9	247.0	375.6	481.0	615.7	584.9	601.1	599.0	608.4	583.3
1390.3	1635.9	1974.2	2578.2	3683.3	3537.1	3502.7	3458.2	3460.6	3469.9
682.0	1336.5	2284.4	3541.9	6162.2	6712.8	7139.7	7328.4	7314.0	7170.1
13676.3	19950.1	29495.6	41243.4	52945.7	54387.0	56880.0	58623.0	61018.5	62085.2
7095.5	8860.0	11107.5	15857.8	16739.7	17100.2	17980.5	18847.6	21732.4	24428.4
2230.0	2352.9	2896.0	2663.3	2926.9	3264.3	3468.9	3593.3	3453.6	3265.6
1358.5	1178.2	1306.4	385.3	265.5	284.6	280.6	309.3	308.3	321.5
399.9	427.0	429.1	593.1	493.2	517.8	522.6	590.2	653.8	677.7
51.6	25.9	14.4			4.8	4.7	4.7	3.7	28.9
420.1	721.9	1146.1	1684.8	2168.2	2457.2	2661.0	2689.0	2487.9	2232.9
158.6	192.9	155.4	194.4	87.6	41.6	47.0	28.8	34.5	18.3
**7.8**	**135.4**	**-7.9**	**77.4**	**-272.2**	**17.7**	**414.9**	**1481.5**	**2394.3**	**2184.6**

Note: a) Data on production refer to the crude oil production.
b) Data on imports include the domestic airplanes and ships refueling abroad. Data on exports include the oversea airplanes and ships refueling domestically.

(C)1994-2024 China Academic Journal Electronic Publishing House. All rights reserved. http://www.cnki.net

5-7 原油平衡表

单位：万吨

项　　目	Item	1980	1985	1990
**可供量**	**Total Energy Available for Consumption**	**9222.9**	**9516.5**	**11770.6**
生产量	Production	10594.6	12489.5	13830.6
进口量	Imports	36.6		292.3
出口量(-)	Exports (-)	1330.9	3003.0	2399.0
年初年末库存差额	Stock Changes in the Year	-77.4	30.0	46.7
**消费量**	**Total Energy Consumption**	**9205.0**	**9509.5**	**11762.2**
在消费量中:	Consumption by Sector			
1.农、林、牧、渔业	Agriculture, Forestry, Animal Husbandry and Fishery	8.0	0.8	0.2
2.工业	Industry	9112.0	9389.9	11653.8
3.建筑业	Construction	28.8	74.0	55.2
4.交通运输、仓储和邮政业	Transport, Storage and Post	50.1	44.3	52.1
5.批发和零售业、住宿和餐饮业	Wholesale and Retail Trades, Hotels and Catering Services		0.1	0.3
6.其他	Others	6.1	0.4	0.6
7.居民生活	Residential			
在消费量中:	Consumption by Usage			
(一) 终端消费	(I) Final Consumption	499.6	350.4	402.1
#工业	Industry	429.7	254.9	333.4
(二) 中间消费	(II) Intermediate Consumption			
(用于加工转换)	(Consumed in Transformation)	8443.0	8929.7	11106.9
火力发电	Thermal Power	574.0	279.5	124.6
供热	Heating		61.3	21.1
炼油	Petroleum Refineries	7869.0	8588.9	10961.2
(三) 油田原油损失量	(III) Losses in Oil Field for Crude Oil	262.4	229.4	253.2
**平衡差额**	**Balance**	**17.9**	**7.0**	**8.4**

(C)1994-2024 China Academic Journal Electronic Publishing House. All rights reserved. http://www.cnki.net

Crude Oil Balance Sheet

(10^4 tons)

1995	2000	2005	2010	2015	2016	2017	2018	2019	2020
**14794.9**	**21383.0**	**30089.2**	**42876.6**	**54593.5**	**57332.3**	**59969.0**	**63849.2**	**68007.5**	**69969.6**
15004.4	16300.0	18135.3	20301.4	21455.6	19968.5	19150.6	18932.4	19101.4	19476.9
1709.0	7026.5	12681.7	23768.2	33548.3	38100.7	41946.2	46188.5	50567.6	54200.7
1822.7	1030.6	806.7	303.0	286.6	294.1	486.1	262.7	81.0	163.8
-95.8	-912.9	78.8	-890.0	-123.8	-442.8	-641.8	-1009.1	-1580.5	-3544.1
**14886.4**	**21232.0**	**30088.9**	**42874.6**	**54788.3**	**57125.9**	**59402.2**	**63004.3**	**67268.3**	**69477.1**
10.1									
14716.3	21052.1	29962.1	42716.6	54752.4	57103.6	59393.5	62995.5	67259.1	69476.5
2.7	3.3								
156.8	175.1	126.9	158.0	35.9	22.3	8.7	8.8	9.2	0.6
0.5	0.2								
1390.3	1.4								
309.9	636.8	850.4	806.1	782.7	630.1	364.7	348.3	326.5	419.0
274.7	612.3	850.4	806.1	782.7	630.1	364.7	348.3	326.5	419.0
14419.4	20404.3	29084.8	41876.4	53918.4	56455.4	58991.5	62627.7	66907.9	69040.5
61.6	85.0	41.3	3.7	12.5	13.1	14.2	15.3	16.0	16.5
4.4	14.0	3.0	3.3	6.7					
14353.4	20305.3	29040.5	41869.4	53899.2	56442.3	58977.3	62612.4	66891.9	69023.9
157.1	190.9	153.8	192.0	87.2	40.4	46.0	28.3	33.8	17.6
**-91.5**	**151.0**	**0.2**	**2.1**	**-194.8**	**206.4**	**566.8**	**844.8**	**739.2**	**492.5**

(C)1994-2024 China Academic Journal Electronic Publishing House. All rights reserved. http://www.cnki.net

5-8 燃料油平衡表

单位: 万吨

项　　目	Item	1980	1985	1990
**可供量**	**Total Energy Available for Consumption**	**3096.1**	**2848.0**	**3320.7**
生产量	Production	3142.0	2835.8	3267.9
进口量	Imports	39.0	70.0	167.3
出口量(–)	Exports (-)	45.4	64.9	97.2
年初年末库存差额	Stock Changes in the Year	-39.5	7.1	-17.3
**消费量**	**Total Energy Consumption**	**3073.7**	**2837.4**	**3367.8**
在消费量中:	Consumption by Sector			
1.农、林、牧、渔业	Agriculture, Forestry, Animal Husbandry and Fishery	2.3	3.1	2.9
2.工业	Industry	2937.4	2662.2	3091.7
3.建筑业	Construction	15.0	18.9	47.3
4.交通运输、仓储和邮政业	Transport, Storage and Post	109.0	144.1	208.2
5.批发和零售业、住宿和餐饮业	Wholesale and Retail Trades, Hotels and Catering Services	2.9	3.1	1.6
6.其他	Others	7.1	6.0	16.1
7.居民生活	Residential			
在消费量中:	Consumption by Usage			
(一) 终端消费	(I) Final Consumption	1617.9	1538.8	2042.6
#工　业	Industry	1481.6	1363.5	1766.5
(二) 中间消费	(II) Intermediate Consumption			
(用于加工转换)	(Consumed in Transformation)	1455.8	1296.1	1325.2
火力发电	Thermal Power	1419.1	1042.3	977.3
供　热	Heating		219.3	308.3
炼油再投入量	Petroleum Production			
制　气	Gas Production	36.7	34.5	39.6
(三)损失量	(III) Other Losses		2.5	
**平衡差额**	**Balance**	**22.4**	**10.6**	**-47.1**

(C)1994-2024 China Academic Journal Electronic Publishing House. All rights reserved.　http://www.cnki.net

Fuel Oil Balance Sheet

(10⁴ tons)

1995	2000	2005	2010	2015	2016	2017	2018	2019	2020
**3717.3**	**3836.7**	**4237.3**	**3765.4**	**4632.8**	**4644.5**	**4895.8**	**4585.2**	**4851.0**	**5344.3**
2960.8	2053.7	1767.4	2487.0	3963.0	4236.9	4563.5	3899.7	4506.0	5780.7
859.1	1704.3	2883.9	2695.2	2068.2	1743.2	1915.3	2271.3	2028.5	1760.9
68.6	57.9	427.6	1419.7	1402.1	1342.0	1516.7	1673.2	1548.5	2132.2
-34.0	136.6	13.5	3.0	3.7	6.4	-66.2	87.4	-135.0	-65.1
**3693.7**	**3872.8**	**4244.2**	**3758.0**	**4662.0**	**4631.0**	**4887.3**	**4536.1**	**4690.3**	**5364.6**
8.4	0.4	0.7	1.1	0.9	1.0	1.3	1.3	1.2	1.1
3406.2	2975.1	2986.9	2377.3	3133.0	3035.4	3043.7	2688.2	2612.5	3262.3
14.2	16.7	14.2	30.8	53.5	51.9	43.2	31.8	31.8	40.7
227.5	850.0	1201.0	1326.7	1439.5	1511.4	1771.3	1795.7	2025.3	2042.0
6.6	11.6	27.5	8.6	19.0	17.2	15.1	10.1	10.2	11.8
30.8	19.0	13.9	13.5	16.1	14.1	12.5	9.0	9.2	6.6
2262.8	2741.4	2989.9	2403.2	2123.7	2060.4	2196.9	2155.9	2295.4	2333.0
1975.3	1843.7	1732.6	1022.5	594.8	464.8	353.4	308.0	217.6	230.7
1430.9	1131.3	1254.3	1354.8	2538.3	2570.7	2690.4	2380.2	2395.0	3031.6
1071.5	814.2	1068.7	123.9	31.5	31.1	16.6	15.1	13.9	29.4
307.8	291.2	171.1	201.3	165.1	158.5	67.0	59.5	60.2	231.4
			1029.6	2341.6	2381.1	2606.7	2305.5	2320.8	2770.8
51.6	25.9	14.4							
**23.6**	**-36.1**	**-6.9**	**7.4**	**-29.2**	**13.5**	**8.5**	**49.1**	**160.7**	**-20.3**

(C)1994-2024 China Academic Journal Electronic Publishing House. All rights reserved. http://www.cnki.net

5-9 汽油平衡表

单位: 万吨

项　　目	Item	1980	1985	1990
**可供量**	**Total Energy Available for Consumption**	**999.4**	**1399.6**	**1884.1**
生产量	Production	1079.0	1471.9	2173.4
进口量	Imports		0.3	16.9
出口量(-)	Exports (-)	117.8	129.9	233.8
年初年末库存差额	Stock Changes in the Year	38.2	57.3	-72.4
**消费量**	**Total Energy Consumption**	**998.6**	**1396.3**	**1899.5**
在消费量中:	Consumption by Sector			
1.农、林、牧、渔业	Agriculture, Forestry, Animal Husbandry and Fishery	53.3	122.3	145.9
2.工业	Industry	273.2	451.3	589.3
3.建筑业	Construction	54.1	73.0	89.5
4.交通运输、仓储和邮政业	Transport, Storage and Post	404.9	477.4	620.1
5.批发和零售业、住宿和餐饮业	Wholesale and Retail Trades, Hotels and Catering Services	19.4	23.4	46.0
6.其他	Others	193.7	238.3	390.7
7.居民生活	Residential		10.6	18.0
**平衡差额**	**Balance**	**0.8**	**3.3**	**-15.4**

5-10 煤油平衡表

单位: 万吨

项　　目	Item	1980	1985	1990
**可供量**	**Total Energy Available for Consumption**	**359.0**	**383.2**	**350.9**
生产量	Production	398.5	405.3	392.5
进口量	Imports		15.2	26.1
出口量(-)	Exports (-)	46.8	46.0	55.5
年初年末库存差额	Stock Changes in the Year	2.3	8.7	-12.2
**消费量**	**Total Energy Consumption**	**365.9**	**385.5**	**350.9**
在消费量中:	Consumption by Sector			
1.农、林、牧、渔业	Agriculture, Forestry, Animal Husbandry and Fishery	2.3	3.3	3.1
2.工业	Industry	15.7	20.1	20.6
3.建筑业	Construction	0.8	1.3	1.3
4.交通运输、仓储和邮政业	Transport, Storage and Post	31.4	56.2	93.4
5.批发和零售业、住宿和餐饮业	Wholesale and Retail Trades, Hotels and Catering Services	0.2	0.1	0.6
6.其他	Others	216.7	182.9	127.3
7.居民生活	Residential	98.8	121.6	104.6
**平衡差额**	**Balance**	**-6.9**	**-2.3**	

(C)1994-2024 China Academic Journal Electronic Publishing House. All rights reserved. http://www.cnki.net

Gasoline Balance Sheet

(10^4 tons)

1995	2000	2005	2010	2015	2016	2017	2018	2019	2020
**2902.0**	**3504.5**	**4855.3**	**6964.3**	**11385.0**	**11829.4**	**12200.5**	**13035.3**	**13690.3**	**12829.0**
3051.6	4134.7	5433.6	7410.5	12103.6	12932.0	13276.2	14264.7	14880.7	14256.7
15.9				17.0	20.8	1.6	44.5	33.3	48.0
193.1	467.7	559.7	517.0	589.3	969.3	1051.4	1287.9	1637.1	1600.0
27.6	-162.5	-18.6	70.8	-146.3	-154.2	-26.0	13.9	413.3	124.3
**2909.6**	**3504.6**	**4854.9**	**6956.2**	**11368.5**	**11866.0**	**12296.3**	**13055.3**	**13628.0**	**12767.2**
179.7	89.2	159.6	169.1	231.3	224.4	229.6	242.9	253.2	257.3
812.4	682.0	441.7	689.5	477.1	436.3	382.1	296.5	262.0	184.0
103.6	115.6	172.1	274.7	408.6	437.3	452.3	505.0	499.9	508.4
982.3	1527.8	2430.1	3274.9	5306.6	5511.1	5698.5	6067.6	6244.9	5573.6
197.2	69.8	129.4	168.2	243.3	240.9	244.5	275.5	287.9	273.2
570.7	792.7	998.2	1166.2	2108.5	2046.4	2075.0	2163.6	2240.9	2253.1
63.7	227.6	523.8	1213.7	2593.1	2969.7	3214.2	3504.2	3839.1	3717.6
**-7.6**	**-0.1**	**0.4**	**8.1**	**16.6**	**-36.7**	**-95.8**	**-20.0**	**62.3**	**61.9**

Kerosene Balance Sheet

(10^4 tons)

1995	2000	2005	2010	2015	2016	2017	2018	2019	2020
**486.4**	**880.9**	**1070.0**	**1767.6**	**2732.6**	**3020.7**	**3332.8**	**3714.3**	**4002.8**	**3412.4**
445.8	872.3	1006.5	1924.4	3658.6	3983.9	4230.9	4770.3	5322.6	4129.4
115.7	322.5	476.1	726.1	716.4	776.7	852.3	942.3	930.0	514.0
62.4	256.3	447.6	870.5	1626.6	1721.7	1765.2	1947.0	2239.8	1226.3
-12.7	-57.6	35.0	-12.3	-15.8	-18.2	14.8	-51.3	-10.0	-4.8
**512.1**	**871.6**	**1076.8**	**1765.2**	**2663.7**	**2970.7**	**3326.4**	**3653.5**	**3950.2**	**3352.1**
3.6	1.5	1.6	0.9	1.1	2.2	1.5	4.9	11.0	11.0
44.9	84.0	57.5	40.2	21.2	20.0	14.5	24.9	11.0	9.4
3.5	4.0		8.8	12.5	10.0	9.7	17.3	16.0	10.8
250.0	535.9	952.4	1601.1	2504.9	2814.9	3173.3	3462.5	3689.2	3110.8
8.5	14.0	3.7	35.0	11.7	11.2	11.3	15.5	15.5	14.8
137.3	160.1	36.2	58.7	83.3	85.9	88.4	103.8	184.2	183.0
64.3	72.2	25.5	20.5	29.1	26.4	27.6	24.6	23.4	12.4
**-25.7**	**9.3**	**-6.8**	**2.4**	**68.9**	**50.0**	**6.4**	**60.8**	**52.6**	**60.3**

(C)1994-2024 China Academic Journal Electronic Publishing House. All rights reserved. http://www.cnki.net

5-11 柴油平衡表

单位：万吨

项　　目	Item	1980	1985	1990
**可供量**	**Total Energy Available for Consumption**	**1663.2**	**1944.1**	**2689.4**
生产量	Production	1827.8	2023.2	2609.0
进口量	Imports	2.1	4.5	233.8
出口量(-)	Exports (-)	166.5	225.6	169.8
年初年末库存差额	Stock Changes in the Year	-0.2	142.0	16.4
**消费量**	**Total Energy Consumption**	**1663.2**	**1939.4**	**2691.7**
在消费量中:	Consumption by Sector			
1.农、林、牧、渔业	Agriculture, Forestry, Animal Husbandry and Fishery	749.0	629.2	881.5
2.工业	Industry	457.4	644.1	728.1
3.建筑业	Construction	76.5	125.0	133.0
4.交通运输、仓储和邮政业	Transport, Storage and Post	316.1	454.4	709.4
5.批发和零售业、住宿和餐饮业	Wholesale and Retail Trades, Hotels and Catering Services	6.5	10.9	22.5
6.其他	Others	57.7	74.0	217.0
7.居民生活	Residential			
在消费量中:	Consumption by Usage			
(一) 终端消费	(I) Final Consumption	1590.9	1827.4	2564.8
#工　业	Industry	385.1	532.1	601.2
(二) 中间消费	(II) Intermediate Consumption			
(用于加工转换)	(Consumed in Transformation)	72.3	108.6	126.9
火力发电	Thermal Power	72.3	103.6	124.5
供　热	Heating		5.0	2.4
(三) 损失量	(III) Other Losses		3.4	
**平衡差额**	**Balance**		**4.7**	**-2.3**

(C)1994-2024 China Academic Journal Electronic Publishing House. All rights reserved. http://www.cnki.net

Diesel Oil Balance Sheet

(10⁴ tons)

1995	2000	2005	2010	2015	2016	2017	2018	2019	2020
**4404.2**	**6806.5**	**10972.6**	**14701.9**	**17353.6**	**16765.2**	**16994.5**	**16567.3**	**15359.6**	**14459.7**
3972.6	7079.6	11090.2	14924.4	18007.9	17917.7	18667.9	18360.1	17308.3	16529.9
645.3	51.9	61.0	190.2	71.5	116.1	107.6	89.0	144.5	137.5
169.5	77.5	170.9	490.2	731.3	1556.9	1736.8	1872.8	2189.1	2011.2
-44.2	-247.6	-7.7	77.5	5.5	288.3	-44.2	-9.0	95.9	-196.5
**4321.4**	**6806.2**	**10974.9**	**14699.0**	**17360.3**	**16839.0**	**16916.5**	**16409.6**	**14917.9**	**14282.7**
1001.4	697.1	1286.3	1206.7	1492.9	1495.9	1546.8	1468.2	1475.1	1497.2
1189.9	1696.5	1710.0	2090.0	1516.4	1412.9	1459.9	1259.5	1290.6	1026.1
118.2	205.9	386.6	490.2	555.7	561.3	596.1	543.4	530.3	503.9
1246.6	3293.8	6169.4	8657.6	11162.8	11068.5	11173.7	11166.9	9867.3	9532.0
103.6	95.9	116.0	196.6	257.7	232.0	233.8	211.8	203.9	197.6
645.7	638.7	900.1	1287.2	1384.2	1307.2	1233.3	1107.4	954.0	946.2
16.1	178.4	406.4	770.7	990.7	761.3	673.0	652.3	596.8	579.8
4070.0	6578.6	10889.4	14655.2	17280.4	16736.4	16722.4	16340.0	14805.6	14136.2
938.5	1468.8	1624.5	2046.2	1436.5	1310.3	1265.8	1189.9	1178.3	879.6
251.4	227.7	85.5	43.8	79.9	102.7	194.1	69.6	112.3	146.5
204.9	227.7	81.9	40.1	22.4	29.2	23.9	26.4	24.2	27.6
46.6		3.6	3.8	6.2	6.1	5.9	5.2	5.3	2.9
**82.7**	**0.3**	**-2.4**	**2.9**	**-6.7**	**-73.9**	**78.0**	**157.8**	**441.6**	**177.0**

(C)1994-2024 China Academic Journal Electronic Publishing House. All rights reserved. http://www.cnki.net

5-12 液化石油气平衡表

单位: 万吨

项目	Item	1980	1985	1990
**可供量**	**Total Energy Available for Consumption**	**122.5**	**157.3**	**258.5**
生产量	Production	122.5	159.7	261.6
进口量	Imports			
出口量(–)	Exports (-)		1.9	1.1
年初年末库存差额	Stock Changes in the Year		-0.5	-2.0
**消费量**	**Total Energy Consumption**	**119.6**	**155.7**	**254.2**
在消费量中:	Consumption by Sector			
1.农、林、牧、渔业	Agriculture, Forestry, Animal Husbandry and Fishery			
2.工业	Industry	76.1	59.9	82.0
3.建筑业	Construction			1.0
4.交通运输、仓储和邮政业	Transport, Storage and Post			
5.批发和零售业、住宿和餐饮业	Wholesale and Retail Trades, Hotels and Catering Services		0.5	6.6
6.其他	Others	0.4	4.5	6.1
7.居民生活	Residential	43.1	90.8	158.5
**平衡差额**	**Balance**	**2.9**	**1.6**	**4.3**

5-13 天然气平衡表

单位: 亿立方米

项目	Item	1980	1985	1990
**可供量**	**Total Energy Available for Consumption**	**142.7**	**129.3**	**153.0**
生产量	Production	142.7	129.3	153.0
进口量	Imports			
出口量(–)	Exports (-)			
年初年末库存差额	Stock Changes in the Year			
**消费量**	**Total Energy Consumption**	**140.6**	**129.3**	**152.5**
在消费量中:	Consumption by Sector			
1.农、林、牧、渔业	Agriculture, Forestry, Animal Husbandry and Fishery			
2.工业	Industry	131.4	109.6	120.2
3.建筑业	Construction	6.0	14.1	10.6
4.交通运输、仓储和邮政业	Transport, Storage and Post	0.7	0.8	1.9
5.批发和零售业、住宿和餐饮业	Wholesale and Retail Trades, Hotels and Catering Services			
6.其他	Others	0.5	0.5	1.2
7.居民生活	Residential	2.0	4.3	18.6
**平衡差额**	**Balance**	**2.1**		**0.5**

注：从2010年起包括液化天然气数据。

(C)1994-2024 China Academic Journal Electronic Publishing House. All rights reserved. http://www.cnki.net

Liquefied Petroleum Gas Balance Sheet

(10⁴ tons)

1995	2000	2005	2010	2015	2016	2017	2018	2019	2020
**774.3**	**1396.2**	**2052.2**	**2323.8**	**4008.2**	**5034.0**	**5472.9**	**5733.4**	**6160.6**	**6381.1**
540.8	916.6	1432.7	2092.3	2934.4	3503.9	3677.3	3915.6	4210.0	4489.0
232.6	481.7	617.0	327.0	1244.0	1678.5	1921.9	1966.4	2109.3	2004.6
7.1	1.6	2.7	93.0	144.2	132.3	132.2	113.5	140.9	94.9
8.0	-0.6	5.2	-2.5	-26.0	-16.0	5.9	-35.1	-17.8	-17.7
**750.6**	**1389.7**	**2046.5**	**2321.9**	**3961.2**	**5015.1**	**5457.8**	**5673.1**	**6066.4**	**6221.2**
0.1	0.4	3.5	4.7	7.2	6.8	7.1	7.6	7.8	6.5
192.5	426.1	534.4	586.8	1113.9	1766.8	1896.3	2215.5	2870.3	3064.8
0.5	8.9	6.3	7.2	15.1	14.8	15.8	17.0	14.0	11.3
0.5	16.5	48.7	61.0	100.3	104.2	123.7	125.1	156.5	111.2
17.4	55.5	99.0	72.6	84.0	83.6	96.4	86.2	90.9	85.9
5.7	24.0	25.8	52.6	91.4	83.5	93.5	74.4	72.2	81.1
534.0	858.3	1328.7	1537.0	2549.3	2955.4	3225.0	3147.3	2854.7	2860.4
**23.7**	**6.5**	**5.7**	**1.9**	**47.0**	**18.9**	**15.1**	**60.3**	**94.2**	**159.9**

Natural Gas Balance Sheet

(10⁸ cu.m)

1995	2000	2005	2010	2015	2016	2017	2018	2019	2020
**179.5**	**240.6**	**463.5**	**1082.3**	**1925.0**	**2080.5**	**2390.7**	**2814.3**	**3057.5**	**3270.2**
179.5	272.0	493.2	957.9	1346.1	1368.7	1480.4	1601.6	1761.7	1925.0
			164.7	611.4	745.6	945.6	1246.4	1331.8	1397.0
	31.4	29.7	40.3	32.5	33.8	35.3	33.6	36.1	51.7
**177.4**	**245.0**	**466.1**	**1080.2**	**1931.8**	**2078.1**	**2393.7**	**2817.1**	**3059.7**	**3339.9**
			0.5	0.9	1.1	1.1	1.3	1.2	1.3
154.4	199.0	327.2	691.8	1234.5	1338.6	1575.2	1940.1	2092.1	2304.0
0.3	0.8	1.5	1.2	2.2	1.9	1.8	2.5	2.8	2.6
1.6	8.8	38.0	106.7	237.6	254.8	284.7	286.2	341.5	354.3
0.6	3.4	10.8	27.2	51.3	53.7	57.6	60.8	62.5	62.1
1.2	0.6	9.1	26.0	45.4	48.2	52.9	57.9	57.3	55.6
19.4	32.3	79.4	226.9	359.8	379.7	420.3	468.4	502.3	560.0
**2.1**	**-4.4**	**-2.6**	**2.1**	**-6.7**	**2.4**	**-3.0**	**-2.8**	**-2.2**	**0.3**

Note: Include the data of Liquefied Natural Gas since 2010.

(C)1994-2024 China Academic Journal Electronic Publishing House. All rights reserved. http://www.cnki.net

5-14 电力平衡表

单位：亿千瓦时

项目	Item	1980	1985	1990
**可供量**	**Total Energy Available for Consumption**	**3006.3**	**4117.6**	**6230.4**
生产量	Production	3006.3	4106.9	6212.0
#水电	#Hydro Power	582.1	923.7	1267.2
火电	Thermal Power	2424.2	3183.2	4944.8
核电	Nuclear Power			
风电	Wind Power			
进口量	Imports		11.1	19.3
出口量(-)	Exports (-)		0.4	0.9
**消费量**	**Total Energy Consumption**	**3006.3**	**4117.6**	**6230.4**
在消费量中:	Consumption by Sector			
1.农、林、牧、渔业	Agriculture, Forestry, Animal Husbandry and Fishery	270.0	317.4	426.8
2.工业	Industry	2471.9	3283.4	4873.3
3.建筑业	Construction	47.1	71.2	65.0
4.交通运输、仓储和邮政业	Transport, Storage and Post	26.5	63.4	105.9
5.批发和零售业、住宿和餐饮业	Wholesale and Retail Trades, Hotels and Catering Services	16.8	38.0	76.2
6.其他	Others	68.8	121.7	202.4
7.居民生活	Residential	105.2	222.5	480.8
在消费量中:	Consumption by Usage			
(一) 终端消费	(I) Final Consumption	2763.4	3813.3	5795.8
#工业	#Industry	2229.0	2979.1	4438.7
(二) 输配电损失量	(II) Losses in Transmission	242.9	304.3	434.6

(C)1994-2024 China Academic Journal Electronic Publishing House. All rights reserved. http://www.cnki.net

Electricity Balance Sheet

(10[8] kW•h)

1995	2000	2005	2010	2015	2016	2017	2018	2019	2020
**10023.4**	**13472.7**	**24940.8**	**41936.5**	**58021.3**	**61204.4**	**65914.0**	**71509.2**	**74866.3**	**77620.2**
10077.3	13556.0	25002.6	42071.6	58145.7	61331.6	66044.5	71661.3	75034.3	77790.6
1905.8	2224.1	3970.2	7221.7	11302.7	11840.5	11978.7	12317.9	13044.4	13552.1
8043.2	11141.9	20473.4	33319.3	42841.9	44370.7	47546.0	50963.2	52201.5	53302.5
128.3	167.4	530.9	738.8	1707.9	2132.9	2480.7	2943.6	3483.5	3662.5
			446.2	1857.7	2370.7	2972.3	3659.7	4060.3	4664.7
6.4	15.5	50.1	55.5	62.1	61.9	64.2	56.9	48.6	47.5
60.3	98.8	111.9	190.6	186.5	189.1	194.7	209.1	216.5	217.9
**10023.4**	**13472.4**	**24940.3**	**41934.5**	**58020.0**	**61205.1**	**65914.0**	**71508.2**	**74866.1**	**77620.2**
582.4	533.0	776.3	976.5	1039.8	1091.9	1175.1	1242.5	1336.2	1422.1
7659.8	10004.6	18521.7	30871.8	41550.0	42996.9	46052.8	49094.9	50698.3	52353.4
159.6	159.8	233.9	483.2	698.7	725.6	789.2	887.8	991.2	1011.1
182.3	281.2	430.3	734.5	1125.6	1251.5	1418.0	1608.5	1752.3	1751.0
199.5	418.7	752.3	1292.0	2122.0	2323.8	2526.6	2900.4	3187.1	3169.0
234.2	623.2	1340.9	2451.8	3918.6	4394.8	4880.6	5716.5	6263.8	6517.0
1005.6	1452.0	2884.8	5124.6	7565.2	8420.6	9071.6	10057.6	10637.2	11396.5
9278.9	12535.7	23233.8	39366.3	55032.1	58142.2	62718.1	68156.5	71536.0	74386.7
6915.3	9067.9	16815.2	28303.5	38562.1	39934.0	42857.0	45743.2	47368.2	49120.0
744.5	936.7	1706.5	2568.2	2987.9	3062.9	3195.8	3351.7	3330.1	3233.5

(C)1994-2024 China Academic Journal Electronic Publishing House. All rights reserved. http://www.cnki.net
