# Supplementary material for: Prediction analysis of carbon emission in China’s electricity industry based on the dual carbon background
Source: PLoS One. 2024 May 17;19(5):e0302068. doi: 10.1371/journal.pone.0302068 (PMC11101092; doi:10.1371/journal.pone.0302068)
Supplement: S4 File — (ZIP) [file pone.0302068.s004.zip › China Energy Statistic Yearbook 2001-2021/五_全国能源平衡表_五_全国能源平衡表_文兼武总编_-2011.pdf]

5-1 中国能源平衡表(实物量) -2010

项 目	Item	煤合计 (万吨) Coal Total (10^4 tn)	原煤 (万吨) Raw Coal (10^4 tn)
**一.可供本地区消费的能源量**	**Total Primary Energy Supply**	**319771.98**	**322524.47**
1.一次能源生产量	Indigenous Production	323500.00	323500.00
水电	Hydro Power		
核电	Nuclear Power		
风电	Wind Power		
2.回收能	Recovery of Energy		
3.进口量	Import	16309.52	16306.50
4.境内轮船和飞机在境外的加油量	Domestic Airplanes&Ships Refueling in Abroad		
5.出口量(-)	Export (-)	-1910.37	-1903.00
6.境外轮船和飞机在境内的加油量(-)	Oversea Airplanes&Ships Refueling in China		
7.库存增(-)、减(+)量	Stock Change	-18127.17	-15379.03
**二.加工转换投入(-)产出(+)量**	**Input(-) & Output(+) of Transformation**	**-227885.57**	**-242393.85**
1.火力发电	Thermal Power	-154542.48	-151307.09
2.供　热	Heating Supply	-15253.08	-14565.47
3.洗 选 煤	Coal Washing	-9484.62	-69758.46
4.炼　焦	Coking	-47150.37	-5842.10
5.炼油及煤制油	Petroleum Refineries	-213.42	-114.30
#油品再投入量(-)	Petroleum Products Input (-)		
6.制　气	Gas Works	-1040.06	-603.38
#焦炭再投入量(-)	Coke Input (-)		
7.天然气液化	Natural Gas Liquefaction		
8.煤制品加工	Briquettes	-201.54	-203.05
**三.损 失 量**	**Loss**		
**四.终端消费量**	**Total Final Consumption**	**84350.93**	**73864.92**
1.农、林、牧、渔业	Farming, Forestry, Animal Husbandry, Fishery Conservancy	1711.10	1671.35
2.工业	Industry	68146.05	59538.89
#用作原料、材料	Non-Energy Use	4289.25	3915.52
3.建筑业	Construction	718.92	707.13
4.交通运输、仓储和邮政业	Transport, Storage and Post	639.23	612.99
5.批发、零售业和住宿、餐饮业	Wholesale, Retail Trade and Hotel, Restaurants	1969.87	1897.89
6.其他	Others	2006.59	1818.03
7.生活消费	Residential Consumption	9159.17	7618.64
城 镇	Urban	1902.68	1403.17
乡 村	Rural	7256.49	6215.47
**五.平衡差额**	**Statistical Difference**	**7535.48**	**6265.71**
**六.消费量合计**	**Total Final Consumption**	**312236.50**	**316258.76**

?1994-2018 China Academic Journal Electronic Publishing House. All rights reserved.　http://www.cnki.net

ENERGY BANLANCE OF CHINA -2010(PHYSICAL QUANTITY)

洗精煤 (万吨) Cleaned Coal (10^4 tn)	其他洗煤 (万吨) Other Washed Coal (10^4 tn)	型煤 (万吨) Briquettes (10^4 tn)	煤矸石 (万吨) Gangue (10^4 tn)	焦炭 (万吨) Coke (10^4 tn)	焦炉煤气 (亿立方米) Coke Oven Gas (10^8 cu.m)	高炉煤气 (亿立方米) Blast Furnace Gas (10^8 cu.m)	转炉煤气 (亿立方米) Converter Gas (10^8 cu.m)	其他煤气 (亿立方米) Other Gas (10^8 cu.m)
**-938.55**	**-1805.06**	**-8.88**		**-1950.74**		**2270.61**	**255.86**	
						2270.61	255.86	
		3.02		10.95				
		-7.37		-335.00				
-938.55	-1805.06	-4.53		-1626.69				
**4152.31**	**9268.46**	**1087.51**	**-0.08**	**36329.59**	**563.84**	**-1152.33**	**-78.45**	**174.50**
-4.69	-3230.70		-2226.54		-105.48	-792.00	-46.15	
-10.53	-677.08		-470.96		-72.83	-360.33	-32.30	
45949.21	14324.62		2697.42					
-41251.77	-56.49			36209.60	718.38			
-93.23	-5.89							
-436.68				248.23	23.77			174.50
				-128.24				
	-1086.00	1087.51						
**2541.78**	**6905.45**	**1038.79**		**33559.56**	**551.29**	**1110.00**	**179.02**	**168.61**
	39.75			46.82				
2529.37	5667.70	410.09		33455.45	494.55	1110.00	179.02	55.00
130.01	243.71			1338.45	4.95			
1.36	10.43			5.81				
11.05	15.19			0.12				
	39.13	32.86		5.10	0.80			
	177.11	11.46		2.77	2.68			
	956.15	584.38		43.49	53.26			113.61
	322.85	176.65		23.72	53.26			113.17
	633.30	407.73		19.77				0.44
**671.98**	**557.95**	**39.84**	**-0.08**	**819.29**	**12.55**	**8.28**	**-1.61**	**5.89**
**44338.68**	**11961.61**	**1038.79**	**2697.50**	**33687.80**	**705.83**	**2262.33**	**257.47**	**168.61**

?1994-2018 China Academic Journal Electronic Publishing House. All rights reserved. http://www.cnki.net

续表 1

项　目	Item	其他焦化产品(万吨) Other Coking Products (10^4 tn)	油品合计(万吨) Petroleum Products Total (10^4 tn)
**一.可供本地区消费的能源量**	**Total Primary Energy Supply**		**44178.43**
1.一次能源生产量	Indigenous Production		20301.40
水电	Hydro Power		
核电	Nuclear Power		
风电	Wind Power		
2.回收能	Recovery of Energy		
3.进口量	Import		28791.77
4.境内轮船和飞机在境外的加油量	Domestic Airplanes&Ships Refueling in Abroad		645.45
5.出口量(-)	Export (-)		-3357.54
6.境外轮船和飞机在境内的加油量(-)	Oversea Airplanes&Ships Refueling in China		-721.46
7.库存增(-)、减(+)量	Stock Change		-1481.19
**二.加工转换投入(-)产出(+)量**	**Input(-) & Output(+) of Transformation**	**682.68**	**-2657.06**
1.火力发电	Thermal Power		-459.14
2.供　热	Heating Supply		-593.12
3.洗 选 煤	Coal Washing		
4.炼　焦	Coking	693.31	
5.炼油及煤制油	Petroleum Refineries	-44.25	2264.01
#油品再投入量(-)	Petroleum Products Input (-)		-3868.81
6.制　气	Gas Works	44.85	
#焦炭再投入量(-)	Coke Input (-)	-11.23	
7.天然气液化	Natural Gas Liquefaction		
8.煤制品加工	Briquettes		
**三.损 失 量**	**Loss**		**194.40**
**四.终端消费量**	**Total Final Consumption**	**673.00**	**40393.70**
1.农、林、牧、渔业	Farming,Forestry,Animal Husbandry, Fishery Conservancy		1382.50
2.工业	Industry	673.00	14757.76
#用作原料、材料	Non-Energy Use		4246.75
3.建筑业	Construction		3045.11
4.交通运输、仓储和邮政业	Transport, Storage and Post		14709.86
5.批发、零售业和住宿、餐饮业	Wholesale, Retail Trade and Hotel, Restaurants		481.01
6.其他	Others		2556.66
7.生活消费	Residential Consumption		3460.80
城 镇	Urban		2517.38
乡 村	Rural		943.41
**五.平衡差额**	**Statistical Difference**	**9.68**	**933.28**
**六.消费量合计**	**Total Final Consumption**	**728.48**	**43245.15**

?1994-2018 China Academic Journal Electronic Publishing House. All rights reserved. http://www.cnki.net

Continued 1

原油 (万吨) Crude Oil (10^4 tn)	汽油 (万吨) Gasoline (10^4 tn)	煤油 (万吨) Kerosene (10^4 tn)	柴油 (万吨) Diesel Oil (10^4 tn)	燃料油 (万吨) Fuel Oil (10^4 tn)	石脑油 (万吨) Naphtha (10^4 tn)	润滑油 (万吨) Lubricants (10^4 tn)	石蜡 (万吨) Petroleum Waxes (10^4 tn)	溶剂油 (万吨) White Spirit (10^4 tn)
**42876.63**	**-446.17**	**-156.77**	**-222.52**	**1278.47**	**3.70**	**22.95**	**-51.10**	**-0.17**
20301.40								
23768.18	0.01	487.00	180.00	2299.00	290.70	34.20	0.90	3.36
		239.06	10.19	396.20				
-303.00	-517.00	-605.00	-464.00	-990.00	-87.00	-11.25	-52.00	-0.53
		-265.50	-26.24	-429.72				
-889.95	70.82	-12.33	77.53	2.99	-200.00			-3.00
**-41876.43**	**7360.38**	**1921.73**	**14806.74**	**1182.13**	**1931.57**	**218.94**	**160.11**	**74.33**
-3.71	-0.09		-113.86	-123.89				
-3.28			-3.78	-201.34				
-41869.44	7360.47	1924.39	14924.38	2536.97	1940.65	218.94	160.11	74.33
		-2.66		-1029.61	-9.08			
**192.00**								
**806.12**	**6886.12**	**1741.41**	**14516.17**	**2403.18**	**1841.02**	**225.00**	**103.00**	**70.00**
	169.07	0.90	1206.73	1.14				
806.12	689.37	37.54	2046.16	1022.48	1841.02	225.00	103.00	70.00
107.21	22.13				290.00	218.66	101.08	67.23
	274.70	8.77	490.20	30.76				
	3204.93	1601.08	8518.56	1326.65				
	168.18	34.98	196.60	8.62				
	1166.22	38.73	1287.19	13.53				
	1213.65	19.41	770.73					
	844.66	1.69	573.44					
	369.00	17.72	197.29					
**2.08**	**28.09**	**23.55**	**68.06**	**57.42**	**94.25**	**16.89**	**6.01**	**4.16**
**42874.55**	**6886.21**	**1744.07**	**14633.80**	**3758.02**	**1850.10**	**225.00**	**103.00**	**70.00**

?1994-2018 China Academic Journal Electronic Publishing House. All rights reserved. http://www.cnki.net

续表 2

项　目	Item	石油沥青 (万吨) Bitumen Asphalt (10^4 tn)	石油焦 (万吨) Petroleum Coke (10^4 tn)
**一.可供本地区消费的能源量**	**Total Primary Energy Supply**	**392.70**	**443.66**
1.一次能源生产量	Indigenous Production		
水电	Hydro Power		
核电	Nuclear Power		
风电	Wind Power		
2.回收能	Recovery of Energy		
3.进口量	Import	407.70	773.86
4.境内轮船和飞机在境外的加油量	Domestic Airplanes&Ships Refueling in Abroad		
5.出口量(-)	Export (-)	-15.00	-210.20
6.境外轮船和飞机在境内的加油量(-)	Oversea Airplanes&Ships Refueling in China		
7.库存增(-)、减(+)量	Stock Change		-120.00
**二.加工转换投入(-)产出(+)量**	**Input(-) & Output(+) of Transformation**	**1963.90**	**1399.46**
1.火力发电	Thermal Power		-109.47
2.供　热	Heating Supply		-115.58
3.洗 选 煤	Coal Washing		
4.炼　焦	Coking		
5.炼油及煤制油	Petroleum Refineries	1963.90	1624.51
#油品再投入量(-)	Petroleum Products Input (-)		
6.制　气	Gas Works		
#焦炭再投入量(-)	Coke Input (-)		
7.天然气液化	Natural Gas Liquefaction		
8.煤制品加工	Briquettes		
**三.损 失 量**	**Loss**		
**四.终端消费量**	**Total Final Consumption**	**2218.37**	**1800.00**
1.农、林、牧、渔业	Farming, Forestry, Animal Husbandry, Fishery Conservancy		
2.工业	Industry	44.87	1800.00
#用作原料、材料	Non-Energy Use		1582.71
3.建筑业	Construction	2173.50	
4.交通运输、仓储和邮政业	Transport, Storage and Post		
5.批发、零售业和住宿、餐饮业	Wholesale, Retail Trade and Hotel, Restaurants		
6.其他	Others		
7.生活消费	Residential Consumption		
城 镇	Urban		
乡 村	Rural		
**五.平衡差额**	**Statistical Difference**	**138.23**	**43.12**
**六.消费量合计**	**Total Final Consumption**	**2218.37**	**2025.05**

?1994-2018 China Academic Journal Electronic Publishing House. All rights reserved. http://www.cnki.net

Continued 2

液化石油气 (万吨) LPG (10⁴ tn)	炼厂干气 (万吨) Refinery Gas (10⁴ tn)	其他石油制品 (万吨) Other Petroleum Products (10⁴ tn)	天然气 (亿立方米) Natural Gas (10⁸ cu.m)	液化天然气 (万吨) LNG (10⁴ tn)	热力 (万百万千焦) Heat (10¹⁰ kJ)	电力 (亿千瓦小时) Electricity (10⁸ kW•h)	其他能源 (万吨标煤) Other Energy (10⁴tce)
**231.55**		**-194.50**	**943.98**	**934.30**	**28465.63**	**8617.18**	**558.00**
			948.48			8752.32	
						7221.72	
						738.80	
						446.22	
					28465.63		558.00
327.00		219.86	35.80	934.30		55.45	
-93.00		-9.56	-40.30			-190.59	
-2.45		-404.80					
**2044.57**	**1123.96**	**5031.55**	**-200.99**	**-130.52**	**269355.59**	**33319.28**	**-556.89**
	-74.01	-34.11	-161.78	-167.69	-24067.15	33319.28	-378.85
-0.88	-175.78	-92.48	-27.96	-8.78	293422.74		-174.28
2102.27	1460.71	7841.82					-3.76
-56.82	-86.96	-2683.68					
			-3.92				
			-7.33	45.95			
**2.40**			**17.11**	**14.14**	**3400.00**	**2568.24**	
**2180.20**	**1102.44**	**4500.68**	**723.57**	**781.00**	**294418.28**	**39366.25**	
4.66			0.50		91.01	976.49	
529.08	1102.44	4440.68	362.07	655.00	213189.00	28303.53	
57.72		1800.00	90.52	163.75			
7.18		60.00	1.16		661.74	483.24	
58.64			79.71	126.00	1637.94	734.53	
72.63			27.24		3902.19	1292.00	
51.00			26.00		7526.18	2451.83	
1457.01			226.90		67410.23	5124.63	
1097.60			226.22		67410.23	2988.06	
359.41			0.68			2136.57	
**93.52**	**21.52**	**336.37**	**2.31**	**8.64**	**2.94**	**1.97**	**1.11**
**2240.30**	**1439.19**	**7310.95**	**941.67**	**971.61**	**321885.43**	**41934.49**	**556.89**

?1994-2018 China Academic Journal Electronic Publishing House. All rights reserved. http://www.cnki.net

5-2 中国能源平衡表(标准量) -2010

单位: 万吨标准煤

项 目	Item	能源合计 Energy Total (发电煤耗计算法) (coal equivalent calculation)	能源合计 Energy Total (电热当量计算法) (calorific value calculation)
**一.可供本地区消费的能源量**	**Total Primary Energy Supply**	**332703.37**	**315747.30**
1.一次能源生产量	Indigenous Production	296915.72	279693.73
水电	Hydro Power	23085.71	8875.49
核电	Nuclear Power	2361.73	907.99
风电	Wind Power	1426.43	548.40
2.回收能	Recovery of Energy	5143.08	5143.08
3.进口量	Import	54863.59	54754.48
4.境内轮船和飞机在境外的加油量	Domestic Airplanes&Ships Refueling in Abroad	932.61	932.61
5.出口量(-)	Export (-)	-7802.37	-7427.34
6.境外轮船和飞机在境内的加油量(-)	Oversea Airplanes&Ships Refueling in China	-1042.79	-1042.79
7.库存增(-)、减(+)量	Stock Change	-16306.47	-16306.47
**二.加工转换投入(-)产出(+)量**	**Input(-) & Output(+) of Transformation**	**-11072.83**	**-76635.35**
1.火力发电	Thermal Power	0.00	-65562.52
2.供 热	Heating Supply	-3580.18	-3580.18
3.洗 选 煤	Coal Washing	-3530.81	-3530.81
4.炼 焦	Coking	-1479.64	-1479.64
5.炼油及煤制油	Petroleum Refineries	3149.76	3149.76
#油品再投入量(-)	Petroleum Products Input (-)	-5291.78	-5291.78
6.制 气	Gas Works	-135.77	-135.77
#焦炭再投入量(-)	Coke Input (-)	-137.53	-137.53
7.天然气液化	Natural Gas Liquefaction	-16.75	-16.75
8.煤制品加工	Briquettes	-50.13	-50.13
**三.损 失 量**	**Loss**	**8856.66**	**3803.12**
**四.终端消费量**	**Total Final Consumption**	**305009.67**	**227548.49**
1.农、林、牧、渔业	Farming, Forestry, Animal Husbandry, Fishery Conservancy	6477.30	4555.85
2.工业	Industry	211626.09	155933.09
#用作原料、材料	Non-Energy Use	9811.58	9811.58
3.建筑业	Construction	6226.30	5275.42
4.交通运输、仓储和邮政业	Transport, Storage and Post	25614.71	24169.37
5.批发、零售业和住宿、餐饮业	Wholesale, Retail Trade and Hotel, Restaurants	6826.82	4284.55
6.其他	Others	13680.50	8856.03
7.生活消费	Residential Consumption	34557.94	24474.18
城 镇	Urban	20706.84	14827.22
乡 村	Rural	13851.10	9646.96
**五.平衡差额**	**Statistical Difference**	**7764.22**	**7760.34**
**六.消费量合计**	**Total Final Consumption**	**324939.15**	**307986.96**

?1994-2018 China Academic Journal Electronic Publishing House. All rights reserved. http://www.cnki.net

ENERGY BALANCE OF CHINA -2010 (STANDARD QUANTITY)

(10 000 tce)

煤合计<br>Coal Total	原煤<br>Raw Coal	洗精煤<br>Cleaned Coal	其他洗煤<br>Other Washed Coal	型煤<br>Briquettes	煤矸石<br>Gangue	焦炭<br>Coke	焦炉煤气<br>Coke Oven Gas	高炉煤气<br>Blast Furnace Gas
**224756.84**	**226554.94**	**-844.70**	**-948.02**	**-5.39**		**-1894.95**		**2920.00**
227319.77	227319.77							
								2920.00
11649.15	11647.32			1.83		10.64		
-1526.85	-1522.38			-4.48		-325.42		
-12685.23	-10889.77	-844.70	-948.02	-2.75		-1580.17		
**-157863.96**	**-167129.16**	**3737.08**	**4867.80**	**660.33**	**-0.02**	**35290.56**	**3221.78**	**-1481.90**
-100049.33	-98348.34	-4.22	-1696.76		-445.31		-602.71	-1018.51
-11143.02	-10777.94	-9.48	-355.60		-94.19		-416.15	-463.38
-4070.29	-52947.87	41354.29	7523.29		539.48			
-41558.55	-4402.28	-37126.60	-29.67			35174.01	4104.82	
-168.64	-81.64	-83.91	-3.09					
-823.993238	-430.98	-393.012				241.1268903	135.82178	
						-124.57		
-50.13	-140.10		-570.37	660.33				
**61385.13**	**54840.04**	**2287.60**	**3626.74**	**630.75**		**32599.76**	**3150.07**	**1427.46**
1282.47	1261.60		20.88			45.48		
49318.47	43816.36	2276.43	2976.68	249.01		32498.63	2825.86	1427.46
3161.78	2916.77	117.01	128.00			1300.17	28.26	
523.15	516.45	1.22	5.48			5.64		
449.45	431.53	9.95	7.98			0.12		
1469.58	1429.08		20.55	19.95		4.95	4.57	
1467.00	1367.02		93.02	6.96		2.69	15.31	
6875.02	6018.01		502.17	354.84		42.24	304.33	
1355.50	1078.67		169.56	107.26		23.04	304.33	
5519.52	4939.34		332.61	247.57		19.21		
**5507.75**	**4585.74**	**604.78**	**293.04**	**24.19**	**-0.02**	**795.86**	**71.71**	**10.64**

?1994-2018 China Academic Journal Electronic Publishing House. All rights reserved. http://www.cnki.net

续表 1

单位：万吨标准煤

项　目	Item	转炉煤气 Converter Gas	其他煤气 Gasoline
**一.可供本地区消费的能源量**	**Total Primary Energy Supply**	**694.40**	
1.一次能源生产量	Indigenous Production		
水电	Hydro Power		
核电	Nuclear Power		
风电	Wind Power		
2.回收能	Recovery of Energy	694.40	
3.进口量	Import		
4.境内轮船和飞机在境外的加油量	Domestic Airplanes&Ships Refueling in Abroad		
5.出口量(−)	Export (-)		
6.境外轮船和飞机在境内的加油量(−)	Oversea Airplanes&Ships Refueling in China		
7.库存增(−)、减(+)量	Stock Change		
**二.加工转换投入(−)产出(+)量**	**Input(-) & Output(+) of Transformation**	**-212.91**	**311.66**
1.火力发电	Thermal Power	-125.25	
2.供　热	Heating Supply	-87.66	
3.洗 选 煤	Coal Washing		
4.炼　焦	Coking		
5.炼油及煤制油	Petroleum Refineries		
#油品再投入量(−)	Petroleum Products Input (-)		
6.制　气	Gas Works		311.66
#焦炭再投入量(−)	Coke Input (-)		
7.天然气液化	Natural Gas Liquefaction		
8.煤制品加工	Briquettes		
**三.损 失 量**	**Loss**		
**四.终端消费量**	**Total Final Consumption**	**485.86**	**301.14**
1.农、林、牧、渔业	Farming, Forestry, Animal Husbandry, Fishery Conservancy		
2.工业	Industry	485.86	98.23
#用作原料、材料	Non-Energy Use		
3.建筑业	Construction		
4.交通运输、仓储和邮政业	Transport, Storage and Post		
5.批发、零售业和住宿、餐饮业	Wholesale, Retail Trade and Hotel, Restaurants		
6.其他	Others		
7.生活消费	Residential Consumption		202.91
城 镇	Urban		202.13
乡 村	Rural		0.79
**五.平衡差额**	**Statistical Difference**	**-4.37**	**10.51**
**六.消费量合计**	**Total Final Consumption**		

?1994-2018 China Academic Journal Electronic Publishing House. All rights reserved. http://www.cnki.net

Continued 1

(10 000 tce)

其他焦化产品 Other Coking Products	油品合计 Petroleum Products Total	原油 Crude Oil	汽油 Gasoline	煤油 Kerosene	柴油 Diesel Oil	燃料油 Fuel Oil	石脑油 Naphtha	润滑油 Lubricants	石蜡 Petroleum Waxes
	**63233.54**	**61527.84**	**-656.49**	**-230.67**	**-324.23**	**1826.42**	**5.55**	**32.46**	**-69.74**
	29002.58	29002.58							
	40908.64	33955.22	0.01	716.57	262.28	3284.35	436.05	48.37	1.23
	932.61			351.75	14.85	566.01			
	-4804.85	-432.87	-760.71	-890.20	-676.09	-1414.31	-130.50	-15.91	-70.97
	-1042.79			-390.66	-38.23	-613.90			
	-2041.07	-1271.38	104.20	-18.14	112.97	4.27	-300.00		
**787.81**	**-3363.39**	**-59824.67**	**10830.06**	**2827.63**	**21574.91**	**1688.79**	**2897.36**	**309.65**	**218.52**
	-624.93	-5.30	-0.13		-165.90	-176.99			
	-819.91	-4.69			-5.51	-287.63			
800.08									
-51.06	3373.23	-59814.68	10830.20	2831.55	21746.31	3624.32	2910.98	309.65	218.52
	-5291.78			-3.91		-1470.90	-13.62		
51.76									
-12.96									
	**278.41**	**274.29**							
**776.64**	**58005.74**	**1151.62**	**10132.23**	**2562.31**	**21151.51**	**3433.18**	**2761.53**	**318.22**	**140.57**
	2018.04		248.77	1.32	1758.33	1.63			
776.64	20480.66	1151.62	1014.34	55.24	2981.46	1460.71	2761.53	318.22	140.57
	5321.37	153.17	32.56				435.00	309.25	137.95
	4114.71		404.19	12.90	714.27	43.94			
	21479.73		4715.73	2355.83	12412.39	1895.26			
	722.22		247.45	51.47	286.47	12.31			
	3755.28		1715.98	56.98	1875.56	19.33			
	5435.10		1785.77	28.56	1123.02				
	3962.49		1242.83	2.49	835.55				
	1472.62		542.94	26.07	287.47				
**11.17**	**1307.60**	**2.97**	**41.34**	**34.66**	**99.17**	**82.04**	**141.38**	**23.89**	**8.20**

?1994-2018 China Academic Journal Electronic Publishing House. All rights reserved. http://www.cnki.net

续表 2

单位：万吨标准煤

项　目	Item	溶剂油 White spirit	石油沥青 Bitumen Asphalt
**一.可供本地区消费的能源量**	**Total Primary Energy Supply**	**-0.25**	**514.44**
1.一次能源生产量	Indigenous Production		
水电	Hydro Power		
核电	Nuclear Power		
风电	Wind Power		
2.回收能	Recovery of Energy		
3.进口量	Import	4.93	534.09
4.境内轮船和飞机在境外的加油量	Domestic Airplanes&Ships Refueling in Abroad		
5.出口量(-)	Export (-)	-0.78	-19.65
6.境外轮船和飞机在境内的加油量(-)	Oversea Airplanes&Ships Refueling in China		
7.库存增(-)、减(+)量	Stock Change	-4.40	
**二.加工转换投入(-)产出(+)量**	**Input(-) & Output(+) of Transformation**	**109.06**	**2572.71**
1.火力发电	Thermal Power		
2.供　热	Heating Supply		
3.洗 选 煤	Coal Washing		
4.炼　焦	Coking		
5.炼油及煤制油	Petroleum Refineries	109.06	2572.71
#油品再投入量(-)	Petroleum Products Input (-)		
6.制　气	Gas Works		
#焦炭再投入量(-)	Coke Input (-)		
7.天然气液化	Natural Gas Liquefaction		
8.煤制品加工	Briquettes		
**三.损 失 量**	**Loss**		
**四.终端消费量**	**Total Final Consumption**	**102.70**	**2906.06**
1.农、林、牧、渔业	Farming, Forestry, Animal Husbandry, Fishery Conservancy		
2.工业	Industry	102.70	58.78
#用作原料、材料	Non-Energy Use	98.64	
3.建筑业	Construction		2847.29
4.交通运输、仓储和邮政业	Transport, Storage and Post		
5.批发、零售业和住宿、餐饮业	Wholesale, Retail Trade and Hotel, Restaurants		
6.其他	Others		
7.生活消费	Residential Consumption		
城 镇	Urban		
乡 村	Rural		
**五.平衡差额**	**Statistical Difference**	**6.10**	**181.08**
**六.消费量合计**	**Total Final Consumption**		

?1994-2018 China Academic Journal Electronic Publishing House. All rights reserved.　http://www.cnki.net

Continued 2

(10 000 tce)

石油焦<br>Petroleum Coke	液化石油气<br>LPG	炼厂干气<br>Refinery Gas	其他石油制品<br>Other Petroleum Products	天然气<br>Natural Gas	液化天然气<br>LNG	热力<br>Heat	电力<br>Electricity	其他能源<br>Other Energy
**465.84**	**401.06**		**-258.69**	**12554.93**	**1641.75**	**970.68**	**10590.51**	**558.00**
				12614.78			10756.60	
							8875.49	
							907.99	
							548.40	
						970.68		558.00
812.55	560.58		292.41	476.14	1641.75		68.15	
-220.71	-159.43		-12.71	-535.99			-234.24	
-126.00	-4.20		-538.38					
**1469.43**	**3505.01**	**1766.19**	**6691.96**	**-2673.17**	**-229.35**	**9185.03**	**40949.40**	**-556.89**
-114.94		-116.30	-45.37	-2151.67	-294.66	-820.69	40949.40	-378.85
-121.36	-1.51	-276.22	-123.00	-371.87	-15.43	10005.72		-174.28
1705.74	3603.92	2295.36	10429.62					-3.76
	-97.41	-136.65	-3569.29					
				-52.14				
				-97.49	80.74			
	**4.11**			**227.56**	**24.85**	**115.94**	**3156.37**	
**1890.00**	**3737.52**	**1732.37**	**5985.90**	**9623.53**	**1372.37**	**10039.66**	**48381.12**	
	7.99			6.65		3.10	1200.11	
1890.00	907.00	1732.37	5906.10	4815.53	1150.97	7269.74	34785.04	
1661.85	98.95		2394.00	1203.88	287.74			
	12.31		79.80	15.46		22.57	593.90	
	100.53			1060.08	221.41	55.85	902.74	
	124.52			362.30		133.06	1587.87	
	87.43			345.80		256.64	3013.30	
	2497.74			3017.71		2298.69	6298.17	
	1881.61			3008.72		2298.69	3672.33	
	616.13			8.99			2625.84	
**45.28**	**160.32**	**33.82**	**447.37**	**30.67**	**15.18**	**0.10**	**2.42**	**1.11**

?1994-2018 China Academic Journal Electronic Publishing House. All rights reserved. http://www.cnki.net

5-3 综合能源平衡表

单位：万吨标准煤

项目	Item	1980	1985
**可供消费的能源总量**	**Total Energy Available for Consumption**	**61557**	**77603**
一次能源生产量	Primary Energy Output	63735	85546
回收能	Recovery of Energy		
进口量	Imports	261	340
出口量(-)	Exports (-)	3058	5774
年初年末库存差额	Stock Changes in the Year	619	-2509
**能源消费总量**	**Total Energy Consumption**	**60275**	**76682**
在总量中:	Consumption by Sector		
1.农、林、牧、渔业	Farming, Forestry, Animal Husbandry, Fishery Conservancy	4692	4045
2.工业	Industry	38986	51068
3.建筑业	Construction	957	1302
4.交通运输、仓储和邮政业	Transport, Storage and Post	2902	3713
5.批发、零售业和住宿、餐饮业	Wholesale, Retail Trade and Hotel, Restaurants	518	766
6.其他	Others	1205	2470
7.生活消费	Residential Consumption	11015	13318
在总量中:	Consumption by Usage		
(一) 终端消费	(I)Final Consumption	57508	73586
#工业	Industry	38293	48021
(二) 加工转换损失量	(II)Losses in Processing and	1358	1491
#炼焦	Coking	644	572
炼油	Petroleum Refining	113	110
(三) 损失量	(III)Other Losses	1409	1605
**平衡差额**	**Balance**	**1282**	**921**

注：1.村办工业包括在工业中(下同)。
2.电力、热力按等价热值折算，因此加工转换损失量中不包括发电、供热损失量。
3.进口量包括境内轮船和飞机在境外的加油量；出口量包括境外轮船和飞机在境内的加油量。

?1994-2018 China Academic Journal Electronic Publishing House. All rights reserved. http://www.cnki.net

OVERALL ENERGY BALANCE SHEET

(10 000 tce)

1990	1995	2000	2005	2006	2007	2008	2009	2010
**96138**	**129535**	**142605**	**232225**	**256034**	**274800**	**287011**	**311277**	**332703**
103922	129034	135048	216219	232167	247279	260552	274619	296916
	2312	1760	2939	3725	6166	6511	7627	5143
1310	5456	14334	26952	31171	35062	36764	47313	55796
5875	6776	9633	11448	10925	9995	9955	8440	-8845
-3219	-491	1097	-2436	-104	-3711	-6860	-9841	-16306
**98703**	**131176**	**145531**	**235997**	**258676**	**280508**	**291448**	**306647**	**324939**
4852	5505	3914	6071	6331	6228	6013	6251	6477
67578	96191	103774	168724	184945	200531	209302	219197	231102
1213	1335	2179	3403	3761	4128	3813	4562	6226
4541	5863	11242	18391	20284	21959	22917	23692	26068
1247	2018	3048	4848	5314	5689	5734	6412	6827
3473	4519	5762	9255	10276	11158	11771	12690	13681
15799	15745	15614	25305	27765	30814	31898	33843	34558
94289	124252	139008	225690	247520	268610	278546	292299	305010
63239	89473	97597	158767	174225	189032	196832	205322	211626
2264	3634	2461	3823	4056	4241	5166	6283	11073
905		525	702	734	854	819	1010	1480
326		781	1305	1391	1325	1380	1784	2142
2150	3289	4062	6483	7100	7657	7736	8065	8857
**-2565**	**-1641**	**-2926**	**-3772**	**-2642**	**-5708**	**-4437**	**4630**	**7764**

a) Data on industry include the data of village-run industry.(The sane as in the following tables).

b) Electric power and heat are converted on the basic of equal caloric value. Therefore, losses in processing and transformation exclude losses in powei generation and heating.

c) Data on imports include the petroleum consumed by the domestic airplanes and ships in refueling abroad. Data on exports include the petroleum consumed by the oversea airplanes and ships in refueling in China.

?1994-2018 China Academic Journal Electronic Publishing House. All rights reserved. http://www.cnki.net

5-4 煤炭平衡表

单位: 万吨

项　　目	Item	1980	1985
**可供量**	**Total Energy Available for Consumption**	**62601.0**	**82776.6**
生产量	Output	62015.0	87228.4
进口量	Imports	199.0	230.7
出口量(-)	Exports (-)	632.0	777.0
年初年末库存差额	Stock Changes in the Year	1019.0	-3905.5
**消费量**	**Total Energy Consumption**	**61009.5**	**81603.0**
在消费量中:	Consumption by Sector		
1.农、林、牧、渔业	Farming, Forestry, Animal Husbandry, Fishery Conservancy	1550.3	2208.6
2.工业	Industry	43848.4	58613.3
3.建筑业	Construction	556.0	531.9
4.交通运输、仓储和邮政业	Transport, Storage and Post	1934.4	2307.1
5.批发、零售业和住宿、餐饮业	Wholesale, Retail Trade and Hotel, Restaurants	455.2	738.2
6.其他	Others	1091.2	1579.5
7.生活消费	Residential Consumption	11574.0	15624.4
在消费量中:	Consumption by Usage		
(一) 终端消费	(I)Final Consumption	38804.2	52704.4
#工　业	Industry	21643.1	29715.0
(二) 中间消费	(2)Intermediate Consumption		
(用于加工转换)	(Consumed in Transformation)	19461.6	25397.4
发　电	Power Generation	12648.4	16440.7
供　热	Heating		1462.3
炼　焦	Coking	6682.2	7303.8
炼油及煤制油			
制　气	Gas Production	131.0	190.6
(三) 洗选损耗	(3) Losses in Coal Washing and Dressing	2743.7	3501.2
**平衡差额**	**Balance**	**1591.5**	**1173.6**

注: 生产量为原煤产量。

?1994-2018 China Academic Journal Electronic Publishing House. All rights reserved.　　http://www.cnki.net

COAL BALANCE SHEET

(10 000 ton)

1990	1995	2000	2005	2006	2007	2008	2009	2010
**102221.0**	**133461.7**	**136794.5**	**226941.0**	**251336.3**	**265543.5**	**275061.1**	**301283.8**	**319772.0**
107988.3	136073.1	138418.5	234951.8	252855.1	269164.3	280200.0	297300.0	323500.0
200.3	163.5	217.9	2617.1	3810.5	5101.6	4034.1	12584.0	16309.5
1729.0	2861.7	5506.5	7172.4	6327.3	5318.7	4543.4	2239.6	1910.4
-4238.5	86.8	3664.7	-3455.4	997.9	-3403.6	-4629.6	-6360.6	-18127.2
**105523.0**	**137676.5**	**141091.7**	**231851.1**	**255065.5**	**272745.9**	**281095.9**	**295833.1**	**312236.5**
2095.2	1856.7	933.4	1513.8	1502.6	1519.6	1522.6	1582.1	1711.1
81090.9	117570.7	127806.7	215493.3	238510.2	256202.8	265574.2	279888.5	296031.6
437.6	439.8	536.8	603.6	652.0	615.3	603.2	635.6	718.9
2160.9	1315.1	882.2	811.2	769.9	735.9	665.4	640.9	639.2
1058.3	977.4	1314.6	1674.4	1791.5	1868.3	1791.4	1977.9	1969.9
1980.4	1986.7	1161.0	1715.9	1802.9	2043.4	1791.6	1986.1	2006.6
16699.7	13530.1	8457.0	10039.0	10036.3	9760.6	9147.6	9121.9	9159.2
60205.9	66156.1	55913.1	75382.7	77532.6	79676.6	81089.2	83700.5	84350.9
35773.8	46050.3	42628.0	59024.9	60977.4	63133.5	65567.5	67755.9	68146.1
41257.8	69487.6	85178.6	156468.4	177532.8	193069.3	200006.7	212132.6	227885.6
27204.3	44440.2	55811.2	103263.5	118763.9	130548.8	135351.7	143967.3	154542.5
2995.5	5887.3	8794.1	13542.0	14561.4	15394.2	15029.2	15359.7	15253.1
10697.6	18396.4	16496.4	33167.1	37450.1	39659.0	41461.7	43691.7	47150.4
								213.4
360.4	763.7	960.0	1277.0	1257.1	1491.8	1227.2	1150.7	1040.1
4059.3	2032.8	3191.2	4982.1	5279.3	5754.6	6757.8	7765.5	9484.6
**-3302.0**	**-4214.8**	**-4297.2**	**-4910.0**	**-3729.2**	**-7202.4**	**-6034.9**	**5450.7**	**7535.5**

a) Data on output refer to the output of raw coal.

?1994-2018 China Academic Journal Electronic Publishing House. All rights reserved. http://www.cnki.net

5-5 焦炭平衡表

单位: 万吨

项目	Item	1980	1985
**可供量**	**Total Energy Available for Consumption**	**4315.3**	**4689.7**
生产量	Output	4343.0	4802.1
进口量	Imports		2.1
出口量(-)	Exports (-)	27.1	36.9
年初年末库存差额	Stock Changes in the Year	-0.6	-77.6
**消费量**	**Total Energy Consumption**	**4303.0**	**4689.7**
在消费量中:	Consumption by Sector		
1.农、林、牧、渔业	Farming, Forestry, Animal Husbandry, Fishery Conservancy	10.6	20.8
2.工业	Industry	4266.7	4627.7
3.建筑业	Construction	11.9	7.8
4.交通运输、仓储和邮政业	Transport, Storage and Post	8.2	5.7
5.批发、零售业和住宿、餐饮业	Wholesale, Retail Trade and Hotel, Restaurants	0.9	2.7
6.其他	Others	4.7	2.0
7.生活消费	Residential Consumption		23.0
在消费量中:	Consumption by Usage		
(一) 终端消费	(1)Final Consumption	4294.7	4677.9
#工　业	Industry	4258.4	4615.9
(二) 中间消费	(2)Intermediate Consumption		
(用于加工转换)	(Consumed in Transformation)	8.3	11.8
制　气	Gas Production	8.3	11.8
(三) 损失量	(3) Losses in Coal Washing and Dressing		
**平衡差额**	**Balance**	**12.3**	

?1994-2018 China Academic Journal Electronic Publishing House. All rights reserved.   http://www.cnki.net

COKE BALANCE SHEET

(10 000 ton)

1990	1995	2000	2005	2006	2007	2008	2009	2010
**7085.8**	**12207.1**	**10892.3**	**25184.4**	**27990.5**	**29090.1**	**29994.0**	**31961.3**	**34507.1**
7328.3	13424.5	12184.0	26611.7	29768.3	31305.3	32313.9	34244.1	36457.8
	0.1		0.5				15.9	11.0
129.0	886.1	1519.7	1276.4	1446.8	1529.9	1221.3	54.5	335.0
-113.5	-331.4	228.0	-151.4	-331.1	-685.3	-1098.6	-2244.1	-1626.7
**6914.7**	**10725.3**	**10840.8**	**25105.8**	**27892.8**	**29168.1**	**29900.2**	**31850.0**	**33687.8**
60.1	128.6	70.9	63.5	55.7	57.2	53.1	44.6	46.8
6808.8	10412.0	10554.6	24860.9	27653.6	28932.2	29756.7	31743.3	33583.7
5.2	10.8	19.0	18.4	18.5	17.5	10.7	5.7	5.8
4.1	10.1	11.2	1.1	0.9	0.6	0.3	0.1	0.1
7.7	25.7	35.7	64.1	65.4	71.0	7.5	3.9	5.1
1.9	6.4	12.2	7.6	8.3	8.2	6.9	3.5	2.8
26.9	131.6	137.2	90.3	90.4	81.4	64.9	48.8	43.5
6846.3	10648.0	10697.9	24877.9	27664.9	28900.1	29712.6	31689.7	33559.6
6740.4	10334.7	10411.7	24633.0	27425.6	28664.2	29569.1	31583.0	33455.5
68.4	77.3	142.9	227.9	227.9	268.0	187.6	160.3	128.2
68.4	77.3	142.9	227.9	227.9	268.0	187.6	160.3	128.2
**171.1**	**1481.8**	**51.6**	**78.6**	**97.7**	**-78.1**	**93.8**	**111.4**	**819.3**

?1994-2018 China Academic Journal Electronic Publishing House. All rights reserved. http://www.cnki.net

5-6 石油平衡表

单位: 万吨

项目	Item	1980	1985
**可供量**	**Total Energy Available for Consumption**	**8794.5**	**9193.7**
生产量	Output	10594.6	12489.5
进口量	Imports	82.7	90.0
出口量(-)	Exports (-)	1806.2	3630.4
年初年末库存差额	Stock Changes in the Year	-76.6	244.6
**消费量**	**Total Energy Consumption**	**8757.4**	**9168.8**
在消费量中:	Consumption by Sector		
1.农、林、牧、渔业	Farming, Forestry, Animal Husbandry, Fishery Conservancy	814..9	758.7
2.工业	Industry	6203.2	6171.4
3.建筑业	Construction	175.2	292.2
4.交通运输、仓储和邮政业	Transport, Storage and Post	911.5	1176.4
5.批发、零售业和住宿、餐饮业	Wholesale, Retail Trade and Hotel, Restaurants	29.0	38.1
6.其他	Others	481.7	506.1
7.生活消费	Residential Consumption	141.9	225.9
在消费量中:	Consumption by Usage	8838.9	
(一) 终端消费	(I)Final Consumption	6311.0	7063.3
#工 业	Industry	3780.3	4462.0
(二) 中间消费	(II)Intermediate Consumption		
(用于加工转换)	(Consumed in Transformation)	2183.6	1858.5
发 电	Power Generation	2065.4	1425.5
供 热	Heating		285.6
制 气	Gas Production	36.7	34.5
炼油损失量	Losses in Petroleum Refining	81.5	112.9
(三)损失量	(III) Other Losses	262.8	247.0
**平衡差额**	**Balance**	**37.1**	**24.9**

注: 1.生产量为原油产量。
2.进口量包括境内轮船和飞机在境外的加油量；出口量包括境外轮船和飞机在境内的加油量。

?1994-2018 China Academic Journal Electronic Publishing House. All rights reserved. http://www.cnki.net

PETROLEUM BALANCE

(10 000 ton)

1990	1995	2000	2005	2006	2007	2008	2009	2010
**11435.0**	**16072.7**	**22631.8**	**32539.1**	**34930.0**	**36648.9**	**37318.8**	**38462.8**	**44178.4**
13830.6	15005.0	16300.0	18135.3	18476.6	18631.8	19044.0	18949.0	20301.4
755.6	3673.2	9748.5	17163.2	19453.0	21139.4	23015.5	25642.4	29437.2
3110.4	2454.5	2172.1	2888.1	2626.2	2664.3	2945.7	3916.6	4079.0
-40.8	-151.0	-1244.6	128.8	-373.3	-458.0	-1795.0	-2211.9	-1481.2
**11485.6**	**16064.9**	**22495.9**	**32537.7**	**34876.2**	**36658.7**	**37302.9**	**38384.5**	**43245.2**
1033.6	1203.2	788.5	1451.7	1540.2	1399.9	1265.8	1308.1	1382.5
7321.6	9349.3	11248.5	14245.1	14804.3	14905.1	15603.1	15692.9	17448.8
327.3	242.8	840.6	1502.2	1648.5	1823.1	1517.5	1942.3	3045.1
1683.2	2863.6	6399.0	10709.5	11849.2	12906.7	13279.4	13548.5	14870.3
77.6	333.9	247.0	375.6	392.2	426.9	366.4	429.7	481.0
757.8	1390.3	1635.9	1969.2	2077.6	2215.9	2353.8	2296.3	2556.7
284.5	682.0	1336.5	2284.4	2564.2	2981.1	2916.9	3166.8	3460.8
9304.7	13676.3	19950.1	29191.6	31614.1	33857.6	34702.9	35689.9	40393.7
5180.4	7095.5	8860.0	11027.5	11707.5	12269.2	13170.8	13153.7	14757.8
1630.4	2230.0	2352.9	3190.7	3062.1	2601.2	2397.6	2505.9	2657.1
1234.4	1358.5	1178.2	1602.0	1343.7	884.0	618.1	501.2	459.1
356.3	399.9	427.0	407.6	427.8	430.1	413.8	383.3	593.1
39.7	51.6	25.9	14.4	13.4	5.0	2.0	0.3	
295.8	420.1	721.9	1166.7	1277.3	1282.1	1363.7	1621.1	1604.8
254.7	158.6	192.9	155.4	200.0	200.0	202.4	188.7	194.4
**-50.6**	**7.8**	**135.8**	**1.4**	**53.8**	**-9.9**	**15.9**	**78.3**	**933.3**

a) Data on output refer to the output of crude oil.

b) Data on imports include the petroleum consumed by the domestic airplanes and ships in refueling abroad. Data on exports include the petroleum consumed by the oversea airplanes and ships in refueling in China.

?1994-2018 China Academic Journal Electronic Publishing House. All rights reserved. http://www.cnki.net

5-7 原油平衡表

单位：万吨

项　　目	Item	1980	1985
**可供量**	**Total Energy Available for Consumption**	**9222.9**	**9516.5**
生产量	Output	10594.6	12489.5
进口量	Imports	36.6	
出口量(-)	Exports (-)	1330.9	3003.0
年初年末库存差额	Stock Changes in the Year	-77.4	30.0
**消费量**	**Total Energy Consumption**	**9205.0**	**9509.5**
在消费量中:	Consumption by Sector		
1.农、林、牧、渔业	Farming, Forestry, Animal Husbandry, Fishery Conservancy	8.0	0.8
2.工业	Industry	9112.0	9389.9
3.建筑业	Construction	28.8	74.0
4.交通运输、仓储和邮政业	Transport, Storage and Post	50.1	44.3
5.批发、零售业和住宿、餐饮业	Wholesale, Retail Trade and Hotel, Restaurants		0.1
6.其他	Others	6.1	0.4
7.生活消费	Residential Consumption		
在消费量中:	Consumption by Usage		
(一) 终端消费	(I)Final Consumption	499.6	350.4
#工　业	Industry	429.7	254.9
(二) 中间消费	(II)Intermediate Consumption		
(用于加工转换)	(Consumed in Transformation)	8443.0	8929.7
发　电	Power Generation	574.0	279.5
供　热	Heating		61.3
炼　　油	Petroleum Refineries	7869.0	8588.9
(三) 油田原油损失量	(III)Losses in Oil Field for Crude Oil	262.4	229.4
**平衡差额**	**Balance**	**17.9**	**7.0**

?1994-2018 China Academic Journal Electronic Publishing House. All rights reserved. http://www.cnki.net

CRUDE OIL BALANC SHEET

(10 000 ton)

1990	1995	2000	2005	2006	2007	2008	2009	2010
**11770.6**	**14794.9**	**21383.0**	**30089.2**	**32249.2**	**34035.1**	**35498.6**	**38130.5**	**42876.6**
13830.6	15004.4	16300.0	18135.3	18476.6	18631.8	19044.0	18949.0	20301.4
292.3	1709.0	7026.5	12681.7	14517.5	16316.0	17888.5	20365.3	23768.2
2399.0	1822.7	1030.6	806.7	633.7	388.4	423.8	507.3	303.0
46.7	-95.8	-912.9	78.8	-111.1	-524.4	-1010.1	-676.5	-890.0
**11762.2**	**14886.4**	**21232.0**	**30086.2**	**32245.2**	**34031.6**	**35498.2**	**38128.6**	**42874.6**
0.2	10.1							
11653.8	14716.3	21052.1	29959.4	32081.5	33867.9	35332.6	37975.2	42716.6
55.2	2.7	3.3						
52.1	156.8	175.1	126.9	163.7	163.7	165.7	153.4	158.0
0.3	0.5	0.2						
0.6	1390.3	1.4						
402.1	309.9	636.8	870.4	977.1	984.1	1192.8	826.2	806.1
333.4	274.7	612.3	870.4	977.1	984.1	1192.8	826.2	806.1
11106.9	14419.4	20404.3	29062.1	31069.8	32849.1	34105.1	37115.8	41876.4
124.6	61.6	85.0	21.3	22.9	15.9	8.9	4.4	3.7
21.1	4.4	14.0	0.3	0.8	0.6	1.0	2.0	3.3
10961.2	14353.4	20305.3	29040.5	31046.0	32832.6	34095.3	37109.4	41869.4
253.2	157.1	190.9	153.8	198.4	198.4	200.4	186.7	192.0
**8.4**	**-91.5**	**151.0**	**2.9**	**4.0**	**3.5**	**0.4**	**1.9**	**2.1**

?1994-2018 China Academic Journal Electronic Publishing House. All rights reserved. http://www.cnki.net

5-8 燃料油平衡表

单位: 万吨

项目	Item	1980	1985
**可供量**	**Total Energy Available for Consumption**	**3096.1**	**2848.0**
生产量	Output	3142.0	2835.8
进口量	Imports	39.0	70.0
出口量(-)	Exports (-)	45.4	64.9
年初年末库存差额	Stock Changes in the Year	-39.5	7.1
**消费量**	**Total Energy Consumption**	**3073.7**	**2837.4**
在消费量中:	Consumption by Sector		
1.农、林、牧、渔业	Farming, Forestry, Animal Husbandry, Fishery Conservancy	2.3	3.1
2.工业	Industry	2937.4	2662.2
3.建筑业	Construction	15.0	18.9
4.交通运输、仓储和邮政业	Transport, Storage and Post	109.0	144.1
5.批发、零售业和住宿、餐饮业	Wholesale, Retail Trade and Hotel, Restaurants	2.9	3.1
6.其他	Others	7.1	6.0
7.生活消费	Residential Consumption		
在消费量中:	Consumption by Usage		
(一) 终端消费	(I)Final Consumption	1617.9	1538.8
#工 业	Industry	1481.6	1363.5
(二) 中间消费	(II)Intermediate Consumption		
(用于加工转换)	(Consumed in Transformation)	1455.8	1296.1
发 电	Power Generation	1419.1	1042.3
供 热	Heating		219.3
炼油再投入量	Petroleum Production		
制 气	Gas Production	36.7	34.5
(三)损失量	(III)Other Losses		2.5
**平衡差额**	**Balance**	**22.4**	**10.6**

?1994-2018 China Academic Journal Electronic Publishing House. All rights reserved. http://www.cnki.net

FUEL OIL BALANCE SHEET

(10 000 ton)

1990	1995	2000	2005	2006	2007	2008	2009	2010
**3320.7**	**3717.3**	**3836.7**	**4237.3**	**4371.8**	**4158.4**	**3242.4**	**2829.3**	**3815.4**
3267.9	2960.8	2053.7	1767.4	1784.7	1967.2	1737.4	1353.4	2537.0
167.3	859.1	1704.3	2883.9	3138.1	2807.6	2533.2	2723.9	2695.2
97.2	68.6	57.9	427.6	466.9	631.4	1036.4	1222.4	1419.7
-17.3	-34.0	136.6	13.5	-84.1	14.9	8.3	-25.5	3.0
**3367.8**	**3693.7**	**3872.8**	**4242.1**	**4368.3**	**4157.5**	**3237.1**	**2827.8**	**3758.0**
2.9	8.4	0.4	0.7	0.7	1.0	1.5	1.1	1.1
3091.7	3406.2	2975.1	2924.8	2836.1	2329.2	2039.5	1521.5	2377.3
47.3	14.2	16.7	14.2	16.3	30.7	37.7	34.2	30.8
208.2	227.5	850.0	1261.0	1480.6	1760.0	1142.8	1250.6	1326.7
1.6	6.6	11.6	27.5	21.4	24.8	6.3	8.1	8.6
16.1	30.8	19.0	13.9	13.1	11.8	9.5	12.3	13.5
2042.6	2262.8	2741.4	2949.9	3267.7	3455.5	2757.8	2533.7	2403.2
1766.5	1975.3	1843.7	1632.6	1735.5	1627.2	1560.1	1227.5	1022.5
1325.2	1430.9	1131.3	1292.2	1100.6	702.0	479.3	294.1	1354.8
977.3	1071.5	814.2	1106.1	928.0	565.6	347.5	193.0	123.9
308.3	307.8	291.2	171.7	159.2	131.5	129.8	100.7	201.3
								1029.6
39.6	51.6	25.9	14.4	13.4	5.0	2.0	0.3	
**-47.1**	**23.6**	**-36.1**	**-4.8**	**3.5**	**0.9**	**5.3**	**1.5**	**57.4**

?1994-2018 China Academic Journal Electronic Publishing House. All rights reserved. http://www.cnki.net

5-9 汽油平衡表

单位：万吨

项目	Item	1980	1985
**可供量**	**Total Energy Available for Consumption**	**999.4**	**1399.6**
生产量	Output	1079.0	1471.9
进口量	Imports		0.3
出口量(-)	Exports (-)	117.8	129.9
年初年末库存差额	Stock Changes in the Year	38.2	57.3
**消费量**	**Total Energy Consumption**	**998.6**	**1396.3**
在消费量中:	Consumption by Sector		
1.农、林、牧、渔业	Farming, Forestry, Animal Husbandry, Fishery Conservancy	53.3	122.3
2.工业	Industry	273.2	451.3
3.建筑业	Construction	54.1	73.0
4.交通运输、仓储和邮政业	Transport, Storage and Post	404.9	477.4
5.批发、零售业和住宿、餐饮业	Wholesale, Retail Trade and Hotel, Restaurants	19.4	23.4
6.其他	Others	193.7	238.3
7.生活消费	Residential Consumption		10.6
**平衡差额**	**Balance**	**0.8**	**3.3**

5-10 煤油平衡表

单位：万吨

项目	Item	1980	1985
**可供量**	**Total Energy Available for Consumption**	**359.0**	**383.2**
生产量	Output	398.5	405.3
进口量	Imports		15.2
出口量(-)	Exports (-)	46.8	46.0
年初年末库存差额	Stock Changes in the Year	2.3	8.7
**消费量**	**Total Energy Consumption**	**365.9**	**385.5**
在消费量中:	Consumption by Sector		
1.农、林、牧、渔业	Farming, Forestry, Animal Husbandry, Fishery Conservancy	2.3	3.3
2.工业	Industry	15.7	20.1
3.建筑业	Construction	0.8	1.3
4.交通运输、仓储和邮政业	Transport, Storage and Post	31.4	56.2
5.批发、零售业和住宿、餐饮业	Wholesale, Retail Trade and Hotel, Restaurants	0.2	0.1
6.其他	Others	216.7	182.9
7.生活消费	Residential Consumption	98.8	121.6
**平衡差额**	**Balance**	**-6.9**	**-2.3**

?1994-2018 China Academic Journal Electronic Publishing House. All rights reserved. http://www.cnki.net

GASOLINE BALANCE SHEET

(10 000 ton)

1990	1995	2000	2005	2006	2007	2008	2009	2010
**1884.1**	**2902.0**	**3504.5**	**4854.7**	**5243.0**	**5519.3**	**6147.6**	**6181.8**	**6914.3**
2173.4	3051.6	4134.7	5433.0	5595.0	5917.9	6347.2	7320.7	7360.5
16.9	15.9			6.1	22.7	198.7	4.4	
233.8	193.1	467.7	559.7	350.5	464.3	203.4	491.9	517.0
-72.4	27.6	-162.5	-18.6	-7.5	42.9	-194.9	-651.4	70.8
**1899.5**	**2909.6**	**3504.6**	**4854.9**	**5242.5**	**5519.1**	**6145.5**	**6172.7**	**6886.2**
145.9	179.7	89.2	159.6	167.7	172.8	160.4	168.1	169.1
589.3	812.4	682.0	441.7	498.5	524.5	586.1	671.1	689.5
89.5	103.6	115.6	172.1	180.7	178.8	196.2	235.4	274.7
620.1	982.3	1527.8	2430.1	2592.4	2613.2	3090.4	2881.6	3204.9
46.0	197.2	69.8	129.4	123.3	131.7	135.3	147.5	168.2
390.7	570.7	792.7	998.2	1064.1	1119.7	1121.9	1069.9	1166.2
18.0	63.7	227.6	523.8	615.7	778.4	855.1	999.1	1213.7
**-15.4**	**-7.6**	**-0.1**	**-0.2**	**0.5**	**0.2**	**2.1**	**9.1**	**28.1**

KEROSENE BALANCE SHEET

(10 000 ton)

1990	1995	2000	2005	2006	2007	2008	2009	2010
**350.9**	**486.4**	**880.9**	**1070.0**	**1117.9**	**1239.2**	**1290.4**	**1448.2**	**1767.6**
392.5	445.8	872.3	1006.5	975.5	1153.3	1158.9	1480.3	1924.4
26.1	115.7	322.5	476.1	731.7	725.0	836.8	795.1	726.1
55.5	62.4	256.3	447.6	584.4	637.1	706.5	826.1	870.5
-12.2	-12.7	-57.6	35.0	-4.9	-2.0	1.3	-1.1	-12.3
**350.9**	**512.1**	**871.6**	**1076.8**	**1124.7**	**1243.7**	**1294.0**	**1439.4**	**1744.1**
3.1	3.6	1.5	1.6	1.5	0.9	1.3	0.8	0.9
20.6	44.9	84.0	57.5	48.2	45.2	49.1	32.0	40.2
1.3	3.5	4.0				9.7	10.4	8.8
93.4	250.0	535.9	952.4	1010.5	1130.0	1174.6	1314.3	1601.1
0.6	8.5	14.0	3.7	3.8	4.9	20.8	29.1	35.0
127.3	137.3	160.1	36.2	38.0	43.2	25.9	33.7	38.7
104.6	64.3	72.2	25.5	22.7	19.5	12.7	19.2	19.4
	**-25.7**	**9.3**	**-6.8**	**-6.9**	**-4.5**	**-3.6**	**8.8**	**23.6**

?1994-2018 China Academic Journal Electronic Publishing House. All rights reserved. http://www.cnki.net

5-11 柴油平衡表

单位: 万吨

项　目	Item	1980	1985
**可供量**	**Total Energy Available for Consumption**	**1663.2**	**1944.1**
生产量	Output	1827.8	2023.2
进口量	Imports	2.1	4.5
出口量(-)	Exports (-)	166.5	225.6
年初年末库存差额	Stock Changes in the Year	-0.2	142.0
**消费量**	**Total Energy Consumption**	**1663.2**	**1939.4**
在消费量中:	Consumption by Sector		
1.农、林、牧、渔业	Farming, Forestry, Animal Husbandry, Fishery Conservancy	749.0	629.2
2.工业	Industry	457.4	644.1
3.建筑业	Construction	76.5	125.0
4.交通运输、仓储和邮政业	Transport, Storage and Post	316.1	454.4
5.批发、零售业和住宿、餐饮业	Wholesale, Retail Trade and Hotel, Restaurants	6.5	10.9
6.其他	Others	57.7	74.0
7.生活消费	Residential Consumption		
在消费量中:	Consumption by Usage		
(一) 终端消费	(I)Final Consumption	1590.9	1827.4
#工　业	Industry	385.1	532.1
(二) 中间消费	(II)Intermediate Consumption		
(用于加工转换)	(Consumed in Transformation)	72.3	108.6
发　电	Power Generation	72.3	103.6
供　热	Heating		5.0
(三) 损失量	(III)Other Losses		3.4
**平衡差额**	**Balance**		**4.7**

5-12 液化石油气平衡表

单位: 万吨

项　目	Item	1980	1985
**可供量**	**Total Energy Available for Consumption**	**122.5**	**157.3**
生产量	Output	122.5	159.7
进口量	Imports		
出口量(-)	Exports (-)		1.9
年初年末库存差额	Stock Changes in the Year		-0.5
**消费量**	**Total Energy Consumption**	**119.6**	**155.7**
在消费量中:	Consumption by Sector		
1.农、林、牧、渔业	Farming, Forestry, Animal Husbandry, Fishery Conservancy		
2.工业	Industry	76.1	59.9
3.建筑业	Construction		
4.交通运输、仓储和邮政业	Transport, Storage and Post		
5.批发、零售业和住宿、餐饮业	Wholesale, Retail Trade and Hotel, Restaurants		0.5
6.其他	Others	0.4	4.5
7.生活消费	Residential Consumption	43.1	90.8
**平衡差额**	**Balance**	**2.9**	**1.6**

?1994-2018 China Academic Journal Electronic Publishing House. All rights reserved. http://www.cnki.net

DIESEL OIL BALANCE SHEET

(10 000 ton)

1990	1995	2000	2005	2006	2007	2008	2009	2010
**2689.4**	**4404.2**	**6806.5**	**10972.6**	**11836.1**	**12494.2**	**13543.1**	**13768.1**	**14701.9**
2609.0	3972.6	7079.6	11090.2	11762.4	12359.1	13409.2	14288.6	14924.4
233.8	645.3	51.9	61.0	80.7	173.7	633.1	192.5	190.2
169.8	169.5	77.5	170.9	102.6	93.3	89.1	478.7	490.2
16.4	-44.2	-247.6	-7.7	95.6	54.7	-410.0	-234.3	77.5
**2691.7**	**4321.4**	**6806.2**	**10972.2**	**11835.4**	**12496.7**	**13532.6**	**13756.6**	**14633.8**
881.5	1001.4	697.1	1286.3	1365.5	1219.0	1098.9	1134.1	1206.7
728.1	1189.9	1696.5	1991.3	1961.6	1972.8	2517.0	2348.8	2163.8
133.0	118.2	205.9	386.6	428.7	433.8	370.8	415.3	490.2
709.4	1246.6	3293.8	5890.4	6547.3	7184.4	7649.3	7892.0	8518.6
22.5	103.6	95.9	116.0	129.8	133.9	152.7	181.7	196.6
217.0	645.7	638.7	895.1	933.0	1007.5	1151.8	1131.8	1287.2
	16.1	178.4	406.4	469.6	545.3	592.1	652.9	770.7
2564.8	4070.0	6578.6	10605.4	11522.7	12265.3	13347.5	13604.8	14516.2
601.2	938.5	1468.8	1624.5	1648.9	1741.4	2331.9	2197.0	2046.2
126.9	251.4	227.7	366.7	312.7	231.4	185.1	151.8	117.6
124.5	204.9	227.7	366.7	312.7	231.4	185.1	151.8	113.9
2.4	46.6							3.8
**-2.3**	**82.7**	**0.3**	**0.4**	**0.7**	**-2.4**	**10.6**	**11.4**	**68.1**

LPG BALANCE SHEET

(10 000 ton)

1990	1995	2000	2005	2006	2007	2008	2009	2010
**258.5**	**774.3**	**1396.2**	**2052.2**	**2252.5**	**2320.2**	**2114.3**	**2157.7**	**2333.8**
261.6	540.8	916.6	1432.7	1745.3	1944.7	1914.8	1831.7	2102.3
	232.6	481.7	617.0	535.6	405.4	259.2	408.0	327.0
1.1	7.1	1.6	2.7	15.1	33.8	67.9	84.9	93.0
-2.0	8.0	-0.6	5.2	-13.3	3.8	8.1	2.9	-2.5
**254.2**	**750.6**	**1389.7**	**2046.5**	**2207.6**	**2327.9**	**2118.9**	**2153.1**	**2240.3**
	0.1	0.4	3.5	4.7	6.2	3.7	4.1	4.7
82.0	192.5	426.1	534.4	541.2	455.6	499.4	478.5	586.8
1.0	0.5	8.9	6.3	7.5	7.2	6.2	6.5	7.2
	0.5	16.5	48.7	54.7	55.5	56.7	56.6	61.0
6.6	17.4	55.5	99.0	113.9	131.6	51.4	63.2	72.6
6.1	5.7	24.0	25.8	29.4	33.8	44.7	48.6	51.0
158.5	534.0	858.3	1328.7	1456.2	1637.9	1457.0	1495.7	1457.0
**4.3**	**23.7**	**6.5**	**5.7**	**44.9**	**-7.6**	**-4.7**	**4.6**	**93.5**

?1994-2018 China Academic Journal Electronic Publishing House. All rights reserved. http://www.cnki.net

5-13 天然气平衡表

单位: 亿立方米

项　目	Item	1980	1985
**可供量**	**Total Energy Available for Consumption**	**142.7**	**129.3**
生产量	Output	142.7	129.3
进口量	Imports		
出口量(-)	Exports (-)		
年初年末库存差额	Stock Changes in the Year		
**消费量**	**Total Energy Consumption**	**140.6**	**129.3**
在消费量中:	Consumption by Sector		
1.农、林、牧、渔业	Farming, Forestry, Animal Husbandry, Fishery Conservancy		
2.工业	Industry	131.4	109.6
3.建筑业	Construction	6.0	14.1
4.交通运输、仓储和邮政业	Transport, Storage and Post	0.7	0.8
5.批发、零售业和住宿、餐饮业	Wholesale, Retail Trade and Hotel, Restaurants		
6.其他	Others	0.5	0.5
7.生活消费	Residential Consumption	2.0	4.3
**平衡差额**	**Balance**	**2.1**	

注: 从2010年起包括液化天然气数据。

5-14 电力平衡表

单位: 亿千瓦小时

项　目	Item	1980	1985
**可供量**	**Total Energy Available for Consumption**	**3006.3**	**4117.6**
生产量	Output	3006.3	4106.9
水　电	Hydropower	582.1	923.7
火　电	Thermal Power	2424.2	3183.2
核　电	Nuclear Power		
风　电	Wind Power		
进口量	Imports		11.1
出口量(-)	Exports (-)		0.4
**消费量**	**Total Energy Consumption**	**3006.3**	**4117.6**
在消费量中:	Consumption by Sector		
1.农、林、牧、渔业	Farming, Forestry, Animal Husbandry, Fishery Conservancy	270	317.4
2.工业	Industry	2471.9	3283.4
3.建筑业	Construction	47.1	71.2
4.交通运输、仓储和邮政业	Transport, Storage and Post	26.5	63.4
5.批发、零售业和住宿、餐饮业	Wholesale, Retail Trade and Hotel, Restaurants	16.8	38.0
6.其他	Others	68.8	121.7
7.生活消费	Residential Consumption	105.2	222.5
在消费量中:	Consumption by Usage		
(一) 终端消费	(I)Final Consumption	2763.4	3813.3
#工　业	Industry	2229.0	2979.1
(二) 输配电损失量	(II)Losses in Transmission	242.9	304.3

?1994-2018 China Academic Journal Electronic Publishing House. All rights reserved.　http://www.cnki.net

NATURAL GAS BALANCE SHEET

(100 million cu.m)

1990	1995	2000	2005	2006	2007	2008	2009	2010
**153.0**	**179.5**	**240.6**	**463.5**	**566.1**	**706.6**	**816.6**	**896.9**	**1072.9**
153.0	179.5	272.0	493.2	585.5	692.4	803.0	852.7	948.5
				9.5	40.2	46.0	76.3	164.7
		31.4	29.7	29.0	26.0	32.5	32.1	40.3
**152.5**	**177.4**	**245.0**	**467.6**	**561.4**	**705.2**	**812.9**	**895.2**	**1075.8**
								0.5
120.2	154.4	199.0	328.8	384.0	479.7	531.6	577.9	687.3
10.6	0.3	0.8	1.5	1.7	2.1	1.0	1.0	1.2
1.9	1.6	8.8	38.0	47.2	46.9	71.6	91.1	106.7
	0.6	3.4	10.8	13.2	17.1	17.8	24.0	27.2
1.2	1.2	0.6	9.1	12.8	16.1	20.9	23.6	26.0
18.6	19.4	32.3	79.4	102.6	143.4	170.1	177.7	226.9
**0.5**	**2.1**	**-4.4**	**-4.1**	**4.6**	**1.4**	**3.7**	**1.7**	**3.5**

a) Include the data of LNG since 2010.

ELECTRICITY BALANCE

(100 million kW•h)

1990	1995	2000	2005	2006	2007	2008	2009	2010
**6230.4**	**10023.4**	**13472.7**	**24940.8**	**28588.4**	**32712.4**	**34540.8**	**37032.7**	**41936.5**
6212.0	10077.3	13556.0	25002.6	28657.3	32815.5	34668.8	37146.5	42071.6
1267.2	1905.8	2224.1	3970.2	4357.9	4852.6	5851.9	6156.4	7221.7
4944.8	8043.2	11141.9	20473.4	23696.0	27229.3	27900.8	29827.8	33319.3
	128.3	167.4	530.9	548.4	621.3	683.9	701.3	738.8
								446.2
19.3	6.4	15.5	50.1	53.9	42.5	38.4	60.1	55.5
0.9	60.3	98.8	111.9	122.7	145.7	166.4	173.9	190.6
**6230.4**	**10023.4**	**13472.4**	**24940.3**	**28588.0**	**32711.8**	**34541.4**	**37032.2**	**41934.5**
426.8	582.4	533.0	776.3	827.0	879.0	887.1	939.9	976.5
4873.3	7659.8	10004.6	18521.7	21267.7	24290.8	25388.6	26854.5	30871.8
65.0	159.6	159.8	233.9	271.0	309.0	367.3	421.9	483.2
105.9	182.3	281.2	430.3	467.4	531.9	571.8	617.0	734.5
76.2	199.5	418.7	752.3	847.3	929.8	1017.4	1136.8	1292.0
202.4	234.2	623.2	1340.9	1555.9	1708.6	1913.0	2189.9	2451.8
480.8	1005.6	1452.0	2884.8	3351.6	4062.7	4396.1	4872.2	5124.6
5795.8	9278.9	12535.7	23233.8	26729.1	30650.1	32403.5	34773.9	39366.3
4438.7	6915.3	9067.9	16815.2	19408.9	22229.1	23250.8	24596.3	28303.5
434.6	744.5	936.7	1706.5	1858.8	2061.7	2137.9	2258.2	2568.2

?1994-2018 China Academic Journal Electronic Publishing House. All rights reserved. http://www.cnki.net
