# Supplementary material for: Prediction analysis of carbon emission in China’s electricity industry based on the dual carbon background
Source: PLoS One. 2024 May 17;19(5):e0302068. doi: 10.1371/journal.pone.0302068 (PMC11101092; doi:10.1371/journal.pone.0302068)
Supplement: S4 File — (ZIP) [file pone.0302068.s004.zip › China Energy Statistic Yearbook 2001-2021/五_全国能源平衡表_五_全国能源平衡表_文兼武总编_-2016.pdf]

5-1 中国能源平衡表(实物量) -2015

项目	Item	煤合计 (万吨) Coal Total (10^4 tons)	原煤 (万吨) Raw Coal (10^4 tons)
**一.可供本地区消费的能源量**	**Total Primary Energy Supply**	**397073.75**	**396371.63**
1.一次能源生产量	Indigenous Production	374654.16	374654.16
水电	Hydro Power		
核电	Nuclear Power		
风电	Wind Power		
2.进口量	Import	20406.48	20401.30
3.境内轮船和飞机在境外的加油量	Domestic Airplanes&Ships Refueling in Abroad		
4.出口量(-)	Export (-)	533.80	519.73
5.境外轮船和飞机在境内的加油量(-)	Oversea Airplanes&Ships Refueling in China (-)		
6.库存增(-)、减(+)量	Stock Change	2546.91	1835.90
**二.加工转换投入(-)产出(+)量**	**Input(-) & Output(+) of Transformation**	**-284818.72**	**-302290.15**
1.火力发电	Thermal Power	-179318.38	-176643.54
2.供热	Heating Supply	-24095.38	-23370.24
3.洗选煤	Coal Washing	-18337.66	-93395.97
4.炼焦	Coking	-60643.56	-6891.22
5.炼油及煤制油	Petroleum Refineries	-679.03	-518.32
#油品再投入量(-)	Petroleum Products Input (-)		
6.制气	Gas Works	-1270.45	-1132.67
#焦炭再投入量(-)	Coke Input (-)		
7.天然气液化	Natural Gas Liquefaction		
8.煤制品加工	Briquettes	-474.26	-338.19
9.回收能	Recovery of Energy		
**三.损失量**	**Loss**		
**四.终端消费量**	**Total Final Consumption**	**112195.35**	**94027.49**
1.农、林、牧、渔业	Agriculture, Forestry, Animal Husbandry and Fishery	2625.00	2581.03
2.工业	Industry	90831.25	74393.53
#用作原料、材料	Non-Energy Use	9451.26	8354.15
3.建筑业	Construction	878.06	851.29
4.交通运输、仓储和邮政业	Transport, Storage and Post	491.60	460.97
5.批发、零售业和住宿、餐饮业	Wholesale, Retail Trade and Hotel, Restaurants	3863.65	3767.18
6.其他	Others	4158.66	4043.84
7.生活消费	Residential Consumption	9347.13	7929.65
城镇	Urban	1361.50	990.12
乡村	Rural	7985.63	6939.53
**五.平衡差额**	**Statistical Difference**	**59.68**	**53.99**
**六.消费量合计**	**Total Energy Consumption**	**397014.07**	**396317.64**

?1994-2018 China Academic Journal Electronic Publishing House. All rights reserved. http://www.cnki.net

Energy Balance of China (Physical Quantity) -2015

洗精煤 (万吨) Cleaned Coal (10^4 tons)	其他洗煤 (万吨) Other Washed Coal (10^4 tons)	型煤 (万吨) Briquettes (10^4 tons)	煤矸石 (万吨) Gangue (10^4 tons)	焦炭 (万吨) Coke (10^4 tons)	焦炉煤气 (亿立方米) Coke Oven Gas (10^8 cu.m)	高炉煤气 (亿立方米) Blast Furnace Gas (10^8 cu.m)	转炉煤气 (亿立方米) Converter Gas (10^8 cu.m)	其他煤气 (亿立方米) Other Gas (10^8 cu.m)
**614.85**	**99.26**	**-11.99**		**-803.63**				
		5.18		0.38				
		14.07		964.82				
614.85	99.26	-3.10		160.81				
**5261.27**	**10284.23**	**1925.93**	**-11.50**	**44538.75**	**590.74**	**5520.91**	**360.31**	**153.19**
-31.82	-2643.02		-2709.60	-5.92	-169.63	-1465.75	-113.46	-2.80
-56.03	-669.11		-725.34	-276.03	-60.85	-620.23	-45.40	-0.51
59304.68	15753.63		3423.44					
-53674.91	-77.43			44633.92	817.32			
-149.89	-10.82							
-130.76	-7.02			188.62	3.90			156.50
				-1.84				
	-2062.00	1925.93						
						7606.89	519.17	
**5854.30**	**10408.35**	**1905.21**		**43774.95**	**588.51**	**5522.55**	**356.90**	**153.09**
	43.97			49.49				
5837.17	9281.06	1319.49		43639.19	566.70	5522.55	356.90	86.29
663.69	433.43			1745.83	14.53			
8.09	18.68			6.68				
9.04	21.59			3.02				
	62.08	34.39		40.06	0.91			6.14
	102.25	12.57		5.35	1.18			
	878.72	538.77		31.16	19.72			60.66
	203.44	167.94		9.17	19.72			60.46
	675.28	370.82		21.99				0.20
**21.82**	**-24.86**	**8.73**	**-11.50**	**-39.83**	**2.23**	**-1.64**	**3.41**	**0.10**
**59897.71**	**15877.75**	**1905.21**	**3434.94**	**44058.74**	**818.99**	**7608.53**	**515.76**	**156.40**

?1994-2018 China Academic Journal Electronic Publishing House. All rights reserved. http://www.cnki.net

5-1 续表 1

项　目	Item	其他焦化产品 (万吨) Other Coking Products (10^4 tons)	油品合计 (万吨) Petroleum Products Total (10^4 tons)
**一.可供本地区消费的能源量**	**Total Primary Energy Supply**		**55188.00**
1.一次能源生产量	Indigenous Production		21455.58
水电	Hydro Power		
核电	Nuclear Power		
风电	Wind Power		
2.进口量	Import		38824.29
3.境内轮船和飞机在境外的加油量	Domestic Airplanes&Ships Refueling in Abroad		924.33
4.出口量(-)	Export (-)		4373.46
5.境外轮船和飞机在境内的加油量(-)	Oversea Airplanes&Ships Refueling in China (-)		754.69
6.库存增(-)、减(+)量	Stock Change		-888.05
**二.加工转换投入(-)产出(+)量**	**Input(-) & Output(+) of Transformation**	**1074.33**	**-2626.94**
1.火力发电	Thermal Power		-265.54
2.供热	Heating Supply		-493.21
3.洗选煤	Coal Washing		
4.炼焦	Coking	1183.11	
5.炼油及煤制油	Petroleum Refineries	-121.43	7133.09
#油品再投入量(-)	Petroleum Products Input (-)		-9001.28
6.制气	Gas Works	21.87	
#焦炭再投入量(-)	Coke Input (-)	-9.22	
7.天然气液化	Natural Gas Liquefaction		
8.煤制品加工	Briquettes		
9.回收能	Recovery of Energy		
**三.损失量**	**Loss**		**87.56**
**四.终端消费量**	**Total Final Consumption**	**1077.23**	**52445.68**
1.农、林、牧、渔业	Agriculture, Forestry, Animal Husbandry and Fishery		1733.41
2.工业	Industry	1077.23	16229.74
#用作原料、材料	Non-Energy Use	392.67	8254.95
3.建筑业	Construction		3507.50
4.交通运输、仓储和邮政业	Transport, Storage and Post		20513.75
5.批发、零售业和住宿、餐饮业	Wholesale, Retail Trade and Hotel, Restaurants		615.70
6.其他	Others		3683.34
7.生活消费	Residential Consumption		6162.25
城镇	Urban		4305.58
乡村	Rural		1856.67
**五.平衡差额**	**Statistical Difference**	**-2.90**	**27.82**
**六.消费量合计**	**Total Energy Consumption**	**1207.88**	**55160.18**

?1994-2018 China Academic Journal Electronic Publishing House. All rights reserved. http://www.cnki.net

Continued 1

原油 (万吨) Crude Oil (10^4 tons)	汽油 (万吨) Gasoline (10^4 tons)	煤油 (万吨) Kerosene (10^4 tons)	柴油 (万吨) Diesel Oil (10^4 tons)	燃料油 (万吨) Fuel Oil (10^4 tons)	石脑油 (万吨) Naphtha (10^4 tons)	润滑油 (万吨) Lubricants (10^4 tons)	石蜡 (万吨) Paraffin Waxes (10^4 tons)	溶剂油 (万吨) White Spirit (10^4 tons)
**54093.50**	**-718.52**	**-926.01**	**-654.27**	**669.77**	**632.72**	**20.72**	**-53.86**	**-7.06**
21455.58								
33548.28	17.03	348.45	42.80	1540.40	664.72	32.56	8.49	2.21
		367.93	28.65	527.75				
286.56	589.29	1237.33	716.25	1051.69		11.84	62.35	0.42
		389.29	15.00	350.40				
-623.80	-146.26	-15.77	5.53	3.71	-32.00			-8.85
**-53218.37**	**12103.01**	**3658.62**	**17928.02**	**1424.73**	**3934.63**	**113.58**	**161.33**	**147.26**
-12.46	-0.17		-22.36	-31.52				
-6.69	-0.07		-6.22	-165.13				
-53199.22	12103.56	3658.62	18007.89	3963.01	4585.04	117.17	161.35	147.26
	-0.31		-51.29	-2341.63	-650.41	-3.59	-0.02	
**87.22**								
**782.69**	**11367.91**	**2663.71**	**17280.44**	**2123.73**	**4573.66**	**133.80**	**108.20**	**142.00**
	231.33	1.10	1492.88	0.94				
782.69	476.53	21.16	1436.50	594.75	4573.66	133.80	108.20	142.00
135.66	9.33	1.94	27.50	131.74	4249.48	116.67	105.54	136.46
	408.57	12.50	555.71	53.51				
	5306.59	2504.88	11162.80	1439.49				
	243.29	11.68	257.74	18.95				
	2108.47	83.27	1384.15	16.08				
	2593.11	29.13	990.66					
	1804.08	3.74	549.70					
	789.03	25.39	440.96					
**5.22**	**16.58**	**68.90**	**-6.69**	**-29.23**	**-6.31**	**0.50**	**-0.73**	**-1.80**
**54088.28**	**11368.46**	**2663.71**	**17360.31**	**4662.01**	**5224.07**	**137.39**	**108.22**	**142.00**

?1994-2018 China Academic Journal Electronic Publishing House. All rights reserved. http://www.cnki.net

5-1 续表 2

项 目	Item	石油沥青 (万吨) Bitumen Asphalt (10^4 tons)	石油焦 (万吨) Petroleum Coke (10^4 tons)
**一.可供本地区消费的能源量**	**Total Primary Energy Supply**	**441.89**	**361.84**
1.一次能源生产量	Indigenous Production		
水电	Hydro Power		
核电	Nuclear Power		
风电	Wind Power		
2.进口量	Import	470.59	588.76
3.境内轮船和飞机在境外的加油量	Domestic Airplanes&Ships Refueling in Abroad		
4.出口量(-)	Export (-)	28.70	241.92
5.境外轮船和飞机在境内的加油量(-)	Oversea Airplanes&Ships Refueling in China (-)		
6.库存增(-)、减(+)量	Stock Change		15.00
**二.加工转换投入(-)产出(+)量**	**Input(-) & Output(+) of Transformation**	**2007.80**	**1613.62**
1.火力发电	Thermal Power		-136.27
2.供热	Heating Supply		-150.51
3.洗选煤	Coal Washing		
4.炼焦	Coking		
5.炼油及煤制油	Petroleum Refineries	2016.52	1900.40
#油品再投入量(-)	Petroleum Products Input (-)	-8.72	
6.制气	Gas Works		
#焦炭再投入量(-)	Coke Input (-)		
7.天然气液化	Natural Gas Liquefaction		
8.煤制品加工	Briquettes		
9.回收能	Recovery of Energy		
**三.损失量**	**Loss**		
**四.终端消费量**	**Total Final Consumption**	**2451.09**	**1975.82**
1.农、林、牧、渔业	Agriculture, Forestry, Animal Husbandry and Fishery		
2.工业	Industry	68.79	1975.82
#用作原料、材料	Non-Energy Use	39.58	1351.11
3.建筑业	Construction	2382.30	
4.交通运输、仓储和邮政业	Transport, Storage and Post		
5.批发、零售业和住宿、餐饮业	Wholesale, Retail Trade and Hotel, Restaurants		
6.其他	Others		
7.生活消费	Residential Consumption		
城镇	Urban		
乡村	Rural		
**五.平衡差额**	**Statistical Difference**	**-1.40**	**-0.36**
**六.消费量合计**	**Total Energy Consumption**	**2459.81**	**2262.60**

?1994-2018 China Academic Journal Electronic Publishing House. All rights reserved. http://www.cnki.net

Continued 2

液化石油气 (万吨) LPG (10^4 tons)	炼厂干气 (万吨) Refinery Gas (10^4 tons)	其他石油制品 (万吨) Other Petroleum Products (10^4 tons)	天然气 (亿立方米) Natural Gas (10^8 cu.m)	液化天然气 (万吨) LNG (10^4 tons)	热力 (万百万千焦) Heat (10^{10} kJ)	电力 (亿千瓦小时) Electricity (10^8 kW•h)	其他能源 (万吨标煤) Other Energy (10^4 tce)
**1073.80**		**253.48**	**1654.29**	**1961.87**		**15179.41**	**5751.10**
			1346.10			15303.85	5751.10
						11302.70	
						1707.89	
						1857.66	
1243.95		316.05	340.64	1961.87		62.10	
144.15		2.96	32.45			186.54	
-26.00		-59.61					
**2552.12**	**1570.69**	**3376.02**	**-430.66**	**345.57**	**401604.52**	**42841.88**	**-223.42**
	-50.42	-12.34	-291.59	-163.12	-59452.63	42841.88	-1058.97
-3.27	-131.71	-29.61	-61.70	-9.84	399029.66		-310.67
2934.42	1853.65	8883.42	-3.75				-38.86
-379.03	-100.83	-5465.45					
			3.24				
			-76.86	518.53			
					62027.49		1185.08
**0.34**			**20.38**	**13.00**	**4875.29**	**2987.86**	
**3578.56**	**1576.01**	**3688.06**	**1209.66**	**2296.70**	**396778.72**	**55032.12**	**5526.78**
7.16			0.95		106.70	1039.83	492.73
731.57	1576.01	3608.26	559.36	2040.00	280611.96	38562.13	1231.53
233.99	34.20	1681.75	96.94	182.17			
15.10		79.80	2.16		903.67	698.67	35.65
99.98			190.65	256.70	2809.72	1125.61	1171.85
84.04			51.29		6113.91	2122.04	92.52
91.36			45.44		12391.77	3918.63	298.37
2549.35			359.81		93840.97	7565.21	2204.13
1948.06			358.38		93840.97	4103.94	249.53
601.29			1.43			3461.27	1954.60
**47.02**	**-5.32**	**-58.56**	**-6.41**	**-2.26**	**-49.49**	**1.31**	**0.90**
**3961.20**	**1858.97**	**9195.46**	**1589.14**	**2482.66**	**461106.64**	**58019.98**	**6935.28**

?1994-2018 China Academic Journal Electronic Publishing House. All rights reserved. http://www.cnki.net

5-2 中国能源平衡表(标准量) -2015

单位：万吨标准煤

项　目	Item	能源合计 (发电煤耗计算法) (coal equivalent calculation)	Energy Total (电热当量计算法) (calorific value calculation)
**一.可供本地区消费的能源量**	**Total Primary Energy Supply**	**429960.16**	**402217.03**
1.一次能源生产量	Indigenous Production	361475.51	333504.95
水电	Hydro Power	34548.76	13891.02
核电	Nuclear Power	5220.48	2099.00
风电	Wind Power	5678.28	2283.06
2.进口量	Import	76114.18	76000.68
3.境内轮船和飞机在境外的加油量	Domestic Airplanes&Ships Refueling in Abroad	1337.06	1337.06
4.出口量(-)	Export (-)	8688.77	8347.84
5.境外轮船和飞机在境内的加油量(-)	Oversea Airplanes&Ships Refueling in China (-)	1095.24	1095.24
6.库存增(-)、减(+)量	Stock Change	817.41	817.41
**二.加工转换投入(-)产出(+)量**	**Input(-) & Output(+) of Transformation**	**-2698.85**	**-81000.17**
1.火力发电	Thermal Power		-78301.32
2.供热	Heating Supply	-5291.00	-5291.00
3.洗选煤	Coal Washing	-4996.49	-4996.49
4.炼焦	Coking	-4098.80	-4098.80
5.炼油及煤制油	Petroleum Refineries	10259.45	10259.45
#油品再投入量(-)	Petroleum Products Input (-)	-12489.85	-12489.85
6.制气	Gas Works	-318.65	-318.65
#焦炭再投入量(-)	Coke Input (-)	-12.43	-12.43
7.天然气液化	Natural Gas Liquefaction	-88.02	-88.02
8.煤制品加工	Briquettes	-154.77	-154.77
9.回收能	Recovery of Energy	14491.71	14491.71
**三.损失量**	**Loss**	**9712.15**	**4251.30**
**四.终端消费量**	**Total Final Consumption**	**417494.08**	**316912.88**
1.农、林、牧、渔业	Agriculture, Forestry, Animal Husbandry and Fishery	8231.66	6331.18
2.工业	Industry	280205.66	209726.35
#用作原料、材料	Non-Energy Use	22100.70	22100.70
3.建筑业	Construction	7696.41	6419.47
4.交通运输、仓储和邮政业	Transport, Storage and Post	37976.93	35919.67
5.批发、零售业和住宿、餐饮业	Wholesale, Retail Trade and Hotel, Restaurants	11403.69	7525.28
6.其他	Others	21880.78	14718.77
7.生活消费	Residential Consumption	50098.96	36272.17
城镇	Urban	28668.14	21167.44
乡村	Rural	21430.83	15104.73
**五.平衡差额**	**Statistical Difference**	**55.07**	**52.68**
**六.消费量合计**	**Total Energy Consumption**	**429905.09**	**402164.35**

?1994-2018 China Academic Journal Electronic Publishing House. All rights reserved. http://www.cnki.net

Energy Balance of China (Standard Quantity) -2015

(10 000 tce)

煤合计<br>Coal Total	原煤<br>Raw Coal	洗精煤<br>Cleaned Coal	其他洗煤<br>Other Washed Coal	型煤<br>Briquettes	煤矸石<br>Gangue	焦炭<br>Coke	焦炉煤气<br>Coke Oven Gas	高炉煤气<br>Blast Furnace Gas	转炉煤气<br>Converter Gas
**274744.93**	**274146.24**	**553.37**	**52.61**	**-7.28**		**-780.65**			
260794.67	260794.67								
12474.98	12471.83			3.15		0.37			
471.22	462.68			8.54		937.23			
1946.50	1342.41	553.37	52.61	-1.88		156.21			
**-195909.46**	**-207264.67**	**4735.14**	**5450.64**	**1169.43**	**66.17**	**43264.94**	**3375.49**	**7099.89**	**977.88**
-119741.17	-118311.73	-28.64	-1400.80		-541.92	-5.75	-969.27	-1884.95	-307.93
-15420.58	-15015.53	-50.43	-354.63		-145.07	-268.14	-347.70	-797.62	-123.22
-5749.65	-67473.28	53374.21	8349.42		753.16				
-53491.67	-5143.21	-48307.42	-41.04			43357.39	4670.17		
-480.58	-339.95	-134.90	-5.73						
-871.03	-749.62	-117.68	-3.7206			183.23	22.28		
						-1.79			
-154.77	-231.34		-1092.86	1169.43					
								9782.46	1409.03
**78801.98**	**66859.84**	**5268.87**	**5516.42**	**1156.85**		**42522.99**	**3362.75**	**7102.00**	**968.63**
1965.56	1942.26		23.31			48.07			
62686.28	51712.67	5253.45	4918.96	801.19		42391.11	3238.12	7102.00	968.63
6804.08	5977.05	597.32	229.72			1695.90	83.02		
701.15	683.97	7.28	9.90			6.49			
349.13	329.55	8.14	11.44			2.93			
2972.64	2918.86		32.90	20.88		38.91	5.21		
3157.79	3095.96		54.19	7.63		5.20	6.74		
6969.44	6176.58		465.72	327.14		30.27	112.68		
985.17	775.37		107.82	101.98		8.91	112.68		
5984.27	5401.21		357.90	225.16		21.36			
**33.49**	**21.73**	**19.64**	**-13.17**	**5.30**	**66.17**	**-38.69**	**12.74**	**-2.11**	**9.25**

?1994-2018 China Academic Journal Electronic Publishing House. All rights reserved. http://www.cnki.net

5-2 续表 1

单位：万吨标准煤

项 目	Item	其他煤气 Gasoline	其他焦化产品 Other Coking Products
**一.可供本地区消费的能源量**	**Total Primary Energy Supply**		
1.一次能源生产量	Indigenous Production		
水电	Hydro Power		
核电	Nuclear Power		
风电	Wind Power		
2.进口量	Import		
3.境内轮船和飞机在境外的加油量	Domestic Airplanes&Ships Refueling in Abroad		
4.出口量(-)	Export (-)		
5.境外轮船和飞机在境内的加油量(-)	Oversea Airplanes&Ships Refueling in China (-)		
6.库存增(-)、减(+)量	Stock Change		
**二.加工转换投入(-)产出(+)量**	**Input(-) & Output(+) of Transformation**	**273.60**	**1239.78**
1.火力发电	Thermal Power	-5.00	
2.供热	Heating Supply	-0.91	
3.洗选煤	Coal Washing		
4.炼焦	Coking		1365.31
5.炼油及煤制油	Petroleum Refineries		-140.13
#油品再投入量(-)	Petroleum Products Input (-)		
6.制气	Gas Works	279.51	25.24
#焦炭再投入量(-)	Coke Input (-)		-10.64
7.天然气液化	Natural Gas Liquefaction		
8.煤制品加工	Briquettes		
9.回收能	Recovery of Energy		
**三.损失量**	**Loss**		
**四.终端消费量**	**Total Final Consumption**	**273.41**	**1243.12**
1.农、林、牧、渔业	Agriculture, Forestry, Animal Husbandry and Fishery		
2.工业	Industry	154.11	1243.12
#用作原料、材料	Non-Energy Use		453.14
3.建筑业	Construction		
4.交通运输、仓储和邮政业	Transport, Storage and Post		
5.批发、零售业和住宿、餐饮业	Wholesale, Retail Trade and Hotel, Restaurants	10.97	
6.其他	Others		
7.生活消费	Residential Consumption	108.33	
城镇	Urban	107.98	
乡村	Rural	0.36	
**五.平衡差额**	**Statistical Difference**	**0.19**	**-3.35**
**六.消费量合计**	**Total Energy Consumption**		

?1994-2018 China Academic Journal Electronic Publishing House. All rights reserved. http://www.cnki.net

Continued 1

(10 000 tce)

油品合计 Petroleum Products Total	原油 Crude Oil	汽油 Gasoline	煤油 Kerosene	柴油 Diesel Oil	燃料油 Fuel Oil	石脑油 Naphtha	润滑油 Lubricants	石蜡 Paraffin Waxes
**78892.98**	**77277.97**	**-1057.23**	**-1362.53**	**-953.34**	**956.83**	**949.08**	**29.30**	**-73.51**
30651.44	30651.44							
55573.30	47927.07	25.06	512.71	62.36	2200.62	997.08	46.05	11.59
1337.06			541.37	41.75	753.94			
6288.28	409.38	867.08	1820.61	1043.65	1502.44		16.75	85.10
1095.24			572.80	21.86	500.58			
-1285.30	-891.16	-215.21	-23.20	8.06	5.30	-48.00		
**-2521.08**	**-76027.76**	**17808.37**	**5383.29**	**26122.92**	**2035.37**	**5901.95**	**160.64**	**220.18**
-334.39	-17.80	-0.25		-32.58	-45.03			
-664.62	-9.56	-0.10		-9.06	-235.90			
10967.77	-76000.41	17809.18	5383.29	26239.30	5661.56	6877.56	165.71	220.21
-12489.85		-0.46		-74.73	-3345.25	-975.615	-5.077337	-0.027296
**125.19**	**124.60**							
**76185.23**	**1118.15**	**16726.74**	**3919.39**	**25179.33**	**3033.96**	**6860.49**	**189.23**	**147.67**
2530.88		340.38	1.62	2175.27	1.34			
22893.36	1118.15	701.17	31.13	2093.12	849.66	6860.49	189.23	147.67
11484.26	193.80	13.73	2.85	40.07	188.20	6374.22	165.01	144.04
4758.57		601.18	18.39	809.72	76.45			
29986.98		7808.12	3685.68	16265.32	2056.46			
921.85		357.98	17.18	375.55	27.07			
5421.37		3102.41	122.52	2016.85	22.97			
9672.20		3815.51	42.86	1443.49				
6800.55		2654.53	5.50	800.96				
2871.66		1160.98	37.36	642.53				
**61.48**	**7.46**	**24.40**	**101.37**	**-9.75**	**-41.75**	**-9.46**	**0.71**	**-1.00**

?1994-2018 China Academic Journal Electronic Publishing House. All rights reserved. http://www.cnki.net

5-2 续表 2

单位: 万吨标准煤

项　目	Item	溶剂油 White spirit	石油沥青 Bitumen Asphalt
**一.可供本地区消费的能源量**	**Total Primary Energy Supply**	**-10.36**	**578.88**
1.一次能源生产量	Indigenous Production		
水电	Hydro Power		
核电	Nuclear Power		
风电	Wind Power		
2.进口量	Import	3.24	616.47
3.境内轮船和飞机在境外的加油量	Domestic Airplanes&Ships Refueling in Abroad		
4.出口量(-)	Export (-)	0.62	37.60
5.境外轮船和飞机在境内的加油量(-)	Oversea Airplanes&Ships Refueling in China (-)		
6.库存增(-)、减(+)量	Stock Change	-12.98	
**二.加工转换投入(-)产出(+)量**	**Input(-) & Output(+) of Transformation**	**216.06**	**2630.22**
1.火力发电	Thermal Power		
2.供热	Heating Supply		
3.洗选煤	Coal Washing		
4.炼焦	Coking		
5.炼油及煤制油	Petroleum Refineries	216.06	2641.64
#油品再投入量(-)	Petroleum Products Input (-)		-11.4232
6.制气	Gas Works		
#焦炭再投入量(-)	Coke Input (-)		
7.天然气液化	Natural Gas Liquefaction		
8.煤制品加工	Briquettes		
9.回收能	Recovery of Energy		
**三.损失量**	**Loss**		
**四.终端消费量**	**Total Final Consumption**	**208.34**	**3210.93**
1.农、林、牧、渔业	Agriculture, Forestry, Animal Husbandry and Fishery		
2.工业	Industry	208.34	90.12
#用作原料、材料	Non-Energy Use	200.21	51.85
3.建筑业	Construction		3120.81
4.交通运输、仓储和邮政业	Transport, Storage and Post		
5.批发、零售业和住宿、餐饮业	Wholesale, Retail Trade and Hotel, Restaurants		
6.其他	Others		
7.生活消费	Residential Consumption		
城镇	Urban		
乡村	Rural		
**五.平衡差额**	**Statistical Difference**	**-2.64**	**-1.83**
**六.消费量合计**	**Total Energy Consumption**		

?1994-2018 China Academic Journal Electronic Publishing House. All rights reserved. http://www.cnki.net

Continued 2

(10 000 tce)

石油焦 Petroleum Coke	液化石油气 LPG	炼厂干气 Refinery Gas	其他石油制品 Other Petroleum Products	天然气 Natural Gas	液化天然气 LNG	热力 Heat	电力 Electricity	其他能源 Other Energy
**379.93**	**1840.82**		**337.13**	**21505.77**	**3447.40**		**18655.49**	**5751.10**
				17499.30			18808.43	5751.10
							13891.02	
							2099.00	
							2283.06	
618.20	2132.50		420.35	4428.32	3447.40		76.32	
254.02	247.12		3.94	421.85			229.26	
15.75	-44.57		-79.28					
**1694.30**	**4375.10**	**2468.18**	**4490.11**	**-5598.58**	**607.24**	**13694.71**	**52652.67**	**-223.42**
-143.08		-79.23	-16.41	-3790.67	-286.63	-2027.33	52652.67	-1058.97
-158.04	-5.61	-206.97	-39.38	-802.10	-17.29	13606.91		-310.67
1995.42	5030.48	2912.83	11814.95	-48.75				-38.86
	-649.77	-158.44	-7269.05					
				42.12				
				-999.18	911.16			
						2115.14		1185.08
	**0.58**			**264.94**	**22.84**	**166.25**	**3672.08**	
**2074.61**	**6134.72**	**2476.54**	**4905.12**	**15725.59**	**4035.77**	**13530.15**	**67634.48**	**5526.78**
	12.27			12.34		3.64	1277.95	492.73
2074.61	1254.13	2476.54	4798.99	7271.68	3584.69	9568.87	47392.86	1231.53
1418.67	401.13	53.74	2236.73	1260.18	320.11			
	25.89		106.13	28.12		30.82	858.67	35.65
	171.40			2478.51	451.08	95.81	1383.37	1171.85
	144.07			666.71		208.48	2607.99	92.52
	156.62			590.74		422.56	4816.00	298.37
	4370.35			4677.49		3199.98	9297.64	2204.13
	3339.56			4658.91		3199.98	5043.74	249.53
	1030.79			18.58			4253.90	1954.60
**-0.38**	**80.61**	**-8.36**	**-77.88**	**-83.34**	**-3.98**	**-1.69**	**1.61**	**0.90**

?1994-2018 China Academic Journal Electronic Publishing House. All rights reserved. http://www.cnki.net

5-3 综合能源平衡表

单位：万吨标准煤

项　目	Item	1980	1985	1990
**可供消费的能源总量**	**Total Energy Available for Consumption**	**61557**	**77603**	**96138**
一次能源生产量	Primary Energy Output	63735	85546	103922
回收能	Recovery of Energy			
进口量	Imports	261	340	1310
出口量(-)	Exports (-)	3058	5774	5875
年初年末库存差额	Stock Changes in the Year	619	-2509	-3219
**能源消费总量**	**Total Energy Consumption**	**60275**	**76682**	**98703**
在总量中:	Consumption by Sector			
1.农、林、牧、渔业	Agriculture, Forestry, Animal Husbandry and Fishery	4692	4045	4852
2.工业	Industry	38986	51068	67578
3.建筑业	Construction	957	1302	1213
4.交通运输、仓储和邮政业	Transport, Storage and Post	2902	3713	4541
5.批发、零售业和住宿、餐饮业	Wholesale, Retail Trade and Hotel, Restaurants	518	766	1247
6.其他	Others	1205	2470	3473
7.生活消费	Residential Consumption	11015	13318	15799
在总量中:	Consumption by Usage			
(一) 终端消费	(I) Final Consumption	57508	73586	94289
#工业	Industry	38293	48021	63239
(二) 加工转换损失量	(II) Losses in Processing and	1358	1491	2264
#炼焦	Coking	644	572	905
炼油	Petroleum Refining	113	110	326
(三) 回收能(-)	(III) Recovery of Energy(-)			
(四) 损失量	(IV) Other Losses	1409	1605	2150
**平衡差额**	**Balance**	**1282**	**921**	**-2565**

注：1.村办工业包括在工业中(下同)。
2.电力按等价热值折算，因此加工转换损失量中不包括发电损失量。
3.进口量包括境内轮船和飞机在境外的加油量；出口量包括境外轮船和飞机在境内的加油量。

?1994-2018 China Academic Journal Electronic Publishing House. All rights reserved. http://www.cnki.net

Overall Energy Balance Sheet

(10 000 tce)

1995	2000	2005	2010	2011	2012	2013	2014	2015
**129535**	**144234**	**254619**	**365588**	**390394**	**407594**	**417415**	**426095**	**429960**
129034	138570	229037	312125	340178	351041	358784	361866	361476
2312	3087	7452	8958					
5456	14327	26823	57671	65437	68701	73420	77325	77451
6776	9327	11257	8803	8449	7374	8005	8271	9784
-491	-2424	2564	-4363	-6772	-4773	-6784	-4825	817
**131176**	**146964**	**261369**	**360648**	**387043**	**402138**	**416913**	**425806**	**429905**
5505	4233	6860	7266	7675	7804	8055	8094	8232
96191	103014	187914	261377	278048	284712	291130	295686	292276
1335	2207	3486	5533	6052	6337	7017	7520	7696
5863	11447	19136	27102	29694	32561	34819	36336	38318
2018	3251	5917	7847	9147	10012	10598	10873	11404
4519	6118	10484	15052	16843	18407	19763	20084	21881
15745	16695	27573	36470	39584	42306	45531	47212	50099
124252	140476	250877	337469	373296	386888	403814	413162	417494
89473	96871	177775	238652	264698	269900	278514	283420	280206
3634	2472	3882	14294	15412	16763	15994	17020	17191
	526	855	1595	1833	2179	2433	2731	4099
	781	1273	1960	1792	2153	1899	2115	2230
				10864	11239	13333	14578	14492
3289	4016	6610	8885	9199	9726	10439	10201	9712
**-1641**	**-2730**	**-6751**	**4940**	**3350**	**5456**	**502**	**289**	**55**

a) Data on industry include the data of village-run industry.(The same as in the following tables).
b) Electric power is converted on the basic of equal caloric value. Therefore, losses in processing and transformation exclude losses in power generation.
c) Data on imports include the petroleum consumed by the domestic airplanes and ships in refueling abroad. Data on exports include the petroleum consumed by the oversea airplanes and ships in refueling in China.

?1994-2018 China Academic Journal Electronic Publishing House. All rights reserved. http://www.cnki.net

5-4 煤炭平衡表

单位：万吨

项 目	Item	1980	1985	1990
**可供量**	**Total Energy Available for Consumption**	**62601**	**82777**	**102221**
生产量	Output	62015	87228	107988
进口量	Imports	199	231	200
出口量(−)	Exports (-)	632	777	1729
年初年末库存差额	Stock Changes in the Year	1019	-3906	-4239
**消费量**	**Total Energy Consumption**	**61010**	**81603**	**105523**
在消费量中:	Consumption by Sector			
1.农、林、牧、渔业	Agriculture, Forestry, Animal Husbandry and Fishery	1550	2209	2095
2.工业	Industry	43848	58613	81091
3.建筑业	Construction	556	532	438
4.交通运输、仓储和邮政业	Transport, Storage and Post	1934	2307	2161
5.批发、零售业和住宿、餐饮业	Wholesale, Retail Trade and Hotel, Restaurants	455	738	1058
6.其他	Others	1091	1580	1980
7.生活消费	Residential Consumption	11574	15624	16700
在消费量中:	Consumption by Usage			
(一) 终端消费	(I) Final Consumption	38804	52704	60206
#工业	Industry	21643	29715	35774
(二) 中间消费	(II) Intermediate Consumption			
(用于加工转换)	(Consumed in Transformation)	22205	28899	45317
#发电	Power Generation	12648	16441	27204
供热	Heating		1462	2996
炼焦	Coking	6682	7304	10698
炼油及煤制油	Petroleum Refineries			
制气	Gas Production	131	191	360
(三)洗选损耗	(III) Losses in Coal Washing and Dressing	2744	3501	4059
**平衡差额**	**Balance**	**1592**	**1174**	**-3302**

注：生产量为原煤产量。

?1994-2018 China Academic Journal Electronic Publishing House. All rights reserved. http://www.cnki.net

Coal Balance Sheet

(10 000 tons)

1995	2000	2005	2010	2011	2012	2013	2014	2015
**133462**	**131895**	**235508**	**355578**	**393058**	**418654**	**425015**	**411834**	**397074**
136073	138418	236515	342845	376444	394513	397432	387392	374654
164	218	2622	18307	22236	28841	32702	29122	20406
2862	5506	7173	1911	1467	927	751	574	534
87	-1235	3545	-3663	-4155	-3772	-4368	-4106	2547
**137677**	**135690**	**243375**	**349008**	**388961**	**411727**	**424426**	**411613**	**397014**
1857	1051	1802	2147	2207	2266	2451	2579	2625
117571	121807	224766	329728	368916	391191	403157	390497	375650
440	537	604	731	797	767	811	914	878
1315	882	811	639	646	614	615	558	492
977	1461	2627	3192	3572	3752	3966	3767	3864
1987	1495	2727	3412	3612	3883	4136	4046	4159
13530	8457	10039	9159	9212	9253	9290	9253	9347
66156	50511	86386	114826	120647	118957	119491	116044	112195
46050	36628	67776	95546	100602	98421	98222	94928	90831
71520	81987	152208	222948	252691	266016	282355	272194	266481
44440	55811	103663	153742	175579	183531	195177	184525	179318
5887	8794	13542	17553	19334	23780	22710	22445	24095
18396	16496	33446	49950	56060	56768	62536	62894	60644
			213	346	378	459	650	679
764	960	1277	1040	870	849	846	948	1270
2033	3191	4782	11235	15623	26754	22579	23375	18338
**-4215**	**-3795**	**-7868**	**6569**	**4097**	**6928**	**589**	**220**	**60**

a) Data on output refer to the output of raw coal.

?1994-2018 China Academic Journal Electronic Publishing House. All rights reserved. http://www.cnki.net

5-5 焦炭平衡表

单位: 万吨

项　目	Item	1980	1985	1990
**可供量**	**Total Energy Available for Consumption**	**4315.3**	**4689.7**	**7085.8**
生产量	Output	4343.0	4802.1	7328.3
进口量	Imports		2.1	
出口量(-)	Exports (-)	27.1	36.9	129.0
年初年末库存差额	Stock Changes in the Year	-0.6	-77.6	-113.5
**消费量**	**Total Energy Consumption**	**4303.0**	**4689.7**	**6914.7**
在消费量中:	Consumption by Sector			
1.农、林、牧、渔业	Agriculture, Forestry, Animal Husbandry and Fishery	10.6	20.8	60.1
2.工业	Industry	4266.7	4627.7	6808.8
3.建筑业	Construction	11.9	7.8	5.2
4.交通运输、仓储和邮政业	Transport, Storage and Post	8.2	5.7	4.1
5.批发、零售业和住宿、餐饮业	Wholesale, Retail Trade and Hotel, Restaurants	0.9	2.7	7.7
6.其他	Others	4.7	2.0	1.9
7.生活消费	Residential Consumption		23.0	26.9
在消费量中:	Consumption by Usage			
(一) 终端消费	(I) Final Consumption	4294.7	4677.9	6846.3
#工业	Industry	4258.4	4615.9	6740.4
(二) 中间消费	(II) Intermediate Consumption			
(用于加工转换)	(Consumed in Transformation)	8.3	11.8	68.4
制气	Gas Production	8.3	11.8	68.4
(三) 损失量	(III) Losses in Coal Washing and Dressing			
**平衡差额**	**Balance**	**12.3**		**171.1**

?1994-2018 China Academic Journal Electronic Publishing House. All rights reserved. http://www.cnki.net

Coke Balance Sheet

(10 000 tons)

1995	2000	2005	2010	2011	2012	2013	2014	2015
**12207.1**	**10892.3**	**25084.4**	**38707.1**	**42085.8**	**44813.7**	**45850.1**	**46894.3**	**44018.9**
13424.5	12184.0	26511.7	38657.8	43433.0	43831.4	48347.8	47980.9	44822.5
0.1		0.5	11.0	11.6	7.6	3.5	0.2	0.4
886.1	1519.7	1276.4	335.0	329.7	102.0	467.3	850.7	964.8
-331.4	228.0	-151.4	373.3	-1029.0	1076.7	-2034.0	-236.0	160.8
**10725.3**	**10840.8**	**25105.8**	**38702.8**	**42063.3**	**44805.2**	**45851.9**	**46884.9**	**44058.7**
128.6	70.9	63.5	46.8	54.1	57.5	69.2	34.9	49.5
10412.0	10554.6	24860.9	38598.7	41952.1	44694.8	45694.0	46749.6	43923.0
10.8	19.0	18.4	5.8	4.8	6.3	7.7	9.7	6.7
10.1	11.2	1.1	0.1	0.1	0.1	2.2	2.7	3.0
25.7	35.7	64.1	5.1	9.2	6.7	35.8	46.6	40.1
6.4	12.2	7.6	2.8	1.9	1.9	5.0	5.1	5.4
131.6	137.2	90.3	43.5	41.1	37.9	38.0	36.4	31.2
10648.0	10697.9	24877.9	38574.6	41954.3	44738.5	45817.5	46589.1	43775.0
10334.7	10411.7	24633.0	38470.5	41843.1	44628.1	45659.6	46453.8	43639.2
77.3	142.9	227.9	128.2	109.0	66.7	34.4	295.8	283.8
77.3	142.9	227.9	128.2	109.0	66.7	34.4	32.8	1.8
**1481.8**	**51.6**	**-21.4**	**4.3**	**22.6**	**8.5**	**-1.8**	**9.4**	**-39.8**

?1994-2018 China Academic Journal Electronic Publishing House. All rights reserved. http://www.cnki.net

5-6 石油平衡表

单位: 万吨

项　　目	Item	1980	1985	1990
**可供量**	**Total Energy Available for Consumption**	**8794.5**	**9193.7**	**11435.0**
生产量	Output	10594.6	12489.5	13830.6
进口量	Imports	82.7	90.0	755.6
出口量(-)	Exports (-)	1806.2	3630.4	3110.4
年初年末库存差额	Stock Changes in the Year	-76.6	244.6	-40.8
**消费量**	**Total Energy Consumption**	**8757.4**	**9168.8**	**11485.6**
在消费量中:	Consumption by Sector			
1.农、林、牧、渔业	Agriculture, Forestry, Animal Husbandry and Fishery	814..9	758.7	1033.6
2.工业	Industry	6203.2	6171.4	7321.6
3.建筑业	Construction	175.2	292.2	327.3
4.交通运输、仓储和邮政业	Transport, Storage and Post	911.5	1176.4	1683.2
5.批发、零售业和住宿、餐饮业	Wholesale, Retail Trade and Hotel, Restaurants	29.0	38.1	77.6
6.其他	Others	481.7	506.1	757.8
7.生活消费	Residential Consumption	141.9	225.9	284.5
在消费量中:	Consumption by Usage			
(一) 终端消费	(I) Final Consumption	6311.0	7063.3	9304.7
#工业	Industry	3780.3	4462.0	5180.4
(二) 中间消费	(II) Intermediate Consumption			
(用于加工转换)	(Consumed in Transformation)	2183.6	1858.5	1630.4
发电	Power Generation	2065.4	1425.5	1234.4
供热	Heating		285.6	356.3
制气	Gas Production	36.7	34.5	39.7
炼油损失量	Losses in Petroleum Refining	81.5	112.9	295.8
(三) 损失量	(III) Other Losses	262.8	247.0	254.7
**平衡差额**	**Balance**	**37.1**	**24.9**	**-50.6**

注: 1.生产量为原油产量。
2.进口量包括境内轮船和飞机在境外的加油量; 出口量包括境外轮船和飞机在境内的加油量。

?1994-2018 China Academic Journal Electronic Publishing House. All rights reserved. http://www.cnki.net

Petroleum Balance Sheet

(10 000 tons)

1995	2000	2005	2010	2011	2012	2013	2014	2015
**16072.7**	**22631.4**	**32539.1**	**44178.4**	**45659.2**	**47864.7**	**49993.9**	**51861.8**	**55188.0**
15005.0	16300.0	18135.3	20301.4	20287.6	20747.8	20991.9	21142.9	21455.6
3673.2	9748.5	17163.2	29437.2	31593.7	33088.8	34264.8	36179.6	39748.6
2454.5	2172.1	2888.1	4079.0	4117.0	3884.3	4176.7	4213.9	5128.2
-151.0	-1245.0	128.8	-1481.2	-2105.0	-2087.6	-1086.1	-1246.8	-888.1
**16064.9**	**22495.9**	**32547.0**	**44101.0**	**45619.5**	**47797.3**	**49970.6**	**51814.4**	**55160.2**
1203.2	788.5	1451.7	1382.5	1466.3	1537.9	1650.3	1717.7	1733.4
9349.3	11248.5	14030.4	18555.0	17986.0	17753.2	17594.6	18217.5	18908.1
242.8	840.6	1502.2	2483.1	2581.8	2740.7	3090.6	3311.9	3507.5
2863.6	6399.0	10928.5	15079.3	16221.1	17863.6	18967.6	19546.9	20549.9
333.9	247.0	375.6	481.0	500.0	542.4	565.4	563.2	615.7
1390.3	1635.9	1974.2	2578.2	2880.5	3067.8	3349.7	3152.0	3683.3
682.0	1336.5	2284.4	3541.9	3983.9	4291.6	4752.4	5305.2	6162.2
13676.3	19950.1	29495.6	41243.4	43103.3	45080.7	47458.8	49134.0	52445.7
7095.5	8860.0	11107.5	15857.8	15579.9	15160.4	15235.4	15584.5	16229.7
2230.0	2352.9	2896.0	2663.3	2334.5	2534.6	2295.7	2570.0	2626.9
1358.5	1178.2	1306.4	385.3	319.8	292.4	265.1	254.1	265.5
399.9	427.0	429.1	593.1	525.7	493.5	448.2	521.3	493.2
51.6	25.9	14.4						
420.1	721.9	1146.1	1684.8	1489.1	1748.7	1582.4	1794.6	1868.2
158.6	192.9	155.4	194.4	181.7	182.0	216.1	110.3	87.6
**7.8**	**135.4**	**-7.9**	**77.4**	**39.7**	**67.4**	**23.3**	**47.4**	**27.8**

a) Data on output refer to the output of crude oil.

b) Data on imports include the petroleum consumed by the domestic airplanes and ships in refueling abroad.
Data on exports include the petroleum consumed by the oversea airplanes and ships in refueling in China.

?1994-2018 China Academic Journal Electronic Publishing House. All rights reserved. http://www.cnki.net

5-7 原油平衡表

单位：万吨

项　　目	Item	1980	1985	1990
**可供量**	**Total Energy Available for Consumption**	**9222.9**	**9516.5**	**11770.6**
生产量	Output	10594.6	12489.5	13830.6
进口量	Imports	36.6		292.3
出口量(-)	Exports (-)	1330.9	3003.0	2399.0
年初年末库存差额	Stock Changes in the Year	-77.4	30.0	46.7
**消费量**	**Total Energy Consumption**	**9205.0**	**9509.5**	**11762.2**
在消费量中:	Consumption by Sector			
1.农、林、牧、渔业	Agriculture, Forestry, Animal Husbandry and Fishery	8.0	0.8	0.2
2.工业	Industry	9112.0	9389.9	11653.8
3.建筑业	Construction	28.8	74.0	55.2
4.交通运输、仓储和邮政业	Transport, Storage and Post	50.1	44.3	52.1
5.批发、零售业和住宿、餐饮业	Wholesale, Retail Trade and Hotel, Restaurants		0.1	0.3
6.其他	Others	6.1	0.4	0.6
7.生活消费	Residential Consumption			
在消费量中:	Consumption by Usage			
(一) 终端消费	(I) Final Consumption	499.6	350.4	402.1
#工　业	Industry	429.7	254.9	333.4
(二) 中间消费	(II) Intermediate Consumption			
(用于加工转换)	(Consumed in Transformation)	8443.0	8929.7	11106.9
发　电	Power Generation	574.0	279.5	124.6
供　热	Heating		61.3	21.1
炼油	Petroleum Refineries	7869.0	8588.9	10961.2
(三) 油田原油损失量	(III) Losses in Oil Field for Crude Oil	262.4	229.4	253.2
**平衡差额**	**Balance**	**17.9**	**7.0**	**8.4**

?1994-2018 China Academic Journal Electronic Publishing House. All rights reserved. http://www.cnki.net

Crude Oil Balanc Sheet

(10 000 tons)

1995	2000	2005	2010	2011	2012	2013	2014	2015
**14794.9**	**21383.0**	**30089.2**	**42876.6**	**43961.0**	**46684.7**	**48670.9**	**51544.6**	**54093.5**
15004.4	16300.0	18135.3	20301.4	20287.6	20747.8	20991.9	21142.9	21455.6
1709.0	7026.5	12681.7	23768.2	25377.9	27102.7	28174.2	30837.4	33548.3
1822.7	1030.6	806.7	303.0	251.4	243.2	161.7	60.0	286.6
-95.8	-912.9	78.8	-890.0	-1453.0	-922.6	-333.4	-375.7	-623.8
**14886.4**	**21232.0**	**30088.9**	**42874.6**	**43965.8**	**46678.9**	**48652.2**	**51547.0**	**54088.3**
10.1								
14716.3	21052.1	29962.1	42716.6	43860.4	46559.5	48503.4	51502.1	54052.4
2.7	3.3							
156.8	175.1	126.9	158.0	105.4	119.4	148.7	44.9	35.9
0.5	0.2							
1390.3	1.4							
309.9	636.8	850.4	806.1	522.7	555.5	629.4	855.9	782.7
274.7	612.3	850.4	806.1	522.7	555.5	629.4	855.9	782.7
14419.4	20404.3	29084.8	41876.4	43266.1	45945.7	47810.5	50583.3	53218.4
61.6	85.0	41.3	3.7	11.3	10.9	10.4	8.9	12.5
4.4	14.0	3.0	3.3	4.6	1.4	3.4	7.0	6.7
14353.4	20305.3	29040.5	41869.4	43250.2	45933.5	47796.7	50567.4	53199.2
157.1	190.9	153.8	192.0	177.0	177.7	212.2	107.8	87.2
**-91.5**	**151.0**	**0.2**	**2.1**	**-4.8**	**5.8**	**18.7**	**-2.3**	**5.2**

?1994-2018 China Academic Journal Electronic Publishing House. All rights reserved. http://www.cnki.net

5-8 燃料油平衡表

单位: 万吨

项 目	Item	1980	1985	1990
**可供量**	**Total Energy Available for Consumption**	**3096.1**	**2848.0**	**3320.7**
生产量	Output	3142.0	2835.8	3267.9
进口量	Imports	39.0	70.0	167.3
出口量(-)	Exports (-)	45.4	64.9	97.2
年初年末库存差额	Stock Changes in the Year	-39.5	7.1	-17.3
**消费量**	**Total Energy Consumption**	**3073.7**	**2837.4**	**3367.8**
在消费量中:	Consumption by Sector			
1.农、林、牧、渔业	Agriculture, Forestry, Animal Husbandry and Fishery	2.3	3.1	2.9
2.工业	Industry	2937.4	2662.2	3091.7
3.建筑业	Construction	15.0	18.9	47.3
4.交通运输、仓储和邮政业	Transport, Storage and Post	109.0	144.1	208.2
5.批发、零售业和住宿、餐饮业	Wholesale, Retail Trade and Hotel, Restaurants	2.9	3.1	1.6
6.其他	Others	7.1	6.0	16.1
7.生活消费	Residential Consumption			
在消费量中:	Consumption by Usage			
(一) 终端消费	(I) Final Consumption	1617.9	1538.8	2042.6
#工 业	Industry	1481.6	1363.5	1766.5
(二) 中间消费	(II) Intermediate Consumption			
(用于加工转换)	(Consumed in Transformation)	1455.8	1296.1	1325.2
发 电	Power Generation	1419.1	1042.3	977.3
供 热	Heating		219.3	308.3
炼油再投入量	Petroleum Production			
制 气	Gas Production	36.7	34.5	39.6
(三)损失量	(III) Other Losses		2.5	
**平衡差额**	**Balance**	**22.4**	**10.6**	**-47.1**

?1994-2018 China Academic Journal Electronic Publishing House. All rights reserved. http://www.cnki.net

Fuel Oil Balance Sheet

(10 000 ton)

1995	2000	2005	2010	2011	2012	2013	2014	2015
**3717.3**	**3836.7**	**4237.3**	**3765.4**	**3667.6**	**3691.0**	**3926.1**	**4401.9**	**4632.8**
2960.8	2053.7	1767.4	2487.0	2281.8	2253.2	2775.9	3541.7	3963.0
859.1	1704.3	2883.9	2695.2	3097.4	3102.4	2734.3	2146.3	2068.2
68.6	57.9	427.6	1419.7	1685.6	1601.5	1493.7	1279.9	1402.1
-34.0	136.6	13.5	3.0	-26.0	-63.1	-90.4	-6.2	3.7
**3693.7**	**3872.8**	**4244.2**	**3758.0**	**3662.8**	**3683.3**	**3954.0**	**4400.5**	**4662.0**
8.4	0.4	0.7	1.1	1.3	2.0	2.0	1.3	0.9
3406.2	2975.1	2986.9	2377.3	2260.2	2241.7	2421.1	2835.7	3133.0
14.2	16.7	14.2	30.8	30.6	27.1	59.5	44.6	53.5
227.5	850.0	1201.0	1326.7	1345.2	1383.9	1429.0	1486.4	1439.5
6.6	11.6	27.5	8.6	9.3	8.7	19.1	17.4	19.0
30.8	19.0	13.9	13.5	16.2	19.9	23.4	15.1	16.1
2262.8	2741.4	2989.9	2403.2	2230.2	2072.8	2101.8	2103.0	2123.7
1975.3	1843.7	1732.6	1022.5	827.6	631.2	568.9	538.2	594.8
1430.9	1131.3	1254.3	1354.8	1432.6	1610.5	1852.1	2297.5	2538.3
1071.5	814.2	1068.7	123.9	60.9	43.1	47.3	35.6	31.5
307.8	291.2	171.1	201.3	183.8	187.3	167.2	164.9	165.1
			1029.6	1188.0	1380.0	1637.7	2097.0	2341.6
51.6	25.9	14.4						
**23.6**	**-36.1**	**-6.9**	**7.4**	**4.8**	**7.7**	**-27.8**	**1.4**	**-29.2**

?1994-2018 China Academic Journal Electronic Publishing House. All rights reserved. http://www.cnki.net

5-9 汽油平衡表

单位: 万吨

项　　目	Item	1980	1985	1990
**可供量**	**Total Energy Available for Consumption**	**999.4**	**1399.6**	**1884.1**
生产量	Output	1079.0	1471.9	2173.4
进口量	Imports		0.3	16.9
出口量(-)	Exports (-)	117.8	129.9	233.8
年初年末库存差额	Stock Changes in the Year	38.2	57.3	-72.4
**消费量**	**Total Energy Consumption**	**998.6**	**1396.3**	**1899.5**
在消费量中:	Consumption by Sector			
1.农、林、牧、渔业	Agriculture, Forestry, Animal Husbandry and Fishery	53.3	122.3	145.9
2.工业	Industry	273.2	451.3	589.3
3.建筑业	Construction	54.1	73.0	89.5
4.交通运输、仓储和邮政业	Transport, Storage and Post	404.9	477.4	620.1
5.批发、零售业和住宿、餐饮业	Wholesale, Retail Trade and Hotel, Restaurants	19.4	23.4	46.0
6.其他	Others	193.7	238.3	390.7
7.生活消费	Residential Consumption		10.6	18.0
**平衡差额**	**Balance**	**0.8**	**3.3**	**-15.4**

5-10 煤油平衡表

单位: 万吨

项　　目	Item	1980	1985	1990
**可供量**	**Total Energy Available for Consumption**	**359.0**	**383.2**	**350.9**
生产量	Output	398.5	405.3	392.5
进口量	Imports		15.2	26.1
出口量(-)	Exports (-)	46.8	46.0	55.5
年初年末库存差额	Stock Changes in the Year	2.3	8.7	-12.2
**消费量**	**Total Energy Consumption**	**365.9**	**385.5**	**350.9**
在消费量中:	Consumption by Sector			
1.农、林、牧、渔业	Agriculture, Forestry, Animal Husbandry and Fishery	2.3	3.3	3.1
2.工业	Industry	15.7	20.1	20.6
3.建筑业	Construction	0.8	1.3	1.3
4.交通运输、仓储和邮政业	Transport, Storage and Post	31.4	56.2	93.4
5.批发、零售业和住宿、餐饮业	Wholesale, Retail Trade and Hotel, Restaurants	0.2	0.1	0.6
6.其他	Others	216.7	182.9	127.3
7.生活消费	Residential Consumption	98.8	121.6	104.6
**平衡差额**	**Balance**	**-6.9**	**-2.3**	

?1994-2018 China Academic Journal Electronic Publishing House. All rights reserved. http://www.cnki.net

Gasoline Balance Sheet

(10 000 tons)

1995	2000	2005	2010	2011	2012	2013	2014	2015
**2902.0**	**3504.5**	**4855.3**	**6964.3**	**7597.9**	**8164.5**	**9369.5**	**9770.7**	**11385.0**
3051.6	4134.7	5433.6	7410.5	8117.9	8976.1	9834.0	11029.9	12103.6
15.9				2.9	0.5		3.4	17.0
193.1	467.7	559.7	517.0	406.0	291.7	468.7	507.5	589.3
27.6	-162.5	-18.6	70.8	-117.0	-520.3	4.2	-755.0	-146.3
**2909.6**	**3504.6**	**4854.9**	**6956.2**	**7595.9**	**8165.9**	**9366.4**	**9776.4**	**11368.5**
179.7	89.2	159.6	169.1	186.0	192.9	198.7	216.6	231.3
812.4	682.0	441.7	689.5	604.8	581.1	523.4	489.0	477.1
103.6	115.6	172.1	274.7	282.8	286.9	326.5	331.0	408.6
982.3	1527.8	2430.1	3274.9	3573.5	3778.0	4381.8	4665.0	5306.6
197.2	69.8	129.4	168.2	177.1	200.1	220.9	217.8	243.3
570.7	792.7	998.2	1166.2	1313.2	1460.5	1818.7	1738.1	2108.5
63.7	227.6	523.8	1213.7	1458.6	1666.5	1896.4	2118.8	2593.1
**-7.6**	**-0.1**	**0.4**	**8.1**	**1.9**	**-1.4**	**3.2**	**-5.7**	**16.6**

Kerosene Balance Sheet

(10 000 ton)

1995	2000	2005	2010	2011	2012	2013	2014	2015
**486.4**	**880.9**	**1070.0**	**1767.6**	**1821.7**	**1959.1**	**2189.1**	**2336.4**	**2732.6**
445.8	872.3	1006.5	1924.4	1922.4	2164.0	2523.9	3081.0	3658.6
115.7	322.5	476.1	726.1	875.1	877.3	945.2	721.0	716.4
62.4	256.3	447.6	870.5	966.8	1085.9	1280.6	1455.8	1626.6
-12.7	-57.6	35.0	-12.3	-9.0	3.7	0.6	-9.7	-15.8
**512.1**	**871.6**	**1076.8**	**1765.2**	**1816.7**	**1956.6**	**2164.1**	**2335.4**	**2663.7**
3.6	1.5	1.6	0.9	1.5	1.2	1.2	0.8	1.1
44.9	84.0	57.5	40.2	34.2	32.0	27.4	17.4	21.2
3.5	4.0		8.8	10.8	7.9	11.4	10.4	12.5
250.0	535.9	952.4	1601.1	1646.4	1787.1	1998.2	2216.0	2504.9
8.5	14.0	3.7	35.0	32.2	28.6	13.4	11.3	11.7
137.3	160.1	36.2	58.7	68.2	74.2	84.6	50.7	83.3
64.3	72.2	25.5	20.5	23.5	25.6	27.9	28.9	29.1
**-25.7**	**9.3**	**-6.8**	**2.4**	**5.0**	**2.4**	**25.0**	**1.0**	**68.9**

?1994-2018 China Academic Journal Electronic Publishing House. All rights reserved. http://www.cnki.net

5-11 柴油平衡表

单位: 万吨

项目	Item	1980	1985	1990
**可供量**	**Total Energy Available for Consumption**	**1663.2**	**1944.1**	**2689.4**
生产量	Output	1827.8	2023.2	2609.0
进口量	Imports	2.1	4.5	233.8
出口量(-)	Exports (-)	166.5	225.6	169.8
年初年末库存差额	Stock Changes in the Year	-0.2	142.0	16.4
**消费量**	**Total Energy Consumption**	**1663.2**	**1939.4**	**2691.7**
在消费量中:	Consumption by Sector			
1.农、林、牧、渔业	Agriculture, Forestry, Animal Husbandry and Fishery	749.0	629.2	881.5
2.工业	Industry	457.4	644.1	728.1
3.建筑业	Construction	76.5	125.0	133.0
4.交通运输、仓储和邮政业	Transport, Storage and Post	316.1	454.4	709.4
5.批发、零售业和住宿、餐饮业	Wholesale, Retail Trade and Hotel, Restaurants	6.5	10.9	22.5
6.其他	Others	57.7	74.0	217.0
7.生活消费	Residential Consumption			
在消费量中:	Consumption by Usage			
(一) 终端消费	(I) Final Consumption	1590.9	1827.4	2564.8
#工业	Industry	385.1	532.1	601.2
(二) 中间消费	(II) Intermediate Consumption			
(用于加工转换)	(Consumed in Transformation)	72.3	108.6	126.9
发电	Power Generation	72.3	103.6	124.5
供热	Heating		5.0	2.4
(三) 损失量	(III) Other Losses		3.4	
**平衡差额**	**Balance**		**4.7**	**-2.3**

5-12 液化石油气平衡表

单位: 万吨

项目	Item	1980	1985	1990
**可供量**	**Total Energy Available for Consumption**	**122.5**	**157.3**	**258.5**
生产量	Output	122.5	159.7	261.6
进口量	Imports			
出口量(-)	Exports (-)		1.9	1.1
年初年末库存差额	Stock Changes in the Year		-0.5	-2.0
**消费量**	**Total Energy Consumption**	**119.6**	**155.7**	**254.2**
在消费量中:	Consumption by Sector			
1.农、林、牧、渔业	Agriculture, Forestry, Animal Husbandry and Fishery			
2.工业	Industry	76.1	59.9	82.0
3.建筑业	Construction			1.0
4.交通运输、仓储和邮政业	Transport, Storage and Post			
5.批发、零售业和住宿、餐饮业	Wholesale, Retail Trade and Hotel, Restaurants		0.5	6.6
6.其他	Others	0.4	4.5	6.1
7.生活消费	Residential Consumption	43.1	90.8	158.5
**平衡差额**	**Balance**	**2.9**	**1.6**	**4.3**

?1994-2018 China Academic Journal Electronic Publishing House. All rights reserved. http://www.cnki.net

Diesel Oil Balance Sheet

(10 000 ton)

1995	2000	2005	2010	2011	2012	2013	2014	2015
**4404.2**	**6806.5**	**10972.6**	**14701.9**	**15626.2**	**16966.9**	**17105.9**	**17172.9**	**17353.6**
3972.6	7079.6	11090.2	14924.4	15689.7	17063.8	17275.7	17635.3	18007.9
645.3	51.9	61.0	190.2	243.3	99.9	35.3	55.0	71.5
169.5	77.5	170.9	490.2	228.8	205.7	294.4	423.9	731.3
-44.2	-247.6	-7.7	77.5	-78.0	8.9	89.4	-93.7	5.5
**4321.4**	**6806.2**	**10974.9**	**14699.0**	**15635.1**	**16966.0**	**17150.6**	**17165.3**	**17360.3**
1001.4	697.1	1286.3	1206.7	1271.9	1335.5	1441.5	1492.0	1492.9
1189.9	1696.5	1710.0	2090.0	1824.3	1747.7	1675.9	1595.3	1516.4
118.2	205.9	386.6	490.2	518.6	518.0	557.0	552.0	555.7
1246.6	3293.8	6169.4	8657.6	9485.2	10727.0	10920.5	11042.8	11162.8
103.6	95.9	116.0	196.6	212.3	229.0	233.5	230.1	257.7
645.7	638.7	900.1	1287.2	1428.1	1444.7	1339.8	1268.7	1384.2
16.1	178.4	406.4	770.7	894.7	964.1	982.5	984.4	990.7
4070.0	6578.6	10889.4	14655.2	15593.5	16900.7	17106.8	17127.0	17280.4
938.5	1468.8	1624.5	2046.2	1782.7	1682.3	1632.0	1557.0	1436.5
251.4	227.7	85.5	43.8	41.6	65.4	43.9	38.3	79.9
204.9	227.7	81.9	40.1	39.2	35.6	35.6	25.8	22.4
46.6		3.6	3.8	2.4	2.4	2.6	4.7	6.2
**82.7**	**0.3**	**-2.4**	**2.9**	**-8.9**	**0.9**	**-44.7**	**7.6**	**-6.7**

LPG Balance Sheet

(10 000 tons)

1995	2000	2005	2010	2011	2012	2013	2014	2015
**774.3**	**1396.2**	**2052.2**	**2323.8**	**2474.2**	**2496.0**	**2836.1**	**3292.7**	**4008.2**
540.8	916.6	1432.7	2092.3	2240.8	2268.7	2513.3	2705.8	2934.4
232.6	481.7	617.0	327.0	349.6	358.5	451.7	739.4	1244.0
7.1	1.6	2.7	93.0	119.1	128.2	126.9	144.4	144.2
8.0	-0.6	5.2	-2.5	3.0	-3.0	-2.0	-8.0	-26.0
**750.6**	**1389.7**	**2046.5**	**2321.9**	**2470.2**	**2482.2**	**2823.4**	**3289.8**	**3961.2**
0.1	0.4	3.5	4.7	5.6	6.4	6.8	7.1	7.2
192.5	426.1	534.4	586.8	661.1	621.0	705.1	835.0	1113.9
0.5	8.9	6.3	7.2	7.2	6.8	14.7	16.8	15.1
0.5	16.5	48.7	61.0	65.5	68.1	89.4	91.8	100.3
17.4	55.5	99.0	72.6	69.0	76.0	78.5	86.6	84.0
5.7	24.0	25.8	52.6	54.8	68.5	83.4	79.4	91.4
534.0	858.3	1328.7	1537.0	1607.2	1635.4	1845.6	2173.1	2549.3
**23.7**	**6.5**	**5.7**	**1.9**	**4.0**	**13.8**	**12.7**	**2.9**	**47.0**

?1994-2018 China Academic Journal Electronic Publishing House. All rights reserved. http://www.cnki.net

5-13 天然气平衡表

单位: 亿立方米

项目	Item	1980	1985	1990
**可供量**	**Total Energy Available for Consumption**	**142.7**	**129.3**	**153.0**
生产量	Output	142.7	129.3	153.0
进口量	Imports			
出口量(-)	Exports (-)			
年初年末库存差额	Stock Changes in the Year			
**消费量**	**Total Energy Consumption**	**140.6**	**129.3**	**152.5**
在消费量中:	Consumption by Sector			
1.农、林、牧、渔业	Agriculture, Forestry, Animal Husbandry and Fishery			
2.工业	Industry	131.4	109.6	120.2
3.建筑业	Construction	6.0	14.1	10.6
4.交通运输、仓储和邮政业	Transport, Storage and Post	0.7	0.8	1.9
5.批发、零售业和住宿、餐饮业	Wholesale, Retail Trade and Hotel, Restaurants			
6.其他	Others	0.5	0.5	1.2
7.生活消费	Residential Consumption	2.0	4.3	18.6
**平衡差额**	**Balance**	**2.1**		**0.5**

注: 从2010年起包括液化天然气数据。

5-14 电力平衡表

单位: 亿千瓦小时

项目	Item	1980	1985	1990
**可供量**	**Total Energy Available for Consumption**	**3006.3**	**4117.6**	**6230.4**
生产量	Output	3006.3	4106.9	6212.0
水电	Hydropower	582.1	923.7	1267.2
火电	Thermal Power	2424.2	3183.2	4944.8
核电	Nuclear Power			
风电	Wind Power			
进口量	Imports		11.1	19.3
出口量(-)	Exports (-)		0.4	0.9
**消费量**	**Total Energy Consumption**	**3006.3**	**4117.6**	**6230.4**
在消费量中:	Consumption by Sector			
1.农、林、牧、渔业	Agriculture, Forestry, Animal Husbandry and Fishery	270	317.4	426.8
2.工业	Industry	2471.9	3283.4	4873.3
3.建筑业	Construction	47.1	71.2	65.0
4.交通运输、仓储和邮政业	Transport, Storage and Post	26.5	63.4	105.9
5.批发、零售业和住宿、餐饮业	Wholesale, Retail Trade and Hotel, Restaurants	16.8	38.0	76.2
6.其他	Others	68.8	121.7	202.4
7.生活消费	Residential Consumption	105.2	222.5	480.8
在消费量中:	Consumption by Usage			
(一) 终端消费	(I) Final Consumption	2763.4	3813.3	5795.8
#工业	Industry	2229.0	2979.1	4438.7
(二) 输配电损失量	(II) Losses in Transmission	242.9	304.3	434.6

?1994-2018 China Academic Journal Electronic Publishing House. All rights reserved. http://www.cnki.net

Natural Gas Balance Sheet

(100 million cu.m)

1995	2000	2005	2010	2011	2012	2013	2014	2015
**179.5**	**240.6**	**463.5**	**1082.3**	**1333.0**	**1497.8**	**1706.6**	**1866.8**	**1925.0**
179.5	272.0	493.2	957.9	1053.4	1106.1	1208.6	1301.6	1346.1
			164.7	311.5	420.6	525.4	591.3	611.4
	31.4	29.7	40.3	31.9	28.9	27.5	26.1	32.5
**177.4**	**245.0**	**466.1**	**1080.2**	**1341.1**	**1497.0**	**1705.4**	**1868.9**	**1931.7**
			0.5	0.6	0.6	0.7	0.8	0.9
154.4	199.0	327.2	691.8	875.7	980.7	1129.1	1221.3	1234.5
0.3	0.8	1.5	1.2	1.3	1.3	2.0	1.9	2.2
1.6	8.8	38.0	106.7	138.3	154.5	175.8	214.4	237.6
0.6	3.4	10.8	27.2	33.6	38.7	39.3	46.6	51.3
1.2	0.6	9.1	26.0	27.1	32.9	35.6	41.3	45.4
19.4	32.3	79.4	226.9	264.4	288.3	322.9	342.6	359.8
**2.1**	**-4.4**	**-2.6**	**2.1**	**0.7**	**0.8**	**1.2**	**-2.1**	**-6.7**

a) Include the data of LNG since 2010.

Electricity Balance Sheet

(100 million kW•h)

1995	2000	2005	2010	2011	2012	2013	2014	2015
**10023.4**	**13472.7**	**24940.8**	**41936.5**	**47002.7**	**49767.7**	**54204.1**	**56381.8**	**58021.3**
10077.3	13556.0	25002.6	42071.6	47130.2	49875.5	54316.4	56495.8	58145.7
1905.8	2224.1	3970.2	7221.7	6989.5	8721.1	9202.9	10643.4	11302.7
8043.2	11141.9	20473.4	33319.3	38337.0	38928.1	42470.1	42686.5	42841.9
128.3	167.4	530.9	738.8	863.5	973.9	1116.1	1325.4	1707.9
			446.2	703.3	959.8	1412.0	1560.8	1857.7
6.4	15.5	50.1	55.5	65.6	68.7	74.4	67.5	62.1
60.3	98.8	111.9	190.6	193.1	176.5	186.7	181.6	186.5
**10023.4**	**13472.4**	**24940.3**	**41934.5**	**47000.9**	**49762.6**	**54203.4**	**56383.7**	**58020.0**
582.4	533.0	776.3	976.5	1012.9	1012.6	1026.9	1013.4	1039.8
7659.8	10004.6	18521.7	30871.8	34691.6	36232.2	39236.9	40802.7	41550.0
159.6	159.8	233.9	483.2	571.8	608.4	675.1	721.7	698.7
182.3	281.2	430.3	734.5	848.4	915.4	1000.9	1059.2	1125.6
199.5	418.7	752.3	1292.0	1503.1	1691.5	1876.9	1995.6	2122.0
234.2	623.2	1340.9	2451.8	2753.1	3083.6	3397.6	3615.0	3918.6
1005.6	1452.0	2884.8	5124.6	5620.1	6219.0	6989.2	7176.1	7565.2
9278.9	12535.7	23233.8	39366.3	44300.2	46866.5	51062.7	53283.8	55032.1
6915.3	9067.9	16815.2	28303.5	31990.9	33336.1	36096.2	37702.8	38562.1
744.5	936.7	1706.5	2568.2	2700.7	2896.2	3140.7	3099.9	2987.9

?1994-2018 China Academic Journal Electronic Publishing House. All rights reserved. http://www.cnki.net
